# Iron‐Catalyzed *trans*‐Hydrostannation of Terminal Alkynes

**DOI:** 10.1002/anie.202514794

**Published:** 2025-08-21

**Authors:** Soumyadeep Chakrabortty, Alois Fürstner

**Affiliations:** ^1^ Max‐Planck‐Institut für Kohlenforschung Mülheim/Ruhr 45470 Germany

**Keywords:** Alkynes, Hydrometalation, Iron catalysis, Organotin compounds, *trans*‐addition

## Abstract

The readily accessible iron complex [Cp*FeCl(tmeda)] is an effective catalyst for the highly regio‐ and stereoselective *trans*‐hydrostannylation of terminal alkyl alkynes, affording a type of alkenylstannanes that is difficult to make otherwise. The R_3_Sn‐ moiety is faithfully delivered to the terminal C‐atom, unless a propargylic or homo‐propargylic ─OH or ─NH_2_ group is present in the substrate, which (partly or fully) inverts the regiochemical course; this steering effect, however, can be switched off upon protection of the protic site. The *trans*‐addition likely starts by insertion of the [Cp*FeCl] fragment into the R_3_Sn─H bond, followed by a migratory insertion of the ligated alkyne into the Fe─Sn unit of the Fe(IV) species thus formed. This “modified Chalk–Harrod” mechanism is manifested in a stannylative cyclization of 1,6‐enyne derivatives, which has hardly any precedent either; a pathway via iron vinylidene intermediates can be excluded on the basis of deuterium labeling experiments.

## Graphical abstract

An easily accessible and cheap iron complex is capable of catalyzing highly regio‐ and stereoselective *trans*‐hydrostannation reactions of terminal alkyl alkynes as well as the stannylative cyclization of 1,6‐enynes. The addition likely follows a “modified Chalk–Harrod” mechanism; the intervention of iron vinylidene intermediates can be excluded.

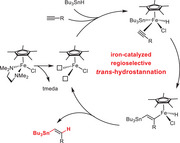

The addition of R_3_SnH across a terminal triple bond can afford three isomeric products **A**, **B**, and **C**. While the major advances in hydrostannation chemistry over the last decades have resulted in excellent methods for the formation of alkenylstannanes of type **A** and **B** with high levels of regio‐ and stereocontrol,^[^ ^1^, ^2^, ^3^, ^4^, ^5^, ^6^, ^7^ ^]^ similarly selective and broadly applicable catalytic entries into (*Z*)‐configured terminal stannanes of type **C** are largely missing. The most noteworthy access route relies on the addition of R_3_SnH to (terminal) alkynes with the aid of strong Lewis acids such as ZrCl_4_, HfCl_4_, B(C_6_F_5_)_3_, or trityl cation^[^ ^8^, ^9^, ^10^, ^11^ ^]^; though highly regio‐ and stereoselective, these reactions face notable limitations with regard to the functional groups that will subsist. Therefore, compounds of type **C** are often made indirectly with the help of stoichiometric organometallic chemistry. The examples shown in Scheme 1 illustrate some commonly employed strategies.^[^ ^12^, ^13^, ^14^, ^15^, ^16^, ^17^ ^]^ Once again, incompatibilities of numerous (polar) substituents with, e. g., transient organolithium (magnesium) reagents or reducing metal hydrides make multi‐step walk‐arounds involving protecting group manipulations necessary in many cases.

**Scheme 1:**
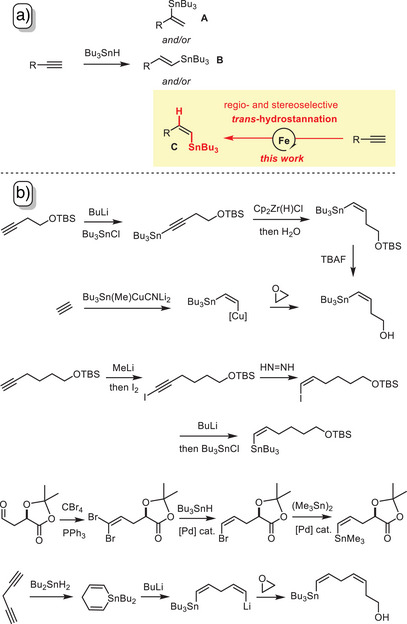
a) Hydrostannation of terminal alkynes and the envisaged selective approach to alkenylstannanes of type **C**; b) representative entries into functionalized compounds of this type; Cp = cyclopentadienyl; TBS = *tert*‐butyldimethylsilyl.

We conjectured that a transition metal catalyzed *trans*‐hydrostannation of a terminal alkyne would be an attractive alternative that might fill the gap in coverage while potentially exhibiting a better chemoselectivity profile. As part of our long‐term project concerning the development of conceptually new *trans*‐hydroelementation and *trans*‐hydrogenation chemistry,^[^ ^18^, ^19^ ^]^ our group has developed a highly efficient *trans*‐hydrostannation of internal alkynes catalyzed by [Cp*Ru] complexes^[^ ^20^, ^21^, ^22^, ^23^ ^]^; this transformation has already served numerous natural product total syntheses remarkably well.^[^ ^24^, ^25^, ^26^, ^27^, ^28^, ^29^, ^30^, ^31^, ^32^, ^33^, ^34^, ^35^, ^36^ ^]^ When applied to terminal alkynes, however, the R_3_Sn‐ group is delivered to the internal position, giving rise to products of type **A**.^[^ ^20^, ^37^ ^]^

A change in catalyst might allow the desired isomer **C** to be formed selectively. This expectation seemed justified in view of the many significant advances in the field of hydroelementation of terminal alkynes. Although not limited to, catalysts based on earth‐abundant transition metals play an increasingly important role in this context, not least because they promise reactivity profiles and selectivity patterns not (easily) encountered in the realm of the noble metals.^[^ ^38^, ^39^, ^40^ ^]^ In addition to this conceptual aspect, base metal catalysts are attractive for their sustained availability, low price and (often) benign character^[^ ^41^, ^42^ ^]^; their affinity to N‐, O‐, and C‐based ligands can lead to additional cost and labor savings as well as to environmental benefits.

This analysis is particularly true for iron‐based systems, which are rightfully becoming a focal point of contemporary catalysis research despite the many inherent challenges that they pose.^[^ ^43^, ^44^, ^45^, ^46^, ^47^, ^48^ ^]^ More specifically, iron‐based catalyst systems have recently been disclosed which allow for intriguing regiocontrol over the course of addition of R_3_SnH across a (terminal) alkyne (Scheme 2). While **Fe**‐**1** and **Fe**‐**3** afford the formal Markovnikov products **A**, their close relatives **Fe**‐**2** and **Mn**‐**1**, respectively, furnish the regio‐complementary alkenylstannanes **B**; no matter which direction is taken, however, a net *cis*‐addition was observed.^[^ ^49^, ^50^, ^51^, ^52^, ^53^ ^]^ Canonical *cis*‐delivery also dominates the closely related field of iron‐catalyzed hydrosilylation and hydroboration reactions,^[^ ^54^, ^55^, ^56^, ^57^ ^]^ even though a few cases of stereo‐complementary *trans*‐additions are documented: specifically, the hydrosilylation of internal alkynes catalyzed by complex **Fe**‐**4** can proceed either *cis*‐ or *trans*‐selectively depending on the size of the substituents on the reaction partners.^[^ ^58^ ^]^ Along similar lines, a temperature‐dependent stereochemical swap was observed in hydroboration reactions catalyzed by complex **Fe**‐**5**.^[^ ^59^ ^]^ Arguably most encouraging is the strict *trans*‐hydroboration of terminal alkynes catalyzed by the non‐classical polyhydride pincer complex **Fe**‐**6**,^[^ ^60^, ^61^ ^]^ as well as the *trans*‐hydrogermylation of internal and terminal alkynes effected by complex **Fe**‐**7**.^[^ ^62^ ^]^ The identical stereochemical outcome must not obscure the fact that the hydroelementation reactions catalyzed by **Fe**‐**6** and **Fe**‐**7** almost certainly follow fundamentally different mechanisms.

**Scheme 2:**
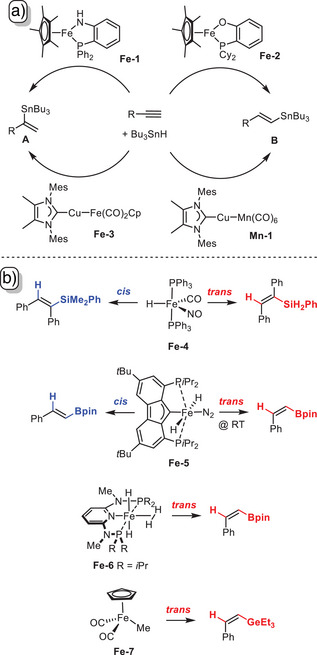
Prior art. a): Representative examples of iron catalyzed regiodivergent, yet *cis*‐selective hydrostannations of terminal alkynes; b): iron‐catalyzed stereodivergent or exclusively *trans*‐selective hydroelementations of terminal alkynes; Cy = cyclohexyl, Mes = mesityl.

In case of **Fe**‐**7**, elevated temperatures are necessary to initiate the formation of the active species (Scheme 3). This process commences with a migratory insertion leading to the formation of the acyl complex **A**, followed by oxidative addition of R_3_GeH to give the Fe(+4) complex **B**, in which the hydride and the CO‐ligand presumably reside *trans* to each other; reductive elimination then affords **C** featuring an open coordination site as necessary for substrate binding.^[^ ^62^ ^]^ Unfortunately, attempts at extending the use of complex **Fe**‐**7** to the desired *trans*‐hydrostannation of terminal alkynes were largely met with failure in our hands; rather, mixtures comprising at least three different tin‐containing species were obtained when the reaction was performed in either toluene or THF at 60 °C.

**Scheme 3:**
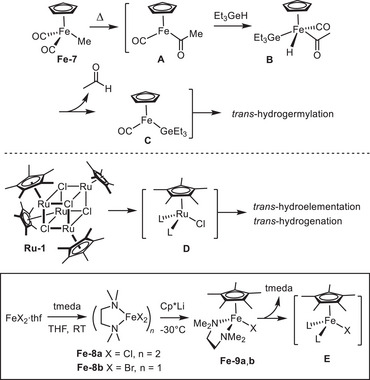
The mode of initiation of catalysts **Fe**‐**7** and **Ru**‐**1** able to effect certain *trans*‐addition reactions suggests that [Cp*FeX(tmeda)] (**Fe**‐**9**) might be another suitable candidate;Cp* = pentamethylcyclopentadienyl;tmeda = tetramethylethylenediamine.

The handicap of **Fe**‐**7** resulting from the high temperature might be overcome by an iron catalyst bearing more labile ligands. This notion is reinforced by the way the ruthenium complex [Cp*RuCl]_4_ (**Ru**‐**1**) operates, which is able to effect numerous *trans*‐addition reactions, including the *trans*‐hydrostannation of internal alkynes mentioned above (Scheme 3).^[^ ^18^, ^20^, ^21^, ^22^, ^23^, ^63^ ^]^ This complex readily disassembles in solution to form a fragment of type **D** in readiness for binding of the alkyne and the R_3_SnH reagent to the two “vacant” sites. Since the iron analogue of the tetrameric complex **Ru**‐**1** is unknown, we turned our attention to complex **Fe**‐**9** as a functional surrogate, which should afford a metal fragment of type **E** under fairly mild conditions. In this context, it is important to note that the Cp ring of **Fe‐7** was deliberately replaced by Cp*, which was deemed advantageous for several reasons: i) as a stronger electron donor, Cp* should facilitate oxidative addition of R_3_SnH to the Fe(+2) center and, at the same time, stabilize the resulting Fe(+4) intermediate; ii) spectroscopic data in the literature suggest that the stoichiometric migratory insertion of the ligated ethylene into the Fe─Si bond of complex [Cp*Fe(CO)(C_2_H_4_)SiMe_3_] is considerably faster than that in its sibling [CpFe(CO)(C_2_H_4_)SiMe_3_]^[^ ^64^ ^]^; if the same holds true for the insertion of an alkyne into a Fe─Sn unit, the choice of a Cp* ligand should benefit the projected catalytic process; iii) piano‐stool iron half‐sandwich complexes are prone to convert into the corresponding ferrocene derivatives; the more bulky Cp* ring disfavors this process,^[^ ^65^, ^66^ ^]^ which is detrimental for catalyst turnover.

With these considerations in mind, we turned our attention to [Cp*FeX(tmeda)] (**Fe**‐**9a**,**b**) as a potential candidate for the envisaged *trans*‐hydroelementation of terminal alkynes.^[^ ^67^ ^]^ As the tmeda ligand is not overly tightly bound, it might vacate the two binding sites necessary for the incoming substrates.^[^ ^68^ ^]^ **Fe**‐**9** is readily prepared by first reacting [FeX_2_⋅thf] with excess tmeda in THF to furnish a clear, pale green solution of [(tmeda)FeX_2_]_n_ (**Fe**‐**8**; X═Cl, *n* = 2; X═Br, *n* = 1); Cp*Li is then added at −30 °C and the resulting mixture stirred for 3 d at this temperature, after which **Fe**‐**9a**,**b** can be isolated in good yield in form of yellow‐greenish crystals (for improved X‐ray structures of these complexes, see the Supporting Information).^[^ ^69^ ^]^ Although we failed to effect *trans*‐hydroboration, *trans*‐hydrosilylation and even *trans*‐hydrogermylation reactions with the aid of **Fe**‐**9a** under a variety of conditions, the weaker Bu_3_Sn─H bond proved amenable to addition across the triple bond of but‐3‐yn‐1‐ylbenzene (**1**) as the model substrate (Scheme 4). Since the iron complex is not overly stable when exposed to Bu_3_SnH and/or the alkyne,^[^ ^70^ ^]^ however, best results were obtained by adding a mixture of the reaction partners to a solution of the catalyst in freshly distilled toluene over the course of ca. 15–30 min; the reaction then proceeds to full conversion within ca. 2 h at ambient temperature.^[^ ^71^ ^]^ The limited stability of **Fe**‐**9** is also the main reason why a loading of ≥ 5 mol% was necessary to render the method robust. Under these conditions, compound **2** as the product of an almost perfectly regio‐ and stereoselective net *trans*‐hydrostannation was isolated in well‐reproducible 85% yield; complex **Fe**‐**9b** (X═Br) is similarly effective (82%). The reaction also scaled well, providing unaltered selectivity and a fully consistent yield when performed on 2 mmol scale (86%, 717 mg). Likewise, Ph_3_SnH could be used instead of Bu_3_SnH to furnish product **2b** without any significant loss in yield or selectivity. Control experiments with an assortment of other iron complexes (**Fe**‐**8a**,^[^ ^72^ ^]^ **Fe**‐**10**,^[^ ^65^, ^73^ ^]^ **Fe**‐**11**,^[^ ^74^ ^]^ **Fe**‐**12**,^[^ ^75^ ^]^ [(dppe)FeCl_2_] (**Fe**‐**13**)^[^ ^74^, ^76^ ^]^) showed **Fe**‐**9a**,**b** to be uniquely effective.

**Scheme 4:**
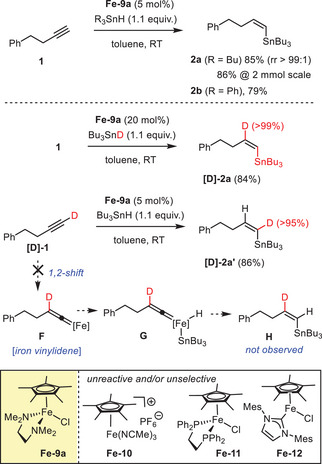
Model reaction, scale‐up, and confirmation of the stereochemical course by deuterium labeling experiments.

The stereochemical course of the addition was rigorously confirmed. To this end, **1** was reacted with Bu_3_SnD under otherwise identical conditions to give the isotopologe **[D]‐2a** exclusively, in which the D‐atom resides at the internal position. As a corollary, the reaction of **[D]‐1** with Bu_3_SnH should furnish **[D]‐2a’**, in which the label is retained at the terminus; this is indeed the case. These results rule out that the observed *trans*‐addition proceeds via a metal vinylidene **F** formed by 1,2‐hydride(deuterium) shift followed by an actual 1,1‐addition step (**G**), as this pathway would entail a different labeling pattern (**H**); this information is relevant as reactions of terminal alkynes passing through vinylidene intermediates are well precedented, including *trans*‐hydroboration.^[^ ^19^, ^77^, ^78^ ^]^ It is equally noteworthy that addition of either 3,5‐di‐*tert*‐butyl‐4‐hydroxytoluene (BHT) or 1,1‐diphenylethylene (DPE) to the mixture leaves the reaction undisturbed, which speaks against a radical mechanism (for details, see the Supporting Information).

The scope of the iron‐catalyzed *trans*‐hydrostannation is broad and the functional group tolerance high as evident from the examples compiled in Scheme 5. For all terminal alkynes that are unbranched at the position adjacent to the triple bond, virtually exclusive formation of the corresponding “*trans‐anti*‐Markovnikov” alkenylstannane isomer of type **C** was observed (rr ≥ 97:3). Polar and apolar functional groups exert no noticeable influence on this outcome as long as they are remote from the reactive triple bond; the list includes i) unprotected (primary or allylic) alcohols; ii) different types of ethers (including acid‐sensitive trityl groups as well as allyl ethers that could succumb to oxidative addition to a transition metal); iii) potentially reducible groups (primaryl alkyl chloride, esters, nitrile, ketone, and phthalimide); iv) various (hetero)aromatic rings; v) potential donor sites (pyridine and nitrile); vi) remote internal or terminal double bonds that would be endangered if the process were a radical addition. It is of note that many of these substituents might not subsist if one of the methods for the preparation of alkenylstannanes of type **C** mentioned in the Introduction had to be used. The fact that the rather labile chiral center of (*S*)‐naproxen passes uncompromised further attests to the mildness of the method (see product **19**). The exquisite regioselectivity manifested in these examples, however, is partly lost if the substrate is branched α to the triple bond (see the Insert). Phenyl‐acetylene denotes another limitation as a mixture of the *cis*‐ and *trans*‐adducts was formed under the standard conditions. All internal alkynes tested (tolane, 1‐phenyl‐1‐propyne, 5‐decyne) invariably resulted in canonical *cis*‐hydrostannation and were therefore not investigated in more detail.

**Scheme 5:**
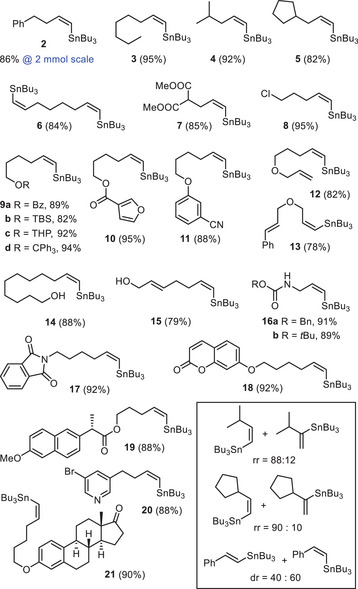
Scope and limitations of the *trans*‐hydrostannation of terminal alkynes catalyzed by the iron complex **Fe**‐**9a** (5 mol%); all reactions were performed with Bu_3_SnH (1.1 equiv.) in toluene at ambient temperature. The insert shows cases in which product mixtures were obtained.

Unprotected –OH groups at the propargylic or homo‐propargylic position exert a massive directing effect on the *trans*‐hydrostannation (silylation) of internal alkynes catalyzed by [Cp*RuCl]_4_ (**Ru**‐**1**); the effect is rooted in strong interligand hydrogen bonding between the protic site and the polarized Ru─Cl unit.^[^ ^21^, ^22^ ^]^ In view of this precedent, it was expected that reactions catalyzed by [Cp*FeX] might be affected in a similar way (Scheme 6). Indeed, significant amounts of the Markovnikov product **24** carrying the tin residue at the internal position were observed when **22** (R═H) was subjected to the standard reaction conditions but using a higher catalyst loading (20 mol%). Unlike the ruthenium series,^[^ ^21^ ^]^ the selectivity is *higher* when **Fe‐9b** (X═Br) is used. Truly enabling is the fact that the steering effect is *more* pronounced in homopropargylic derivatives, which is also in contrast to the trend observed for [Cp*RuCl]^[^ ^21^, ^22^ ^]^; in any case, the hydrostannation of **26** (R═H) resulted in the virtually exclusive formation of **27**. Pertinent control experiments using the combinations **26**/Bu_3_SnD and **[D]‐26**/Bu_3_SnH furnished products **[D]‐27a** and **[D]‐27b**, respectively, which unmistakably prove the *trans*‐addition mode while, once again, excluding a vinylidene pathway. A number of other homopropargylic substrates resulted in the same stannylation pattern (**32**–**37**). Even the proparylic amine derivative **29** (R^1^ = R^2^ = H) gave the “*trans*‐Markovnikov” derivative **30** as the only detectable isomer in the crude product (^1^H NMR).

**Scheme 6:**
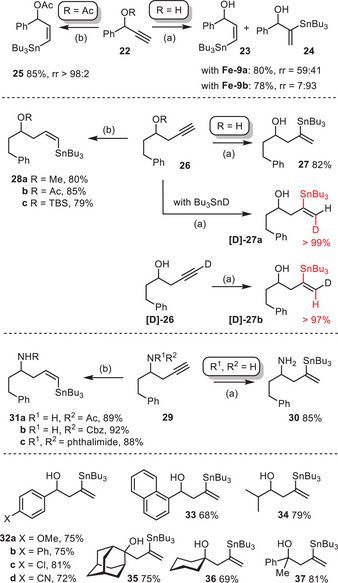
Steering effect of protic sites at the propargylic or homopropargylic position; a) **Fe‐9a** (20 mol%), Bu_3_SnH (1.1 equiv.), toluene, RT; b) **Fe‐9a** (5 mol%), Bu_3_SnH (1.1 equiv.), toluene, and RT.

In analogy to the ruthenium series, the effect is innately linked to the presence of a free –OH group. Upon protection, the iron‐catalyzed reaction reverts to the usual course to furnish alkenylstannanes of type **C**, independent of whether a –OMe, −OAc or ─OTBS group was chosen (see **28a**‐**c**); the same is true for the homopropargylic –NH_2_ substituent, which loses its massive directing ability upon conversion into a non‐protic imide or even into an amide or urethane, although the latter still contain a protic site (cf. **31a‐c**; see also the N‐protected propargylic amine derivatives **16a**,**b** in Scheme 5). The ability to access two different regioisomeric products (compare **27**/**28**, **30**/**31**) with outstanding selectivity each simply by choosing a protected or an unprotected substrate is another asset of the new iron‐catalyzed *trans*‐hydrostannation reaction.

The stannylative cyclization of 1,6‐enynes shows that the iron catalyst is able to engage a neighboring π‐system into the actual bond forming event (Scheme 7). While remote and/or more highly substituted alkenes remain unaffected (see **41** and products **12**, **13**, **15** (Scheme 5)), compounds **38** were converted into the corresponding cyclic alkenylstannanes **39** in good yield as a single diastereoisomer each. A set of characteristic NOE's allowed the configuration of the exocyclic double bond to be assigned. This outcome bears valuable mechanistic information: the fact that the Bu_3_Sn‐group points away from the methyl branch implies that the cyclization does not pass through a metallacyclic intermediate of type **K** as is the case in the cycloisomerization or cycloaddition of enynes catalyzed by low‐valent iron complexes.^[^ ^79^, ^80^, ^81^, ^82^, ^83^ ^]^ Rather, the reaction likely starts with an ordinary *trans*‐hydrostannation of the alkyne with formation of an alkenyliron intermediate **I**, which then engages the presumably ligated olefin in carbometalation to give an alkyliron species **J**; reductive elimination releases product **39** and completes the catalytic cycle. This scenario is in full accord with the isotope labelling experiments furnishing **[D]‐39d**,**e**. The fact that the truncated model compound **42** is reactive while **44** is not shows unmistakably that the stannylative cyclization starts at the triple bond. This particular course of the reaction and the resulting products of type **39** have hardly any precedent^[^ ^84^, ^85^, ^86^, ^87^ ^]^; in any case, the outcome stands in striking contrast to palladium‐catalyzed stannylative cyclizations of 1,6‐enynes in which the R_3_Sn − moiety ends up on the alkyl branch rather than on the olefinic site.^[^ ^88^ ^]^

**Scheme 7:**
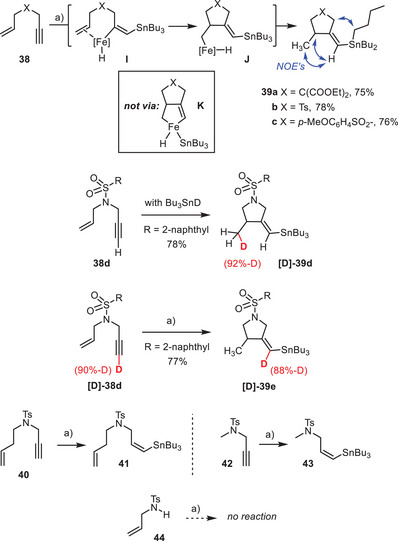
Stannylative cyclization of 1,6‐enynes and control experiments; a) **Fe‐9a** (20 mol%), Bu_3_SnH (1.2 equiv.), toluene, RT; Ts = tosyl.

This analysis implies that the R_3_Sn‐ moiety is delivered to the unsaturated substrate prior to the H‐atom. If the iron‐catalyzed *trans*‐hydrostannation commences by insertion of the terminal alkyne into an Fe─Sn rather than into an Fe─H bond, it follows a “modified Chalk‐Harrod” mechanism.^[^ ^89^ ^]^ Moreover, migratory insertion is supposed to be the turnover‐limiting step because the oxidative addition of a R_3_Sn−H bond to CpFe(+2) species is known to be fast.^[^ ^90^, ^91^, ^92^ ^]^ In any case, a KIE = 1 (kinetic isotope effect) was recorded, which precludes that the activation of the Sn−H/Sn─D bond by the iron catalyst or the formation of the C─H(D) bond by reductive elimination from an intermediate of type **N** is the limiting step (for details, see the Supporting Information). With this information in mind, we propose that complex **Fe**‐**9a** first loses tmeda to liberate two vacant sites, to which the alkyne substrate and the tin reagent will bind (Scheme 8). Rapid oxidative addition then affords complex **M**, in which the substituent R is pointing away from the bulky tin residue; importantly, this orientation entails stannation of the terminus, as experimentally observed. The alkyne in **M** is likely positioned *cis* to the R_3_Sn‐ group but *trans* to the hydride ligand; this notion gains credence by inspection of a number of X‐ray structures of CpFe(+4) carbonyl complexes described in the literature, which were formed by oxidative addition of R_3_SnH to related Fe(+2) fragments; **Fe**‐**13** is representative. In this complex, the hydride resides *trans* to the CO ligand, whereas the R_3_Sn‐ groups are located *cis* to it (see also the proposed mode of initiation of complex **Fe**‐**7** via **B** as shown in Scheme 3).^[^ ^90^ ^]^ Since an alkyne is also a good π‐acceptor ligand, it is reasonable to assume that it will take the very same position as the CO group in **Fe‐13** and is hence poised for migratory insertion into the Fe─Sn rather than the Fe─H bond, which results in a “modified Chalk–Harrod” pathway. Whether the *trans*‐configured organoiron intermediate **N** is reached by isomerization of *cis*‐configured **O** via a transient dipolar carbene **P** ^[^ ^93^, ^94^ ^]^ or if it is formed by passing through a metallacyclopropene intermediate **Q** has yet to be elucidated.^[^ ^95^, ^96^, ^97^, ^98^, ^99^ ^]^

**Scheme 8:**
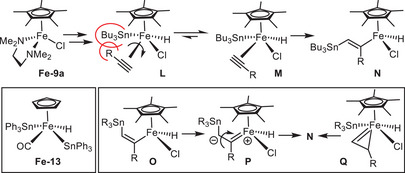
Mechanistic considerations in line with a “modified Chalk–Harrod” mechanism.

This and related questions are subject to ongoing studies in our laboratory, which aim at extending metal‐catalyzed hydroelementation reactions and related transformations at large. In any case, the new method exploiting the reactivity of the well accessible, cheap and benign iron complex **Fe**‐**9** gives access to organostannane derivatives that are hard to make otherwise. This favorable outcome further illustrates that attempts at harnessing the chemistry of earth‐abundant transition metals for catalytic purposes can be truly rewarding.

## Conflict of Interests

The authors declare no conflict of interest.

## Supporting information



Supporting Information

Supporting Information

## Data Availability

The data that support the findings of this study are available in the Supporting Information of this article.
