# Supplementary material for: Iron‐Catalyzed trans‐Hydrostannation of Terminal Alkynes
Source: Angew Chem Int Ed Engl. 2025 Aug 21;64(41):e202514794. doi: 10.1002/anie.202514794 (PMC12501755; doi:10.1002/anie.202514794)
Supplement: Supplementary file 1 — Supporting Information [file ANIE-64-e202514794-s001.pdf]

SUPPORTING INFORMATION

Iron-Catalyzed *trans*-Hydrostannation of Terminal Alkynes

Soumyadeep Chakrabortty and Alois Fürstner

Max-Planck-Institut für Kohlenforschung, 45470 Mülheim/Ruhr, Germany

Email: fuerstner@kofo.mpg.de

Supporting Crystallographic Information

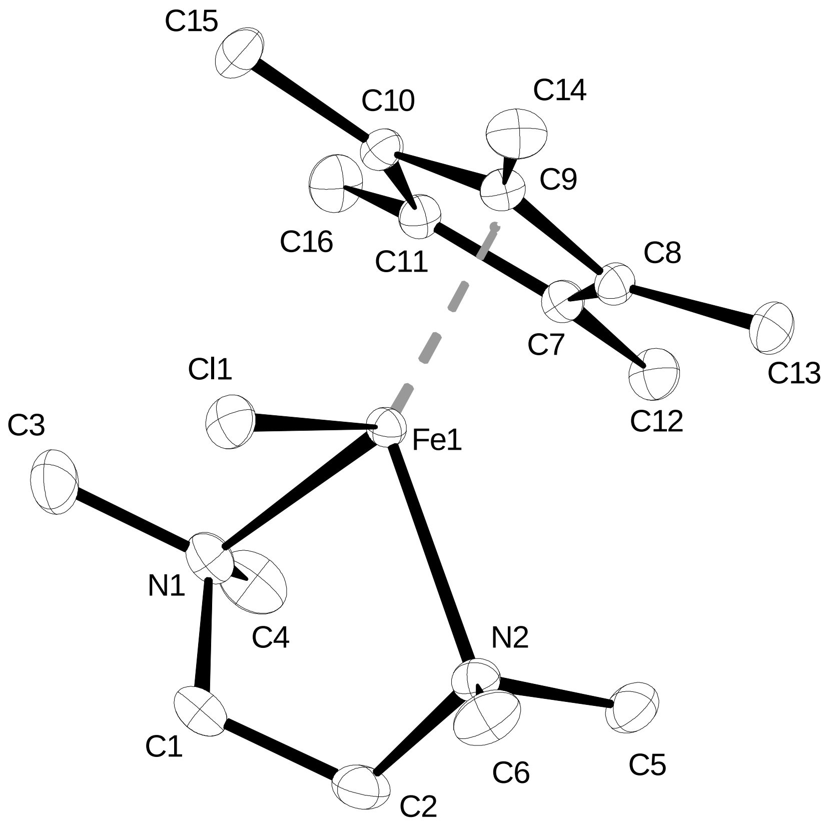

Figure S1. The asymmetric unit of complex **Fe-9a**

X-ray Crystal Structure Analysis of Fe-9a: C_{16} H_{31} Cl Fe N_2, M_r = 342.73 g mol^{-1}, yellow needle, crystal size 0.266 x 0.061 x 0.061 mm^3, monoclinic, space group $P2_1$n [14], a = 10.3523(9) Å, b = 14.2883(13) Å, c = 12.0961(11) Å, β = 91.066(3)°, V = 1788.9(3) Å^3, T = 100(2) K, Z = 4, D_{calc} = 1.273 g·cm^3, λ = 0.71073 Å, μ(Mo-K_α) = 0.986 mm^{-1}, numerical correction ($T_{\min}$ = 0.80, $T_{\max}$ = 1.00), Bruker-AXS D8 VENTURE with APEX-III detector and IµS Diamond Mo-anode X-ray source, 2.206 < θ < 33.787°. 856148 measured reflections, 7165 independent reflections, 6551 reflections with $I > 2\sigma(I)$, R_{int} = 0.0742. The structure was solved by *SHELXT* and refined by full-matrix least-squares (*SHELXL*) against F^2 to R_1 = 0.0208 [$I > 2\sigma(I)$], wR_2 = 0.0596 [all data] with 190 parameters, 0 restraints.

Complete .cif-data of the compound are available under **CCDC-2469934**.

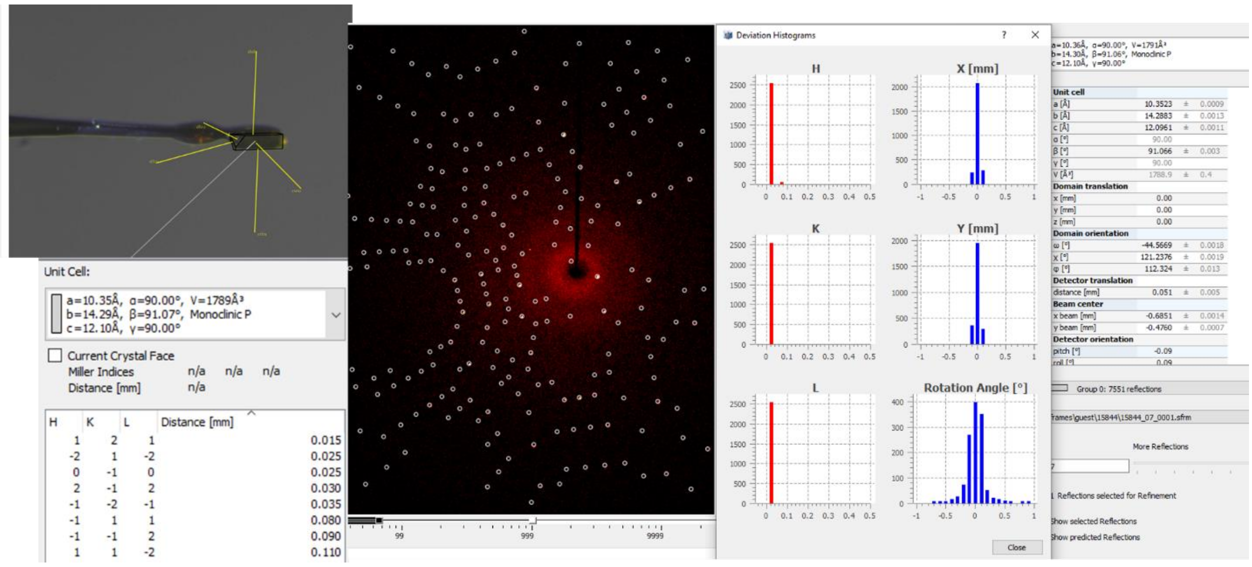

Figure S2. Crystal faces and unit cell determination/refinement of **Fe-9a**

INTENSITY STATISTICS FOR DATASET

Resolution	#Data	#Theory	%Complete	Redundancy	Mean I	Mean I/s	Rmerge	Rsigma
Inf - 2.65	113	115	98.3	149.72	65.12	151.75	0.0387	0.0231
2.65 - 1.75	263	263	100.0	174.92	48.49	163.11	0.0351	0.0063
1.75 - 1.39	376	376	100.0	188.78	27.04	148.06	0.0408	0.0047
1.39 - 1.21	370	370	100.0	185.48	14.81	124.17	0.0592	0.0054
1.21 - 1.09	411	411	100.0	156.77	15.26	112.42	0.0623	0.0060
1.09 - 1.02	324	324	100.0	140.71	12.68	93.58	0.0669	0.0068
1.02 - 0.96	369	369	100.0	132.50	11.88	90.52	0.0752	0.0075
0.96 - 0.91	382	382	100.0	123.95	7.67	70.76	0.0926	0.0092
0.91 - 0.87	363	363	100.0	121.61	6.38	63.49	0.1080	0.0104
0.87 - 0.83	450	450	100.0	115.51	5.29	56.65	0.1216	0.0119
0.83 - 0.80	374	374	100.0	111.12	4.97	52.59	0.1316	0.0130
0.80 - 0.78	309	309	100.0	107.70	4.89	49.90	0.1367	0.0137
0.78 - 0.75	502	502	100.0	102.34	4.82	47.73	0.1394	0.0145
0.75 - 0.73	381	381	100.0	96.06	3.88	38.84	0.1622	0.0175
0.73 - 0.72	205	205	100.0	94.30	4.35	40.21	0.1543	0.0168
0.72 - 0.70	464	464	100.0	93.89	4.11	38.24	0.1614	0.0179
0.70 - 0.68	489	489	100.0	89.58	3.44	32.55	0.1796	0.0208
0.68 - 0.67	283	283	100.0	88.57	2.52	27.31	0.2189	0.0260
0.67 - 0.66	300	300	100.0	81.55	2.66	26.78	0.2198	0.0272
0.66 - 0.64	686	696	98.6	71.51	2.48	22.26	0.2354	0.0568
0.74 - 0.64	2631	2641	99.6	85.35	3.24	30.74	0.1867	0.0277
Inf - 0.64	7414	7426	99.8	117.70	9.86	66.62	0.0728	0.0114

Table S1. Crystal data and structure refinement of **Fe-9a**

Identification code	15844	
Empirical formula	$C_{16} H_{31} Cl Fe N_2$	
Color	yellow	
Formula weight	342.73 g · mol^{-1}	
Temperature	100(2) K	
Wavelength	0.71073 Å	
Crystal system	MONOCLINIC	
Space group	**$P2_1/n$, (no. 14)**	
Unit cell dimensions	a = 10.3523(9) Å	α= 90°.
	b = 14.2883(13) Å	β= 91.066(3)°.
	c = 12.0961(11) Å	γ = 90°.
Volume	1788.9(3) Å3	
Z	4	
Density (calculated)	1.273 Mg · m^{-3}	
Absorption coefficient	0.986 mm^{-1}	
F(000)	736 e	
Crystal size	0.266 x 0.061 x 0.061 mm3	
θ range for data collection	2.206 to 33.787°.	
Index ranges	$-16 \leq h \leq 16, -22 \leq k \leq 22, -18 \leq l \leq 18$	
Reflections collected	856148	
Independent reflections	7165 [R_{int} = 0.0742]	
Reflections with $I>2\sigma(I)$	6551	
Completeness to θ = 25.242°	100.0 %	
Absorption correction	Numerical	
Max. and min. transmission	1.00 and 0.80	
Refinement method	Full-matrix least-squares on F^2	
Data / restraints / parameters	7165 / 0 / 190	
Goodness-of-fit on F^2	1.105	
Final R indices [$I>2\sigma(I)$]	R_1 = 0.0208	wR^2 = 0.0563
R indices (all data)	R_1 = 0.0247	wR^2 = 0.0596
Largest diff. peak and hole	0.6 and -0.2 e · $Å^{-3}$	

Table S2. Bond lengths [Å] and angles [°] of **Fe-9a**

Fe(1)-Cl(1)	2.3566(2)	Fe(1)-N(1)	2.2306(7)
Fe(1)-N(2)	2.3042(6)	Fe(1)-C(7)	2.3941(7)
Fe(1)-C(8)	2.3104(7)	Fe(1)-C(9)	2.3204(7)
Fe(1)-C(10)	2.4045(7)	Fe(1)-C(11)	2.4604(7)
N(1)-C(1)	1.4860(10)	N(1)-C(3)	1.4782(11)
N(1)-C(4)	1.4797(11)	N(2)-C(2)	1.4882(10)
N(2)-C(5)	1.4773(10)	N(2)-C(6)	1.4837(10)
C(1)-C(2)	1.5164(12)	C(7)-C(8)	1.4327(9)
C(7)-C(11)	1.4277(9)	C(7)-C(12)	1.5063(10)
C(8)-C(9)	1.4254(9)	C(8)-C(13)	1.5036(10)
C(9)-C(10)	1.4248(9)	C(9)-C(14)	1.4999(10)
C(10)-C(11)	1.4207(9)	C(10)-C(15)	1.5012(10)
C(11)-C(16)	1.5054(10)		
Cl(1)-Fe(1)-C(7)	150.313(17)	Cl(1)-Fe(1)-C(10)	96.110(18)
Cl(1)-Fe(1)-C(11)	127.309(18)	N(1)-Fe(1)-Cl(1)	94.589(19)
N(1)-Fe(1)-N(2)	78.99(2)	N(1)-Fe(1)-C(7)	107.83(2)
N(1)-Fe(1)-C(8)	142.86(2)	N(1)-Fe(1)-C(9)	148.21(2)
N(1)-Fe(1)-C(10)	113.22(2)	N(1)-Fe(1)-C(11)	94.59(2)
N(2)-Fe(1)-Cl(1)	97.185(17)	N(2)-Fe(1)-C(7)	105.85(2)
N(2)-Fe(1)-C(8)	102.81(2)	N(2)-Fe(1)-C(9)	130.86(2)
N(2)-Fe(1)-C(10)	161.12(2)	N(2)-Fe(1)-C(11)	135.49(2)
C(7)-Fe(1)-C(10)	57.54(2)	C(7)-Fe(1)-C(11)	34.17(2)
C(8)-Fe(1)-Cl(1)	121.479(18)	C(8)-Fe(1)-C(7)	35.40(2)
C(8)-Fe(1)-C(9)	35.85(2)	C(8)-Fe(1)-C(10)	58.61(2)
C(8)-Fe(1)-C(11)	57.78(2)	C(9)-Fe(1)-Cl(1)	92.116(18)
C(9)-Fe(1)-C(7)	58.64(2)	C(9)-Fe(1)-C(10)	35.04(2)
C(9)-Fe(1)-C(11)	57.46(2)	C(10)-Fe(1)-C(11)	33.94(2)
C(1)-N(1)-Fe(1)	102.69(4)	C(3)-N(1)-Fe(1)	111.20(5)
C(3)-N(1)-C(1)	108.53(6)	C(3)-N(1)-C(4)	108.70(7)
C(4)-N(1)-Fe(1)	115.24(5)	C(4)-N(1)-C(1)	110.21(7)
C(2)-N(2)-Fe(1)	108.04(5)	C(5)-N(2)-Fe(1)	114.15(5)
C(5)-N(2)-C(2)	108.95(6)	C(5)-N(2)-C(6)	107.55(6)
C(6)-N(2)-Fe(1)	108.57(4)	C(6)-N(2)-C(2)	109.53(6)
N(1)-C(1)-C(2)	110.72(6)	N(2)-C(2)-C(1)	111.21(6)
C(8)-C(7)-Fe(1)	69.11(4)	C(8)-C(7)-C(12)	126.29(6)
C(11)-C(7)-Fe(1)	75.46(4)	C(11)-C(7)-C(8)	107.64(6)
C(11)-C(7)-C(12)	125.46(6)	C(12)-C(7)-Fe(1)	127.96(5)
C(7)-C(8)-Fe(1)	75.49(4)	C(7)-C(8)-C(13)	127.45(6)
C(9)-C(8)-Fe(1)	72.46(4)	C(9)-C(8)-C(7)	107.81(5)
C(9)-C(8)-C(13)	123.80(6)	C(13)-C(8)-Fe(1)	126.42(5)
C(8)-C(9)-Fe(1)	71.69(4)	C(8)-C(9)-C(14)	125.43(6)
C(10)-C(9)-Fe(1)	75.70(4)	C(10)-C(9)-C(8)	108.24(6)

C(10)-C(9)-C(14)	126.20(6)	C(14)-C(9)-Fe(1)	121.69(5)
C(9)-C(10)-Fe(1)	69.25(4)	C(9)-C(10)-C(15)	125.60(6)
C(11)-C(10)-Fe(1)	75.19(4)	C(11)-C(10)-C(9)	107.94(6)
C(11)-C(10)-C(15)	126.45(6)	C(15)-C(10)-Fe(1)	121.36(5)
C(7)-C(11)-Fe(1)	70.37(4)	C(7)-C(11)-C(16)	125.89(6)
C(10)-C(11)-Fe(1)	70.87(4)	C(10)-C(11)-C(7)	108.35(6)
C(10)-C(11)-C(16)	125.20(6)	C(16)-C(11)-Fe(1)	131.16(5)

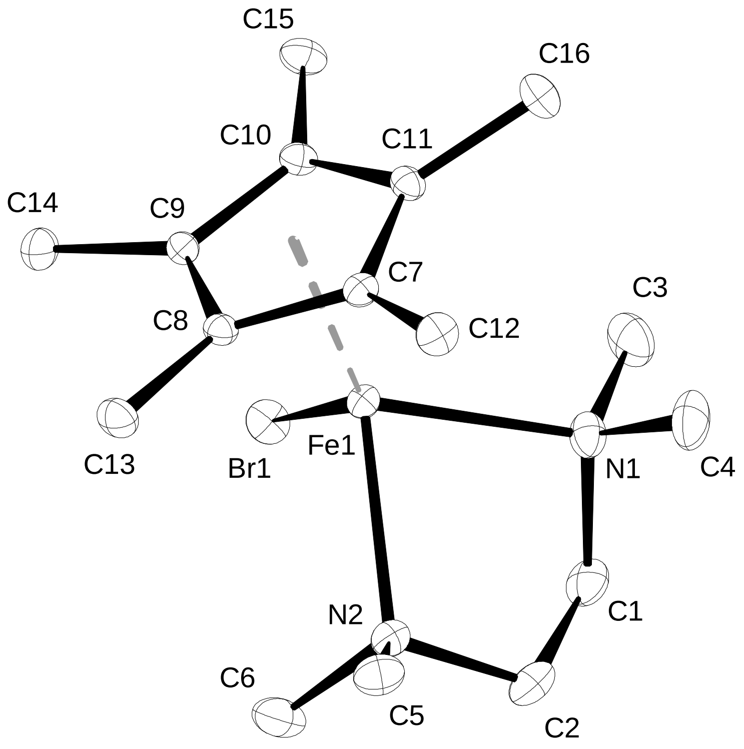

Figure S3. The asymmetric unit of **Fe-9b**. H atoms have been removed for clarity.

X-ray Crystal Structure Analysis of Fe-9b: $C_{16}H_{31}BrFeN_2$, M_r = 387.19 g mol^{-1}, yellow needle, crystal size 0.221 x 0.131 x 0.057 mm^3, monoclinic, space group $P2_1n$ [14], a = 10.3475(5) Å, b = 14.3632 (8) Å, c = 12.1802(7) Å, β = 90.457(2)°, V = 1810.20(17) Å^3,T = 100(2) K, Z = 4, D_{calc} = 1.421 g·cm^3, λ = 0.71073 Å, $\mu(Mo\text{-}K_\alpha)$ = 3.034 mm^{-1}, Gaussian correction (T_{min} = 0.57, T_{max} = 0.88), Bruker-AXS Kappa Mach3 with APEX-II detector and IμS microfocus Mo-anode X-ray source, 2.192 < θ< 30.998°. 59892 measured reflections, 5765 independent reflections, 4914 reflections with I > 2σ(I), R_{int} = 0.0422. The structure was solved by *SHELXT* and refined by full-matrix least-squares (*SHELXL*) against F^2 to R_1 = 0.0231 [I > 2σ(I)], wR_2 = 0.0506 [all data] with 190 parameters, 0 restraints.

Complete .cif-data of the compound are available under **CCDC-2469933**.

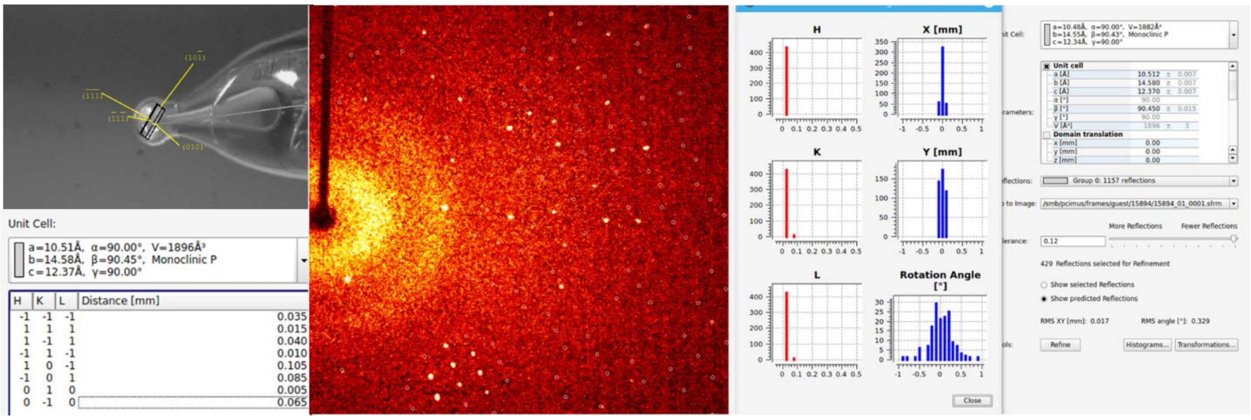

Figure S4. Crystal faces and unit cell determination/refinement of **Fe-9b**

INTENSITY STATISTICS FOR DATASET

Resolution	#Data	#Theory	%Complete	Redundancy	Mean I	Mean I/s	Rmerge	Rsigma
Inf - 2.91	94	94	100.0	16.01	77.30	90.27	0.0202	0.0066
2.91 - 1.89	221	221	100.0	17.55	66.50	99.69	0.0203	0.0072
1.89 - 1.48	316	316	100.0	17.98	44.23	82.77	0.0240	0.0082
1.48 - 1.29	318	318	100.0	17.92	26.51	67.97	0.0332	0.0104
1.29 - 1.17	310	310	100.0	17.53	24.44	59.47	0.0369	0.0115
1.17 - 1.08	339	339	100.0	15.99	24.02	54.36	0.0416	0.0131
1.08 - 1.02	291	291	100.0	12.17	18.29	37.91	0.0496	0.0184
1.02 - 0.97	306	306	100.0	10.50	18.35	34.09	0.0532	0.0214
0.97 - 0.92	375	375	100.0	9.14	11.83	23.64	0.0670	0.0303
0.92 - 0.89	249	249	100.0	8.35	11.12	21.31	0.0756	0.0347
0.89 - 0.85	410	410	100.0	7.66	10.28	18.70	0.0831	0.0404
0.85 - 0.83	241	241	100.0	7.48	8.52	15.72	0.1000	0.0484
0.83 - 0.80	379	379	100.0	7.16	9.33	15.68	0.0911	0.0474
0.80 - 0.78	326	326	100.0	7.05	9.29	15.85	0.0930	0.0510
0.78 - 0.76	320	320	100.0	6.80	6.63	11.10	0.1255	0.0683
0.76 - 0.74	371	371	100.0	6.52	6.98	11.40	0.1236	0.0707
0.74 - 0.73	204	204	100.0	6.36	6.94	10.92	0.1231	0.0727
0.73 - 0.71	436	436	100.0	6.29	6.37	9.87	0.1408	0.0828
0.71 - 0.70	228	228	100.0	5.99	5.88	8.95	0.1515	0.0924
0.70 - 0.69	248	248	100.0	5.93	4.53	7.28	0.1865	0.1148
0.69 - 0.68	291	341	85.3	3.24	4.41	5.10	0.1934	0.1724
0.78 - 0.68	2098	2148	97.7	5.86	6.03	9.36	0.1404	0.0897
Inf - 0.68	6273	6323	99.2	9.87	16.53	30.76	0.0421	0.0263

Table S3. Crystal data and structure refinement of **Fe-9b**

Identification code	15894	
Empirical formula	$C_{16} H_{31} Br Fe N_2$	
Color	yellow	
Formula weight	387.19 g · mol^{-1}	
Temperature	100(2) K	
Wavelength	0.71073 Å	
Crystal system	MONOCLINIC	
Space group	**$P2_1/n$, (no. 14)**	
Unit cell dimensions	a = 10.3475(5) Å	α= 90°.
	b = 14.3632(8) Å	β= 90.457(2)°.
	c = 12.1802(7) Å	γ = 90°.
Volume	1810.20(17) Å3	
Z	4	
Density (calculated)	1.421 Mg · m^{-3}	
Absorption coefficient	3.034 mm^{-1}	
F(000)	808 e	
Crystal size	0.221 x 0.131 x 0.057 mm^3	
θ range for data collection	2.192 to 30.998°.	
Index ranges	-14 ≤ h ≤ 14, -20≤ k ≤ 20, -17 ≤ l ≤ 17	
Reflections collected	59892	
Independent reflections	5765 [R_{int} = 0.0422]	
Reflections with I>2σ(I)	4914	
Completeness to θ = 25.242°	100.0 %	
Absorption correction	Gaussian	
Max. and min. transmission	0.88 and 0.57	
Refinement method	Full-matrix least-squares on F^2	
Data / restraints / parameters	5765 / 0 / 190	
Goodness-of-fit on F^2	1.030	
Final R indices [I>2σ(I)]	R_1 = 0.0231	wR^2 = 0.0481
R indices (all data)	R_1 = 0.0325	wR^2 = 0.0506
Largest diff. peak and hole	0.5 and -0.4 e · Å^{-3}	

Table S4. Bond lengths [Å] and angles [°] of **Fe-9b**

Br(1)-Fe(1)	2.5265(2)	Fe(1)-N(1)	2.2310(11)
Fe(1)-N(2)	2.2940(11)	Fe(1)-C(7)	2.3800(13)
Fe(1)-C(8)	2.3147(12)	Fe(1)-C(9)	2.3259(12)
Fe(1)-C(10)	2.3946(13)	Fe(1)-C(11)	2.4372(13)
N(1)-C(1)	1.4849(18)	N(1)-C(3)	1.4781(19)
N(1)-C(4)	1.4802(19)	N(2)-C(2)	1.4895(17)
N(2)-C(5)	1.4771(18)	N(2)-C(6)	1.4827(18)
C(1)-C(2)	1.513(2)	C(7)-C(8)	1.4275(17)
C(7)-C(11)	1.4278(18)	C(7)-C(12)	1.5043(18)
C(8)-C(9)	1.4218(18)	C(8)-C(13)	1.5028(18)
C(9)-C(10)	1.4225(17)	C(9)-C(14)	1.5025(18)
C(10)-C(11)	1.4213(18)	C(10)-C(15)	1.5008(18)
C(11)-C(16)	1.5050(18)		
N(1)-Fe(1)-Br(1)	94.34(3)	N(1)-Fe(1)-N(2)	78.86(4)
N(1)-Fe(1)-C(7)	108.55(4)	N(1)-Fe(1)-C(8)	143.48(4)
N(1)-Fe(1)-C(9)	149.26(4)	N(1)-Fe(1)-C(10)	114.29(4)
N(1)-Fe(1)-C(11)	95.36(4)	N(2)-Fe(1)-Br(1)	98.04(3)
N(2)-Fe(1)-C(7)	105.57(4)	N(2)-Fe(1)-C(8)	102.28(4)
N(2)-Fe(1)-C(9)	130.03(4)	N(2)-Fe(1)-C(10)	160.71(4)
N(2)-Fe(1)-C(11)	135.63(4)	C(7)-Fe(1)-Br(1)	149.65(3)
C(7)-Fe(1)-C(10)	57.81(4)	C(7)-Fe(1)-C(11)	34.46(4)
C(8)-Fe(1)-Br(1)	121.16(3)	C(8)-Fe(1)-C(7)	35.37(4)
C(8)-Fe(1)-C(9)	35.68(4)	C(8)-Fe(1)-C(10)	58.63(4)
C(8)-Fe(1)-C(11)	57.99(4)	C(9)-Fe(1)-Br(1)	91.61(3)
C(9)-Fe(1)-C(7)	58.59(4)	C(9)-Fe(1)-C(10)	35.04(4)
C(9)-Fe(1)-C(11)	57.64(4)	C(10)-Fe(1)-Br(1)	95.01(3)
C(10)-Fe(1)-C(11)	34.20(4)	C(11)-Fe(1)-Br(1)	126.32(3)
C(1)-N(1)-Fe(1)	102.97(8)	C(3)-N(1)-Fe(1)	112.22(9)
C(3)-N(1)-C(1)	108.67(11)	C(3)-N(1)-C(4)	108.46(12)
C(4)-N(1)-Fe(1)	114.05(9)	C(4)-N(1)-C(1)	110.28(11)
C(2)-N(2)-Fe(1)	108.43(8)	C(5)-N(2)-Fe(1)	113.60(8)
C(5)-N(2)-C(2)	108.93(11)	C(5)-N(2)-C(6)	107.61(11)
C(6)-N(2)-Fe(1)	108.72(8)	C(6)-N(2)-C(2)	109.51(11)
N(1)-C(1)-C(2)	110.75(11)	N(2)-C(2)-C(1)	111.36(11)
C(8)-C(7)-Fe(1)	69.82(7)	C(8)-C(7)-C(11)	107.72(11)
C(8)-C(7)-C(12)	126.48(12)	C(11)-C(7)-Fe(1)	74.96(7)
C(11)-C(7)-C(12)	125.16(12)	C(12)-C(7)-Fe(1)	127.93(9)
C(7)-C(8)-Fe(1)	74.81(7)	C(7)-C(8)-C(13)	127.60(12)
C(9)-C(8)-Fe(1)	72.59(7)	C(9)-C(8)-C(7)	107.85(11)
C(9)-C(8)-C(13)	123.59(11)	C(13)-C(8)-Fe(1)	127.10(9)

C(8)-C(9)-Fe(1)	71.73(7)	C(8)-C(9)-C(10)	108.38(11)
C(8)-C(9)-C(14)	125.10(11)	C(10)-C(9)-Fe(1)	75.12(7)
C(10)-C(9)-C(14)	126.34(12)	C(14)-C(9)-Fe(1)	122.86(9)
C(9)-C(10)-Fe(1)	69.84(7)	C(9)-C(10)-C(15)	125.85(12)
C(11)-C(10)-Fe(1)	74.54(7)	C(11)-C(10)-C(9)	107.82(11)
C(11)-C(10)-C(15)	126.32(12)	C(15)-C(10)-Fe(1)	121.51(9)
C(7)-C(11)-Fe(1)	70.58(7)	C(7)-C(11)-C(16)	125.86(12)
C(10)-C(11)-Fe(1)	71.26(7)	C(10)-C(11)-C(7)	108.21(11)
C(10)-C(11)-C(16)	125.32(12)	C(16)-C(11)-Fe(1)	130.88(9)

General Considerations

All reactions were carried out under Ar in flame-dried glassware, unless stated otherwise. The solvents were purified by distillation over the drying agents indicated and were transferred under Ar: THF, Et_2O (Mg/anthracene); CH_2Cl_2, MeCN, pyridine (CaH_2); toluene, benzene (Na/K); MeOH (Mg). Flash chromatography: Merck silica gel 60 (40–63 µm). NMR: Spectra were recorded on a Bruker AV 400 spectrometer, AV VIII 300, or AV 600Neo spectrometer in the solvents indicated; chemical shifts (δ) are given in ppm relative to TMS, coupling constants (*J*) in Hz. The solvent signals were used as references and the chemical shifts converted to the TMS scale ($CDCl_3$: $\delta_C \equiv$ 77.16 ppm; residual $CHCl_3$ in $CDCl_3$: $\delta_H \equiv$ 7.26 ppm).

Unless stated otherwise, all commercially available compounds were used as received (ABCR, Acros, Aldrich, Strem, Apollo Scientific, TCI, BLD pharm). Commercial Bu_3SnH is stabilized with 3,5-di-*tert*-butyl-4-hydroxytoluene (0.05% *w/w*), which was not removed in the reactions described herein. Bu_3SnD was prepared following a literature procedure.[1]

Iron Complexes

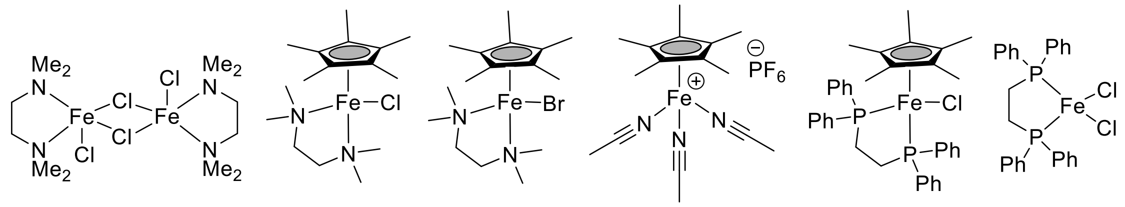

Fe-8a **Fe-9a** **Fe-9b** **Fe-10** **Fe-11** **Fe-13**

The iron complexes were prepared following the cited literature: **Fe-8a**,[2] **Fe-9a**,[3] **Fe-9b**,[4] **Fe-10**,[5] **Fe-11**,[6] **Fe-13**.[6]

[Cp*FeCl(tmeda)] (Fe-9a) (Slightly Modified Procedure).[3] A 100 mL jacketed cooling flask was charged with $FeCl_2\cdot thf$ (2.52 g, 11.4 mmol), THF (31 mL) and TMEDA (13 mL). The resulting slightly greenish solution was cooled to –30°C before Cp*Li (1.61 g, 11.4 mmol) was added in portions, causing a color change from green to yellow. The reaction mixture was stirred at –30 °C for three days before it was brought to ambient temperature and concentrated to a volume of approx. 30 mL. This mixture was then stirred at 65 °C (bath temperature) until the precipitate was dissolved. The resulting solution was cooled to –30°C overnight, causing the precipitation of the product, which was washed with diethyl ether at –30°C and dried under vacuum to furnish the title complex in form of yellow/greenish crystals (3.0 g, 78% yield).

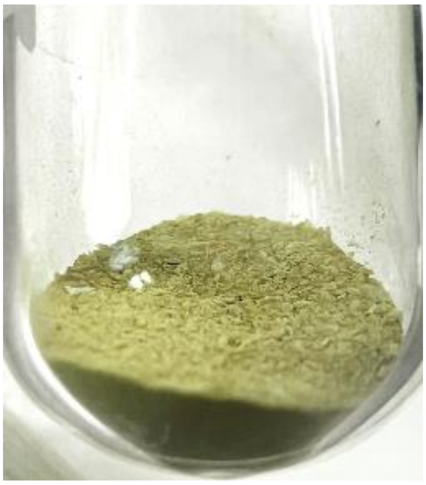

The complex **Fe-9a** can be stored at room temperature under inert atmosphere for several months without decomposition.

Substrates

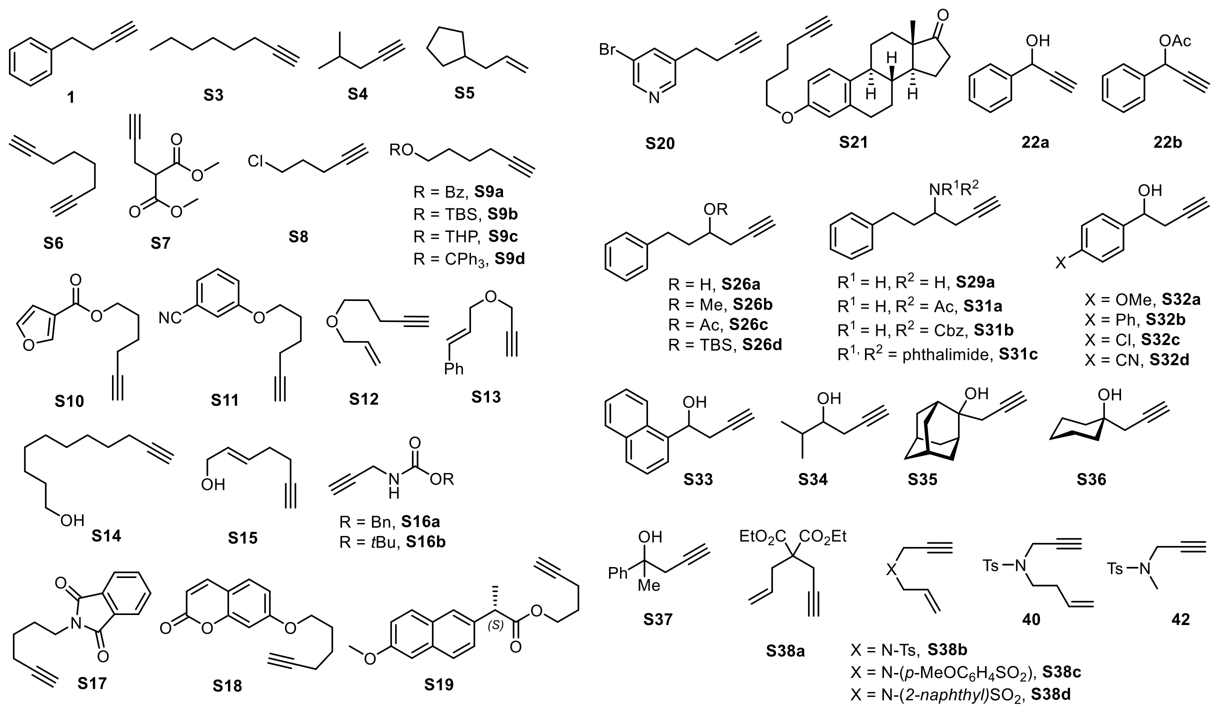

The alkynes **1**, **S3**, **S4**, **S5**, **S6**, **S7**, **S8**, **S16a**, **S16b** are commercially available and directly used as received.

The substrates **S9c**,[7] **S9b**,[8] **S12**,[9] **S13**,[10] **S14**,[11] **S15**,[12] **S20**,[13] **S34**,[14] **S35**,[15] **S36**,[16] **S37**,[17] **40**,[18] **42**,[19] **44**,[20] were prepared following the cited literature.

Hex-5-yn-1-yl benzoate (**S9a**). DMAP (800 mg, 6.4 mmol), DCC (1.67 g, 8.2 mmol) and benzoic acid (1.00 g, 8.2 mmol) were added to a solution of 5-hexyn-1-ol (630 mg, 6.4 mmol) in CH_2Cl_2 (18 mL) at 0°C. The solution was stirred at room temperature for 5 h, during which time a white curdy mixture was formed. The reaction was quenched by H_2O and the aqueous layer was extracted by CH_2Cl_2. The combined organic phases were washed with brine, dried over Na_2SO_4, and concentrated. The residue was purified by flash chromatography (10% EtOAc in hexane) to give the title compound as a transparent liquid (1.00 g, 84%). ^1H NMR (400 MHz, $CDCl_3$) δ 8.06 – 7.03 (m, 2H), 7.55 (t, J = 7.4 Hz, 1H), 7.42 (t, J = 7.6 Hz, 2H), 4.34 (t, J = 6.4 Hz, 2H), 2.27 (td, J = 7.0, 2.6 Hz, 2H), 1.96 (t, J = 2.6 Hz, 1H), 1.94 – 1.82 (m, 2H), 1.76 – 1.62 (m, 2H). ^{13}C NMR (101 MHz, $CDCl_3$) δ 166.7, 133.1, 130.5, 129.7, 128.5, 84.0, 68.9, 64.6, 27.9, 24.8, 18.3. The spectroscopic data was in agreement with the literature.[21]

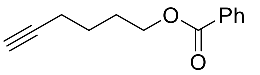

(Hex-5-yn-1-yloxy)methanetriyl)tribenzene (**S9d**). 5-Hexyn-1-ol (196 mg, 2 mmol) was added at room temperature to a solution of trityl chloride (669 mg, 2.4 mmol) and 4-(dimethylamino)pyridine (367 mg, 3 mmol) in THF (5 mL). The mixture was stirred at reflux temperature for 24 h and then allowed to cool before the reaction was quenched with water (5 mL). The aqueous layer was extracted with ethyl acetate (3 × 6 mL), the combined organic phases were washed with brine, dried over Na_2SO_4, and concentrated under reduced pressure. The residue was purified by flash chromatography (EtOAc/hexane, 1:50) to give the title compound as a colorless oil (515 mg, 72%). ^1H NMR (400 MHz, $CDCl_3$) δ 7.49-7.47 (m, 6H), 7.35-7.31 (m, 6H), 7.29-7.24 (m, 3H), 3.12 (t, J = 6.2 Hz, 2H), 2.21 (td, J = 6.9 Hz, 2H), 1.96 (t, J = 2.6 Hz, 1H), 1.80-1.66 (m, 4H), 1.47. ^{13}C NMR (101 MHz, $CDCl_3$) δ

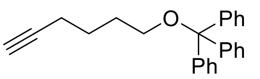

144.5, 128.8, 127.9, 127.0, 86.5, 84.6, 68.4, 63.0, 29.3, 27.1, 25.6, 18.4. The spectroscopic data was in agreement with the literature.[8]

Hex-5-yn-1-yl furan-3-carboxylate (**S10**). 5-Hexyn-1-ol (0.94 mL, 10.0 mmol, 1.0 equiv) was added dropwise at room temperature to the solution of furan-3-carboxylic acid (1.12 g, 10 mmol), N-(3-dimethylaminopropyl)-N'-ethylcarbodiimide hydrochloride (2.30 g, 12 mmol) and DMAP (122 mg, 1 mmol) in CH_2Cl_2 (20 mL). The mixture was stirred at room temperature for 24 h before the reaction was quenched with water. The aqueous phase was extracted with ethyl acetate. The combined organic layers were washed with brine, dried over Na_2SO_4, and concentrated under reduced pressure. The residue was purified by flash chromatography (hexanes/ethyl acetate 4/1) to give the title compound as a colorless liquid (1.30 g, 70%). ^{1}H NMR (400 MHz, $CDCl_3$) δ 8.01 (s, 1H), 7.41 (td, *J* = 1.8 Hz, 1H), 6.74 (td, *J* = 1.7 Hz, 1H), 4.27 (td, *J* = 6.5 Hz, 2H), 2.26 (tdd, *J* = 7.0 Hz, 2H), 1.97 (t, *J* = 2.6 Hz, 1H), 1.89-1.82 (m, 2Hz), 1.69-1.62 (m, 2H). ^{13}C NMR (101 MHz, $CDCl_3$) δ 163.1, 147.7, 143.7, 119.5, 109.8, 83.8, 77.4, 77.2, 77.0, 76.7, 68.8, 63.9, 27.7, 25.0, 18.1. The spectroscopic data was in agreement with the literature.[22]

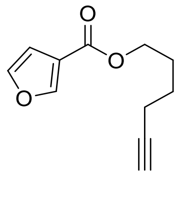

3-(Hex-5-yn-1-yloxy)benzonitrile (**S11**). A solution of 3-cyanophenol (595 mg, 5 mmol), 6-chlorohex-1-yne (641 mg, 5.5 mmol), KI (83 mg, 0.5 mmol) and Cs_2CO_3 (2.5 g, 7.5 mmol) in acetonitrile (20 mL) was stirred for 18 h at 85°C (bath temperature). The mixture was poured into water (100 mL) and the aqueous layer extracted with EtOAc (3 × 100 mL). The combined organic phases were washed with brine (100 mL), dried over Na_2SO_4, filtered and evaporated. The crude product was purified by flash chromatography (hexane/ethyl acetate, 5/1) to give the title compound as a transparent liquid (640 mg, 65%). ^{1}H NMR (400 MHz, $CDCl_3$) δ 7.31 (t, *J* = 7.8 Hz, 1H), 7.23 (dq, *J* = 7.6 Hz, 1H), 7.24-7.11 (m, 2H), 4.00 (t, *J* = 6.3 Hz, 2H), 2.33 (tdd, *J* = 7.0 Hz, 3H), 1.98-1.90(m, 2H), 1.76-1.68 (m, 2H). ^{13}C NMR (101 MHz, $CDCl_3$) δ 159.2, 130.4, 124.5, 119.9, 118.9, 117.5, 113.3, 83.9, 69.0, 67.8, 28.1, 27.0, 25.0, 18.2. The spectroscopic data was in agreement with the literature.[23]

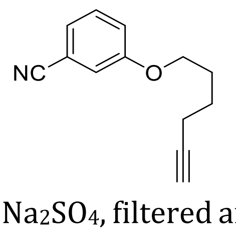

2-(Hex-5-yn-1-yl)isoindoline-1,3-dione (**S17**). A round-bottom flask was charged with 6-chlorohex-1-yne (1.05 g 4.5 mmol) and DMF (20 mL). Potassium phthalimide (2.00 g, 5.4 mmol) was added and the resulting yellowish suspension was stirred at 70 °C (bath temperature) for 24 h. The solution was cooled to room temperature before water (70 mL) was added. The mixture was extracted with Et_2O (4 × 70 mL), and the combined organic layers were dried over anhydrous $MgSO_4$, filtered, and concentrated. The residue was purified by flash chromatography (hexanes/EtOAc, 5/1) to give the title compound as a white solid (660 mg, 65%). ^{1}H NMR (400 MHz, $CDCl_3$) δ 7.84 (dd, *J* = 5.4 Hz, 2H), 7.71 (dd, *J* = 5.4 Hz, 2H), 3.71 (t, *J* = 7 Hz, 2H), 2.24 (td, *J* = 7.0 Hz, 2H), 1.94 (t, *J* = 2.2 Hz, 1H), 1.85-1.75 (m, 2H), 1.62-1.55 (m, 2H). ^{13}C NMR (101 MHz, $CDCl_3$) δ 168.5, 134.0, 132.3, 123.3, 83.9, 77.5, 77.4, 77.2, 76.8, 68.9, 37.6, 27.8, 25.8, 18.1. The spectroscopic data was in agreement with the literature.[21]

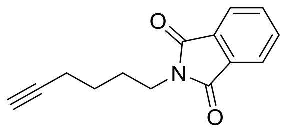

7-(Hex-5-yn-1-yloxy)-2*H*-chromen-2-one (**S18**). 6-Chloro-1-hexyne (641 mg, 5.5 mmol) was added to the suspension of 7-hydroxycumarin (810 mg mg, 5 mmol), cesium carbonate (2.5 g, 7.5 mmol) and potassium iodide (83 mg, 0.5 mmol) in acetonitrile (20 mL) at room temperature. The mixture was stirred at 85 °C (bath temperature) overnight. After cooling, the reaction was quenched with water and the aqueous phase extracted with ethyl acetate. The combined organic layers were washed with brine, dried over Na_2SO_4, and concentrated under reduced pressure. The residue was purified by flash chromatography

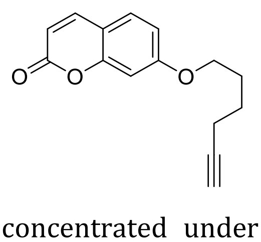

(hexanes/EtOAc, 4/1) to give the title compound as a white solid (907 mg, 75%). ^{1}H NMR (400 MHz, $CDCl_3$) δ 7.63-7.61 (m, 1H), 7.35 (d, *J* = 8.6 Hz, 1H), 6.84-6.78 (m, 2H), 6.23 (dd, *J* = 9.4 Hz, 1H), 4.05-4.02 (m, 2H), 2.29-2.24 (m, 2H), 1.98-1.92 (m, 3H), 1.75-1.68 (m, 2H). ^{13}C NMR (101 MHz, $CDCl_3$) δ 162.4, 161.4, 156.0, 143.6, 128.8, 113.0, 112.6, 83.9, 69.0, 68.0, 44.6, 31.5, 28.1, 25.7, 25.0, 18.2, 17.8. The spectroscopic data was in agreement with the literature.[22]

Pent-4-yn-1-yl (*S*)-2-(6-methoxynaphthalen-2-yl)propanoate (**S19**). A solution of pent-4-yn-1-ol (336 mg, 4.0 mmol) in CH_2Cl_2 (4 mL) was slowly added via cannula to a solution of DCC (990 mg, 4.8 mmol), DMAP (48 mg, 0.4 mmol, 0.1 equiv.) and (*S*)-Naproxen (967 mg, 4.2 mmol) in CH_2Cl_2 (50 mL) at ambient temperature. The mixture was stirred overnight before it was filtered, and the filtrate was concentrated. The residue was purified by flash chromatography (hexane/EtOAc, 10/1) to give the title compound as a white solid (828 mg, 70%). ^{1}H NMR (400 MHz, $CDCl_3$) δ 7.72 (s, 1H), 7.70 (s, 1H), 7.66 (d, *J* = 1.7 Hz, 1H), 7.44 – 7.36 (m, 1H), 7.17 – 7.09 (m, 2H), 4.18 (t, *J* = 6.3 Hz, 2H), 3.92 (s, 3H), 3.86 (q, *J* = 7.2 Hz, 1H), 2.17 (td, *J* = 7.3, 2.7 Hz, 2H), 1.93 (t, *J* = 2.7 Hz, 1H), 1.85 – 1.74 (m, 2H), 1.58 (d, *J* = 7.3 Hz, 3H). ^{13}C NMR (101 MHz, $CDCl_3$) δ 174.7, 157.8, 135.8, 133.8, 129.4, 129.0, 127.3, 126.3, 126.0, 119.1, 105.7, 83.1, 69.1, 63.4, 55.4, 45.6, 27.6, 18.6, 15.2. The spectroscopic data was in agreement with the literature.[24]

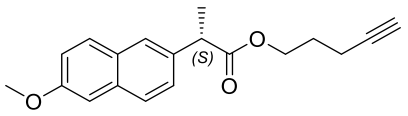

Compound S21. A mixture of estrone (1.4 g, 5.0 mmol), 5-chloro-pent-1-yne (641 mg, 5.5 mmol), KI (83 mg, 0.5 mmol) and Cs_2CO_3 (2.5 g, 7.5 mmol) in MeCN (20 mL) was stirred for 18 h at 85°C (bath temperature). After cooling to ambient temperature, the mixture was poured into water (100 mL) and the aqueous phase was extracted with EtOAc (3 × 100 mL). The combined organic layers were washed with brine (100 mL), dried over Na_2SO_4, filtered and evaporated. The residue was purified by flash chromatography (petroleum ether/EtOAc, 5/1) to afford the title compound as a white solid (1.32 g, 78%). ^{1}H NMR (400 MHz, $CDCl_3$) δ 7.19 (dd, *J* = 8.6 Hz, 1H), 6.71 (dd, *J* = 8.6 Hz, 1H), 6.69-6.63 (m, 1H), 3.96(t, *J* = 6.2 Hz, 2H), 2.91-2.87 (m, 2H), 2.54-2.47 (m, 1H), 2.44-2.37 (m, 1H), 2.29-2.25 (m, 3H), 2.17-1.98 (m, 4H), 1.93-1.88 (m, 2H), 1.74-1.71 (m, 2H), 1.62-1.43 (m, 4H), 0.91 (s, 3H). ^{13}C NMR (101 MHz, $CDCl_3$) δ 221.1, 157.2, 137.9, 132.1, 126.5, 114.7, 112.2, 84.3, 68.7, 67.3, 50.6, 48.2, 44.1, 38.5, 36.0, 31.7, 29.8, 28.5, 26.7, 26.1, 25.2, 21.7, 18.3, 14.0. The spectroscopic data was in agreement with the literature.[22]

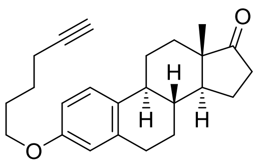

1-Phenylprop-2-yn-1-yl acetate (**22b**). 1-Phenyl-prop-2-yn-1-ol (**22a**, 925 mg, 7 mmol) was added to a solution of acetic anhydride (765 mg, 7.5 mmol), triethylamine (760 mg, 7.5 mmol), and N,N-dimethyl-4-aminopyridine (68 mg, 0.56 mmol, 0.1 eq.) in dichloromethane (30 mL). The mixture was stirred at room temperature for 13 h and the reaction was quenched with aq. HCl (2 M, 60 mL). The aqueous phase was extracted with diethyl ether (3 x 20 mL), the combined organic layers were washed with sat. aq. $NaHCO_3$ (2 x 80 mL) and dried ($MgSO_4$). The solvent was evaporated and the residue was purified by flash chromatography (hexane/ethyl acetate, 10/1) to give the corresponding product as a light yellow liquid (652 mg, 75%). ^{1}H NMR (400 MHz, $CDCl_3$) δ 7.55-7.52 (m, 2H), 7.42-7.37 (m, 3H), 6.46 (d, *J* = 2.3 Hz, 1H), 2.66 (d, *J* = 2.3 Hz, 1H), 2.12 (s, 3H). ^{13}C NMR (101 MHz, $CDCl_3$) δ 169.8, 136.6, 129.2, 129.1, 128.8, 128.6, 128.4, 128.1, 127.8, 80.4, 77.5, 77.1, 76.8, 75.5, 65.4, 21.2. The spectroscopic data was in agreement with the literature.[25]

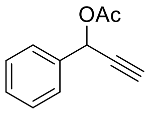

1-Phenylhex-5-yn-3-ol (**26a**). **Representative Method A**. A 100 mL three-neck flask

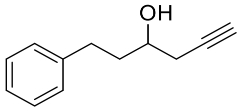

was charged a stir bar, magnesium turnings (1.10 g, 45 mmol) and $HgCl_2$ (0.12 g, 0.45 mmol, 1.5 mol %). The reaction flask was then equipped with a condenser and purged with Ar before Et_2O (40 mL) was added. A solution of propargyl bromide (80% *w/w* in toluene, 5.1 mL, 45 mmol) in Et_2O (10 mL) was added dropwise to the suspension, causing gentle boiling of the solvent. Once the addition was complete, the resulting solution was stirred at reflux temperature for 1 h before it was cooled to −25°C. A solution of hydrocinnamaldehyde (3.9 mL, 30 mmol) in Et_2O (10 mL) was added slowly and the resulting mixture stirred at −25°C for another 3 h before it was warmed to room temperature. sat. NH_4Cl solution was carefully added, the aqueous phase was extracted with Et_2O for three times, the combined organic layers were dried over $MgSO_4$ and concentrated in vacuo. The residue was purified by flash chromatography (hexane/ethyl acetate, 4/1) to afford the title compound as a pale yellow oil (5.1 g, 98%). ^1H NMR (400 MHz, $CDCl_3$) ^1H NMR (400 MHz, $CDCl_3$) δ 7.31- 7.28 (m, 2H), 7.22-7.18 (m, 2H), 3.82-3.76 (m, 1H), 2.85-2.78 (m, 1H), 2.74-2.67 (m, 1H), 2.46 (ddd, *J* = 16.7 Hz, 1H), 2.36 (ddd, *J* = 16.7 Hz, 1H), 2.07 (t, *J* = 2.7 Hz, 1H), 1.96-1.90 (br, 1H), 1.90-1.85 (m, 2H). ^{13}C NMR (101 MHz, $CDCl_3$) δ 141.6, 128.8, 128.5, 128.4, 126.0, 80.7, 77.4, 77.0, 76.7, 71.0, 69.1, 37.8, 31.9, 27.5, 27.0. The spectroscopic data was in agreement with the literature.[26]

(3-Methoxyhex-5-yn-1-yl)benzene (**26b**). A solution of the homo-propargylic alcohol **S26a**

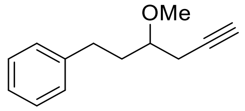

(764 mg, 4.3 mmol) in THF (5 mL) was added dropwise at ambient temperature to a suspension of NaH (126 mg, 5.2 mmol) in THF (10 mL). Stirring was continued for 30 min before methyl iodide (1.28 g, 8.83 mmol) was added. The mixture was stirred at room temperature for 2 h to give a light yellow turbid solution. Water (10 mL) was added and the aqueous phase was extracted with *tert*-butyl methyl ether (3 x 15 mL). The organic layers were combined, dried over $MgSO_4$, filtered and concentrated. The yellow residue was purified by flash chromatography (hexanes/EtOAc, 9:1) to afford the title compound as a colorless liquid (330 mg, 90%). ^1H NMR (400 MHz, $CDCl_3$) δ 7.32-7.28 (m, 2H), 7.23-7.19 (m, 3H), 3.41 (s, 3H), 3.36-3.31 (m, 1H), 2.83-2.76 (m, 1H), 2.72-2.64 (m, 1H), 2.46-2.43 (m, 2H), 2.02-1.94 (m, 3H). ^{13}C NMR (101 MHz, $CDCl_3$) δ 142.1, 128.6, 125.9, 80.9, 78.4, 70.2, 57.1, 35.5, 31.5, 23.1.

1-Phenylhex-5-yn-3-yl acetate (**26c**). Alcohol **S26a** (1.2 g, 7 mmol) was added to a solution of

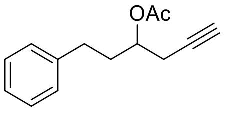

acetic anhydride (756 mg, 7.5 mmol), triethylamine (1.2 g, 7.5 mmol) and N,N-dimethyl-4-aminopyridine (68 mg, 0.56 mmol) in dichloromethane (30 mL). The mixture was stirred at room temperature for 13 h before HCl (2 M, 60 mL) was carefully added. The mixture was extracted with diethyl ether (20 mL x 3). The combined organic layers were washed with sat. aq. $NaHCO_3$ (80 mL x 2) and dried ($MgSO_4$), the solvent was evaporated, and the residue was purified by flash chromatography (hexane/EtOAc, 8:1) to afford the title compound as a colorless liquid (1.2 g, 85%). ^1H NMR (400 MHz, $CDCl_3$) δ 7.31-7.27 (m, 2H), 7.21-7.17 (m, 3H), 5.00-4.94 (m, 1H), 2.74-2.61 (m,2H), 2.52-2.50 (m, 2H), 2.08-2.07 (s, 3H), 2.04-2.00 (m, 3H). ^{13}C NMR (101 MHz, $CDCl_3$) δ 170.7, 141.3, 128.6, 128.5, 126.2, 79.6, 71.3, 70.7, 34.7, 31.7, 24.1, 21.2.

***tert*-Butyldimethyl((1-phenylhex-5-yn-3-yl)oxy)silane** (**26d**). Imidazole (544 mg, 8 mmol)

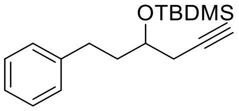

and *tert*-butyldimethylchlorosilane (904 mg, 6 mmol) were added to the solution of alcohol **S26a** (696 mg, 4 mmol) in DMF (10 mL). The mixture was stirred for 5 h before the reaction was quenched with aq. sat. NH_4Cl. The aqueous phase was extracted with CH_2Cl_2 (3 x 10 mL), the combined organic layers were dried over $MgSO_4$, filtered and concentrated. The residue was purified by flash

chromatography (cyclohexane/EtOAc, 5-10%) to give the title compound as a colorless liquid (1.20 g, 85%). ^{1}H NMR (400 MHz, $CDCl_3$) δ 7.31-7.27 (m, 2H), 7.21-7.18 (m, 3H), 3.91-3.85 (m, 1H), 2.79-2.71 (m, 1H), 2.66-2.58 (m, 1H), 2.40-2.36 (m, 2H), 2.00-1.94 (m, 2H), 1.91-1.81 (m, 1H), 0.92 (s, 9H), 0.09 (d, *J* = 1.3 Hz, 6H). ^{13}C NMR (101 MHz, $CDCl_3$) δ 142.5, 128.6, 125.9, 81.6, 70.6, 70.2, 38.5, 31.6, 27.4, 26.0, 18.3, –4.3, –4.5.

1-Phenylhex-5-yn-3-amine (**29a**). Prepared according to the a literature procedure as a

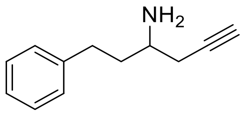

colorless liquid (562 mg, 65%).[27] ^{1}H NMR (400 MHz, $CDCl_3$) δ 7.31-7.27 (m, 2H), 7.21-7.18 (m, 3H), 2.97-2.91 (m, 1H), 2.79-2.62 (m, 2H), 2.38 (ddd, *J* = 16.6 Hz, 1H), 2.23 (ddd, *J* = 16.6 Hz, 1H), 2.03 (t, *J* = 2.6 Hz, 1H), 1.87-1.79 (m, 1H), 1.74-1.67 (m, 1H), 1.42 (br, 2H). ^{13}C NMR (101 MHz, $CDCl_3$) δ 142.0, 128.5, 126.2, 81.7, 70.5, 49.8, 38.7, 32.7, 28.1.

N-(1-Phenylhex-5-yn-3-yl)acetamide (**S31a**). The crude amine **29a** (34 mg, 0.2 mmol) was

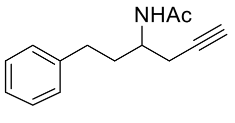

dissolved in pyridine (1.5 mL) and Ac_2O (40 mg, 0.4 mmol) was added. This mixture was stirred at ambient temperature for 12 h before aq. HCl (1 M, 10 mL) was carefully added. The aqueous layer was extracted with EtOAc (3 x 10 mL) and the combined organic extracts were dried ($MgSO_4$) and concentrated in vacuo. The residue was purified by flash chromatography (hexanes/EtOAc, 4:1) to give the title compound as a white solid (40 mg, 95%). ^{1}H NMR (400 MHz, $CDCl_3$) δ 7.30-7.27 (m, 2H), 7.21-7.17 (m, 3H), 5.59 (br, 1H), 4.17-4.09 (brm, 1H), 2.69- 2.64 (m, 2H), 2.55-2.48 (m, 1H), 2.43-2.37 (m, 1H), 2.02 (t, *J* = 2.7 Hz, 1H), 1.97 (s, 3H), 1.94-1.88 (m, 2H). ^{13}C NMR (101 MHz, $CDCl_3$) δ 169.7, 141.4, 128.6, 128.4, 126.0, 80.2, 71.1, 47.0, 35.2, 32.5, 27.0, 24.4, 23.5.

Benzyl (1-phenylhex-5-yn-3-yl)carbamate (**S31b**). Benzyl chloroformate (68 mg, 0.4 mmol)

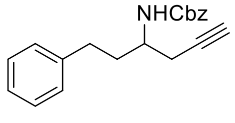

and triethylamine (30 mg, 0.3 mmol) were slowly added at 0°C to a solution of amine **29a** (34 mg, 0.2 mmol) in THF (5 mL). The mixture was stirred at room temperature for 3 h, the solvent was evaporated under reduced pressure, and the residue extracted with ether (3 × 20 mL). The organic phase was dried over anhydrous Na_2SO_4 and evaporated under the reduced pressure, and the residue was purified by flash chromatography (hexane/EtOAc = 9:1) to give the title compound as a white solid (52 mg, 85%). ^{1}H NMR (400 MHz, $CDCl_3$) δ 7.40-7.29 (m, 7H), 7.23-7.19 (m, 3H), 5.15 (br, 2H), 5.03 (br, 1H), 3.88 (brs, 1H), 2.70-2.57 (m, 2H), 2.52-2.46 (br, 1H), 2.45-2.42 (m, 1H), 2.03 (s, 1H), 1.96-1.90 (m, 2H). ^{13}C NMR (101 MHz, $CDCl_3$) δ 155.9, 141.4, 136.5, 128.6, 128.5, 128.4, 128.2, 128.2, 126.1, 80.0, 71.2, 66.7, 49.0, 35.5, 32.4, 24.7.

2-(1-Phenylhex-5-yn-3-yl)isoindoline-1,3-dione (**S31c**). DIAD (48 mg, 0.24 mmol) was added

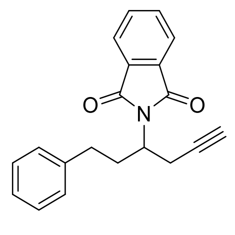

dropwise to the solution of alcohol **S26a** (34 mg, 0.2 mmol), phthalimide (35 mg, 0.24 mmol) and PPh_3 (1.2 equiv) in THF. The color of the solution turned yellow upon addition of DIAD. After stirring for 16 h, H_2O was added to the aqueous phase was extracted with hexane. The organic layer was washed with brine, dried with $MgSO_4$, filtered and evaporated. The residue was purified by flash chromatography (2-5% EtOAc in cyclohexane) to give the title compound as a sticky oil (48 mg, 80%). ^{1}H NMR (400 MHz, $CDCl_3$) δ 7.82-7.80 (m, 1H), 7.71-7.70 (m, 2H), 7.20-7.04 (m, 5H), 4.53-4.46 (m, 1H), 2.96 (ddd, *J* = 16.8 Hz, 1H), 2.69 (ddd, *J* = 16.7 Hz, 1H), 2.68-2.51 (m, 3H), 2.17- 2.10 (m, 1H), 1.86 (s, 1H) 1.43 (s, 1H). ^{13}C NMR (101 MHz, $CDCl_3$) δ 168.5, 140.8, 134.1, 131.9, 128.5, 128.4, 126.0, 123.3, 80.6, 70.4, 51.0, 33.2, 33.0, 27.0, 23.0.

1-(4-Methoxyphenyl)but-3-yn-1-ol (**S32a**). **Representative Method B**. A solution of 4-methoxybenzaldehyde (136 mg, 1.0 mmol) in THF (3.0 mL) and propargyl bromide (223 mg, 1.5 mmol, 80% *w/w* in toluene) was treated with activated zinc (71 mg, 1.1 mmol) at 0°C. After stirring for 2 h at 0°C, the reaction was quenched with sat. aq. NH_4Cl. After extraction of the aqueous layer with *tert*-butyl methyl ether (two times), the combined organic layer was washed with H_2O, dried over $MgSO_4$, filtered, and concentrated in vacuo. The residue was purified by flash chromatography (10% EtOAc in hexane) to afford the title compound as a pale yellow oil (158 mg, 97%). ^{1}H NMR (400 MHz, $CDCl_3$) δ 7.31 (d, *J* = 6.7 Hz, 2H), 6.74 (d, *J* = 8.7 Hz, 2H), 4.85-4.81 (br, 1H), 3.80 (s, 3H), 2.64-2.61 (m, 2H), 2.38-2.34 (m, 2H), 2.06 (dt, *J* = 2.6 Hz, 1H). ^{13}C NMR (101 MHz, $CDCl_3$) δ 159.5, 127.1, 114.0, 81.0, 72.1, 71.0, 55.4, 29.5. The spectroscopic data was in agreement with the literature.[16]

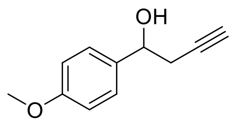

1-([1,1'-Biphenyl]-4-yl)but-3-yn-1-ol (**S32b**). Prepared by method B as a transparent liquid (217 mg, 98%). ^{1}H NMR (400 MHz, $CDCl_3$) δ 7.61-7.33 (m, 9H), 4.93 (t, 1H, *J* = 6.4 Hz), 2.70-2.68 (m, 2H), 2.37 (s, 1H), 2.10 (t, 1H, *J* = 2.8 Hz). ^{13}C NMR (101 MHz, $CDCl_3$) δ 141.6, 141.1, 140.9, 128.9, 127.5, 127.4, 127.2, 126.3, 80.8, 72.2, 71.2, 29.6. The spectroscopic data was in agreement with the literature.[16]

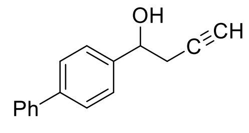

1-(4-Chlorophenyl)but-3-yn-1-ol (**S32c**). Prepared by method B as a yellowish oil (171 mg, 95%). ^{1}H NMR (400 MHz, $CDCl_3$) δ 7.33 (s, 4H), 4.86 (dt, 1H, *J* = 3.6, 6.4 Hz), 2.63-2.60 (m, 2H, 2.37 (d, 1H, *J* = 3.6 Hz), 2.07 (t, 1H, *J* = 2.8 Hz). ^{13}C NMR (101 MHz, $CDCl_3$) δ 128.8, 127.3, 80.3, 71.8, 71.5, 29.6. The spectroscopic data was in agreement with the literature.[16]

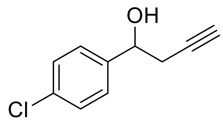

4-(1-Hydroxybut-3-yn-1-yl)benzonitrile (**S32d**). Prepared by method B as a yellowish liquid (162 mg, 95%). ^{1}H NMR (400 MHz, $CDCl_3$) δ 7.65 (d, *J* = 8.4 Hz, 2H), 7.52 (d, *J* = 8.0 Hz, 2H), 4.96-4.91 (m, 1H), 2.70- 2.55 (m, 3H), 2.10 (t, *J* = 2.6 Hz, 1H). ^{13}C NMR (101 MHz, $CDCl_3$) δ 147.6, 132.7, 132.4, 126.7, 118.8, 111.8, 79.6, 72.0, 71.6, 29.6. The spectroscopic data was in agreement with the literature.[16]

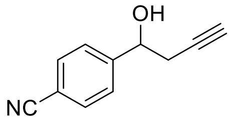

1-(Naphthalen-1-yl)but-3-yn-1-ol (**S33**). Prepared by method B as a colorless liquid (186 mg, 95%). ^{1}H NMR (400 MHz, $CDCl_3$) δ 8.07-7.25 (m, 7H), 5.66 (br s, 1H), 2.98-2.72 (m, 2H), 2.59 (s, 1H), 2.14 (t, 1H, *J* = 2.8 Hz). ^{13}C NMR (101 MHz, $CDCl_3$) δ 137.9, 133.9, 130.3, 129.2, 128.6, 126.4, 125.8, 125.5, 123.1, 122.9, 81.1, 71.4, 69.4, 28.8. The spectroscopic data was in agreement with the literature.[16]

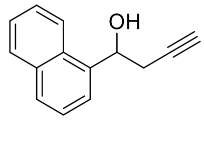

Deuterated Substrates

4-Deutero-(but-3-yn-1-yl)benzene (**[D]-1**). A 50 mL Schlenk flask was loaded with alkyne **1** (650 mg, 5 mmol) and THF (20 mL). The solution was cooled to −78 °C before *n*BuLi (1.6 M in hexane, 3.75 mL, 6 mmol) was added dropwise. The resulting mixture was stirred at that temperature for 1 h, then the cooling bath was removed and the mixture allowed to reach ambient temperature where stirring was continued for another 1 h. The reaction was quenched with D_2O (5 mL) causing a color change from brown to light yellow. The aqueous layer was extracted with *tert*-butyl methyl ether (3 x 10 mL), the combined organic layers were dried over $MgSO_4$, filtered and concentrated to give the title compound as a pale yellow liquid (641 mg, 98%). ^1H NMR (600 MHz, $CDCl_3$) δ: 7.34-7.31 (m, 2H), 7.26-7.24 (m, 3H), 2.88 (t, *J* = 7.6Hz, 2H), 2.51 (t, *J* = 7.6Hz, 2H). ^2H NMR (92 MHz, $CDCl_3$) δ: 2.00 (br s). ^{13}C NMR (151 MHz, $CDCl_3$) δ: 140.5, 128.6, 126.5, 83.5, 83.5, 83.4, 69.0, 68.8, 68.5, 34.8, 20.7.

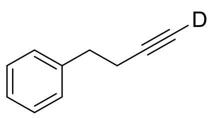

6-Deutero-1-phenylhex-5-yn-3-ol (**[D]-26**). Prepared analogously from **26a** as a light yellow liquid (831 mg, 95%). ^1H NMR (400 MHz, $CDCl_3$) δ ^1H NMR (400 MHz, $CDCl_3$) δ 7.31- 7.28 (m, 2H), 7.22-7.18 (m, 2H), 3.82-3.76 (m, 1H), 2.85-2.78 (m, 1H), 2.74-2.67 (m, 1H), 2.46 (ddd, *J* = 16.7 Hz, 1H), 2.36 (ddd, *J* = 16.7 Hz, 1H), 1.96-1.90 (br, 1H), 1.90-1.85 (m, 2H). ^2H NMR (92 MHz, $CDCl_3$) δ 2.06. ^{13}C NMR (101 MHz, $CDCl_3$) δ 141.8, 128.6, 126.1, 80.3, 70.9, 69.2, 68.1, 37.9, 32.0, 27.6, 27.1, 25.7.

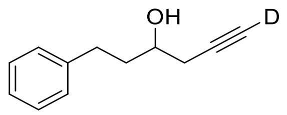

Mechanistic Studies

Radical Trapping Experiments

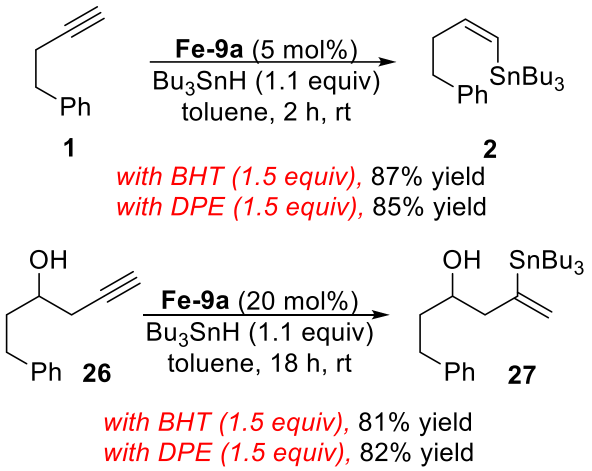

Scheme S1. Radical scavenging experiments; BHT = butylated hydroxytoluene; DPE = 1,1-diphenylethene

A 25 mL Schlenk flask was charged with complex **Fe-9a** (5 mol%, 3.5 mg for **1** & 20 mol%, 14 mg for **26** for 0.2 mmol scale of alkyne) and toluene (2 mL). A mixture of the respective substrate (1 eq.), $HSnBu_3$ (1.1 equiv.) and the additive (1.5 equiv., either BHT or 1,1-diphenylethylene) in toluene (2 mL) was added with a syringe pump at ambient temperature over 15 min. A color

change from greenish yellow to dark brown was observed and the mixture was stirred for the time indicated in Scheme S1, monitoring the reaction by TLC. The solvent was removed upon completion of the reaction, the residue was dissolved in hexane (or cyclohexane), the resulting suspension was filtered through a plug of Celite, the filtrate was evaporated, and the residue inspected by ^{1}H NMR to determine the product ratio (proximal/distal addition). The residue was then purified by flash chromatography (hexane/ethyl acetate + 1-2% Et_3N).

No significant change in the yield or isomer ratio was observed. Hence, a radical mechanism can likely be excluded.

Isotope Labelling Experiments

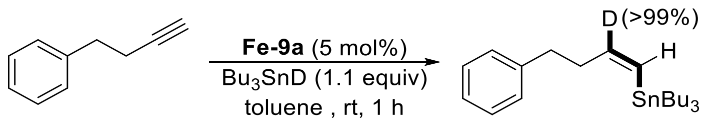

Isotopologue [D]-2a. A 25 mL Schlenk flask was charged with complex **9a** (3.5 mg, 5 mol%) and toluene (2 mL). A mixture of alkyne **1** (26 mg, 0.2 mmol) and $DSnBu_3$ (64 mg, 0.22 mmol) in toluene (2 mL) was added via syringe pump at ambient temperature over 15 min, causing a color change from greenish yellow to dark brown. Once TLC indicated complete conversion, the solvent was removed, the residue was dissolved in hexane, the resulting suspension was filtered through a pad of Celite, the filtrate was evaporated and the crude product inspected by ^{1}H NMR to determine the isomer ratio. The residue was then purified by flash chromatography (hexane, + 1-2% Et_3N) to furnish the title compound as a light yellow liquid (70 mg, 84%). ^{1}H NMR (400 MHz, $CDCl_3$) δ 7.31-7.27 (m, 2H), 7.22-7.17 (m, 3H), 5.85 (s, 1H), 2.72-2.68 (m, 2H), 2.38-2.32 (m, 2H), 1.53-1.42 (m, 6H), 1.34-1.27 (m, 6H), 0.92-0.85 (m, 15H). ^{2}H NMR (92 MHz, $CDCl_3$) δ 6.57. ^{13}C NMR (101 MHz, $CDCl_3$) δ 148.1, 142.0, 128.6, 128.5, 126.0, 39.1, 36.5, 29.3, 27.5, 13.9, 10.4. ^{119}Sn NMR (149 MHz, $CDCl_3$) δ –60.9.

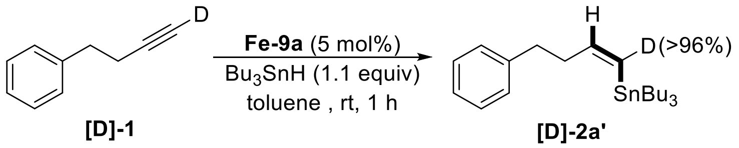

Isotopologue [D]-2a'. Prepared analogously from alkyne **[D]-1** (26 mg, 0.2 mmol) and Bu_3SnH (63 mg, 0.22 mmol) as a light yellow liquid (72 mg, 86%). ^{1}H NMR (400 MHz, $CDCl_3$) δ 7.31-7.27 (m, 2H), 7.22-7.17 (m, 3H), 6.60 (t, $^{3}J_{H\text{-}H}$ = 7.1 Hz, 1H), 2.72-2.68 (m, 2H), 2.38-2.32 (m, 2H), 1.53-1.42 (m, 6H), 1.34-1.27 (m, 6H), 0.92-0.85 (m, 15H). ^{2}H NMR (92 MHz, $CDCl_3$) δ 5.92. ^{13}C NMR (101 MHz, $CDCl_3$) δ 148.1, 142.0, 128.6, 128.5, 126.0, 39.1, 36.5, 29.3, 27.5, 13.9, 10.4. ^{119}Sn NMR (149 MHz, $CDCl_3$) δ –60.9.

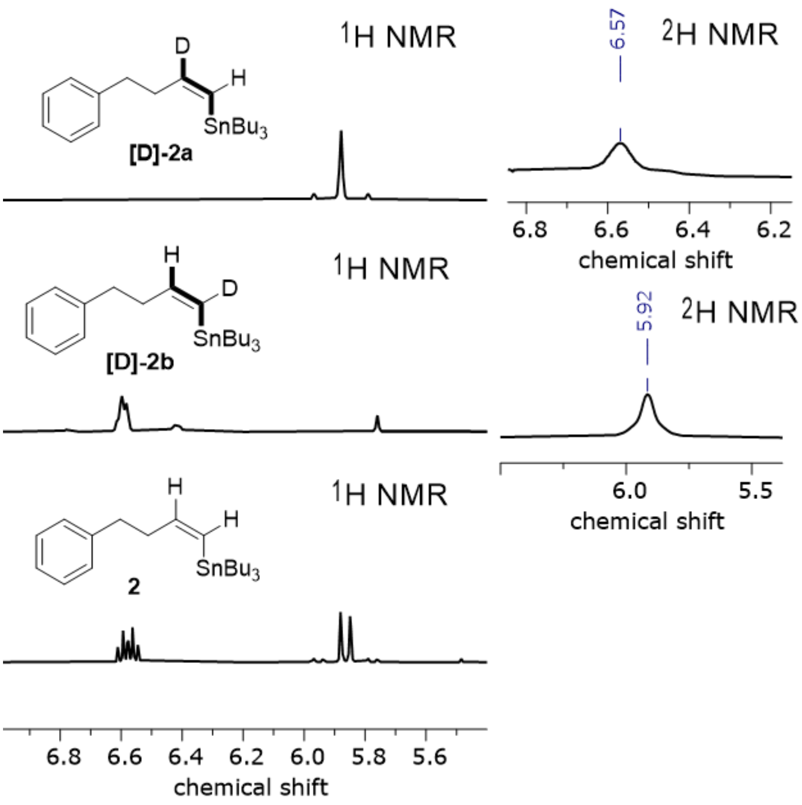

Figure S5. ^{1}H and ^{2}H NMR spectra (olefinic region) of the deuterium labelled products **[D]-2a** and **[D]-2a'** in comparison to product **2**

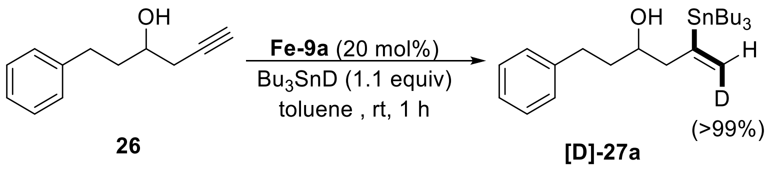

Isotopologue [D]-27a. Prepared analogously from alkyne **26** (34 mg, 0.2 mmol) and Bu_3SnD (64 mg, 0.22 mmol) as a light yellow oil (76 mg, 81%). ^{1}H NMR (400 MHz, $CDCl_3$) δ 7.32-7.28 (m, 2H), 7.25-7.18 (m, 3H), 5.33 (d, $^{2}J_{H\text{-}H}$ = 2.8 Hz, 1H), 3.63-3.56 (m, 1H), 2.88-2.81 (m, 1H), 2.77-2.69 (m, 1H), 2.55 (d, J = 14.3 Hz, 1H), 2.36-2.30 (m, 3H), 1.87-1.75 (m, 6H), 1.56-1.46 (m, 7H), 0.97-0.83 (m, 15H). ^{2}H NMR (92 MHz, $CDCl_3$) δ 5.84. ^{13}C NMR (101 MHz, $CDCl_3$) δ 152.3, 142.2, 128.6, 128.5, 125.9, 69.1, 38.7, 32.2, 29.2, 27.5, 13.8, 9.8. ^{119}Sn NMR (149 MHz, $CDCl_3$) δ –44.7.

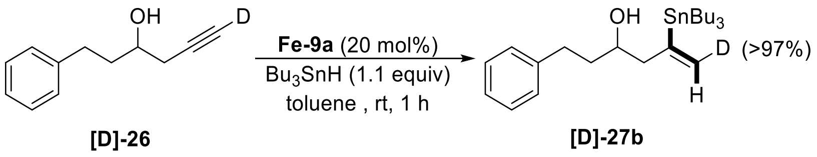

Isotopologue [D]-27b. Prepared analogously from alkyne **[D]-26** (34 mg, 0.2 mmol) and Bu_3SnH (63 mg, 0.22 mmol) as a light yellow oil (78 mg, 84%). ^{1}H NMR (400 MHz, $CDCl_3$) δ 7.32-7.28 (m, 2H), 7.25-7.18 (m, 3H), 5.82 (d, $^{2}J_{H\text{-}H}$ = 2.7 Hz, 1H), 3.63-3.56 (m, 1H), 2.88-2.81 (m, 1H), 2.77-2.69 (m, 1H), 2.55 (d, J = 14.3 Hz, 1H), 2.36-2.30 (m, 3H), 1.87-1.75 (m, 6H), 1.56-1.46 (m, 7H), 0.97-

0.83 (m, 15H). ^{2}H NMR (92 MHz, $CDCl_3$) δ 5.36. ^{13}C NMR (101 MHz, $CDCl_3$) δ 152.3, 142.2, 128.6, 128.5, 125.9, 69.1, 38.7, 32.2, 29.2, 27.5, 13.8, 9.8. ^{119}Sn NMR (149 MHz, $CDCl_3$) δ –44.7.

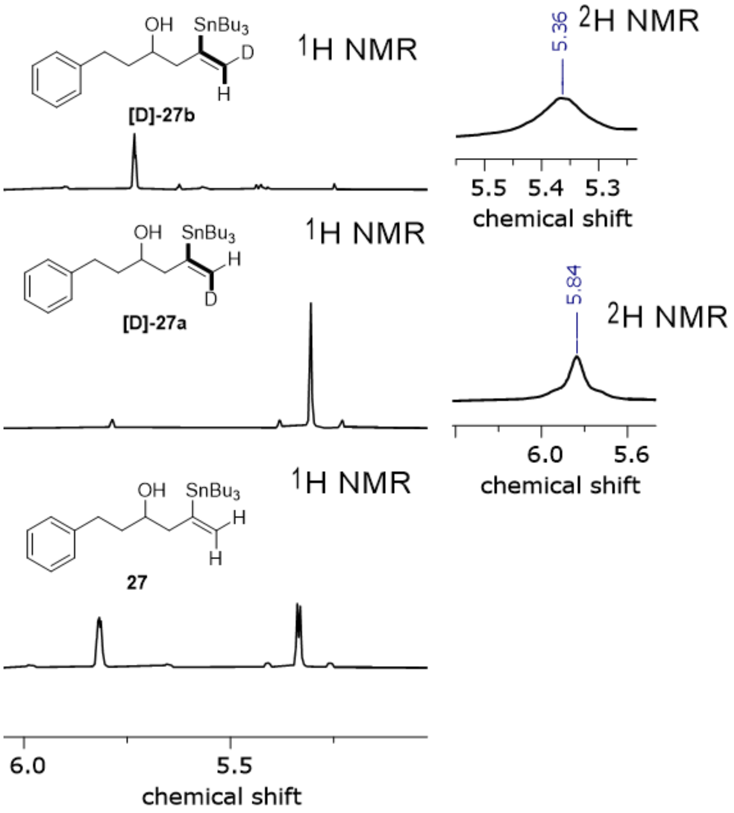

Figure S6. ^{1}H and ^{2}H NMR spectra (olefinic region) of the deuterium labelled products **[D]-27a** and **[D]-27b** in comparison with product **27**. The assignment of the proton *trans* to the –$SnBu_3$ moiety is based, i. a., on an NOE to the –C<u>H</u>$_2$CH(OH)- group, whereas the proton *cis* to it shows an NOE to the butyl substituents of the –$SnBu_3$ moiety.

Determination of the Kinetic Isotope Effect

Intermolecular Competition Experiment (Bu_3SnH versus Bu_3SnD)

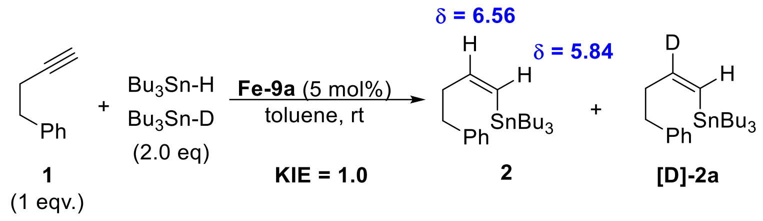

A 25 mL Schlenk flask was loaded with the Fe-catalyst **Fe-9a** (3.5 mg, 0.01 mmol) and toluene (2 mL). A mixture of substrate **1** (26 mg, 0.2 mmol), $HSnBu_3$ (116 mg, 0.4 mmol) and $DSnBu_3$ (116 mg, 0.4 mmol) in toluene (2 mL) was added via syringe pump at ambient temperature over 15 min causing a color change from greenish yellow to dark brown. The mixture was stirred for 30 min, the solvent was removed in vacuo, the residue was dispersed in hexane, the suspension was filtered through a plug of Celite, the filtrate was evaporated and the residue inspected by ^1H NMR to determine the isomer ratio.

Based on the relative integral of the $-CH_{internal}$ (I = 1.00, δ = 6.56, *trans* to the $SnBu_3$ group) and $-CH_{terminal}$ (I = 2.01, δ = 5.84, *cis* to the $SnBu_3$ group), the ratio of **2/[D]-2a** was determined to be **1.0**.

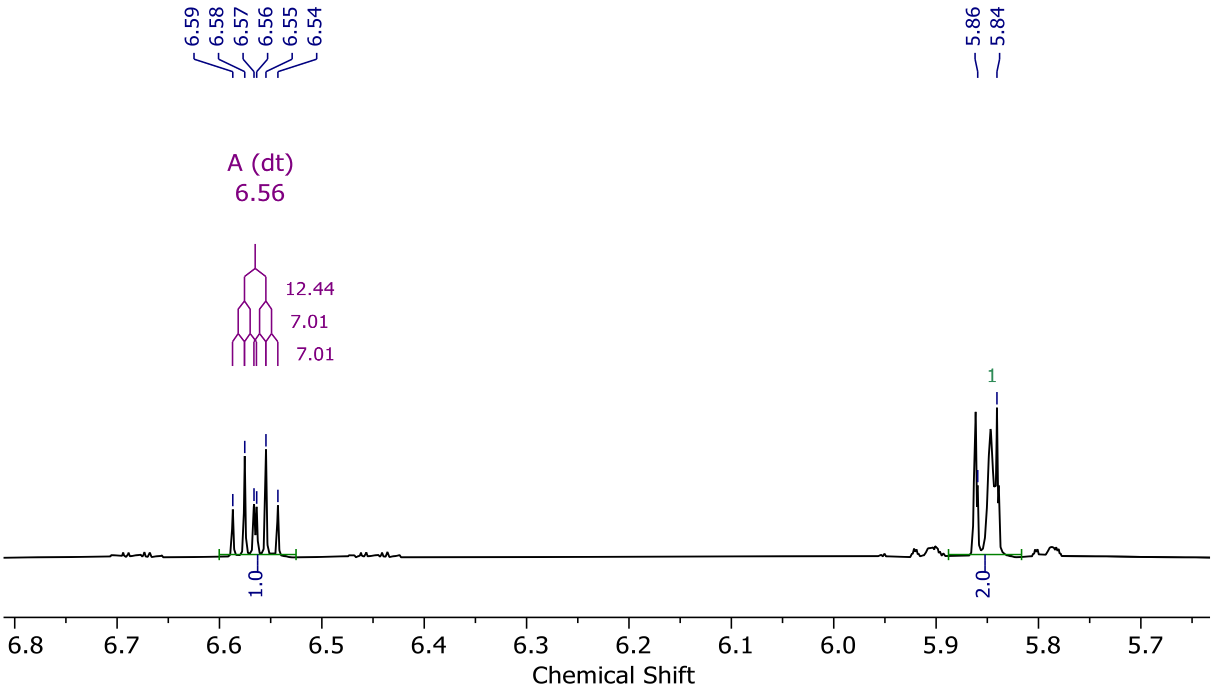

Figure S7. Olefinic region of the ^1H NMR spectrum of the crude product mixture **2/[D]-2a** obtained in the intermolecular competition experiment.

Intermolecular Competition Experiment (1 versus [D]-1)

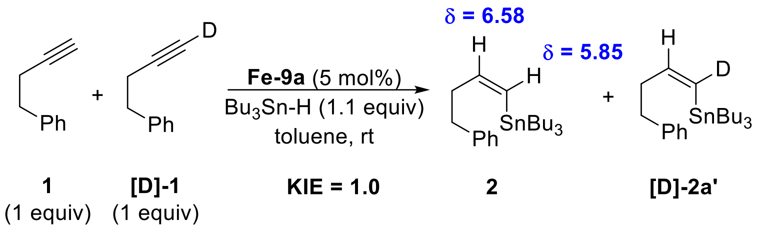

A 25 mL Schlenk flask was loaded with the Fe-catalyst **Fe-9a** (3.5 mg, 0.01 mmol) and toluene (2 mL). A mixture of substrate **1** (26 mg, 0.2 mmol), **[D]-1** (26 mg, 0.2 mmol) and $HSnBu_3$ (63 mg, 0.22 mmol) in toluene (2 mL) was added via syringe pump at ambient temperature over 15 min causing a color change from greenish yellow to dark brown. The mixture was stirred for 60 min, the solvent was removed in vacuo, the residue was dispersed in hexane, the suspension was filtered through a plug of Celite, the filtrate was evaporated and the residue inspected by ^1H NMR to determine the isomer ratio.

Based on the relative integral of the $-CH_{internal}$ (I = 2.00, δ = 6.58, *trans* to the $-SnBu_3$ group) and $-CH_{terminal}$ (I = 1.00, δ = 5.85, *cis* to the $-SnBu_3$ group), the ratio of ratio of **2a/[D]-2a’** was determined to be **1.0**.

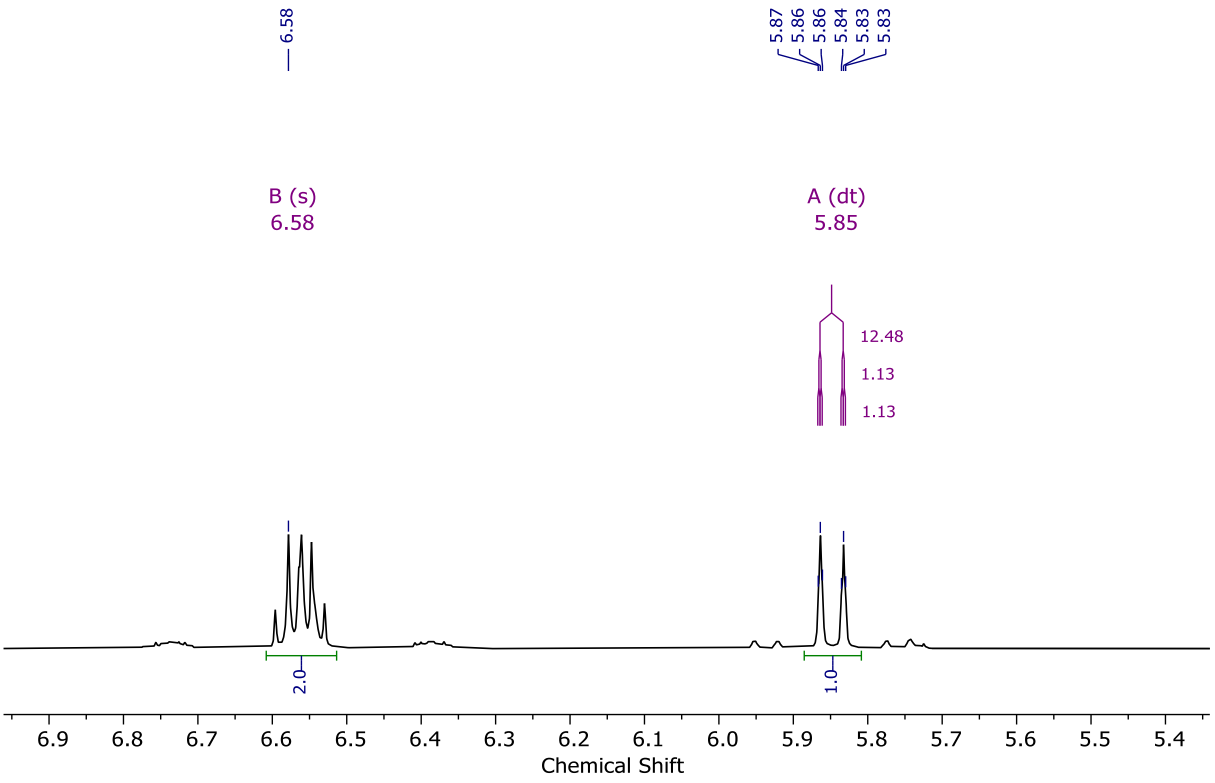

Figure S8. Olefinic region of the ^1H NMR spectrum of the crude product obtained in the intermolecular competition experiment (**1** versus **[D]-1**)

Intermolecular Competition Experiment Using Substrate 26 (Bu_3SnH versus Bu_3SnD)

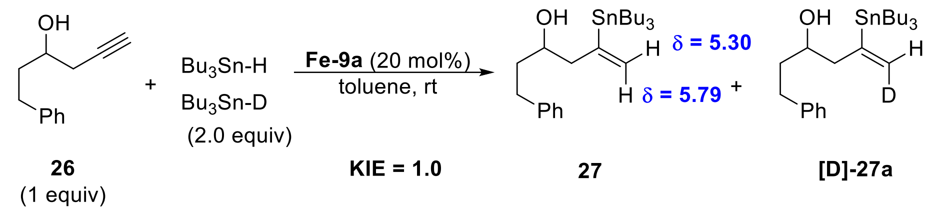

The reaction was performed analogously using substrate **26** (34 mg, 0.2 mmol), $HSnBu_3$ (116 mg, 0.4 mmol) and $DSnBu_3$ (116 mg, 0.4 mmol).

Based on the relative integral of the –CH (I = 1.00, δ = 5.79, *trans* to the $SnBu_3$ group) and –CH (I = 2.06, δ = 5.30, *cis* to the $SnBu_3$ group), the ratio of products **27/[D]-27a** was **1.0**.

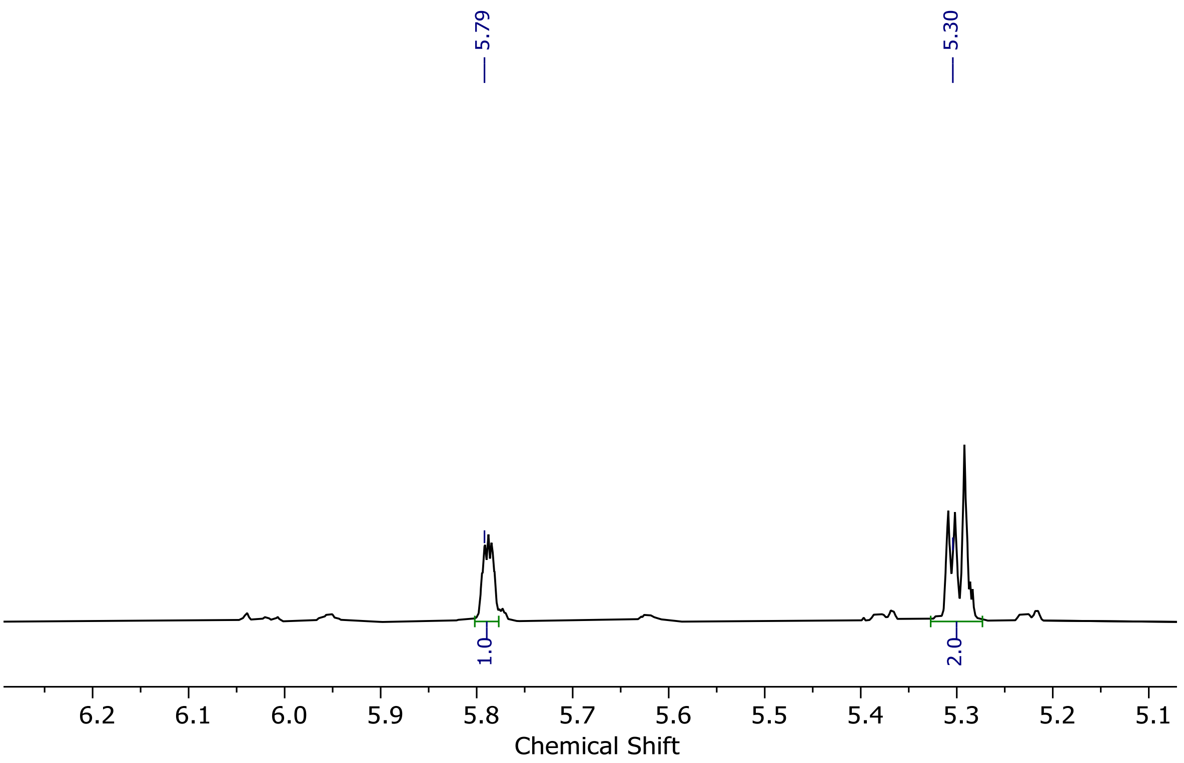

Figure S9. Olefinic region of the ^1H NMR spectrum of the crude product mixture **27/[D]-27a** obtained in the intermolecular competition experiment.

Intermolecular Competition Experiment (26 versus [D]-26)

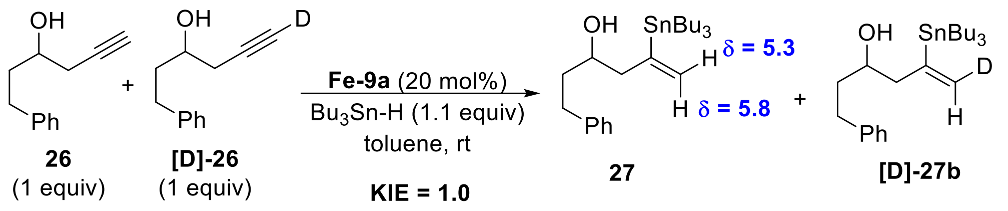

The reaction was performed analogously using substrate **26** (34 mg, 0.2 mmol), **[D]-26** (34 mg, 0.2 mmol) and $HSnBu_3$ (63 mg, 0.22 mmol)

Based on the relative integral of the -CH (I = 1.98, δ = 5.80, *trans* to the $-SnBu_3$ group) and -CH (I = 1.01, δ = 5.31, *cis* to the $-SnBu_3$ group), the ratio of product **27/[D]-27b** was **1.0**.

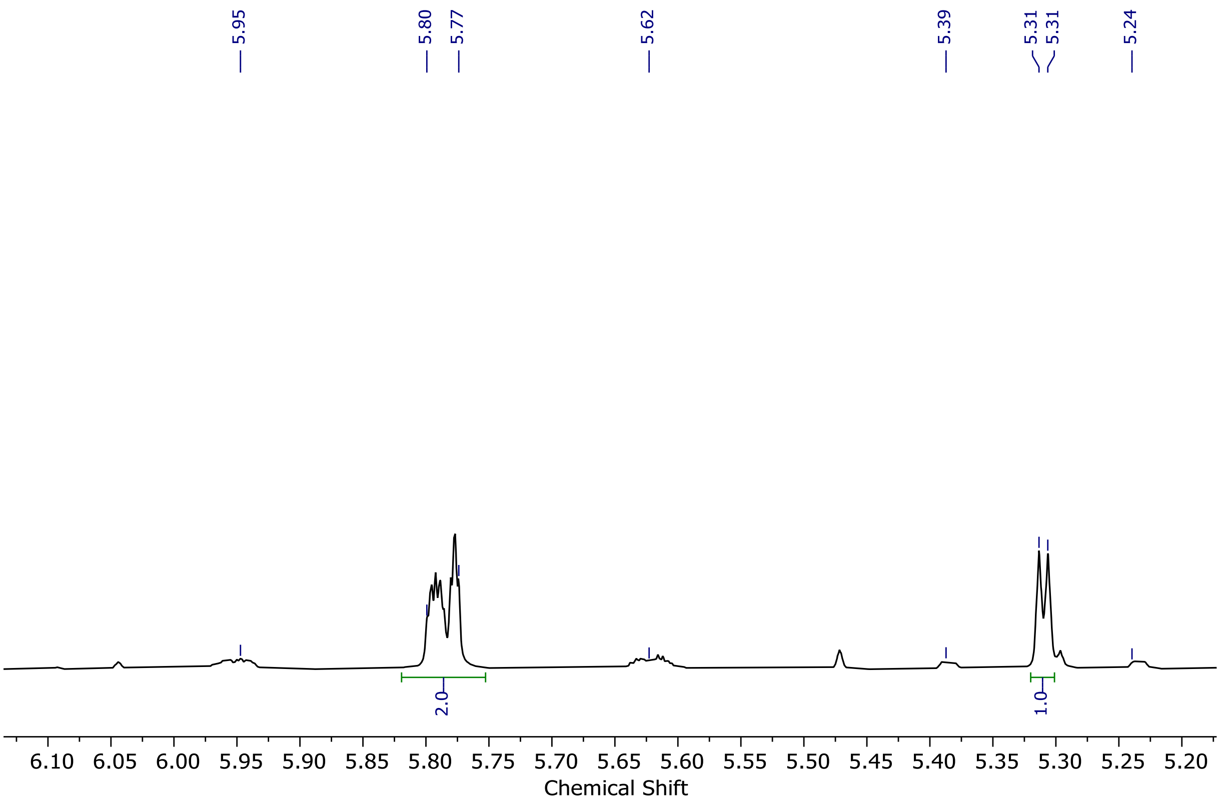

Figure S10. Olefinic region of the ^{1}H NMR spectrum of the crude product obtained in the intermolecular competition experiment (**26** versus **[D]-26**)

Stannylative Cyclization Reactions: Isotope Labelling Experiments

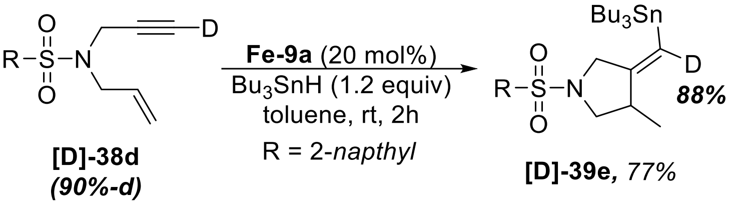

Compound [D]-39e. A 25 mL Schlenk flask was loaded with complex **Fe-9a** (14 mg, 0.04 mmol) and toluene (2 mL). A mixture of the deuterium labelled alkyne **[D]-38d** (90% deuteration, 57 mg, 0.2 mmol) and $HSnBu_3$ (70 mg, 0.24 mmol) in toluene (2 mL) was added with a syringe pump at ambient temperature over 15 min, causing a color change from greenish yellow to dark brown. The mixture was stirred for 2 h before the solvent was removed, the residue was suspended in hexane (4 mL), and the resulting suspension filtered through a plug of Celite. The filtrate was evaporated and the product ratio determined by recording a ^{1}H NMR spectrum of the crude material. The product was purified by flash chromatography (hexane/ethyl acetate, 4/1 + 1-2% Et_3N) to furnish the product as a colorless liquid (89 mg, 77%). ^{1}H NMR (400 MHz, $CDCl_3$) δ 8.39 (s, 1H), 8.00-7.96 (m, 2H), 7.93-7.91 (m, 1H), 7.83-7.81 (m, 1H), 5.67-5.66 (m, 2H), 5.65 (q, J = 2.2 Hz, 0.12H, 88% D-incorporation), 3.96 (dd, J = 13.9 Hz, 1H), 3.71-3.66 (m, 2H), 2.76-2.64 (m, 7H), 1.41-1.37 (m, 7H), 1.02 (d, J = 6.5 Hz, 3H), 0.88-0.82 (m, 15H). ^{2}H NMR (92 MHz, $CDCl_3$) δ 5.73. ^{13}C NMR (101 MHz, $CDCl_3$) δ 158.0, 135.0, 133.3, 132.4, 129.4, 129.1, 128.9, 128.0, 127.7, 123.3, 55.4, 53.7, 40.1, 29.2, 27.3, 27.0, 16.2, 13.7, 9.8. ^{119}Sn NMR (149 MHz, $CDCl_3$) δ –53.9.

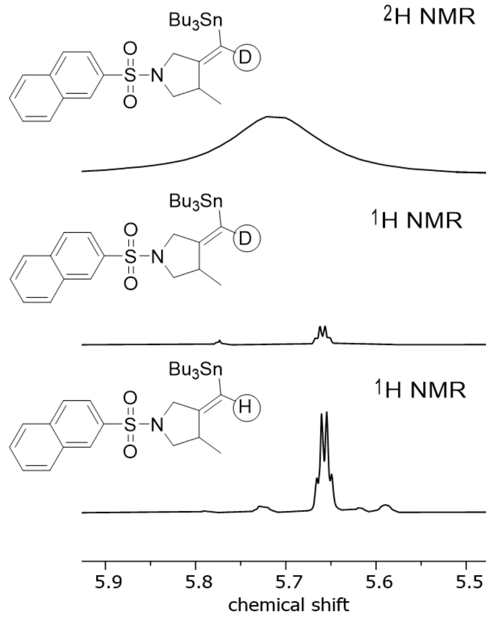

Figure S11. Olefinic region of the ^{1}H and ^{2}H NMR spectra of **[D]-39e** and unlabeled **39d.**

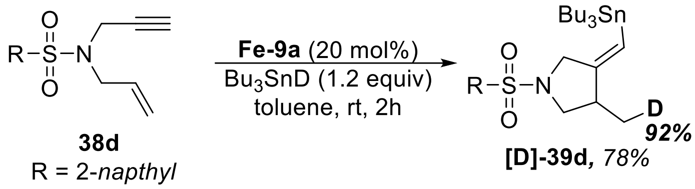

Compound [D]-39d. Prepared analogously using enyne **38d** (56 mg, 0.2 mmol), $DSnBu_3$ (71 mg, 0.24 mmol) and complex **Fe-9a** (14 mg, 0.04 mmol); colorless liquid (89 mg, 78%). ^1H NMR (400 MHz, $CDCl_3$) δ 8.39 (s, 1H), 8.00-7.96 (m, 2H), 7.93-7.91 (m, 1H), 7.83-7.81 (m, 1H), 5.67-5.66 (m, 2H), 5.65 (q, *J* = 2.2 Hz, 1H), 3.96 (dd, *J* = 14.0 Hz, 1H), 3.71-3.66 (m, 2H), 2.76-2.64 (m, 7H), 1.41-1.37 (m, 7H), 1.02 (d, *J* = 6.5 Hz, 2.08H, 92% D-incorporation), 0.88-0.82 (m, 15H). ^2H NMR (92 MHz, $CDCl_3$) δ 1.03. ^{13}C NMR (101 MHz, $CDCl_3$) δ 158.0, 135.0, 133.0, 132.4, 129.4, 129.1, 128.9, 128.0, 127.7, 123.3, 118.4, 55.4, 53.7, 40.1, 29.1, 27.3, 27.1, 13.7, 9.9. ^{119}Sn NMR (149 MHz, $CDCl_3$) δ –53.6.

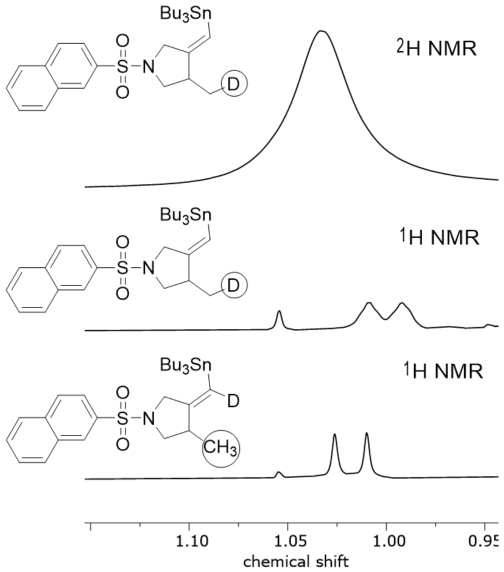

Figure S12. Region of the signals of the methyl groups in the ^1H and ^2H NMR spectra of **[D]-39d** and **[D]-39e.**

Alkyne Hydrostannlyation

Characteristic Spectral Features of Representative Alkenylstannanes

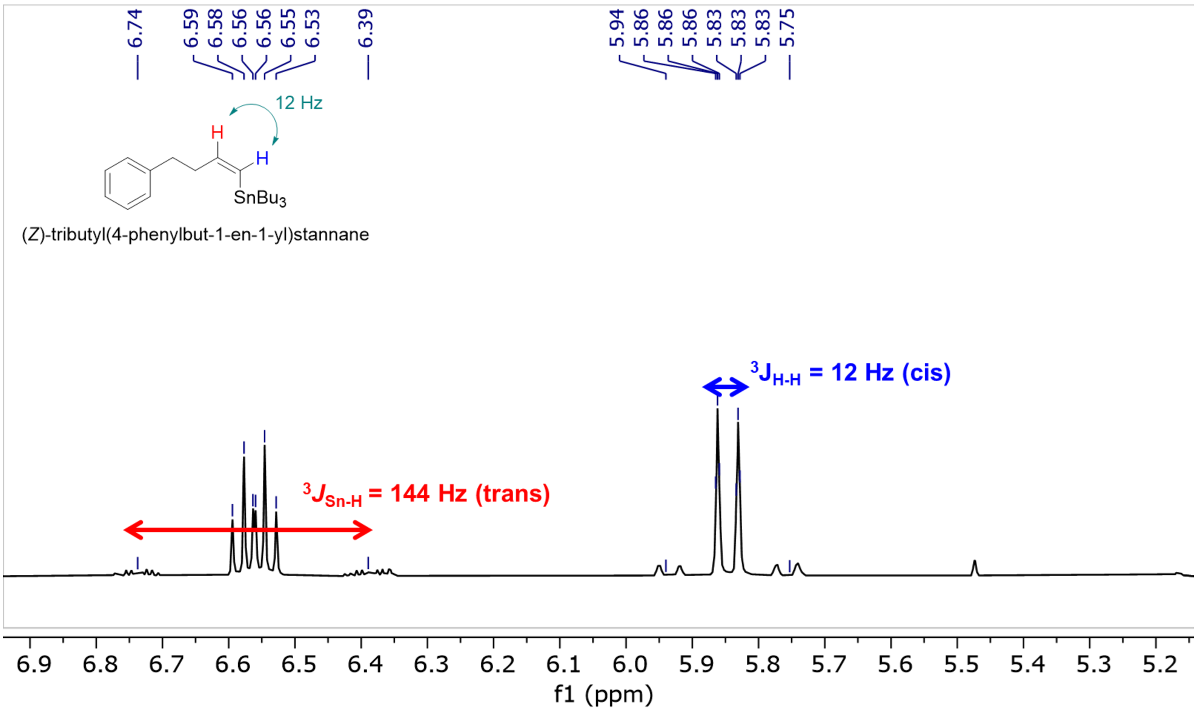

Figure S13. Olefinic region of the ^{1}H NMR spectrum of compound **2** prepared by Fe-catalyzed *trans*-hydrostannation, showing the characteristic satellites caused by coupling with the ^{117}Sn/^{119}Sn nuclei as well as the $^{3}J_{H,H}$ characteristic of a *cis*-alkene.

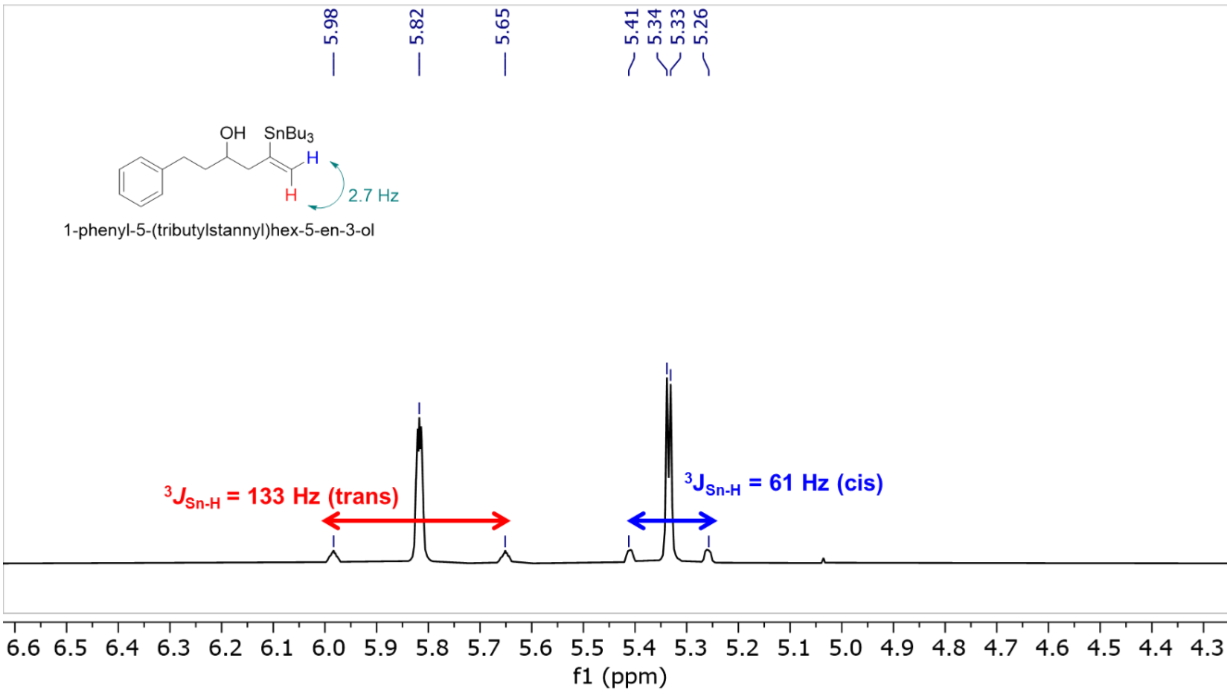

Figure S14. Olefinic region of the ^{1}H NMR spectrum of compound **27** prepared by Fe-catalyzed *trans*-hydrostannylation showing the characteristic satellites caused by coupling with the ^{117}Sn/^{119}Sn nuclei.

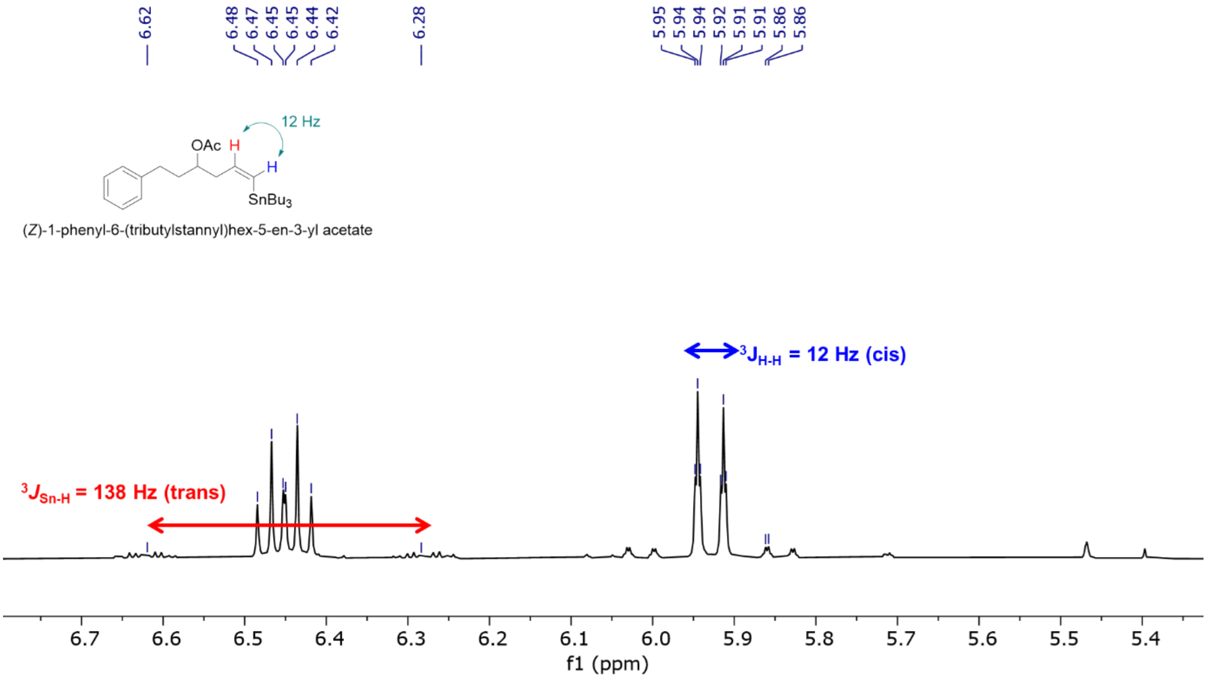

Figure S15. Olefinic region of the ^{1}H NMR spectrum of compound **28b** prepared by Fe-catalyzed *trans*-hydrostannylation showing the characteristic satellites caused by coupling with the ^{117}Sn/ ^{119}Sn nuclei as well as the $^3J_{H,H}$ characteristic of a *cis*-alkene.

Remarks: *Freshly distilled toluene or THF are required for the hydrostannylation reaction. A brown coloration of the catalyst solution was observed when poor quality solvent was used, which must be avoided. The tributyltin hydride should not be added at once to the iron complex, as such mixtures showed no long-term stability.*

Representative Procedure. A 25 mL Schlenk flask was charged with complex **Fe-9a** (5 mol%, 3.5 mg or 20 mol%, 14 mg; for 0.2 mmol scale of alkyne) and toluene (2 mL). A mixture of the respective substrate (1 equiv.) and $HSnBu_3$ (1.1 equiv.) in toluene (2 mL) was added with a syringe pump at ambient temperature over 15 min. A color change from greenish yellow to dark brown was observed and the mixture was stirred for the required time (2 h to 18 h), monitoring the reaction by TLC. The solvent was removed upon completion of the reaction, the residue was dissolved in hexane (or cyclohexane), the resulting suspension was filtered through a plug of Celite, the filtrate was evaporated, and the residue inspected by ^{1}H NMR to determine the product ratio (proximal/distal addition). The residue was then purified by flash chromatography (hexane/ethyl acetate + 1-2% Et_3N).

(*Z*)-Tributyl(4-phenylbut-1-en-1-yl)stannane (**2a**). **Large-Scale Experiment.** A 25 mL Schlenk flask was charged with complex **Fe-9a** (35 mg, 5 mol%) and toluene (5 mL). A mixture of alkyne **1** (260 mg, 2 mmol) and $HSnBu_3$ (640 mg, 2.2 mmol) in toluene (5 mL) was added with a syringe pump at ambient temperature over 15 min. A color change from greenish yellow to dark brown was observed and the mixture was stirred for 2 h monitoring the reaction by TLC. The solvent was removed upon completion of the reaction, hexane was added, the resulting suspension was filtered through a plug of Celite, the filtrate was evaporated, and the residue inspected by ^{1}H NMR to determine the product ratio (proximal/distal addition). The residue was then purified by flash chromatography (hexane + 1-2% Et_3N) to afford product **2** as colorless liquid (717 mg, 86%). ^{1}H NMR (400 MHz, $CDCl_3$) δ 7.31-7.27 (m, 2H), 7.22-7.17 (m, 3H), 6.55 ($^3J_{Sn\text{-}H}$ = 140.0 Hz, 1H, *trans* H), 5.54 ($^3J_{Sn\text{-}H}$ =

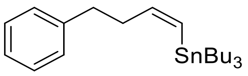

76.0 Hz, 1H, *cis* H), 2.72-2.68 (m, 2H), 2.38-2.32 (m, 2H), 1.53-1.42 (m, 6H), 1.34-1.27 (m, 6H), 0.92-0.85 (m, 15H). ^{13}C NMR (101 MHz, $CDCl_3$) δ 148.2, 142.0, 128.9, 128.6, 128.5, 126.0, 39.1, 36.5, 29.4, 27.5, 13.9, 10.4. ^{119}Sn NMR (149 MHz, $CDCl_3$) δ –60.7. ESI-MS calcd. for $C_{22}H_{37}Sn$ $[M-H]^+$ 421.19117; found 421.19142.

The following compounds were prepared analogously

(*Z*)-Triphenyl(4-phenylbut-1-en-1-yl)stannane (**2b**). Pale yellow liquid (75 mg, 79%). ^{1}H NMR (400 MHz, $CDCl_3$) δ 7.59-7.56 (m, 6H), 7.39-7.36 (m, 10H), 7.18-7.11 (m, 3H), 6.86 (dt, $^3J_{H-H}$ = 12.1 Hz, 1H), 6.79 (dq, *J* = 7.9 Hz, 1H), 6.18 (dq, $^3J_{H-H}$ = 12.1 Hz, 1H), 2.57-2.53 (m, 2H), 2.43-2.37 (m, 2H). ^{13}C NMR (101 MHz, $CDCl_3$) δ 151.6, 141.5, 139.2, 137.1, 129.1, 128.7, 128.6, 128.3, 125.9, 125.1, 39.5, 35.9. ^{119}Sn NMR (149 MHz, $CDCl_3$) δ –149.8. API-MS (HRMS) calcd. for $C_{28}H_{26}Sn$ $[M]^+$ 482.10529; found 482.10510.

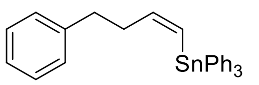

(*Z*)-Tributyl(oct-1-en-1-yl)stannane (**3**). Colorless liquid (76 mg, 95%). ^{1}H NMR (400 MHz, $CDCl_3$) δ 6.51 (d, $^3J_{H-H}$ = 12.4 Hz, 1H), 5.77 (d, $^3J_{H-H}$ = 13.5 Hz, 1H), 2.04-1.99 (m, 2H), 1.54-1.46 (m, 10H), 1.35-1.28 (m, 12H), 0.90-0.89 (m, 16H). ^{13}C NMR (101 MHz, $CDCl_3$) δ 149.5, 127.7, 37.3, 32.1, 30.0, 29.4, 27.5, 22.8, 13.8, 10.3. ^{119}Sn NMR (149 MHz, $CDCl_3$) δ –60.8. API-MS calcd. for $C_{20}H_{41}Sn$ $[M-H]^+$ 401.22247; found 401.22186.

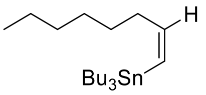

(*Z*)-Tributyl(4-methylpent-1-en-1-yl)stannane (**4**). Colorless liquid (68 mg, 92%). ^{1}H NMR (400 MHz, $CDCl_3$) δ 6.53 (dt, $^3J_{H-H}$ = 12.5 Hz, 1H), 5.82 (d, $^3J_{H-H}$ = 13.7 Hz, 1H), 1.92 (td, *J* = 7.0 Hz, 2H), 1.52-1.47 (m, 5H), 1.34-1.29 (m, 9H), 0.92-0.88 (m, 20H). ^{13}C NMR (101 MHz, $CDCl_3$) δ 148.3, 128.7, 46.4, 29.4, 27.5, 22.6, 13.8, 10.4. ^{119}Sn NMR (149 MHz, $CDCl_3$) δ –61.7.

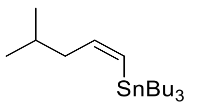

(*Z*)-Tributyl(3-cyclopentylprop-1-en-1-yl)stannane (**5**). Colorless liquid (65 mg, 82%). ^{1}H NMR (400 MHz, $CDCl_3$) δ 6.57 (dt, $^3J_{H-H}$ = 12.4 Hz, 1H), 5.78 (d, $^3J_{H-H}$ = 11.1 Hz, 1H), 2.04 (td, *J* = 7.0 Hz, 2H), 1.53-1.48 (m, 9H), 1.35-1.28 (m, 10H), 0.91-0.88 (m, 17H). ^{13}C NMR (101 MHz, $CDCl_3$) δ 148.8, 128.1, 43.4, 40.5, 32.6, 29.4, 27.6, 25.3, 13.9, 10.4. ^{119}Sn NMR (149 MHz, $CDCl_3$) δ –61.4.

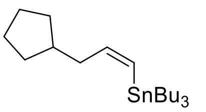

(1*Z*,7*Z*)-1,8-Bis(tributylstannyl)octa-1,7-diene (**6**). Colorless liquid (110 mg, 84%). ^{1}H NMR (400 MHz, $CDCl_3$) δ 6.51 (dt, $^3J_{H-H}$ = 12.3 Hz, 2H), 5.78 (dt, $^3J_{H-H}$ = 12.4 Hz, 2H), 2.04 (br, 5H), 1.53-1.46 (m, 15H), 1.34-1.28 (m, 12H), 0.92-0.87 (m, 30H). ^{13}C NMR (101 MHz, $CDCl_3$) δ 149.1, 128.1, 37.4, 29.9, 29.4, 27.5, 13.9, 10.4. ^{119}Sn NMR (149 MHz, $CDCl_3$) δ –60.8.

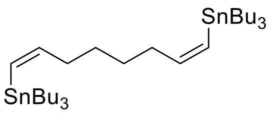

Dimethyl (*Z*)-2-(3-(tributylstannyl)allyl)malonate (**7**). Colorless liquid (78 mg, 85%). ^{1}H NMR (400 MHz, $CDCl_3$) δ 6.41 (dt, $^3J_{H-H}$ = 12.6 Hz, 1H), 5.96 (dt, $^3J_{H-H}$ = 12.5 Hz, 1H), 3.72 (s, 6H), 3.40 (t, *J* = 7.6 Hz, 1H), 2.62 (t, *J* = 7.8 Hz, 2H), 1.51-1.45 (m, 6H), 1.33-1.26 (m, 6H), 0.94- 0.87 (m, 15H). ^{13}C NMR (101 MHz, $CDCl_3$) δ 169.4, 143.6, 132.6, 52.6, 52.1, 35.7, 29.3, 27.5, 27., 13.7, 10.3. ^{119}Sn NMR (149 MHz, $CDCl_3$) δ -60.22. API-MS calcd. for $C_{20}H_{38}O_4Sn$+Na $[M+Na]^+$ 485.16843; found 485.16882.

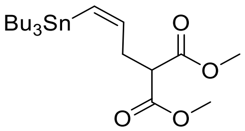

(*Z*)-Tributyl(5-chloropent-1-en-1-yl)stannane (**8**). Colorless liquid (79 mg, 95%). ^{1}H NMR (400 MHz, $CDCl_3$) δ 6.48 (dt, $^3J_{H-H}$ = 12.4 Hz, 1H), 5.87 (dt, $^3J_{H-H}$ = 12.4 Hz, 1H), 3.55 (t, *J* 6.6 Hz, 2H), 2.21-2.16 (m, 2H), 1.90-1.85 (m, 2H), 1.52-1.48 (m, 5H), 1.34-1.29 (m, 7H), 0.92-0.89 (m, 15H). ^{13}C NMR (101 MHz, $CDCl_3$) δ 147.0, 130.0, 44.7, 34.5, 32.8, 29.5, 27.4, 13.8, 10.5. ^{119}Sn NMR (149 MHz, $CDCl_3$) δ –60.4. API-MS (HRMS) calcd. for $C_{17}H_{35}ClSn$+Na $[M+Na]^+$ 417.20515; found 417.20498

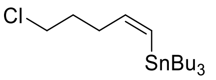

(*Z*)-6-(Tributylstannyl)hex-5-en-1-yl benzoate (**9a**). Pale yellow liquid (89 mg, 89%). ^{1}H NMR (400 MHz, $CDCl_3$) δ 8.06-8.04 (m, 2H), 7.57-7.53 (m, 1H), 7.45-7.42 (m, 2H), 6.53 (dt, $^{3}J_{H\text{-}H}$ = 12.4 Hz, 1H), 5.83 (dt, $^{3}J_{H\text{-}H}$ = 12.4 Hz, 1H), 4.33 (t, J = 6.6 Hz, 2H), 2.14-2.08 (m, 2H), 1.84-1.79 (m, 2H), 1.61-1.47 (m, 8H), 1.34-1.28 (m, 6H), 0.95- 0.87 (m, 15H). ^{13}C NMR (101 MHz, $CDCl_3$) δ 166.8, 148.5, 132.9, 130.6, 129.7, 128.8, 128.4, 65.1, 36.8, 29.3, 28.7, 27.4, 27.0, 26.5, 13.8, 10.4. ^{119}Sn NMR (149 MHz, $CDCl_3$) δ –60.7. API-MS (HRMS) calcd. for $C_{25}H_{42}O_2Sn$+Na [M+Na]$^+$ 517.20990; found 517.20984.

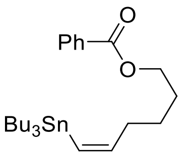

(*Z*)-*tert*-butyldimethyl((6-(tributylstannyl)hex-5-en-1-yl)oxy)silane (**9b**). Colorless liquid (84 mg, 82%). ^{1}H NMR (400 MHz, $CDCl_3$) δ 6.52 (dt, $^{3}J_{H\text{-}H}$ = 12.4 Hz, 1H), 5.80 (dt, $^{3}J_{H\text{-}H}$ = 12.3 Hz, 1H), 3.63 (t, J = 6.5 Hz, 2H), 2.12-2.05 (m, 2H), 1.66-1.59 (m, 4H), 1.53-1.46 (m, 7H), 1.34-1.29 (m, 8H), 0.91-0.88 (m, 21H), 0.06 (s, 6H). ^{13}C NMR (101 MHz, $CDCl_3$) δ 148.8, 128.2, 63.1, 33.8, 33.3, 29.4, 27.5, 27.1, 26.1, 13.9, 10.4, –5.14. ^{119}Sn NMR (149 MHz, $CDCl_3$) δ –60.7. API-MS (HRMS) calcd. for $C_{24}H_{52}OSiSn$+Na [M+Na]$^+$ 527.24136; found 527.24209

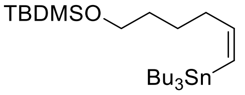

(*Z*)-Tributyl(6-((tetrahydro-2*H*-pyran-2-yl)oxy)hex-1-en-1-yl)stannane (**9c**). Colorless liquid (88 mg, 92%). ^{1}H NMR (400 MHz, $CDCl_3$) δ 6.51 (dt, $^{3}J_{H\text{-}H}$ = 12.4 Hz, 1H) , 5.79 (dt, $^{3}J_{H\text{-}H}$ = 12.4 Hz, 1H), 4.57 (br, 1H), 3.89-3.84 (m, 1H), 3.77-3.71 (m, 1H), 3.52-3.46 (m, 1H), 3.42-3.36 (m, 1H), 2.04 (br, 4H), 1.67-1.57 (4H), 1.52- 1.45 (m, 10H), 1.34-1.27 (m, 6H), 0.92-0.87 (m, 15H). ^{13}C NMR (101 MHz, $CDCl_3$) δ 149.0, 128.2, 98.9, 67.6, 62.4, 37.2, 30.8, 29.7, 29.3, 27.5, 26.8, 25.7, 19.8, 13.8, 10.1. ^{119}Sn NMR (149 MHz, $CDCl_3$) δ –60.8. API-MS (HRMS) calcd. for $C_{23}H_{46}NaO_2Sn$+Na [M+Na]$^+$ 497.24083; found 497.241129.

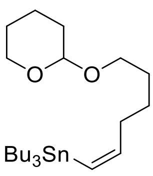

(*Z*)-Tributyl(6-(trityloxy)hex-1-en-1-yl)stannane (**9d**). Pale yellow liquid (115 mg, 94%). ^{1}H NMR (400 MHz, $CDCl_3$) δ 7.46-7.44 (m, 7H), 7.32-7.28 (m, 7H), 7.24-7.21 (m, 3H), 6.50 (dt, $^{3}J_{H\text{-}H}$ = 12.4 Hz, 1H), 5.79 (dt, $^{3}J_{H\text{-}H}$ = 12.4 Hz, 1H), 3.07 (t, J = 6.6 Hz, 2H), 2.04-1.99 (m, 2H), 1.53-1.47 (m, 6H), 1.34- 1.28 (m, 7H), 0.91-0.87 (m, 16H). ^{13}C NMR (101 MHz, $CDCl_3$) δ 149.1, 144.6, 128.8, 127.8, 126.9, 86.5, 63.8, 37.2, 29.3, 27.5, 27.1, 13.9, 10.4. ^{119}Sn NMR (149 MHz, $CDCl_3$) δ –60.8. API-MS (HRMS) calcd. for $C_{37}H_{52}OSn$+Na [M+Na]$^+$ 655.29323; found 655.29287.

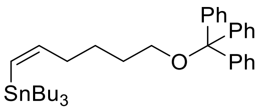

(*Z*)-6-(Tributylstannyl)hex-5-en-1-yl furan-3-carboxylate (**10**). Colorless liquid (92 mg, 95%). ^{1}H NMR (400 MHz, $CDCl_3$) δ 8.01 (s, 1H), 7.41 (t, J = 1.7 Hz, 1H), 6.74 (dd, J = 1.9 Hz, 1H), 6.51 (dt, $^{3}J_{H\text{-}H}$ = 12.4 Hz, 1H), 5.82 (dt, $^{3}J_{H\text{-}H}$ = 12.4 Hz, 1H), 4.25 (t, J = 6.6 Hz, 2H), 2.11-2.06 (m, 2H), 1.78-1.70 (m, 2H), 1.53-1.47 (m, 7H), 1.35-1.26 (m, 7H), 0.92-0.86 (m, 15H). ^{13}C NMR (101 MHz, $CDCl_3$) δ 163.3, 148.5, 147.7, 143.8, 128.8, 119.7, 110.0, 64.6, 36.5, 29.4, 28.6, 27.5, 27.0, 26.4, 13.9, 10.4. ^{119}Sn NMR (149 MHz, $CDCl_3$) δ –60.7. API-MS (HRMS) calcd. for $C_{23}H_{40}O_3Sn$+Na [M+Na]$^+$ 507.18916; found 507.18928.

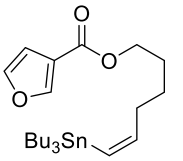

(*Z*)-3-((6-(Tributylstannyl)hex-5-en-1-yl)oxy)benzonitrile (**11**). Pale yellow liquid (89 mg, 88%). ^{1}H NMR (400 MHz, $CDCl_3$) δ 7.35 (td, J = 7.7 Hz, 1H), 7.22 (dt, J = 7.6 Hz, 1H), 7.12-7.09 (m, 2H), 6.52 (dt, $^{3}J_{H\text{-}H}$ = 12.4 Hz, 1H), 5.84 (dt, $^{3}J_{H\text{-}H}$ = 12.4 Hz, 1H), 3.97 (t, J = 6.3 Hz, 2H), 2.13-2.08 (m, 2H), 1.84-1.79 (m, 2H), 1.57-1.46 (m, 8H), 1.33-1.28 (m, 6H), 0.91-0.86 (m, 15H). ^{13}C NMR (101 MHz, $CDCl_3$) δ 159.3, 148.3, 130.4, 128.9, 124.4, 119.8, 117.4, 113.3, 68.2, 36.7, 29.3, 28.9, 27.4, 27.0, 26.3, 13.7, 10.4. ^{119}Sn NMR (149 MHz, $CDCl_3$) δ –60.8. API-MS (HRMS) calcd. for $C_{25}H_{41}NOSn$+Na [M+Na]$^+$ 514.21023; found 514.21081.

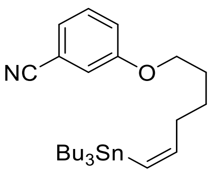

(*Z*)-(5-(Allyloxy)pent-1-en-1-yl)tributylstannane (**12**). Colorless liquid (71 mg, 82%). ^{1}H NMR (400 MHz, CDCl$_3$) δ 6.51 (dt, *J* = 12.4 Hz, 1H), 5.92 (ddt, *J* = 17.3 Hz, 1H), 5.81 (dt, $^{3}J_{H\text{-}H}$ = 12.4 Hz, 1H), 5.27 (dt, $^{3}J_{H\text{-}H}$ = 12.2 Hz, 1H), 5.18-5.15 (m, 1H), 3.97 (dt, *J* = 5.5 Hz, 2H), 3.44 (t, *J* = 6.7 Hz, 2H), 2.13-2.09 (m, 2H), 1.71-1.66 (m, 2H), 1.54-1.47 (m, 6H), 1.35-1.28 (m, 6H), 0.93-0.88 (m, 15H). ^{13}C NMR (101 MHz, CDCl$_3$) δ 148.5, 146.7, 135.2, 128.6, 116.8, 71.9, 70.0, 33.7, 30.0, 29.4, 27.5, 13.8, 10.4. ^{119}Sn NMR (149 MHz, CDCl$_3$) δ –60.6. API-MS calcd. for $C_{20}H_{40}OSn$+Na [M+Na]$^+$ 439.19933; found 439.19960.

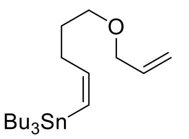

Tributyl((*Z*)-3-(cinnamyloxy)prop-1-en-1-yl)stannane (**13**). Colorless liquid (74 mg, 78%). ^{1}H NMR (400 MHz, CDCl$_3$) δ 7.41 (d, *J* = 8.5 Hz, 2H), 7.34 (t, *J* = 7.5 Hz, 2H), 7.28-7.25 (m, 1H), 6.70 (dt, $^{3}J_{H\text{-}H}$ = 13.1 Hz, 1H), 6.33 (dt, $^{3}J_{H\text{-}H}$ = 12.9 Hz, 1H), 6.14 (d, *J* = 13.0 Hz, 1H), 4.18 (d, *J* = 6.2 Hz, 2H), 4.03 (d, *J* = 6.8 Hz, 2H), 1.56-1.50 (m, 6H), 1.36-1.30 (m, 7H), 0.93-0.89 (m, 15H). ^{13}C NMR (101 MHz, CDCl$_3$) δ 144.2, 136.8, 132.8, 132.2, 128.7, 127.8, 126.6, 126.2, 73.2, 71.2, 29.3, 27.5, 27.0, 13.8, 10.8. ^{119}Sn NMR (149 MHz, CDCl$_3$) δ –59.8. API-MS calcd. for $C_{24}H_{40}OSn$+Na [M+Na]$^+$ 487.19933; found 487.19945.

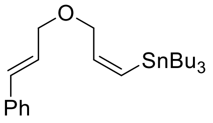

(*Z*)-11-(Tributylstannyl)undec-10-en-1-ol (**14**). Colourless liquid (79 mg, 88%). ^{1}H NMR (400 MHz, CDCl$_3$) δ 6.51 (dt, $^{3}J_{H\text{-}H}$ = 12.4 Hz, 1H), 5.77 (d, $^{3}J_{H\text{-}H}$ = 12.3 Hz, 1H), 3.64 (t, *J* = 6.7 Hz, 2H), 2.04-1.98 (m, 2H)1.67-1.63 (m, 3H), 1.58-1.48 (m, 9H), 1.37-1.28 (15H), 0.93-0.89 (m, 15H). ^{13}C NMR (101 MHz, CDCl$_3$) δ 149.5, 127.8, 63.3, 37.3, 33.0, 30.8, 29.4, 27.6, 27.5, 25.9, 17.7, 13.9, 10.1, 8.3. ^{119}Sn NMR (149 MHz, CDCl$_3$) δ -60.8. API-MS calcd. for $C_{23}H_{48}OSn$+Na [M+Na]$^+$ 483.26193; found 483.26149.

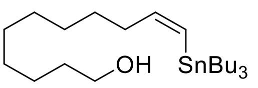

(2*E*,6*Z*)-7-(Tributylstannyl)hepta-2,6-dien-1-ol (**15**). Colorless liquid (65 mg, 79%). ^{1}H NMR (400 MHz, CDCl$_3$) δ 6.49 (dt, $^{3}J_{H\text{-}H}$ = 12.3 Hz, 1H), 5.82 (dt, $^{3}J_{H\text{-}H}$ = 12.5 Hz, 1H), 4.09 (br, 2H), 2.15- 2.09 (brm, 4H), 1.53-1.45 (m, 6H), 1.33-1.28 (m, 9H), 0.92-0.87 (m, 15H). ^{13}C NMR (101 MHz, CDCl$_3$) δ 148.1, 132.5, 129.6, 128.9, 63.9, 36.7, 32.7, 29.3, 27.4, 27.0, 13.8, 10.3. ^{119}Sn NMR (149 MHz, CDCl$_3$) δ –60.9. API-MS calcd. for $C_{19}H_{38}OSn$+Na [M+Na]$^+$ 425.18368; found 425.18403.

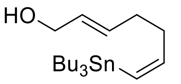

Benzyl (*Z*)-(3-(tributylstannyl)allyl)carbamate (**16a**). Colorless liquid (91 mg, 91%). ^{1}H NMR (400 MHz, CDCl$_3$) δ 7.37-7.33 (m, 5H), 6.50 (dt, $^{3}J_{H\text{-}H}$ = 13.0 Hz, 1H), 6.05 (dt, $^{3}J_{H\text{-}H}$ = 12.6 Hz, 1H), 5.12 (s, 2H), 4.70 (br, 1H), 3.78 (t, *J* = 6.0, 2H), 1.53-1.46 (m, 5H), 1.36-1.28 (m, 7H), 0.93-0.88 (m, 15H). ^{13}C NMR (101 MHz, CDCl$_3$) δ 156.2, 143.8, 136.7, 133.2, 128.7, 128.3, 66.9, 46.5, 29.3, 27.4, 27.1, 13.8, 10.5. ^{119}Sn NMR (149 MHz, CDCl$_3$) δ –58.9. API-MS (HRMS) calcd. for $C_{23}H_{39}NO_2Sn$+Na [M+Na]$^+$ 504.18949; found 504.18976.

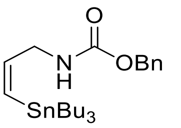

***tert*-Butyl (*Z*)-(3-(tributylstannyl)allyl)carbamate** (**16b**). Colorless liquid (83 mg, 89%). ^{1}H NMR (400 MHz, CDCl$_3$) δ 6.49 (dt, $^{3}J_{H\text{-}H}$ = 13.0 Hz, 1H), 6.01 (dt, $^{3}J_{H\text{-}H}$ = 12.6 Hz, 1H), 4.52 (br, 1H), 3.69-3.68 (br, 2H), 1.50-1.42 (m, 15H), 1.33-1.28 (m, 6H), 0.92-0.86 (m, 15H). ^{13}C NMR (101 MHz, CDCl$_3$) δ 155.7, 144.3, 132.6, 46.0, 29.2, 28.5, 27.4, 27.0, 13.7, 10.4. ^{119}Sn NMR (149 MHz, CDCl$_3$) δ –59.0. API-MS (HRMS) calcd. for $C_{20}H_{41}NO_2Sn$+Na [M+Na]$^+$ 470.20514; found 470.20547.

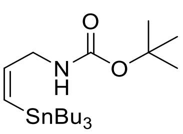

(*Z*)-2-(6-(Tributylstannyl)hex-5-en-1-yl)isoindoline-1,3-dione (**17**). Colorless liquid (94 mg, 92%). ^{1}H NMR (400 MHz, CDCl$_3$) δ 7.84-7.81 (m, 2H), 7.70-7.68 (m, 2H), 6.46 (dt, $^{3}J_{H\text{-}H}$ = 12.4 Hz, 1H), 5.78 (dt, $^{3}J_{H\text{-}H}$ = 12.4 Hz, 1H), 6.68 (t, *J* = 7.2 Hz, 2H), 2.08-2.01 (m, 2H), 1.73-1.64 (m, 3H), 1.50-1.42 (m, 6H), 1.30- 1.24 (m, 7H), 0.90-0.83 (m, 15H). ^{13}C NMR (101 MHz, CDCl$_3$) δ 168.5, 148.5, 133.9, 132.3, 128.7, 123.3, 38.1, 36.7, 29.4, 28.5, 27.4, 27.3, 27.0, 13.8, 10.4. ^{119}Sn NMR (149 MHz, CDCl$_3$) δ –60.8. API-MS (HRMS) calcd. for $C_{26}H_{41}NO_2Sn$+Na [M+Na]$^+$ 542.20514; found 542.20513.

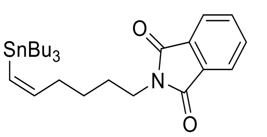

(*Z*)-7-((6-(Tributylstannyl)hex-5-en-1-yl)oxy)-2*H*-chromen-2-one (**18**). Colorless liquid (98 mg, 92%). ^{1}H NMR (400 MHz, $CDCl_3$) δ 7.62 (d, *J* = 9.49 Hz, 1H), 7.35 (d, *J* = 8.52 Hz, 1H), 6.83-6.78 (m, 2H), 6.52 (dt, $^{3}J_{H\text{-}H}$ = 12.4 Hz, 1H), 6.23 (d, *J* = 9.4 Hz, 1H), 5.83 (dt, $^{3}J_{H\text{-}H}$ = 12.4 Hz, 1H), 4.01 (t, *J* = 6.4 Hz, 2H), 2.13-2.08 (m, 2H), 1.86-1.79 (m, 2H), 1.60-1.45 (m, 7H), 1.32-1.27 (m, 7H), 0.90-0.86 (m, 15H). ^{13}C NMR (101 MHz, $CDCl_3$) δ 162.5, 161.3, 156.0, 148.4, 143.5, 128.9, 113.0, 112.5, 101.4, 68.5, 36.7, 29.3, 28.8, 27.4, 27.0, 26.3, 13.8, 10.4. ^{119}Sn NMR (149 MHz, $CDCl_3$) δ –60.7. API-MS (HRMS) calcd. for $C_{27}H_{42}O_3Sn$+Na [M+Na]$^+$ 557.20481; found 557.20494.

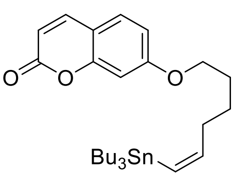

(*Z*)-5-(Tributylstannyl)pent-4-en-1-yl (*S*)-2-(6-methoxynaphthalen-2-yl)propanoate (**19**). Colorless liquid (101 mg, 88%). ^{1}H NMR (400 MHz, $CDCl_3$) δ 7.70 (d, *J* = 8.60 Hz, 1H), 7.67 (s, 1H), 7.41 (dd, *J* = 8.4, 1H), 7.16-7.11 (m, 2H), 6.44 (dt, $^{3}J_{H\text{-}H}$ = 12.4 Hz, 1H), 5.81 (dt, $^{3}J_{H\text{-}H}$ = 12.4 Hz, 1H), 4.16-4.03 (m, 2H), 3.91 (s, 3H), 3.86 (d, *J* =7.1 Hz, 1H), 2.07-2.00 (m, 2H), 1.71-1.66 (m, 3H), 1.58 (d, *J* = 7.2 Hz, 2H), 1.52-1.45 (m, 6H), 1.35-1.28 (m, 7H), 0.92-0.87 (m, 15H). ^{13}C NMR (101 MHz, $CDCl_3$) δ 174.8, 157.8, 147.6, 135.9, 133.8, 129.4, 129.2, 129.1, 127.2, 126.3, 126.0, 119.0, 105.7, 64.6, 55.4, 45.6, 33.4, 29.3, 27.4, 18.8, 13.8, 10.3. ^{119}Sn NMR (149 MHz, $CDCl_3$) δ –60.4. API-MS (HRMS) calcd. for $C_{31}H_{48}O_3Sn$+Na [M+Na]$^+$ 611.25176; found 611.25188.

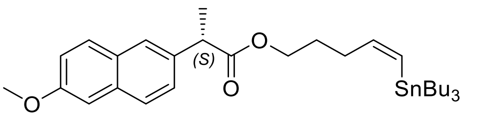

(*Z*)-3-Bromo-5-(4-(tributylstannyl)but-3-en-1-yl)pyridine (**20**). Colorless liquid (85 mg, 88%). ^{1}H NMR (400 MHz, $CDCl_3$) δ 8.51 (d, *J* = 2.2 Hz, 1H), 8.36 (d, *J* = 1.9 Hz, 1H), 7.65 (t, *J* = 2.1 Hz, 1H), 6.49 (dt, $^{3}J_{H\text{-}H}$ = 12.4 Hz, 1H), 5.90 (dt, $^{3}J_{H\text{-}H}$ = 12.5 Hz, 1H), 2.68 (dd, *J* = 8.6 Hz, 2H), 2.36-2.31 (m, 2H), 1.49-1.42 (m, 6H), 1.32-1.28 (m, 6H), 0.89-0.86 (m, 15H). ^{13}C NMR (101 MHz, $CDCl_3$) δ 148.7, 148.2, 146.5, 138.9, 138.6, 130.6, 38.2, 33.0, 29.4, 27.6, 13.8, 10.4. ^{119}Sn NMR (149 MHz, $CDCl_3$) δ –60.7. API-MS (HRMS) calcd. for $C_{21}H_{36}BrSn$+Na [M+Na]$^+$ 502.111840; found 502.112529.

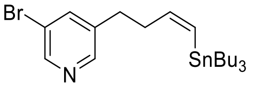

(8*R*,9*S*,13*S*,14*S*)-13-methyl-3-(((*Z*)-6-(tributylstannyl)hex-5-en-1-yl)oxy) 6,7,8,9,11,12,13,14,15,16-decahydro-17*H*-cyclopenta[a]phenanthren-17-one (**21**). Colorless liquid (115 mg, 90%). ^{1}H NMR (400 MHz, $CDCl_3$) δ 7.19 (d, *J* = 8.6 Hz, 1H), 6.71 (dd, *J* = 8.6 Hz, 1H), 6.64 (m, 1H), 6.53 (dt, $^{3}J_{H\text{-}H}$ = 12.4 Hz, 1H), 5.82 (dt, $^{3}J_{H\text{-}H}$ = 12.3 Hz, 1H), 3.94 (t, *J* = 6.5 Hz, 2H), 2.90 (br, 2H), 2.54-2.47 (m, 1H), 2.29-2.22 (m, 1H), 2.17-2.07 (m, 4H), 2.00-1.94 (m, 2H), 1.83-1.76 (m, 2H), 1.65-1.47 (m, 15H), 1.35-1.28 (m, 8H), 0.91-0.88 (m, 16H). ^{13}C NMR (101 MHz, $CDCl_3$) δ 221.0, 157.2, 148.8, 137.8, 132.0, 128.5, 126.6, 114.7, 112.2, 67.8, 50.6, 48.1, 44.1, 38.5, 36.9, 36.0, 31.7, 29.8, 29.3, 29.2, 27.5, 27.0, 26.7, 26.5, 26.0, 21.7, 14.0, 13.9, 10.4. ^{119}Sn NMR (149 MHz, $CDCl_3$) δ –60.7. API-MS (HRMS) calcd. for $C_{36}H_{58}O_2Sn$+Na [M+Na]$^+$ 665.33510; found 665.33556.

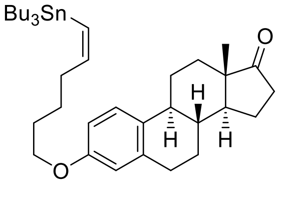

(*Z*)-Tributyl(3-methylbut-1-en-1-yl)stannane (major component of isomer mixture, rr = 88:12). Colorless liquid (54 mg, 75%). ^{1}H NMR (400 MHz, $CDCl_3$) δ 6.30 (dd, $^{3}J_{H\text{-}H}$ = 12.2 Hz, 1H), 5.64 (d, $^{3}J_{H\text{-}H}$ = 12.2 Hz, 1H), 2.10-2.02 (m, 1H), 1.52-1.47 (m, 6H), 1.35-1.30 (m, 5H), 0.99 (d, J = 6.6 Hz, 8H), 0.92-0.87 (m, 14H). ^{13}C NMR (101 MHz, $CDCl_3$) δ 156.4, 124.7, 122.0, 36.9, 29.4, 27.5, 23.2, 13.8, 10.5. ^{119}Sn NMR (149 MHz, $CDCl_3$) δ –45.2 (*minor*), –59.0 (*major*).

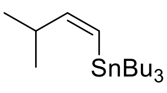

major isomer

(*Z*)-Tributyl(2-cyclopentylvinyl)stannane (major component of isomer mixture, rr = 90:10) (##). Colorless liquid (82 mg, 81%). ^{1}H NMR (400 MHz, $CDCl_3$) δ 6.39 (dd, $^{3}J_{H\text{-}H}$ = 12.2 Hz, 1H), 5.68 (d, $^{3}J_{H\text{-}H}$ = 12.2 Hz, 1H), 2.25-2.17 (brm, 1H), 1.71-1.63 (m, 3H), 1.58-1.47 (m, 7H), 1.36-1.29 (m, 9 H), 0.93-0.88 (m, 16H). ^{13}C NMR (101

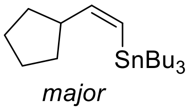

major isomer

MHz, $CDCl_3$) δ 154.2, 125.7, 48.5, 34.0, 29.4, 27.5, 25.8, 13.9, 10.5. ^{119}Sn NMR (149 MHz, $CDCl_3$) δ –45.4 (*minor*), –58.6 (*major*).

(*Z*)-1-Phenyl-3-(tributylstannyl)allyl acetate (**25**). Colorless liquid (81 mg, 85%). ^{1}H NMR (400 MHz, $CDCl_3$) δ 7.39-7.37 (m, 4H), 7.33-7.30 (m, 1H), 6.75 (dt, $^{3}J_{H\text{-}H}$ = 12.9 Hz, 1H), 6.22 (dt, $^{3}J_{H\text{-}H}$ = 12.9 Hz, 1H), 6.06 (d, J = 8.7 Hz, 1H), 2.11 (s, 3H), 1.56-1.49 (m, 5H), 1.37-1.29 (m, 7H), 1.04-1.00 (m, 5H), 0.90 (t, J = 7.3 Hz, 10H). ^{13}C NMR (101 MHz, $CDCl_3$) δ 169.9, 145.3, 140.1, 134.5, 128.7, 128.1, 127.0, 79.0, 29.3, 27.4, 27.1, 21.5, 13.7, 10.6. ^{119}Sn NMR (149 MHz, $CDCl_3$) δ –58.4. API-MS (HRMS) calcd. for $C_{23}H_{38}O_2Sn$+Na [M+Na]$^+$ 489.17860; found 489.17899.

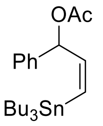

1-Phenyl-2-(tributylstannyl)prop-2-en-1-ol. (**24**) Using catalyst **Fe-9b**; pale yellow liquid (66 mg, 78%, mixture of regioisomers ca. 7:93). ^{1}H NMR (400 MHz, $CDCl_3$) δ 7.33-7.31(brm, 4H), 7.29-7.27 (br, 1H), 5.92 (d, $^{2}J_{H\text{-}H}$ = 2.2 Hz, 1H), 5.34 (d, $^{2}J_{H\text{-}H}$ = 2.2 Hz, 1H), 2.24 (s, 1H), 1.93 (br, 1H), 1.39-1.31 (br, 7H), 1.26-1.21 (m, 6H), 0.91-0.83 (m, 14H). ^{13}C NMR (101 MHz, $CDCl_3$) δ 158.2, 143.1, 128.5, 127.5, 126.6, 124.5, 80.7, 29.4, 28.0, 13.8, 10.1. ^{119}Sn NMR (149 MHz, $CDCl_3$) δ –43.8 (*major*), –59.0 (*minor regioisomer*). API-MS (HRMS) calcd. for $C_{21}H_{36}OSn$+Na [M+Na]$^+$ 447.17462; found 489.17453.

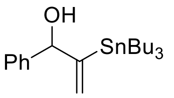

1-Phenyl-5-(tributylstannyl)hex-5-en-3-ol (**27**). Colorless liquid (75 mg, 84%). ^{1}H NMR (400 MHz, $CDCl_3$) δ 7.32-7.28 (m, 2H), 7.25-7.18 (m, 3H), 5.82 (d, $^{2}J_{H\text{-}H}$ = 2.7 Hz, 1H), 5.33 (d, $^{2}J_{H\text{-}H}$ = 2.8 Hz, 1H), 3.63-3.56 (m, 1H), 2.88-2.81 (m, 1H), 2.77-2.69 (m, 1H), 2.55 (d, J = 14.3 Hz, 1H), 2.36-2.30 (m, 3H), 1.87-1.75 (m, 6H), 1.56-1.46 (m, 7H), 0.97-0.83 (m, 15H). ^{13}C NMR (101 MHz, $CDCl_3$) δ 152.4, 142.2, 129.0, 128.6, 128.5, 125.9, 69.2, 50.1, 38.8, 32.2, 29.2, 27.5, 13.8, 9.8. ^{119}Sn NMR (149 MHz, $CDCl_3$) δ –44.7. API-MS (HRMS) (HRMS) calcd. for $C_{24}H_{42}OSn$+Na [M+Na]$^+$ 489.19424; found 489.19425.

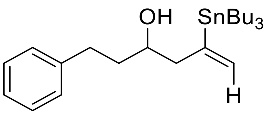

(*Z*)-Tributyl(4-methoxy-6-phenylhex-1-en-1-yl)stannane (**28a**). Colorless liquid (78 mg, 80%). ^{1}H NMR (400 MHz, $CDCl_3$) δ 7.30-7.28 (m, 2H), 7.20- 7.18 (m, 2H), 6.52 (dt, $^{3}J_{H\text{-}H}$ = 12.6 Hz, 1H), 5.92 (dt, $^{3}J_{H\text{-}H}$ = 12.6 Hz, 1H), 3.38 (s, 3H), 3.27-3.21 (m, 1H), 2.80-2.73 (m, 1H), 2.68-2.60 (m, 1H), 2.40-2.32 (m, 1H), 2.25-2.19 (m, 1H), 1.82-1.77 (m, 2H), 1.52-1.46 (m, 7H), 1.34-1.27 (m, 6H), 0.93-0.86 (m, 15H). ^{13}C NMR (101 MHz, $CDCl_3$) δ 144.8, 142.5, 130.8, 128.5, 125.8, 80.2, 56.9, 40.5, 35.8, 31.8, 29.2, 27.2, 13.8, 10.4. ^{119}Sn NMR (149 MHz, $CDCl_3$) δ –61.8. API-MS (HRMS) calcd. for $C_{24}H_{44}OSn$+Na [M+Na]$^+$ 503.2255; found 503.22538

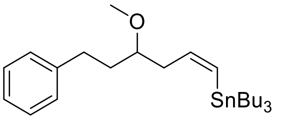

(*Z*)-1-Phenyl-6-(tributylstannyl)hex-5-en-3-yl acetate (**28b**). Colorless liquid (88 mg, 85%). ^{1}H NMR (400 MHz, $CDCl_3$) δ 7.30-7.27 (m, 2H), 7.20-7.15 (m, 3H), 6.45 (dt, $^{3}J_{H\text{-}H}$ = 12.5 Hz, 1H), 5.93 (dt, $^{3}J_{H\text{-}H}$ = 12.6 Hz, 1H), 5.00 (dt, J = 7.9 Hz, 1H), 2.34-2.31 (m, 2H), 2.03-1.91 (m, 2H), 2.01 (s, 3H) 1.83-1.68 (m, 2H), 1.61- 1.52 (m, 4H), 1.46-1.34 (m, 4H), 1.29 (br, 4H), 0.90-0.86 (m, 15H). ^{13}C NMR (101 MHz, $CDCl_3$) δ 170.9, 143.6, 141.6, 131.8, 128.6, 128.4, 126.1, 73.2, 41.7, 35.8, 32.0, 29.2, 27.5, 27.1, 13.8, 10.4. ^{119}Sn NMR (149 MHz, $CDCl_3$) δ –61.3. API-MS (HRMS) calcd. for $C_{26}H_{44}O_2Sn$+Na [M+Na]$^+$ 531.2255; found 531.22538.

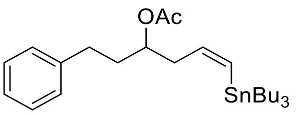

(*Z*)-*tert*-Butyldimethyl((1-phenyl-6-(tributylstannyl)hex-5-en-3-yl)oxy)silane (**28c**). Pale yellow liquid (92 mg, 79%). ^{1}H NMR (400 MHz, $CDCl_3$) δ 7.33-7.28 (m, 3H), 7.21 (d, J = 7.2 Hz, 2H), 6.58 (dt, $^{3}J_{H\text{-}H}$ = 12.77 Hz, 1H), 5.93 (dt, $^{3}J_{H\text{-}H}$ = 12.7 Hz, 1H), 3.88-3.82 (m, 1H), 2.81-2.73 (m, 1H), 2.68-2.60 (m, 2H), 2.36-2.27 (m, 8H), 1.86-1.76 (m, 7H), 0.97 (s, 9H), 0.96-0.90 (m, 15H), 0.11 (d, J = 3.0 Hz, 6H). ^{13}C NMR (101 MHz, $CDCl_3$) δ 145.6, 142.8, 130.2, 128.5, 125.8, 72.0, 44.7,

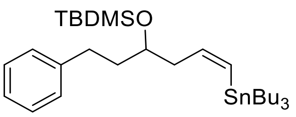

39.3, 31.9, 29.5, 27.6, 27.1, 26.1, 18.3, 13.8, 10.4, –4.0, –4.3. ^{119}Sn NMR (149 MHz, $CDCl_3$) δ –61.9. API-MS (HRMS) calcd. for $C_{30}H_{56}OSiSn$+Na [M+Na]$^+$ 603.30146; found 603.30117.

1-Phenyl-5-(tributylstannyl)hex-5-en-3-amine (**30**). Colorless liquid (74 mg, 85%). ^{1}H NMR (600 MHz, $CDCl_3$) δ 7.29-7.26 (m, 2H), 7.20-7.16 (m, 3H), 5.74, 5.74 (d, $^2J_{H\text{-}H}$ = 2.9 Hz, 1H), 5.24 (d, $^2J_{H\text{-}H}$ = 2.6 Hz, 1H), 2.79-2.71 (m, 2H), 2.67-2.62 (m, 1H), 2.51-2.48 (m, 3H), 1.66-1.61 (m, 5H), 1.51-1.43 (m, 9H), 0.90-0.84 (m, 16H). ^{13}C NMR (151 MHz, $CDCl_3$) δ 153.3, 142.4, 128.5, 128.5, 127.9, 125.9, 77.4, 77.2, 76.9, 50.8, 49.8, 39.7, 32.8, 29.3, 27.5, 13.8, 9.8. ^{119}Sn NMR (149 MHz, $CDCl_3$) δ –46.0. API-MS (HRMS) calcd. for $C_{24}H_{43}NSn$ (M+H$^+$) 466.24907; found 466.24902.

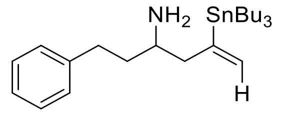

(*Z*)-N-(1-Phenyl-6-(tributylstannyl)hex-5-en-3-yl)acetamide (**31a**). Colorless liquid (92 mg, 89%). ^{1}H NMR (400 MHz, $CDCl_3$) δ 7.30-7.27 (m, 2H), 7.19-7.17 (m, 3H), 6.48 (dt, $^3J_{H\text{-}H}$ = 12.5 Hz, 1H), 5.96 (dd, $^3J_{H\text{-}H}$ = 12.5 Hz, 1H), 5.16 (d, J = 9.1 Hz, 1H), 4.15 (br, 1H), 2.68-2.66 (m, 2H), 2.64 (br, 2H), 2.27-2.22 (m, 3H), 1.94 (s, 3H), 1.52-1.47 (m, 5H), 1.35-1.29 (m, 6H), 0.93-0.88 (15H). ^{13}C NMR (101 MHz, $CDCl_3$) δ 169.6, 144.2, 141.9, 132.1, 128.6, 128.4, 126.1, 49.0, 42.1, 36.7, 32.7, 30.8, 29.2, 27.5, 23.7, 17.7, 13.8, 10.5. ^{119}Sn NMR (149 MHz, $CDCl_3$) δ –61.8. API-MS (HRMS) calcd. for $C_{26}H_{45}NOSn$+Na [M+Na]$^+$ 530.24153; found 530.24147.

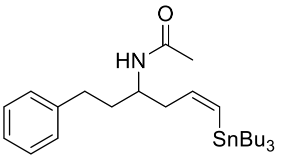

Benzyl (*Z*)-(1-phenyl-6-(tributylstannyl)hex-5-en-3-yl)carbamate (**31b**). Colorless liquid (108 mg, 92%). ^{1}H NMR (400 MHz, $CDCl_3$) δ 7.79-7.77 (m, 2H), 7.69-7.67 (m, 2H), 7.18-7.05 (m, 5H), 6.39 (dt, $^3J_{H\text{-}H}$ = 12.6 Hz, 1H), 5.86 (dt, $^3J_{H\text{-}H}$ = 12.6 Hz, 1H), 4.41-4.33 (m, 1H), 2.79-2.71 (m, 1H), 2.61-2.52 (m, 2H), 1.67-1.58 (m, 5H), 1.48-1.43 (m, 3H), 1.35-1.25 (m, 11H), 0.92-0.86 (m, 15H). ^{13}C NMR (101 MHz, $CDCl_3$) δ 168.6, 144.4, 141.2, 133.9, 132.0, 128.5, 125.9, 123.2, 52.1, 40.0, 33.3, 29.2, 28.0, 27.5, 27.2, 16.5, 13.7, 10.4. ^{119}Sn NMR (149 MHz, $CDCl_3$) δ –60.6. API-MS (HRMS) calcd. for $C_{32}H_{49}NO_2Sn$+Na [M+Na]$^+$ 622.26775; found 622.26736.

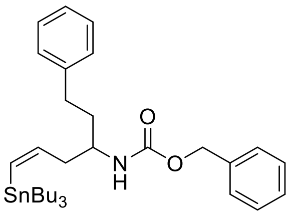

(*Z*)-2-(1-Phenyl-6-(tributylstannyl)hex-5-en-3-yl)isoindoline-1,3-dione (**31c**). Colorless liquid (102 mg, 88%). ^{1}H NMR (400 MHz, $CDCl_3$) δ 7.56-7.37 (m, 4H), 7.36-7.15 (m, 3H), 6.49 (dt, $^3J_{H\text{-}H}$ = 13.4 Hz, 1H), 5.95 (dt, $^3J_{H\text{-}H}$ = 12.6 Hz, 1H), 5.11-5.08 (m, 2H), 4.63-4.57 (m, 1H), 3.82 (br, 1H), 2.74-2.60 (m, 2H), 2.27-2.24 (m, 2H), 1.77-1.60 (m, 2H), 1.48-1.43 (br, 5H), 1.35-1.26 (m, 7H), 0.94-0.84 (m, 14H). ^{13}C NMR (101 MHz, $CDCl_3$) δ 177.0, 156.0, 144.0, 141.8, 136.7, 132.1, 128.5, 128.1, 126.0, 66.7, 60.4, 50.6, 42.6, 37.0, 32.5, 29.3, 28.0, 27.3, 27.1, 16.5, 13.6, 10.3. ^{119}Sn NMR (149 MHz, $CDCl_3$) δ –61.8. API-MS (HRMS) calcd. for $C_{32}H_{45}NO_2Sn$+Na [M+Na]$^+$ 618.23645; found 618.23538.

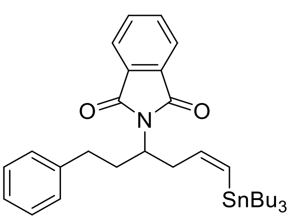

1-(4-Methoxyphenyl)-3-(tributylstannyl)but-3-en-1-ol (**32a**). Colorless liquid (72 mg, 75%). ^{1}H NMR (400 MHz, $CDCl_3$) δ 7.29 (d, J = 8.6 Hz, 2H), 6.89 (d, J = 8.7 Hz, 2H), 5.85 (d, $^2J_{H\text{-}H}$ = 2.9 Hz, 1H), 5.36 (d, $^2J_{H\text{-}H}$ = 2.9 Hz, 1H), 4.61 (dd, J = 9.8 Hz, 1H), 3.80 (s, 3H), 2.73-2.68 (m, 1H), 2.58-2.53 (m, 1H), 1.55-1.49 (m, 5H), 1.38-1.30 (m, 8H), 0.96-0.90 (m, 15H). ^{13}C NMR (101 MHz, $CDCl_3$) δ 159.1, 152.6, 136.4, 129.4, 127.1, 113.9, 72.3, 55.3, 52.0, 29.2, 27.5, 13.8, 9.9. ^{119}Sn NMR (149 MHz, $CDCl_3$) δ –44.4. API-MS (HRMS) calcd. for $C_{23}H_{40}O_2Sn$+Na [M+Na]$^+$ 491.19424; found 491.19425.

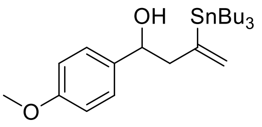

1-([1,1'-Biphenyl]-4-yl)-3-(tributylstannyl)but-3-en-1-ol (**32b**). Colorless liquid (80 mg, 75%). ^{1}H NMR (400 MHz, $CDCl_3$) δ 7.62-7.59 (m, 5 H), 7.47-7.43 (m, 3H), 7.37-7.34 (m, 1H), 5.91 (d, $^2J_{H\text{-}H}$ = 2.9 Hz, 1H), 5.41 (d, $^2J_{H\text{-}H}$ = 2.3 Hz, 1H), 4.79-4.70 (m, 1H), 2.77 (d, J = 3.3 Hz, 1H), 2.63-2.57 (m, 1H), 2.21-2.19 (m, 1H), 1.67-1.62 (2 H), 1.58-1.49 (brm, 4 H), 1.40-1.30 (brm, 5H), 1.00-0.89

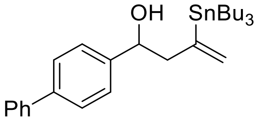

(m, 16 H). ^{13}C NMR (101 MHz, $CDCl_3$) δ 152.5, 143.2, 141.1, 140.5, 129.7, 128.9, 127.3, 127.2, 126.3, 52.2, 29.3, 27.6, 13.8, 10.0. ^{119}Sn NMR (149 MHz, $CDCl_3$) δ –44.0. API-MS (HRMS) calcd. for $C_{28}H_{42}OSn$+Na $[M+Na]^+$ 537.19446; found 537.19465.

1-(4-Chlorophenyl)-3-(tributylstannyl)but-3-en-1-ol (**32c**). Pale yellow liquid (75 mg, 81%).

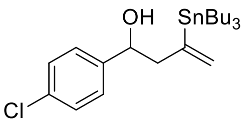

^{1}H NMR (400 MHz, $CDCl_3$) δ 7.33-7.28 (m, 4H), 5.84 (d, $^2J_{H\text{-}H}$ = 2.8 Hz, 1H), 5.38 (d, $^2J_{H\text{-}H}$ = 2.9 Hz, 1H), 4.62 (dd, J = 10.0 Hz, 1H), 4.61-4.60 (m, 1H), 2.71-2.68 (m, 1H), 2.52-2.44 (m, 1H), 1.59-1.49 (m, 4H), 1.37-1.30 (m, 7H), 0.96-0.90 (m, 15H). ^{13}C NMR (101 MHz, $CDCl_3$) δ 152.1, 142.7, 133.1, 129.9, 128.6, 127.3, 72.0, 52.1, 29.2, 27.7, 14.0, 10.1. ^{119}Sn NMR (149 MHz, $CDCl_3$) δ –44.0. API-MS (HRMS) calcd. for $C_{22}H_{36}ClOSn$ $[M\text{-}H]^-$ 471.14821; found 471.14858.

4-(1-Hydroxy-3-(tributylstannyl)but-3-en-1-yl)benzonitrile (**32d**). Colorless liquid (66 mg, 72%).

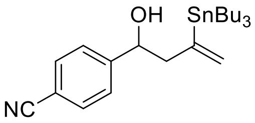

^{1}H NMR (400 MHz, $CDCl_3$) δ 7.64 (d, J = 8.4 Hz, 2H), 7.48 (d, J = 8.1 Hz, 2H), 5.86 (d, $^2J_{H\text{-}H}$ = 2.1 Hz, 1H), 5.41 (d, $^2J_{H\text{-}H}$ = 2.4 Hz, 1H), 4.68 (d, J = 10.1 Hz, 1H), 2.72 (dd, J = 13.7 Hz, 1H), 2.48-2.40 (m, 1H), 2.30 (brs, 1H), 1.66-1.62 (m, 2H), 1.54-1.49 (m, 4H), 1.38-1.30 (m, 5H), 0.94-0.88 (m, 15H). ^{13}C NMR (101 MHz, $CDCl_3$) δ 151.7, 149.5, 132.4, 130.5, 126.5, 119.0, 111.2, 71.9, 52.2, 29.2, 27.5, 13.9, 10.0. ^{119}Sn NMR (149 MHz, $CDCl_3$) δ –43.5. API-MS (HRMS) calcd. for $C_{23}H_{36}NOSn$ $[M\text{-}H]^-$ 462.18241; found 462.18313.

1-(Naphthalen-1-yl)-3-(tributylstannyl)but-3-en-1-ol (**33**). Pale yellow liquid (96 mg, 68%).

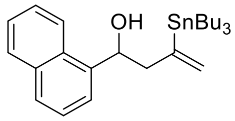

^{1}H NMR (400 MHz, $CDCl_3$) δ 8.12 (d, J = 7.9 Hz, 1H), 7.89 (d, J = 8.0 Hz, 1H), 7.79 (d, J = 8.2 Hz, 1H), 7.72 (d, J = 7.2 Hz, 1H), 7.54-7.48 (m, 3H), 5.98 (d, J = 1.3 Hz, 1H), 5.55-5.43 (brm, 2H), 3.02-2.97 (m, 1H), 2.75-2.66 (m, 1H), 2.36 (br, 1H), 1.70-1.50 (m, 5H), 1.39-1.28 (m, 7H), 1.02-0.86 (m, 15H). ^{13}C NMR (101 MHz, $CDCl_3$) δ 152.7, 150.9, 139.8, 133.9, 130.5, 129.5, 129.1, 128.0, 126.0, 125.7, 125.6, 123.1, 123.1, 69.6, 50.8, 29.4, 27.6, 13.8, 10.1. ^{119}Sn NMR (149 MHz, $CDCl_3$) δ –44.2. API-MS (HRMS) calcd. for $C_{26}H_{40}O_2Sn$+Na $[M+Na]^+$ 511.19933; found 511.199.

2-Methyl-5-(tributylstannyl)hex-5-en-3-ol (**34**). Colorless liquid (67 mg, 79%).

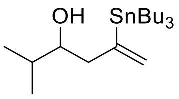

^{1}H NMR (400 MHz, $CDCl_3$) δ 5.80 (d, $^2J_{H\text{-}H}$ = 2.8 Hz, 1H), 5.31 (d, $^2J_{H\text{-}H}$ = 2.9 Hz, 1H), 3.32-3.27 (m, 1H), 2.56 (d, J = 13.2 Hz, 1H), 2.22-2.17 (m, 1H), 1.67-1.62 (m, 4H), 1.53-1.46 (m, 8H), 1.36-1.29 (m, 8H), 0.98-0.90 (m, 15H). ^{13}C NMR (101 MHz, $CDCl_3$) δ 153.3, 129.1, 74.6, 46.5, 33.3, 29.3, 27.6, 18.7, 17.7, 13.9, 9.9. ^{119}Sn NMR (149 MHz, $CDCl_3$) δ –44.9. API-MS (HRMS) calcd. for $C_{19}H_{40}OSn$+Na $(M+Na)^+$ 427.19933; found 427.19890.

(1r,3r,5r,7r)-2-(2-(Tributylstannyl)allyl)adamantan-2-ol (**35**). Colorless liquid (75 mg, 75%).

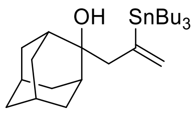

^{1}H NMR (400 MHz, $CDCl_3$) δ 5.77 (d, $^2J_{H\text{-}H}$ = 2.9 Hz, 1H), 5.31 (d, $^2J_{H\text{-}H}$ = 3.2 Hz, 1H), 2.61 (s, 2H), 2.11 (d, J = 16.0 Hz, 2H), 1.86 (d, J = 16.0Hz, 2H), 1.69-1.65 (m, 10H), 1.50-1.45 (m, 6H), 1.35-1.28 (m, 7H), 0.90-0.86 (m, 15H). ^{13}C NMR (101 MHz, $CDCl_3$) δ 151.8, 129.1, 75.3, 49.5, 38.5, 37.1, 34.4, 33.3, 29.4, 27.7, 13.9, 11.0. ^{19}Sn NMR (149 MHz, $CDCl_3$) δ –46.0. API-MS (HRMS) calcd. for $C_{25}H_{46}OSn$+Na $(M+Na^+)$ 505.24628; found 505.24591.

1-(2-(Tributylstannyl)allyl)cyclohexan-1-ol (**36**). Colorless liquid (58 mg, 69%).

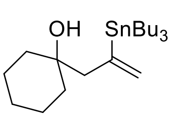

^{1}H NMR (400 MHz, $CDCl_3$) δ 5.73 (d, $^2J_{H\text{-}H}$ = 3.2 Hz, 1H), 5.30 (d, $^2J_{H\text{-}H}$ = 3.2 Hz, 1H), 2.38 (s, 2H), 1.62-1.45 (m, 14H), 1.36-1.28 (m, 8H), 0.92-0.86 (m, 15H). ^{13}C NMR (101 MHz, $CDCl_3$) δ 151.6, 129.6, 71.5, 38.1, 29.3, 27.6, 25.9, 22.3, 13.8, 10.6. ^{119}Sn NMR (149 MHz, $CDCl_3$) δ –46.1. API-MS (HRMS) calcd. for $C_{21}H_{41}OSn$ $(M\text{-}H^-)$ 429.2185; found 429.21848

2-Phenyl-4-(tributylstannyl)pent-4-en-2-ol (**37**). Colorless liquid (75 mg, 81%). ^{1}H NMR (400 MHz, CDCl$_3$) δ 7.45 (d, *J* = 8.4 Hz, 2H), 7.34 (t, *J* = 8.2 Hz, 2H), 7.25-7.20 (m, 1H) 5.74 (d, $^2J_{H-H}$ = 2.7 Hz, 1H), 5.34 (d, $^2J_{H-H}$ = 3.0 Hz, 1H), 2.85 (d, *J* = 13.3 Hz, 1H), 2.62 (d, *J* = 8.1 Hz, 1H), 1.47 (s, 3H), 1.42-1.40 (m, 6H), 1.33-1.28 (m, 6H), 0.90 (t, *J* = 7.3 Hz, 11H), 0.82-0.77 (m, 5H). ^{13}C NMR (101 MHz, CDCl$_3$) δ 151.5, 148.8, 131.0, 128.2, 126.7, 124.9, 74.1, 54.8, 30.4, 29.3, 28.0, 27.6, 27.0, 13.9, 13.8, 10.4. ^{119}Sn NMR (149 MHz, CDCl$_3$) δ –43.5. API-MS (HRMS) calcd. for $C_{23}H_{40}OSn$+Na (M+Na)$^+$ 475.19933; found 475.19916

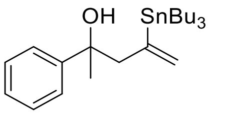

Stannylative Cyclization Reactions and Control Experiments

Diethyl (*E*)-3-methyl-4-((tributylstannyl)methylene)cyclopentane-1,1-dicarboxylate (**39a**). Colorless liquid (80 mg, 75%). ^{1}H NMR (400 MHz, CDCl$_3$) δ 5.61 (q, *J* = 2.2 Hz, 1H), 4.22-4.15 (m, 5H), 1.52-1.45 (m, 7H), 1.33-1.28 (m, 8H), 1.26-1.23 (m, 7H), 1.09 (d, *J* = 6.2 Hz, 3H), 0.92-0.87 (m, 15H). ^{13}C NMR (151 MHz, CDCl$_3$) δ 172.1, 172.1, 162.8, 118.2, 118.2, 116.9, 115.7, 115.6, 77.4, 77.2, 77.0, 61.5, 61.2, 58.4, 42.6, 42.3, 39.8, 29.3, 29.2, 27.6, 27.4, 27.2, 27.1, 18.6, 14.2, 13.8, 9.9. ^{119}Sn NMR (149 MHz, CDCl$_3$) δ –55.7. API-MS calcd. for $C_{25}H_{46}O_4Sn$+Na [M+Na]$^+$ 553.18767; found 553.18758.

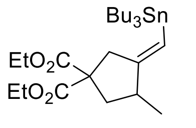

(*Z*)-3-Methyl-1-tosyl-4-((tributylstannyl)methylene)pyrrolidine (**39b**). Colorless liquid (85 mg, 78%). ^{1}H NMR (400 MHz, CDCl$_3$) δ 7.70 (d, *J* = 8.3 Hz, 2H), 7.32 (d, *J* = 7.9 Hz, 2H), 5.67 (q, *J* = 2.1 Hz, 1H), 3.87 (dd, *J* = 14.0 Hz, 1H), 3.62-3.59 (m, 2H), 2.70-2.63 (m, 2H), 2.44 (s, 3H), 1.45-1.40 (m, 6H) 1.31-1.24 (m, 6H), 1.03 (d, *J* = 6.09 Hz, 3H), 0.89-0.85 (m, 15H). ^{13}C NMR (101 MHz, CDCl$_3$) δ 158.2, 143.7, 132.8, 129.8, 127.9, 118.2, 55.4, 53.6, 40.1, 29.1, 27.4, 27.0, 21.6, 16.4, 13.8, 9.8. ^{119}Sn NMR (149 MHz, CDCl$_3$) δ –53.7. API-MS calcd. for $C_{25}H_{43}NO_2SSn$+Na [M+Na]$^+$ 566.19277; found 566.19256.

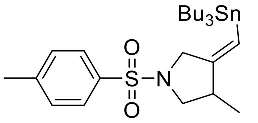

(*Z*)-1-((4-Methoxyphenyl)sulfonyl)-3-methyl-4-((tributylstannyl)methylene)pyrrolidine (**39c**). Colorless liquid (86 mg, 76%). ^{1}H NMR (400 MHz, CDCl$_3$) δ 7.75 (d, *J* = 8.9 Hz, 2H), 6.99 (d, *J* = 8.9 Hz, 2H), 5.67 (q, *J* = 2.2 Hz, 1H), 3.86 (s, 3H), 3.61-3.58 (m, 2H), 2.66-2.63 (m, 2H), 1.45-1.40 (m, 9H), 1.31-1.25 (m, 5H), 1.03 (d, *J* = 6.5 Hz, 3H), 0.88-0.86 (m, 14H). ^{13}C NMR (101 MHz, CDCl$_3$) δ 163.1, 158.3, 130.0, 127.5, 118.2, 114.3, 55.7, 55.4, 53.6, 40.1, 29.3, 27.4, 27.0, 16.4, 13.8, 9.9. ^{119}Sn NMR (149 MHz, CDCl$_3$) δ –53.7. API-MS calcd. for $C_{25}H_{43}NO_3SSn$+Na [M+Na]$^+$ 580.18778; found 580.18829.

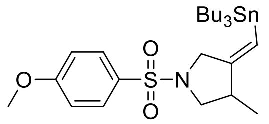

(*Z*)-3-Methyl-1-(naphthalen-2-ylsulfonyl)-4-((tributylstannyl)methylene)pyrrolidine (**39d**). Pale yellow liquid (95 mg, 82%). ^{1}H NMR (400 MHz, CDCl$_3$) δ 8.39 (s, 1H), 8.00-7.96 (m, 2H), 7.93-7.91 (m, 1H), 7.83-7.81 (m, 1H), 5.67-5.66 (m, 2H), 5.65 (q, *J* = 2.2 Hz, 1H), 3.96 (dd, *J* = 13.9 Hz, 1H), 3.71-3.66 (m, 2H), 2.76-2.64 (m, 7H), 1.41-1.37 (m, 6H), 1.02 (d, *J* = 6.5 Hz, 3H), 0.88-0.82 (m, 16H). ^{13}C NMR (101 MHz, CDCl$_3$) δ 158.0, 135.0, 133.0, 132.3, 129.4, 129.3, 129.1, 128.9, 128.0, 127.7, 123.2, 118.4, 55.4, 53.7, 40.2, 29.2, 27.4, 27.0, 16.3, 13.8, 9.8. ^{119}Sn NMR (149 MHz, CDCl$_3$) δ –53.6. API-MS calcd. for $C_{28}H_{43}NO_2SSn$+Na [M+Na]$^+$ 600.19287; found 600.19273.

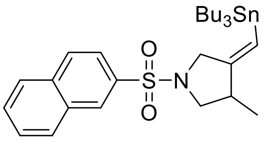

(*Z*)-N-(But-3-en-1-yl)-4-methyl-N-(3-(tributylstannyl)allyl)benzenesulfonamide (**41**).

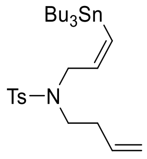

Colorless liquid (90 mg, 81%). ^{1}H NMR (400 MHz, $CDCl_3$) δ 7.69 (d, *J* = 8.4 Hz, 2H), 7.30 (d, *J* = 8.3 Hz, 2H), 6.30 (dt, $^{3}J_{H-H}$ = 12.7 Hz, 1H), 6.01 (dt, $^{3}J_{H-H}$ = 12.8 Hz, 1H), 5.75-5.65 (m, 1H), 5.06-5.00 (m, 2H), 3.77 (d, *J* = 6.3 Hz, 2H), 3.14-3.10 (m, 2H), 2.42 (s, 3H), 2.35-2.29 (m, 2H), 1.47-1.41 (m, 6H), 1.33-1.24 (m, 7H), 0.90-0.83 (m, 15H). ^{13}C NMR (101 MHz, $CDCl_3$) δ 143.7, 143.3, 136.9, 134.8, 133.2, 129.8, 127.3, 117.0, 53.6, 47.1, 33.7, 29.2, 27.5, 21.6, 13.8, 10.4. ^{119}Sn NMR (149 MHz, $CDCl_3$) δ –60.6. API-MS calcd. for $C_{26}H_{46}NSO_2Sn$ $[M+H]^+$ 556.22657; found 556.22687.

(*Z*)-N,4-Dimethyl-N-(3-(tributylstannyl)allyl)benzenesulfonamide (**43**). Colorless liquid (90

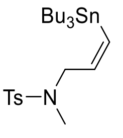

mg, 89%). ^{1}H NMR (400 MHz, $CDCl_3$) δ 7.66 (d, *J* = 8.3 Hz, 2H), 7.32 (d, *J* = 8.5 Hz, 2H), 6.36 (dt, $^{3}J_{H-H}$ = 12.9 Hz, 1H), 6.07 (dt, $^{3}J_{H-H}$ = 12.6 Hz, 1H), 3.57 (d, *J* = 6.5 Hz, 2H), 2.61 (s, 3H), 2.43 (s, 3H), 1.44-1.36 (m, 6H), 1.28-1.21 (m, 6H), 0.88-0.78 (m, 16H). ^{13}C NMR (101 MHz, $CDCl_3$) δ 143.5, 142.7, 134.5, 133.9, 129.7, 127.7, 55.4, 34.4, 29.2, 27.3, 21.6, 13.8, 10.4. ^{119}Sn NMR (149 MHz, $CDCl_3$) δ –60.2. API-MS calcd. for $C_{23}H_{42}NSO_2Sn$ $[M+H]^+$ 516.19527; found 516.19556.

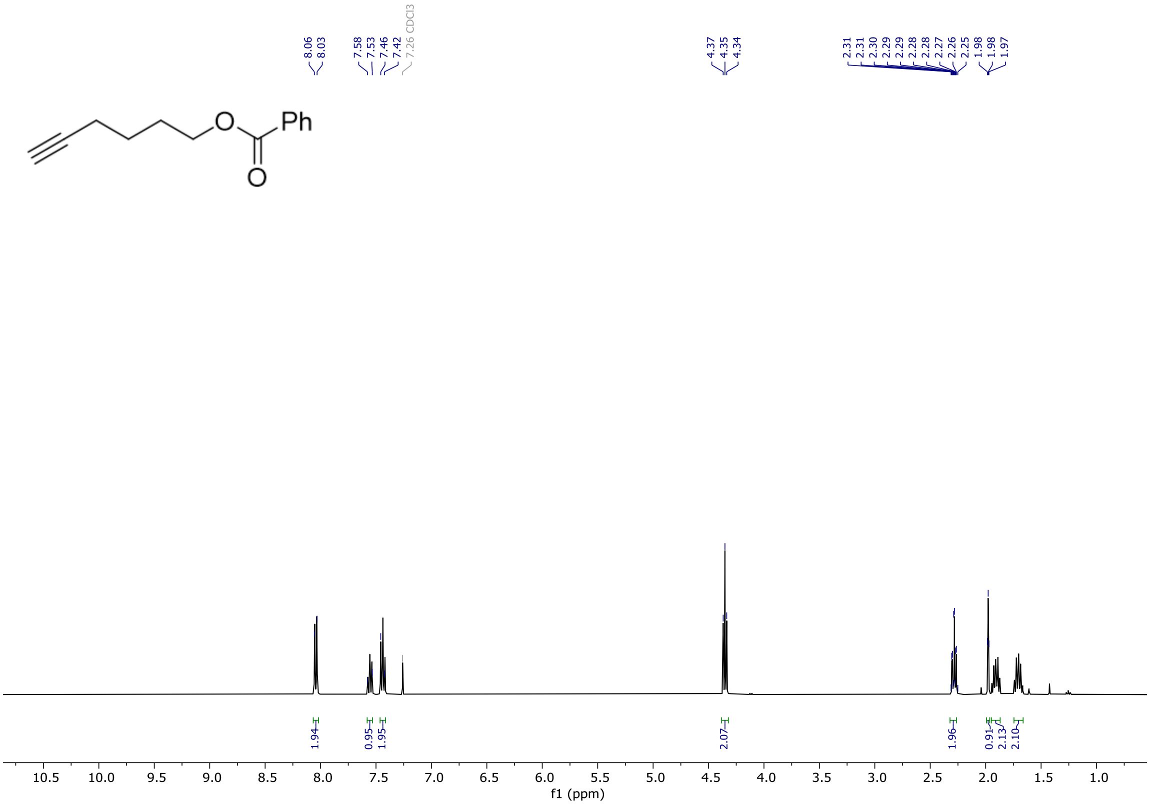
8.06
8.03
7.58
7.53
7.46
7.42
7.26 CDCl3
4.37
4.35
4.34
2.31
2.31
2.30
2.29
2.29
2.28
2.28
2.27
2.26
2.25
1.98
1.98
1.97
O
Ph
O
1.94
0.95
1.95
2.07
1.96
0.91
2.13
2.10
f1 (ppm)

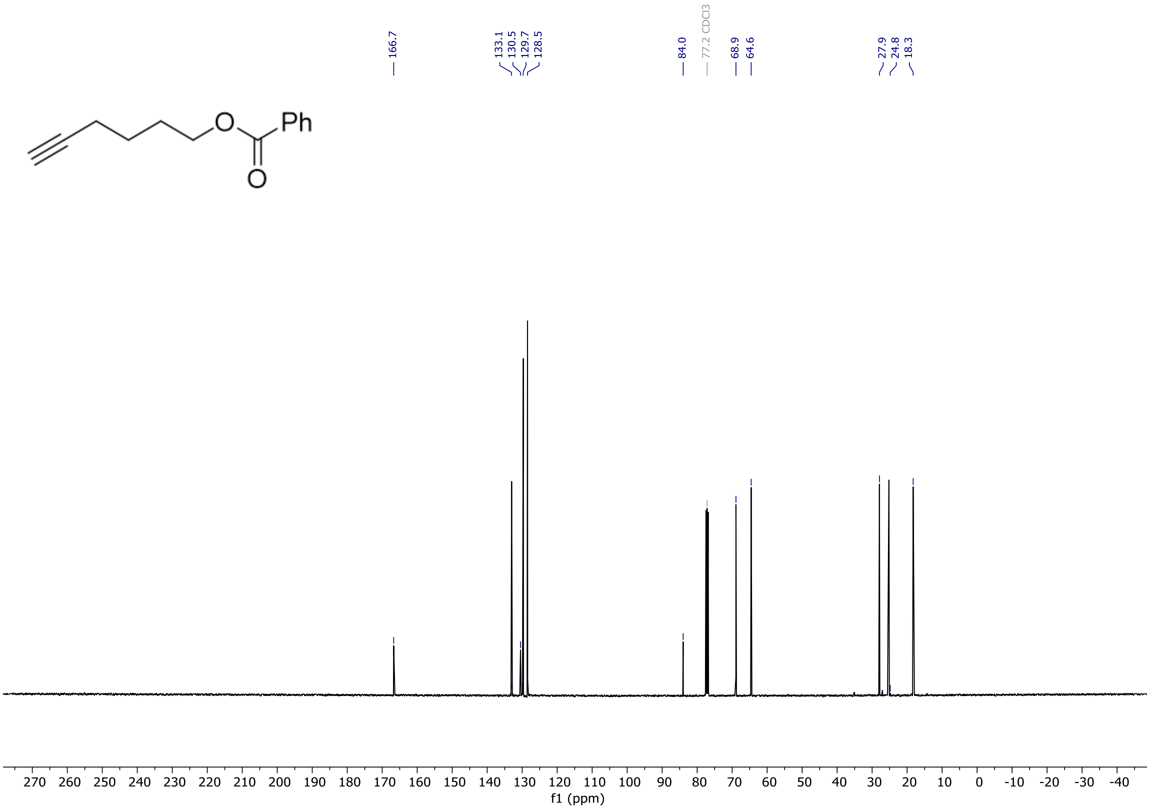
166.7
133.1
130.5
129.7
128.5
84.0
77.2 CDCl3
68.9
64.6
27.9
24.8
18.3
O
Ph
O
f1 (ppm)

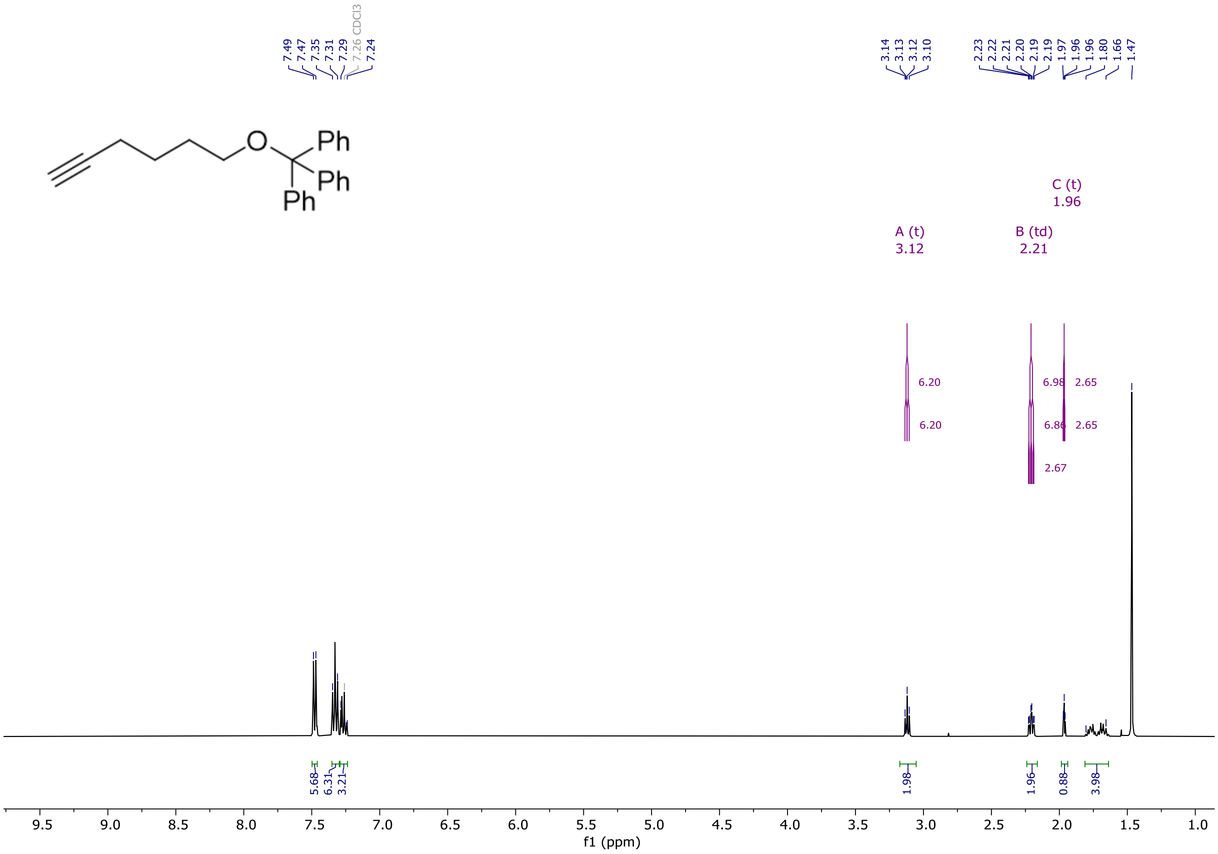
7.49
7.47
7.35
7.31
7.29
7.26 CDCl3
7.24
3.14
3.13
3.12
3.10
2.23
2.22
2.21
2.20
2.19
2.19
1.97
1.96
1.96
1.80
1.66
1.47
Ph
Ph
Ph
O
C (t)
1.96
A (t)
3.12
B (td)
2.21
6.20
6.20
6.98
6.86
2.67
2.65
2.65
5.68
6.31
3.21
1.98
1.96
0.88
3.98
9.5 9.0 8.5 8.0 7.5 7.0 6.5 6.0 5.5 5.0 4.5 4.0 3.5 3.0 2.5 2.0 1.5 1.0
f1 (ppm)

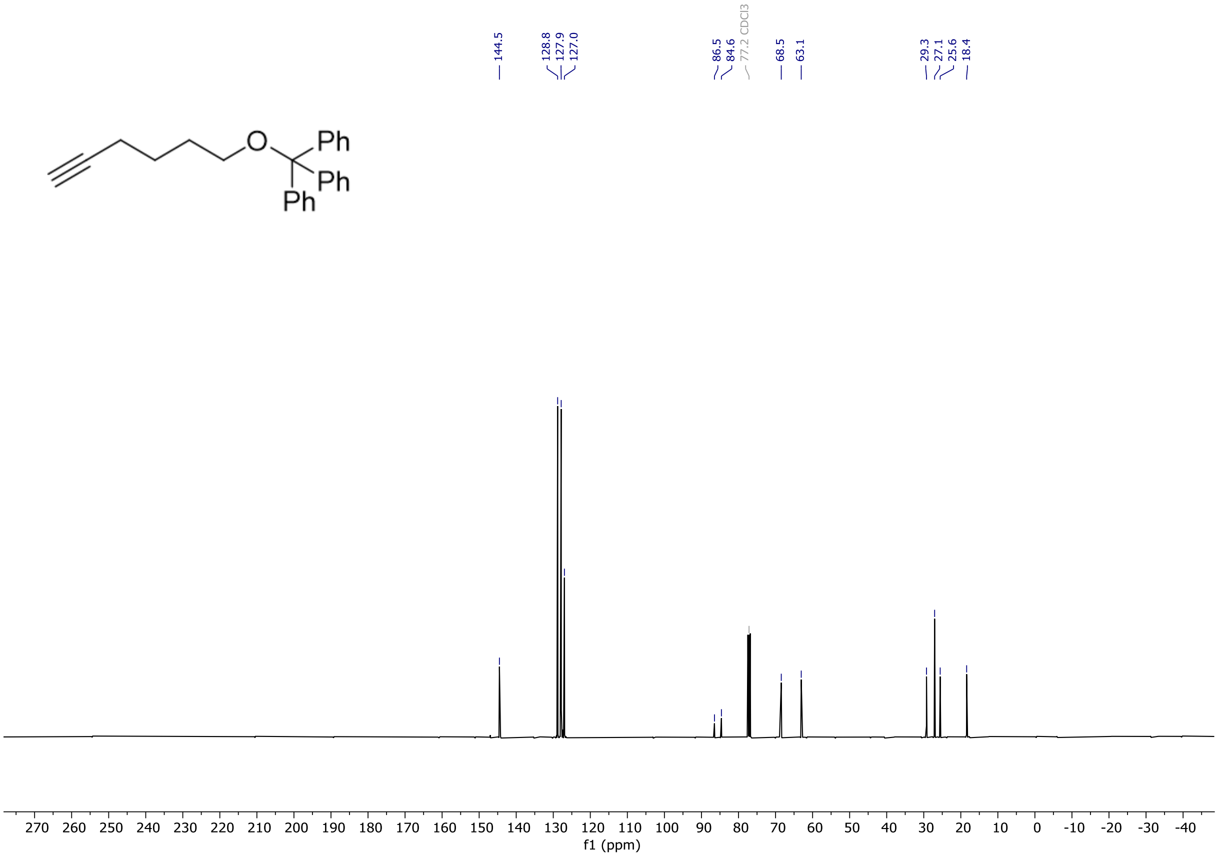
144.5
128.8
127.9
127.0
86.5
84.6
77.2 CDCl3
68.5
63.1
29.3
27.1
25.6
18.4
Ph
Ph
Ph
O
270 260 250 240 230 220 210 200 190 180 170 160 150 140 130 120 110 100 90 80 70 60 50 40 30 20 10 0 -10 -20 -30 -40
f1 (ppm)

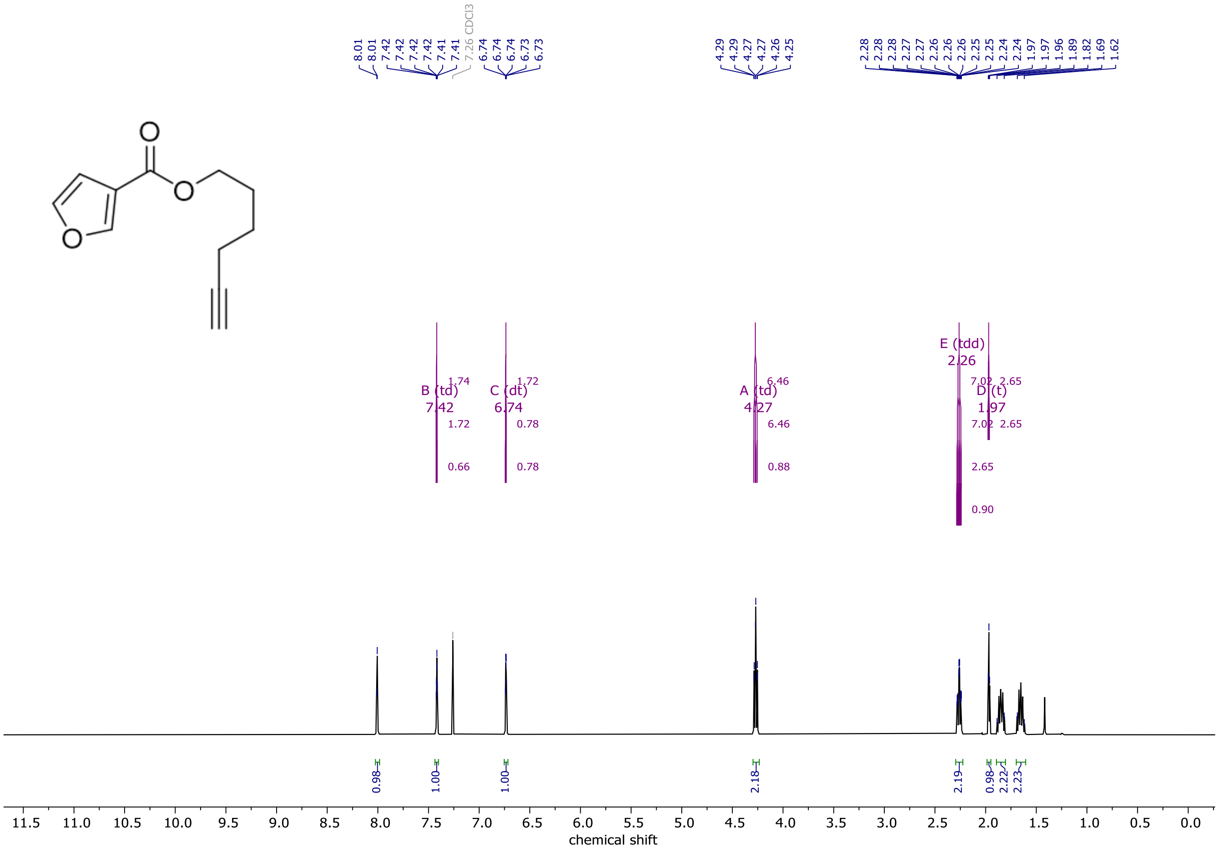
8.01
8.01
7.42
7.42
7.42
7.41
7.41
7.26 CDCl3
6.74
6.74
6.74
6.73
6.73
4.29
4.29
4.27
4.27
4.26
4.25
2.28
2.28
2.28
2.27
2.27
2.26
2.26
2.26
2.25
2.25
2.24
2.24
1.97
1.97
1.96
1.89
1.82
1.69
1.62
B (td)
7.42
C (dt)
6.74
A (td)
4.27
E (tdd)
2.26
D (t)
1.97
0.98
1.00
1.00
2.18
2.19
0.98
2.22
2.23
chemical shift

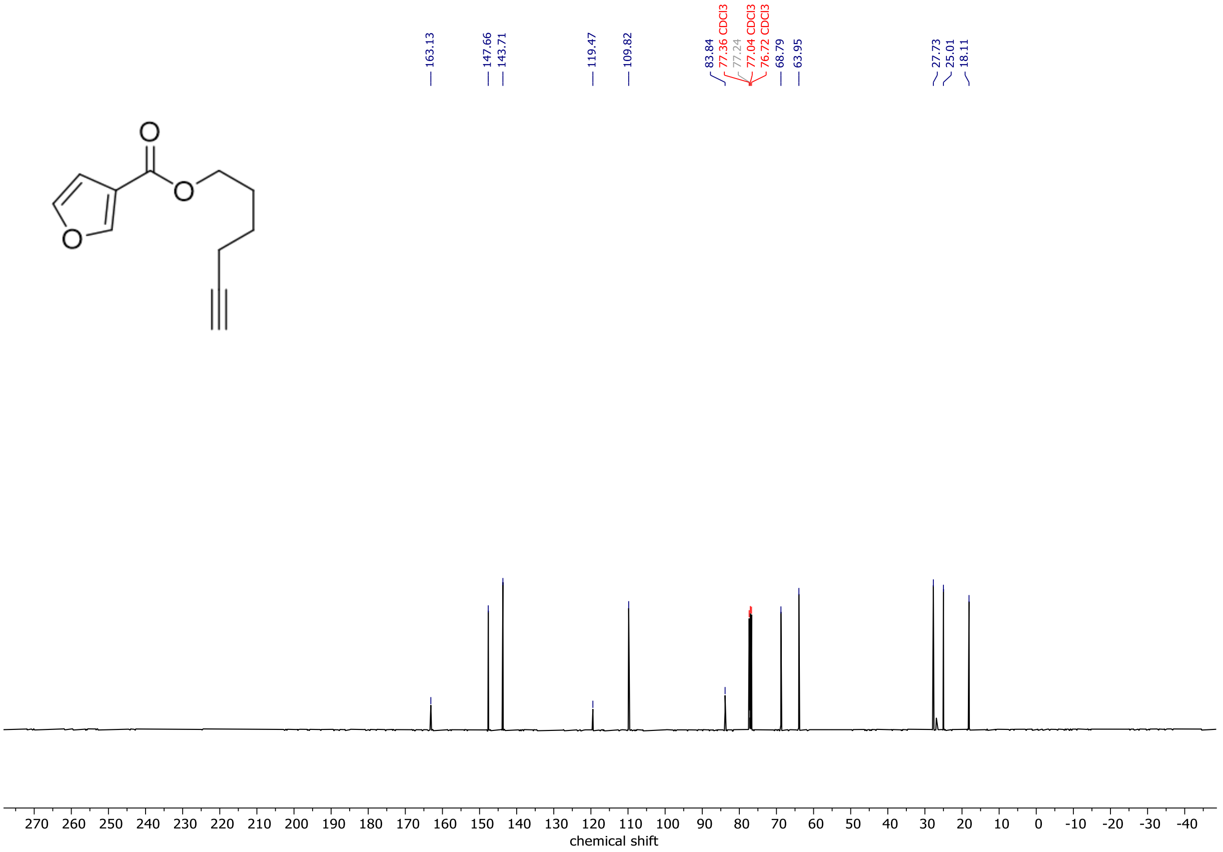
163.13
147.66
143.71
119.47
109.82
83.84
77.36 CDCl3
77.24
77.04 CDCl3
76.72 CDCl3
68.79
63.95
27.73
25.01
18.11
chemical shift

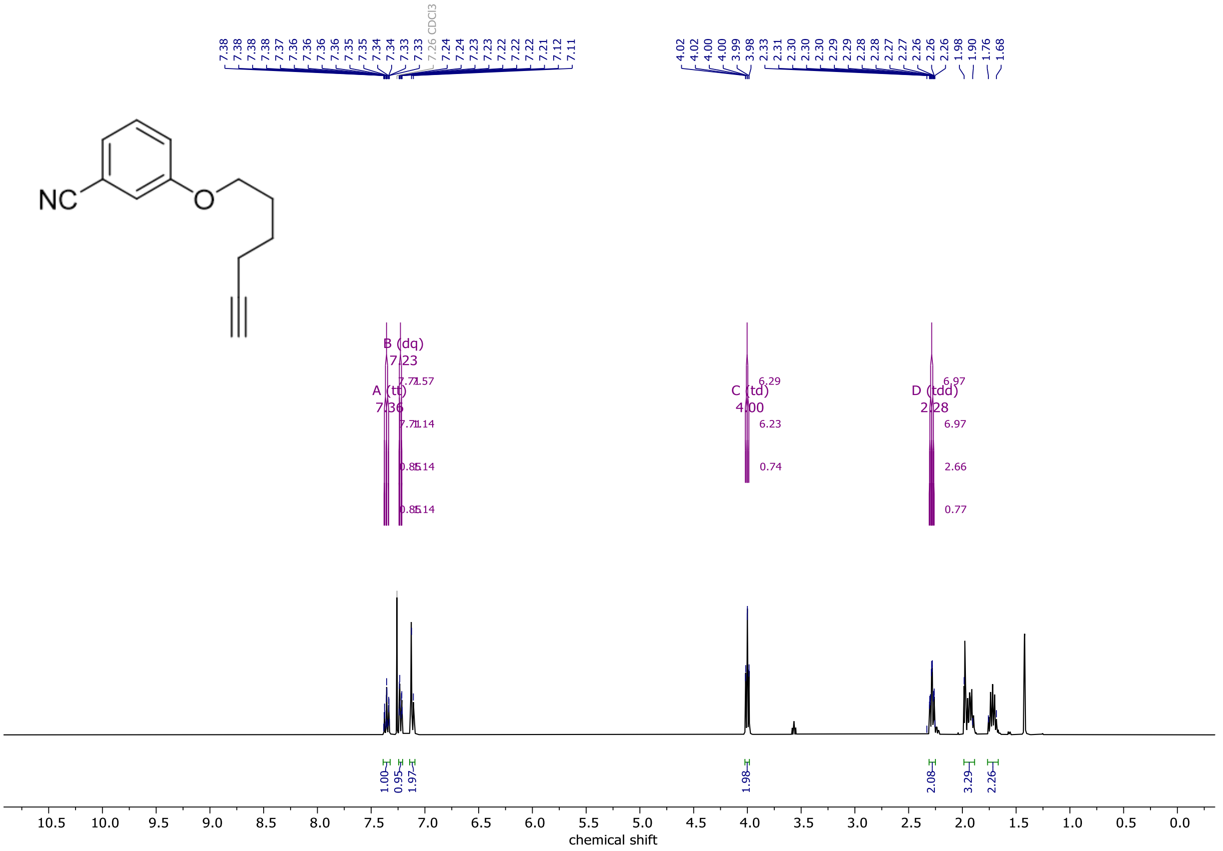

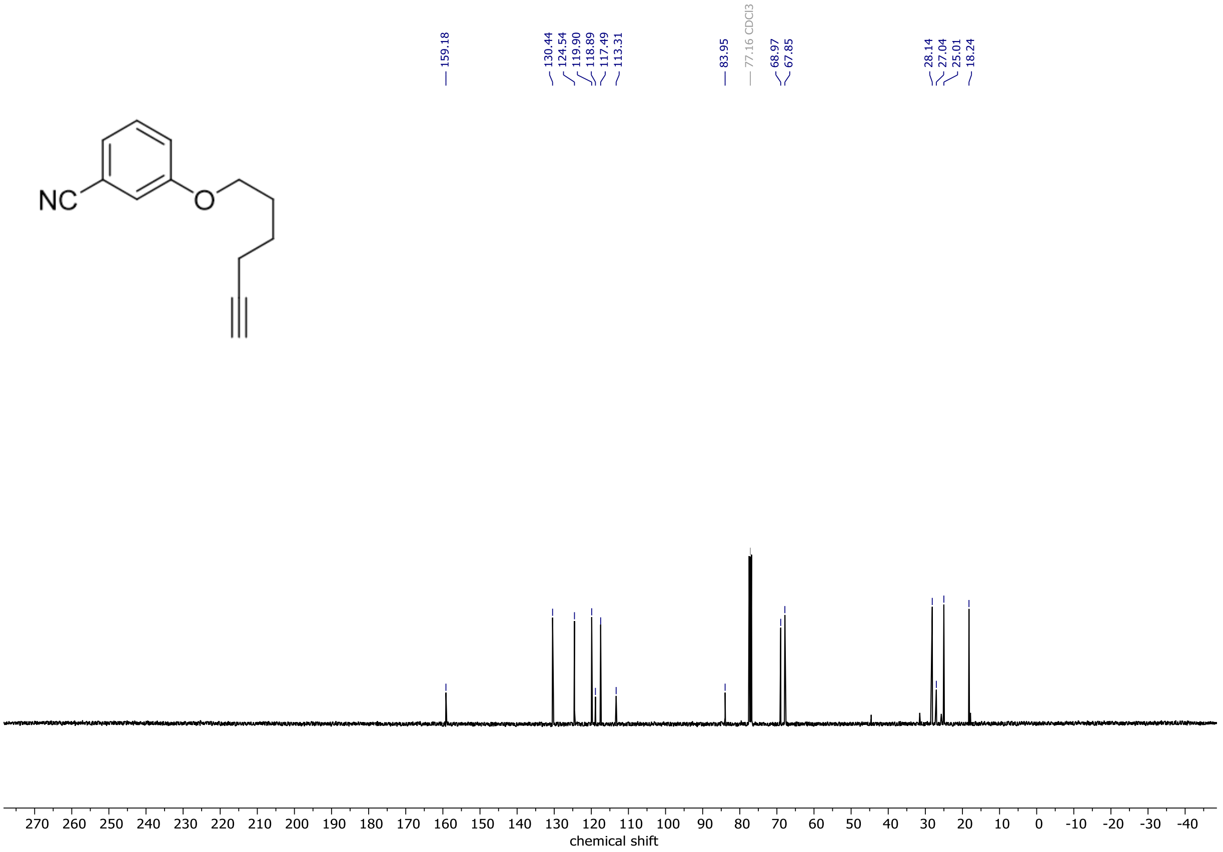

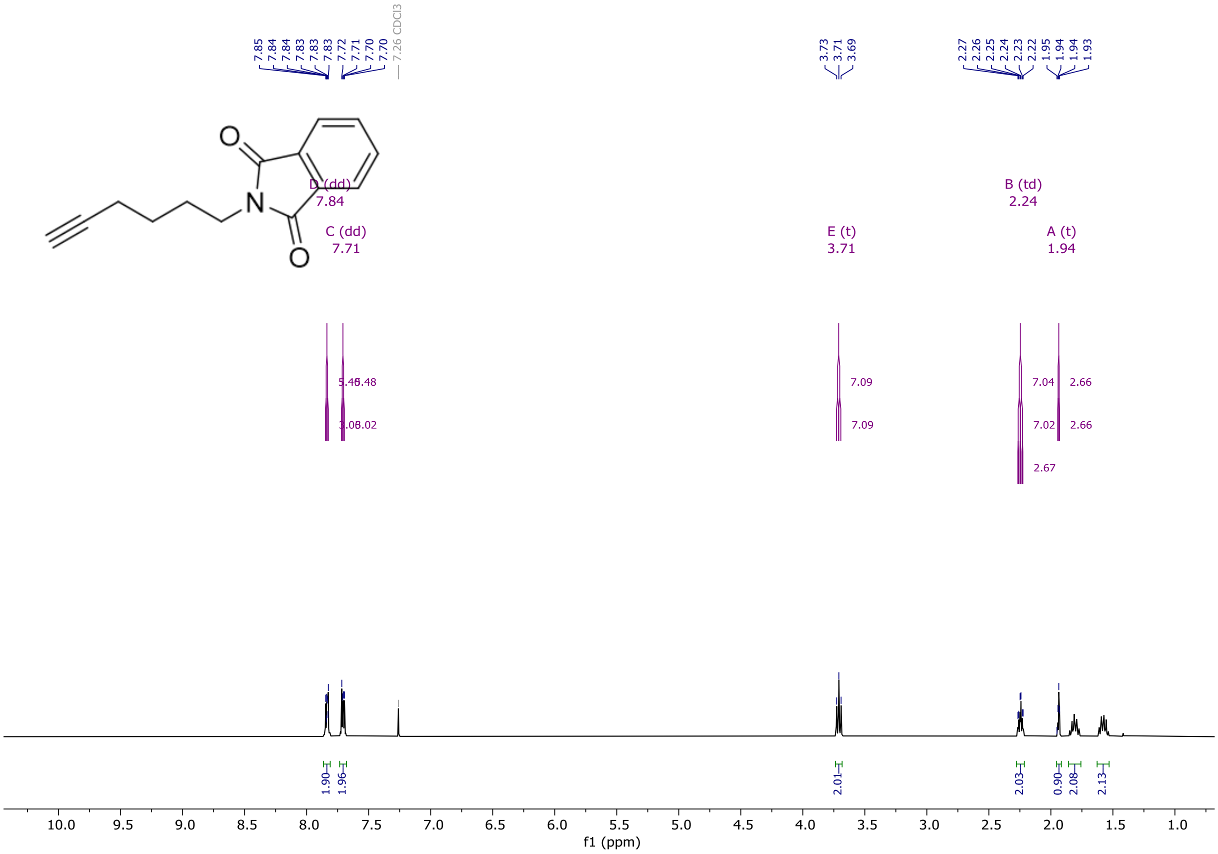

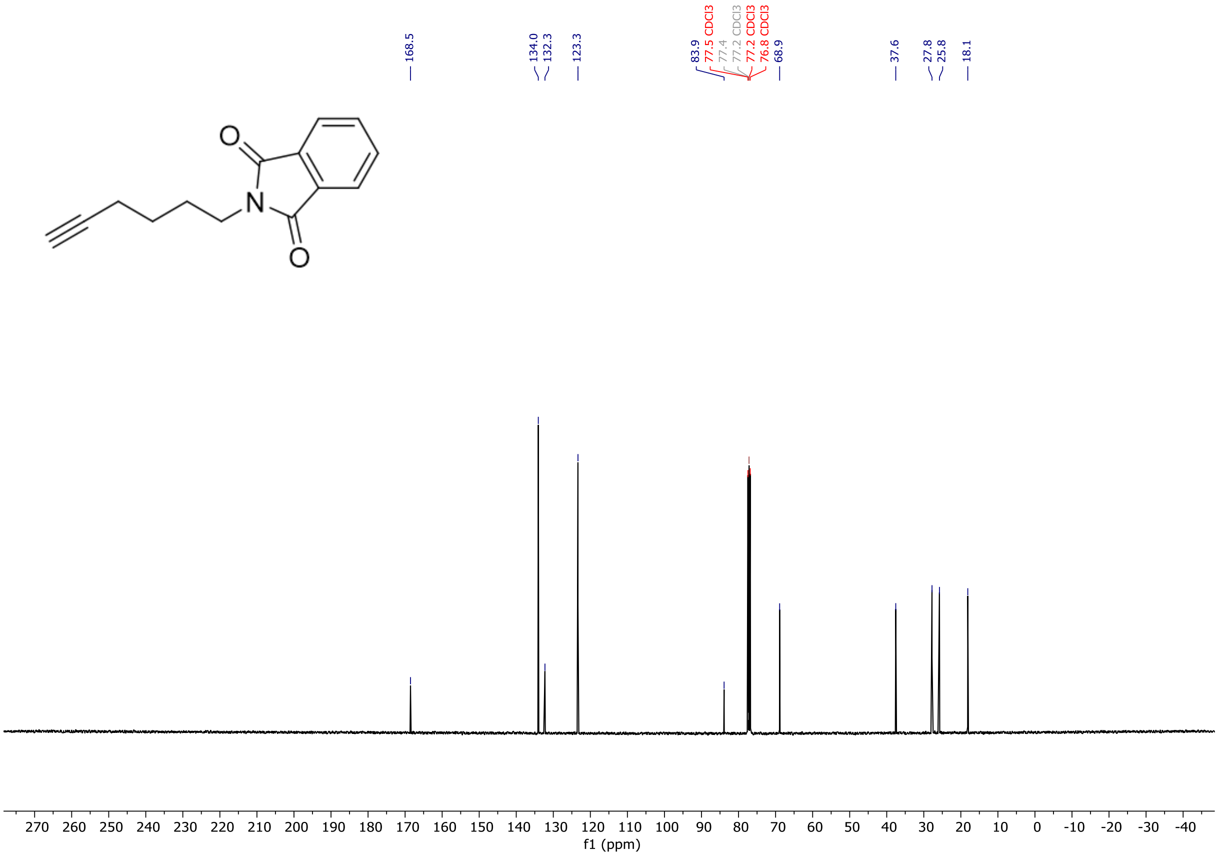

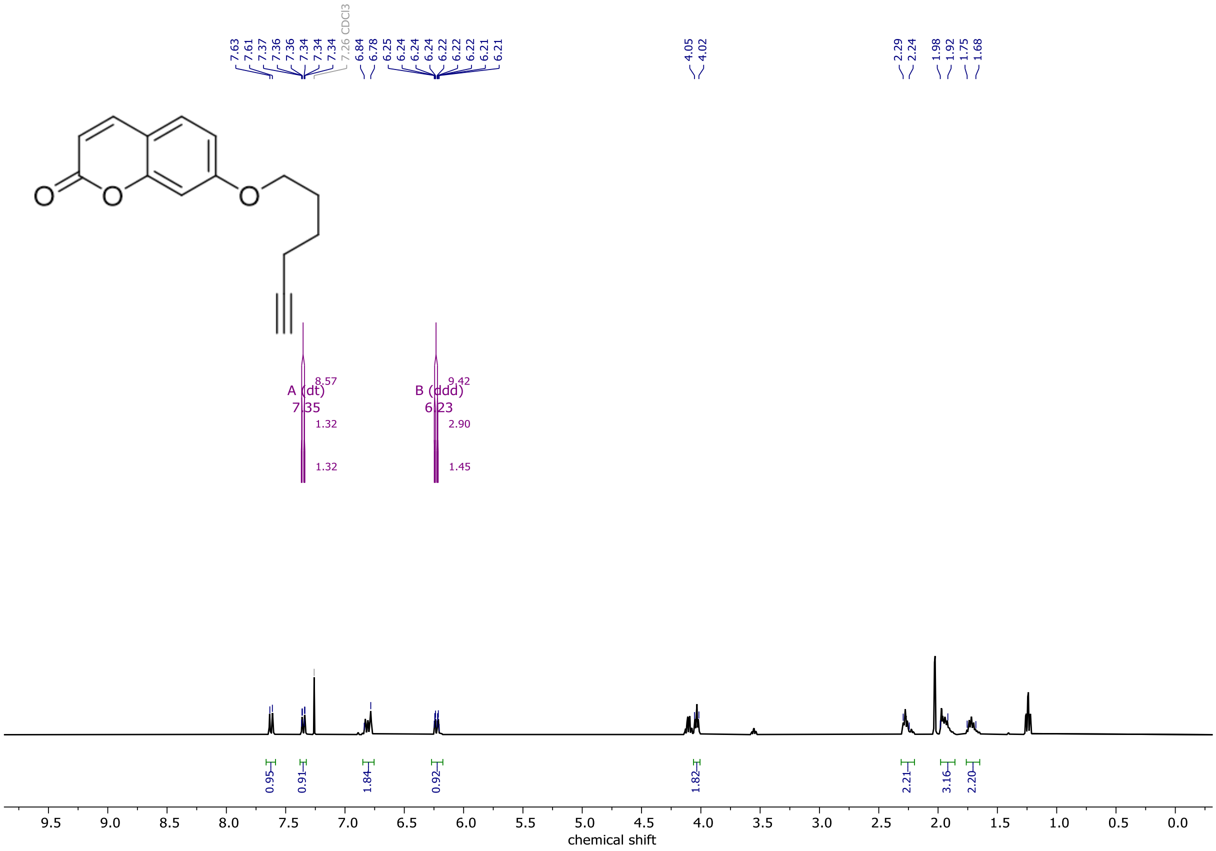
7.63
7.61
7.37
7.36
7.34
7.34
7.34
7.26 CDCl3
6.84
6.78
6.25
6.24
6.24
6.22
6.22
6.22
6.21
6.21
4.05
4.02
2.29
2.24
1.98
1.92
1.75
1.68
A (dt)
7.35
8.57
1.32
1.32
B (ddd)
6.23
9.42
2.90
1.45
0.95
0.91
1.84
0.92
1.82
2.21
3.16
2.20
9.5
9.0
8.5
8.0
7.5
7.0
6.5
6.0
5.5
5.0
4.5
4.0
3.5
3.0
2.5
2.0
1.5
1.0
0.5
0.0
chemical shift

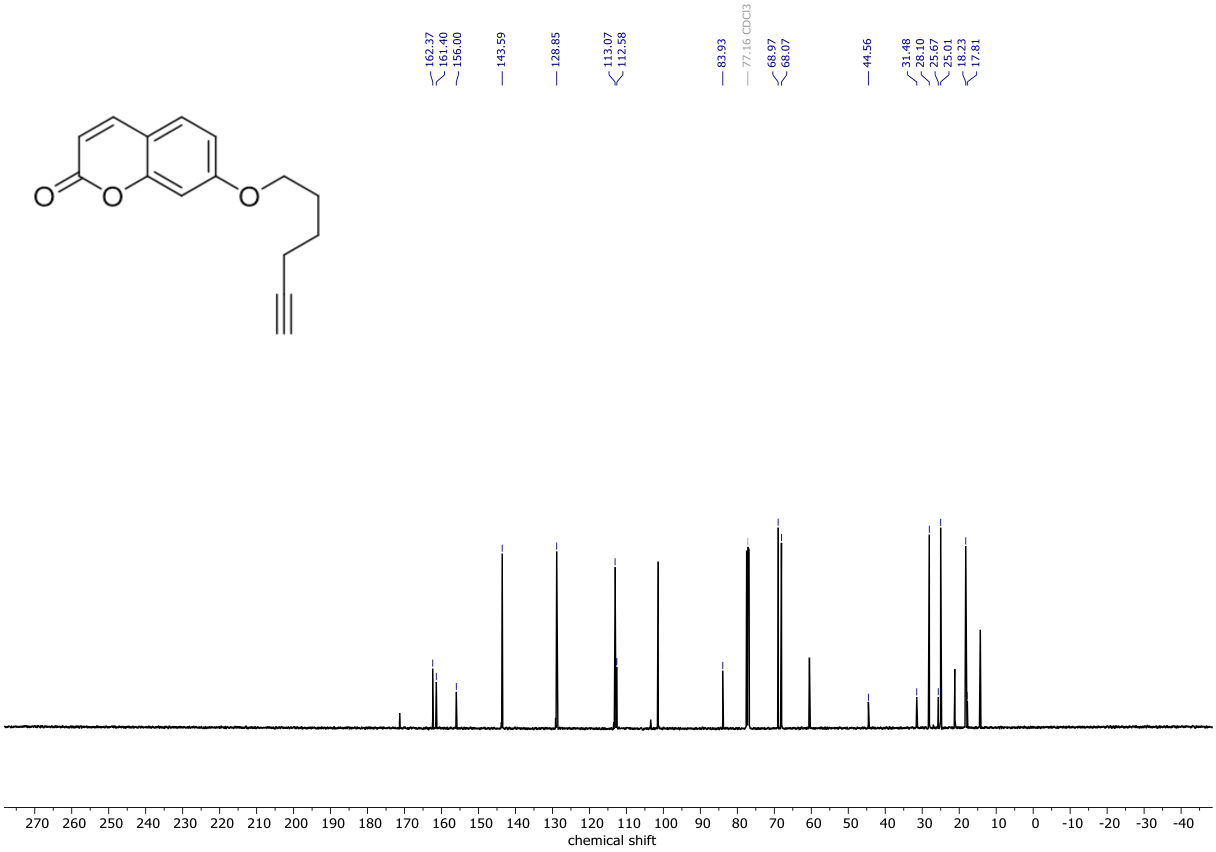
162.37
161.40
156.00
143.59
128.85
113.07
112.58
83.93
77.16 CDCl3
68.97
68.07
44.56
31.48
28.10
25.67
25.01
18.23
17.81
270
260
250
240
230
220
210
200
190
180
170
160
150
140
130
120
110
100
90
80
70
60
50
40
30
20
10
0
-10
-20
-30
-40
chemical shift

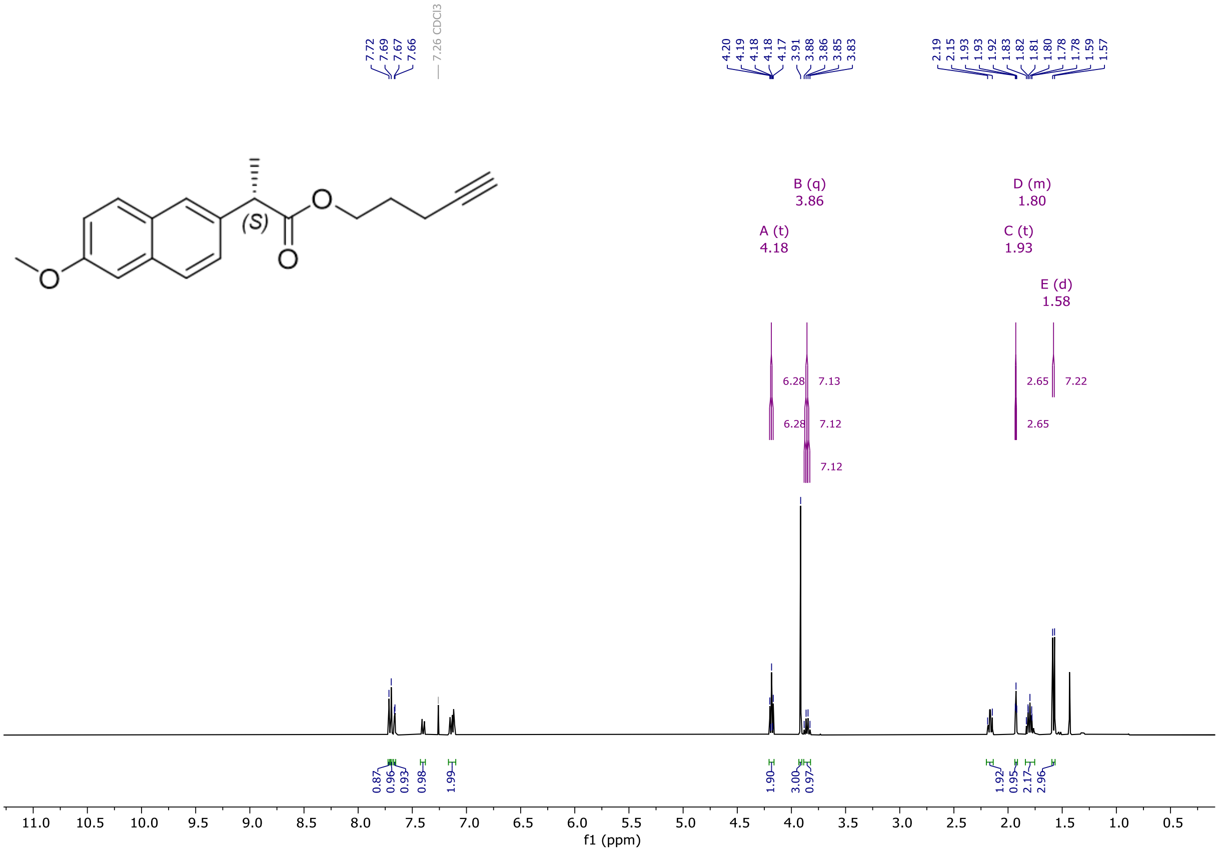

7.72
7.69
7.67
7.66
7.26 CDCl3
4.20
4.19
4.18
4.18
4.17
3.91
3.88
3.86
3.85
3.83
2.19
2.15
1.93
1.93
1.92
1.83
1.82
1.81
1.80
1.78
1.78
1.59
1.57
(S)
B (q)
3.86
A (t)
4.18
D (m)
1.80
C (t)
1.93
E (d)
1.58
0.87
0.96
0.93
0.98
1.99
1.90
3.00
0.97
1.92
0.95
2.17
2.96
f1 (ppm)

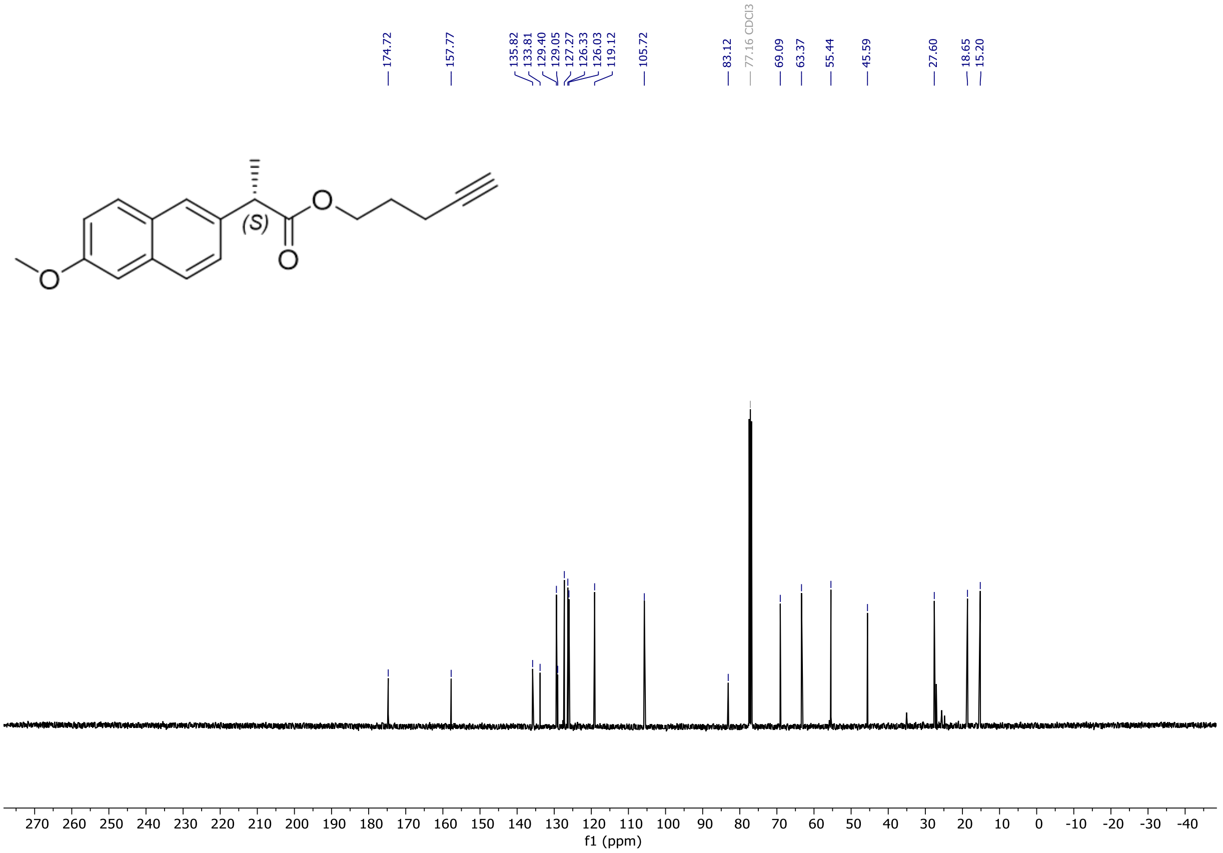

174.72
157.77
135.82
133.81
129.40
129.05
127.27
126.33
126.03
119.12
105.72
83.12
77.16 CDCl3
69.09
63.37
55.44
45.59
27.60
18.65
15.20
(S)
f1 (ppm)

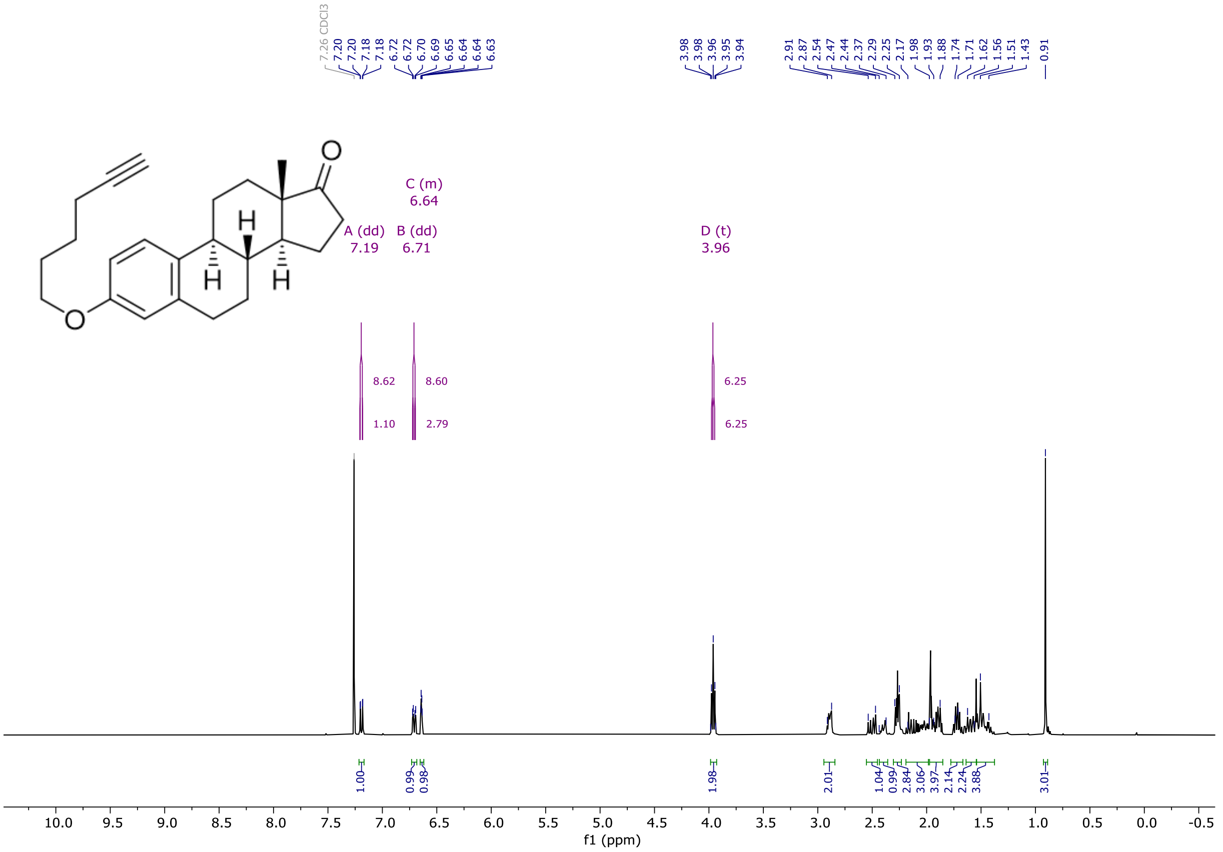
7.26 CDCl3
7.20
7.20
7.18
7.18
6.72
6.72
6.70
6.69
6.65
6.64
6.64
6.63
3.98
3.98
3.96
3.95
3.94
2.91
2.87
2.54
2.47
2.44
2.37
2.29
2.25
2.17
1.98
1.93
1.88
1.74
1.71
1.62
1.56
1.51
1.43
0.91
C (m)
6.64
A (dd)
7.19
B (dd)
6.71
D (t)
3.96
8.62
1.10
8.60
2.79
6.25
6.25
1.00
0.99
0.98
1.98
2.01
1.04
0.99
2.84
3.06
3.97
2.14
2.24
3.88
3.01
f1 (ppm)

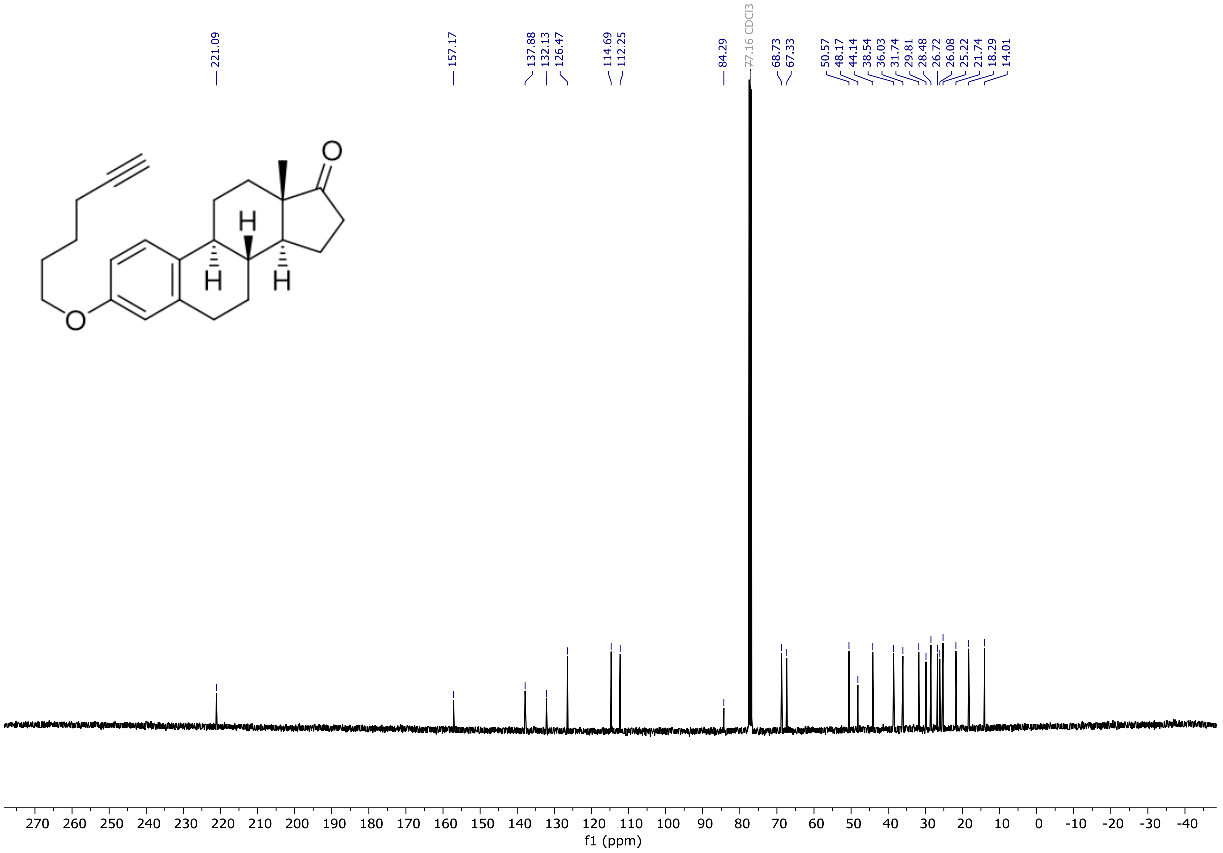
221.09
157.17
137.88
132.13
126.47
114.69
112.25
84.29
77.16 CDCl3
68.73
67.33
50.57
48.17
44.14
38.54
36.03
31.74
29.81
28.48
26.72
26.08
25.22
21.74
18.29
14.01
f1 (ppm)

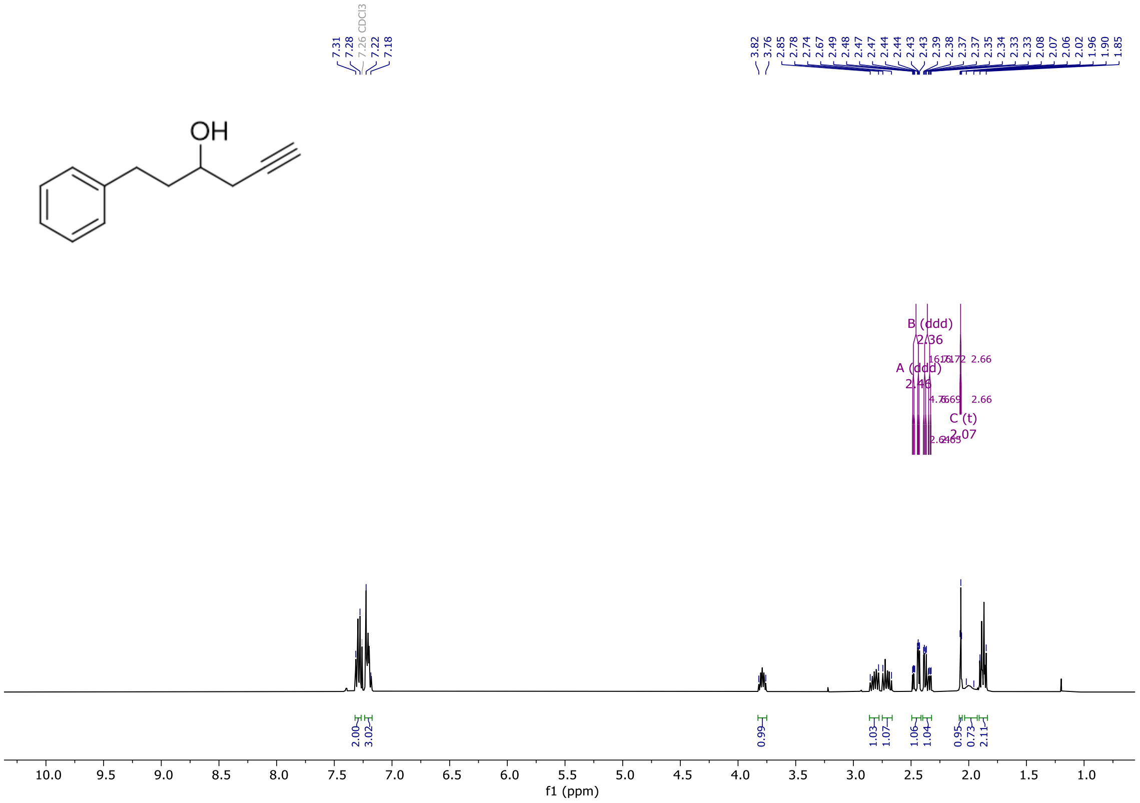

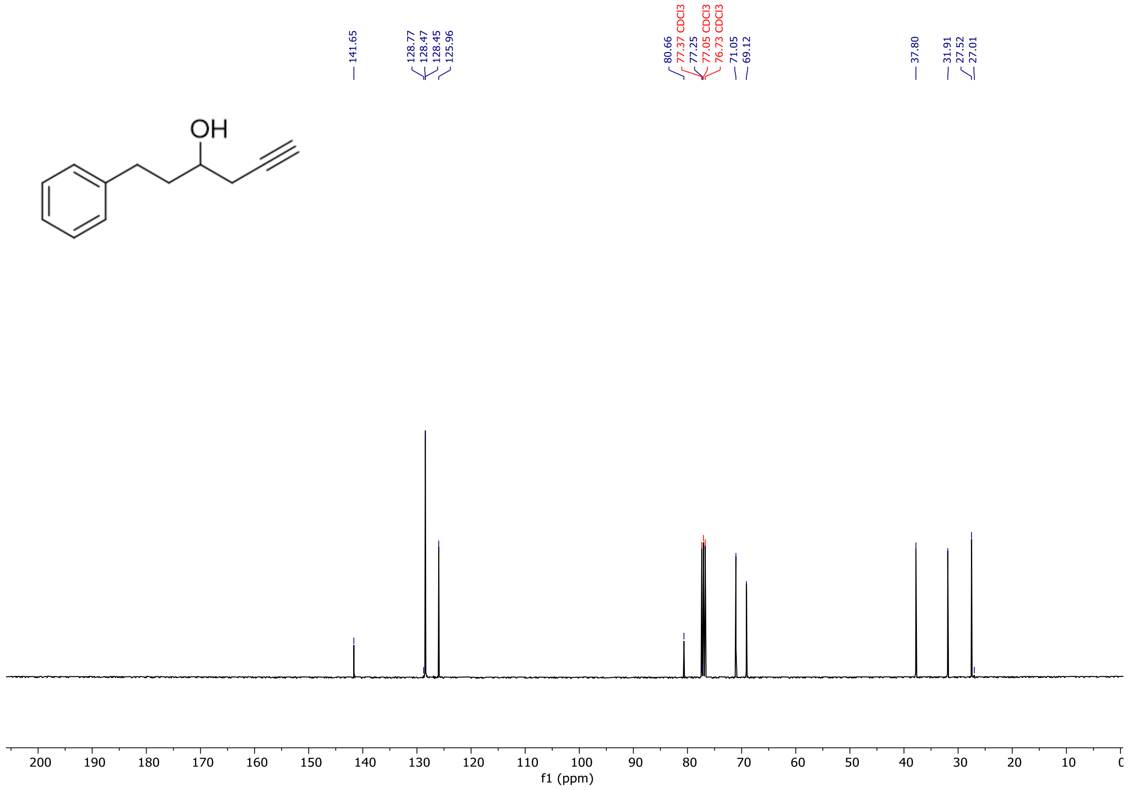

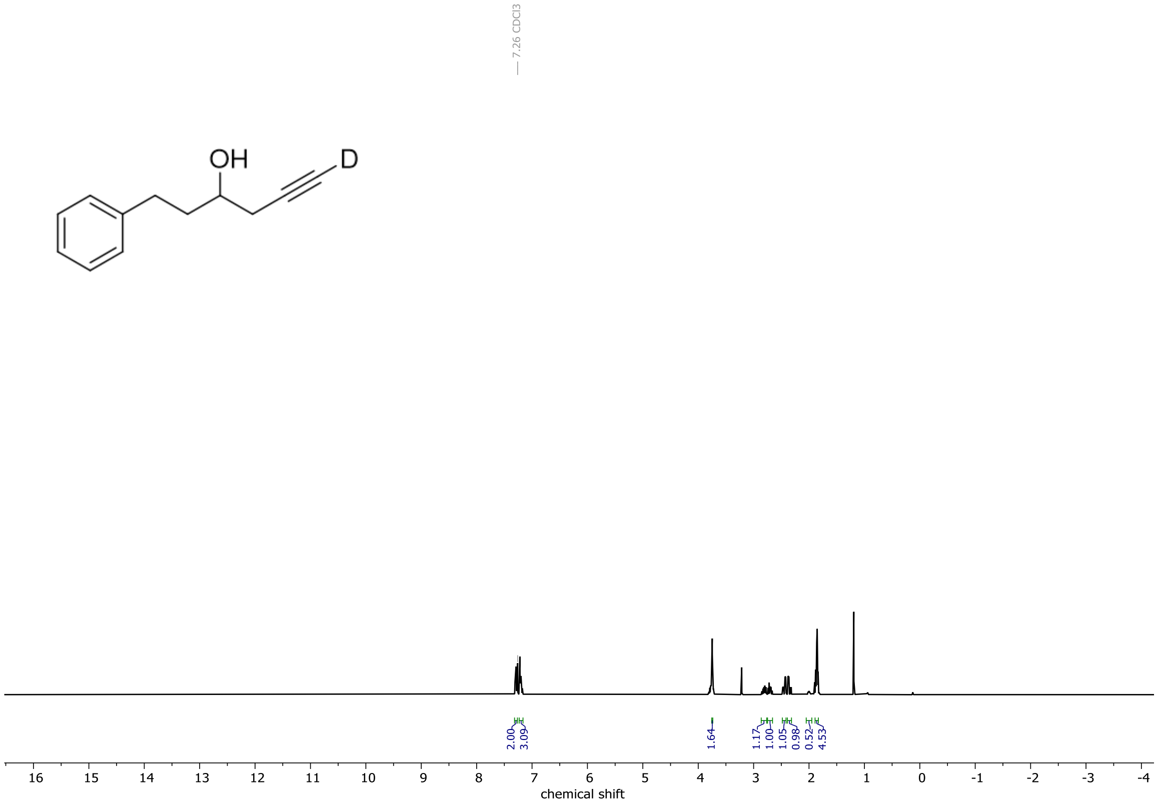
7.26 CDCl3
OH
D
2.00
3.09
1.64
1.17
1.00
1.05
0.98
0.52
4.53
16 15 14 13 12 11 10 9 8 7 6 5 4 3 2 1 0 -1 -2 -3 -4
chemical shift

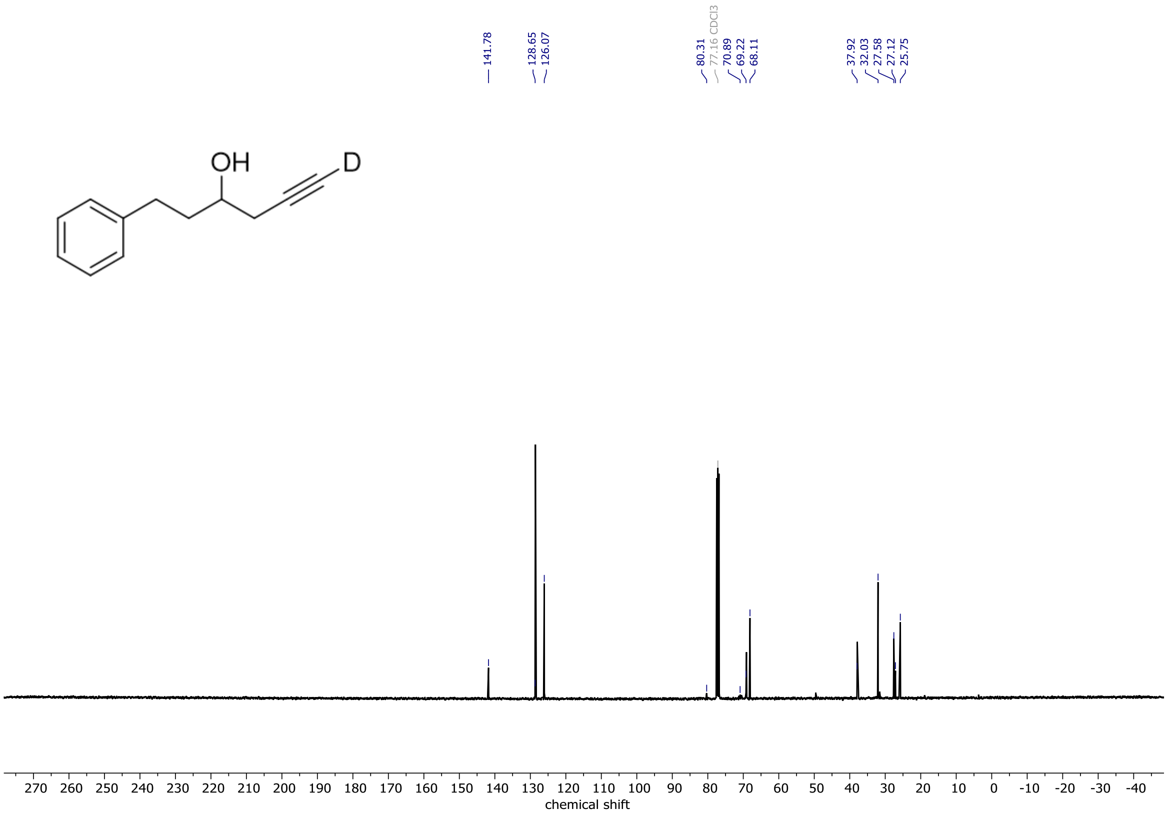
141.78
128.65
126.07
80.31
77.16 CDCl3
70.89
69.22
68.11
37.92
32.03
27.58
27.12
25.75
OH
D
270 260 250 240 230 220 210 200 190 180 170 160 150 140 130 120 110 100 90 80 70 60 50 40 30 20 10 0 -10 -20 -30 -40
chemical shift

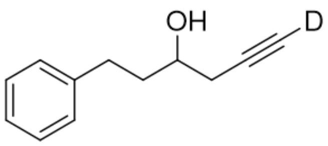

OH
D

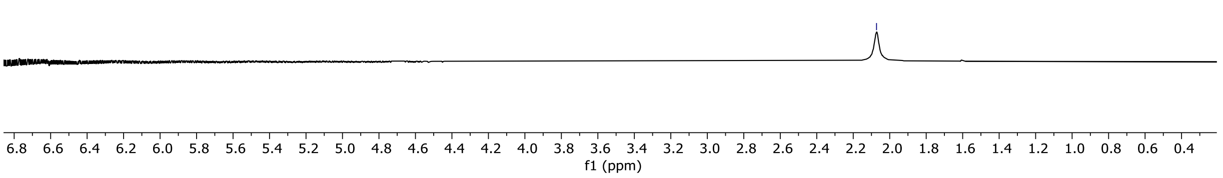

2.07
6.8 6.6 6.4 6.2 6.0 5.8 5.6 5.4 5.2 5.0 4.8 4.6 4.4 4.2 4.0 3.8 3.6 3.4 3.2 3.0 2.8 2.6 2.4 2.2 2.0 1.8 1.6 1.4 1.2 1.0 0.8 0.6 0.4
f1 (ppm)

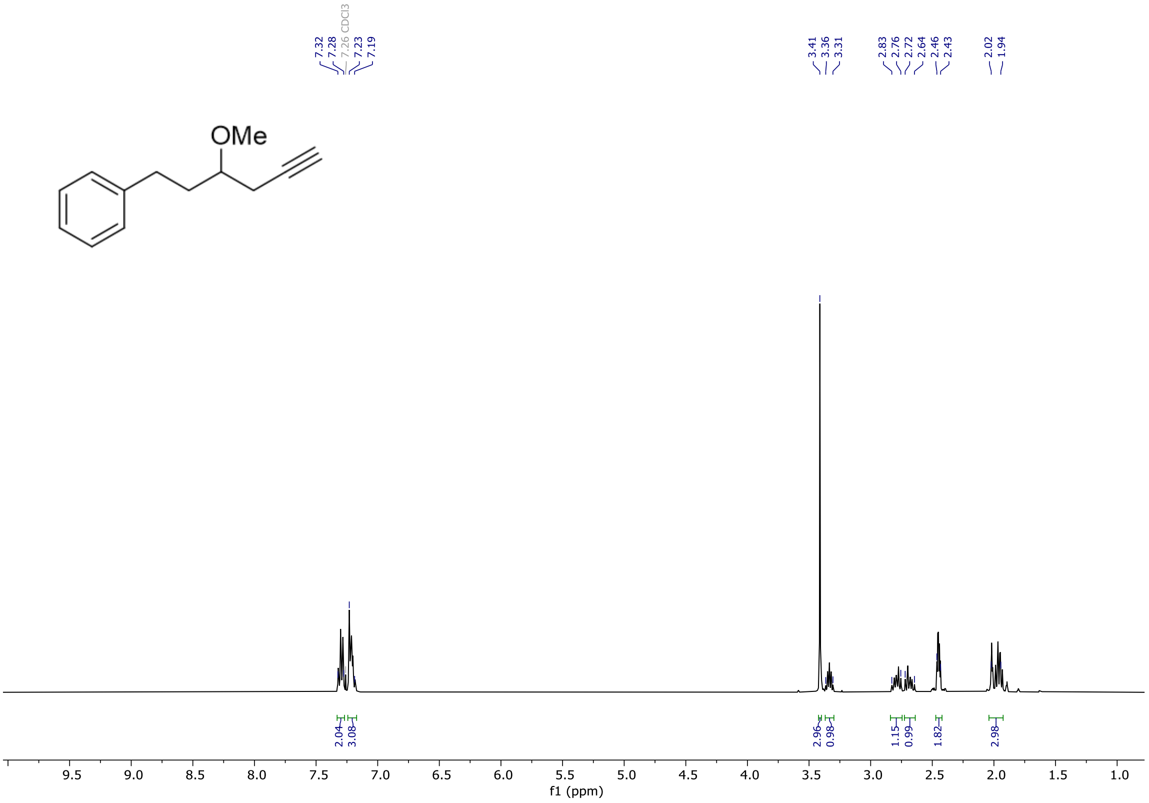
7.32
7.28
7.26 CDCl3
7.23
7.19
3.41
3.36
3.31
2.83
2.76
2.72
2.64
2.46
2.43
2.02
1.94
OMe
2.04
3.08
2.96
0.98
1.15
0.99
1.82
2.98
f1 (ppm)

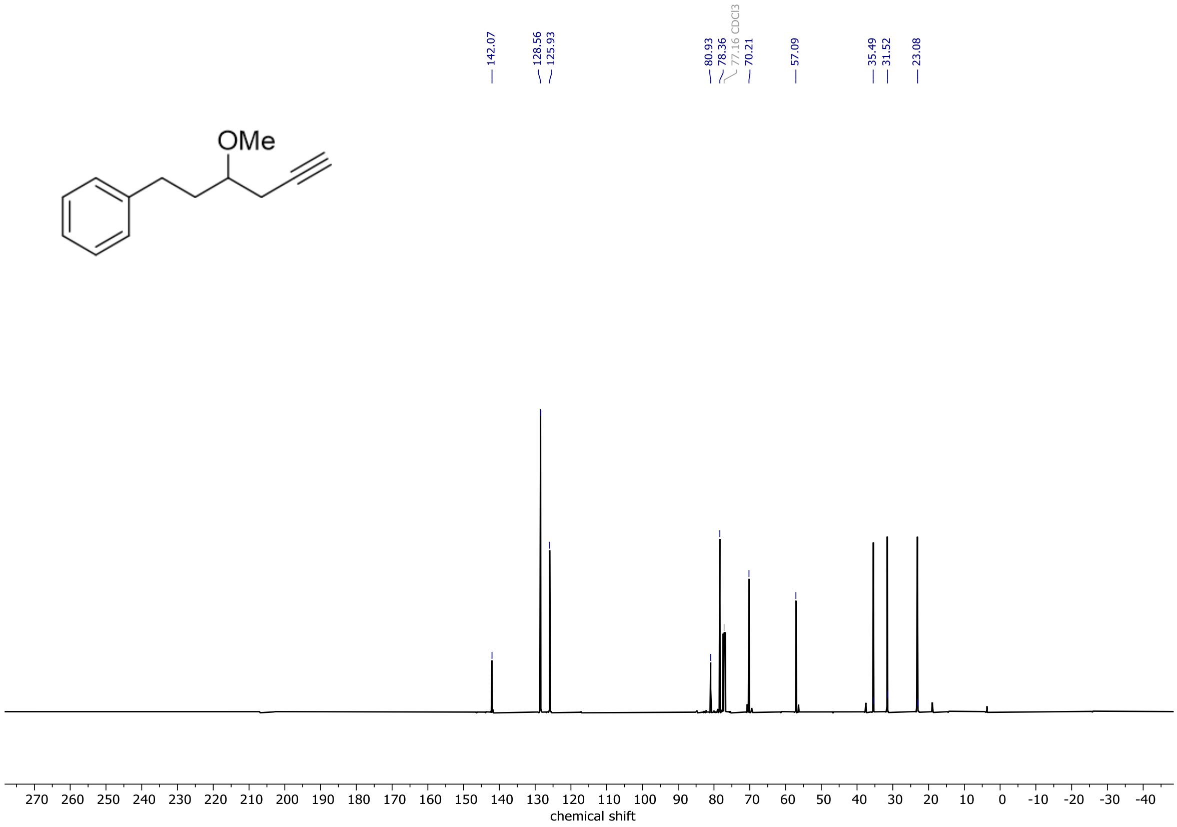
142.07
128.56
125.93
80.93
78.36
77.16 CDCl3
70.21
57.09
35.49
31.52
23.08
OMe
chemical shift

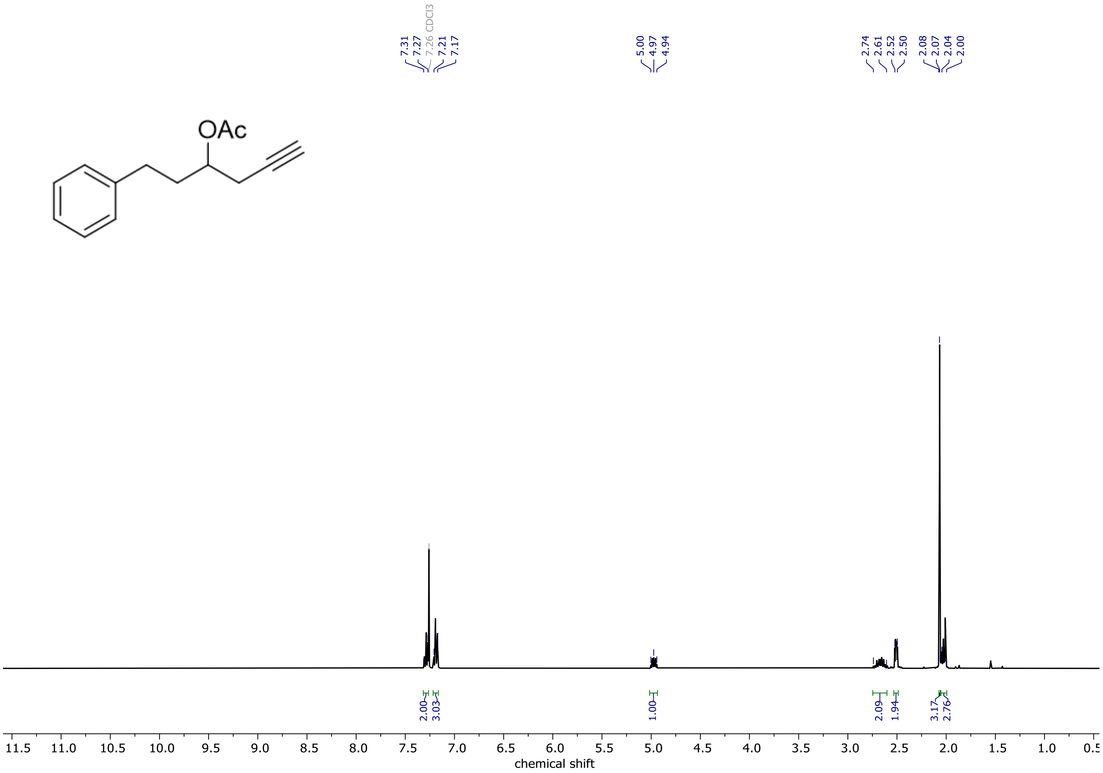
7.31
7.27
7.26 CDCl3
7.21
7.17
5.00
4.97
4.94
2.74
2.61
2.52
2.50
2.08
2.07
2.04
2.00
OAc
2.00
3.03
1.00
2.09
1.94
3.17
2.76
11.5 11.0 10.5 10.0 9.5 9.0 8.5 8.0 7.5 7.0 6.5 6.0 5.5 5.0 4.5 4.0 3.5 3.0 2.5 2.0 1.5 1.0 0.5
chemical shift

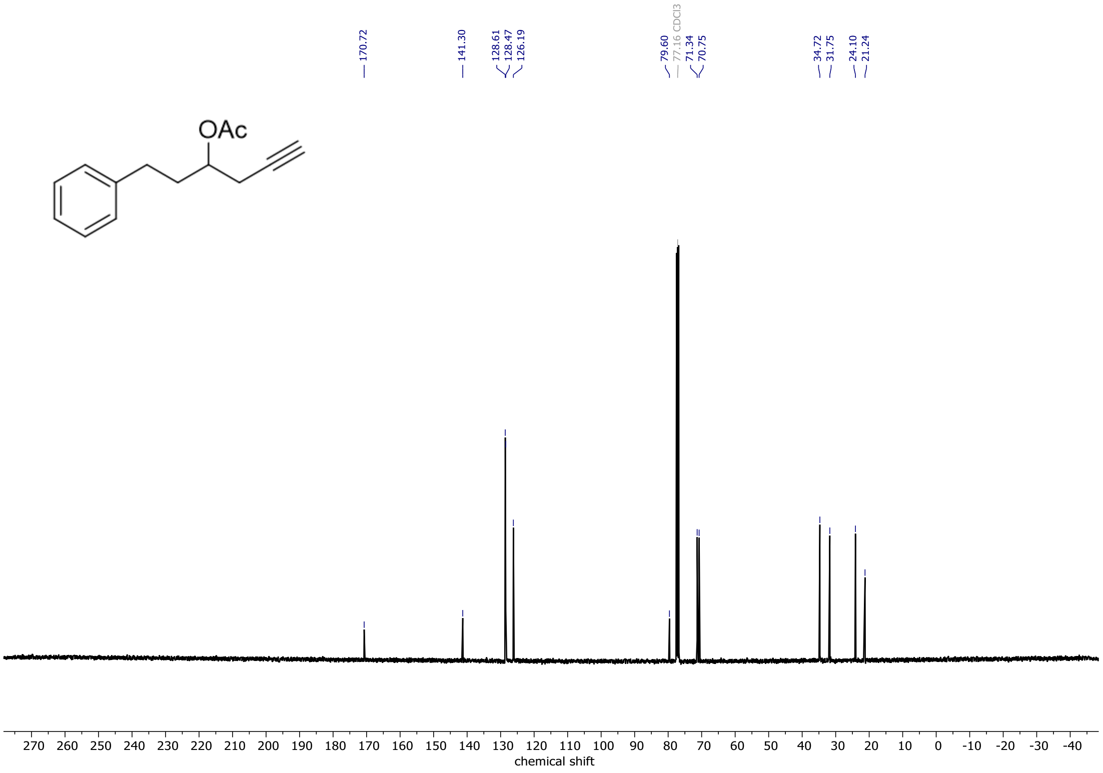
170.72
141.30
128.61
128.47
126.19
79.60
77.16 CDCl3
71.34
70.75
34.72
31.75
24.10
21.24
OAc
270 260 250 240 230 220 210 200 190 180 170 160 150 140 130 120 110 100 90 80 70 60 50 40 30 20 10 0 -10 -20 -30 -40
chemical shift

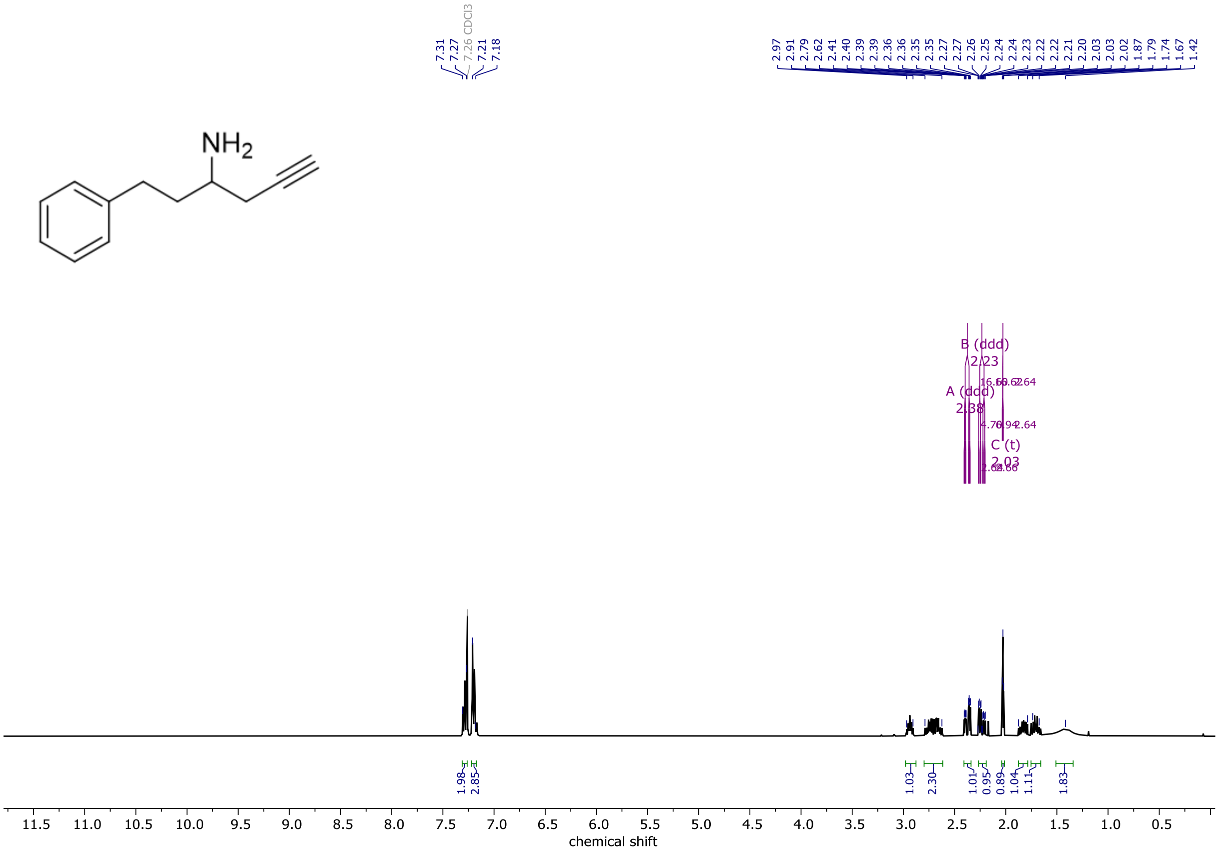

NH2
7.31
7.27
7.26 CDCl3
7.21
7.18
2.97
2.91
2.79
2.62
2.41
2.40
2.39
2.39
2.36
2.36
2.35
2.35
2.27
2.27
2.26
2.25
2.24
2.24
2.23
2.22
2.22
2.21
2.20
2.03
2.03
2.02
1.87
1.79
1.74
1.67
1.42
B (ddd)
2.23
A (ddd)
2.38
C (t)
2.03
1.98
2.85
1.03
2.30
1.01
0.95
0.89
1.04
1.11
1.83
chemical shift

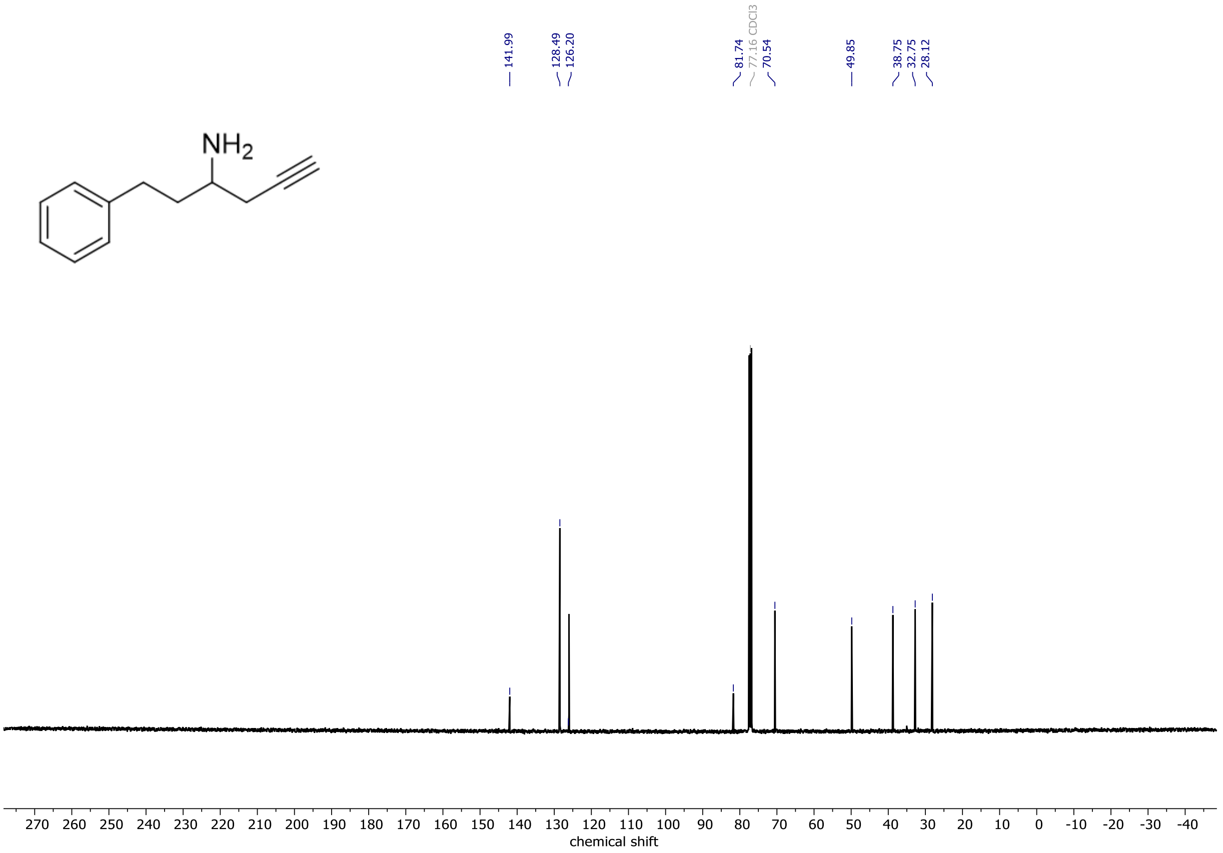

141.99
128.49
126.20
81.74
77.16 CDCl3
70.54
49.85
38.75
32.75
28.12
NH2
chemical shift

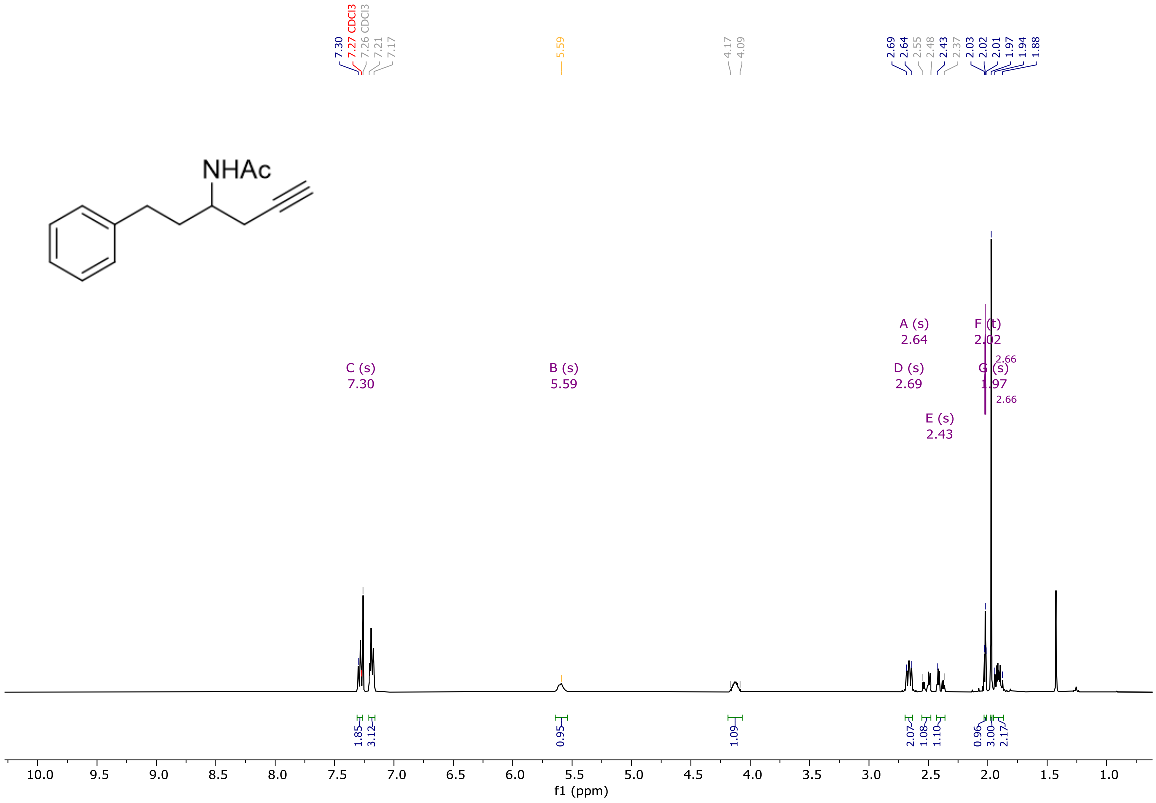
NHAc
C (s)
7.30
B (s)
5.59
A (s)
2.64
D (s)
2.69
E (s)
2.43
F (t)
2.02
G (s)
1.97
f1 (ppm)

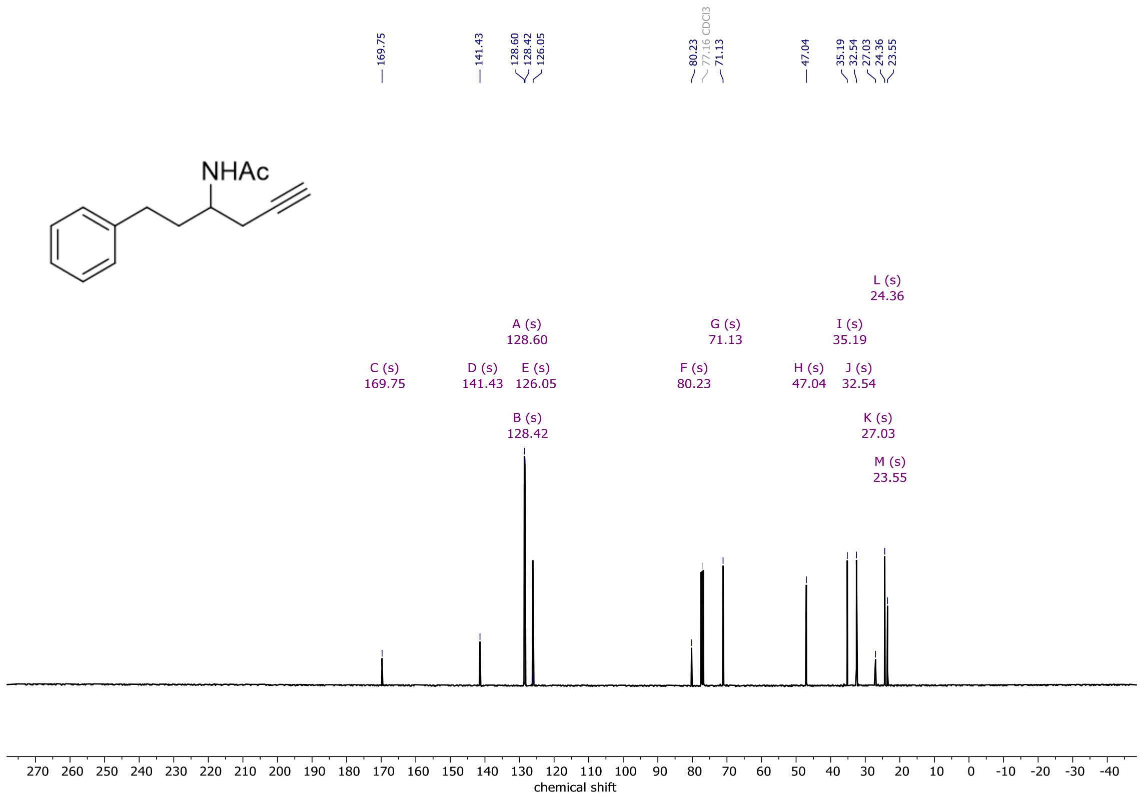
NHAc
C (s)
169.75
D (s)
141.43
A (s)
128.60
E (s)
126.05
B (s)
128.42
F (s)
80.23
G (s)
71.13
H (s)
47.04
I (s)
35.19
J (s)
32.54
K (s)
27.03
L (s)
24.36
M (s)
23.55
chemical shift

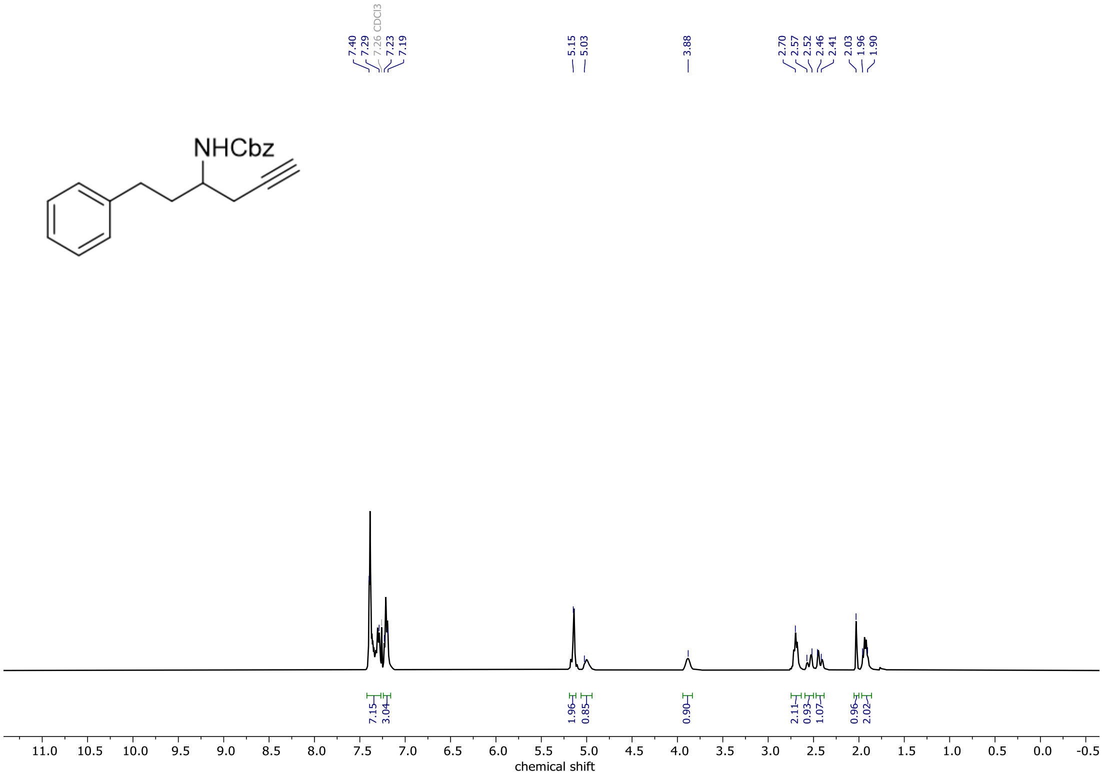
NHCbz
7.40
7.29
7.26 CDCl3
7.23
7.19
5.15
5.03
3.88
2.70
2.57
2.52
2.46
2.41
2.03
1.96
1.90
7.15
3.04
1.96
0.85
0.90
2.11
0.93
1.07
0.96
2.02
11.0 10.5 10.0 9.5 9.0 8.5 8.0 7.5 7.0 6.5 6.0 5.5 5.0 4.5 4.0 3.5 3.0 2.5 2.0 1.5 1.0 0.5 0.0 -0.5
chemical shift

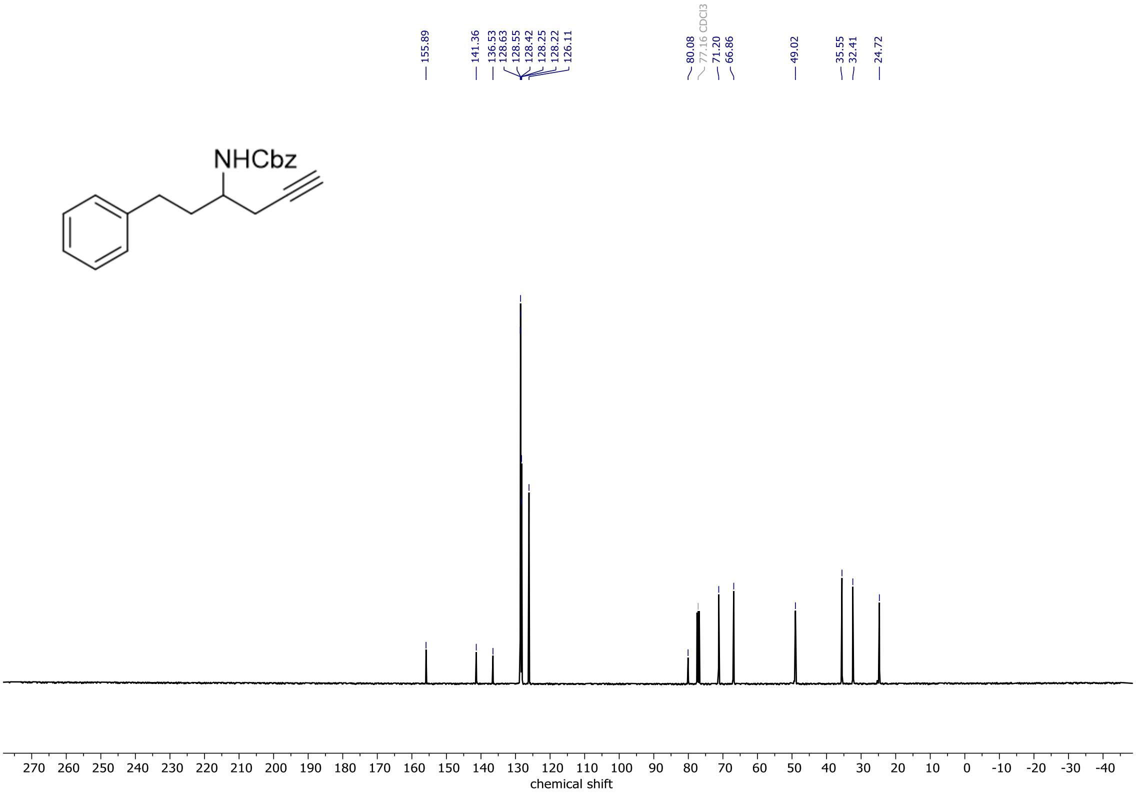
NHCbz
155.89
141.36
136.53
128.63
128.55
128.42
128.25
128.22
126.11
80.08
77.16 CDCl3
71.20
66.86
49.02
35.55
32.41
24.72
270 260 250 240 230 220 210 200 190 180 170 160 150 140 130 120 110 100 90 80 70 60 50 40 30 20 10 0 -10 -20 -30 -40
chemical shift

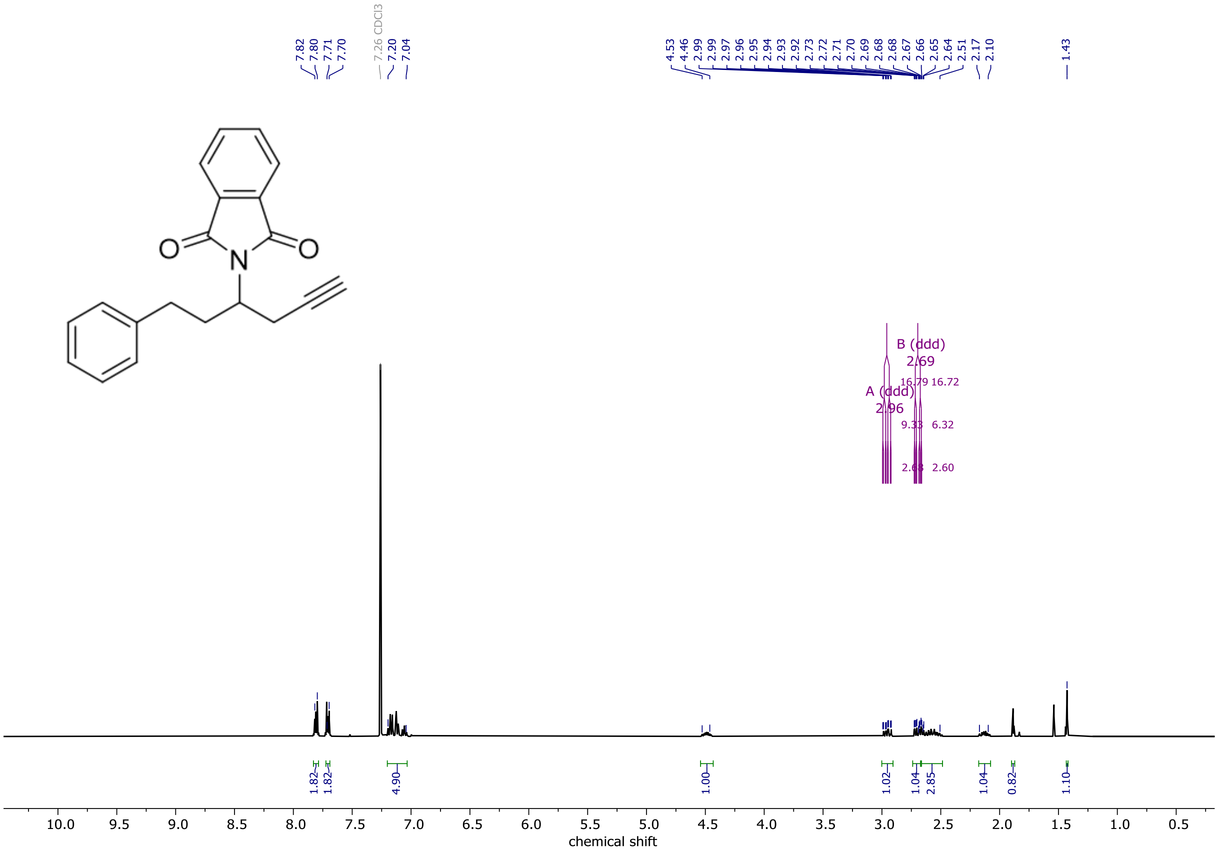

7.82
7.80
7.71
7.70
7.26 CDCl3
7.20
7.04
4.53
4.46
1.43
B (ddd)
2.69
A (ddd)
2.96
1.82
1.82
4.90
1.00
1.02
1.04
2.85
1.04
0.82
1.10
chemical shift

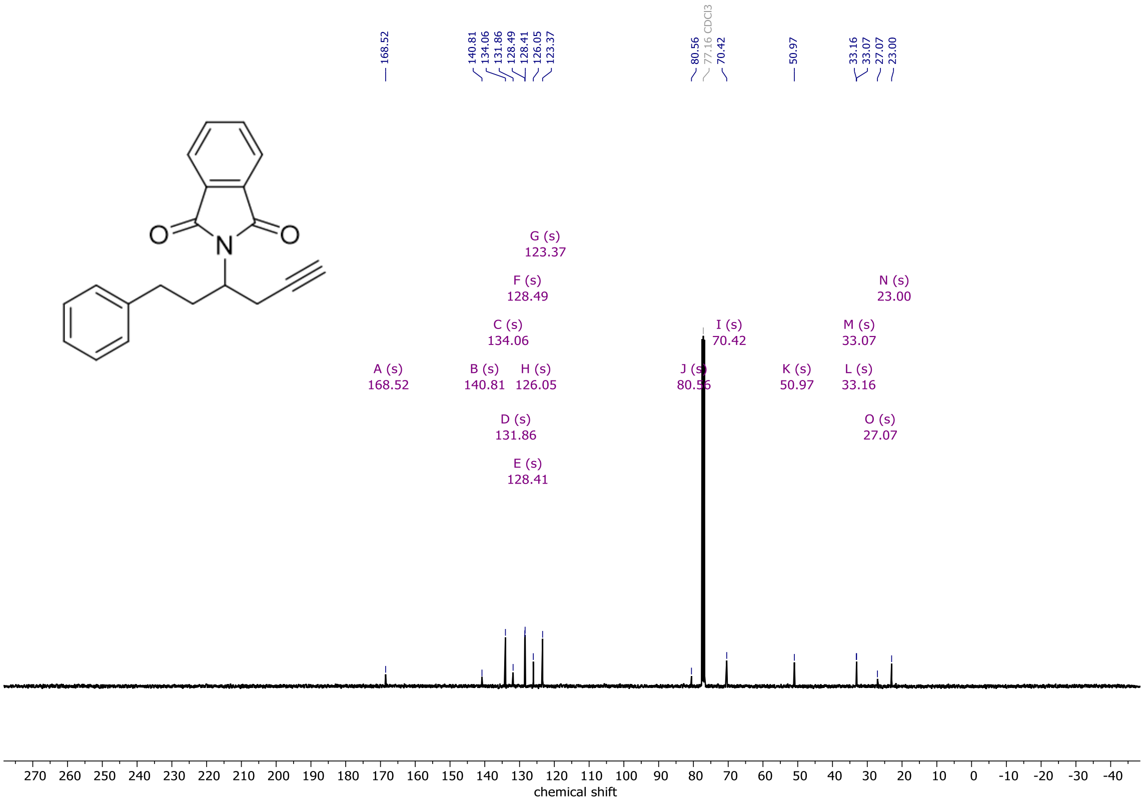

168.52
140.81
134.06
131.86
128.49
128.41
126.05
123.37
80.56
77.16 CDCl3
70.42
50.97
33.16
33.07
27.07
23.00
A (s)
168.52
B (s)
140.81
C (s)
134.06
D (s)
131.86
E (s)
128.41
F (s)
128.49
G (s)
123.37
H (s)
126.05
I (s)
70.42
K (s)
50.97
L (s)
33.16
M (s)
33.07
N (s)
23.00
O (s)
27.07
chemical shift

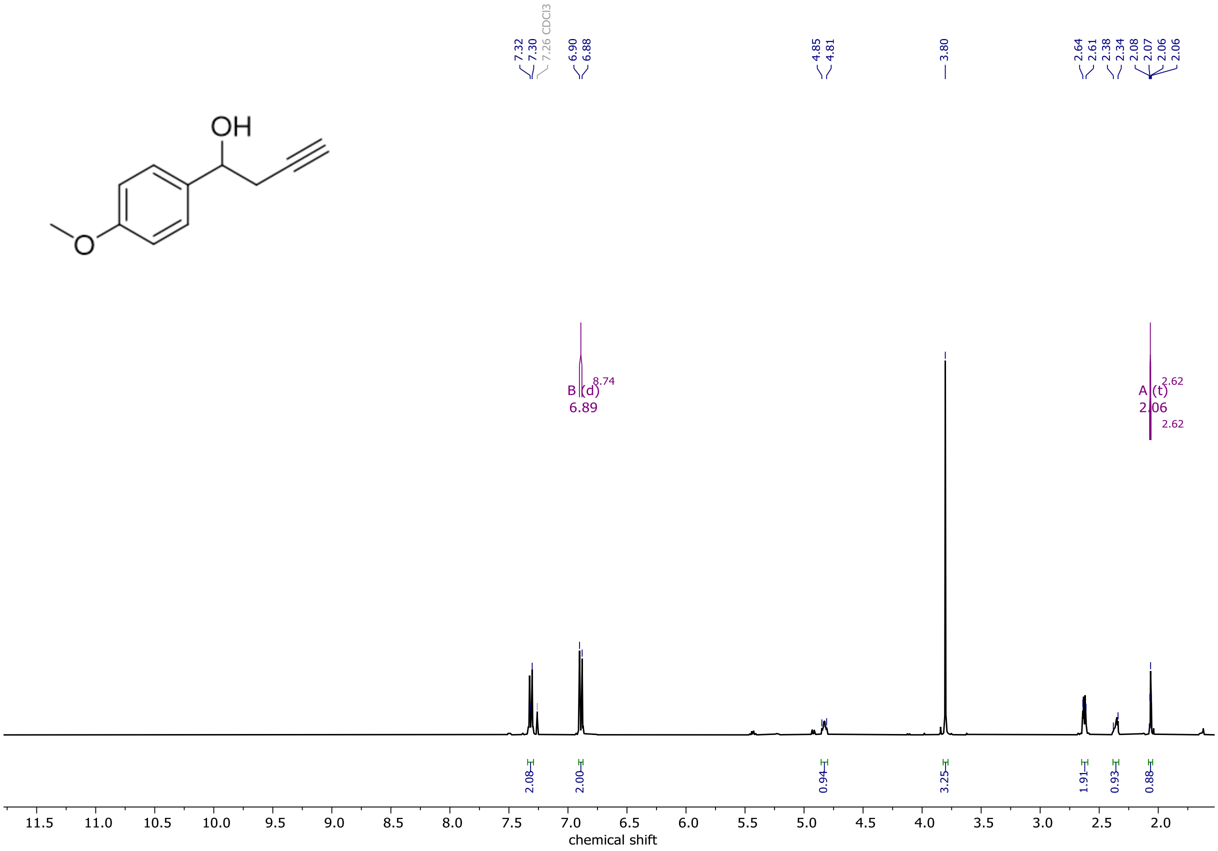
OH
O
7.32
7.30
7.26 CDCl3
6.90
6.88
4.85
4.81
3.80
2.64
2.61
2.38
2.34
2.08
2.07
2.06
2.06
B (d)
6.89
8.74
A (t)
2.06
2.62
2.62
2.08
2.00
0.94
3.25
1.91
0.93
0.88
11.5 11.0 10.5 10.0 9.5 9.0 8.5 8.0 7.5 7.0 6.5 6.0 5.5 5.0 4.5 4.0 3.5 3.0 2.5 2.0
chemical shift

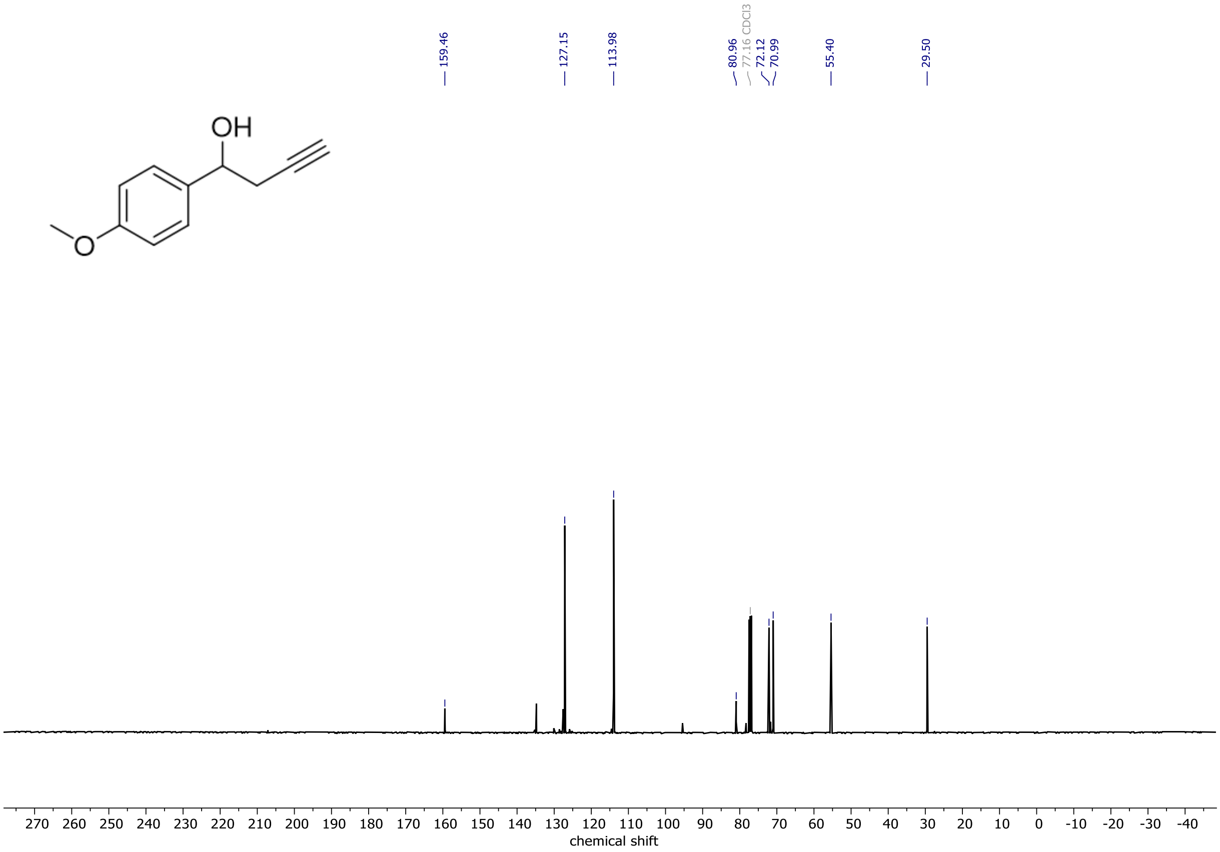
OH
O
159.46
127.15
113.98
80.96
77.16 CDCl3
72.12
70.99
55.40
29.50
270 260 250 240 230 220 210 200 190 180 170 160 150 140 130 120 110 100 90 80 70 60 50 40 30 20 10 0 -10 -20 -30 -40
chemical shift

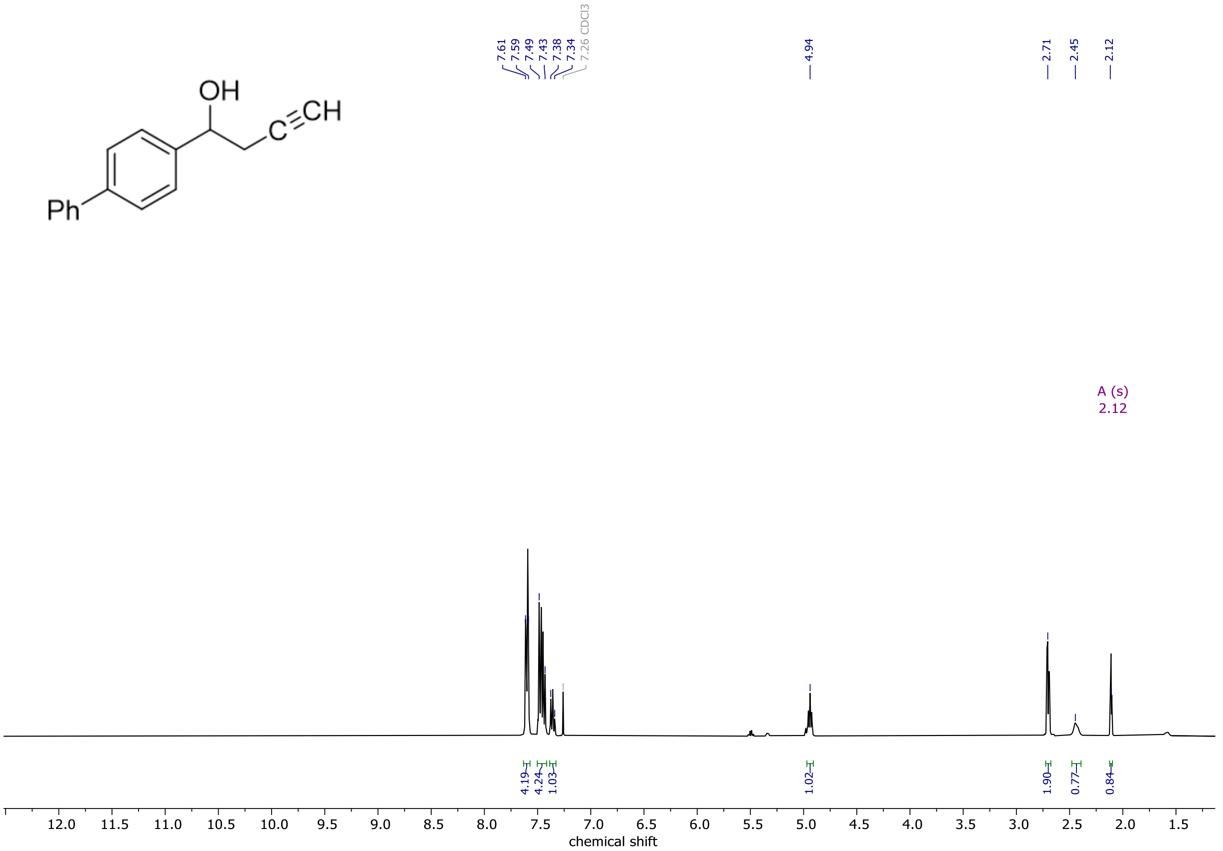
OH
C≡CH
Ph
7.61
7.59
7.49
7.43
7.38
7.34
7.26 CDCl3
4.94
2.71
2.45
2.12
A (s)
2.12
4.19
4.24
1.03
1.02
1.90
0.77
0.84
12.0 11.5 11.0 10.5 10.0 9.5 9.0 8.5 8.0 7.5 7.0 6.5 6.0 5.5 5.0 4.5 4.0 3.5 3.0 2.5 2.0 1.5
chemical shift

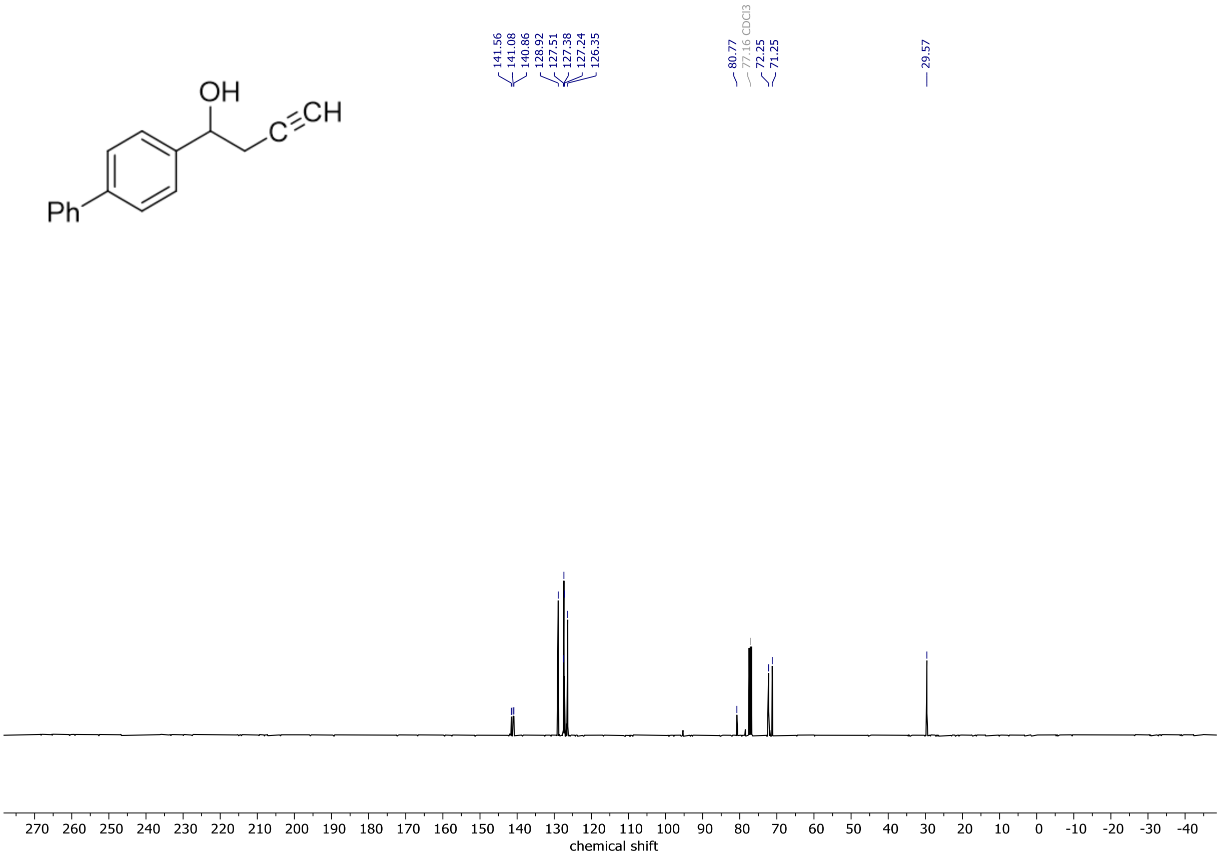
OH
C≡CH
Ph
141.56
141.08
140.86
128.92
127.51
127.38
127.24
126.35
80.77
77.16 CDCl3
72.25
71.25
29.57
270 260 250 240 230 220 210 200 190 180 170 160 150 140 130 120 110 100 90 80 70 60 50 40 30 20 10 0 -10 -20 -30 -40
chemical shift

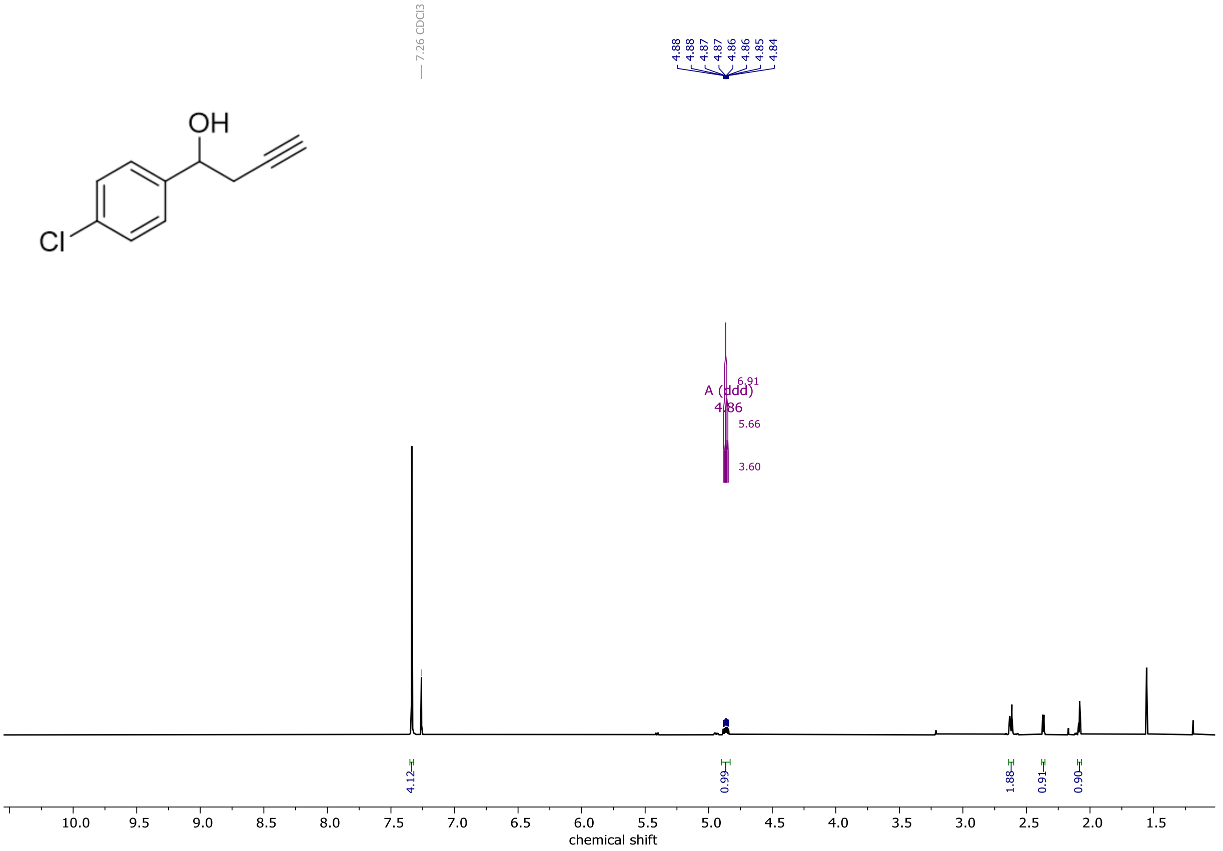
7.26 CDCl3
4.88
4.88
4.87
4.87
4.86
4.86
4.85
4.84
OH
Cl
A (ddd)
4.86
6.91
5.66
3.60
4.12
0.99
1.88
0.91
0.90
10.0 9.5 9.0 8.5 8.0 7.5 7.0 6.5 6.0 5.5 5.0 4.5 4.0 3.5 3.0 2.5 2.0 1.5
chemical shift

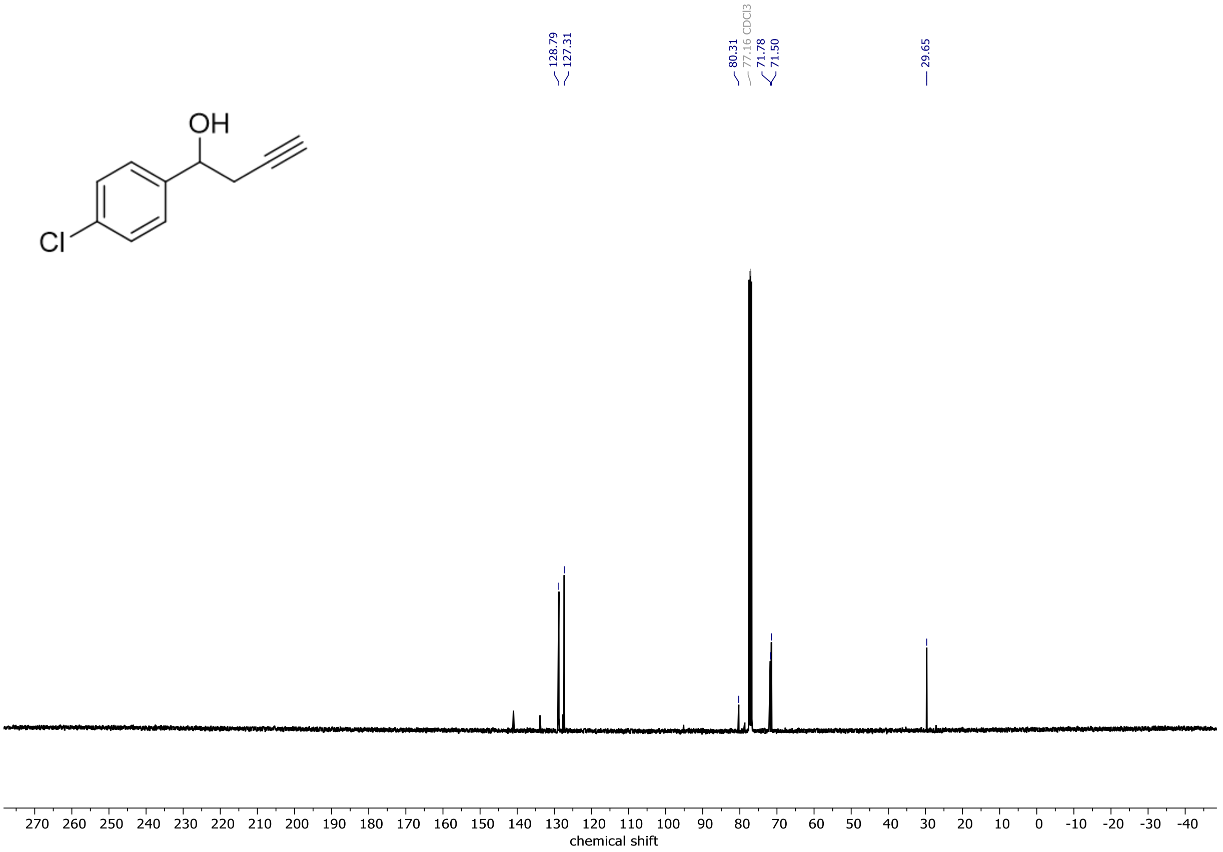
128.79
127.31
80.31
77.16 CDCl3
71.78
71.50
29.65
OH
Cl
270 260 250 240 230 220 210 200 190 180 170 160 150 140 130 120 110 100 90 80 70 60 50 40 30 20 10 0 -10 -20 -30 -40
chemical shift

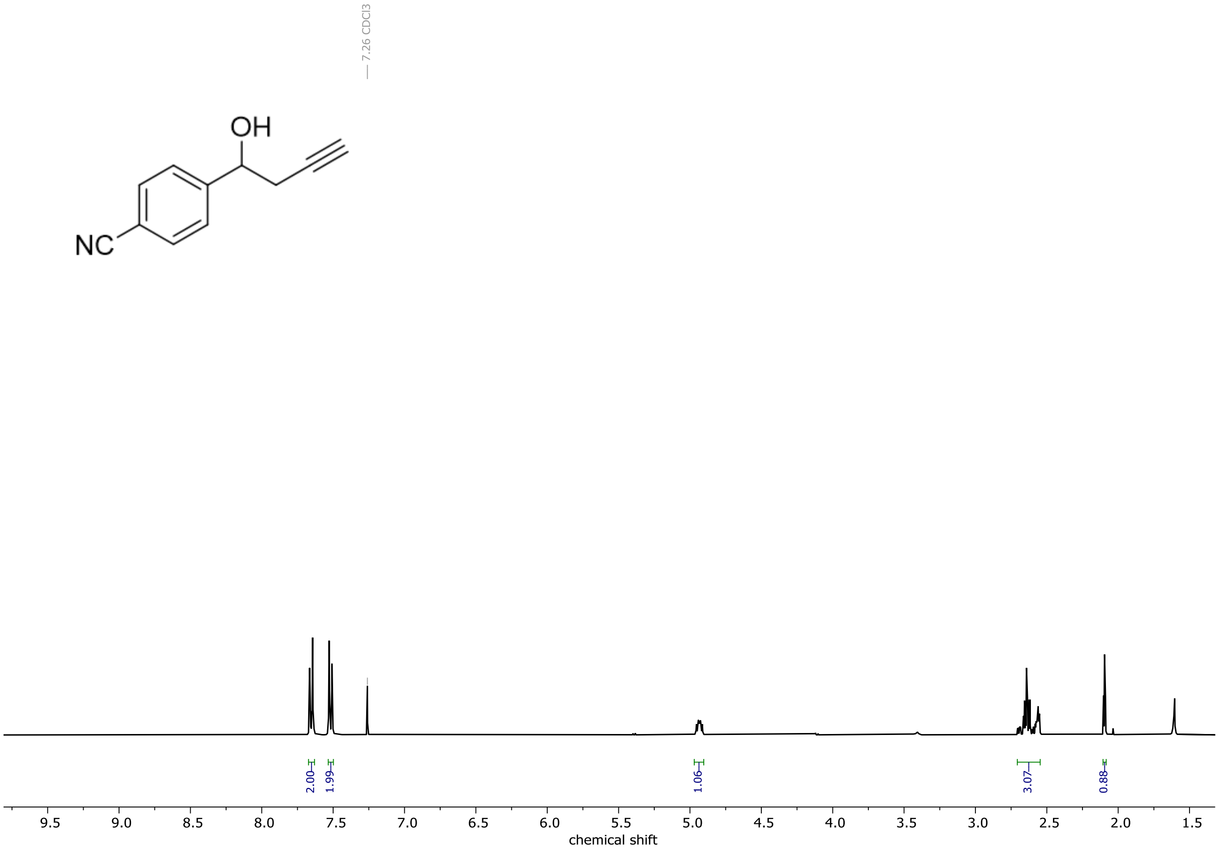
7.26 CDCl3
OH
NC
2.00
1.99
1.06
3.07
0.88
9.5 9.0 8.5 8.0 7.5 7.0 6.5 6.0 5.5 5.0 4.5 4.0 3.5 3.0 2.5 2.0 1.5
chemical shift

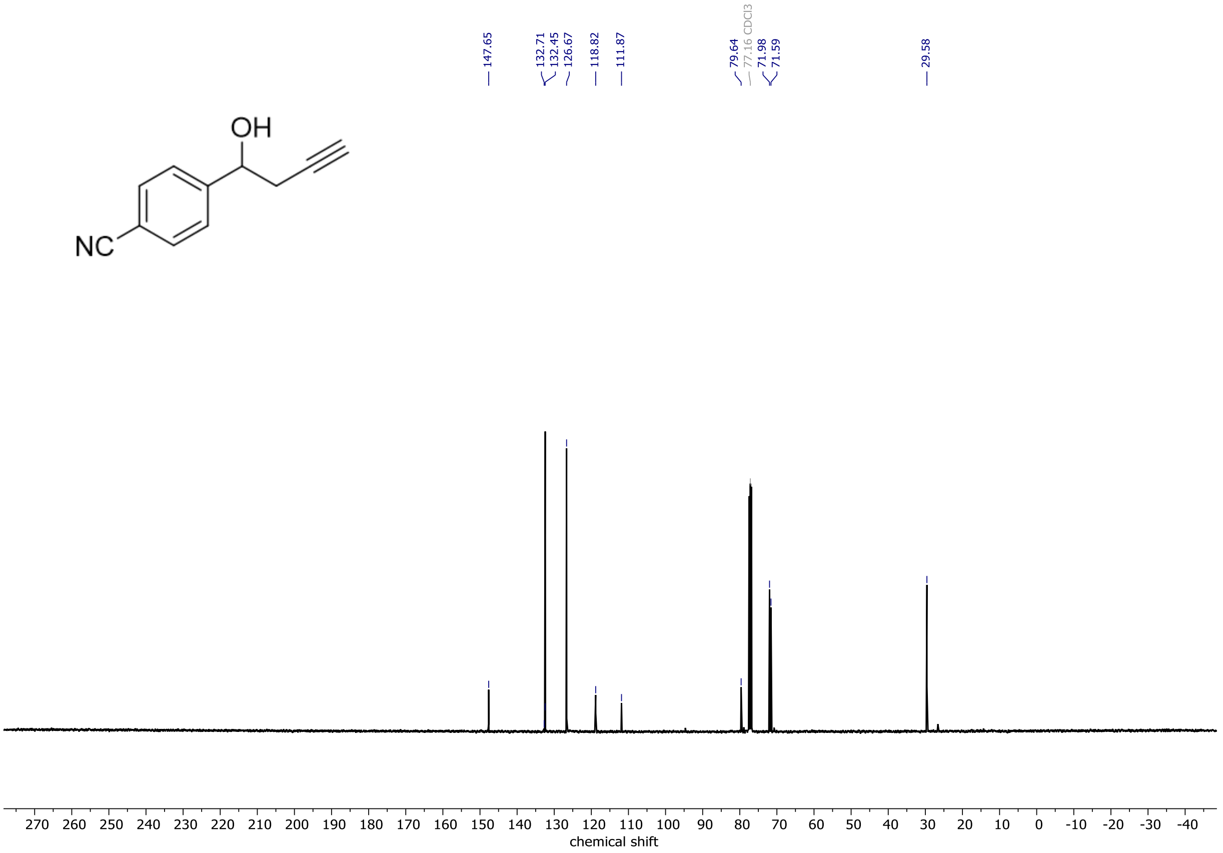
147.65
132.71
132.45
126.67
118.82
111.87
79.64
77.16 CDCl3
71.98
71.59
29.58
OH
NC
270 260 250 240 230 220 210 200 190 180 170 160 150 140 130 120 110 100 90 80 70 60 50 40 30 20 10 0 -10 -20 -30 -40
chemical shift

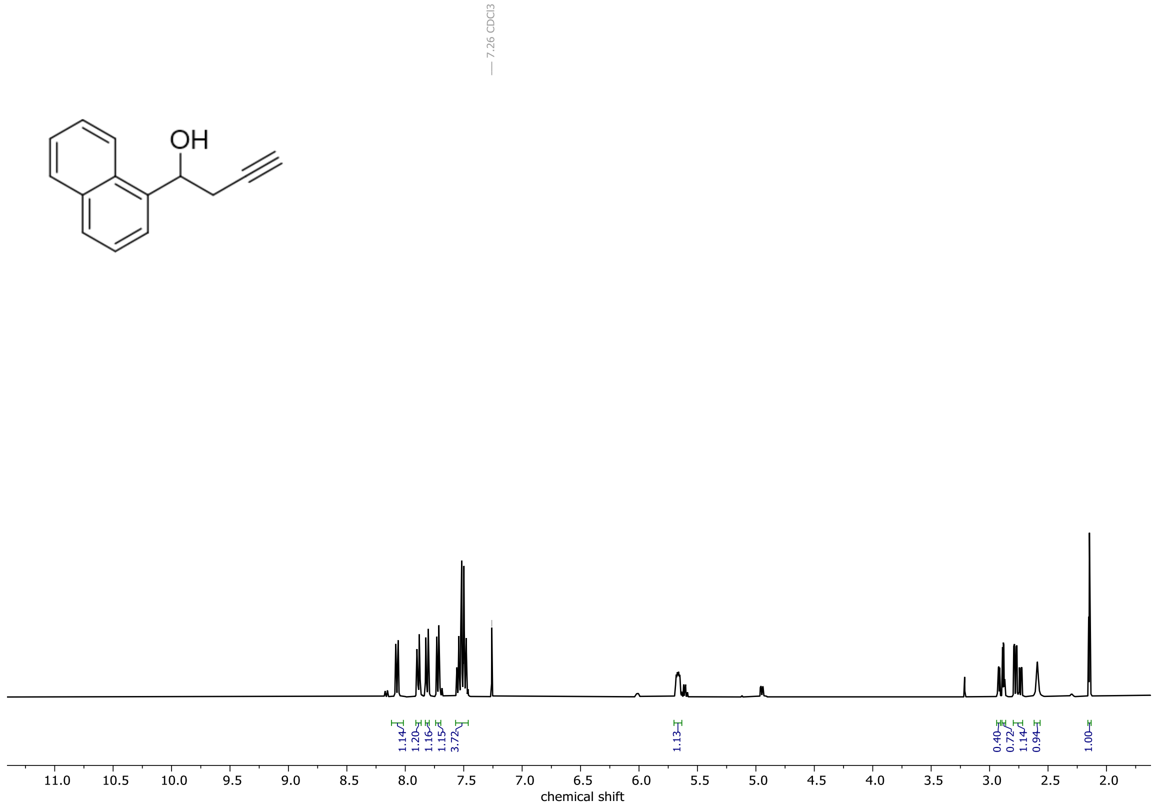
7.26 CDCl3
OH
1.14
1.20
1.16
1.15
3.72
1.13
0.40
0.72
1.14
0.94
1.00
11.0 10.5 10.0 9.5 9.0 8.5 8.0 7.5 7.0 6.5 6.0 5.5 5.0 4.5 4.0 3.5 3.0 2.5 2.0
chemical shift

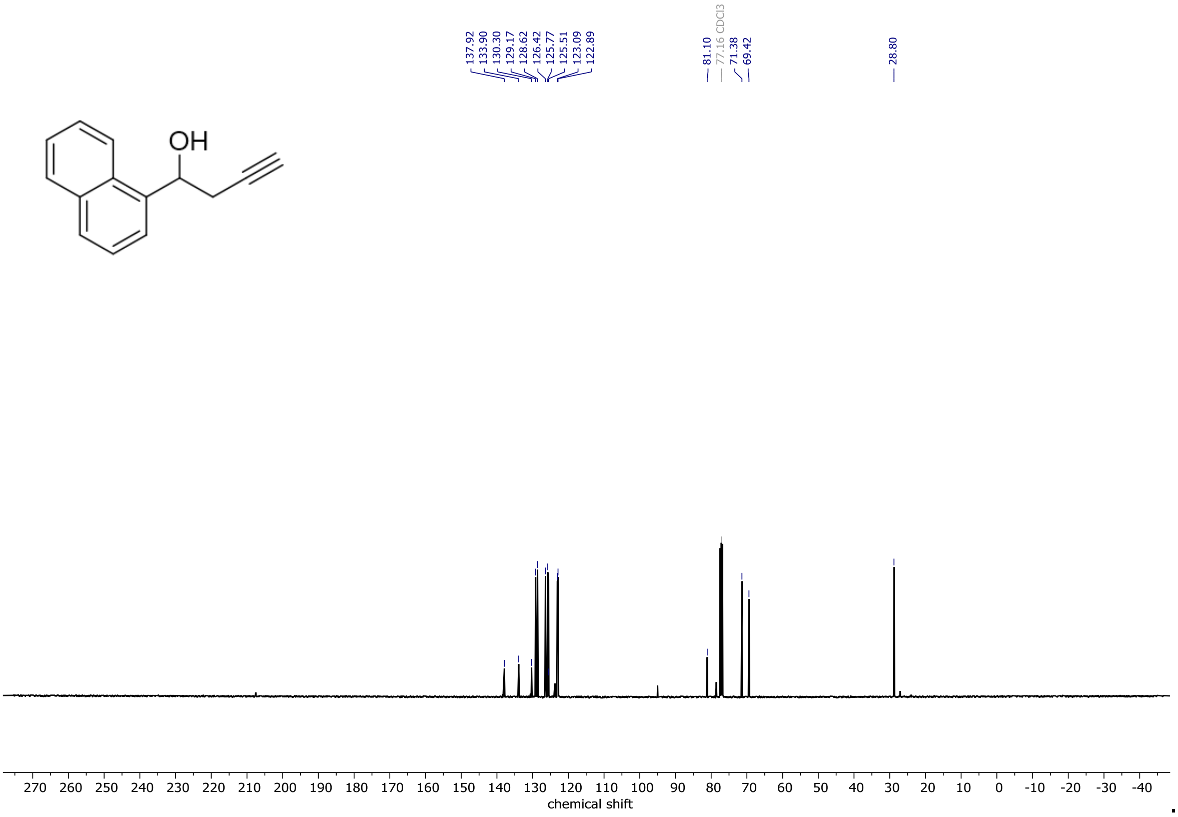
137.92
133.90
130.30
129.17
128.62
126.42
125.77
125.51
123.09
122.89
81.10
77.16 CDCl3
71.38
69.42
28.80
OH
270 260 250 240 230 220 210 200 190 180 170 160 150 140 130 120 110 100 90 80 70 60 50 40 30 20 10 0 -10 -20 -30 -40
chemical shift

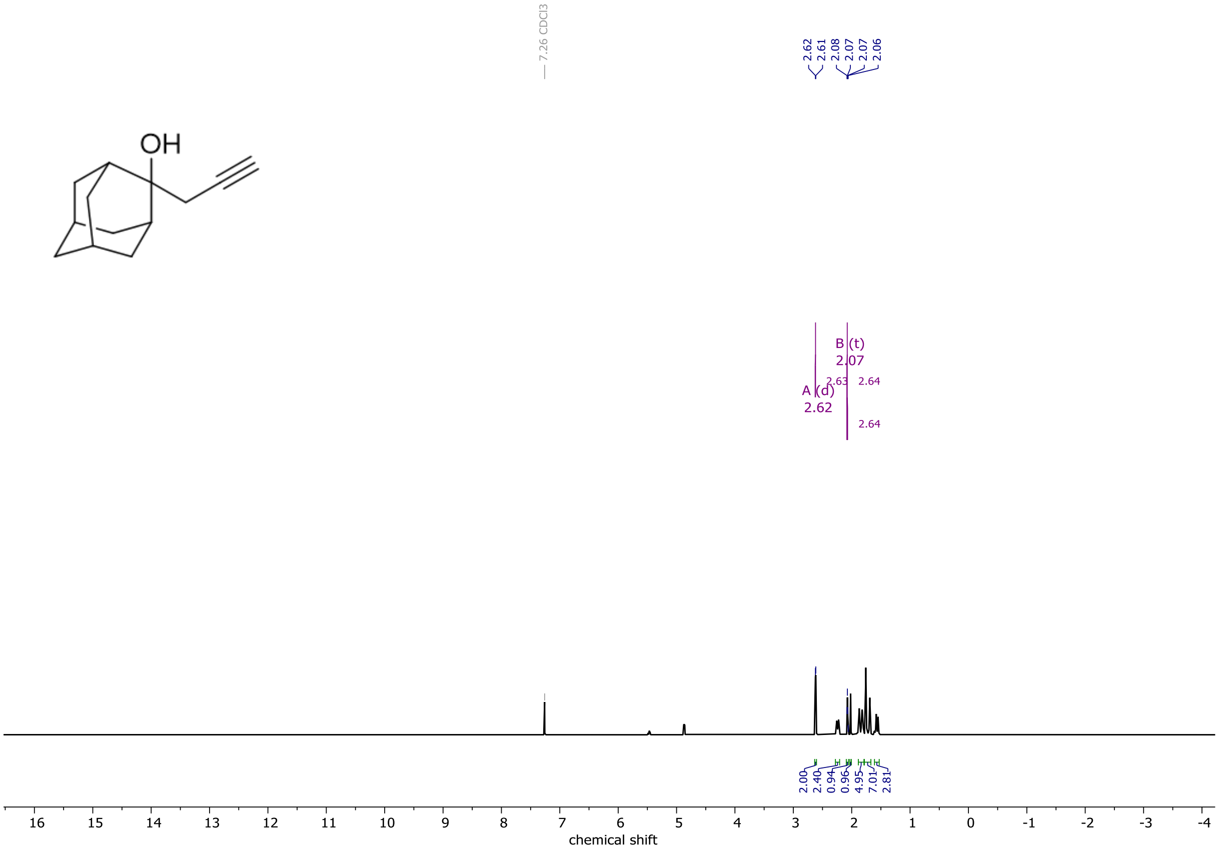
7.26 CDCl3
2.62
2.61
2.08
2.07
2.07
2.06
OH
B (t)
2.07
2.63
2.64
A (d)
2.62
2.64
2.00
2.40
0.94
0.96
4.95
7.01
2.81
16
15
14
13
12
11
10
9
8
7
6
5
4
3
2
1
0
-1
-2
-3
-4
chemical shift

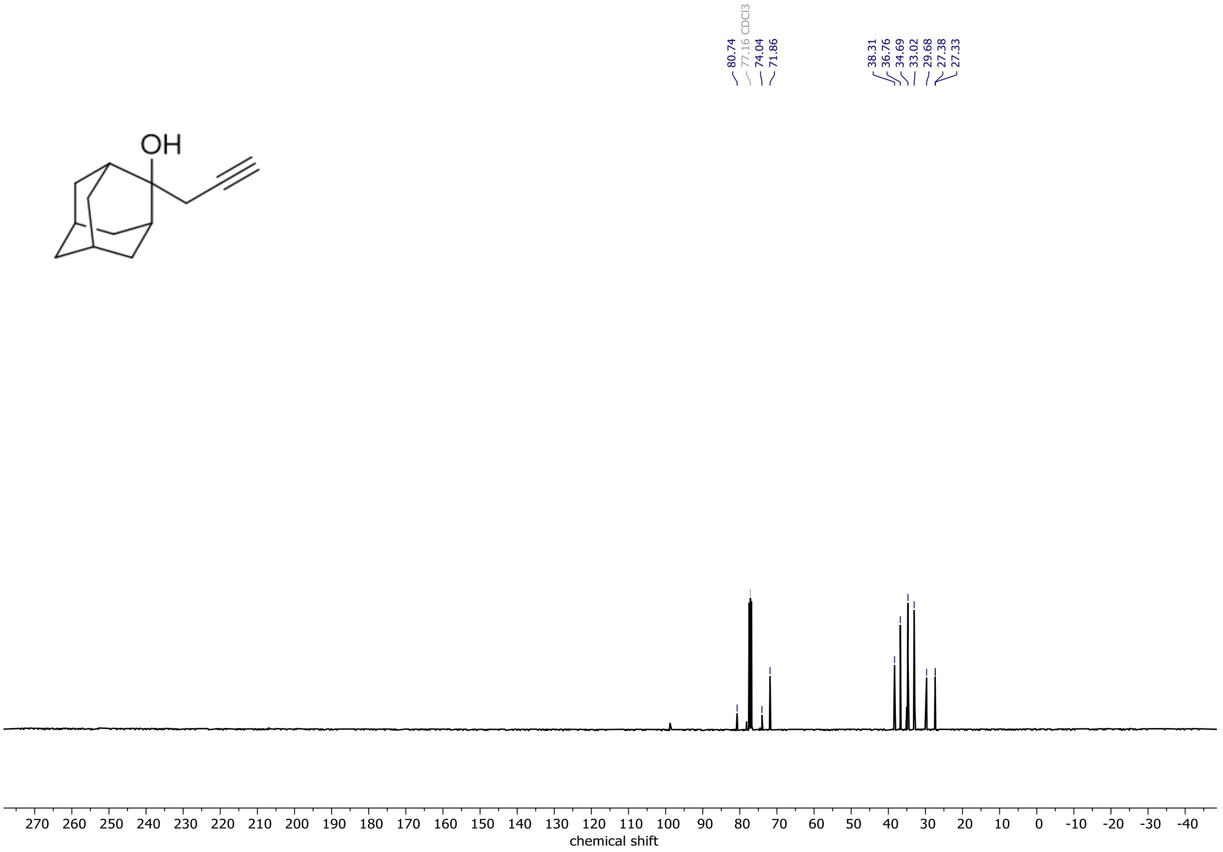
80.74
77.16 CDCl3
74.04
71.86
38.31
36.76
34.69
33.02
29.68
27.38
27.33
OH
270 260 250 240 230 220 210 200 190 180 170 160 150 140 130 120 110 100 90 80 70 60 50 40 30 20 10 0 -10 -20 -30 -40
chemical shift

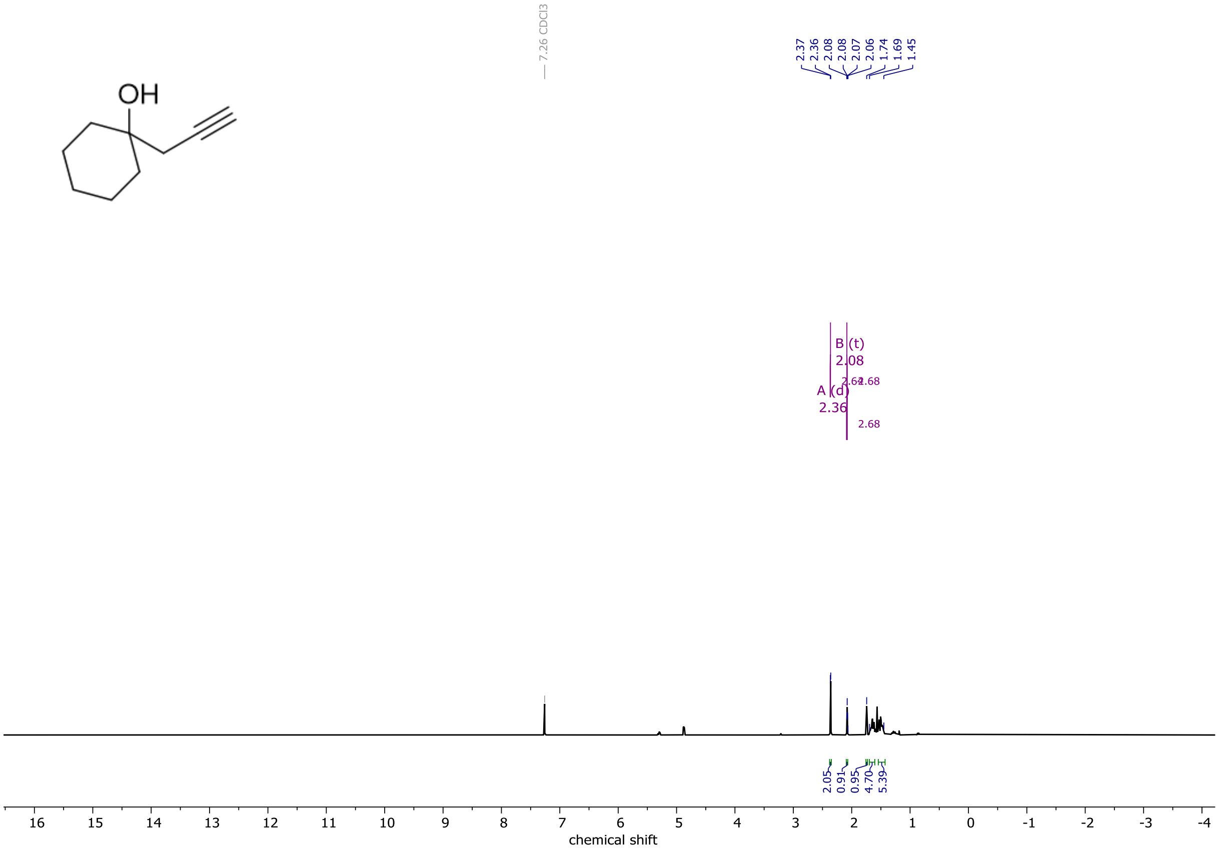
OH
7.26 CDCl3
2.37
2.36
2.08
2.08
2.08
2.07
2.06
1.74
1.69
1.45
B (t)
2.08
A (d)
2.36
2.68
2.05
0.91
0.95
4.70
5.39
16 15 14 13 12 11 10 9 8 7 6 5 4 3 2 1 0 -1 -2 -3 -4
chemical shift

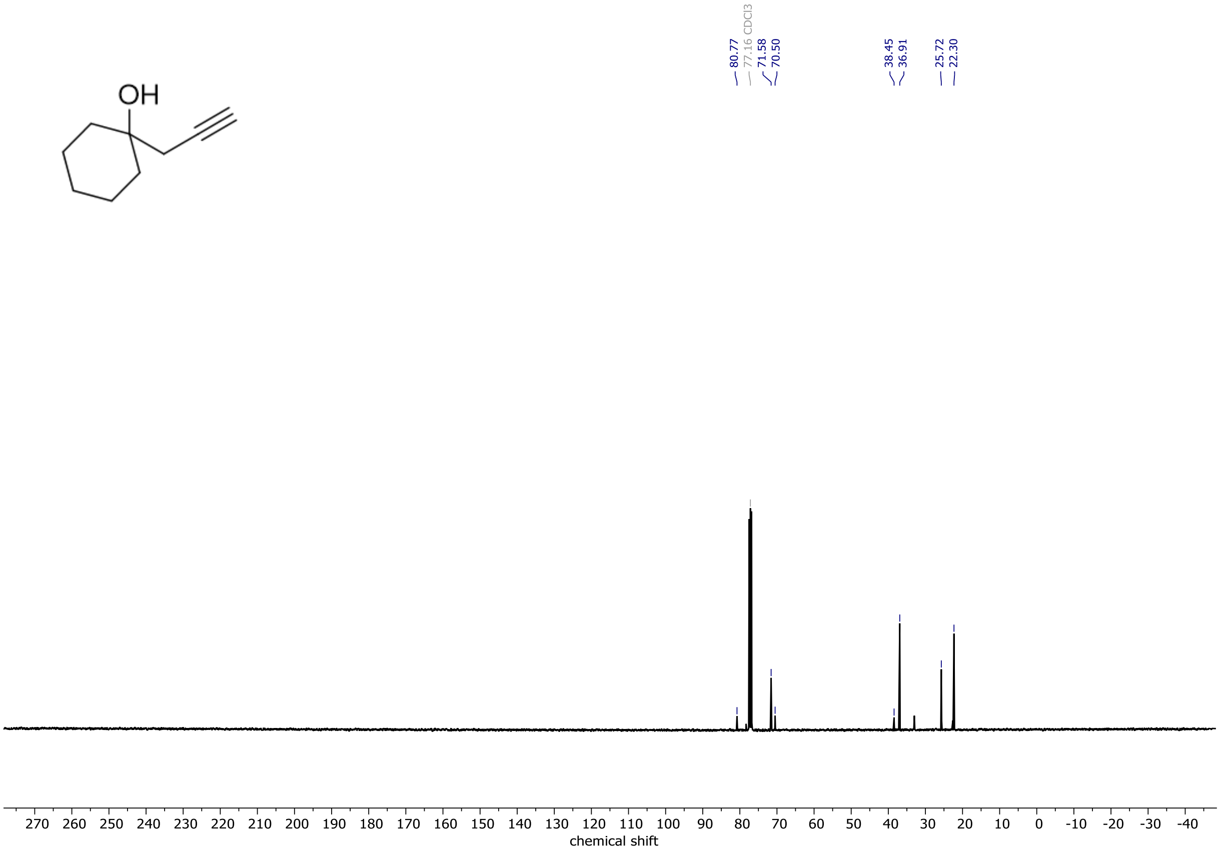
OH
80.77
77.16 CDCl3
71.58
70.50
38.45
36.91
25.72
22.30
270 260 250 240 230 220 210 200 190 180 170 160 150 140 130 120 110 100 90 80 70 60 50 40 30 20 10 0 -10 -20 -30 -40
chemical shift

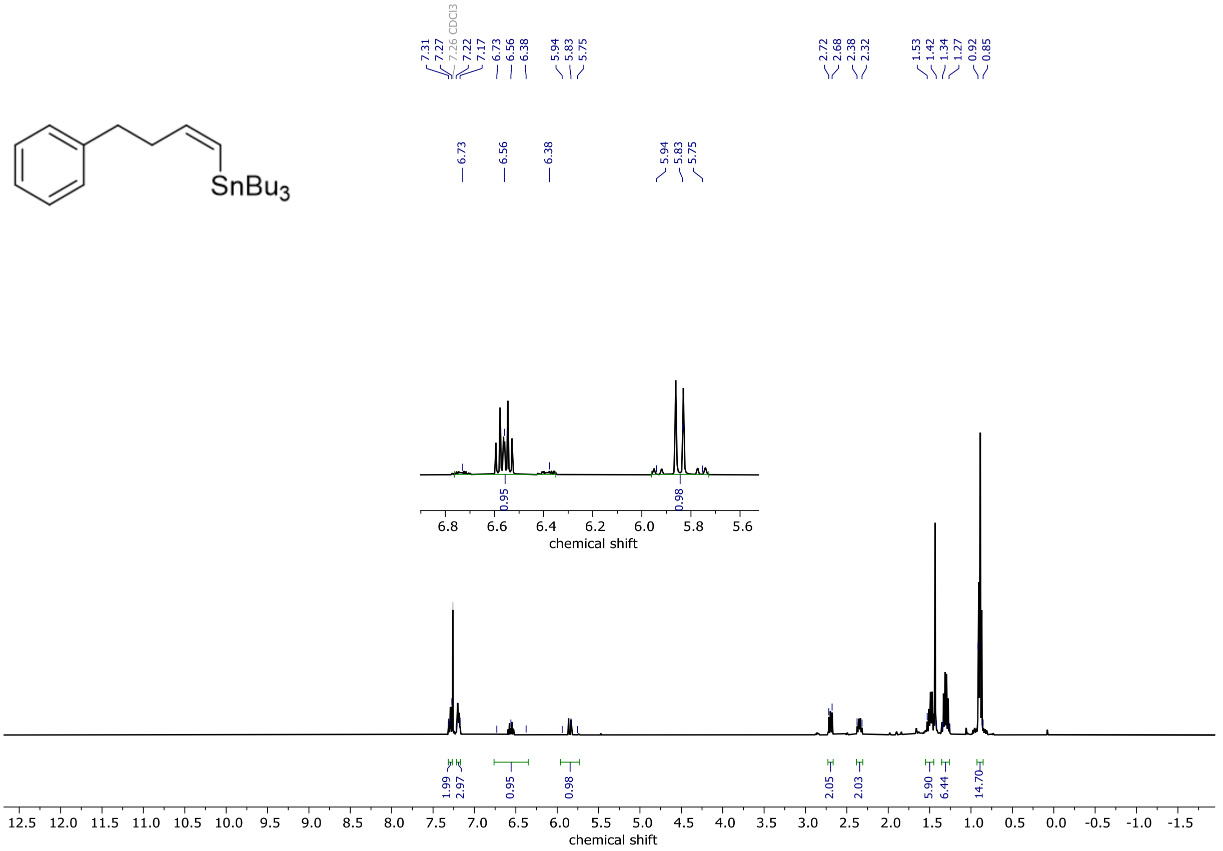
SnBu3
7.31
7.27
7.26 CDCl3
7.22
7.17
6.73
6.56
6.38
5.94
5.83
5.75
2.72
2.68
2.38
2.32
1.53
1.42
1.34
1.27
0.92
0.85
0.95
0.98
1.99
2.97
2.05
2.03
5.90
6.44
14.70
chemical shift

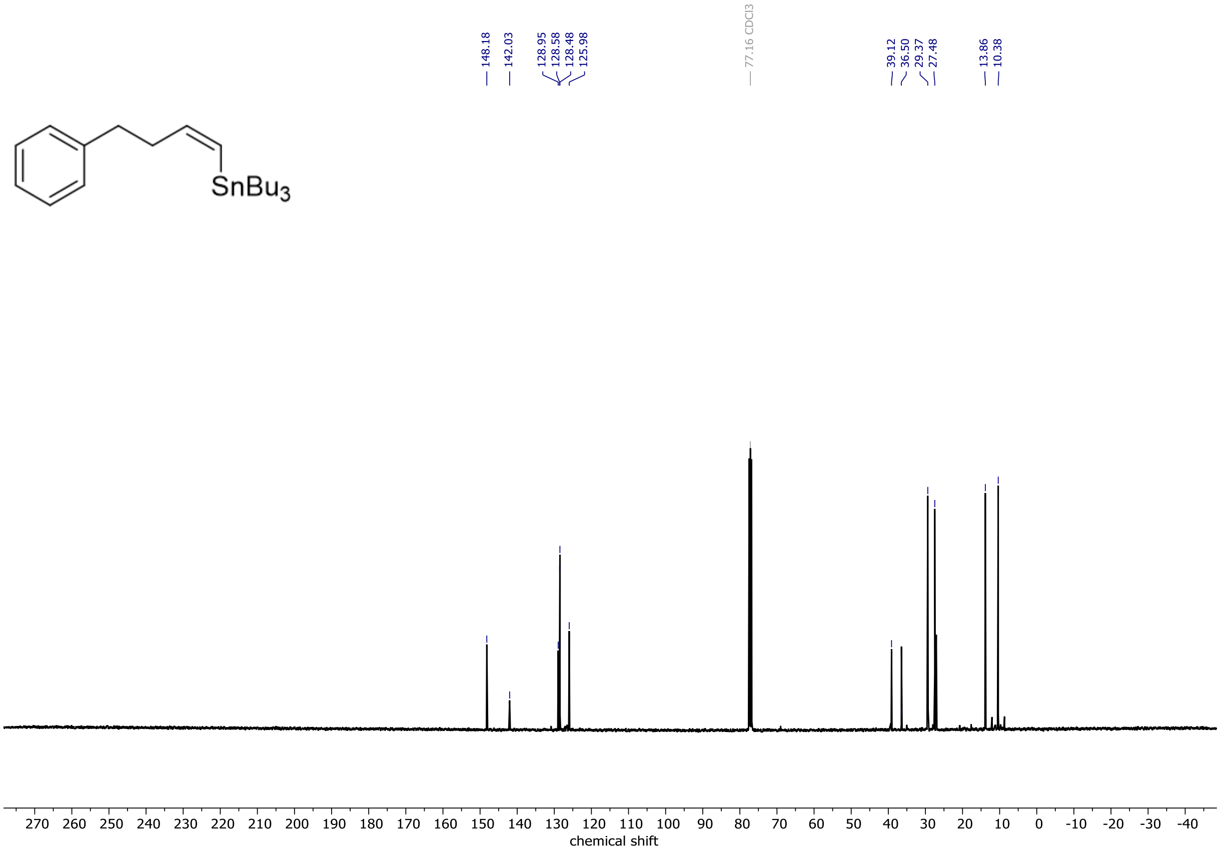
SnBu3
148.18
142.03
128.95
128.58
128.48
125.98
77.16 CDCl3
39.12
36.50
29.37
27.48
13.86
10.38
chemical shift

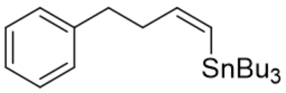
SnBu3

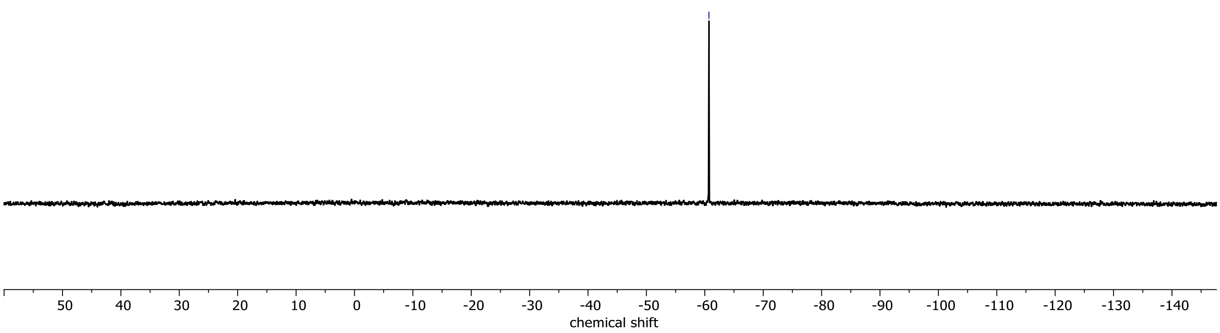
-60.71
50
40
30
20
10
0
-10
-20
-30
-40
-50
-60
-70
-80
-90
-100
-110
-120
-130
-140
chemical shift

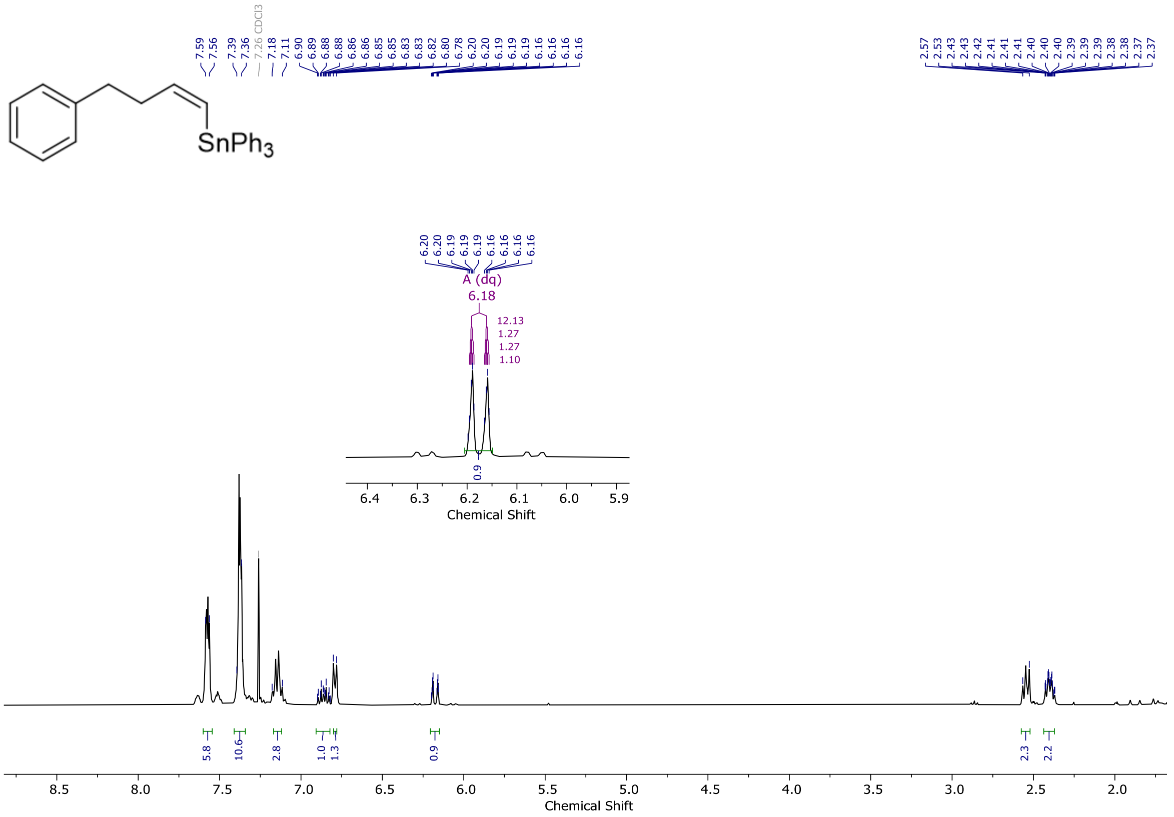
SnPh3
7.59
7.56
7.39
7.36
7.26 CDCl3
7.18
7.11
6.90 6.89 6.88 6.88 6.86 6.86 6.85 6.83 6.83 6.82 6.80 6.78 6.20 6.20 6.19 6.19 6.19 6.16 6.16 6.16
2.57 2.53 2.43 2.43 2.42 2.41 2.41 2.40 2.40 2.39 2.39 2.38 2.37 2.37
6.20 6.20 6.19 6.19 6.19 6.16 6.16 6.16
A (dq)
6.18
12.13
1.27
1.27
1.10
0.9
Chemical Shift
5.8
10.6
2.8
1.0
1.3
0.9
2.3
2.2
Chemical Shift

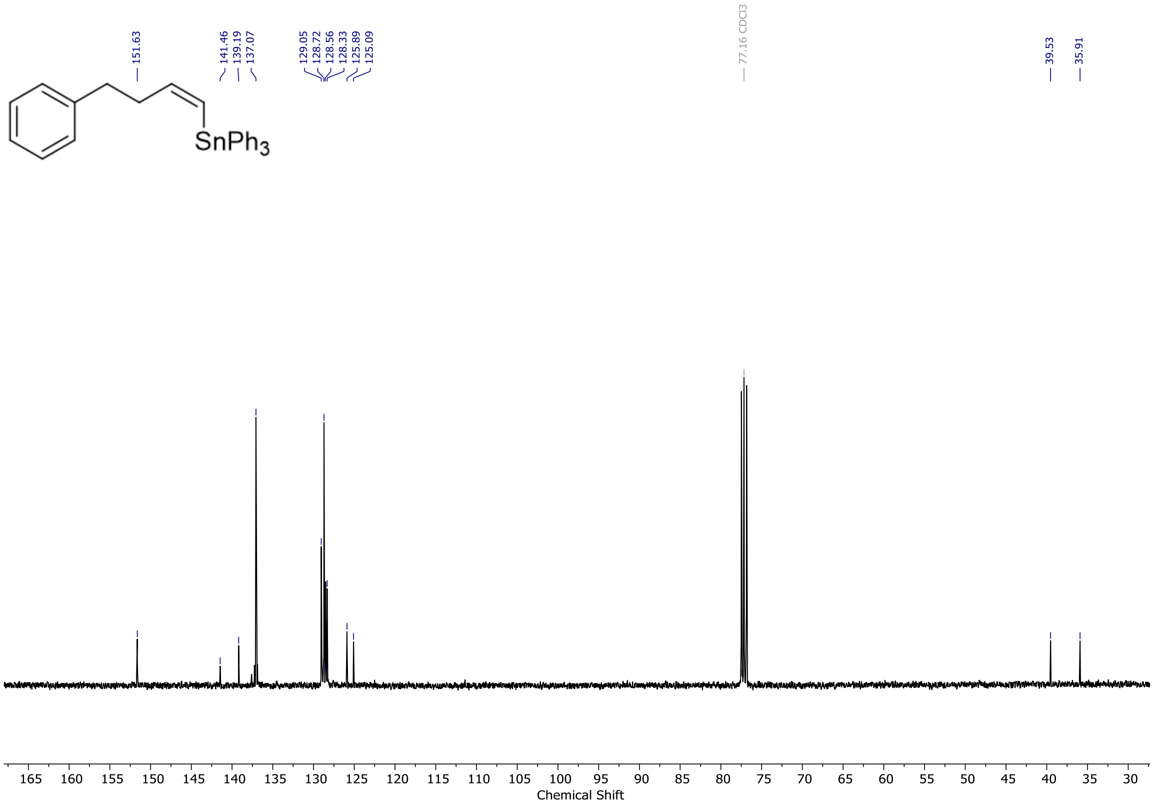
SnPh3
151.63
141.46
139.19
137.07
129.05
128.72
128.56
128.33
125.89
125.09
77.16 CDCl3
39.53
35.91
Chemical Shift

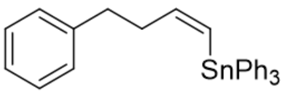

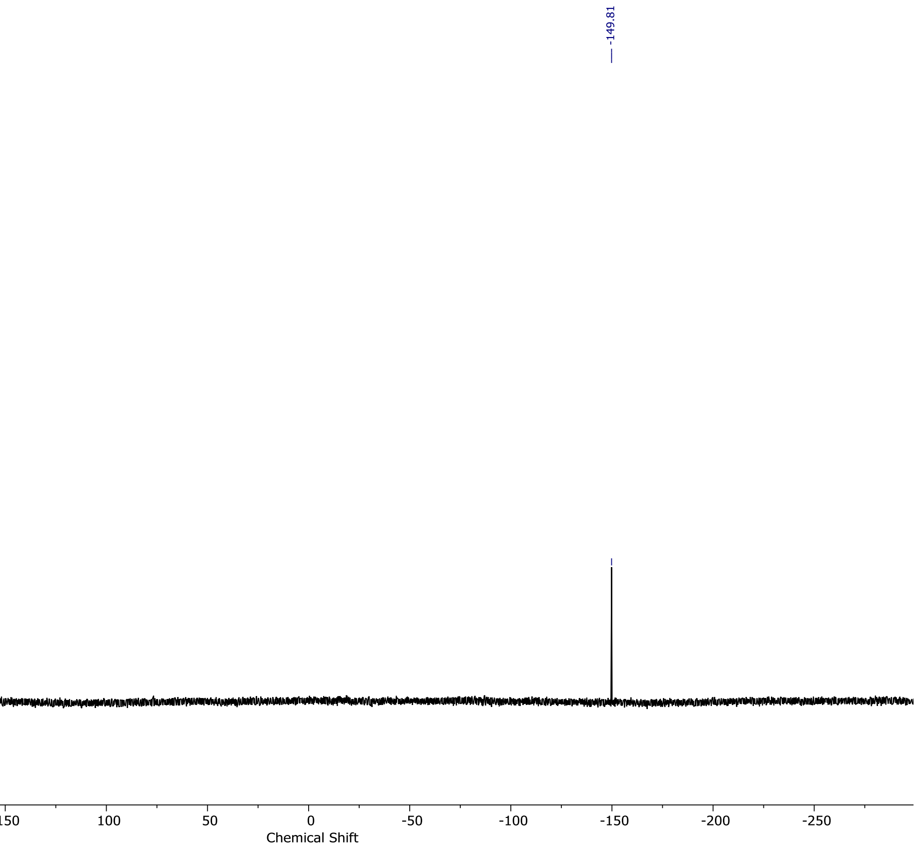

-149.81
250
200
150
100
50
0
-50
-100
-150
-200
-250
Chemical Shift

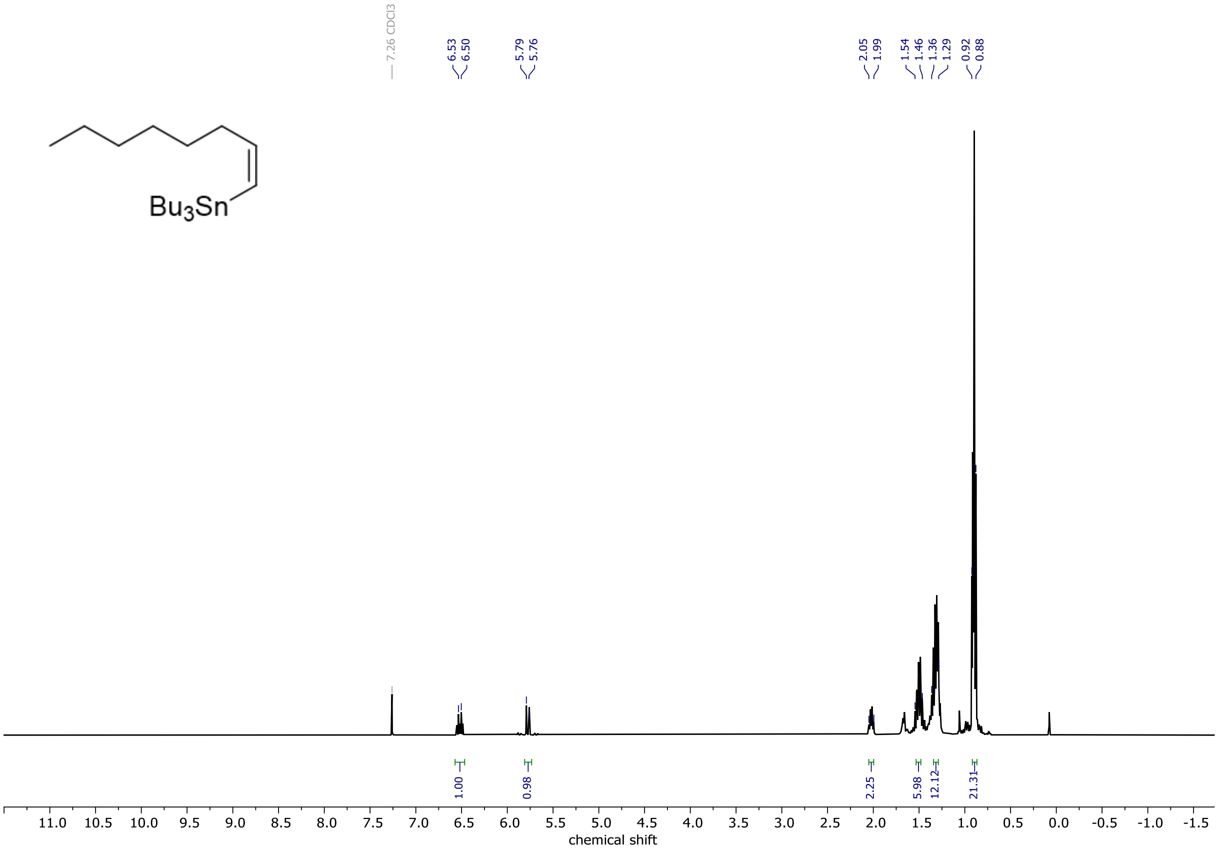
7.26 CDCl3
6.53
6.50
5.79
5.76
2.05
1.99
1.54
1.46
1.36
1.29
0.92
0.88
Bu3Sn
1.00
0.98
2.25
5.98
12.12
21.31
chemical shift

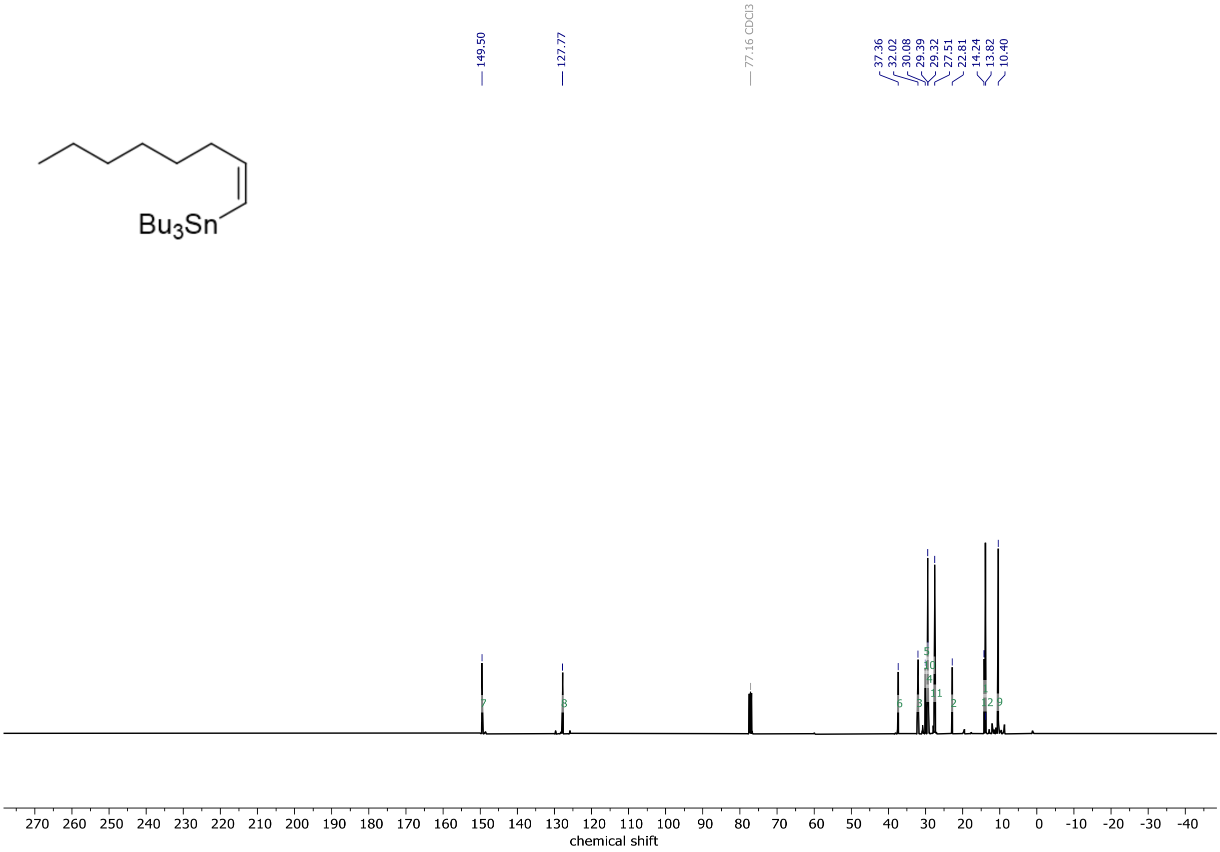
149.50
127.77
77.16 CDCl3
37.36
32.02
30.08
29.39
29.32
27.51
22.81
14.24
13.82
10.40
Bu3Sn
chemical shift

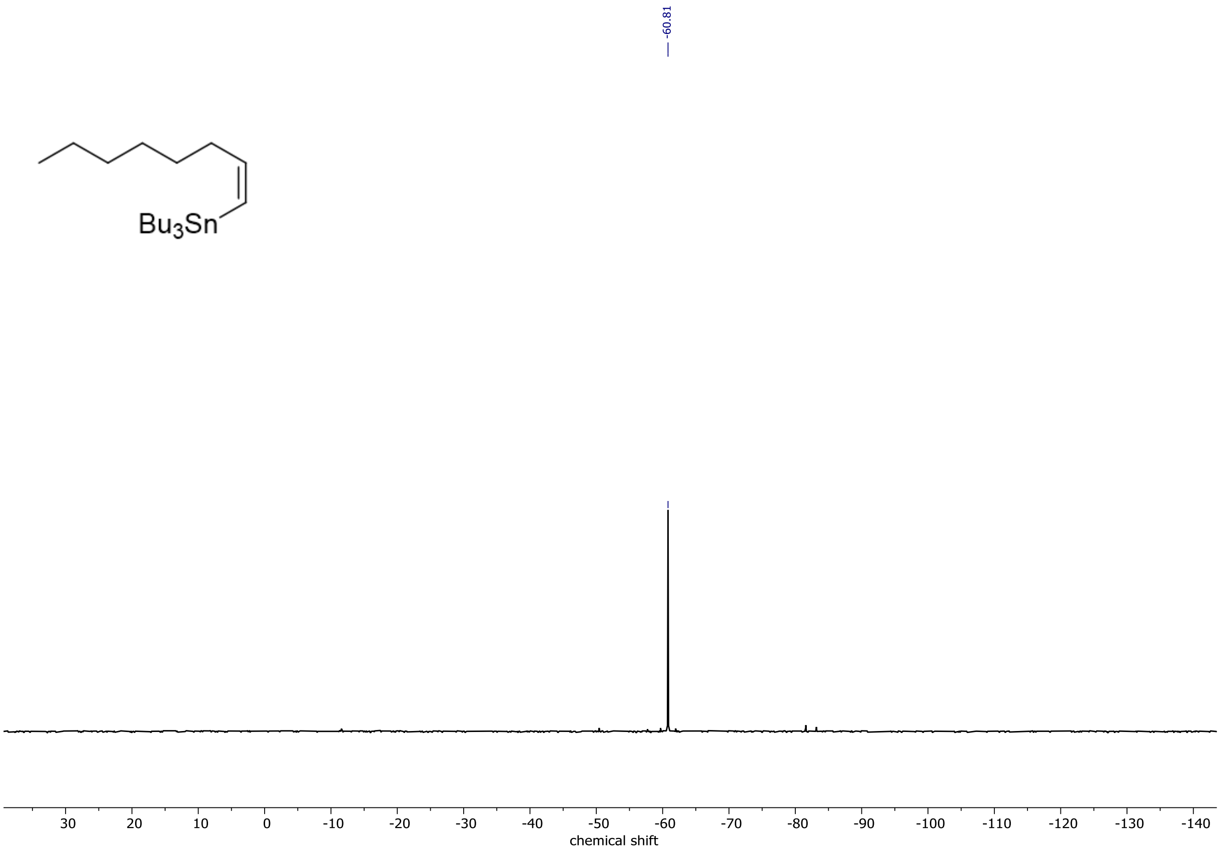
-60.81
Bu3Sn
30
20
10
0
-10
-20
-30
-40
-50
-60
-70
-80
-90
-100
-110
-120
-130
-140
chemical shift

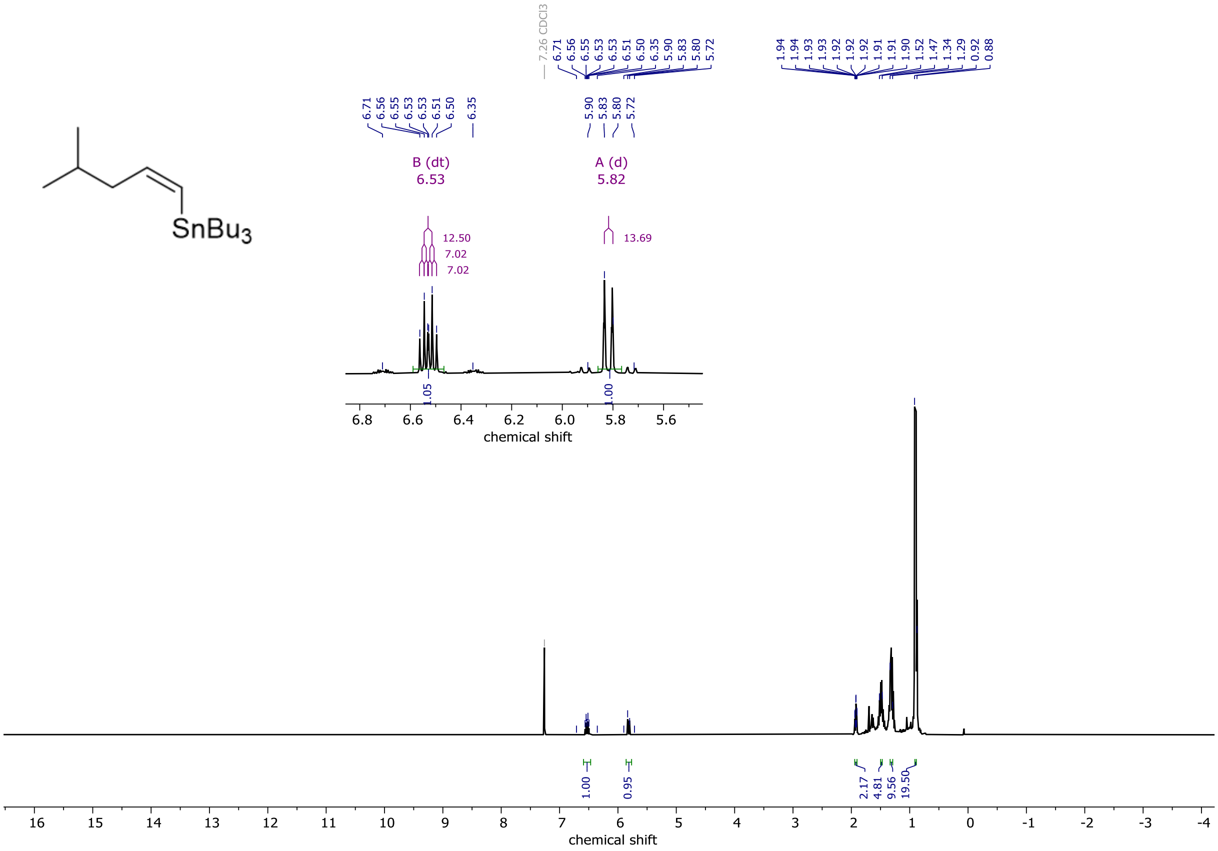

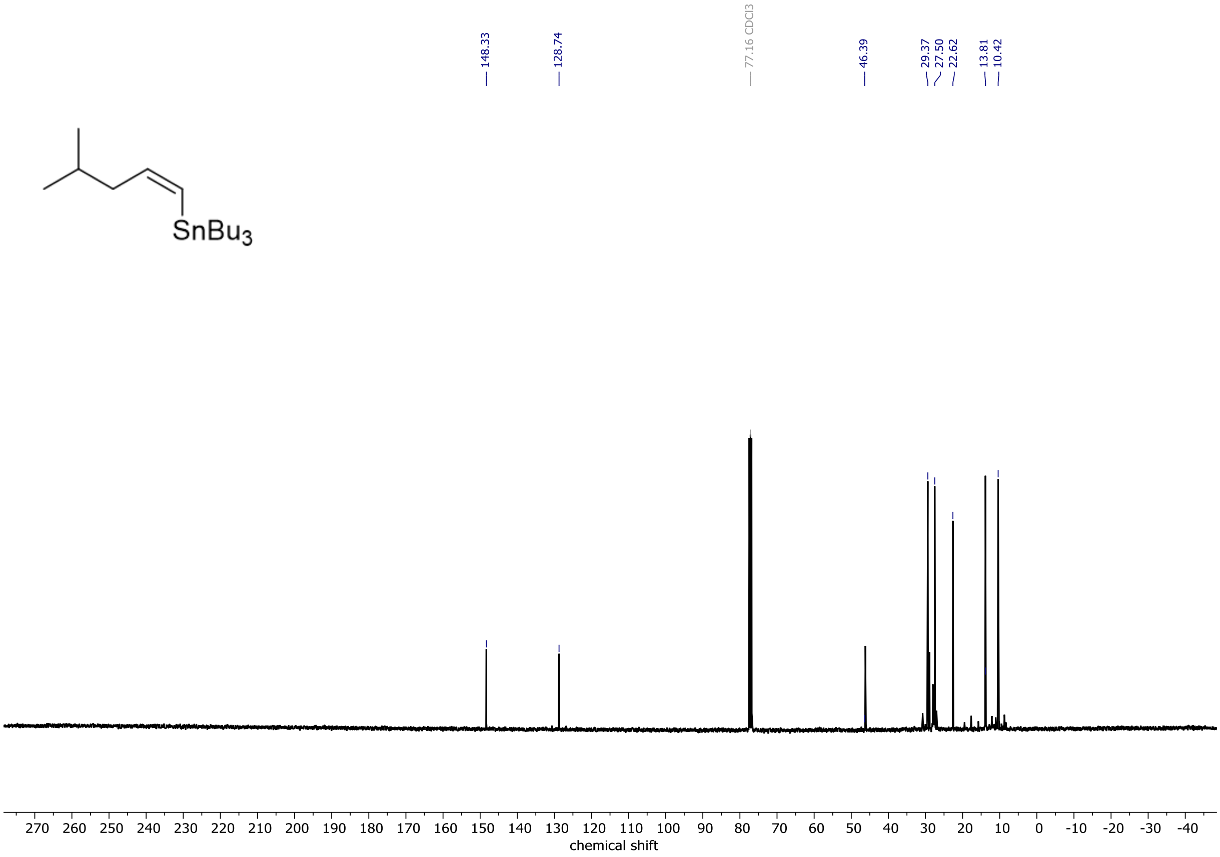

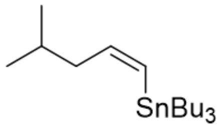

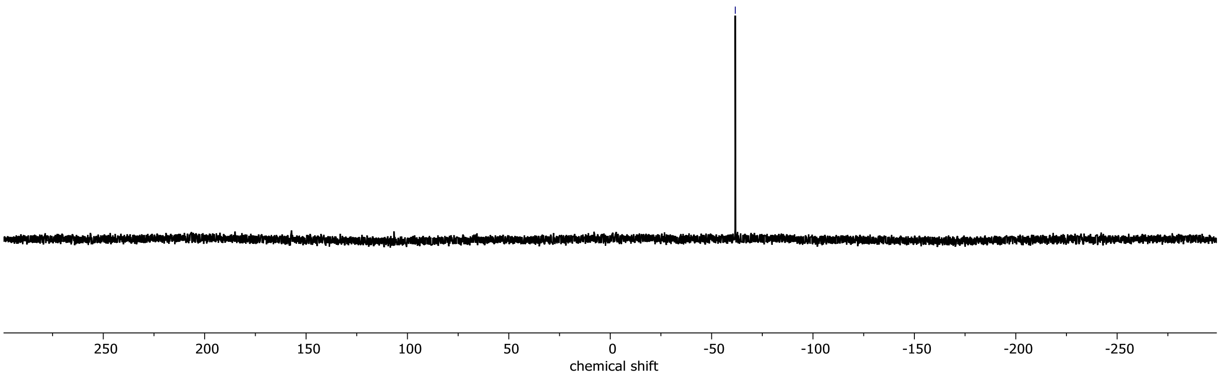

-61.66
250
200
150
100
50
0
-50
-100
-150
-200
-250
chemical shift

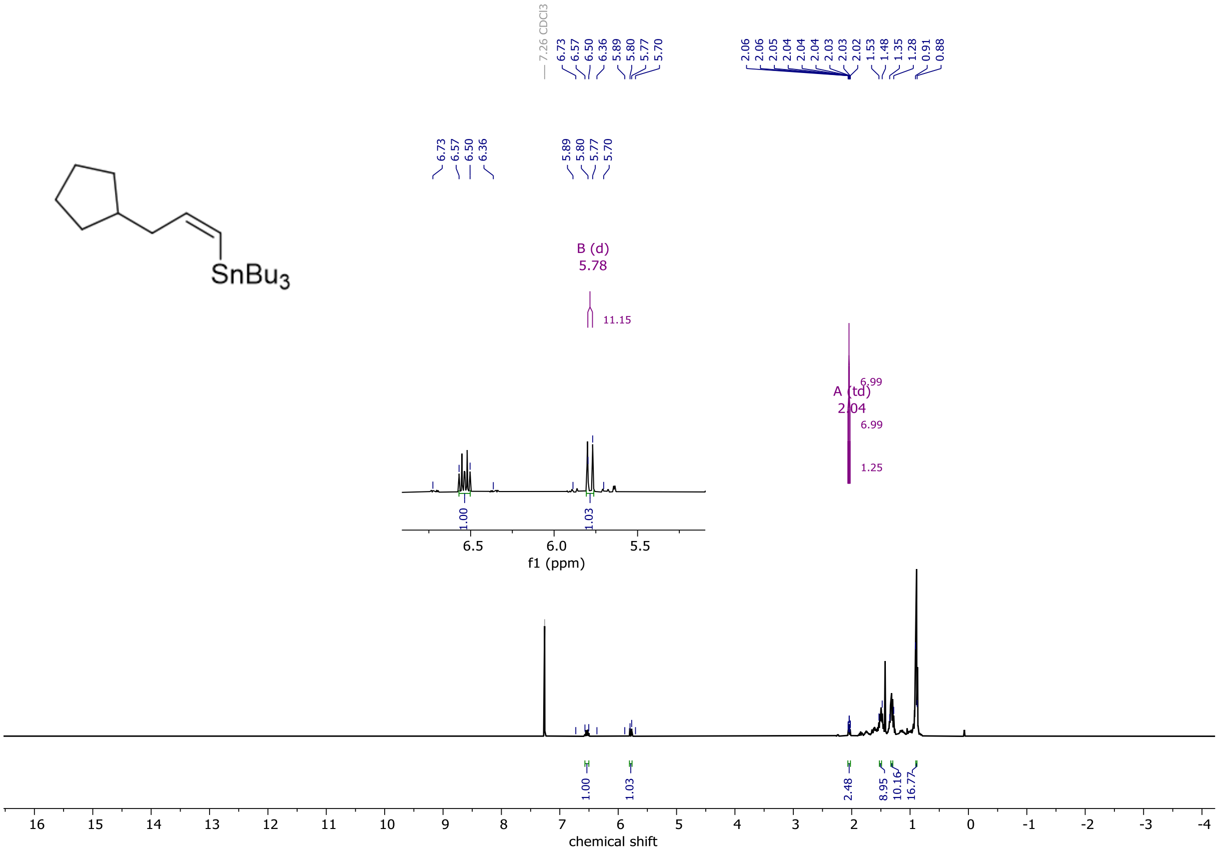
7.26 CDCl3
6.73
6.57
6.50
6.36
5.89
5.80
5.77
5.70
2.06
2.06
2.05
2.04
2.04
2.04
2.03
2.03
2.02
1.53
1.48
1.35
1.28
0.91
0.88
SnBu3
B (d)
5.78
11.15
A (td)
2.04
6.99
6.99
1.25
1.00
1.03
6.5
6.0
5.5
f1 (ppm)
2.48
8.95
10.16
16.77
chemical shift

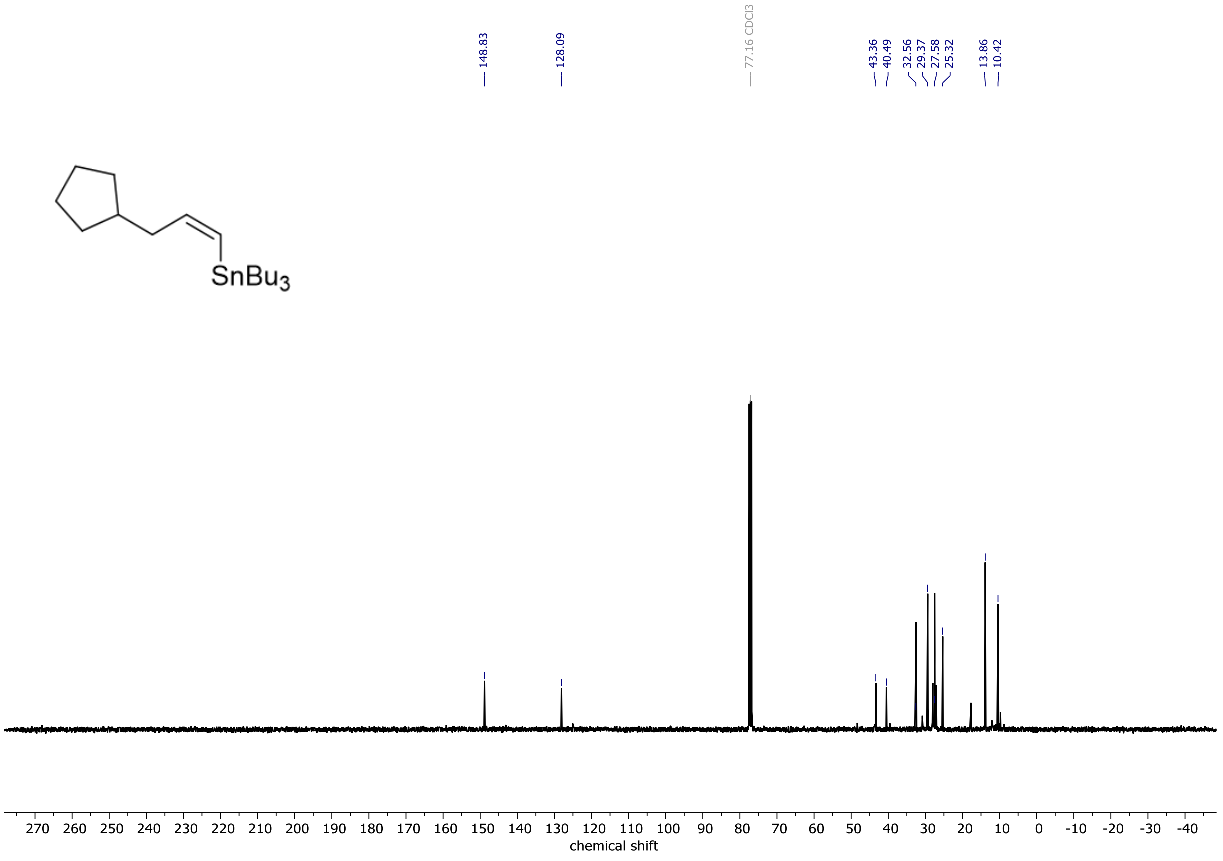
148.83
128.09
77.16 CDCl3
43.36
40.49
32.56
29.37
27.58
25.32
13.86
10.42
SnBu3
chemical shift

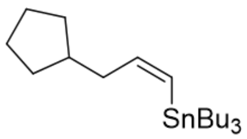
SnBu3

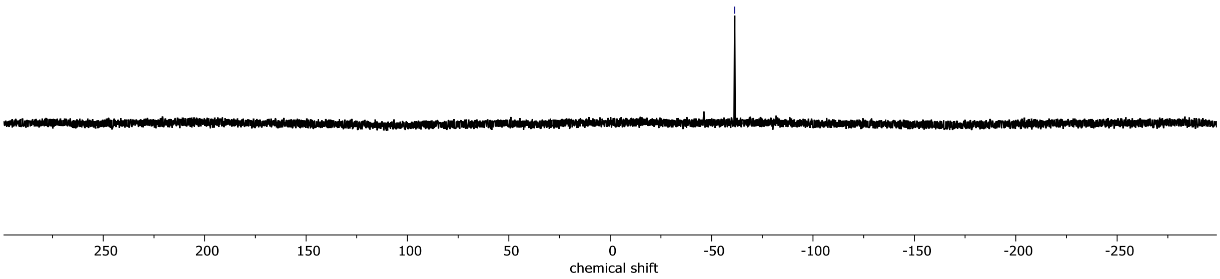
-61.38
250
200
150
100
50
0
-50
-100
-150
-200
-250
chemical shift

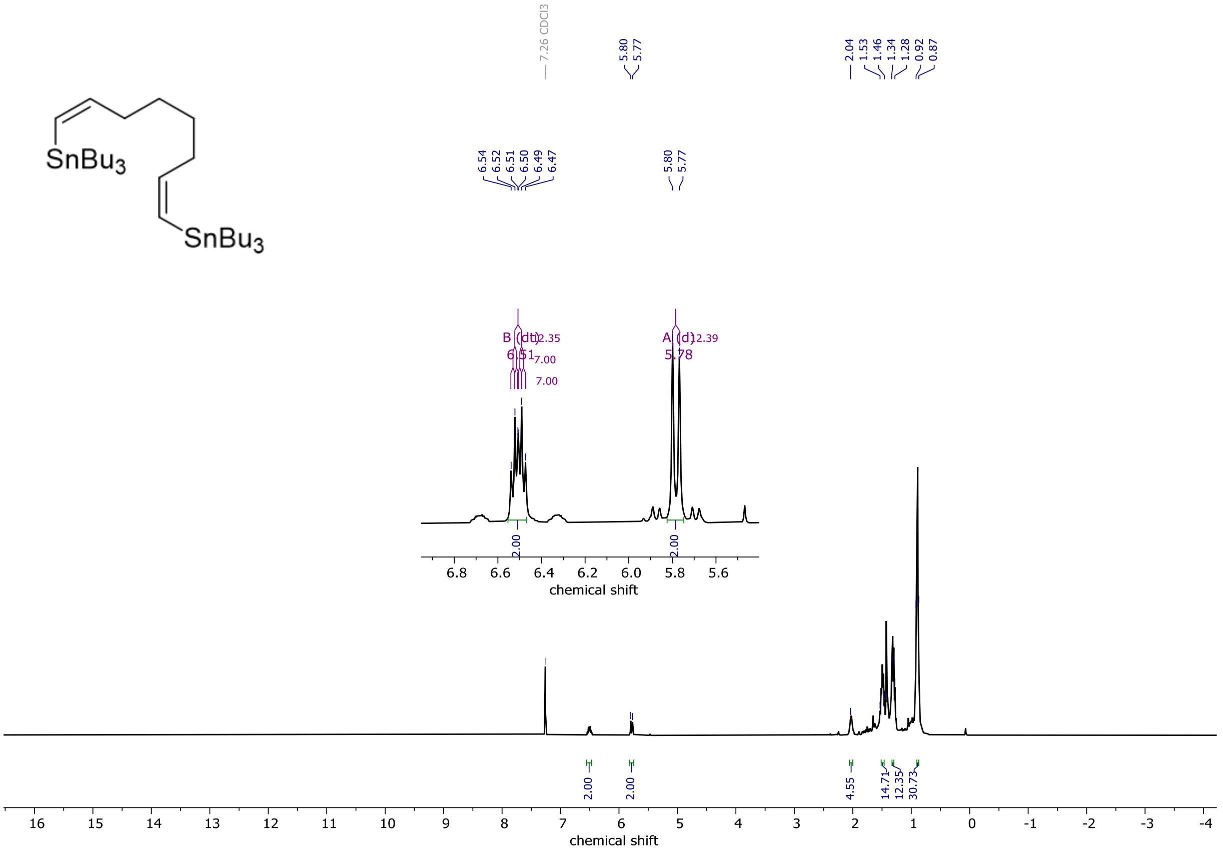

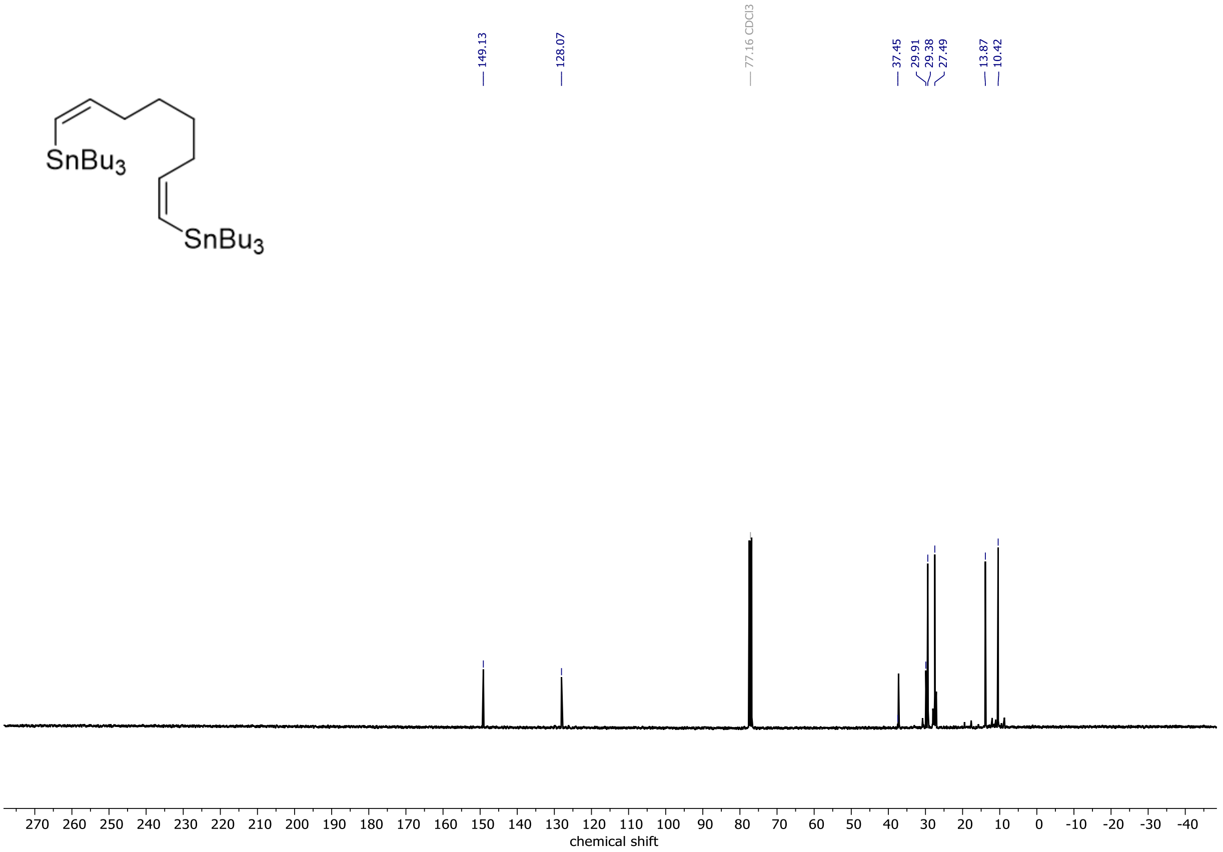

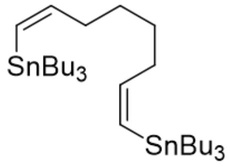
SnBu3
SnBu3

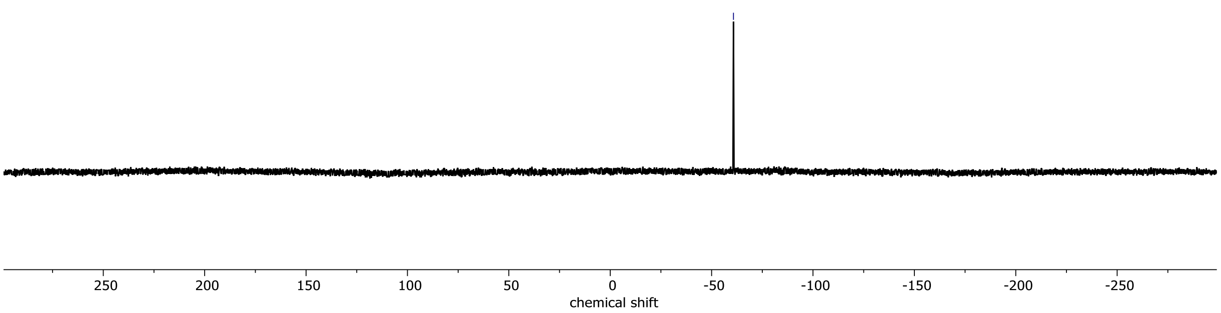
-60.78
250
200
150
100
50
0
-50
-100
-150
-200
-250
chemical shift

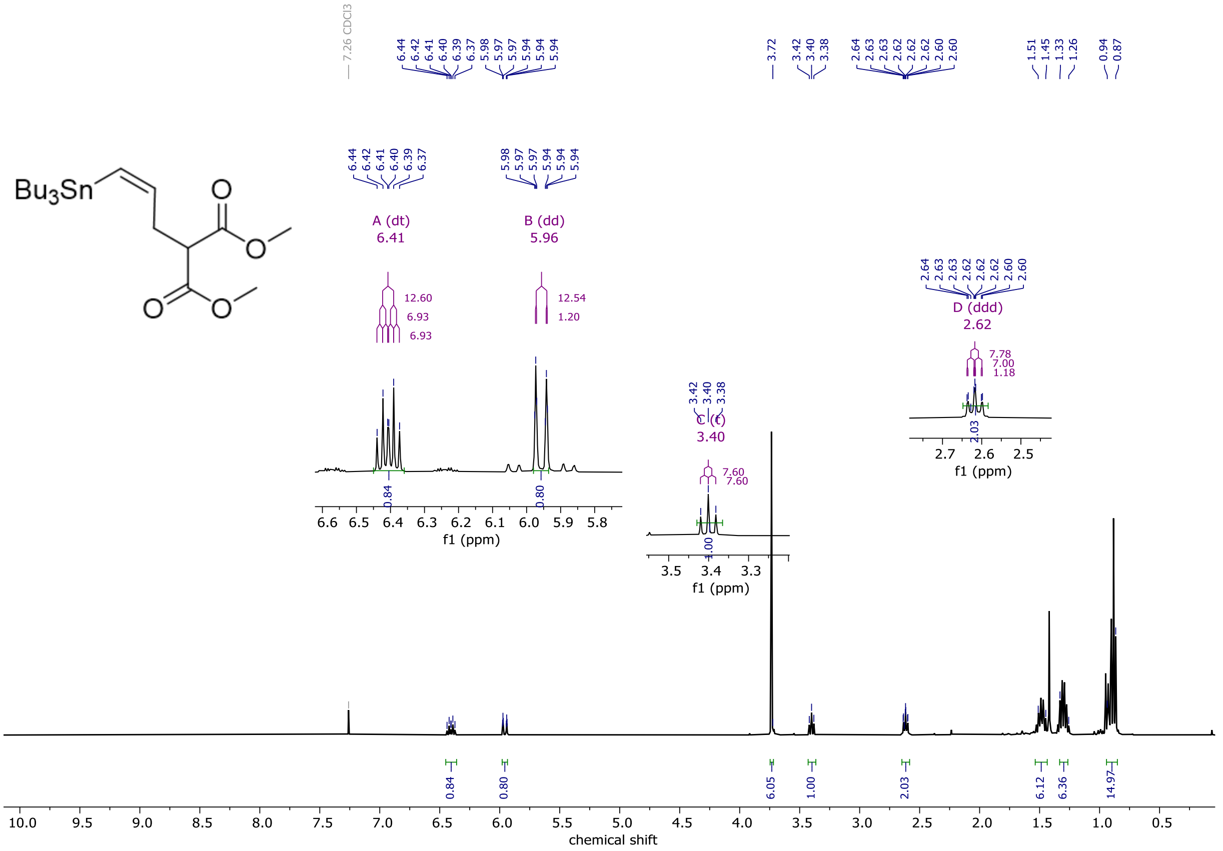

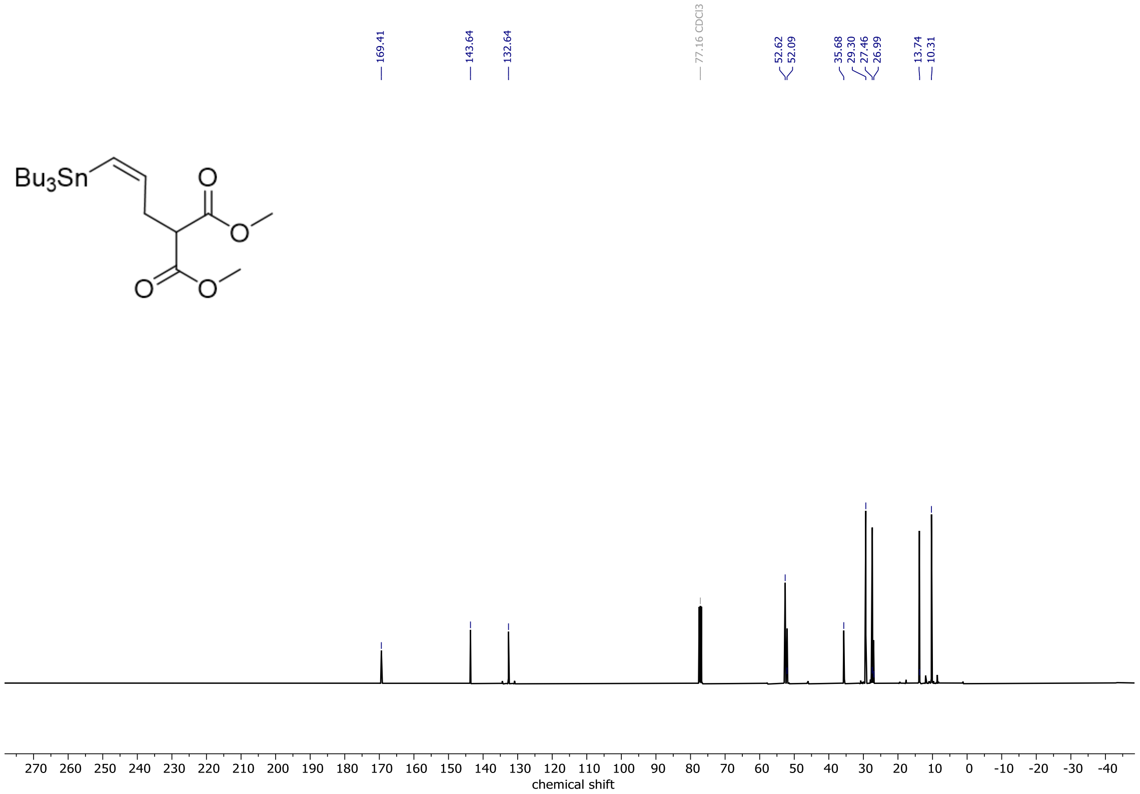

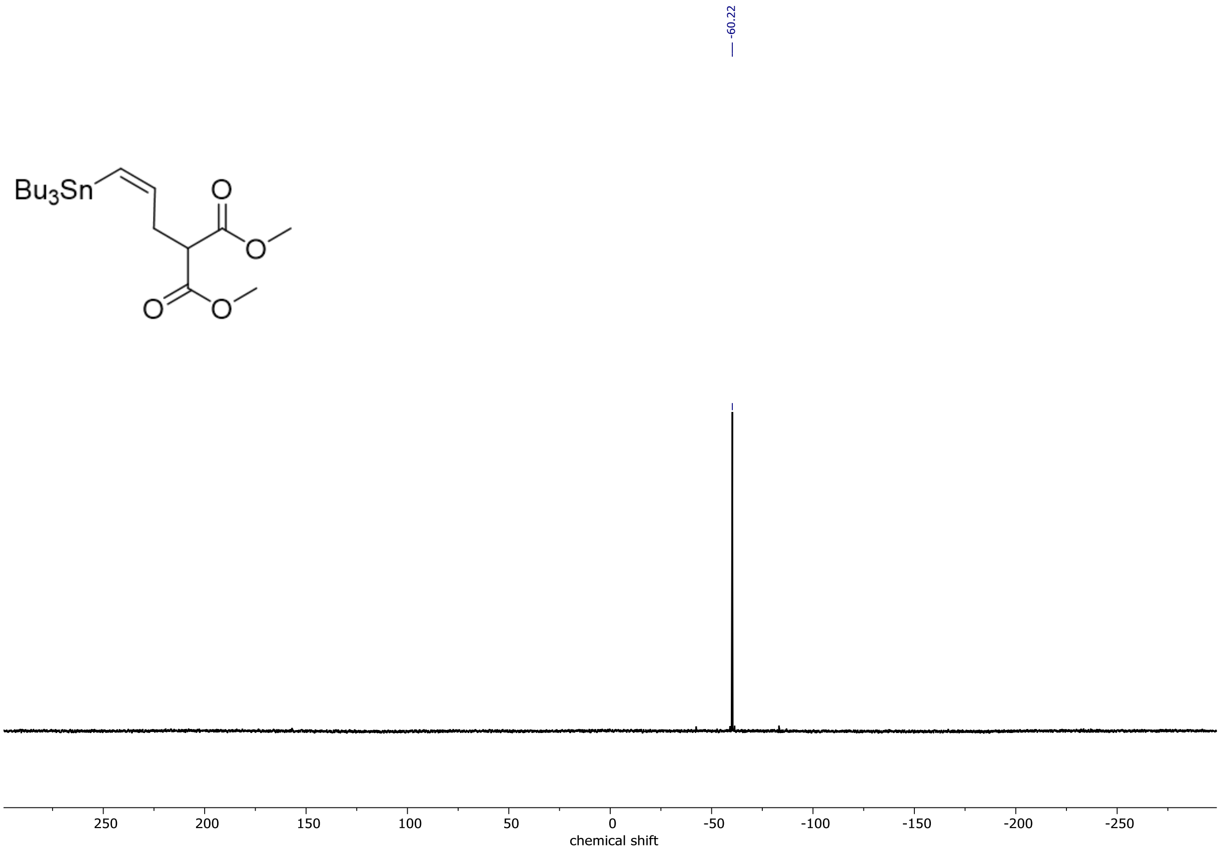
-60.22
Bu3Sn
O
O
O
O
250
200
150
100
50
0
-50
-100
-150
-200
-250
chemical shift

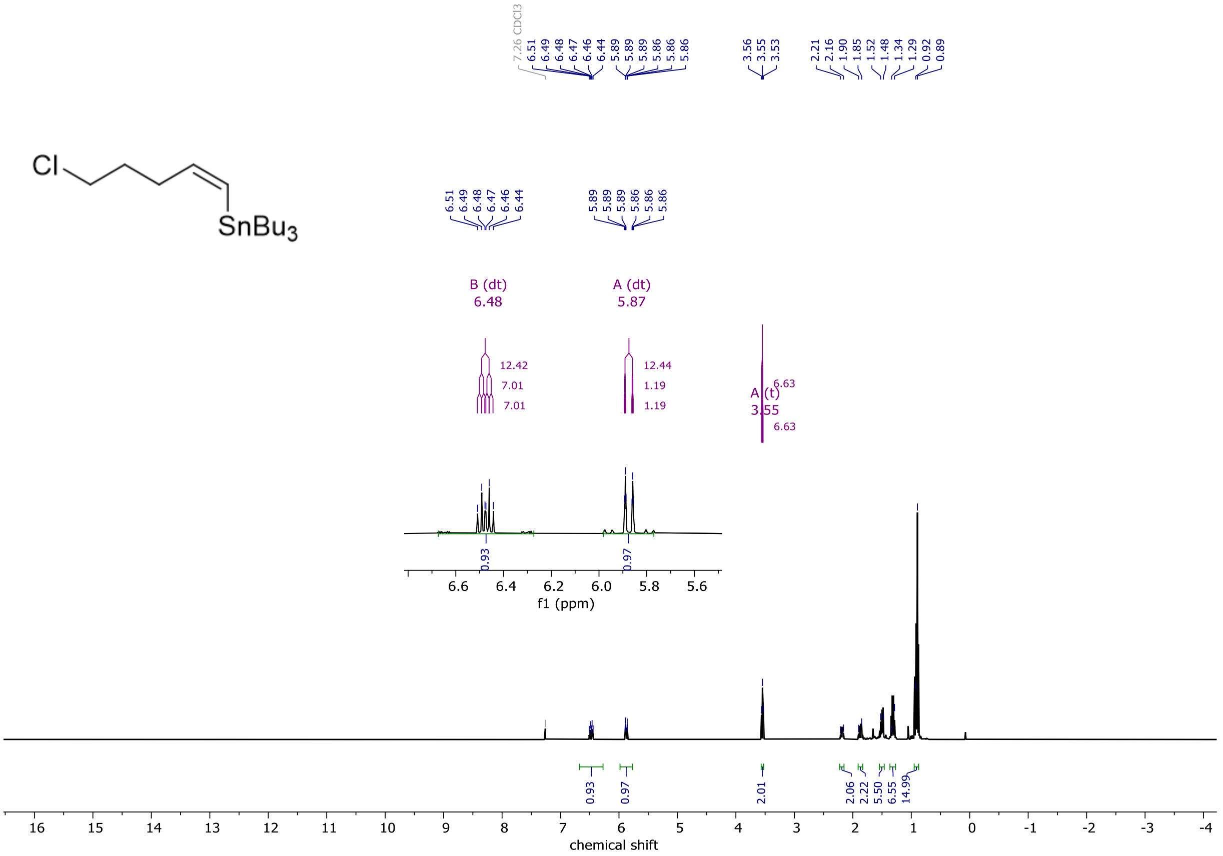

Cl
SnBu3
7.26 CDCl3
6.51
6.49
6.48
6.47
6.46
6.44
5.89
5.89
5.89
5.86
5.86
5.86
3.56
3.55
3.53
2.21
2.16
1.90
1.85
1.52
1.48
1.34
1.29
0.92
0.89
B (dt)
6.48
12.42
7.01
7.01
A (dt)
5.87
12.44
1.19
1.19
A (t)
3.55
6.63
6.63
0.93
0.97
6.6 6.4 6.2 6.0 5.8 5.6
f1 (ppm)
0.93
0.97
2.01
2.06
2.22
5.50
6.55
14.99
16 15 14 13 12 11 10 9 8 7 6 5 4 3 2 1 0 -1 -2 -3 -4
chemical shift

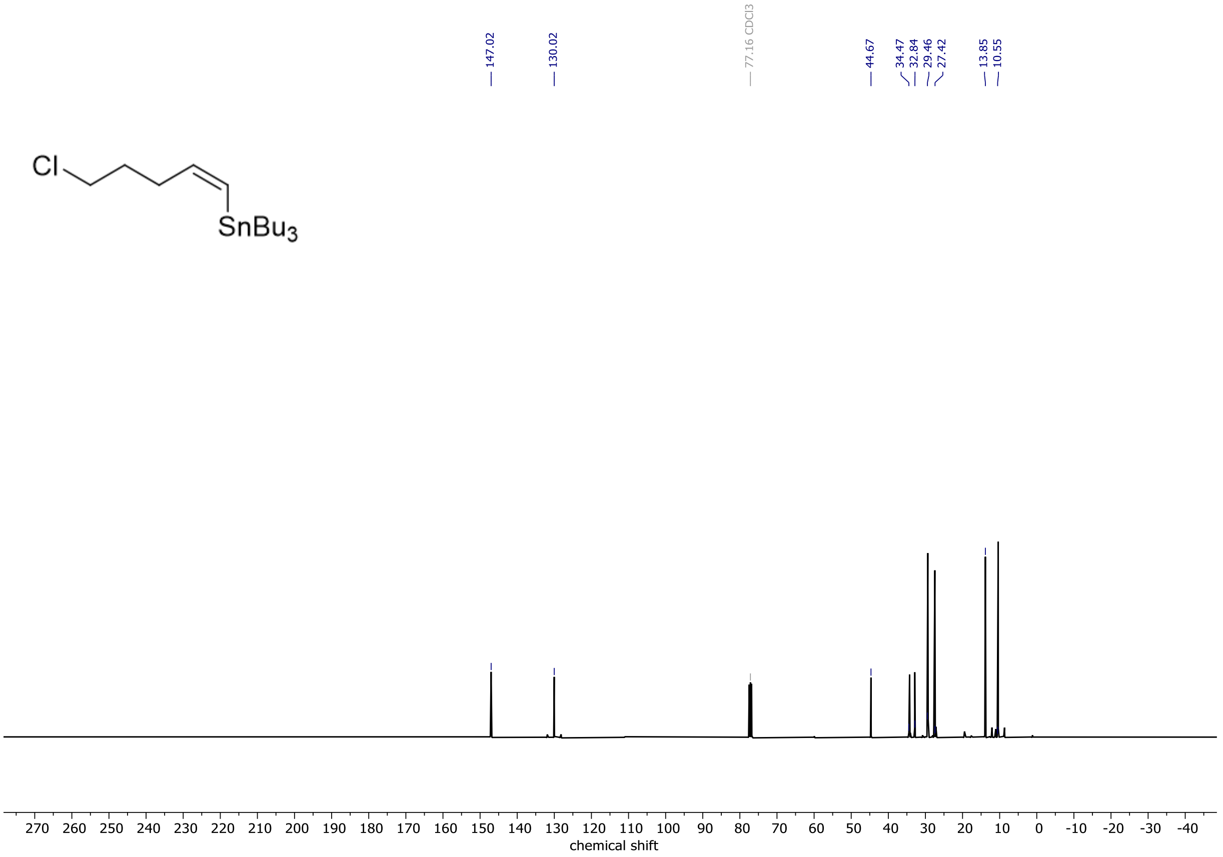

Cl
SnBu3
147.02
130.02
77.16 CDCl3
44.67
34.47
32.84
29.46
27.42
13.85
10.55
270 260 250 240 230 220 210 200 190 180 170 160 150 140 130 120 110 100 90 80 70 60 50 40 30 20 10 0 -10 -20 -30 -40
chemical shift

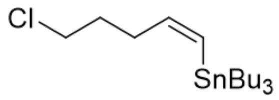

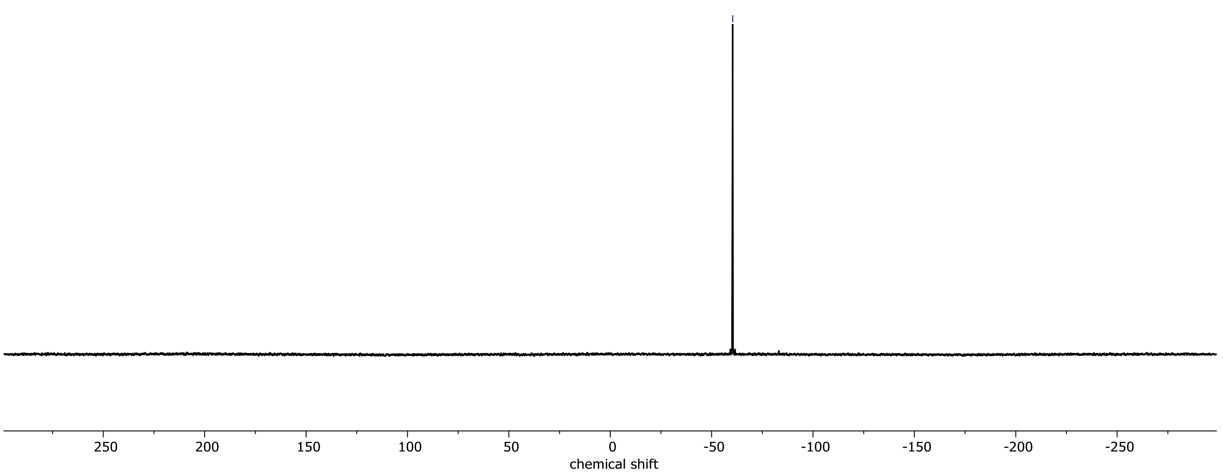
-60.41
250
200
150
100
50
0
-50
-100
-150
-200
-250
chemical shift

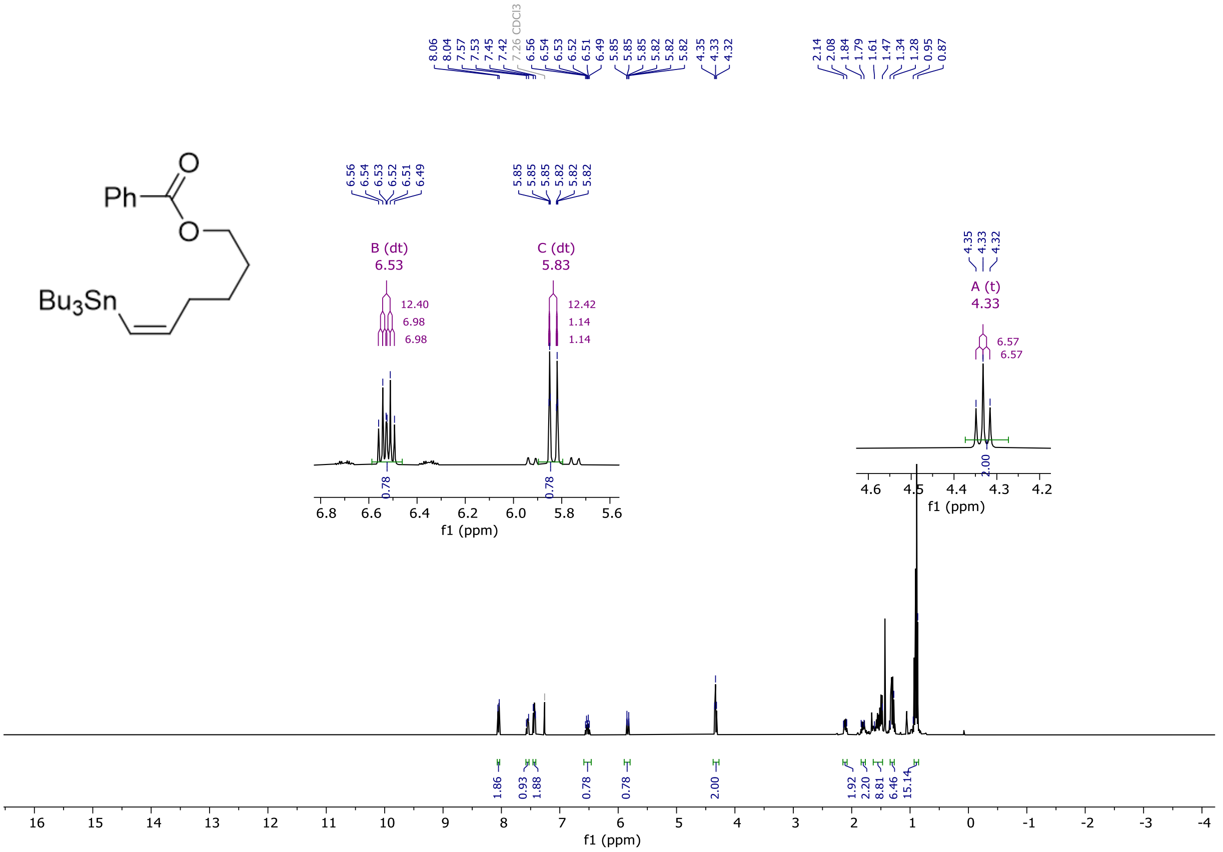

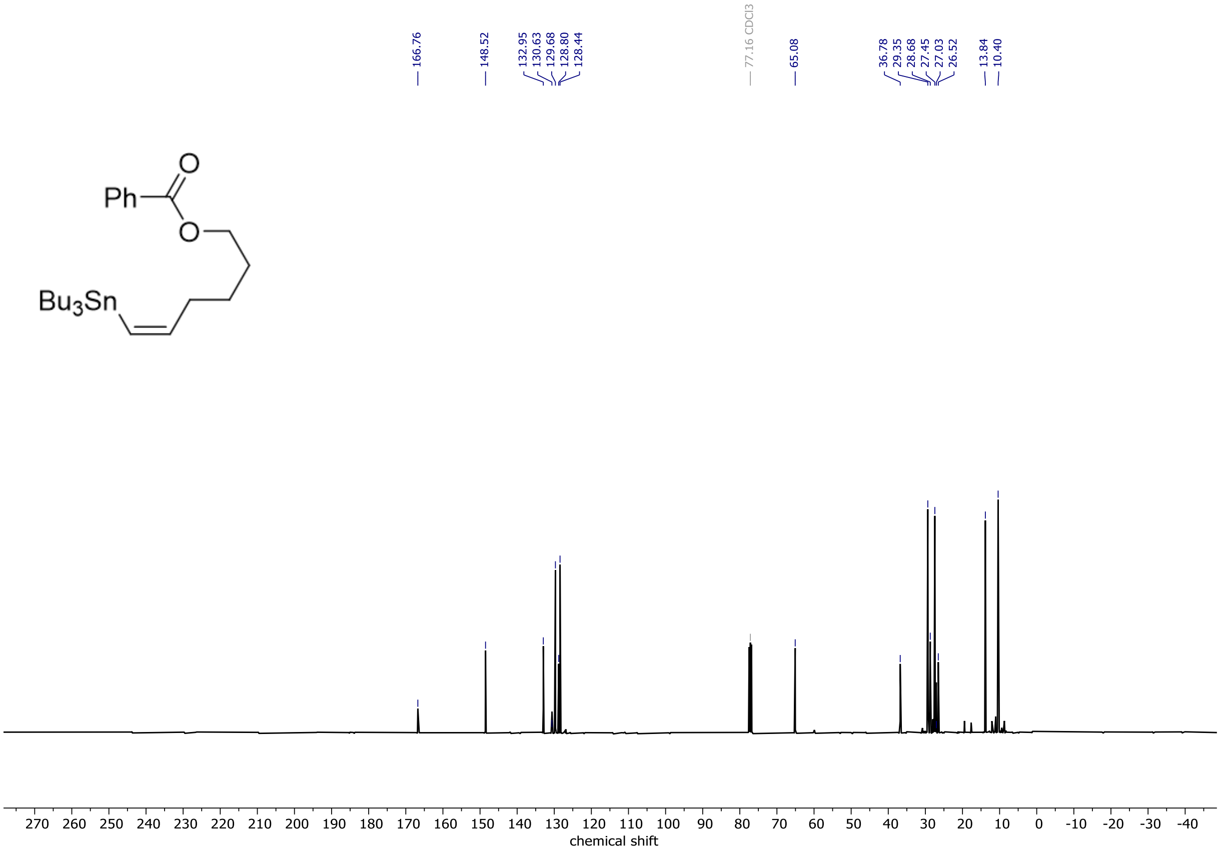

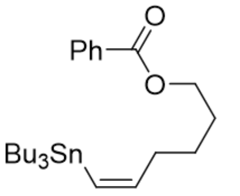
Ph
O
O
Bu3Sn

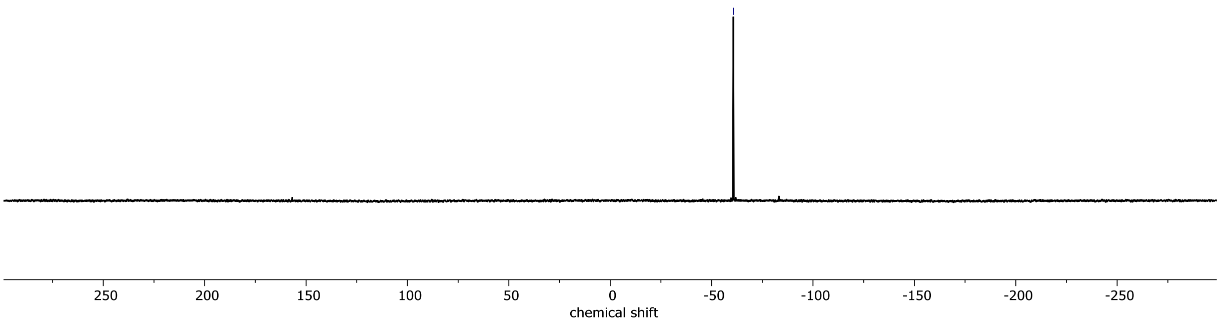
-60.72
250
200
150
100
50
0
-50
-100
-150
-200
-250
chemical shift

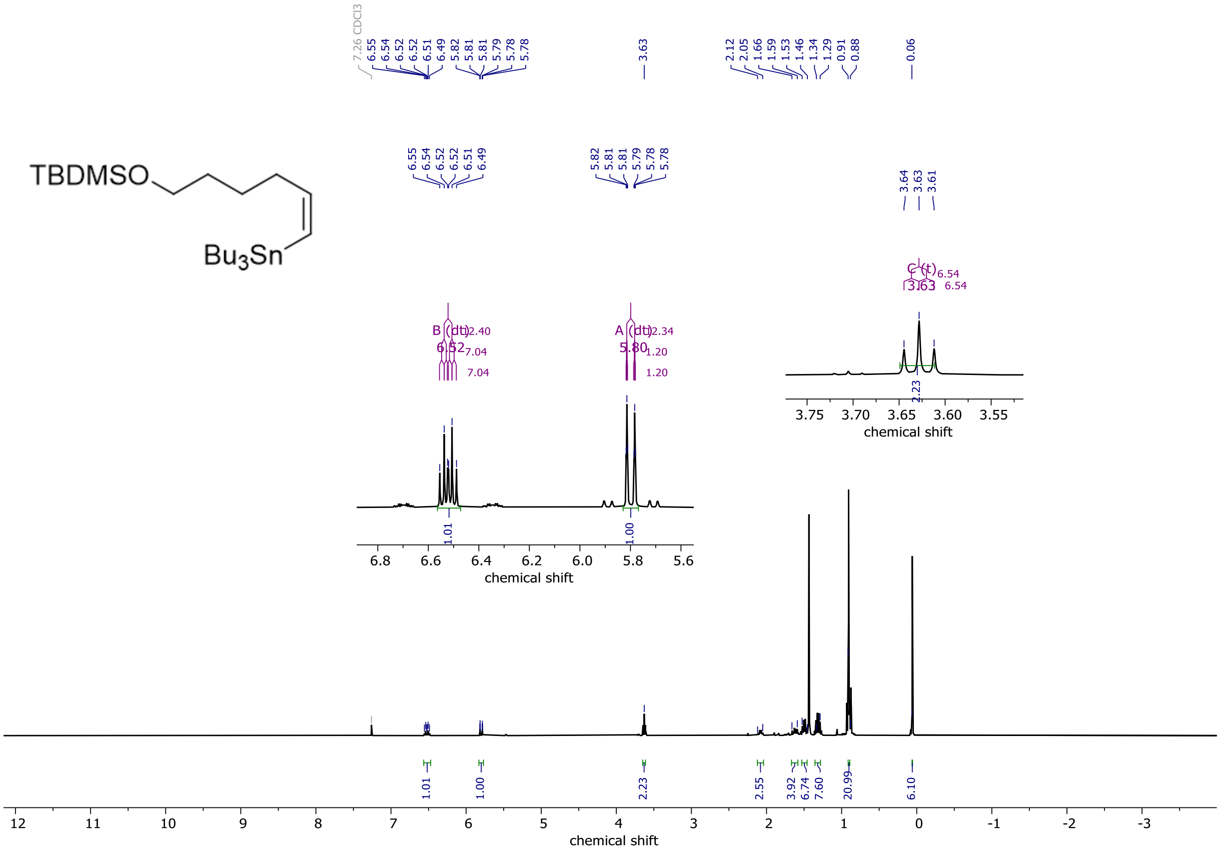
TBDMSO
Bu3Sn
7.26 CDCl3
6.55 6.54 6.52 6.52 6.51 6.49 5.82 5.81 5.81 5.79 5.78 5.78
3.63
2.12 2.05 1.66 1.59 1.53 1.46 1.34 1.29 0.91 0.88
0.06
3.64 3.63 3.61
C (t) 3.63
B (dt) 6.52
A (dt) 5.80
1.01 1.00 2.23 2.55 3.92 6.74 7.60 20.99 6.10
chemical shift

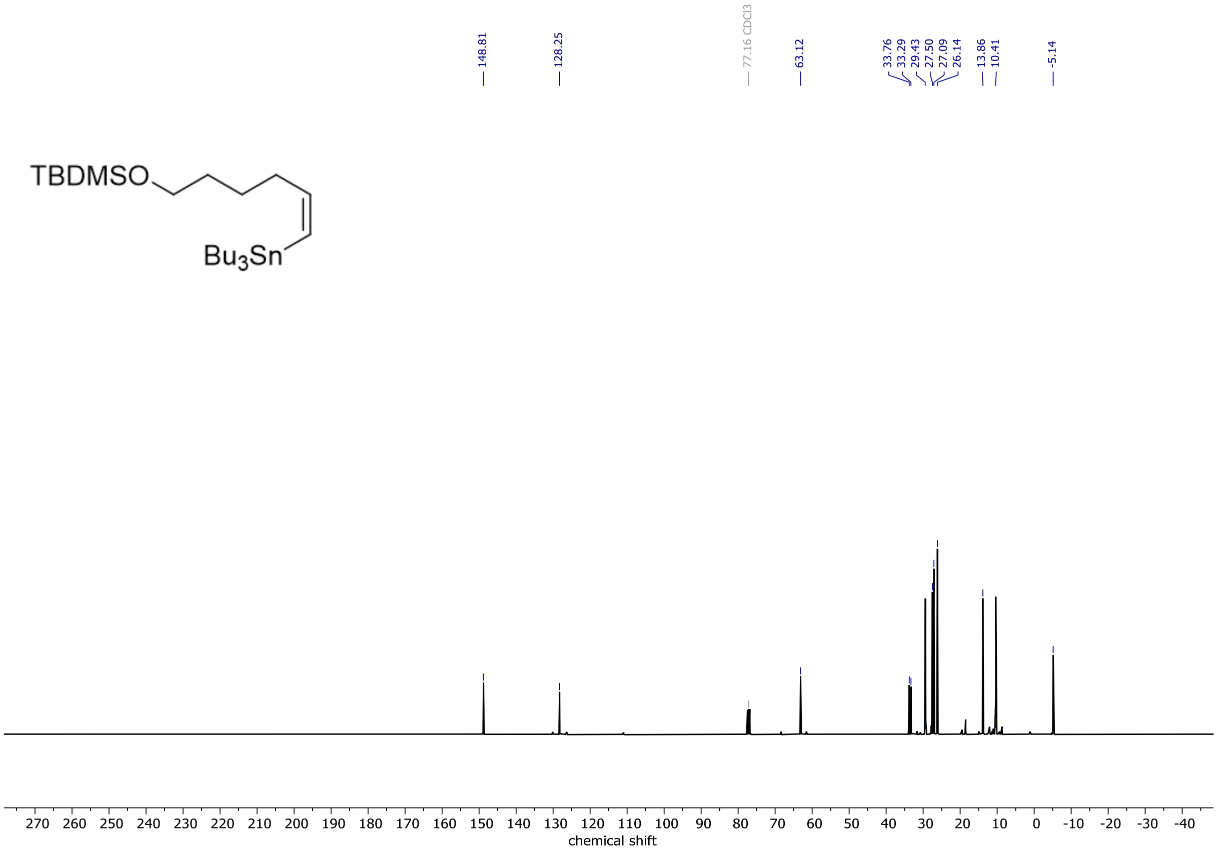
TBDMSO
Bu3Sn
148.81 128.25 77.16 CDCl3 63.12 33.76 33.29 29.43 27.50 27.09 26.14 13.86 10.41 -5.14
chemical shift

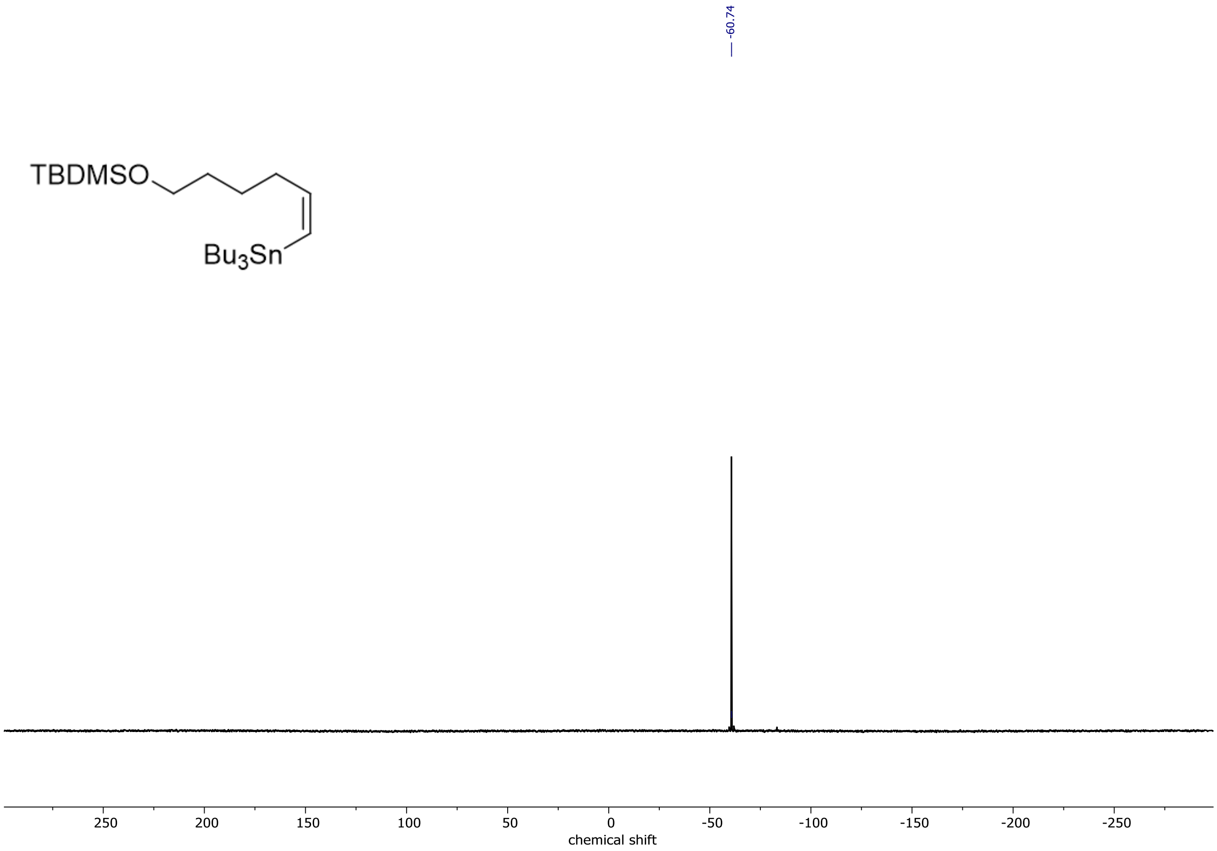

-60.74
TBDMSO
Bu3Sn
250
200
150
100
50
0
-50
-100
-150
-200
-250
chemical shift

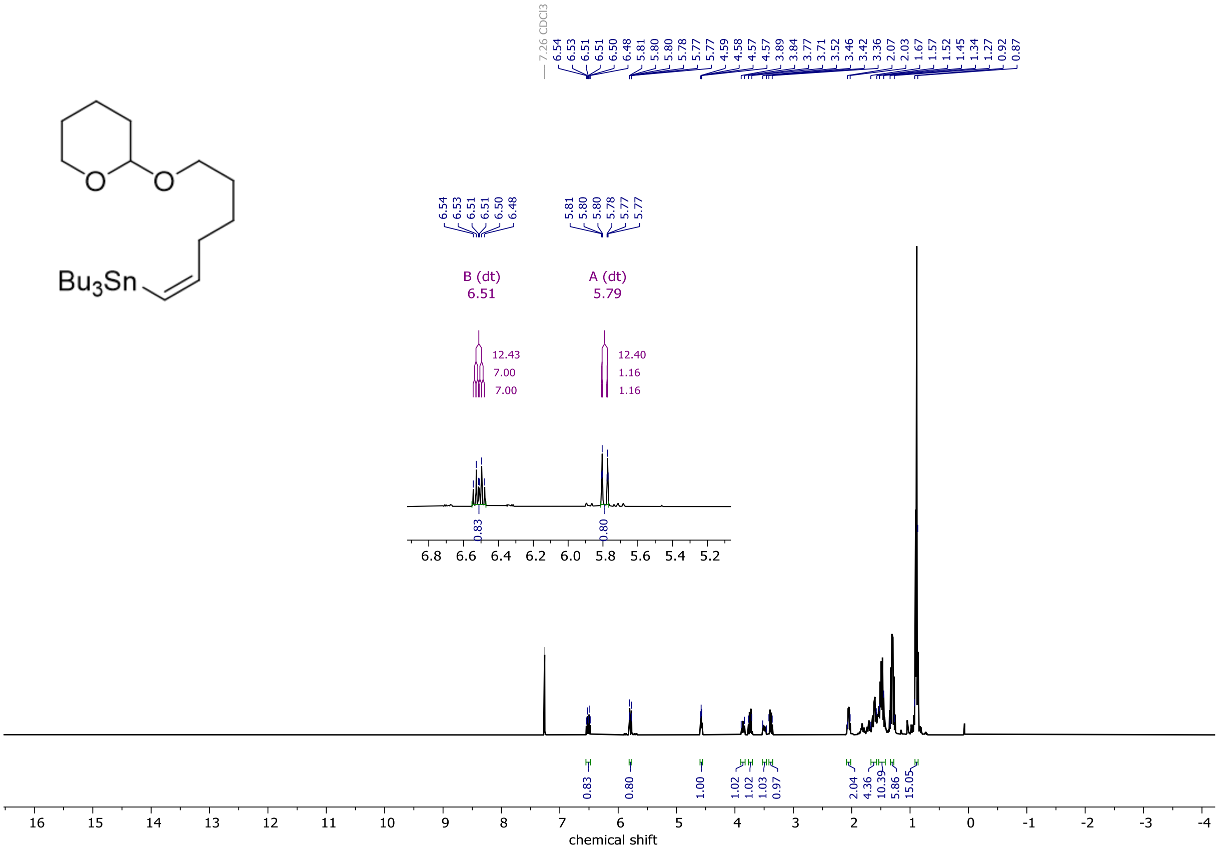

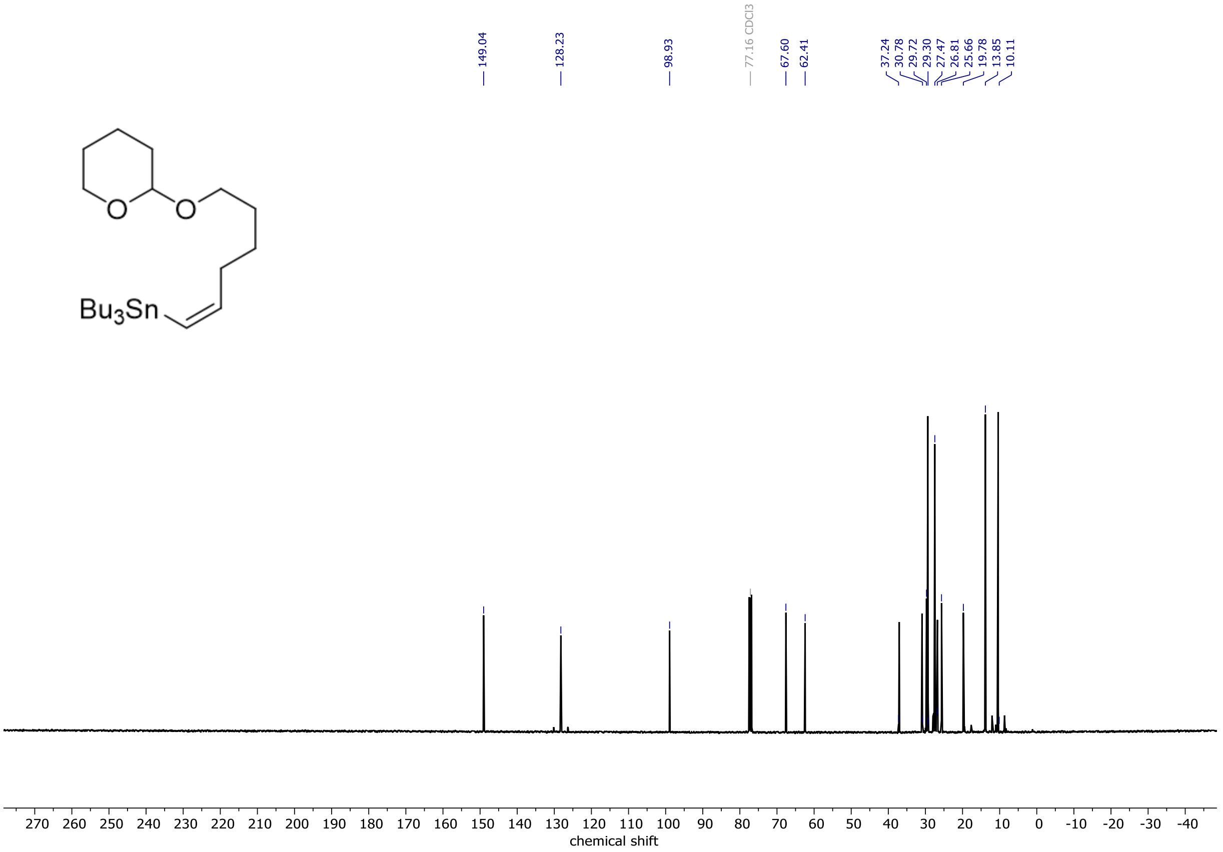

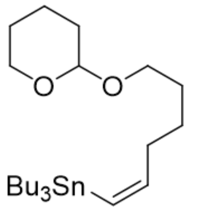

Bu3Sn
O
O

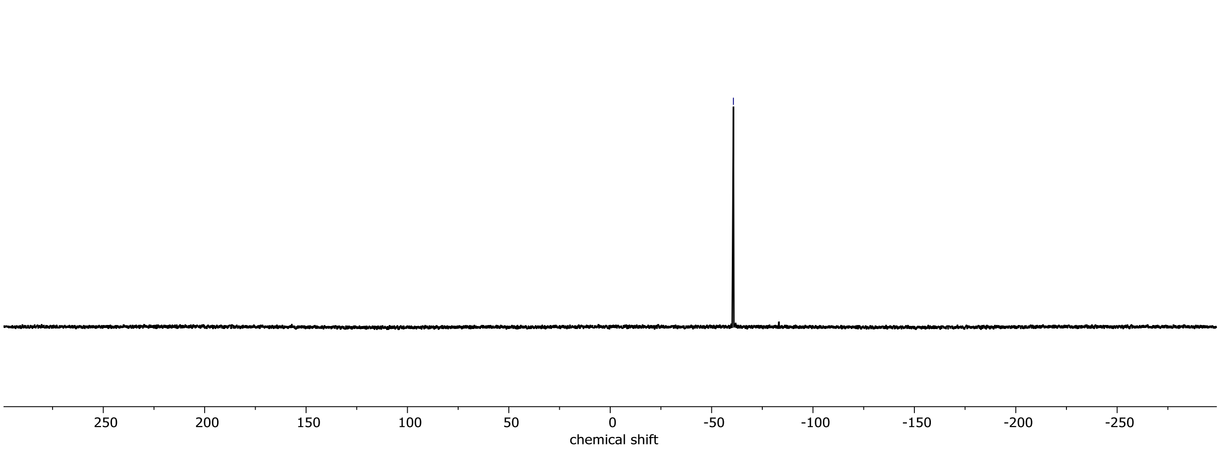

-60.76
250
200
150
100
50
0
-50
-100
-150
-200
-250
chemical shift

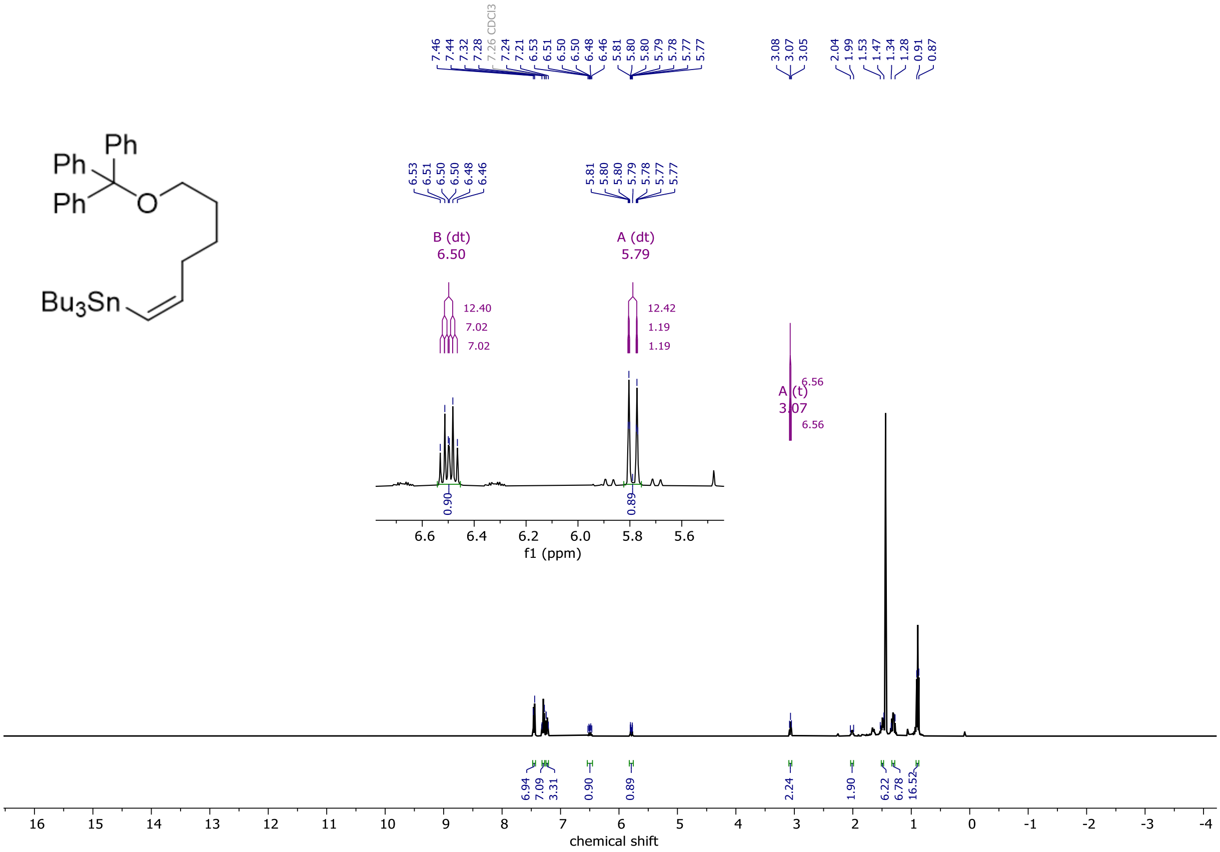

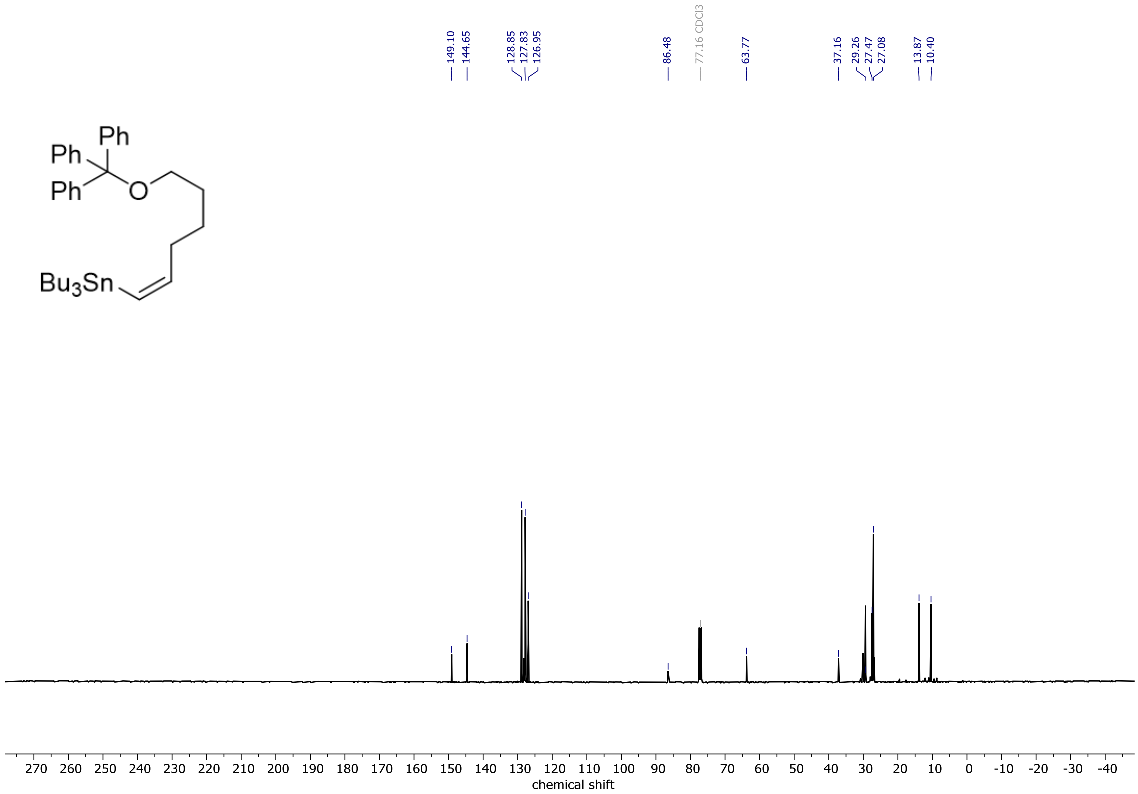

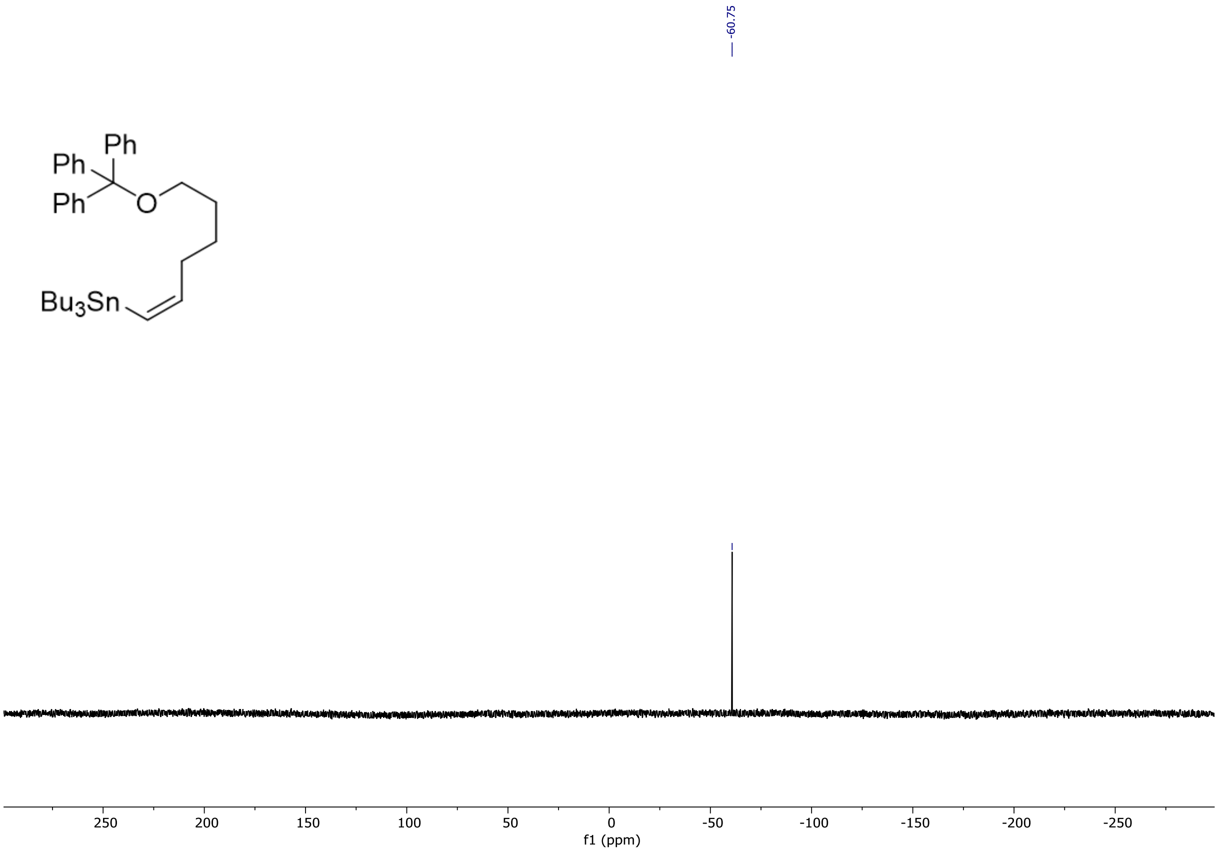
-60.75
Ph
Ph
Ph
O
Bu3Sn
250
200
150
100
50
0
-50
-100
-150
-200
-250
f1 (ppm)

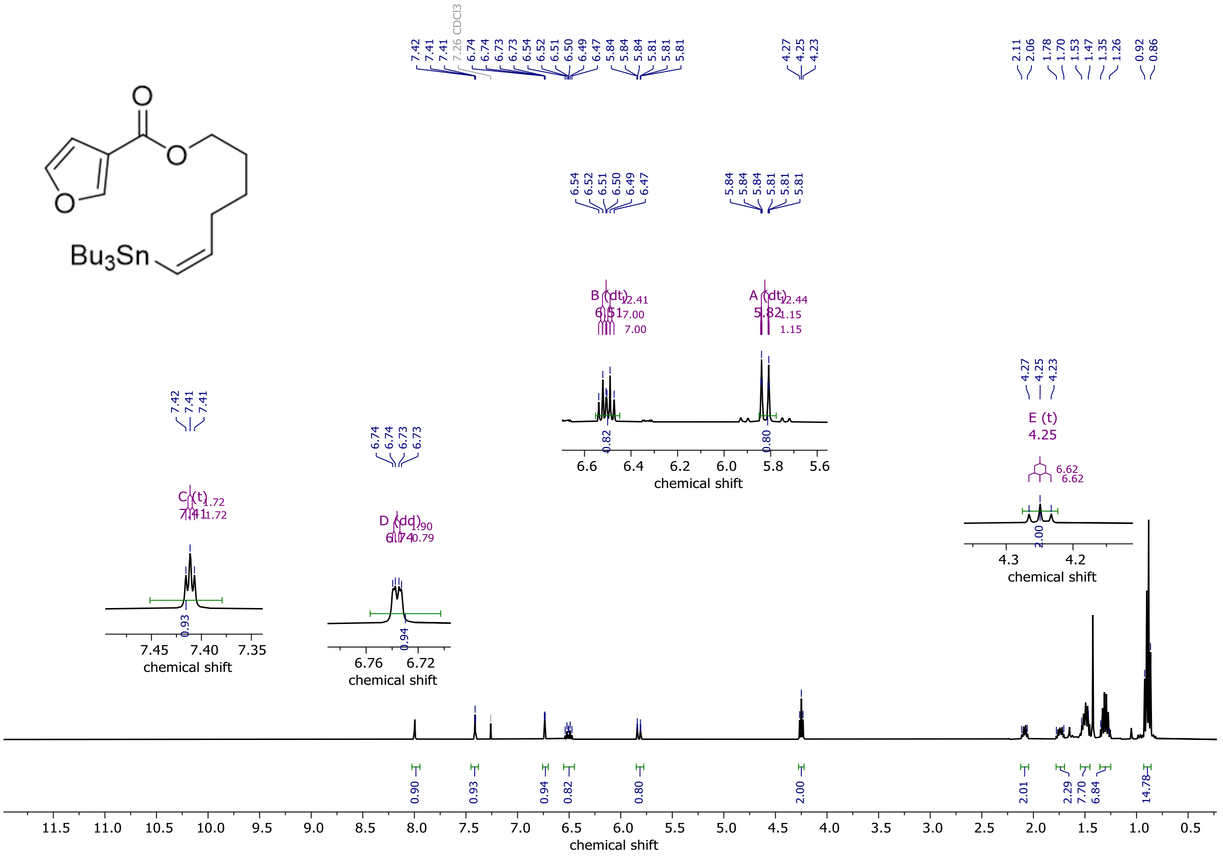

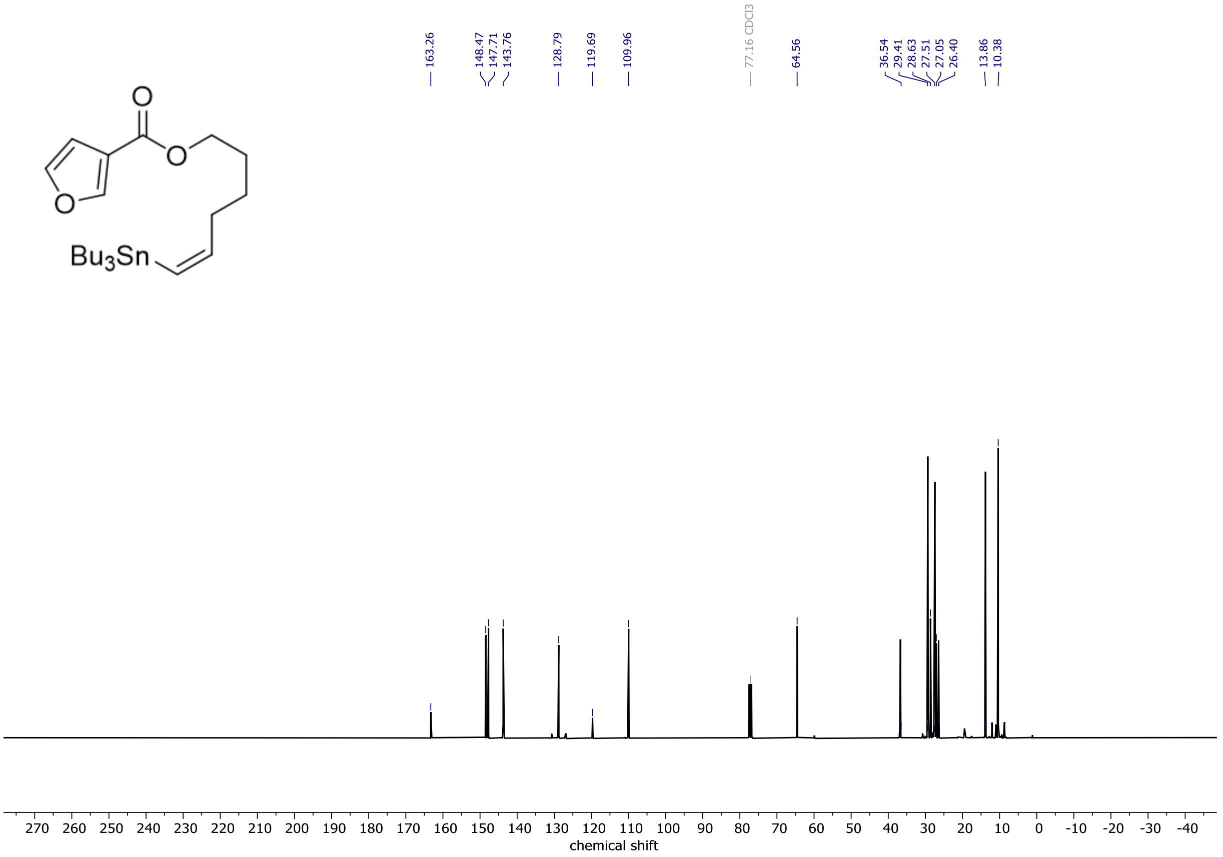

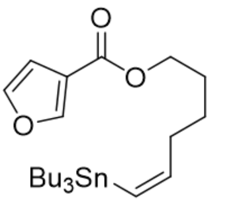
O
O
O
Bu3Sn

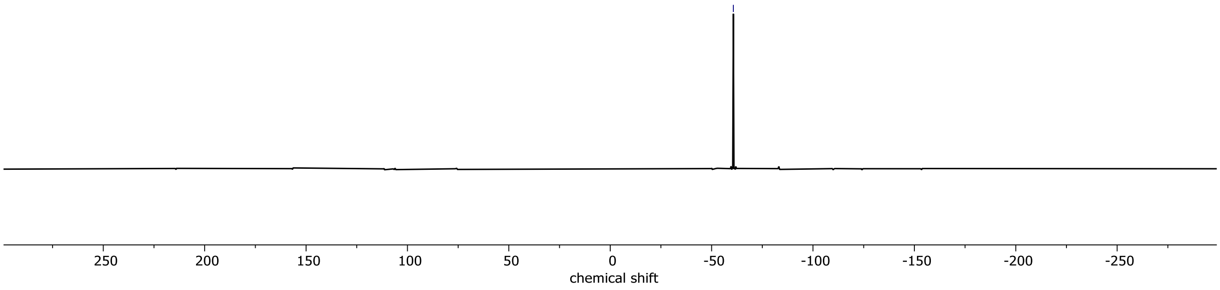
-60.73
250
200
150
100
50
0
-50
-100
-150
-200
-250
chemical shift

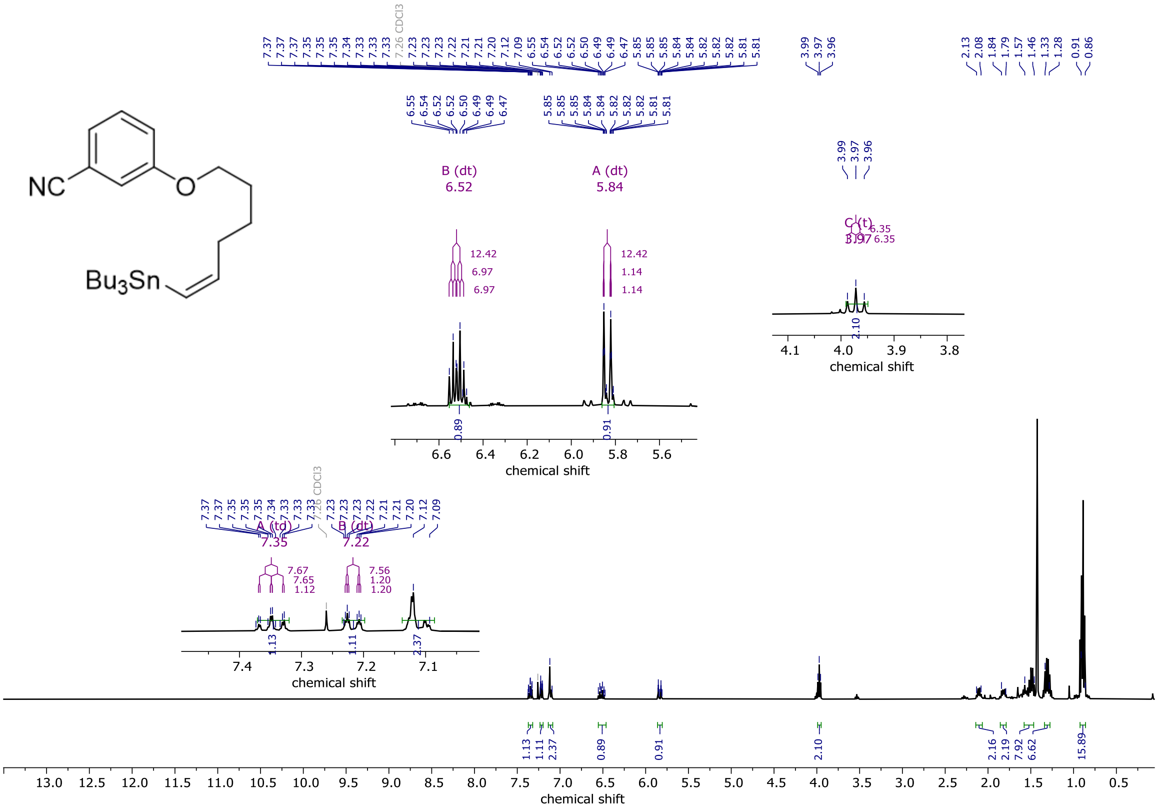

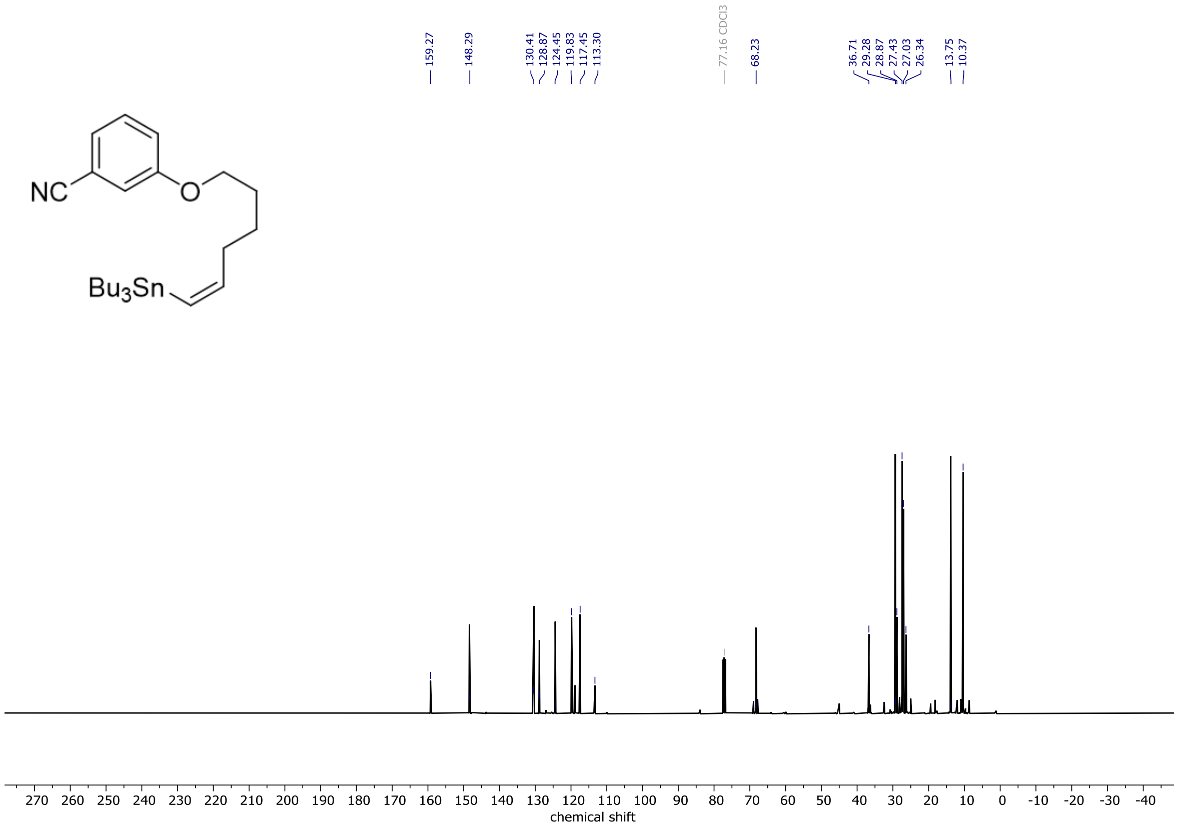

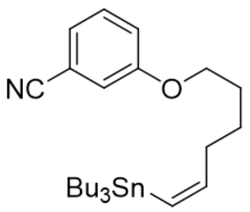

NC
O
Bu3Sn

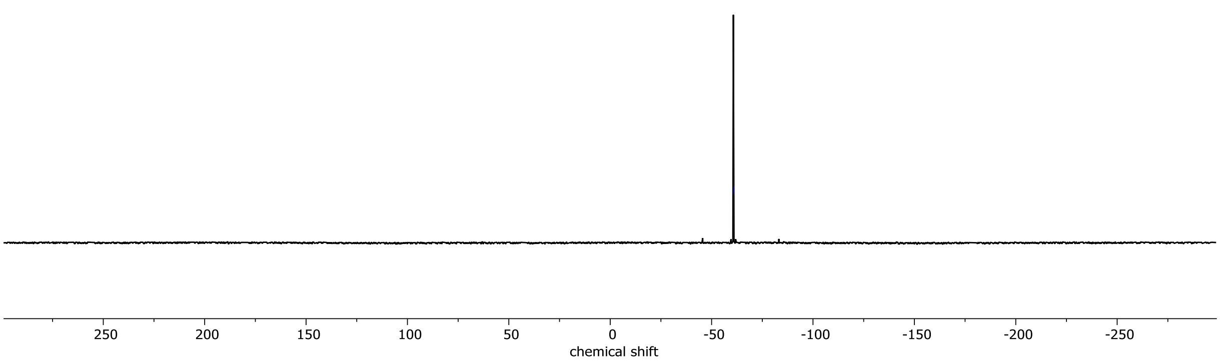

-60.76
250
200
150
100
50
0
-50
-100
-150
-200
-250
chemical shift

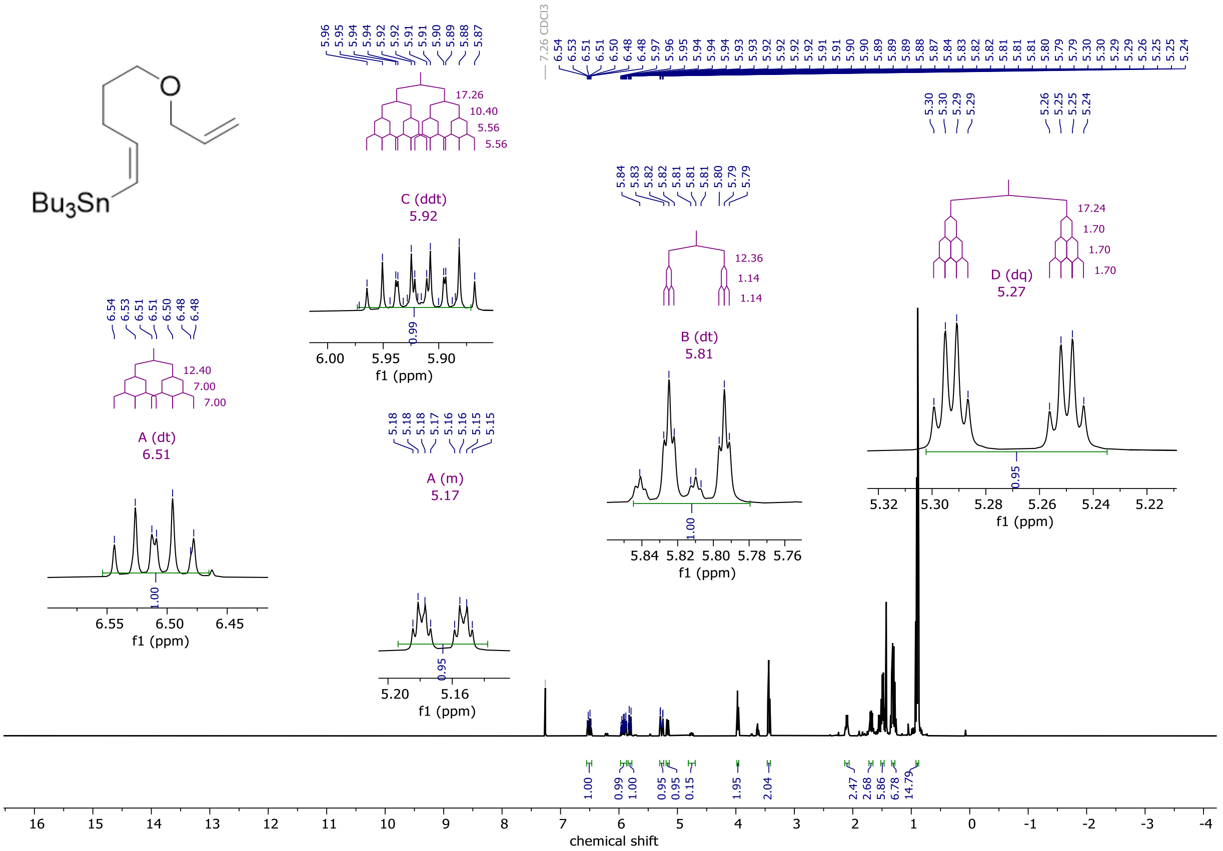

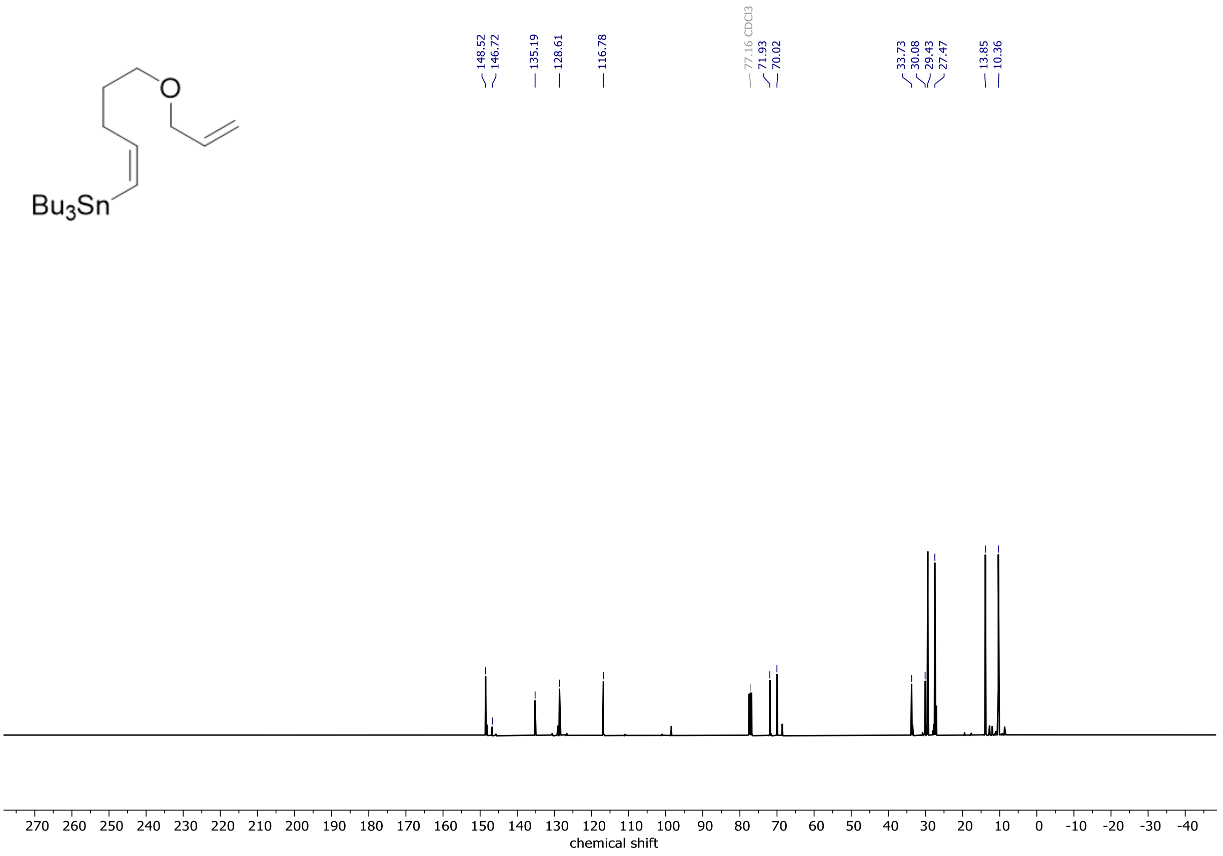

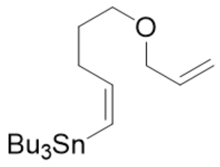
O
Bu3Sn

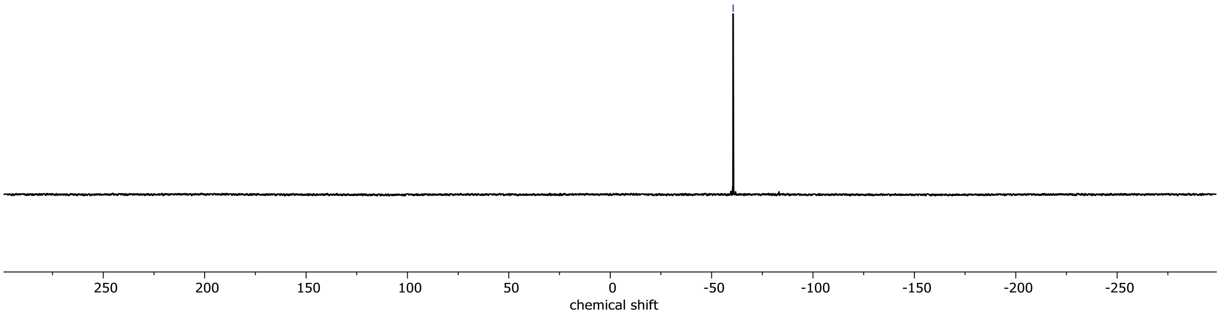
-60.63
250
200
150
100
50
0
-50
-100
-150
-200
-250
chemical shift

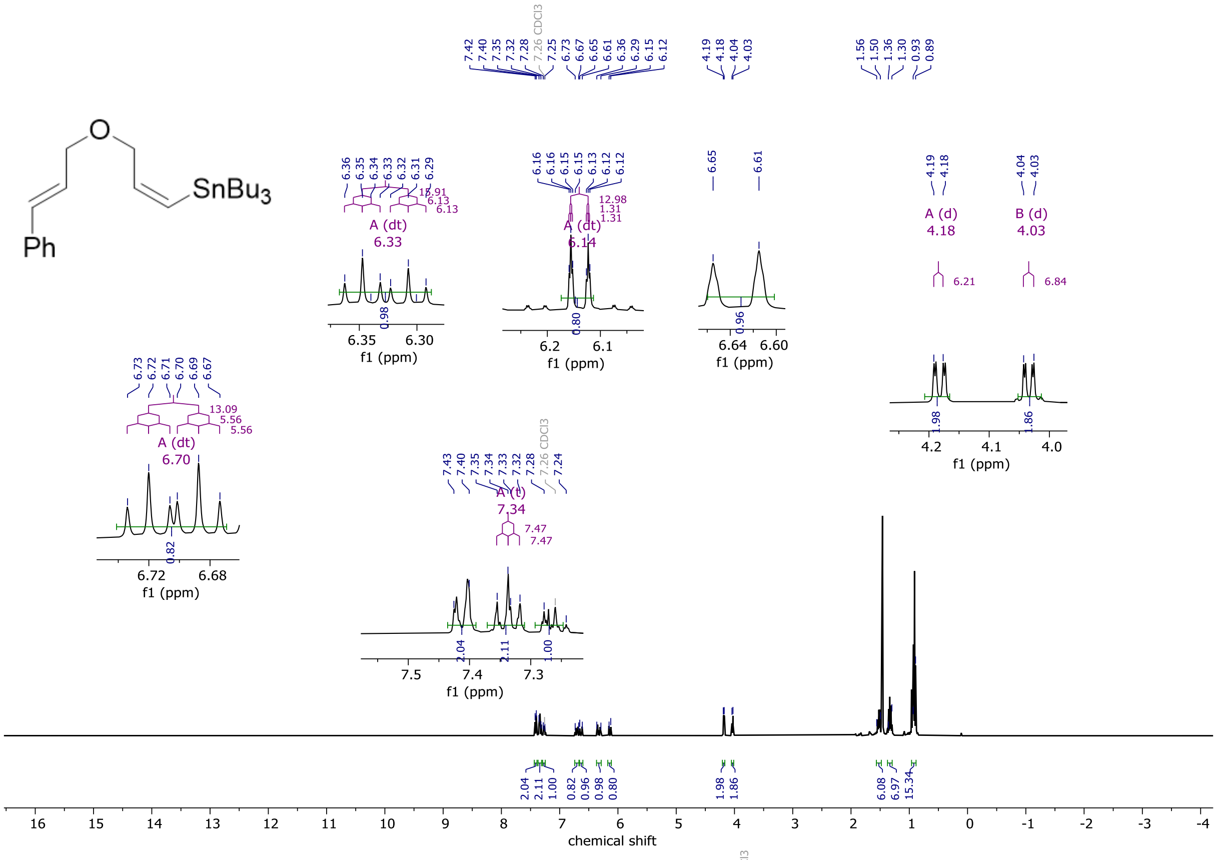

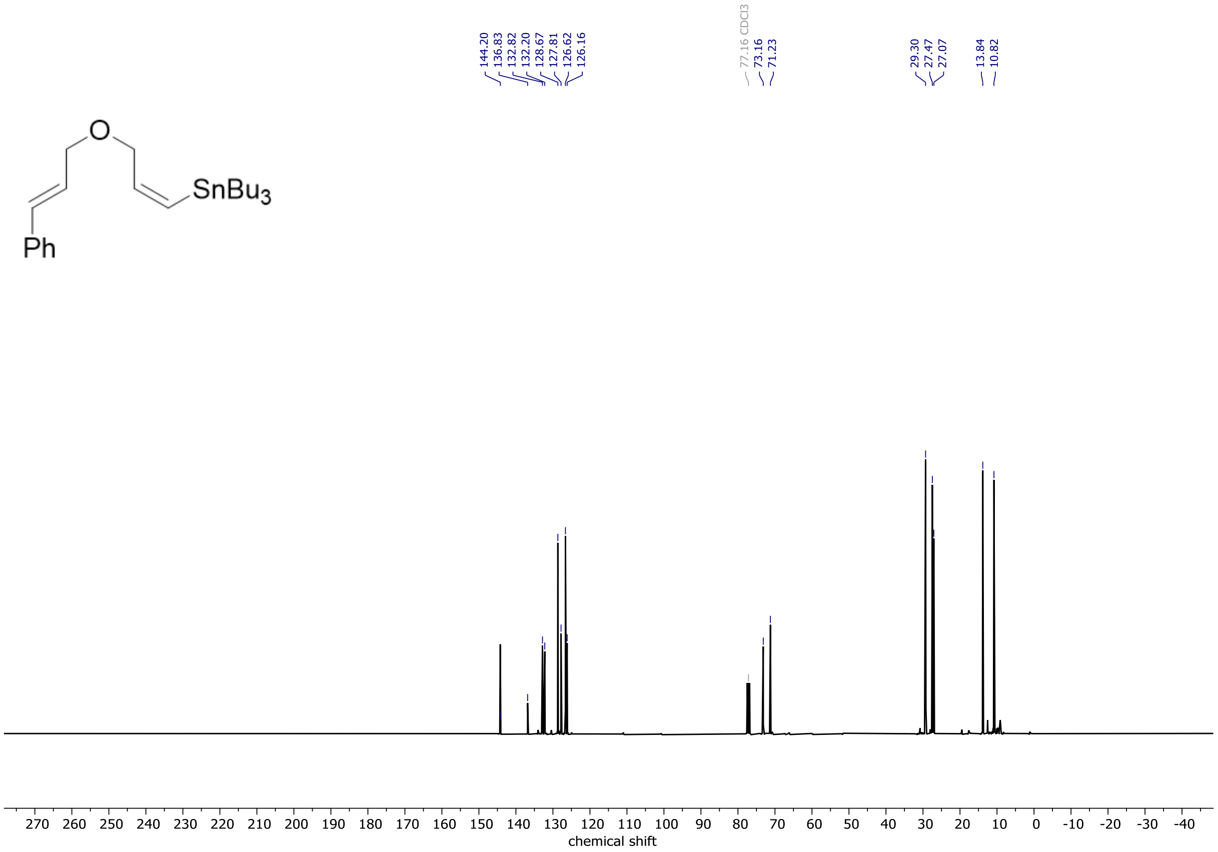

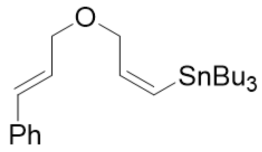

O
SnBu3
Ph

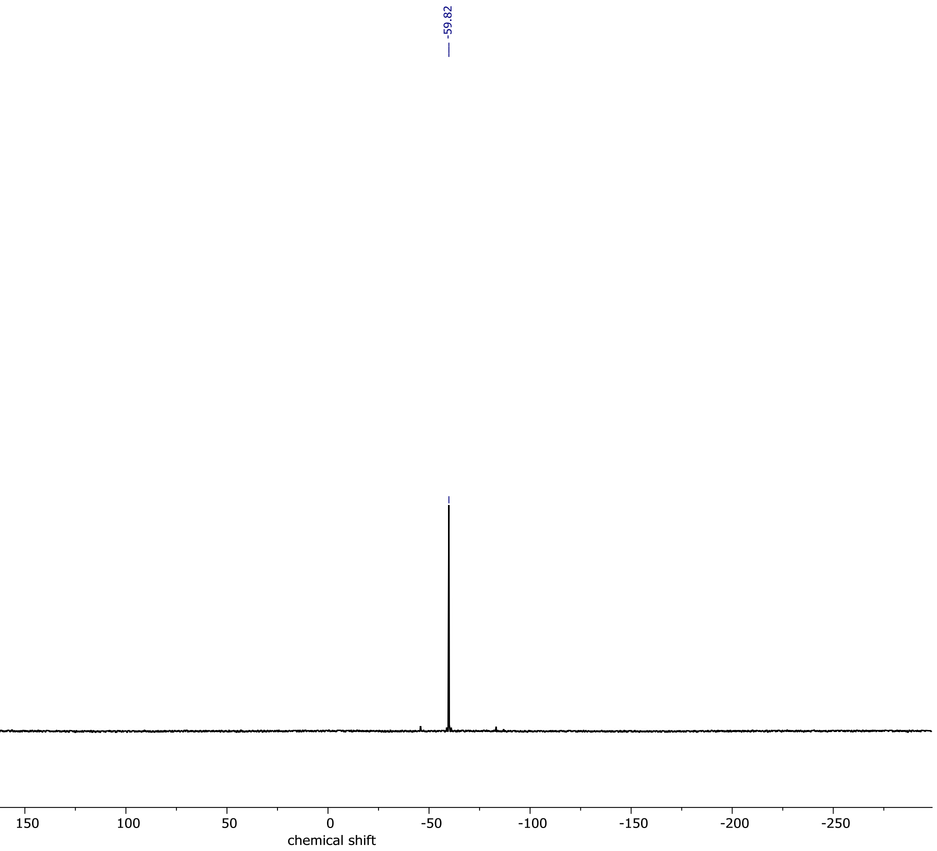

-59.82
250
200
150
100
50
0
-50
-100
-150
-200
-250
chemical shift

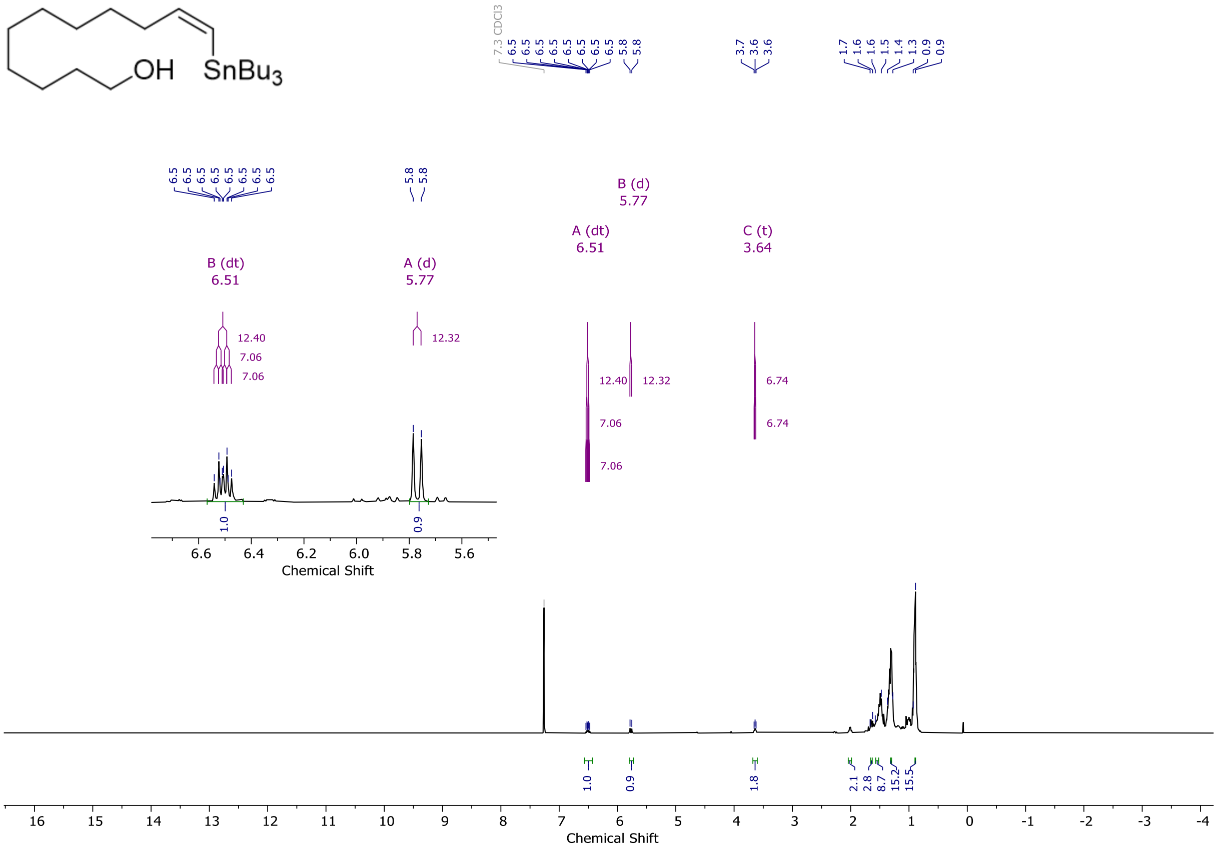

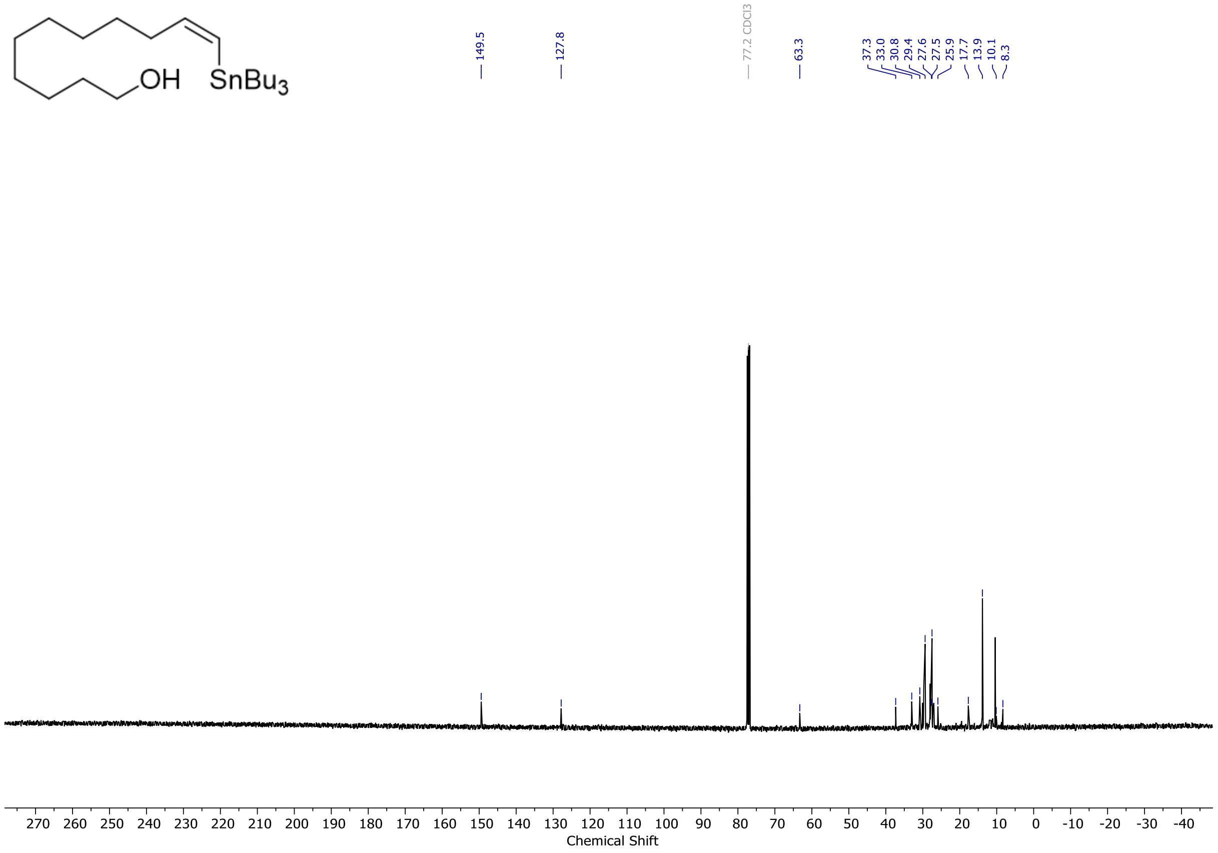

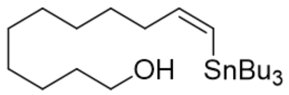
OH
SnBu3

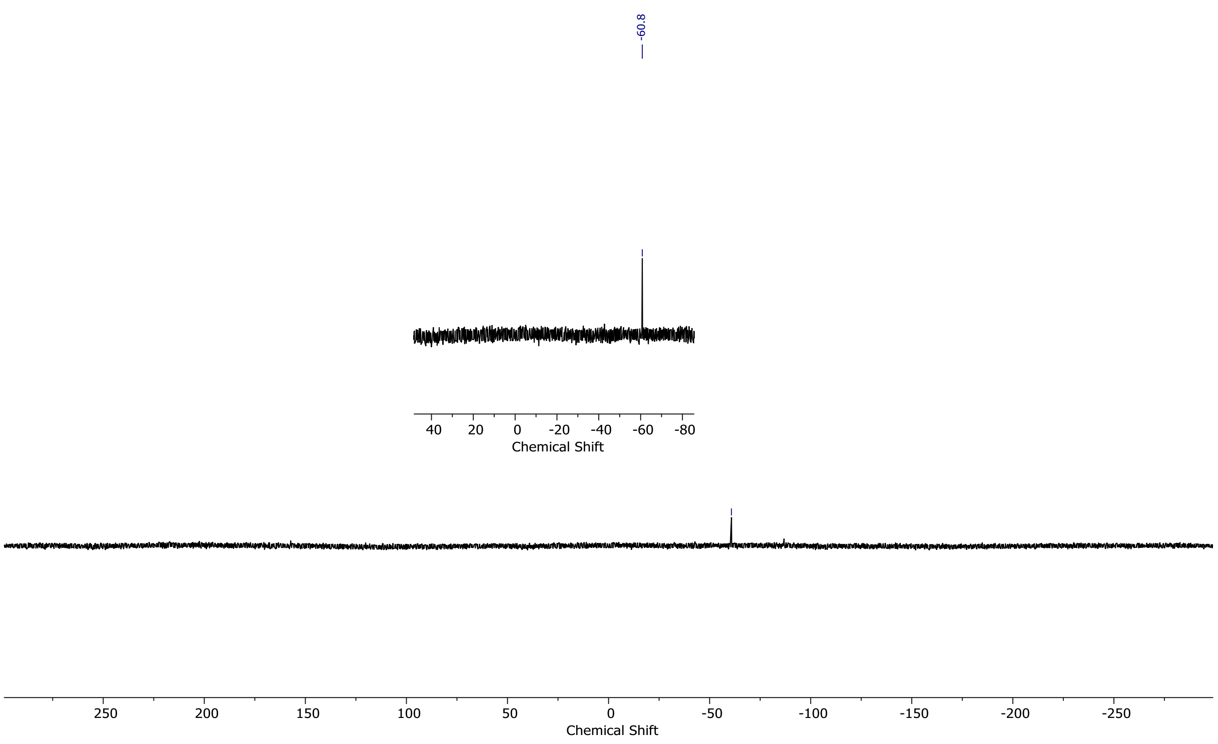
-60.79
-60.8
40
20
0
-20
-40
-60
-80
Chemical Shift
250
200
150
100
50
0
-50
-100
-150
-200
-250
Chemical Shift

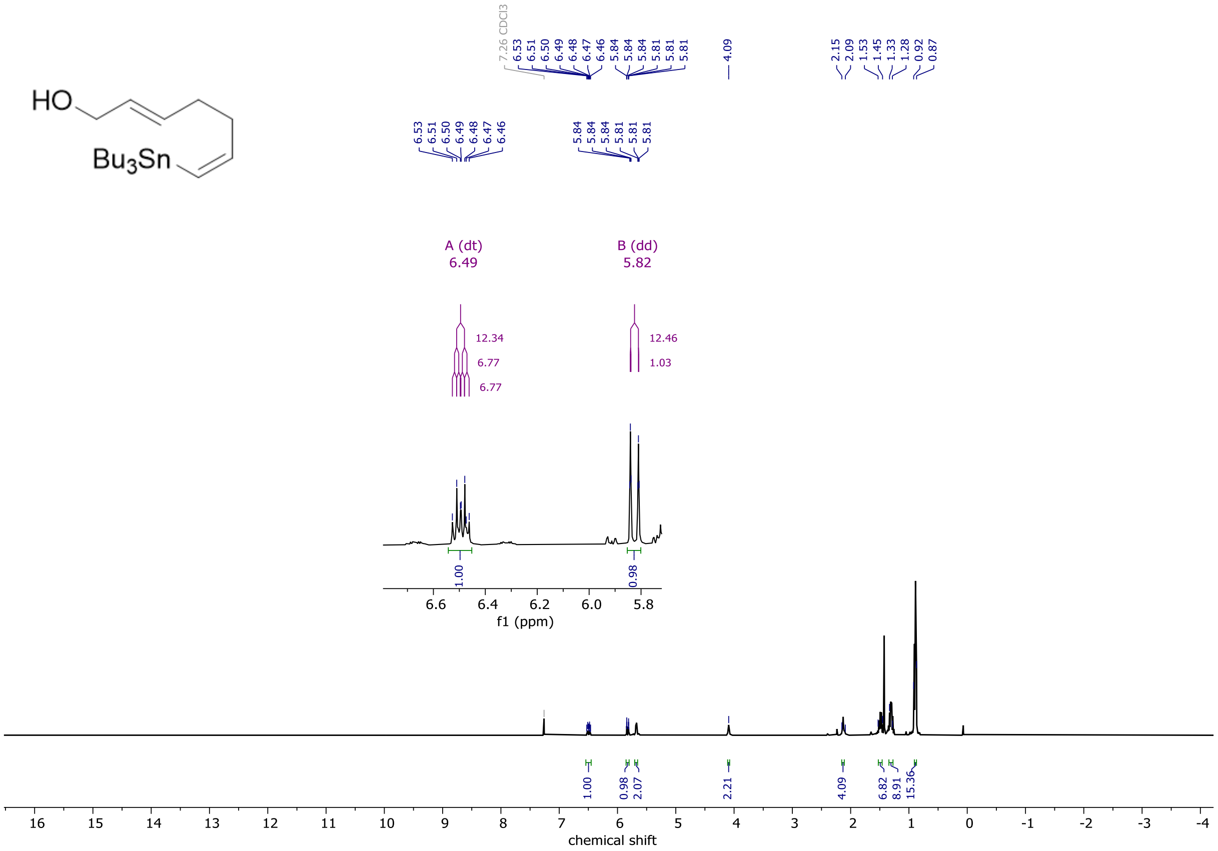

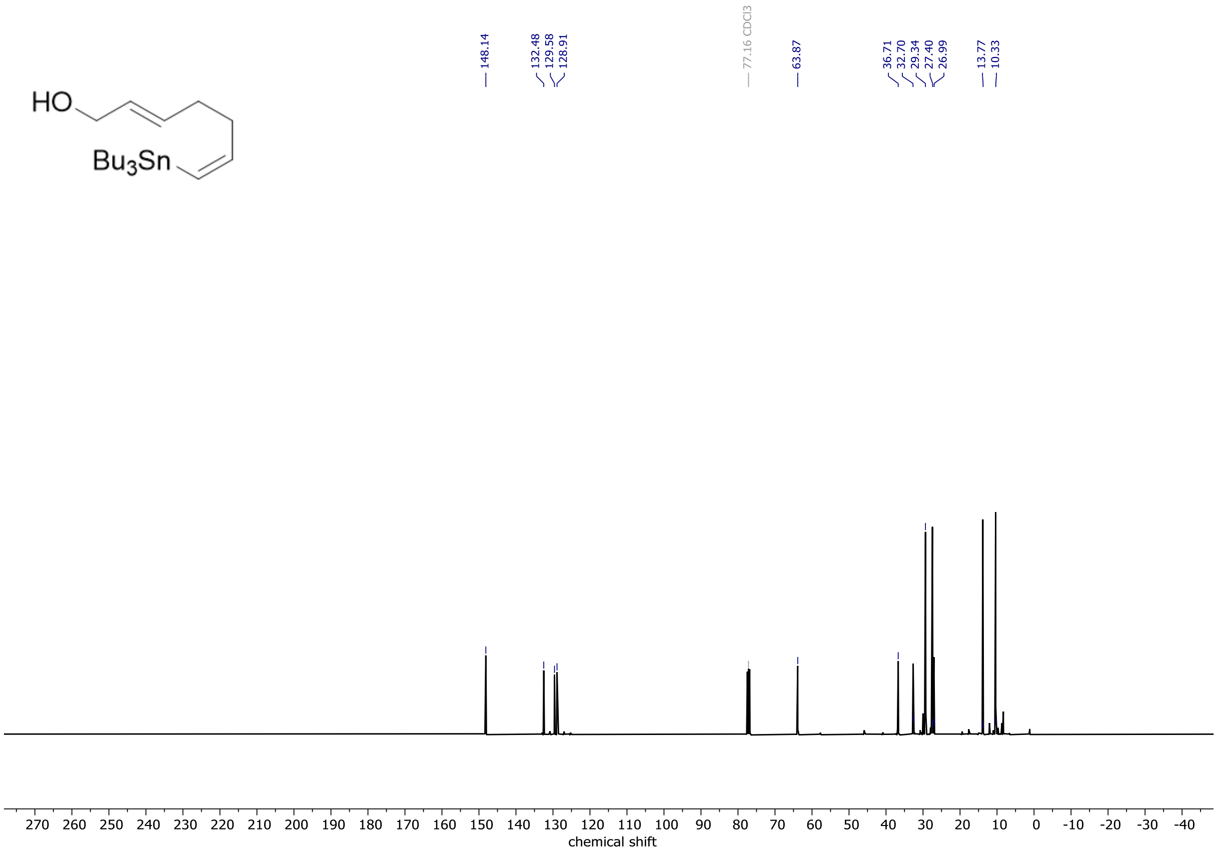

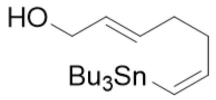

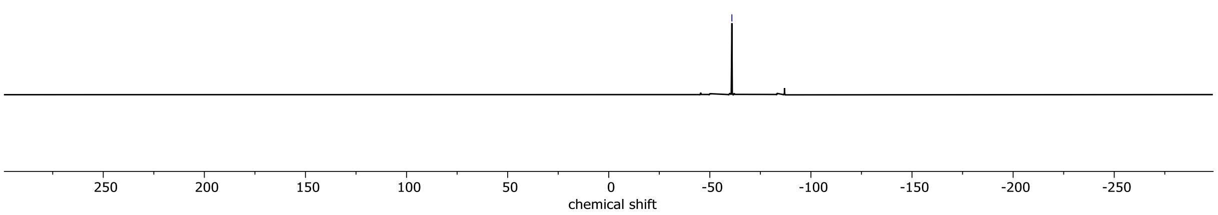

-60.91
250
200
150
100
50
0
-50
-100
-150
-200
-250
chemical shift

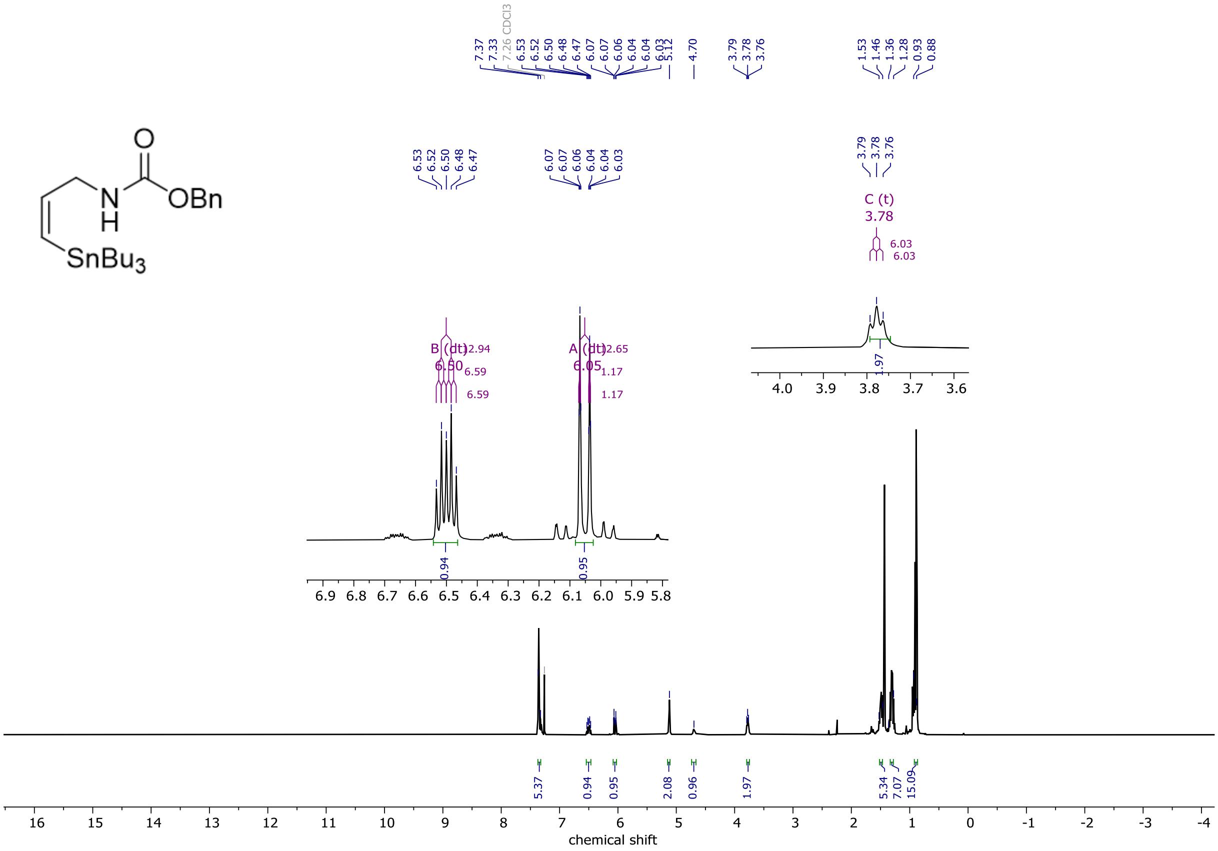

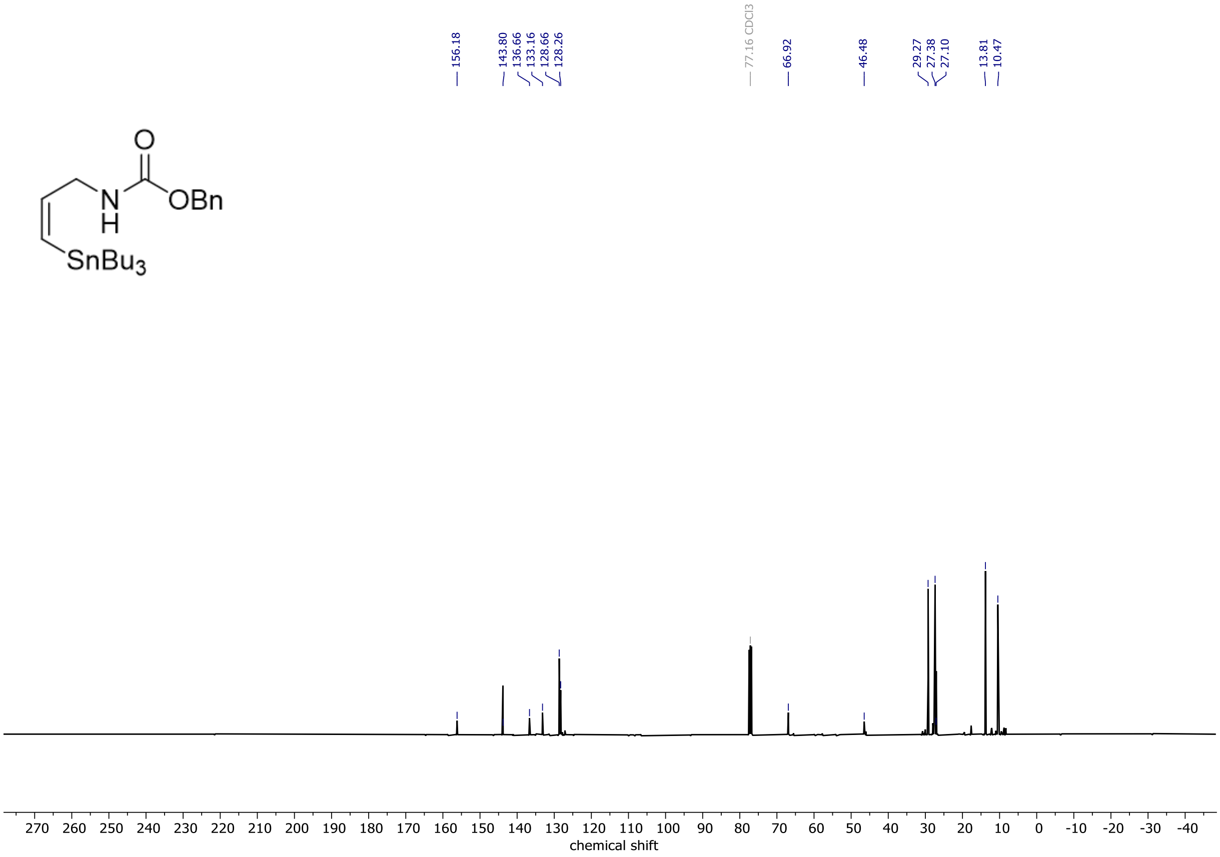

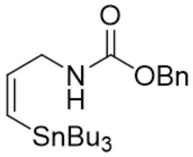

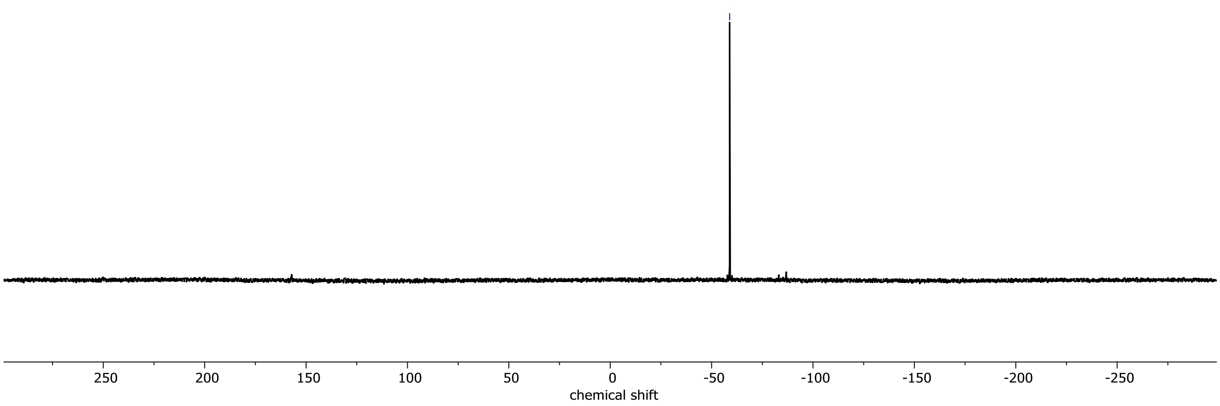

-58.89
250
200
150
100
50
0
-50
-100
-150
-200
-250
chemical shift

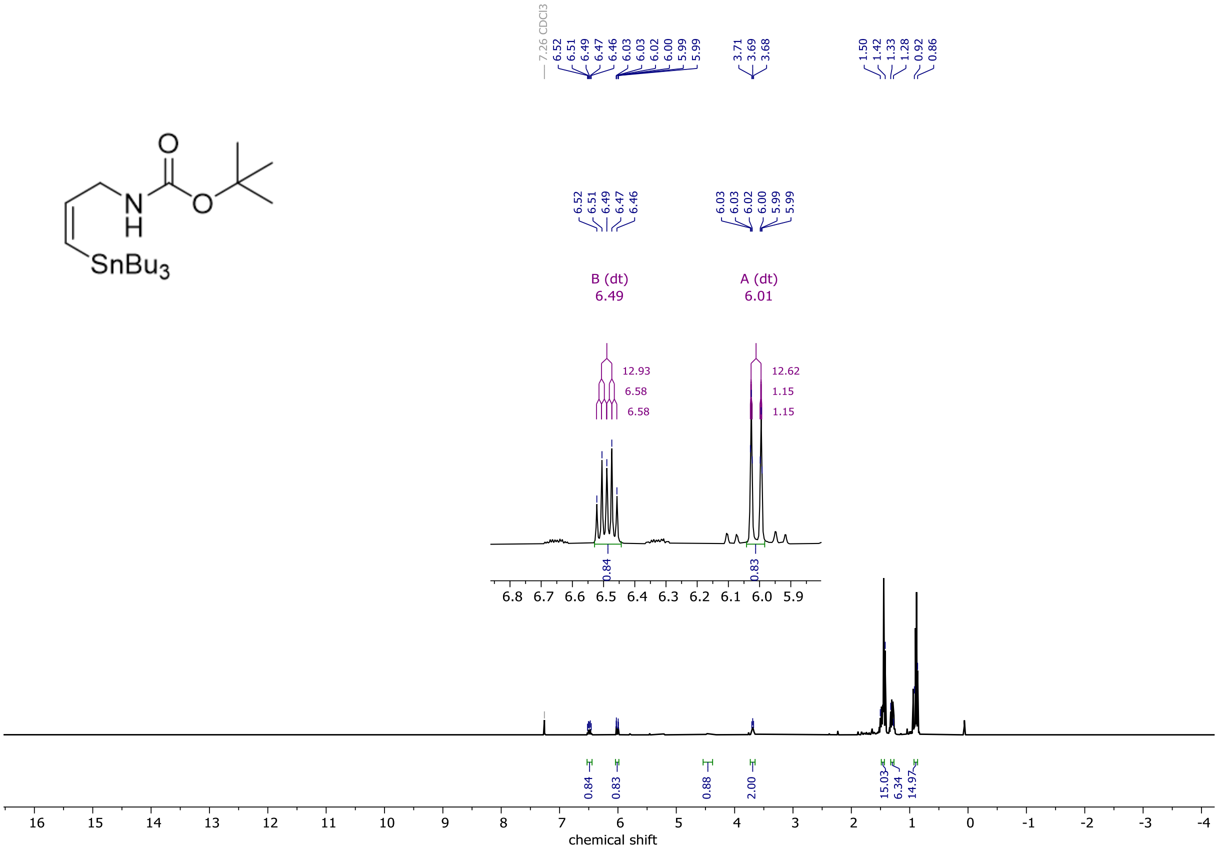

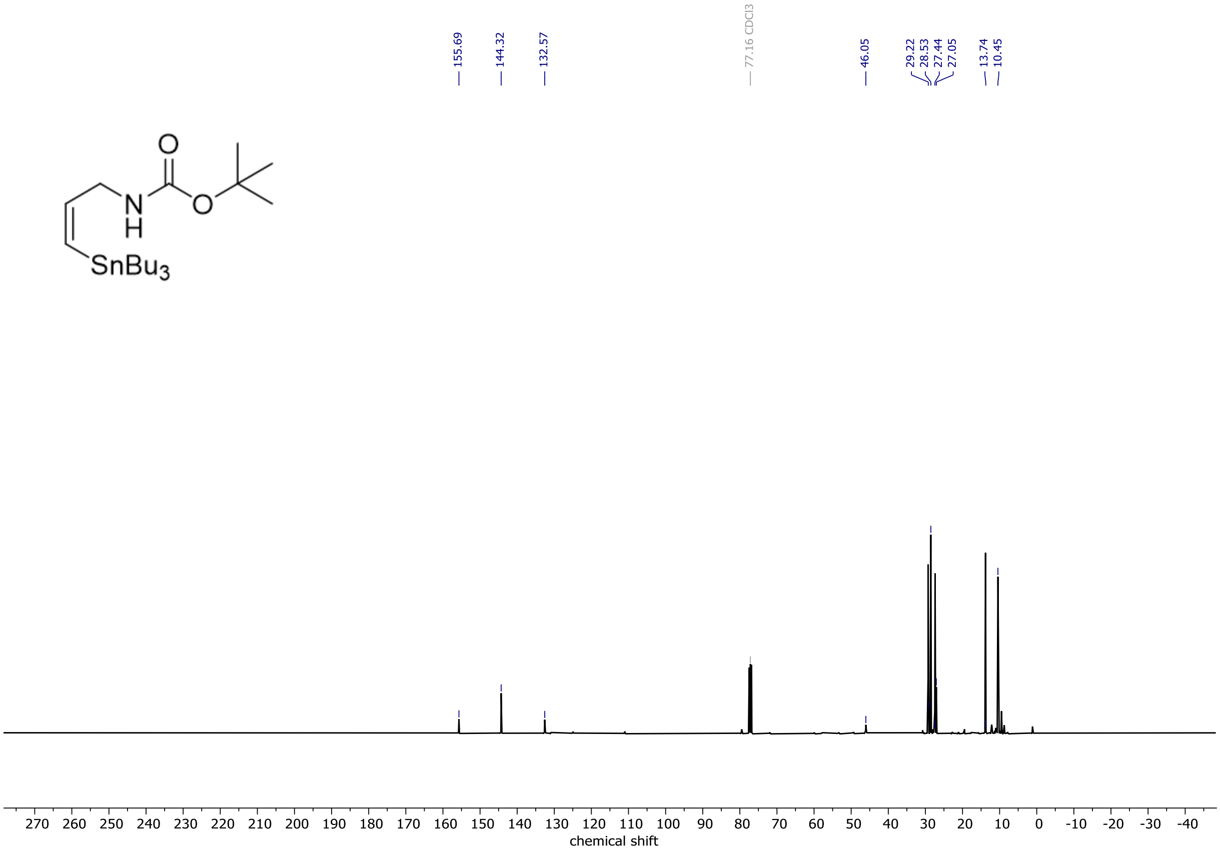

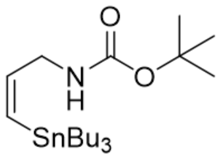

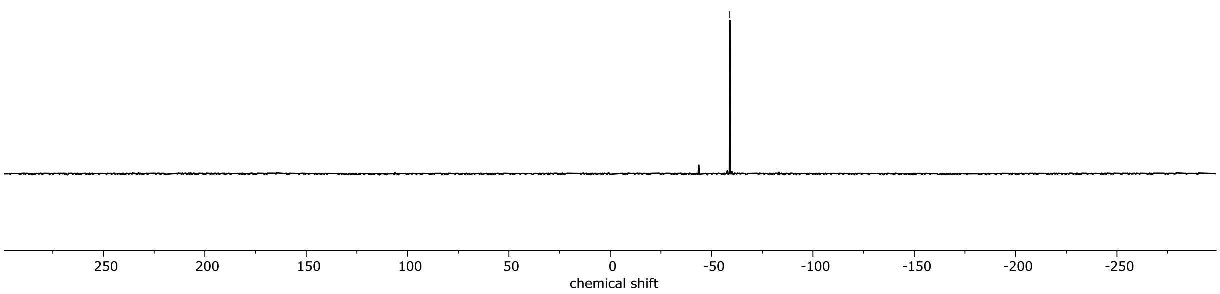
-58.99
250
200
150
100
50
0
-50
-100
-150
-200
-250
chemical shift

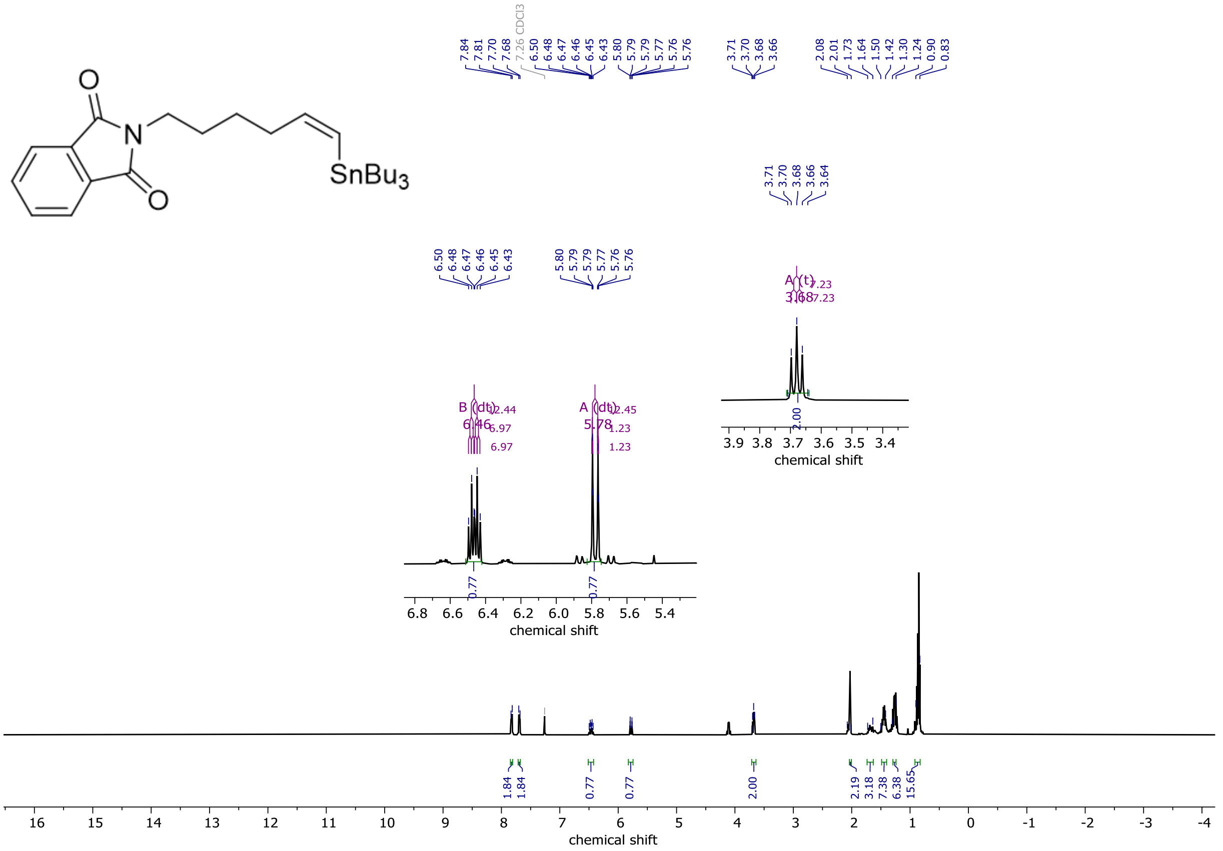

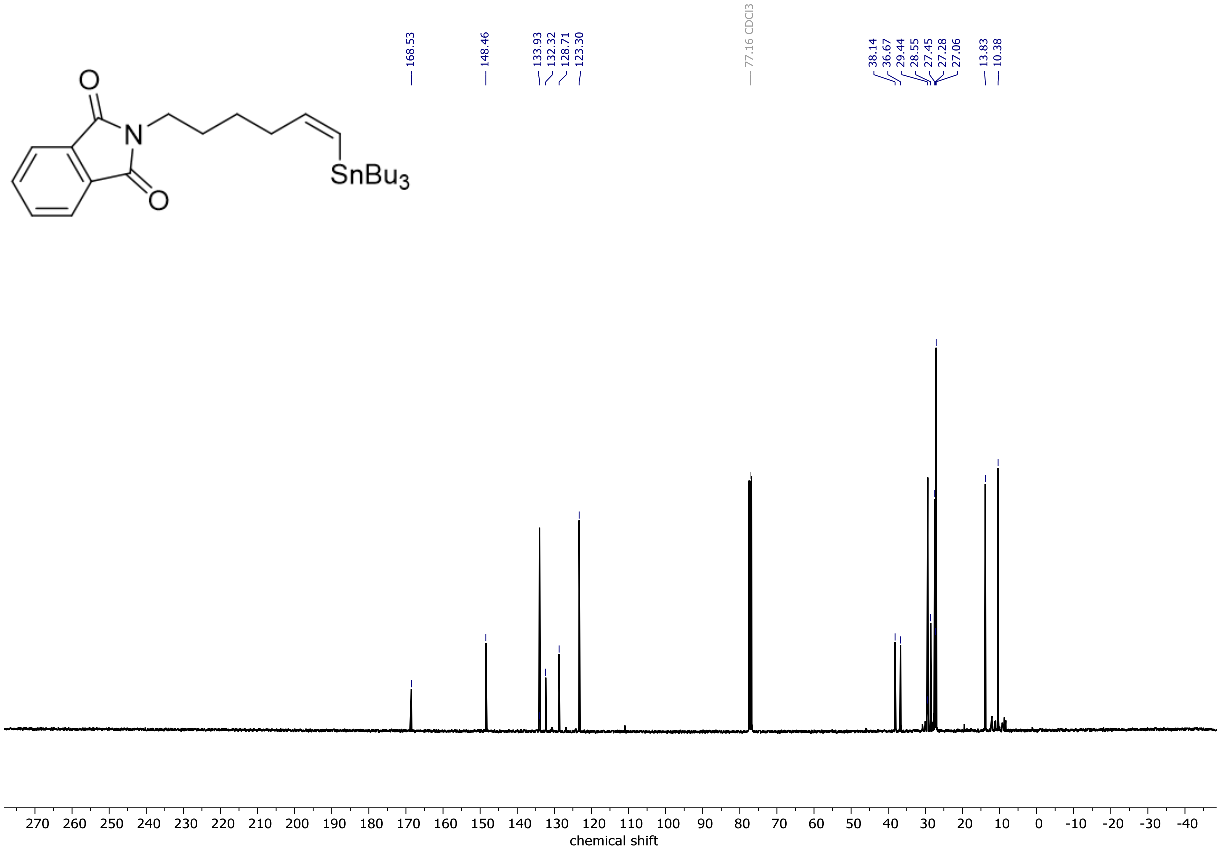

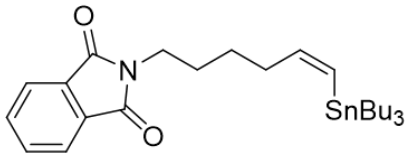

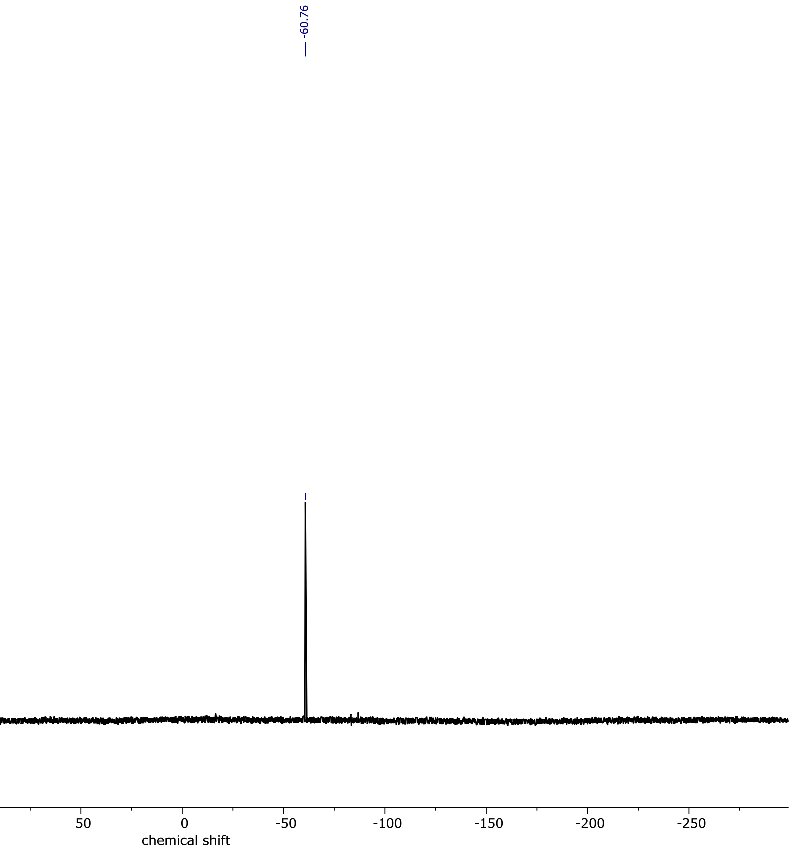

-60.76
250
200
150
100
50
0
-50
-100
-150
-200
-250
chemical shift

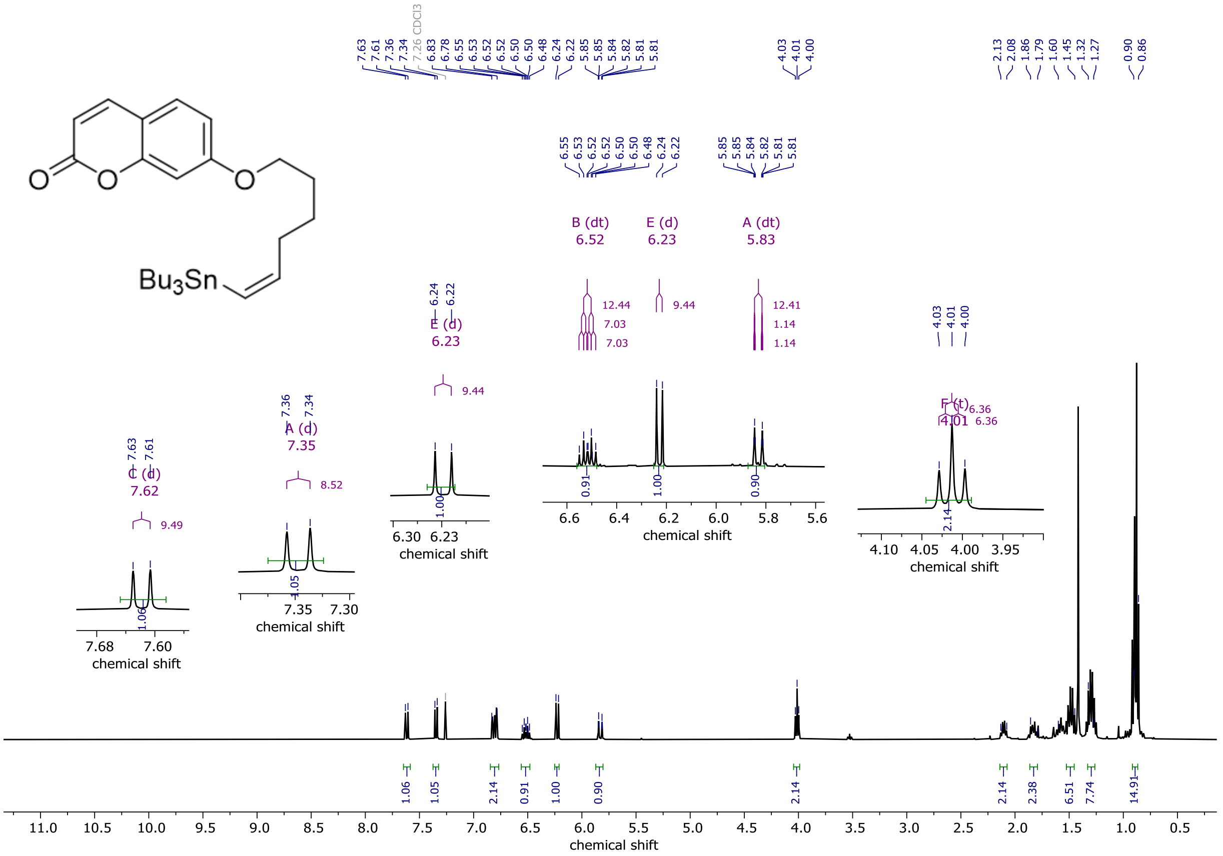

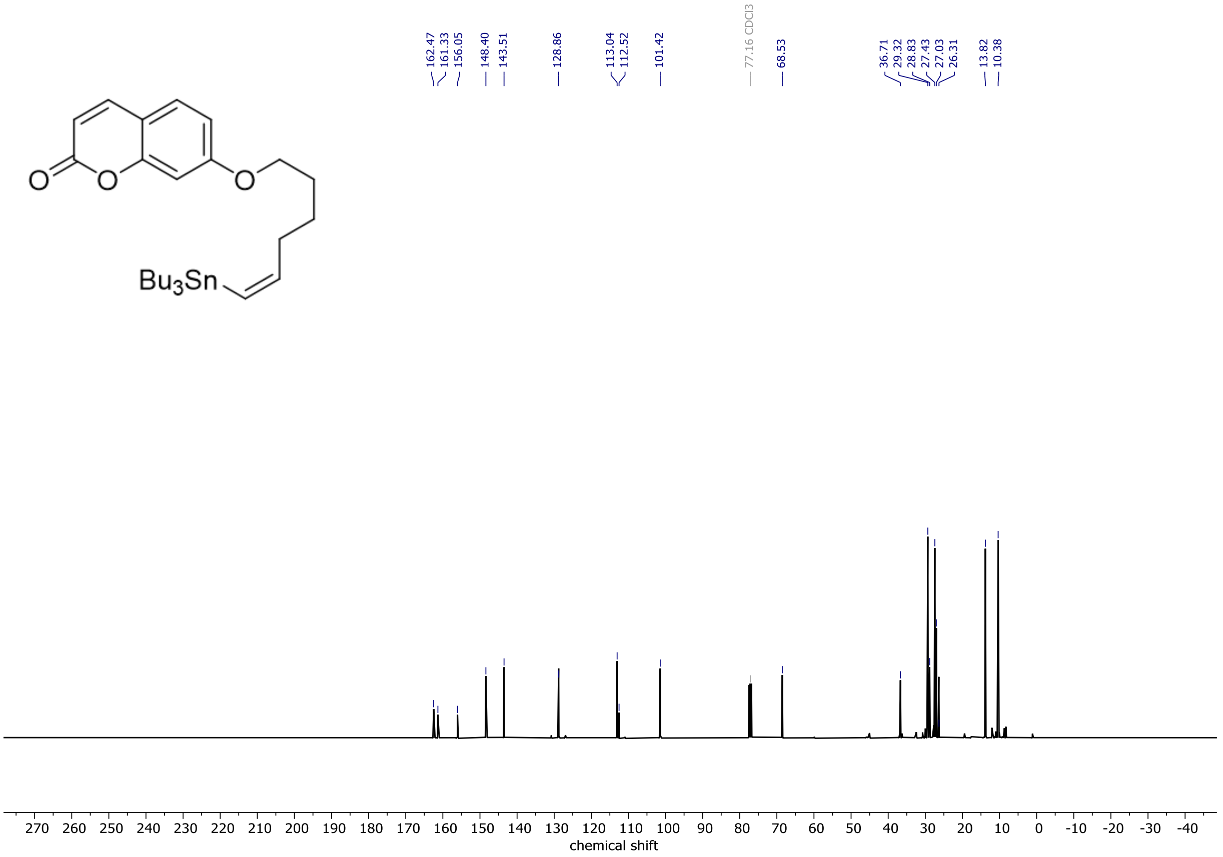

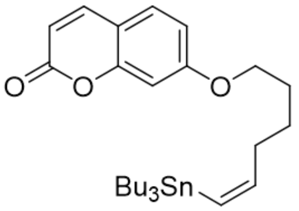
O
O
O
Bu3Sn

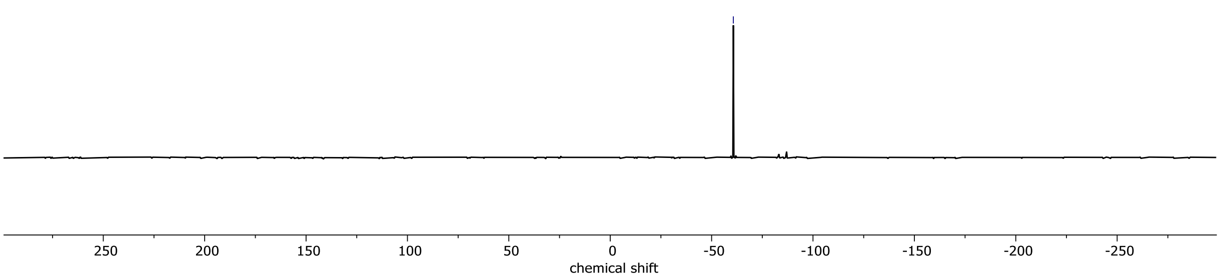
-60.71
250
200
150
100
50
0
-50
-100
-150
-200
-250
chemical shift

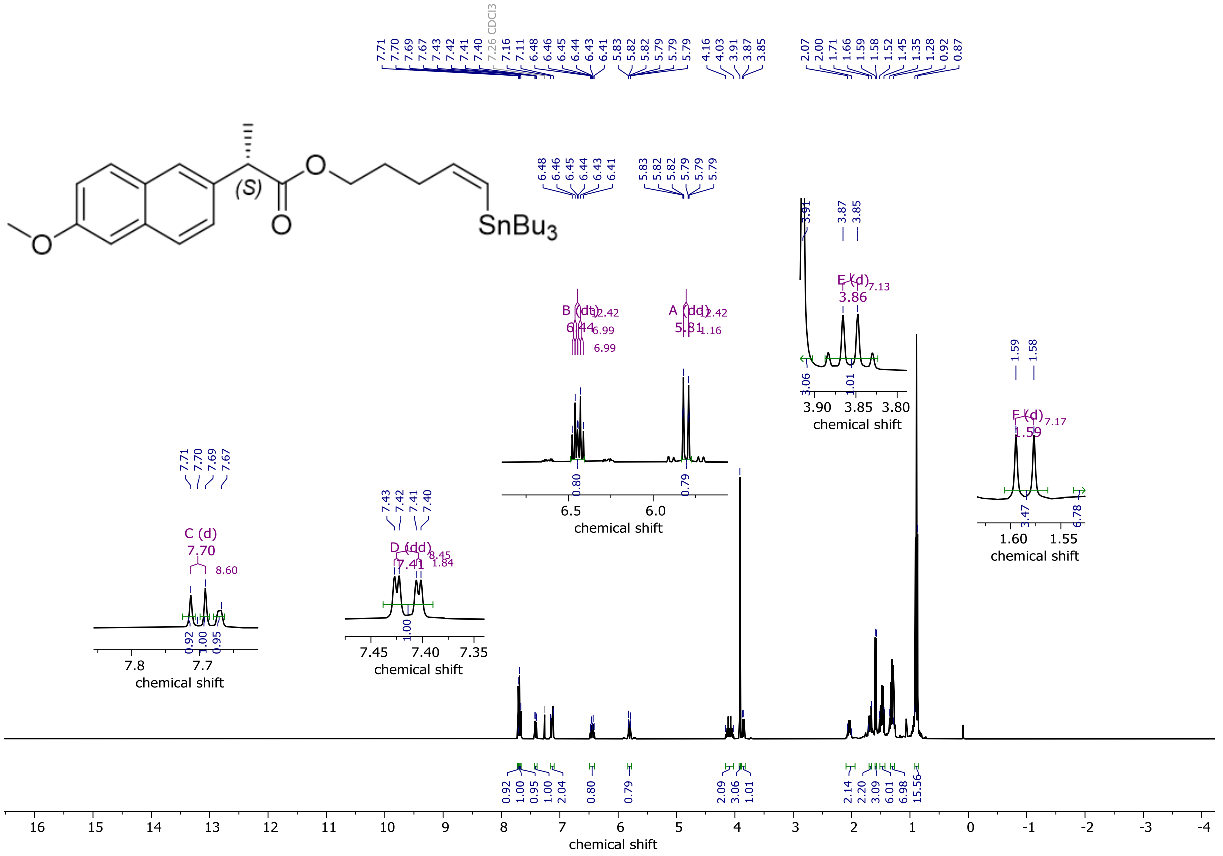

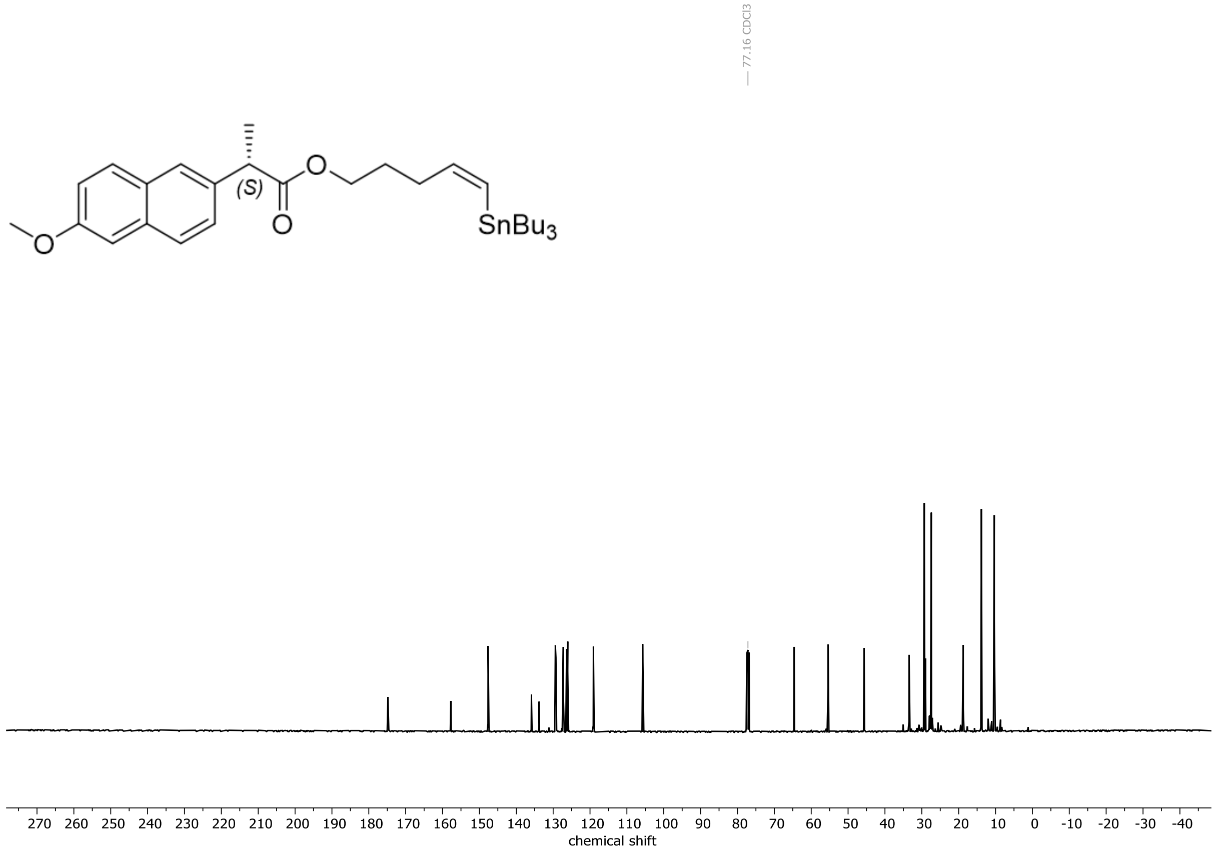

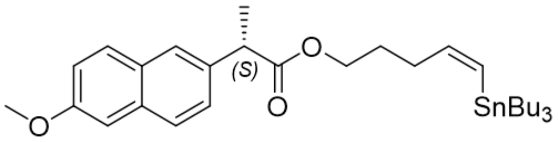
(S)
O
O
O
SnBu3

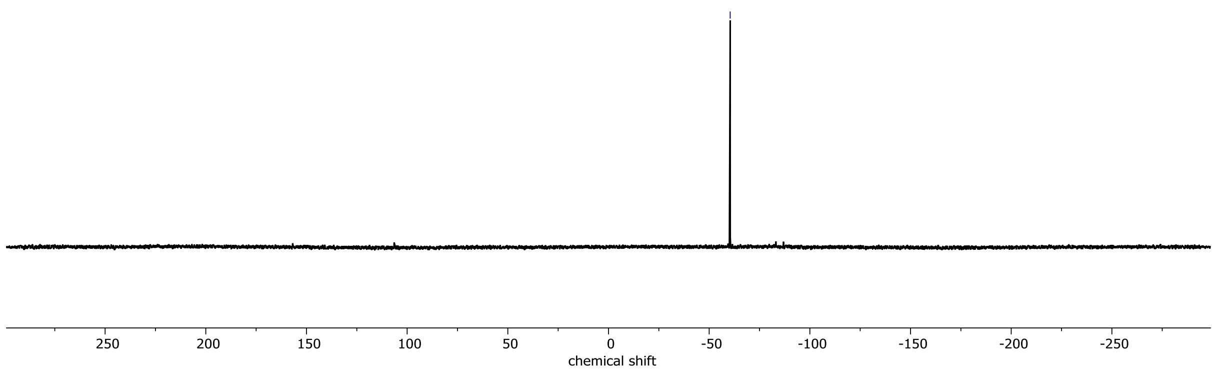
-60.43
250
200
150
100
50
0
-50
-100
-150
-200
-250
chemical shift

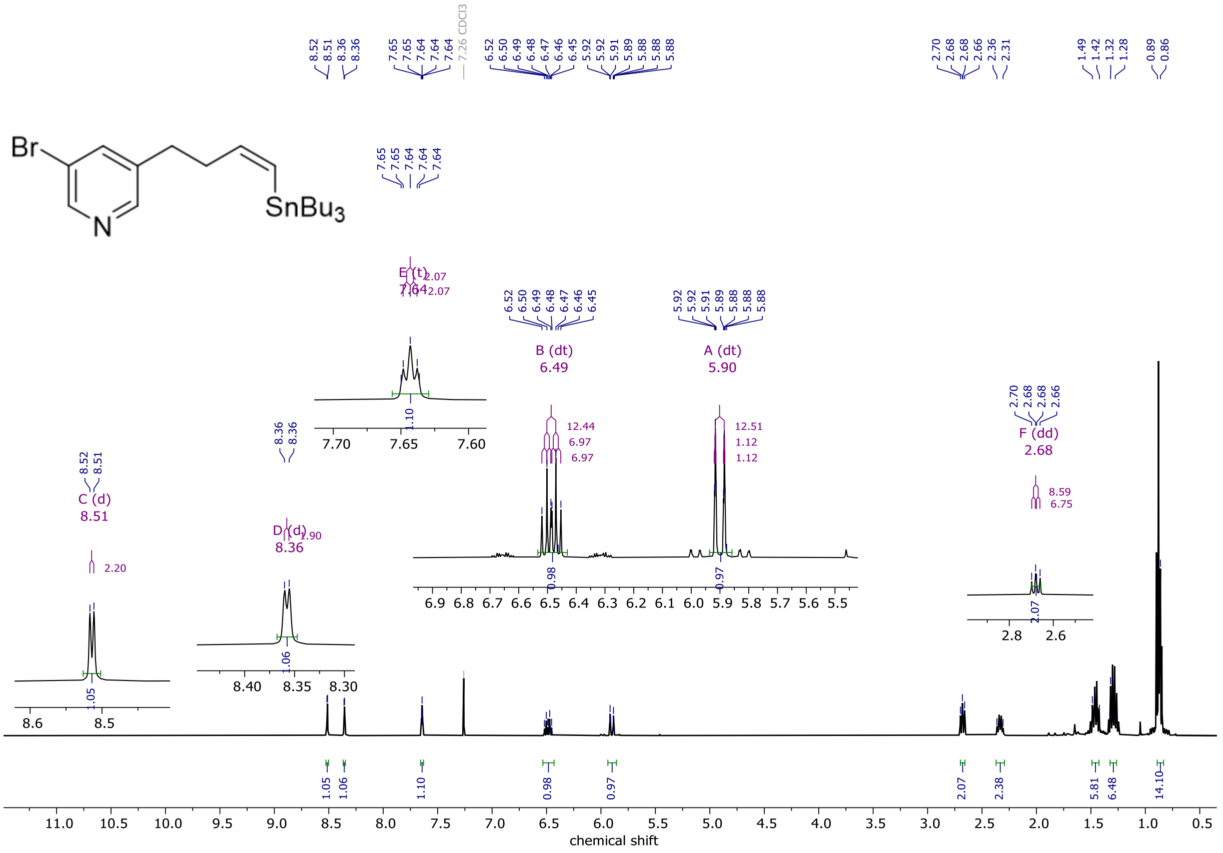
Br
N
SnBu3
8.52
8.51
8.36
8.36
7.65
7.65
7.64
7.64
7.64
7.26 CDCl3
6.52
6.50
6.49
6.48
6.47
6.46
6.45
5.92
5.92
5.91
5.89
5.88
5.88
5.88
2.70
2.68
2.68
2.66
2.36
2.31
1.49
1.42
1.32
1.28
0.89
0.86
C (d)
8.51
D (d)
8.36
B (dt)
6.49
A (dt)
5.90
F (dd)
2.68
1.05
1.06
1.10
0.98
0.97
2.07
2.38
5.81
6.48
14.10
chemical shift

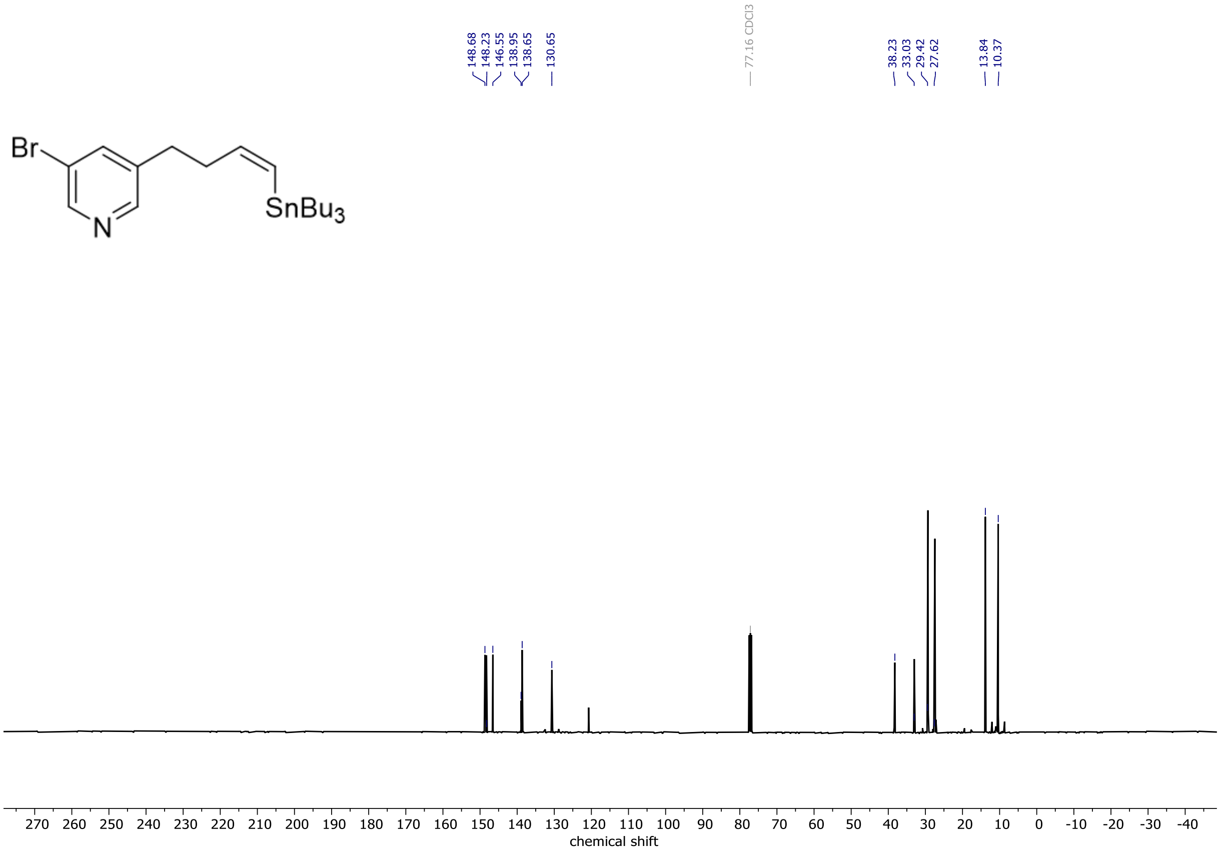
148.68
148.23
146.55
138.95
138.65
130.65
77.16 CDCl3
38.23
33.03
29.42
27.62
13.84
10.37
Br
N
SnBu3
chemical shift

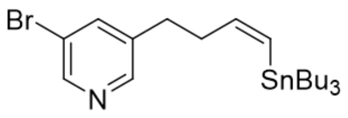
Br
N
SnBu3

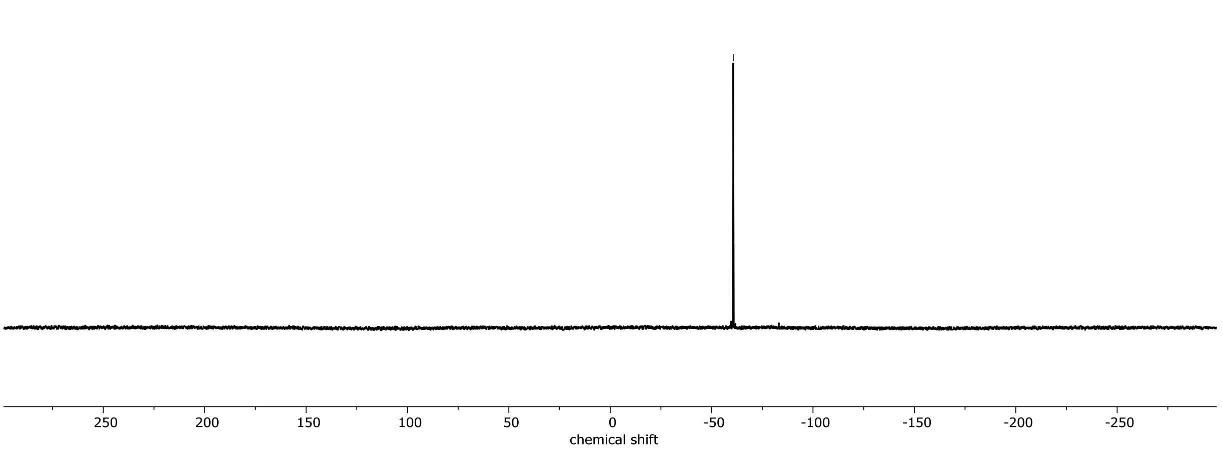
-60.67
250
200
150
100
50
0
-50
-100
-150
-200
-250
chemical shift

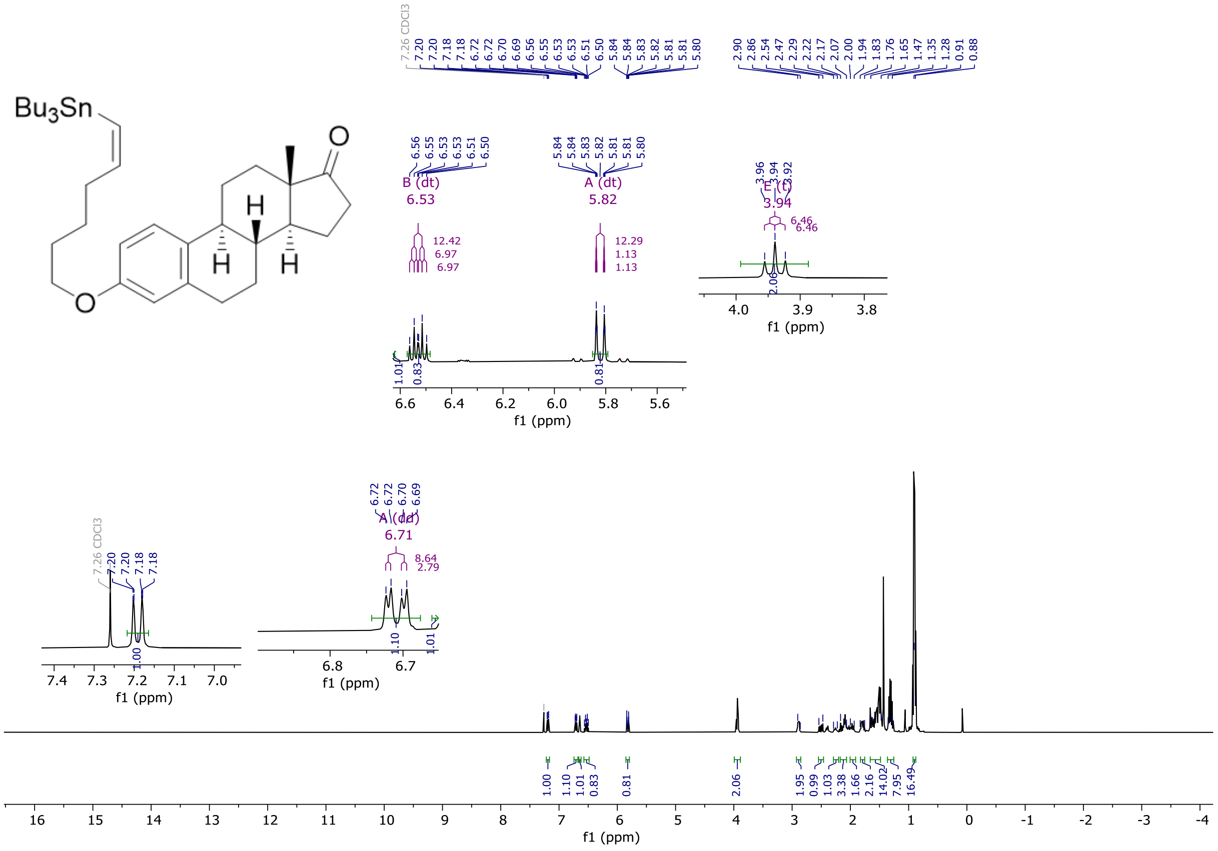

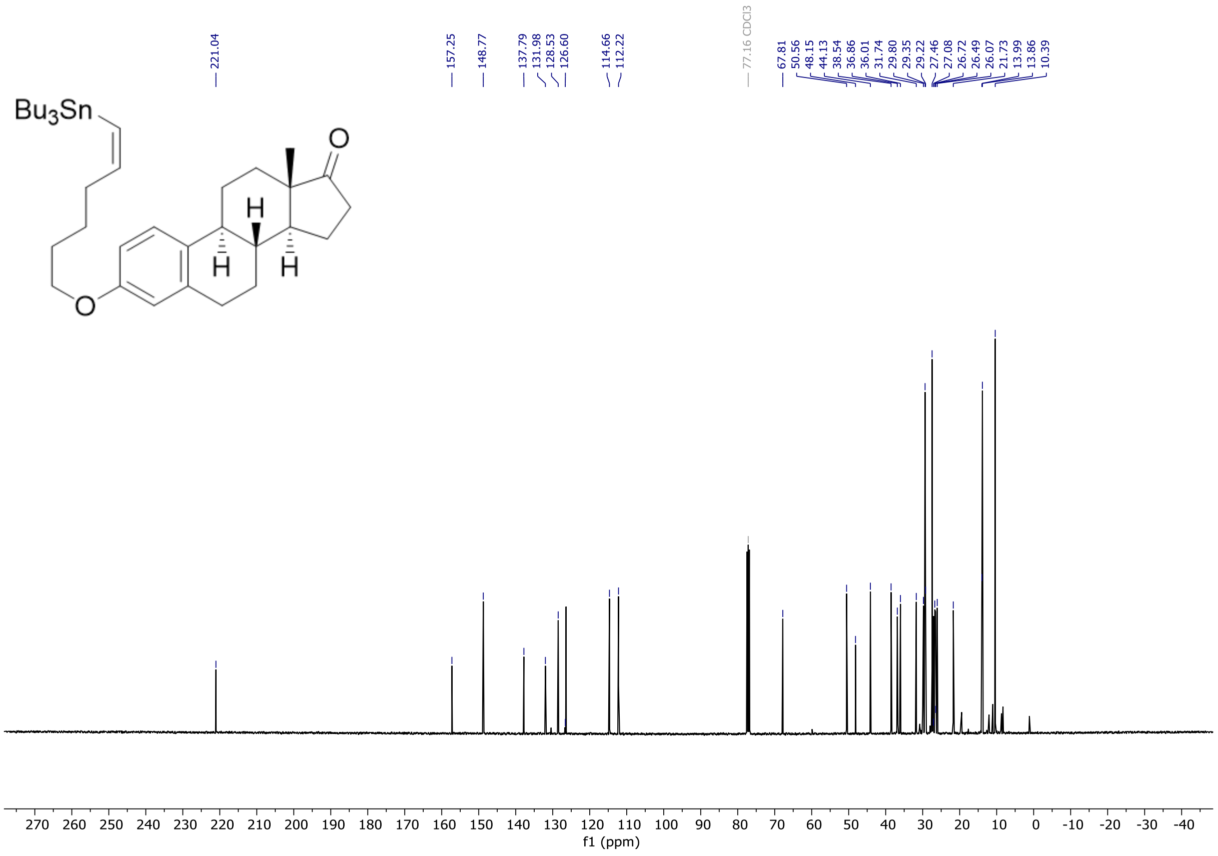

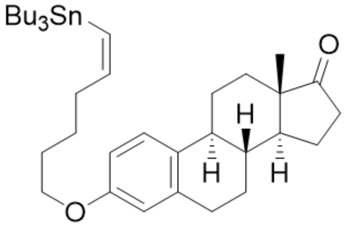
Bu3Sn
O
H
H
H
O

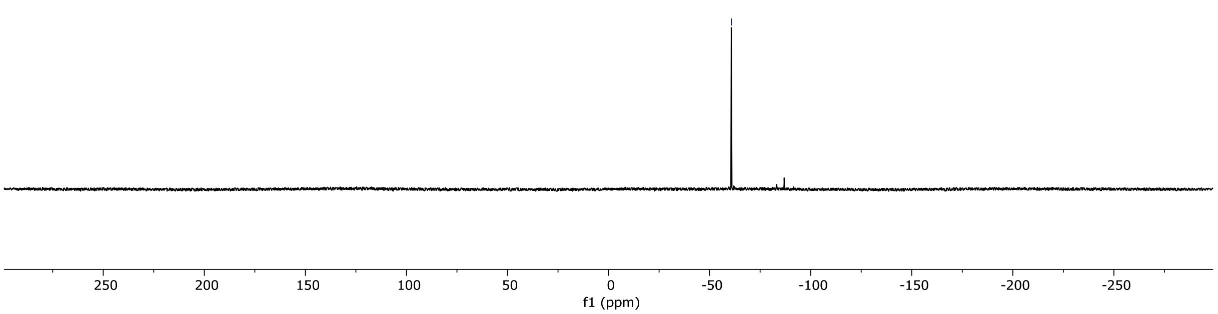
-60.73
250
200
150
100
50
0
-50
-100
-150
-200
-250
f1 (ppm)

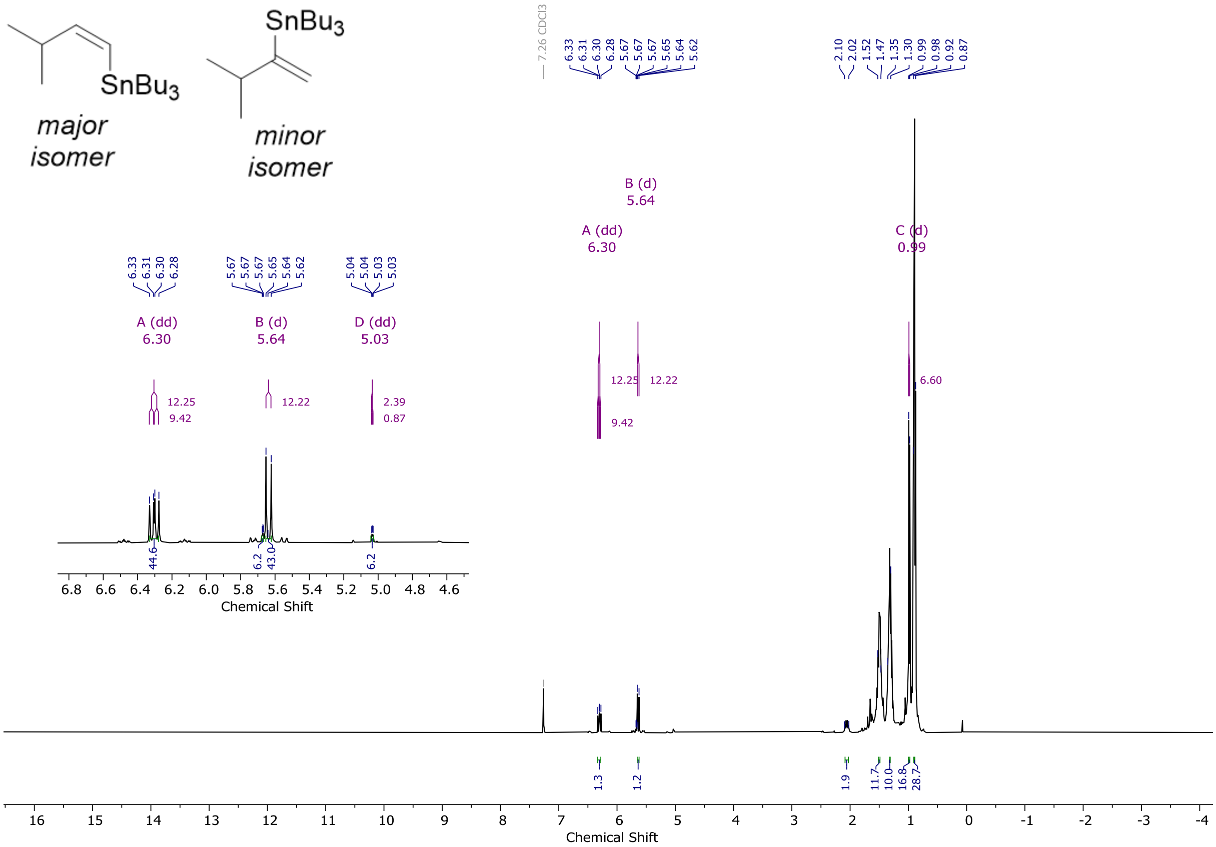
SnBu3
SnBu3
major isomer
minor isomer
7.26 CDCl3
6.33 6.31 6.30 6.28 5.67 5.67 5.67 5.65 5.64 5.62
2.10 2.02 1.52 1.47 1.35 1.30 0.99 0.98 0.92 0.87
A (dd) 6.30
B (d) 5.64
C (d) 0.99
D (dd) 5.03
12.25
9.42
12.22
2.39
0.87
6.60
5.04 5.04 5.03 5.03
44.6
6.2
43.0
6.2
1.3
1.2
1.9
11.7
10.0
16.8
28.7
Chemical Shift
Chemical Shift

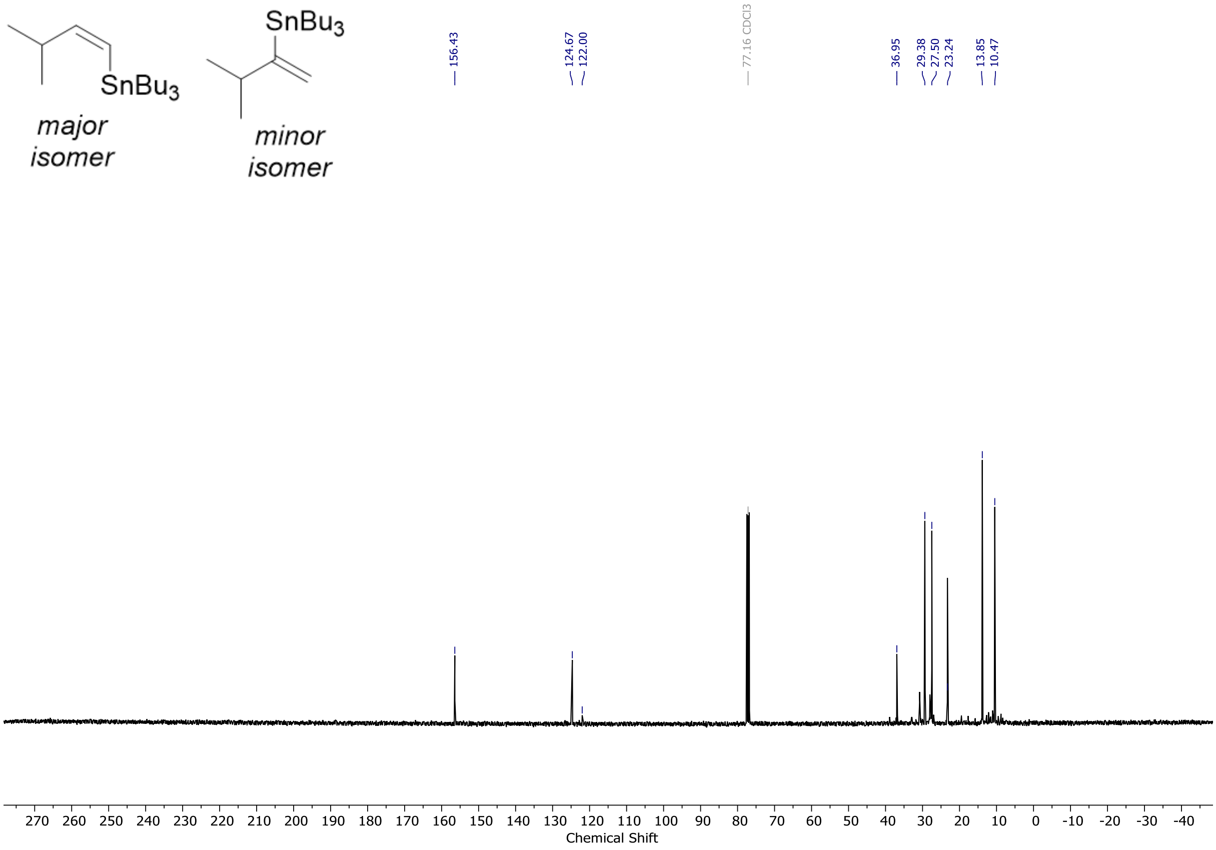
SnBu3
SnBu3
major isomer
minor isomer
156.43
124.67
122.00
77.16 CDCl3
36.95
29.38
27.50
23.24
13.85
10.47
Chemical Shift

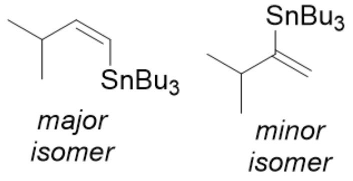
SnBu3
major
isomer
SnBu3
minor
isomer

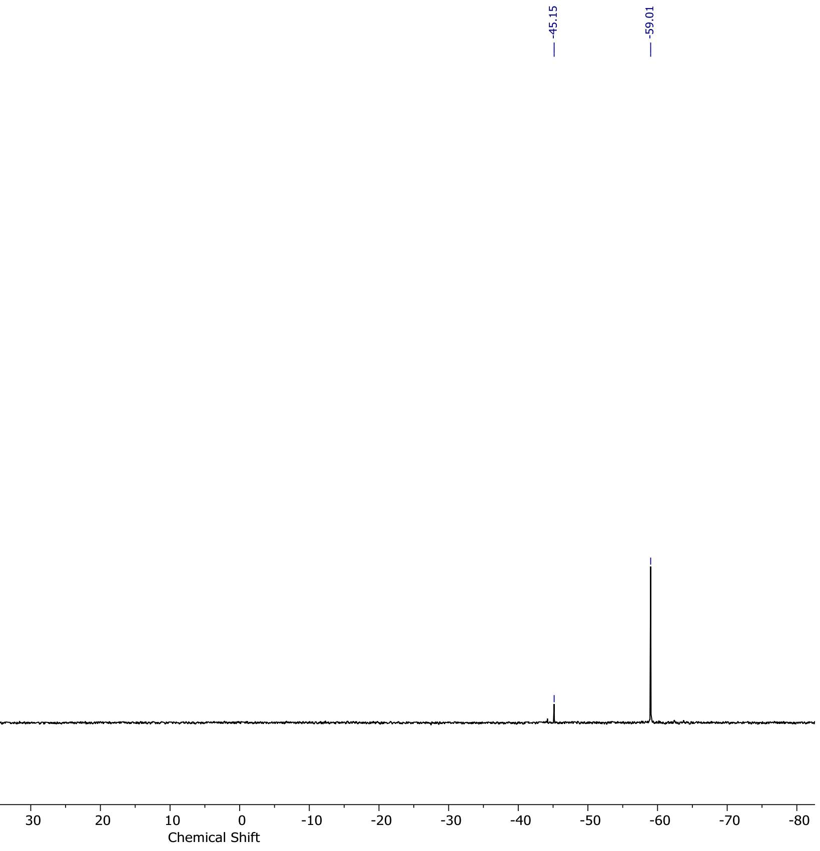
-45.15
-59.01
90
80
70
60
50
40
30
20
10
0
-10
-20
-30
-40
-50
-60
-70
-80
Chemical Shift

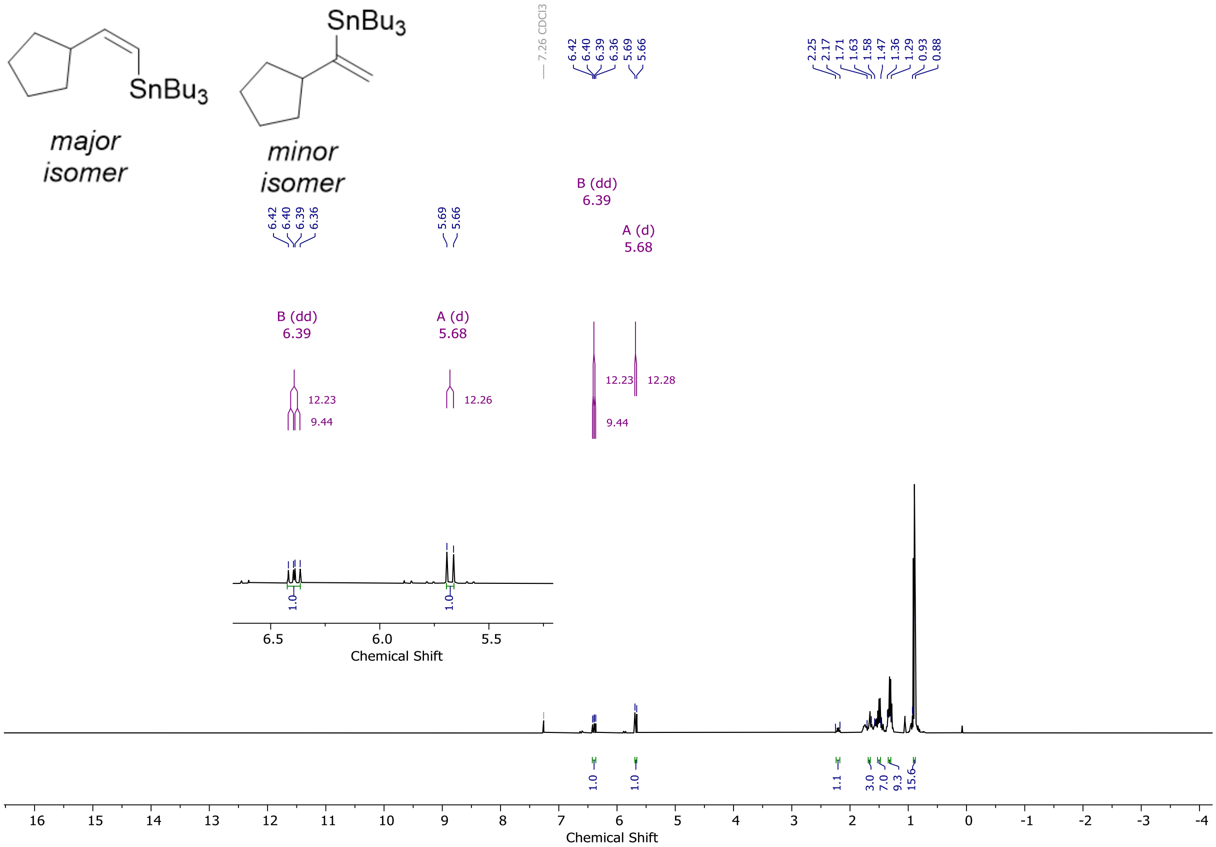
SnBu3
SnBu3
major isomer
minor isomer
7.26 CDCl3
6.42
6.40
6.39
6.36
5.69
5.66
2.25
2.17
1.71
1.63
1.58
1.47
1.36
1.29
0.93
0.88
B (dd)
6.39
A (d)
5.68
12.23
9.44
12.26
12.28
1.0
1.0
1.1
3.0
7.0
9.3
15.6
Chemical Shift
Chemical Shift

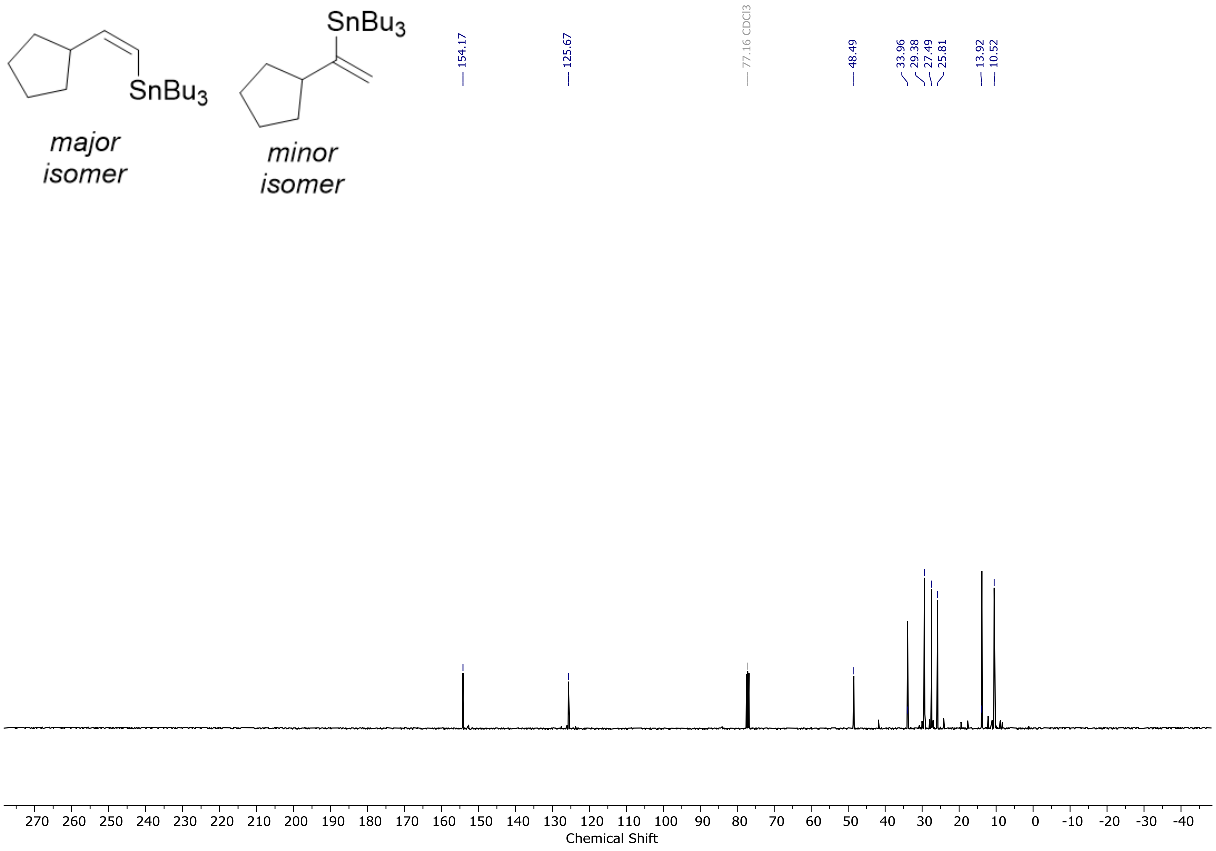
SnBu3
SnBu3
major isomer
minor isomer
154.17
125.67
77.16 CDCl3
48.49
33.96
29.38
27.49
25.81
13.92
10.52
Chemical Shift

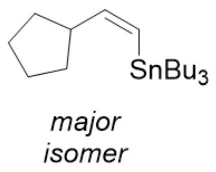
SnBu3
major isomer

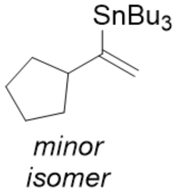
SnBu3
minor isomer

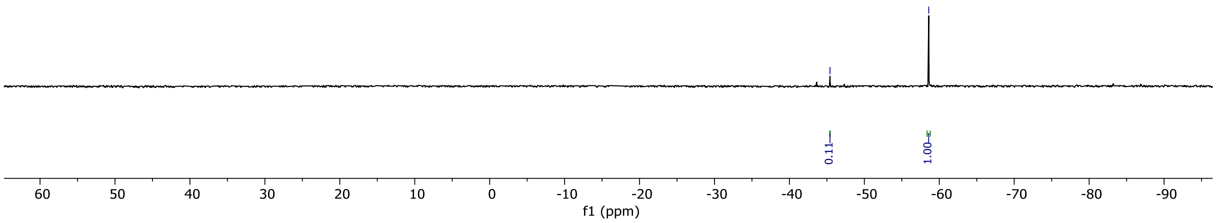
-45.42
-58.59
0.11
1.00
60
50
40
30
20
10
0
-10
-20
-30
-40
-50
-60
-70
-80
-90
f1 (ppm)

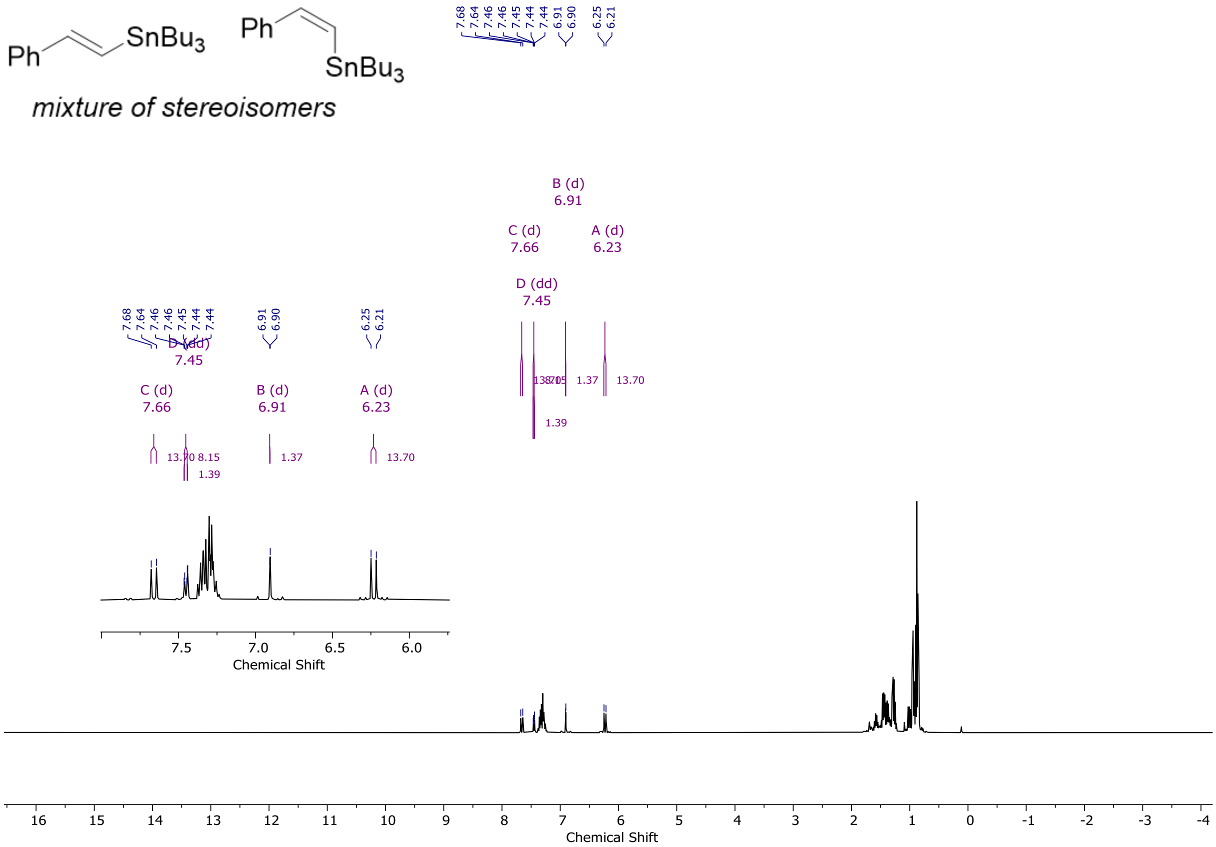
Ph SnBu3 Ph SnBu3
mixture of stereoisomers
7.68 7.64 7.46 7.46 7.45 7.44 7.44 6.91 6.90 6.25 6.21
B (d) 6.91
C (d) 7.66
A (d) 6.23
D (dd) 7.45
1.37
13.70
1.39
8.15
Chemical Shift

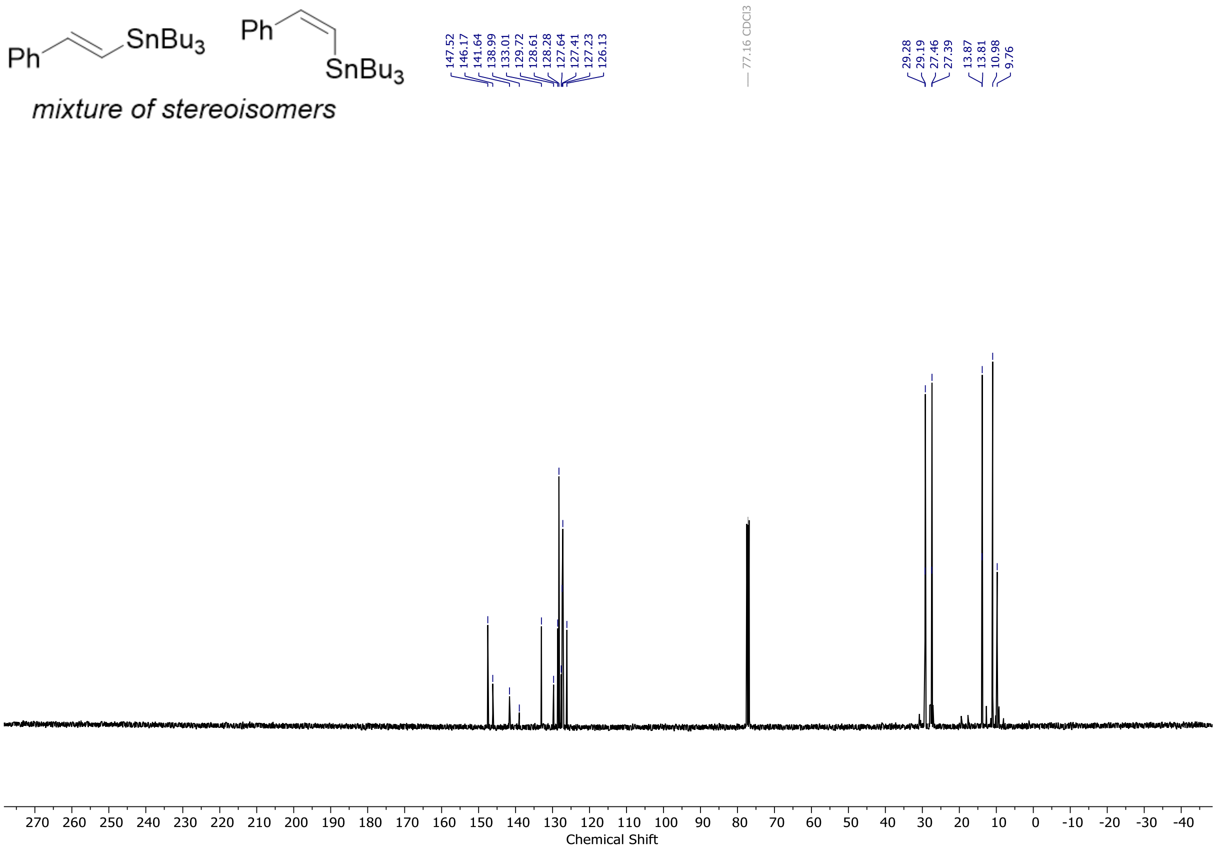
Ph SnBu3 Ph SnBu3
mixture of stereoisomers
147.52 146.17 141.64 138.99 133.01 129.72 128.61 128.28 127.64 127.41 127.23 126.13
77.16 CDCl3
29.28 29.19 27.46 27.39 13.87 13.81 10.98 9.76
Chemical Shift

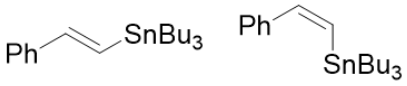

mixture of stereoisomers

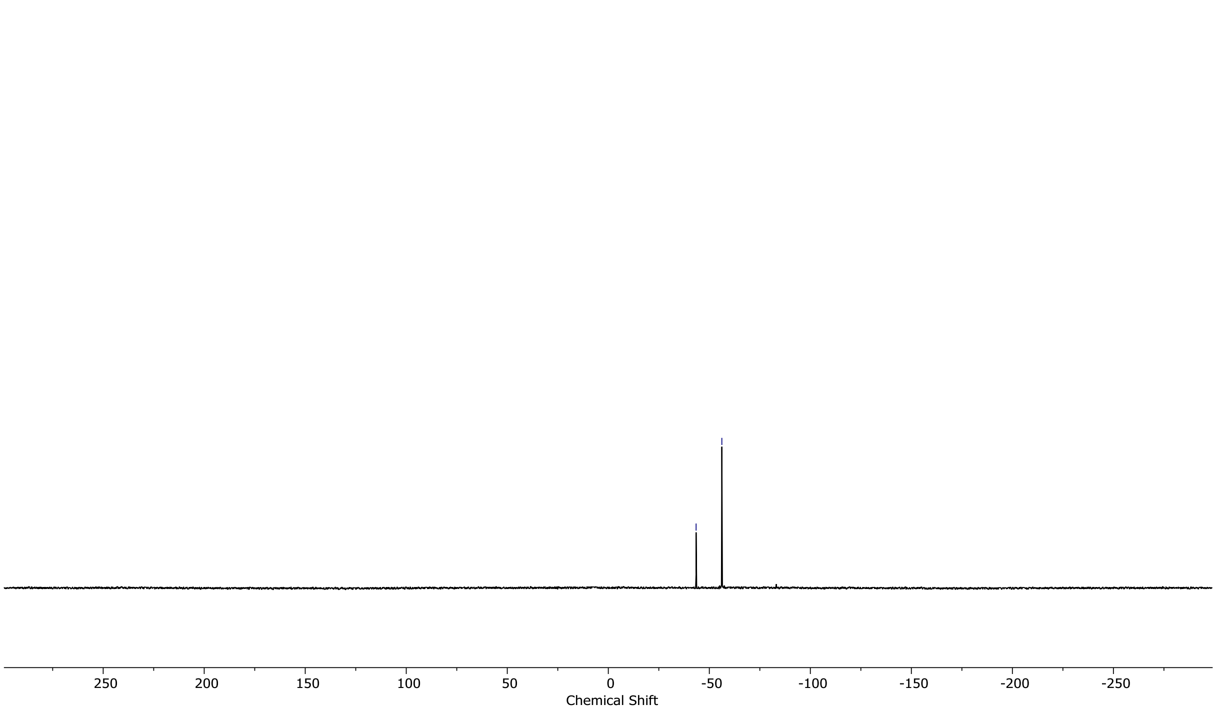

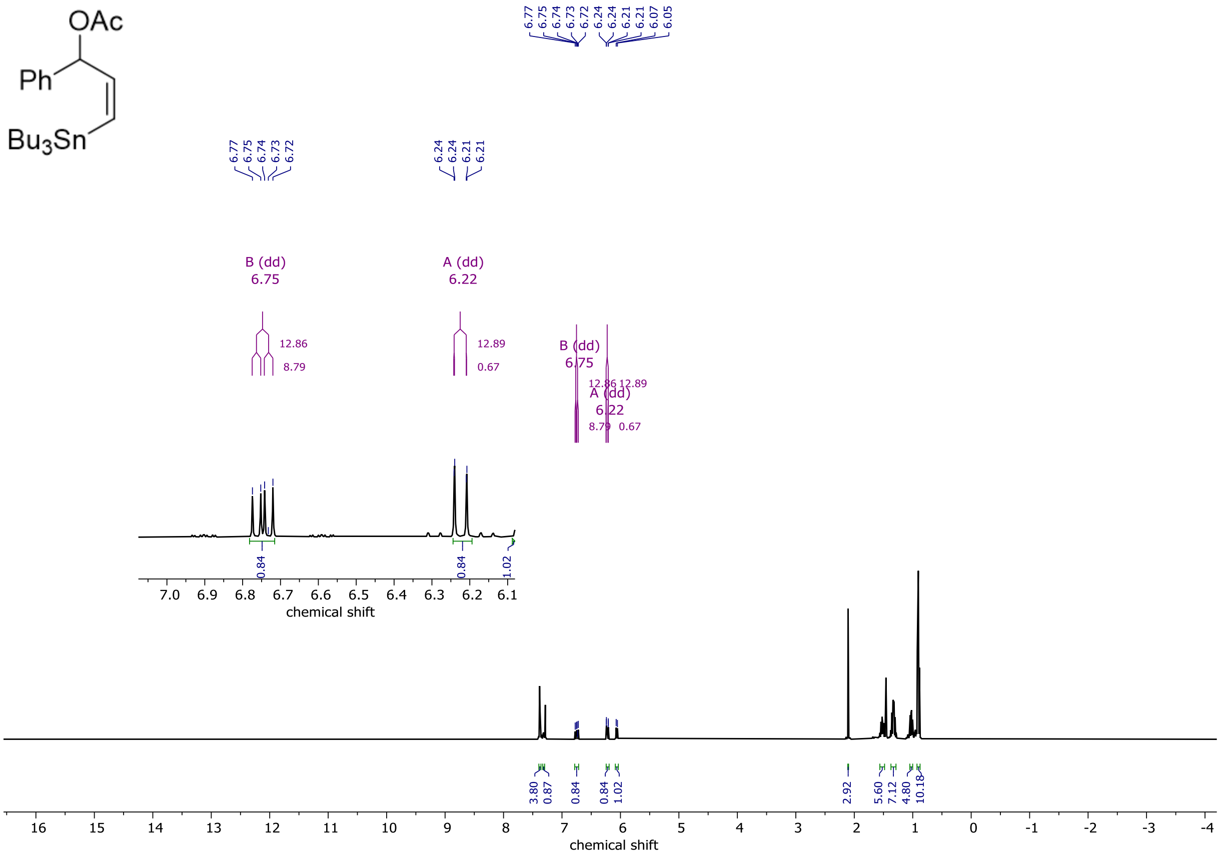
OAc
Ph
Bu3Sn
B (dd)
6.75
A (dd)
6.22
12.86
8.79
12.89
0.67
chemical shift

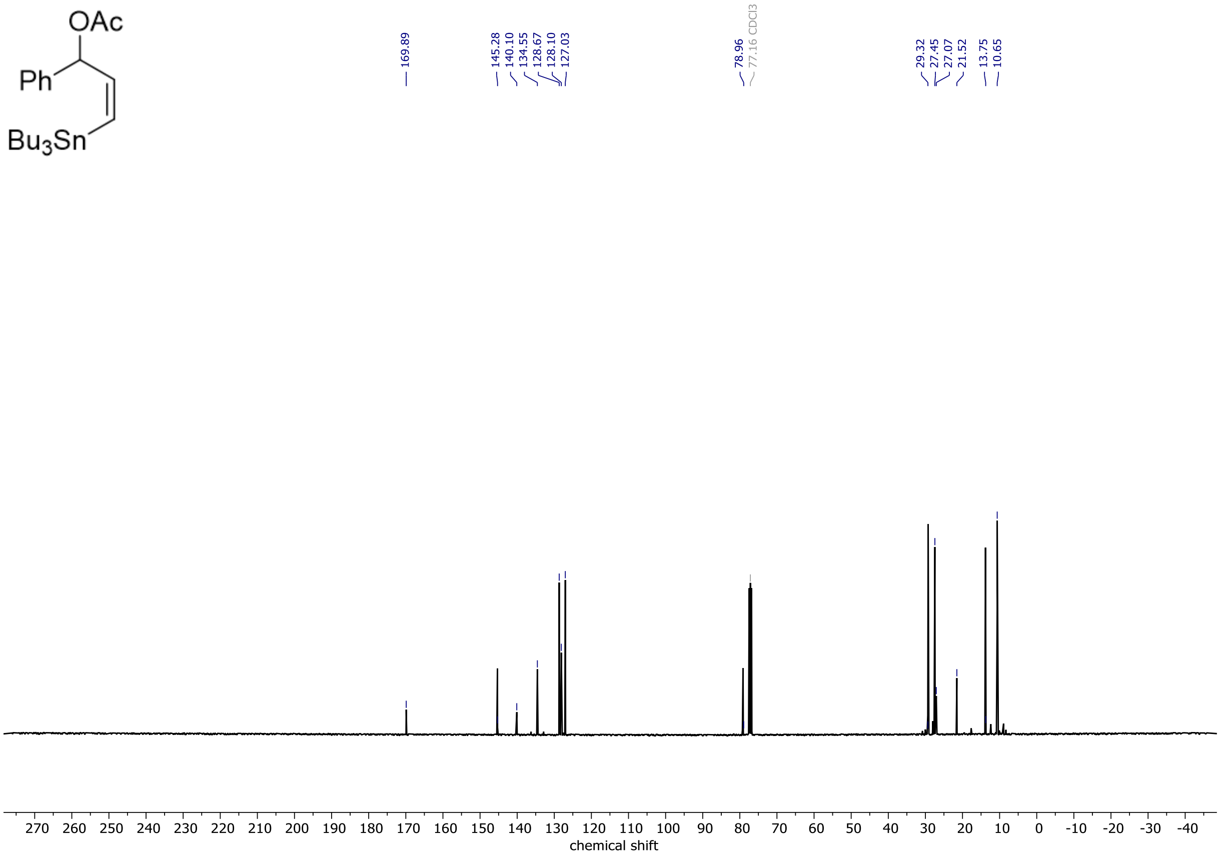
OAc
Ph
Bu3Sn
169.89
145.28
140.10
134.55
128.67
128.10
127.03
78.96
77.16 CDCl3
29.32
27.45
27.07
21.52
13.75
10.65
chemical shift

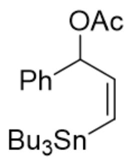
OAc
Ph
Bu3Sn

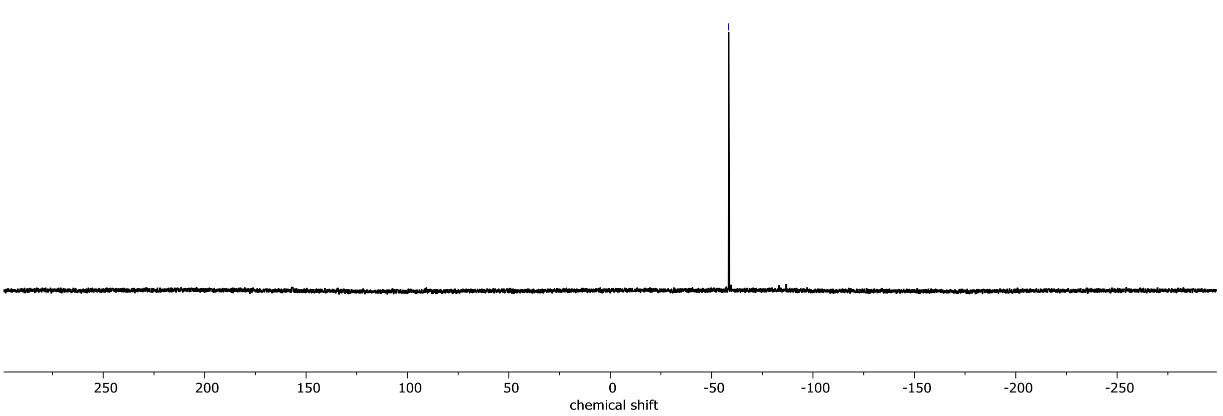
-58.42
250
200
150
100
50
0
-50
-100
-150
-200
-250
chemical shift

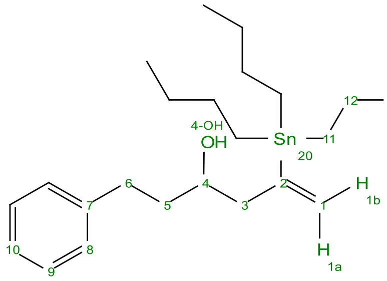

Atom	δ (ppm)	J	COSY	HSQC	HMBC	NOESY
1 C	129.05			1a, 1b	3a, 3b	
1a H	5.82	2.73(1b), 1.28(?), 1.28(?)	1b, 3a, 3b	1	2, 3, 20	1b, 3a, 3b, 4-OH
1b H	5.34	2.73(1a)	1a, 3a, 3b	1	2, 3, 20	1a, 11, 12
2 C	152.44				1a, 1b, 3a, 3b, 11	
3 C	49.89			3a, 3b	1a, 1b, 4-OH, 5	
Ha	2.55	13.30(3b), 3.43(?), 1.23(?), 1.23(?)	1a, 1b, 3b, 4	3	1, 2, 4, 5, 20	1a, 3b, 4, 4-OH, 5, 11
Hb	2.33	13.30(3a), 9.22(?)	1a, 1b, 3a, 4	3	1, 2, 4, 5, 20	1a, 3a, 4, 4-OH
4 C	69.12			4	3a, 3b, 4-OH, 5, 6a, 6b	
H	3.59	2.22(4-OH)	3a, 3b, 4-OH, 5	4		3a, 3b, 4-OH, 5, 6b, 11
4-OH O						
H	1.77	2.22(4)	4		3, 4, 5, 6	1a, 3a, 3b, 4, 11
5 C	38.64			5	3a, 3b, 4-OH, 6a, 6b	
H2	1.82		4, 6a, 6b	5	3, 4, 6, 7	3a, 4, 8
6 C	32.18			6a, 6b	4-OH, 5, 8	
Ha	2.84	14.59(?), 9.49(?), 5.56(?)	5, 6b	6	4, 5, 7, 8	8
Hb	2.73	13.68(?), 9.31(?), 7.08(?)	5, 6a	6	4, 5, 7, 8	4, 8

Atom	δ (ppm)	J	COSY	HSQC	HMBC	NOESY
7 C	142.18				5, 6a, 6b, 9	
8 C	128.57			8	6a, 6b, 8, 9, 10	
H	7.24		9	8	6, 8, 9, 10	5, 6a, 6b
9 C	128.47			9	8, 9, 10	
H	7.30		8, 10	9	7, 8, 9	13
10 C	125.89			10	8	
H	7.20		9	10	8, 9	
11 C	9.81			11	12, 13, 14	
H2	0.90		12	11	2, 12, 13, 20	1b, 3a, 4, 4-OH, 12, 13
12 C	29.19			12	11, 13, 14	
H2	1.49		11, 13	12	11, 13, 14, 20	1b, 11
13 C	27.49			13	11, 12, 14	
H2	1.33	7.22(14), 7.29(?), 7.29(?)	12, 14	13	11, 12, 14	9, 11
14 C	13.80			14	12, 13	
H3	0.92	7.22(13)	13	14	11, 12, 13	
20 Sn	-44.68				1a, 1b, 3a, 3b, 11, 12	

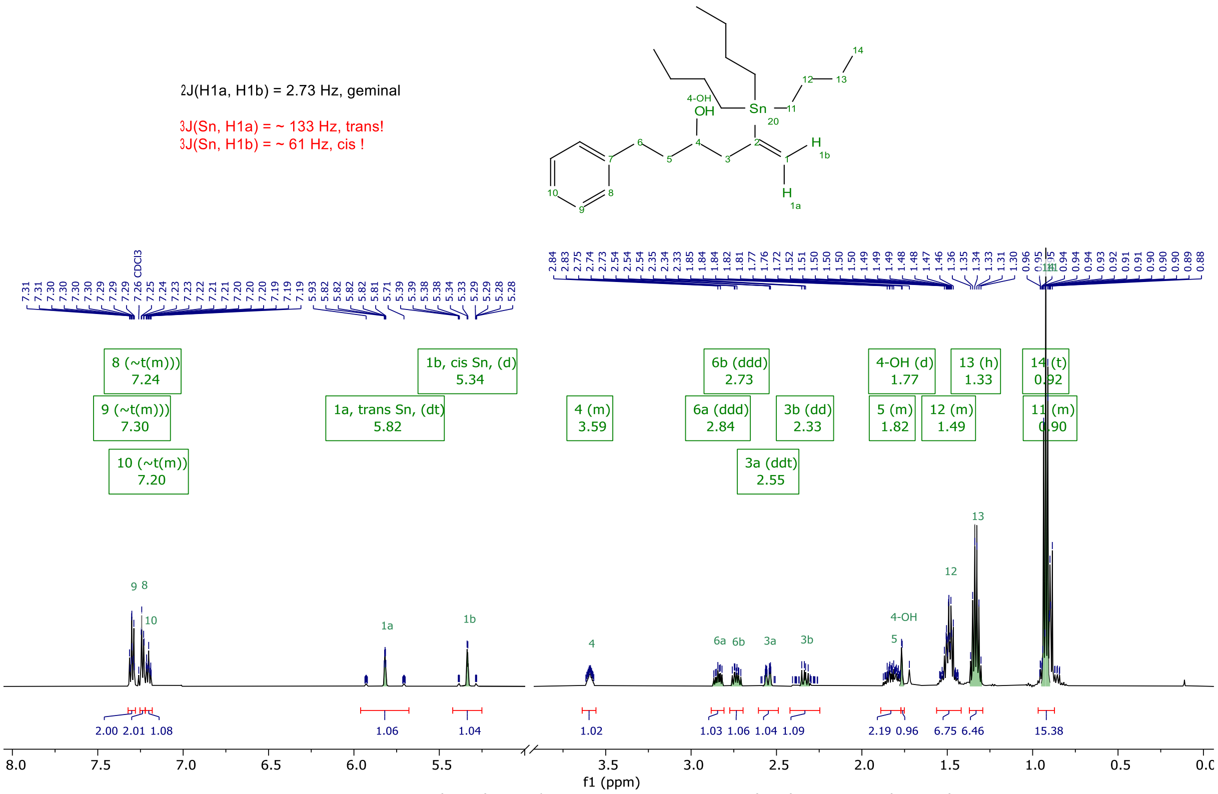

CHW-CA-006-01.10.1.1r — CHW-CA-006-01 (11681) — CDCl3; 298.0 K; 16 mg; 5 mm — 1H (zg30) — AV600neo (cryoBBO) — 27.01.24 01:59:24

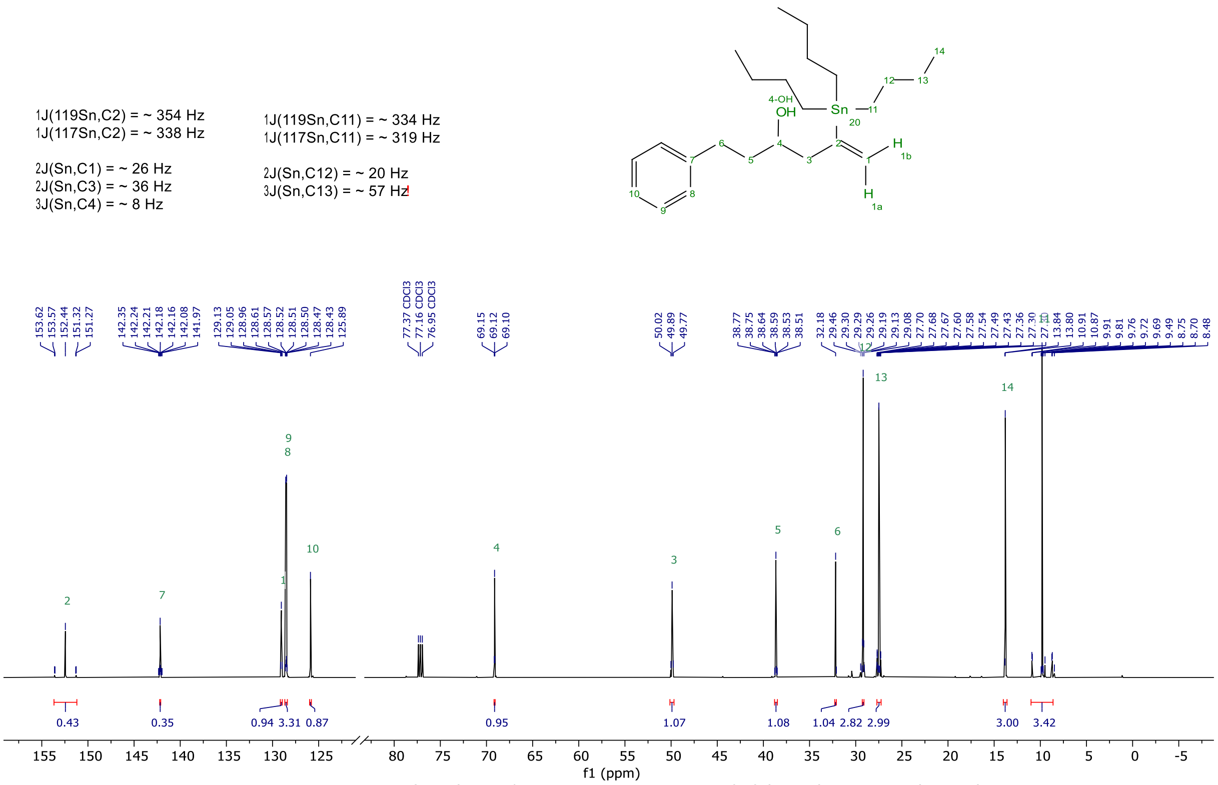

CHW-CA-006-01.11.1.1r — CHW-CA-006-01 (11681) — CDCl3; 298.0 K; 16 mg; 5 mm — 13C{1H} (zgpg30) — AV600neo (cryoBBO) — 27.01.24 02:51:31

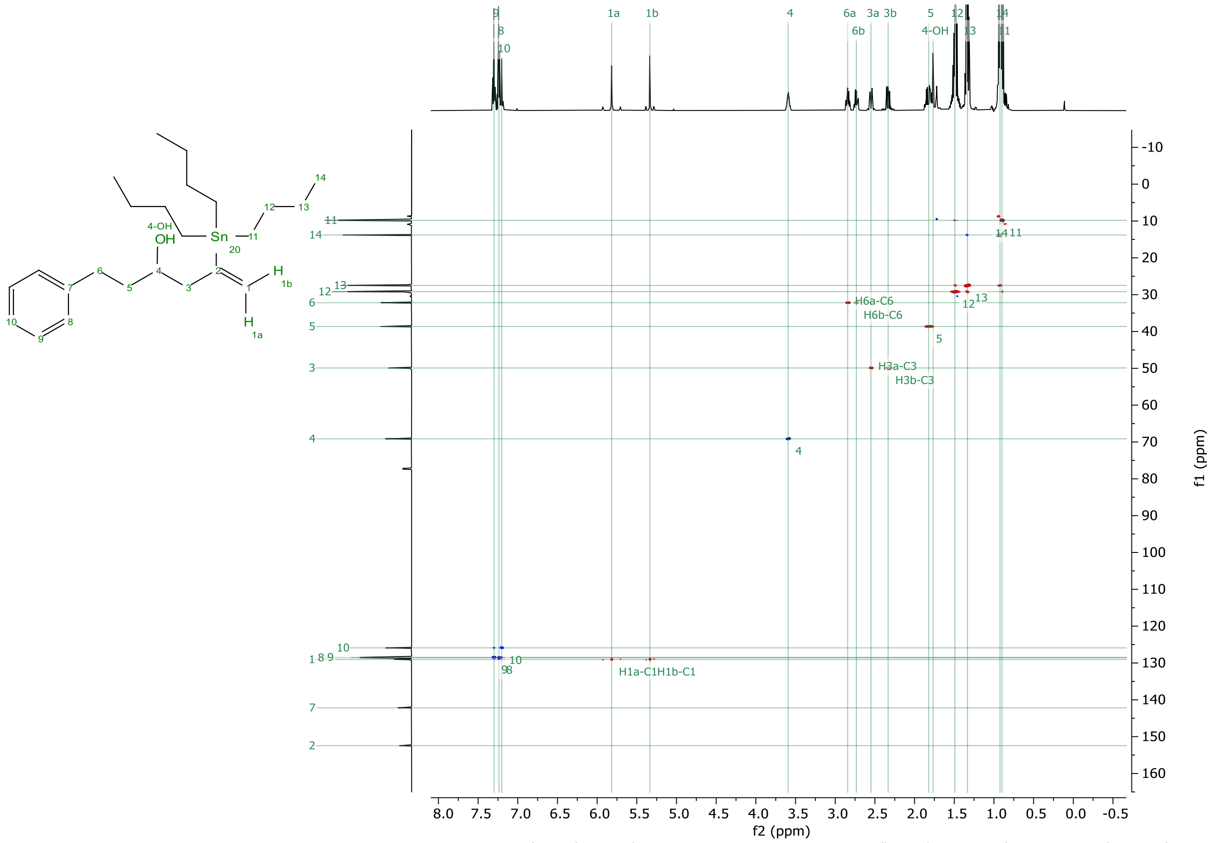

)6-01.12.1.2rr — CHW-CA-006-01 (11681) — CDCl3; 298.0 K; 16 mg; 5 mm — 1H-13C (hsqcedetgpsisp2.3) — AV600neo (cryoBBO) — 27

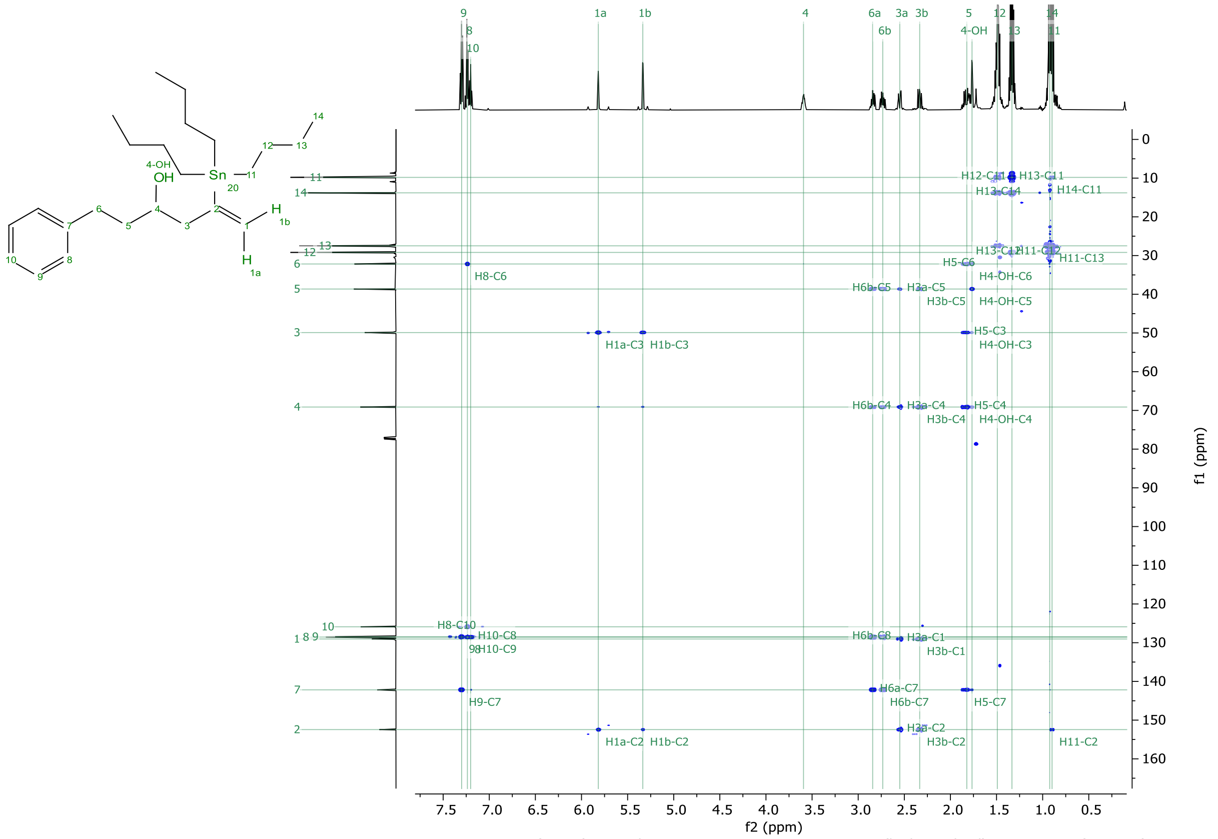

\-006-01.13.1.2rr — CHW-CA-006-01 (11681) — CDCl3; 298.0 K; 16 mg; 5 mm — 1H-13C (hmbcetgpl3nd) — AV600neo (cryoBBO) — 27.01.

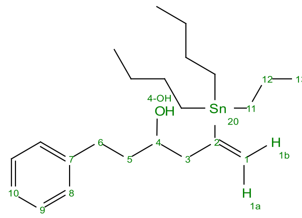

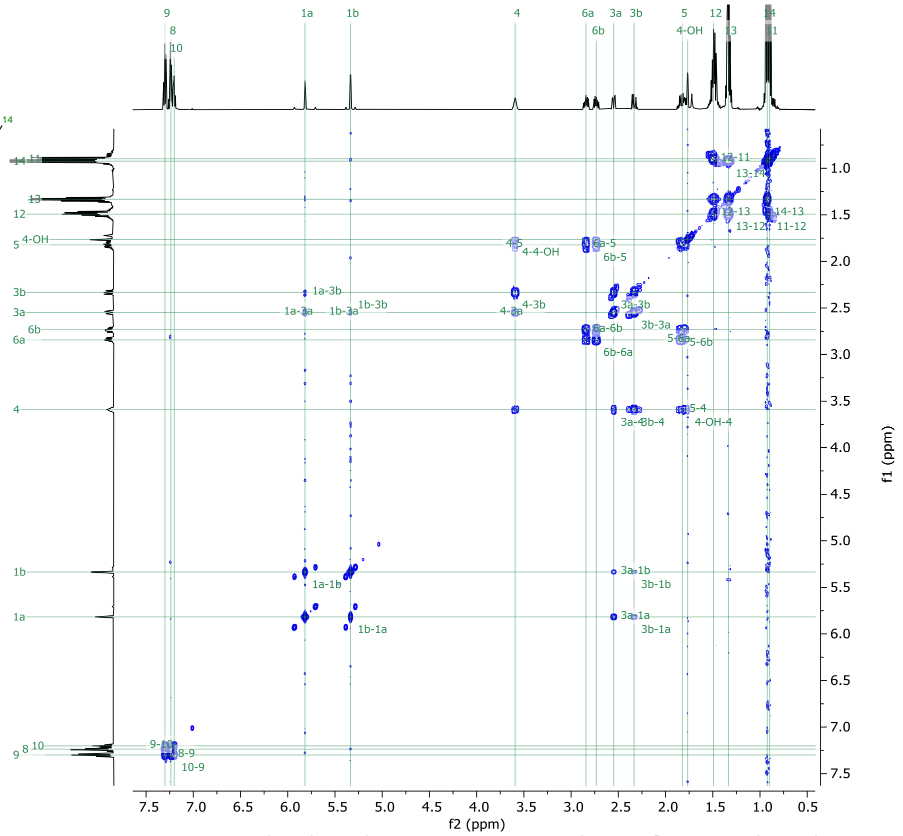

CA-006-01.14.1.2rr — CHW-CA-006-01 (11681) — CDCl3; 298.0 K; 16 mg; 5 mm — 1H (cosygpppqf) — AV600neo (cryoBBO) — 27.01.2[illegible]

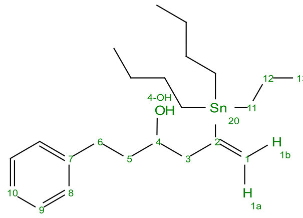

- in NOESY: H1a - H3a, H3b, 4-OH
H1b - H11, H12

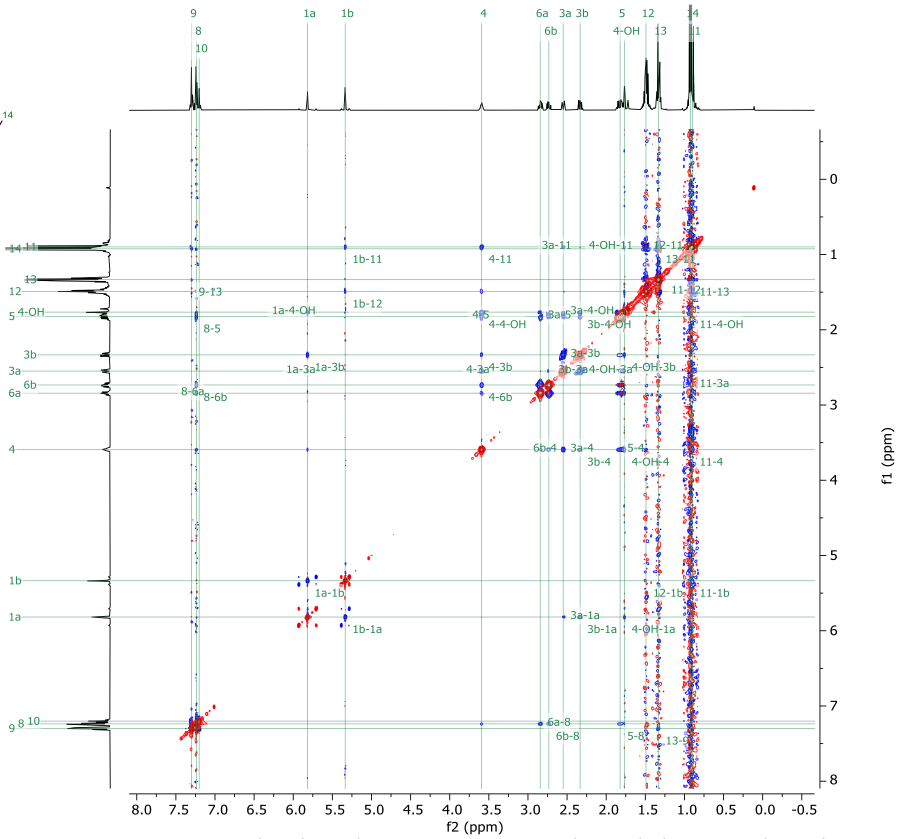

[illegible]A-006-01.15.1.2rr — CHW-CA-006-01 (11681) — CDCl3; 298.0 K; 16 mg; 5 mm — 1H (noesygpphpp) — AV600neo (cryoBBO) — 27.01.2[illegible]

CHW-CA-006-01
5 mm, 16 mg, CDCl3, *298 K, AV600neo, AG NMR/ Petra Philipps

119Sn NMR (224 MHz, $CDCl_3$) δ -44.68.

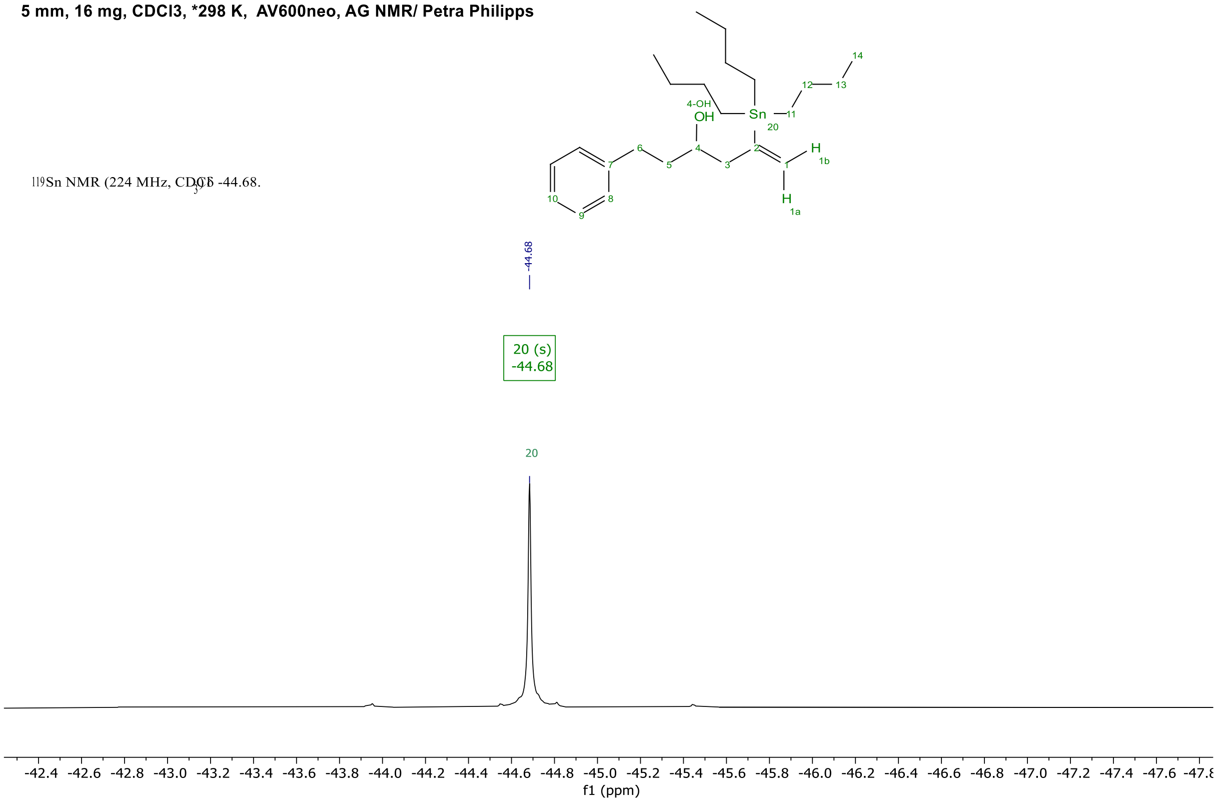

CHW-CA-006-01.16.1.1r — CHW-CA-006-01 (11681) — CDCl3; 298.0 K; 16 mg; 5 mm — 119Sn{1H} (dept45) — AV600neo (cryoBBO) — 27.01.24 04:19:35

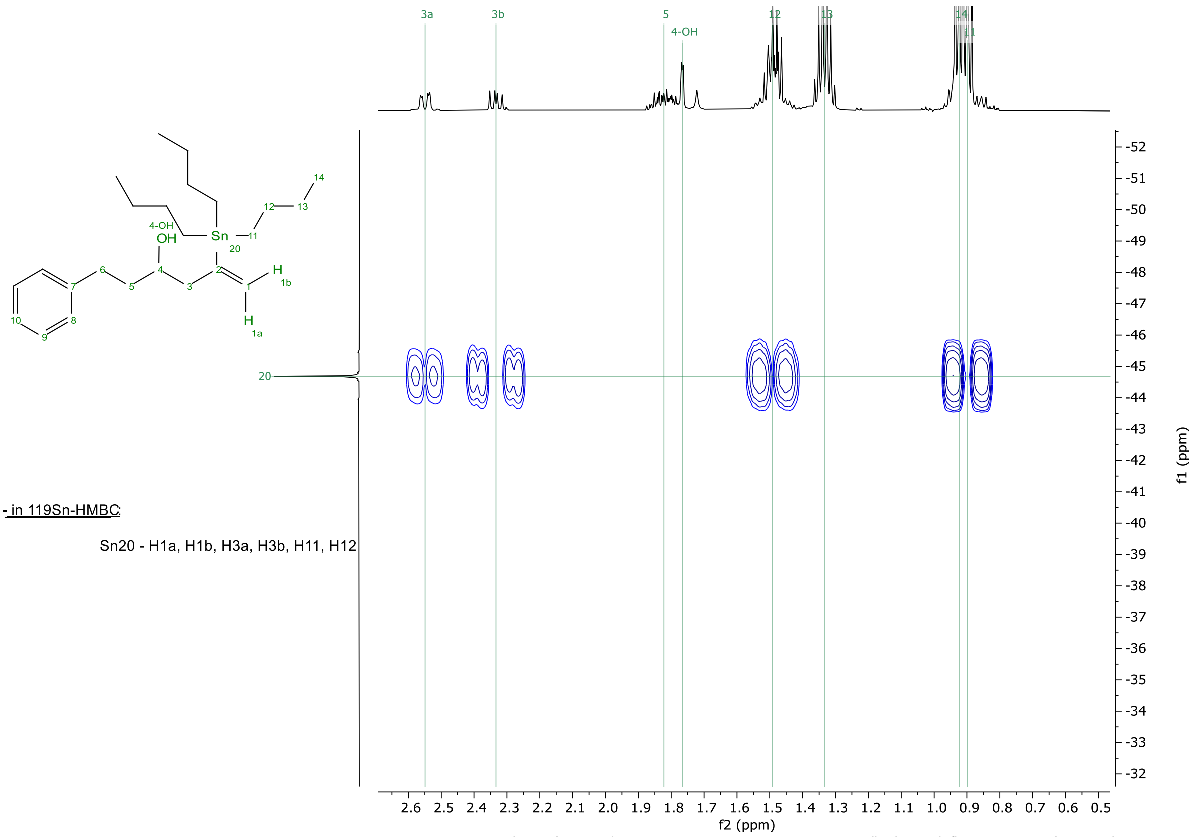

- in 119Sn-HMBC:

Sn20 - H1a, H1b, H3a, H3b, H11, H12

CA-006-01.17.1.2rr — CHW-CA-006-01 (11681) — CDCl3; 298.0 K; 16 mg; 5 mm — 1H-119Sn (hmbcgpndqf) — AV600neo (cryoBBO) — 27.01.2

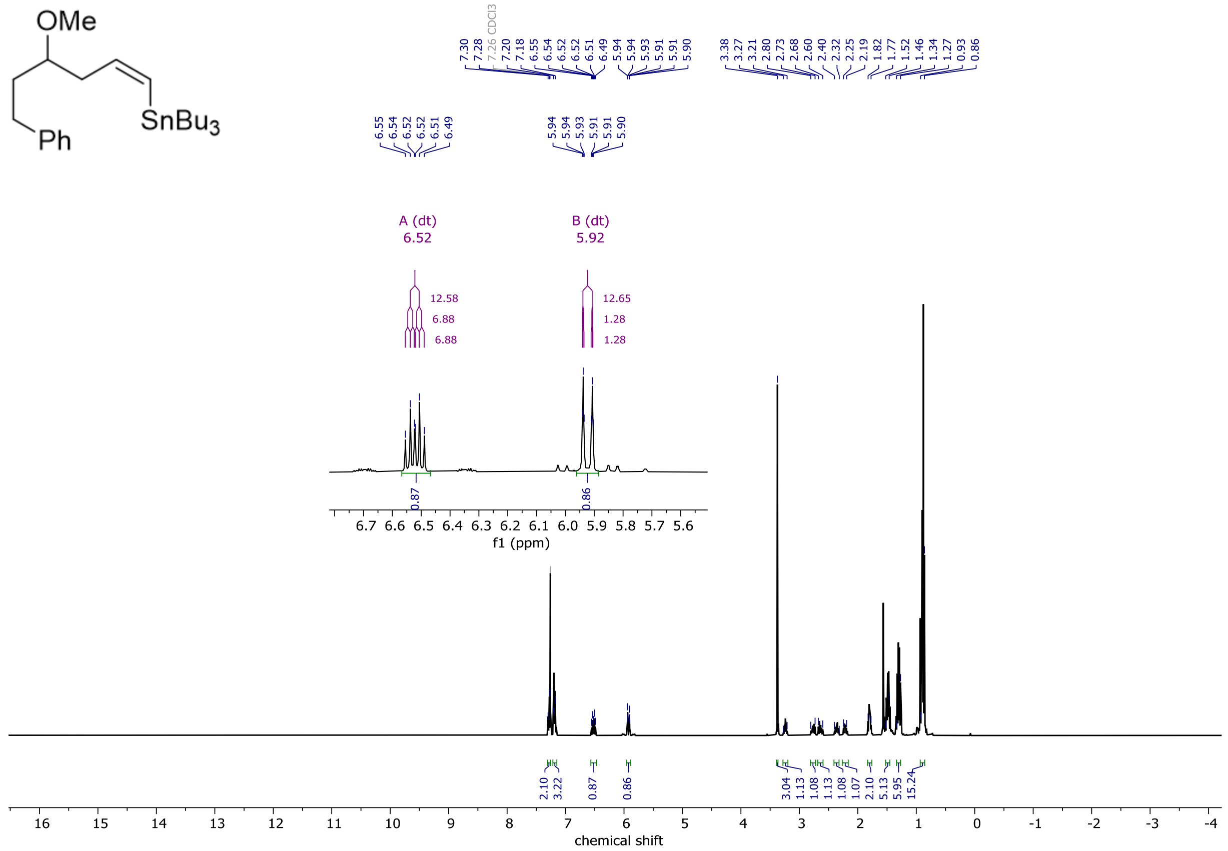

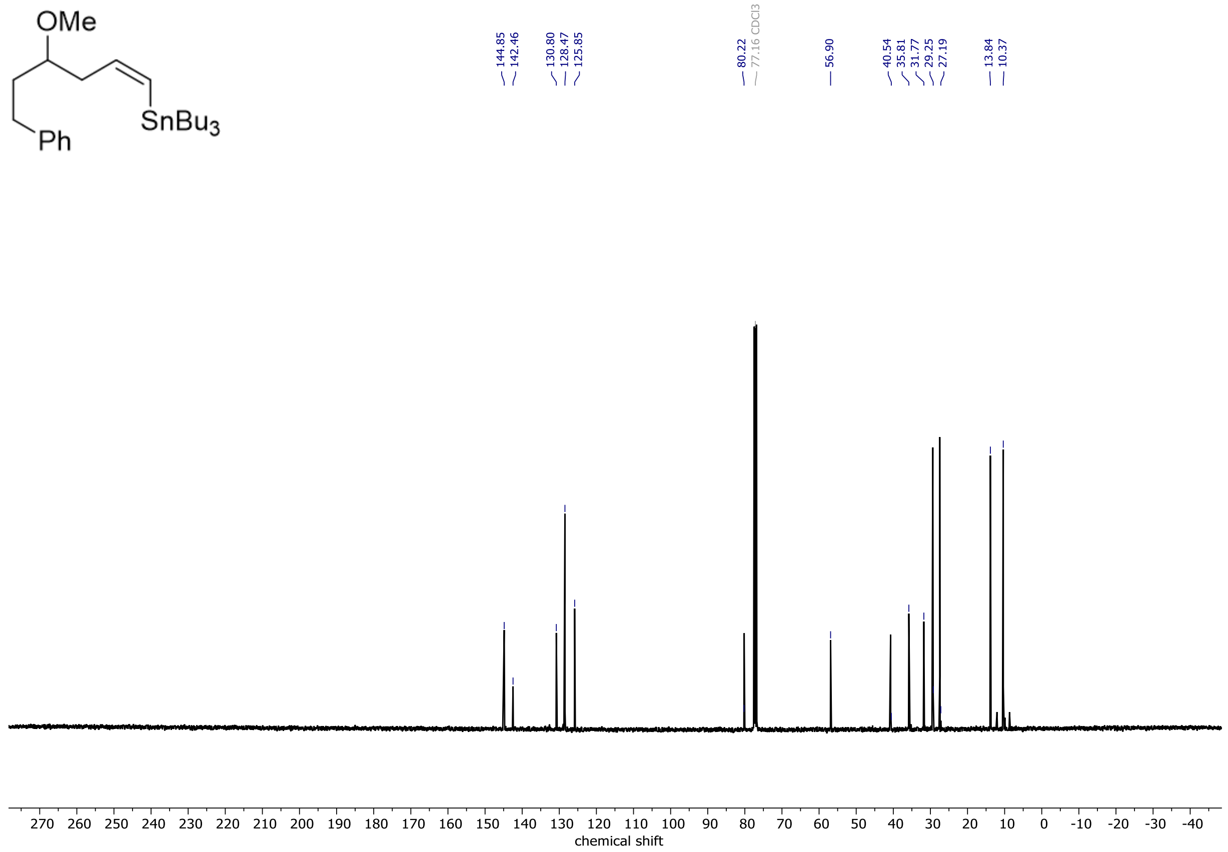

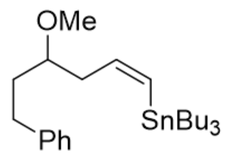
OMe
SnBu3
Ph

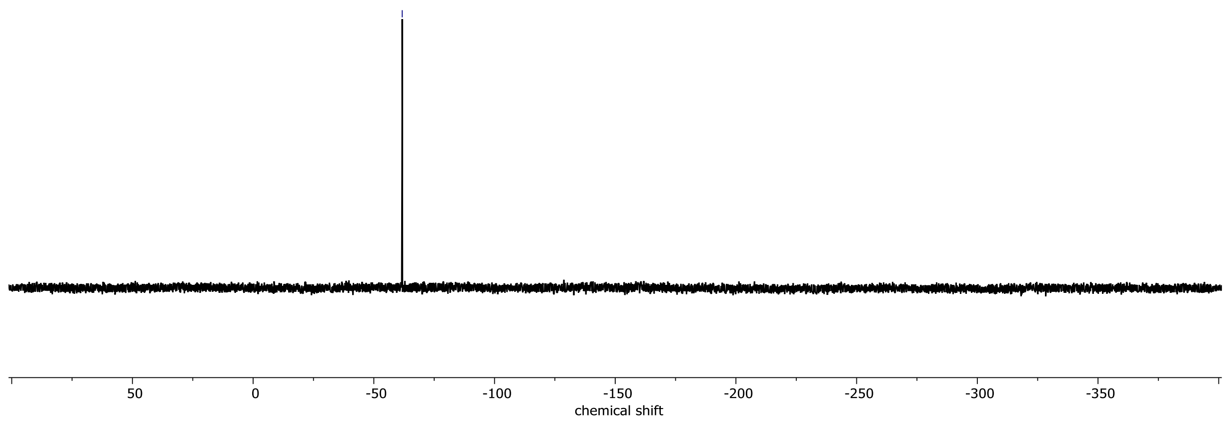
-61.76
50
0
-50
-100
-150
-200
-250
-300
-350
chemical shift

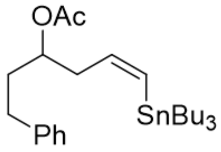
OAc
SnBu3
Ph

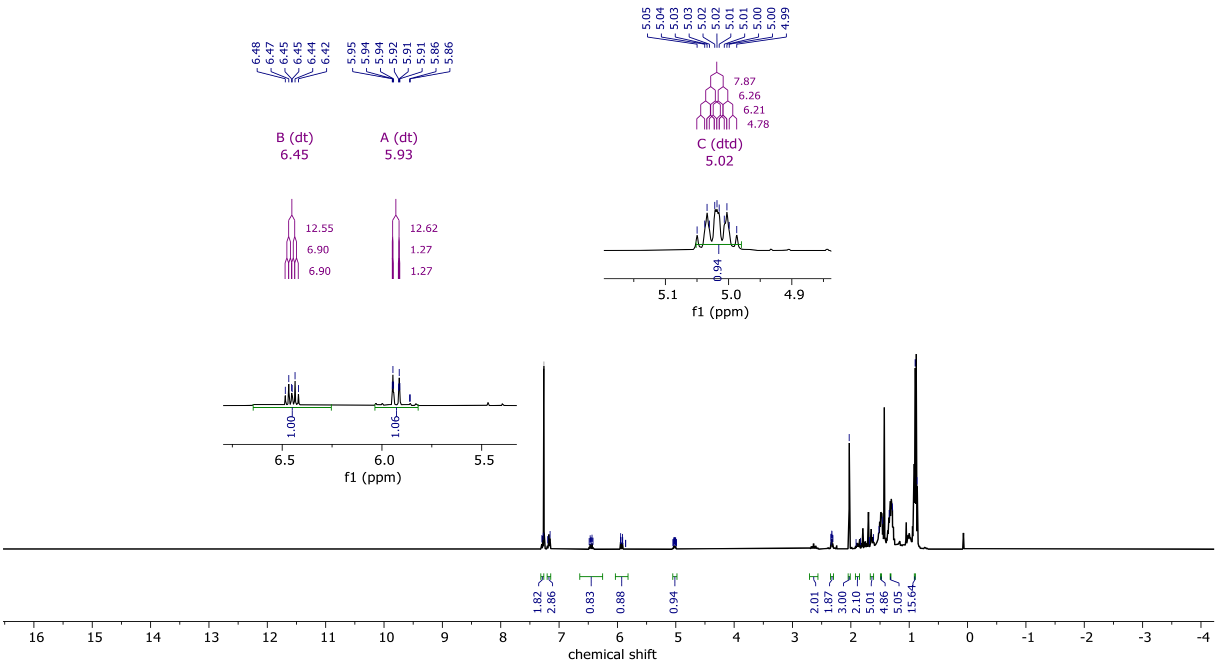
7.30 7.27 7.26 CDCl3 7.20 7.15 6.48 6.47 6.45 6.45 6.44 6.42 5.95 5.94 5.94 5.92 5.91 5.91 5.86 5.86 5.05 5.04 5.03 5.03 5.02 5.02 5.01 5.01 5.00 5.00 4.99 2.34 2.34 2.34 2.33 2.32 2.31 2.31 2.03 1.91 1.83 1.68 1.61 1.52 1.46 1.34 1.29 0.90 0.86
B (dt)
6.45
12.55
6.90
6.90
A (dt)
5.93
12.62
1.27
1.27
C (dtd)
5.02
7.87
6.26
6.21
4.78
0.94
5.1 5.0 4.9
f1 (ppm)
1.00
1.06
6.5 6.0 5.5
f1 (ppm)
1.82 2.86 0.83 0.88 0.94 2.01 1.87 3.00 2.10 5.01 4.86 5.05 15.64
16 15 14 13 12 11 10 9 8 7 6 5 4 3 2 1 0 -1 -2 -3 -4
chemical shift

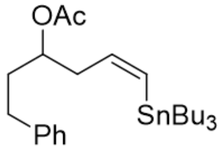
OAc
SnBu3
Ph

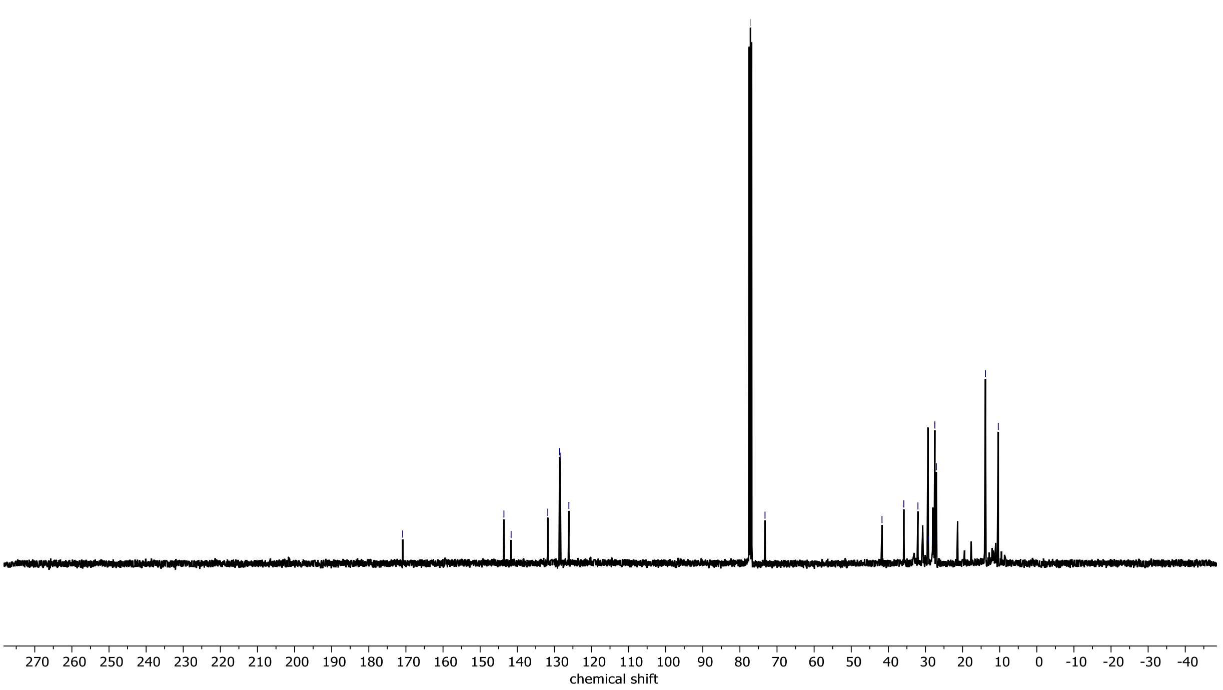
170.87
143.59 141.65 131.76 128.57 128.44 126.09
77.16 CDCl3 73.23
41.71 35.82 32.03 29.22 27.47 27.08
13.85 10.38
270 260 250 240 230 220 210 200 190 180 170 160 150 140 130 120 110 100 90 80 70 60 50 40 30 20 10 0 -10 -20 -30 -40
chemical shift

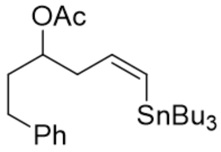

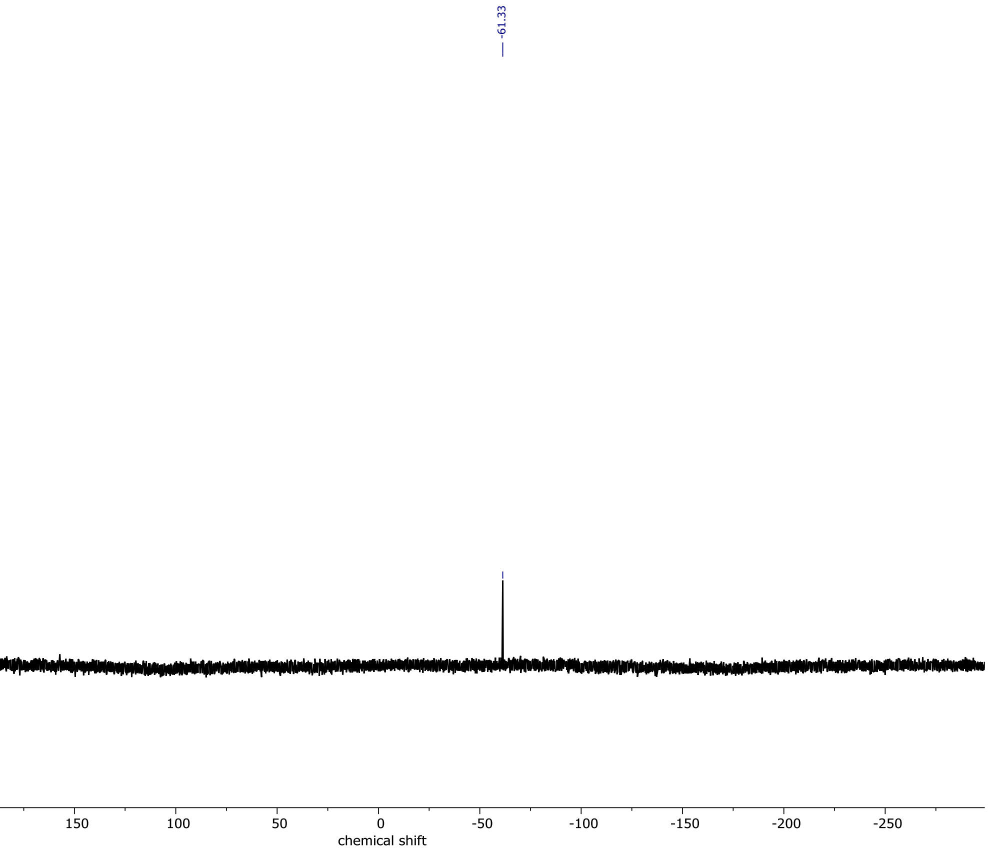
-61.33
250
200
150
100
50
0
-50
-100
-150
-200
-250
chemical shift

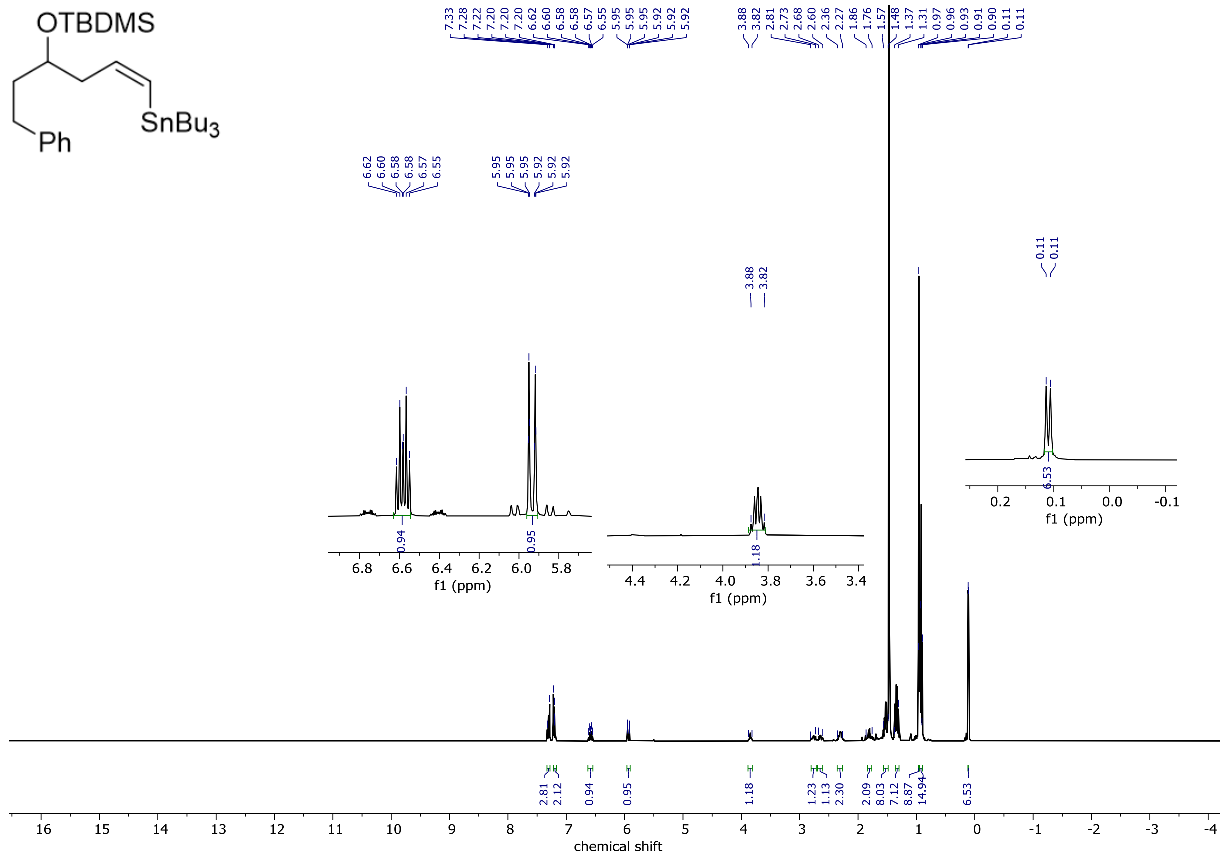
OTBDMS
SnBu3
Ph
7.33 7.28 7.22 7.20 7.20 7.20 6.62 6.60 6.58 6.58 6.57 6.55 5.95 5.95 5.95 5.92 5.92 5.92
3.88 3.82 2.81 2.73 2.68 2.60 2.36 2.27 1.86 1.76 1.57 1.48 1.37 1.31 0.97 0.96 0.93 0.91 0.90 0.11 0.11
2.81 2.12 0.94 0.95 1.18 1.23 1.13 2.30 2.09 8.03 7.12 8.87 14.94 6.53
f1 (ppm)
chemical shift

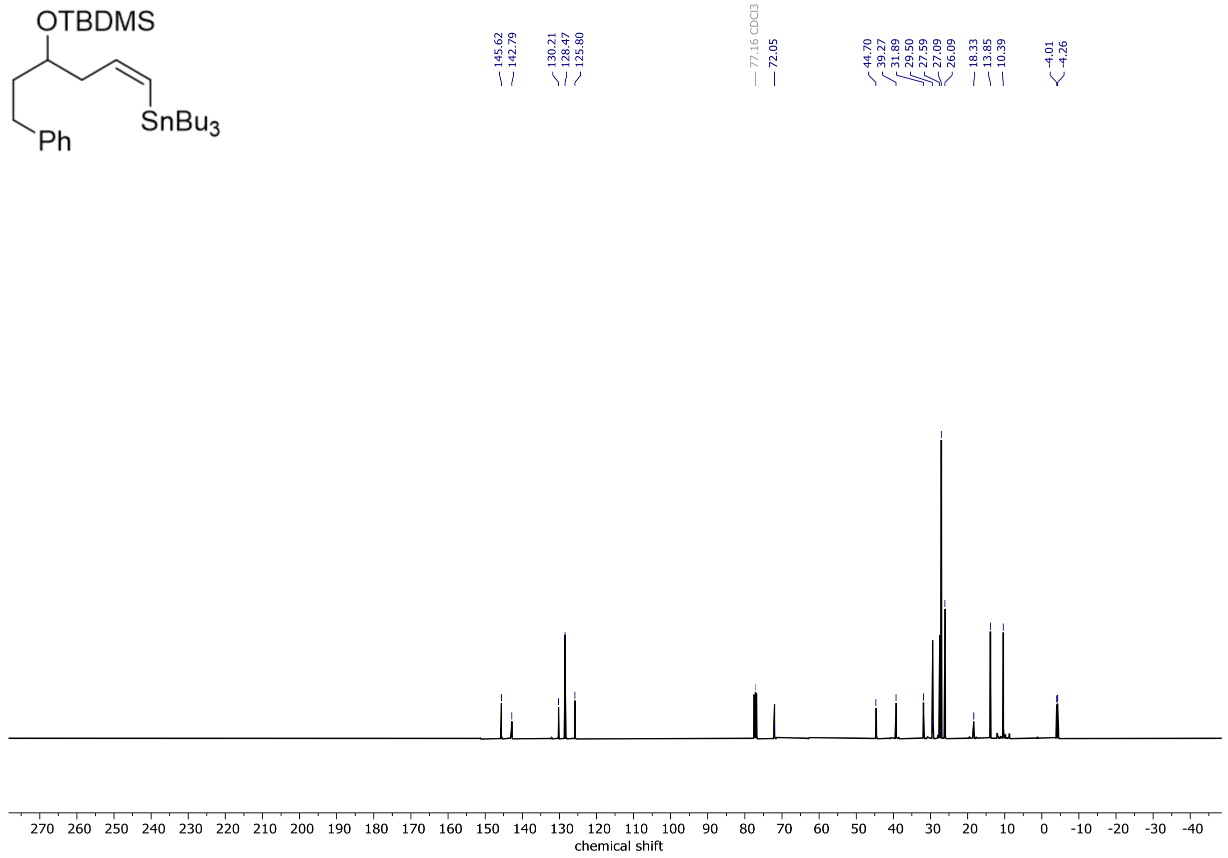
OTBDMS
SnBu3
Ph
145.62 142.79 130.21 128.47 125.80 77.16 CDCl3 72.05 44.70 39.27 31.89 29.50 27.59 27.09 26.09 18.33 13.85 10.39 -4.01 -4.26
chemical shift

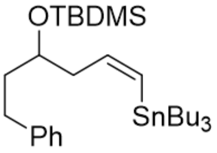
OTBDMS
SnBu3
Ph

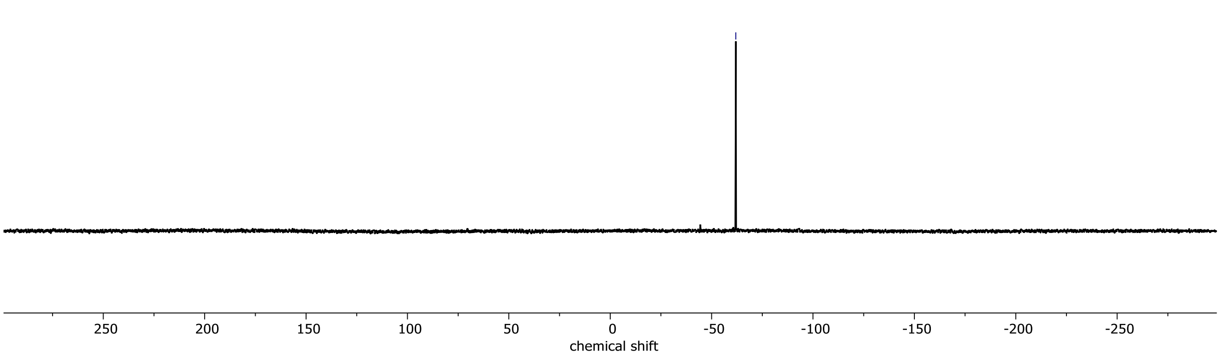
-61.94
250
200
150
100
50
0
-50
-100
-150
-200
-250
chemical shift

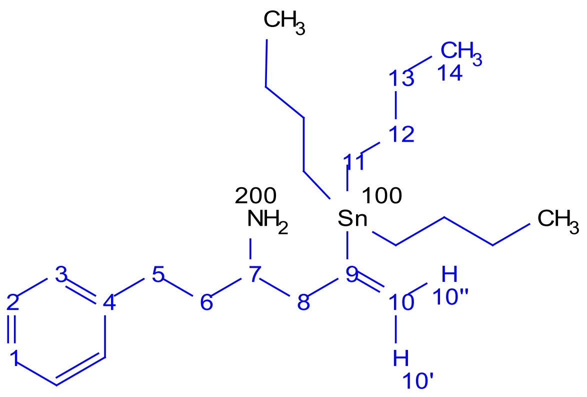

Atom	δ (ppm)	Predicted Shift	J	COSY	HSQC	HMBC	NOESY
1 C	125.916	126.26			1	3	
H	7.174	7.23			1		
2 C	128.520	128.49			2	2	
H	7.275	7.26	7.00(3)		2	2, 4	
3 C	128.488	128.39			3	3, 5', 5''	
H	7.196	7.20	7.00(2)		3	1, 3, 5	5', 5'', 6'
4 C	142.355	141.99				2, 5', 5'', 6', 6''	
5 C	32.841	32.48			5', 5''	3, 6', 6''	
H'	2.649	2.61, 2.72		5''	5	3, 4, 6, 7	3, 6''
H''	2.770	2.61, 2.72		5', 6', 6''	5	3, 4, 6, 7	3, 6', 6''
6 C	39.744	36.38			6', 6''	5', 5'', 8', 8''	
H'	1.634	1.57, 1.81		5''	6	4, 5, 7, 8	3, 5''
H''	1.738	1.57, 1.81		5''	6	4, 5, 7, 8	5', 5'', 8', 8''
7 C	49.794	50.76			7	5', 5'', 6', 6'', 8', 8''	
H	2.737	2.77		8'	7		11''
8 C	50.829	46.00			8', 8''	6', 6'', 10', 10''	
H'	2.122	1.87, 2.12		7, 8''	8	6, 7, 9	6'', 8'', 10'
H''	2.492	1.87, 2.12		8'	8	6, 7, 9	6'', 8', 11''

Atom	δ (ppm)	Predicted Shift	J	COSY	HSQC	HMBC	NOESY
9 C	153.308	161.64				8', 8'', 10', 10'', 11''	
10 C	127.954	126.19					
10' H	5.736	5.32		10''		8, 9	8'
10'' H	5.237	5.36		10'		8, 9	11'', 12
11 C	9.849	10.88			11', 11''	11'', 12, 13', 13''	
H'		0.91, 0.92			11		
H''	0.856	0.91, 0.92		12	11	9, 11	7, 8'', 10''
12 C	29.257	28.81			12	13', 13'', 14	
H2	1.465	1.46		11'', 13', 13''	12	11, 13, 14	10''
13 C	27.534	27.39			13', 13''	12, 14	
H'	1.318	1.32		12, 14	13	11, 12, 14	
H''		1.32		12	13	11, 12, 14	
14 C	13.828	13.55			14	12, 13', 13''	
H3	0.903	0.89		13'	14	12, 13	
100 Sn							
200 N							
H2		2.52					

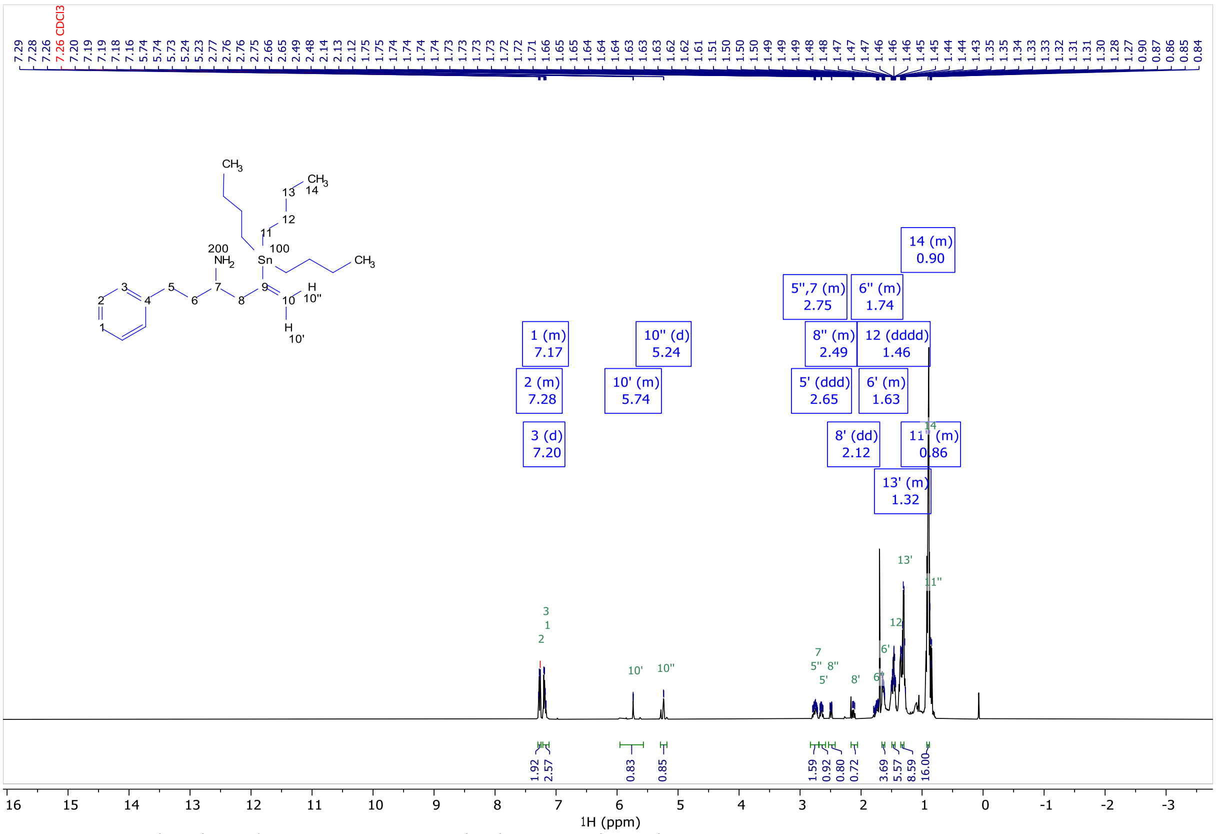

1D — CHW-CA-055-01 (12409) — CDCl3; 298.0 K; 8 mg; 5 mm — 1H (zg30) — AV600neo (cryoBBO) — 14.05.24 18:18:07 — zg30

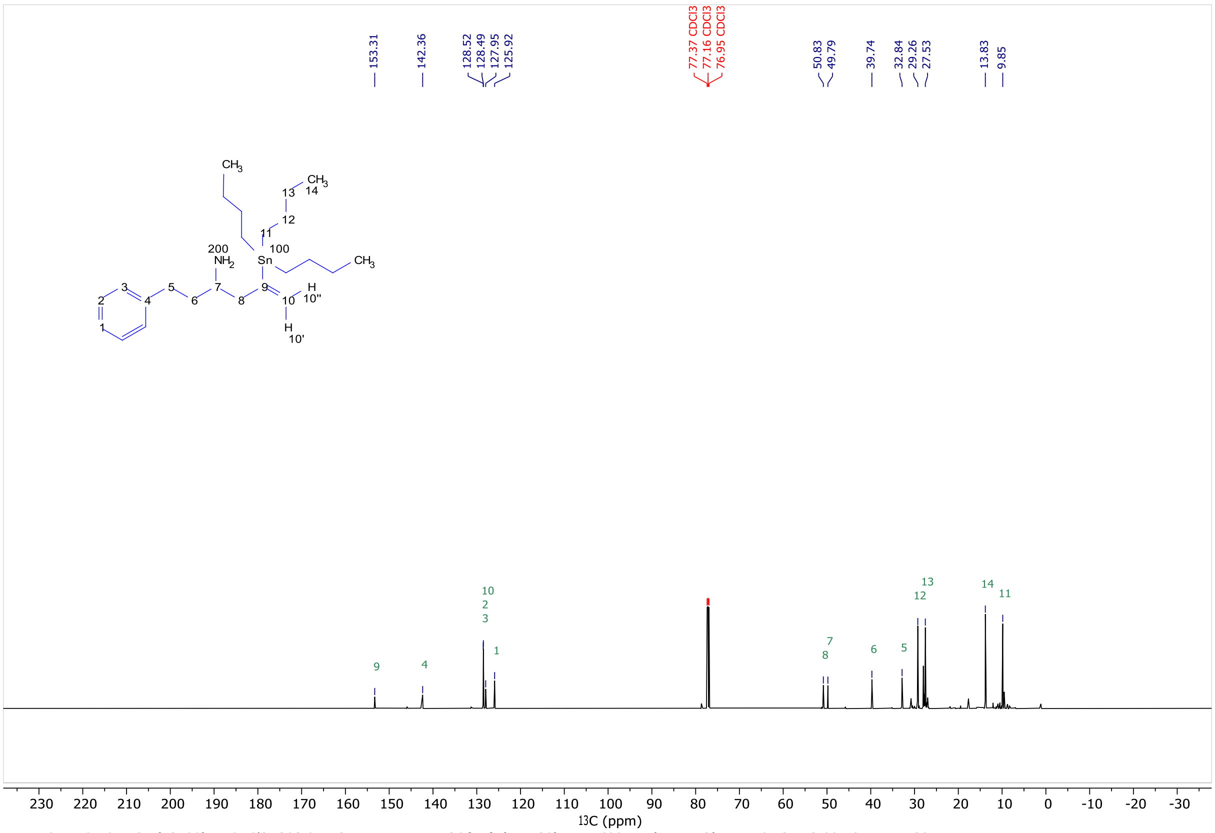

1D — CHW-CA-055-01 (12409) — CDCl3; 298.0 K; 8 mg; 5 mm — 13C{1H} (zgpg30) — AV600neo (cryoBBO) — 14.05.24 18:09:19 — zgpg30

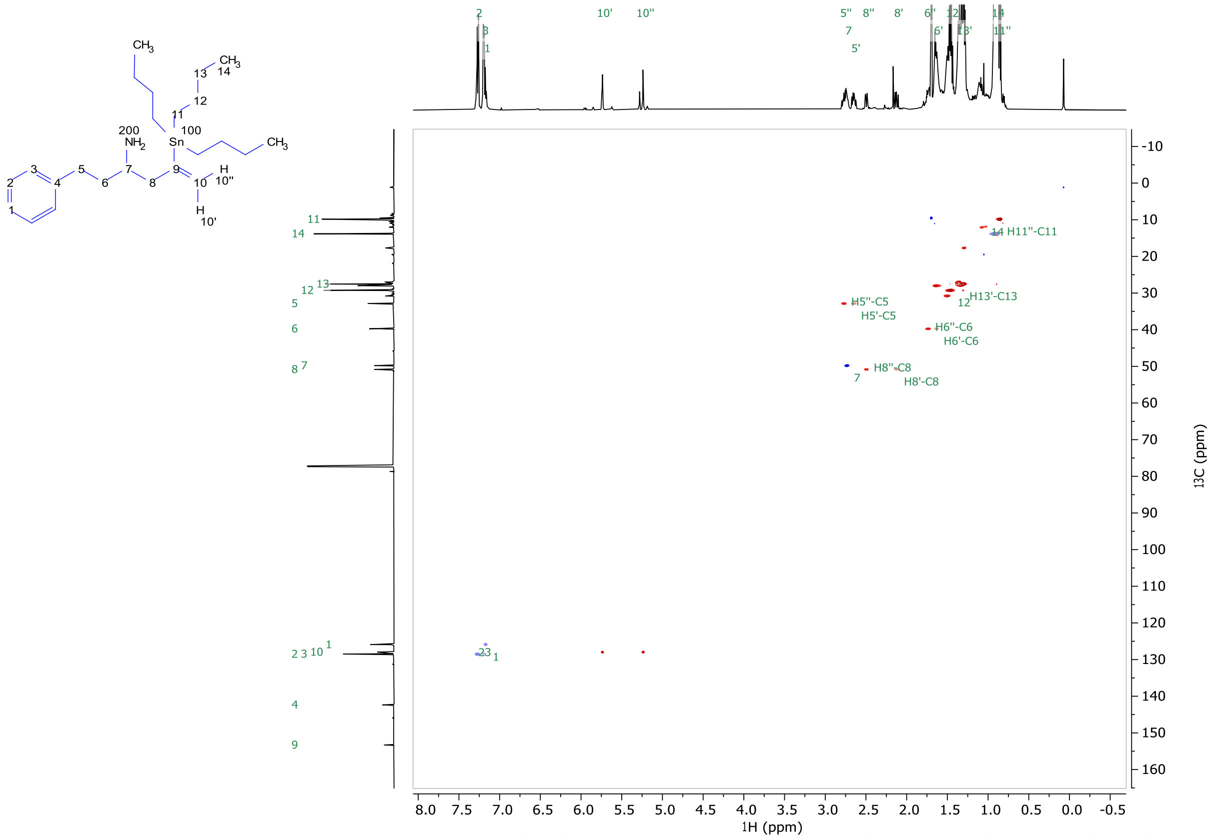

HSQC-EDITED — CHW-CA-055-01 (12409) — CDCl3; 298.0 K; 8 mg; 5 mm — 1H-13C (hsqcedetgpsisp2.3) — AV600neo (cryoBBO) — 14.0!

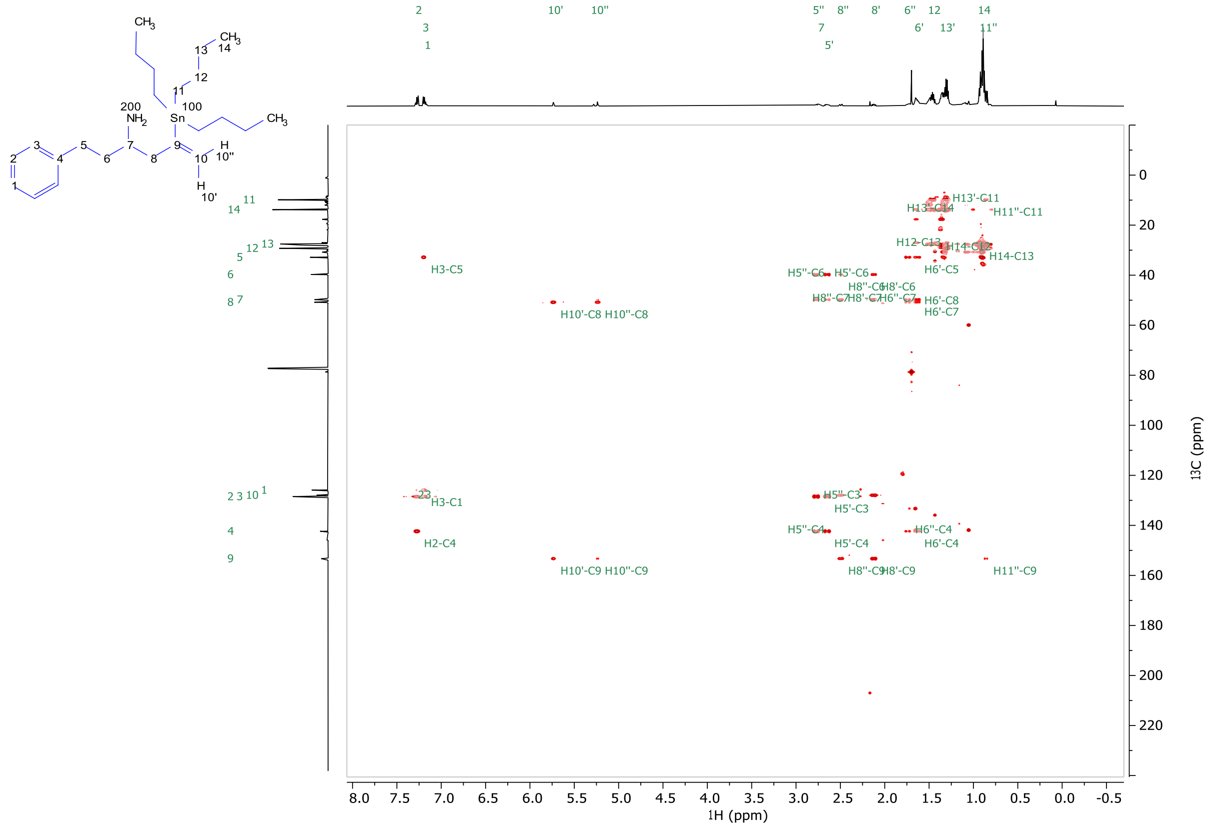

HMBC — CHW-CA-055-01 (12409) — CDCl3; 298.0 K; 8 mg; 5 mm — 1H-13C (hmbcetgpl3nd) — AV600neo (cryoBBO) — 14.05.24 22:05:16 — hmbce

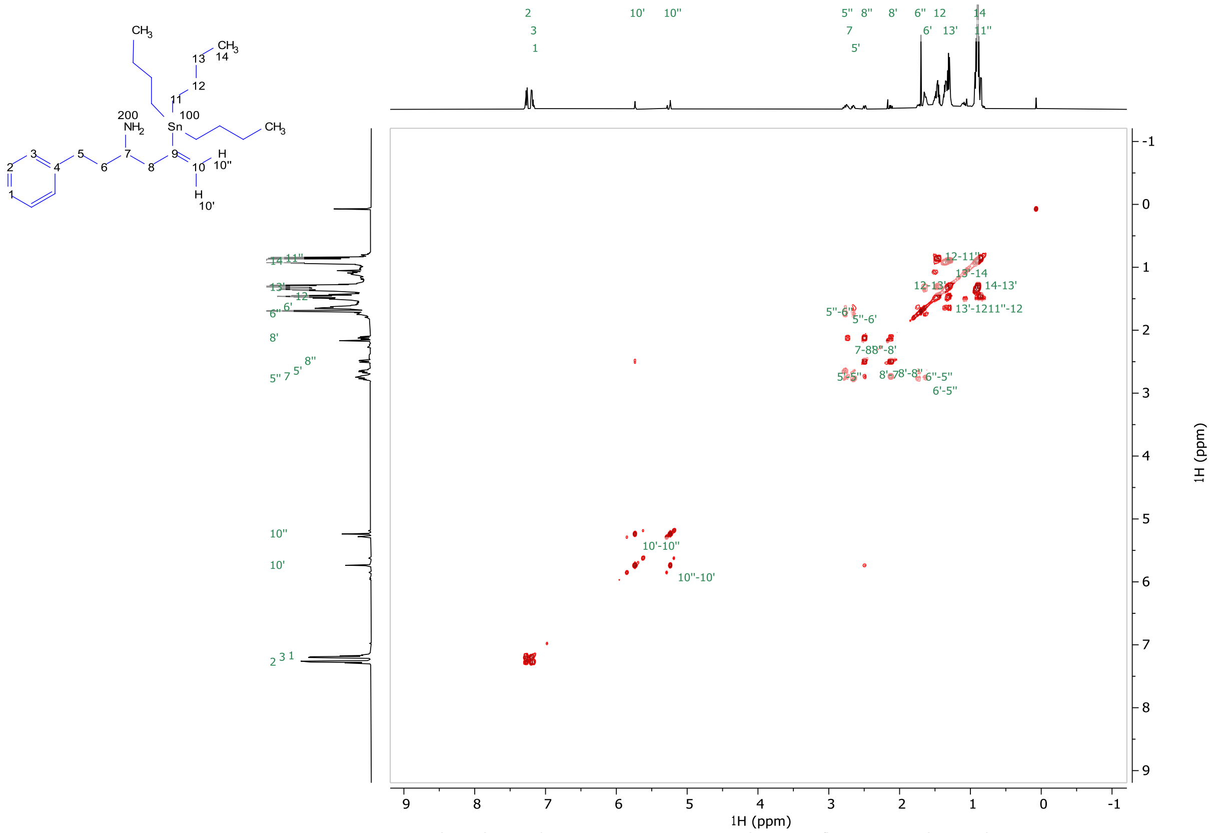

COSY — CHW-CA-055-01 (12409) — CDCl3; 298.0 K; 8 mg; 5 mm — 1H (cosygpppqf) — AV600neo (cryoBBO) — 14.05.24 22:19:25 — cosygpp

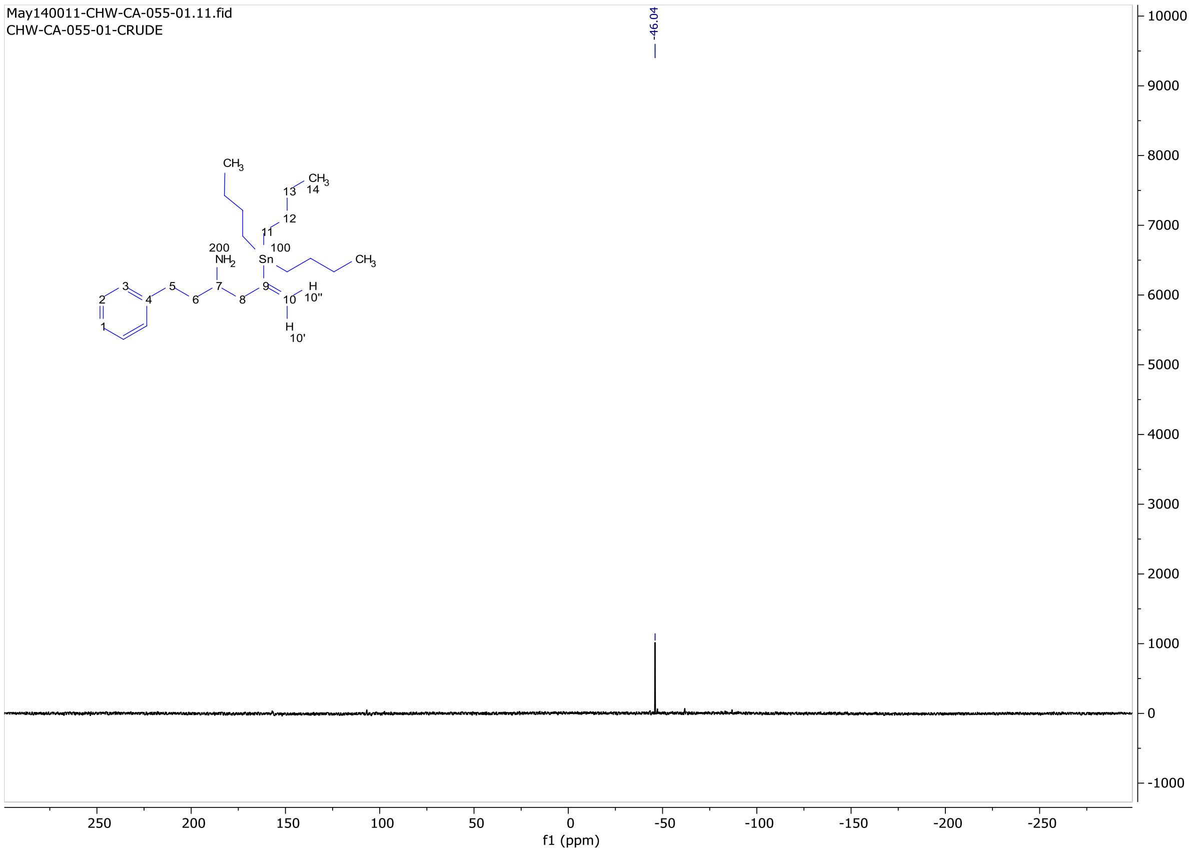

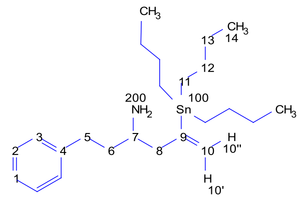

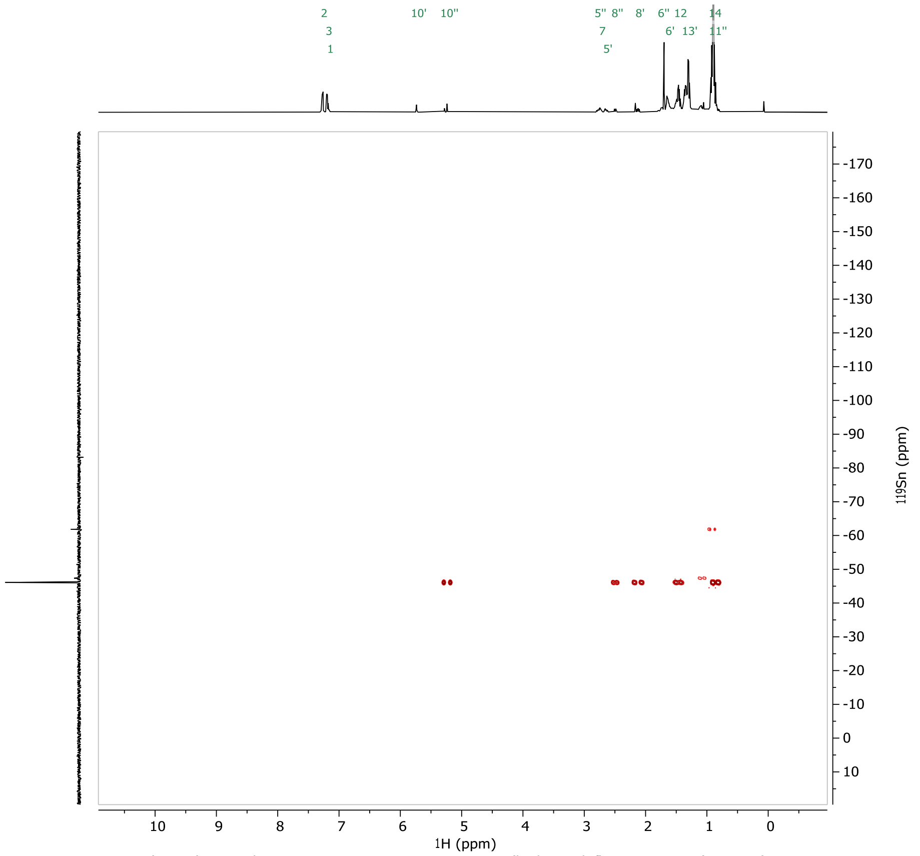

HMBC — CHW-CA-055-01 (12409) — CDCl3; 298.0 K; 8 mg; 5 mm — 1H-119Sn (hmbcgpndqf) — AV600neo (cryoBBO) — 14.05.24 22:10:42

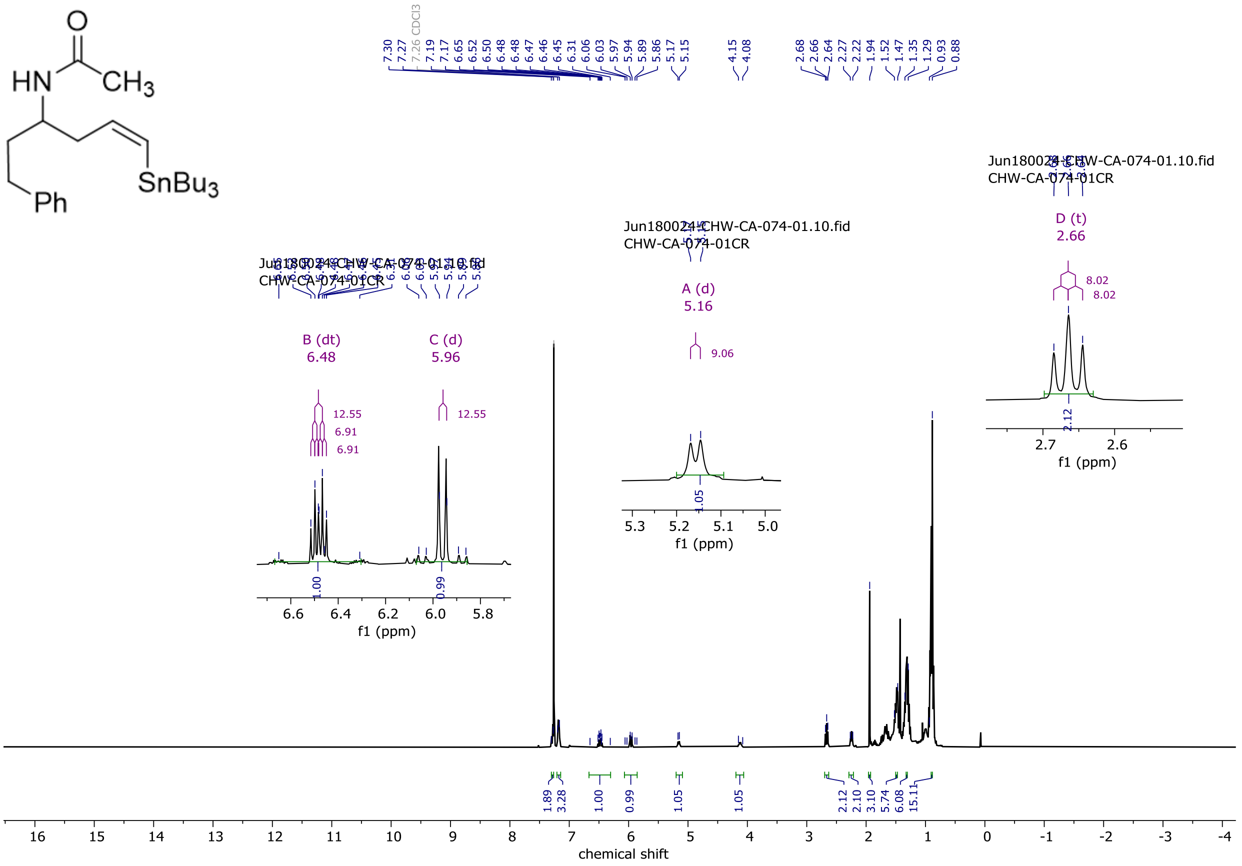

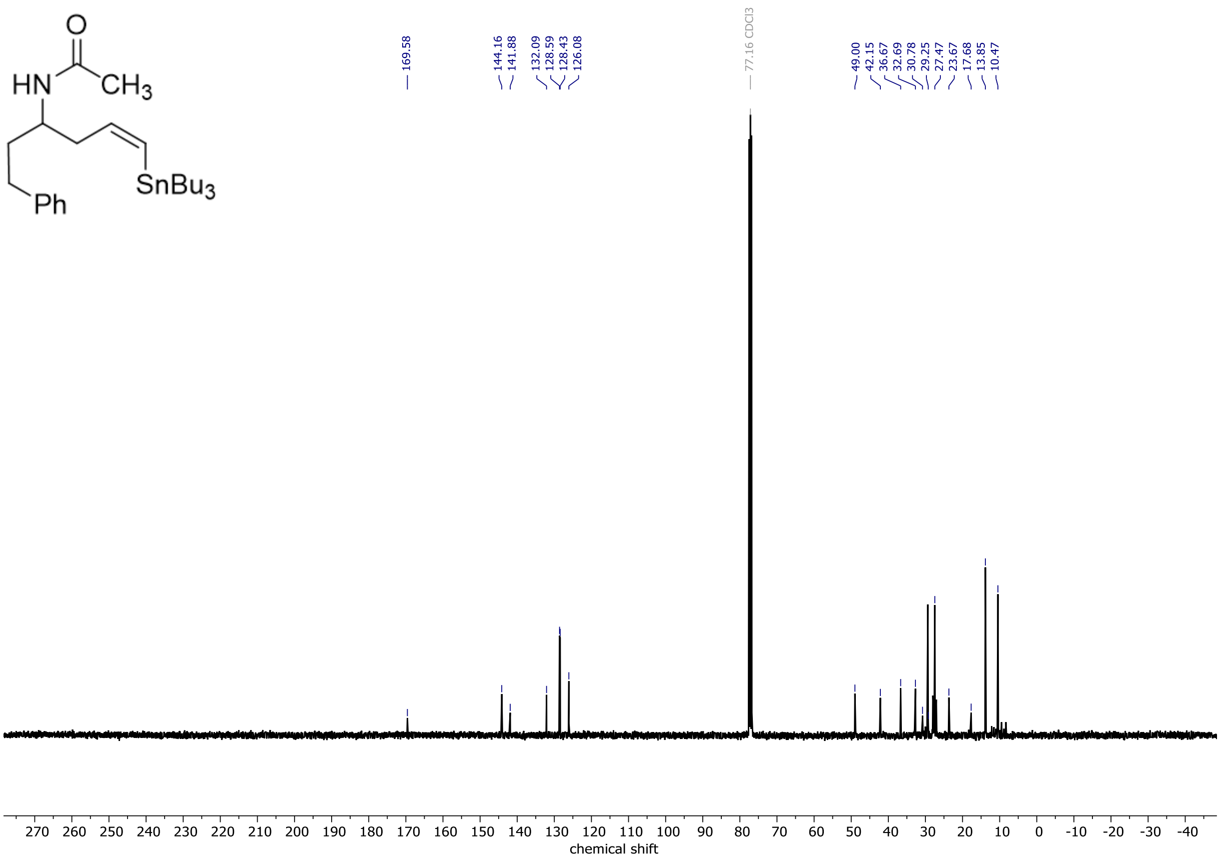

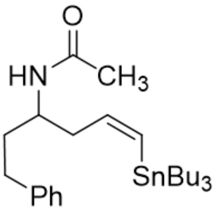

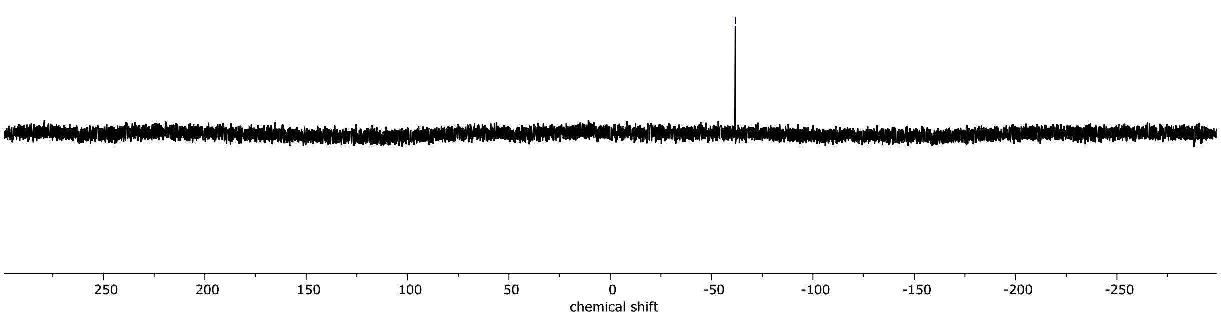
-61.77
250
200
150
100
50
0
-50
-100
-150
-200
-250
chemical shift

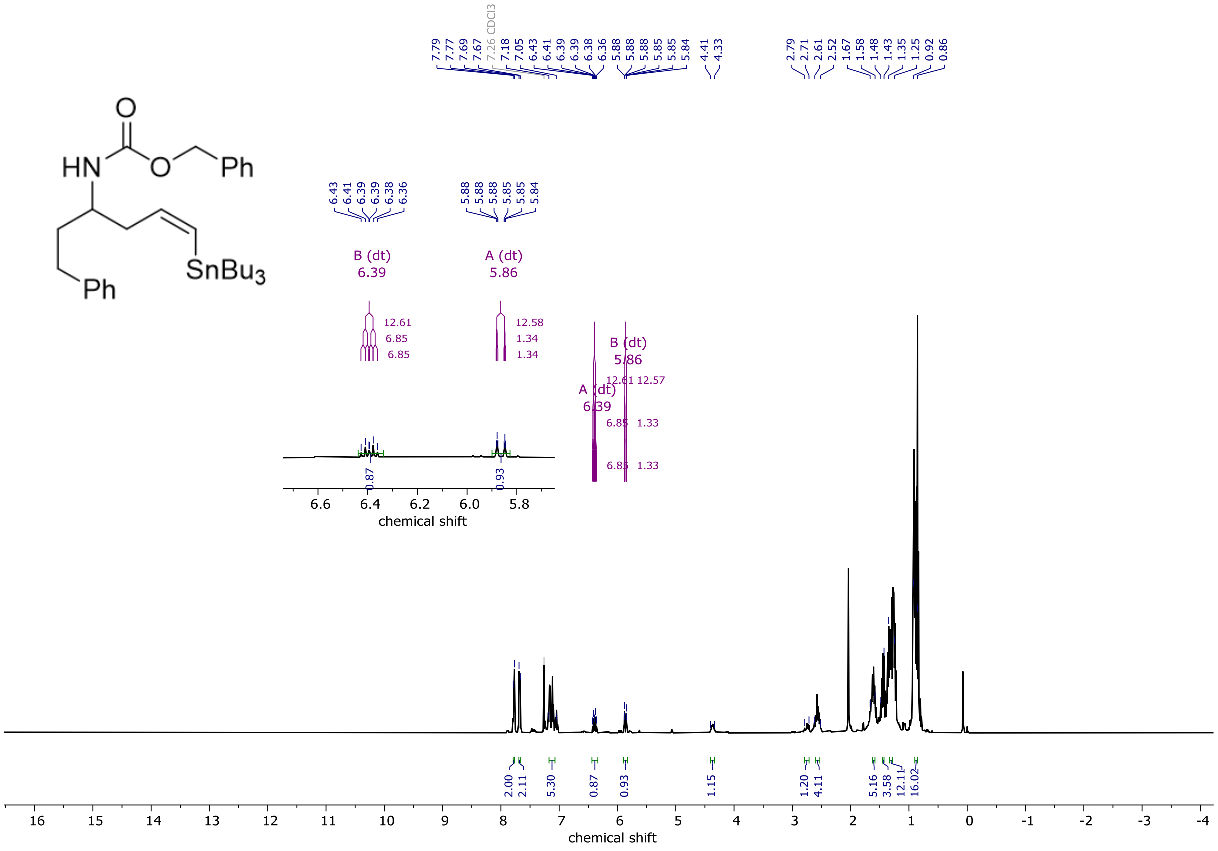
7.79 7.77 7.69 7.67 7.26 CDCl3 7.18 7.05 6.43 6.41 6.39 6.39 6.38 6.36 5.88 5.88 5.88 5.85 5.85 5.84 4.41 4.33 2.79 2.71 2.61 2.52 1.67 1.58 1.48 1.43 1.35 1.25 0.92 0.86
HN
O
O
Ph
SnBu3
Ph
B (dt)
6.39
12.61
6.85
6.85
A (dt)
5.86
12.58
1.34
1.34
0.87
0.93
chemical shift
2.00 2.11 5.30 0.87 0.93 1.15 1.20 4.11 5.16 3.58 12.11 16.02
chemical shift

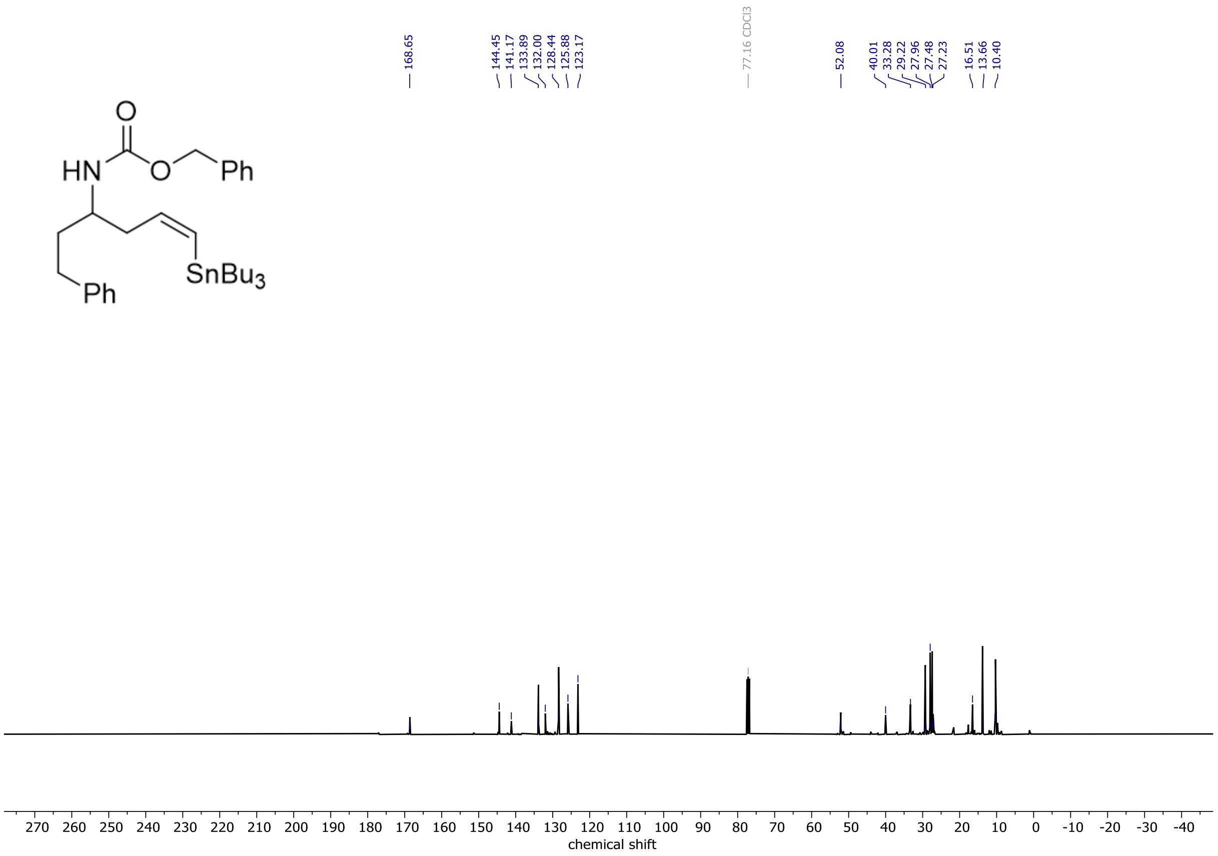
168.65 144.45 141.17 133.89 132.00 128.44 125.88 123.17 77.16 CDCl3 52.08 40.01 33.28 29.22 27.96 27.48 27.23 16.51 13.66 10.40
HN
O
O
Ph
SnBu3
Ph
chemical shift

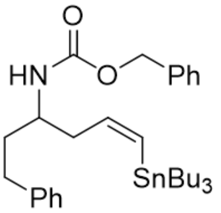
HN
O
O
Ph
SnBu3
Ph

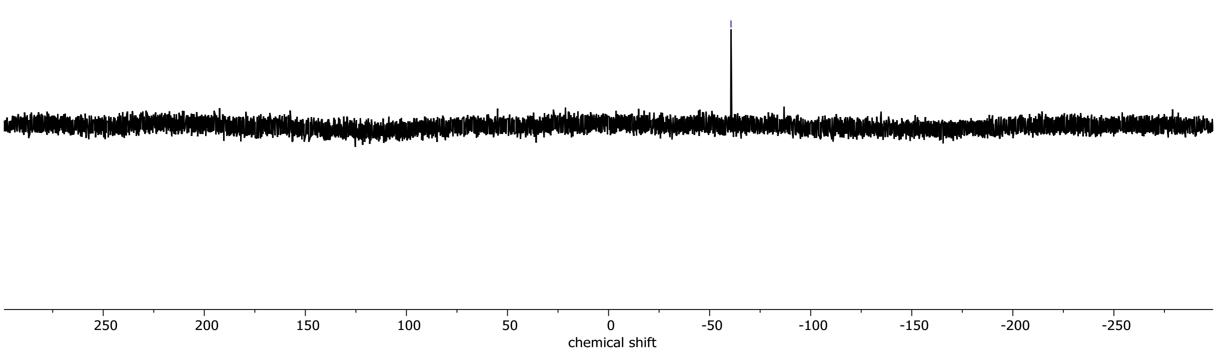
-60.59
250
200
150
100
50
0
-50
-100
-150
-200
-250
chemical shift

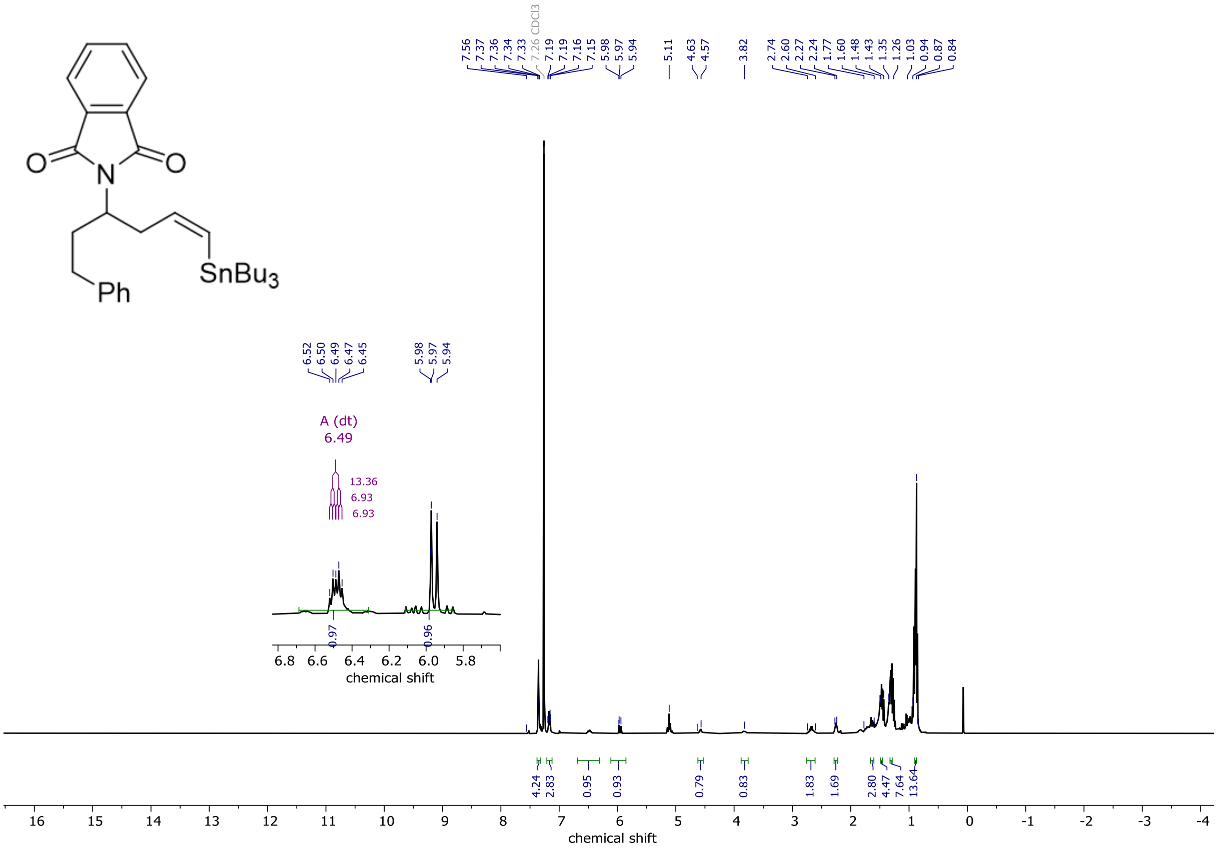
SnBu3
Ph
7.56 7.37 7.36 7.34 7.33 7.26 CDCl3 7.19 7.19 7.16 7.15 5.98 5.97 5.94
5.11 4.63 4.57 3.82
2.74 2.60 2.27 2.24 1.77 1.60 1.48 1.43 1.35 1.26 1.03 0.94 0.87 0.84
6.52 6.50 6.49 6.47 6.45 5.98 5.97 5.94
A (dt)
6.49
13.36
6.93
6.93
0.97
0.96
chemical shift
4.24 2.83 0.95 0.93 0.79 0.83 1.83 1.69 2.80 4.47 7.64 13.64
chemical shift

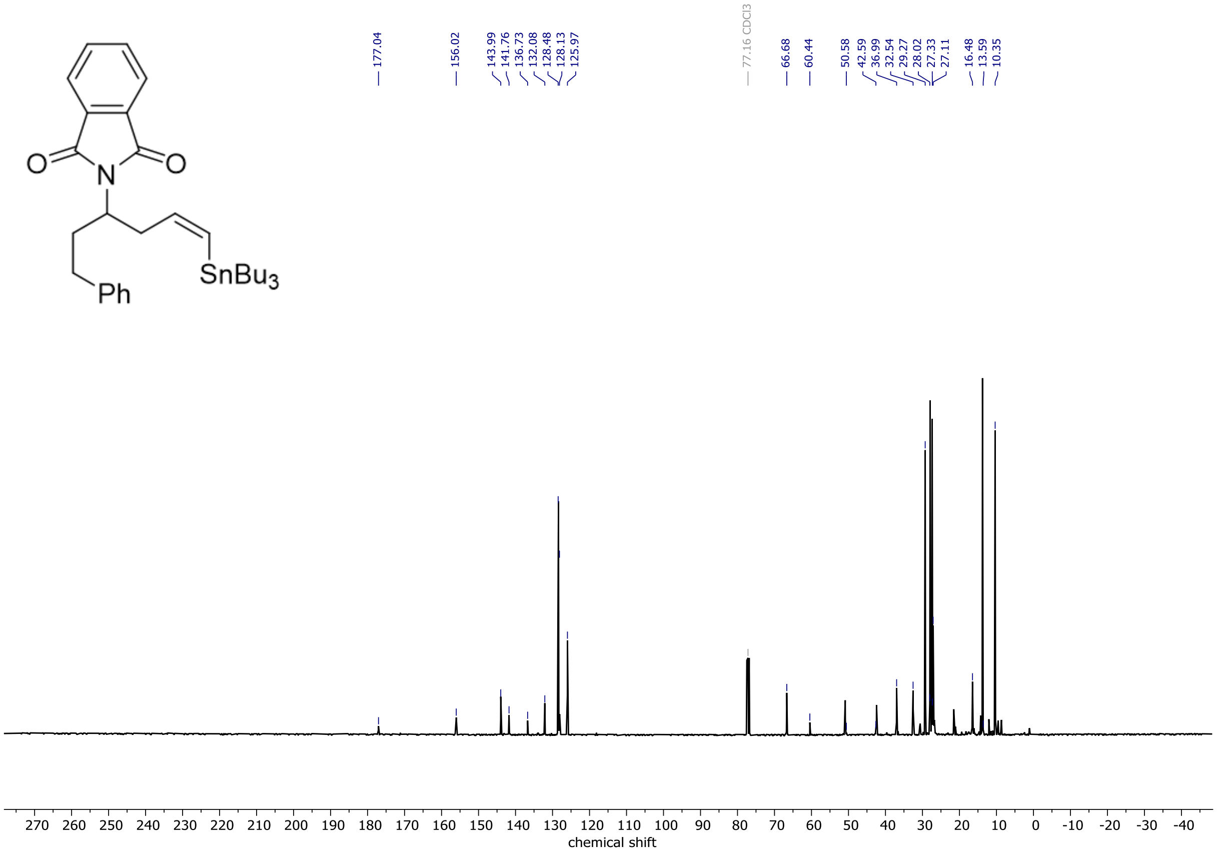
SnBu3
Ph
177.04
156.02
143.99 141.76 136.73 132.08 128.48 128.13 125.97
77.16 CDCl3
66.68 60.44
50.58 42.59 36.99 32.54 29.27 28.02 27.33 27.11
16.48 13.59 10.35
chemical shift

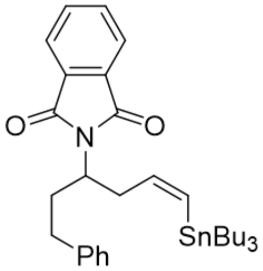

O
N
O
SnBu3
Ph

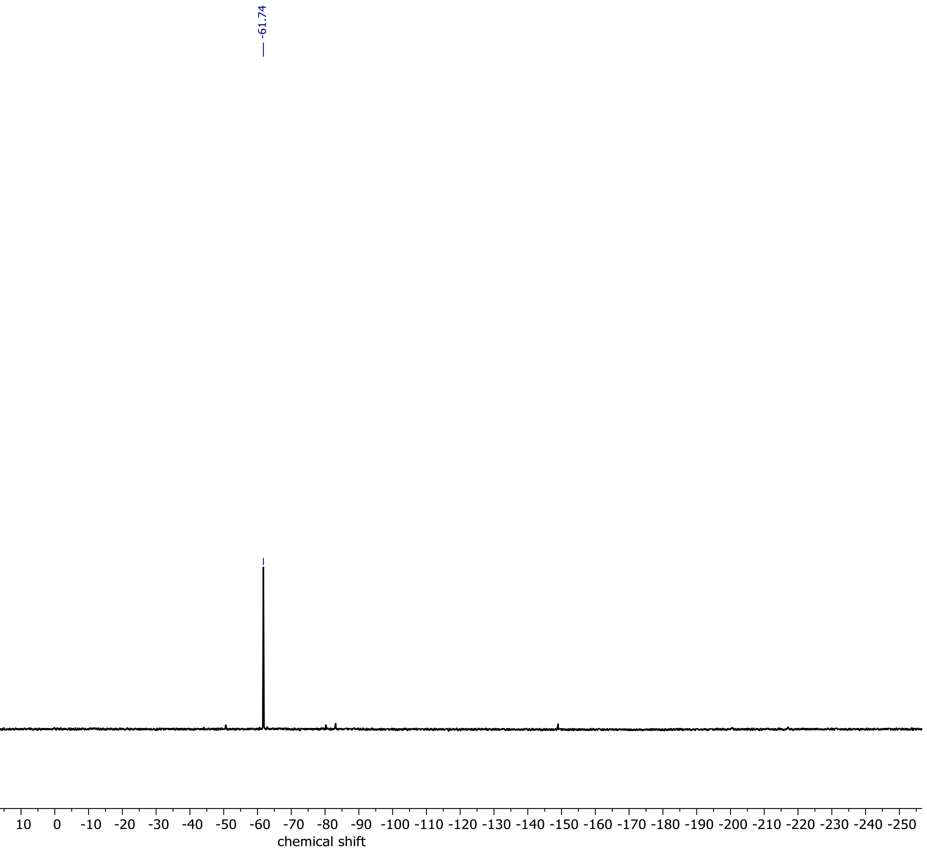

-61.74
90 80 70 60 50 40 30 20 10 0 -10 -20 -30 -40 -50 -60 -70 -80 -90 -100 -110 -120 -130 -140 -150 -160 -170 -180 -190 -200 -210 -220 -230 -240 -250
chemical shift

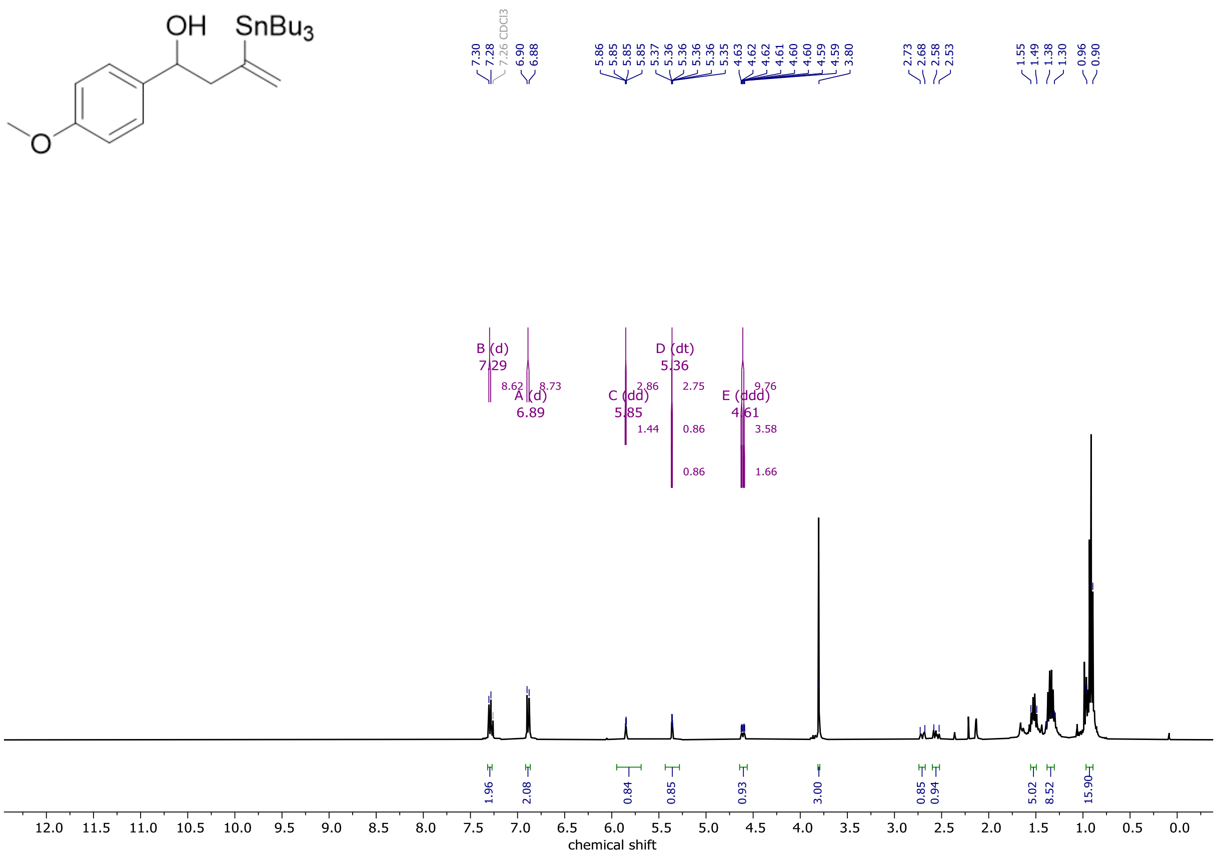

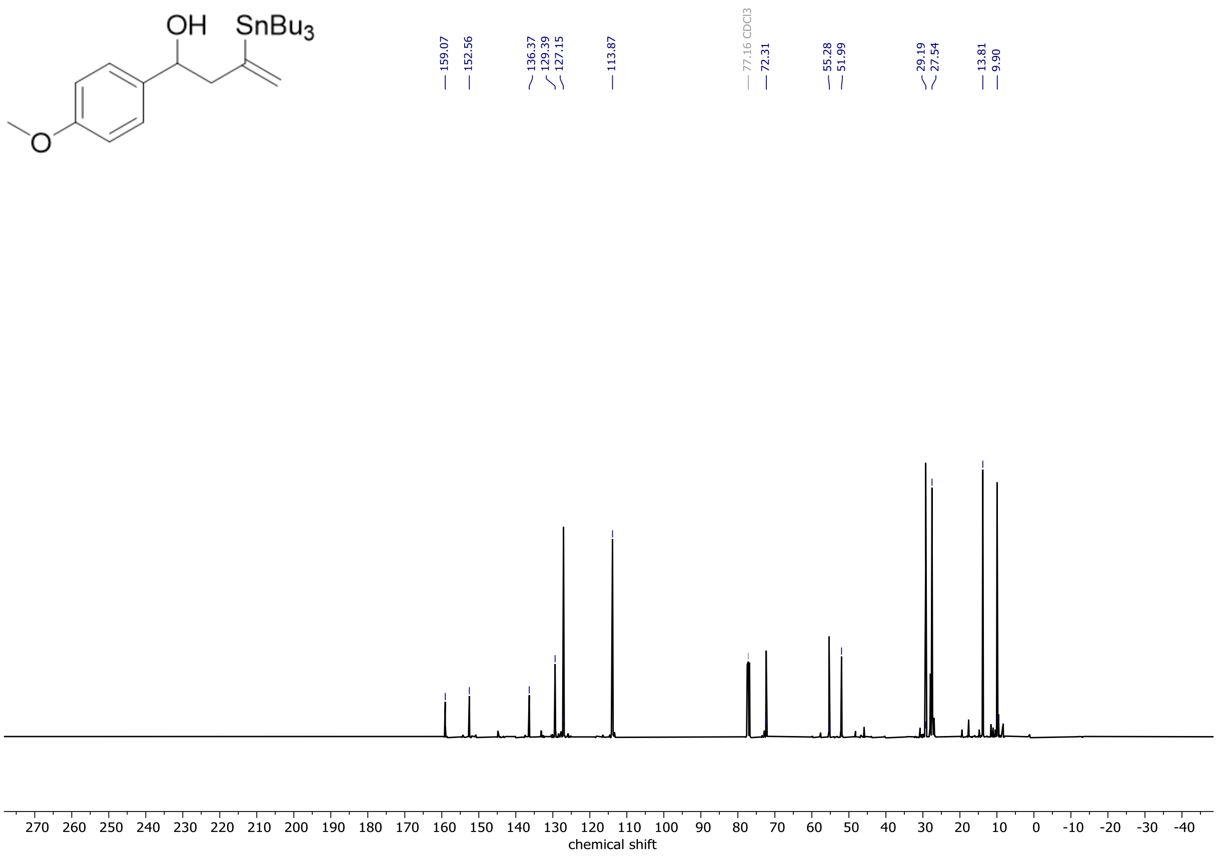

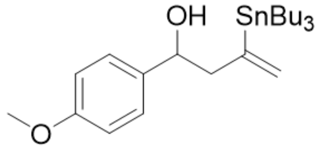
OH
SnBu3

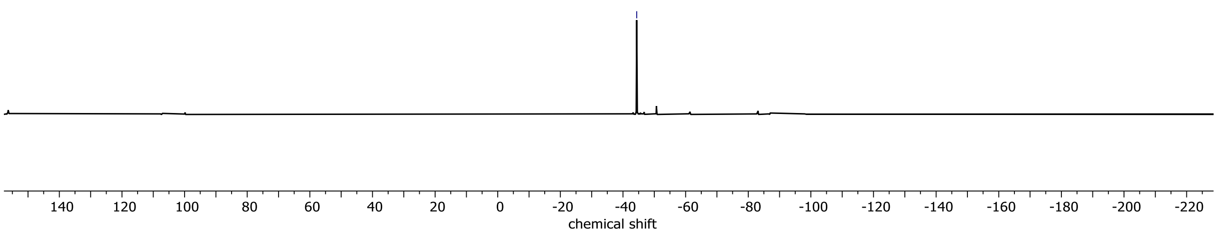
-44.39
140
120
100
80
60
40
20
0
-20
-40
-60
-80
-100
-120
-140
-160
-180
-200
-220
chemical shift

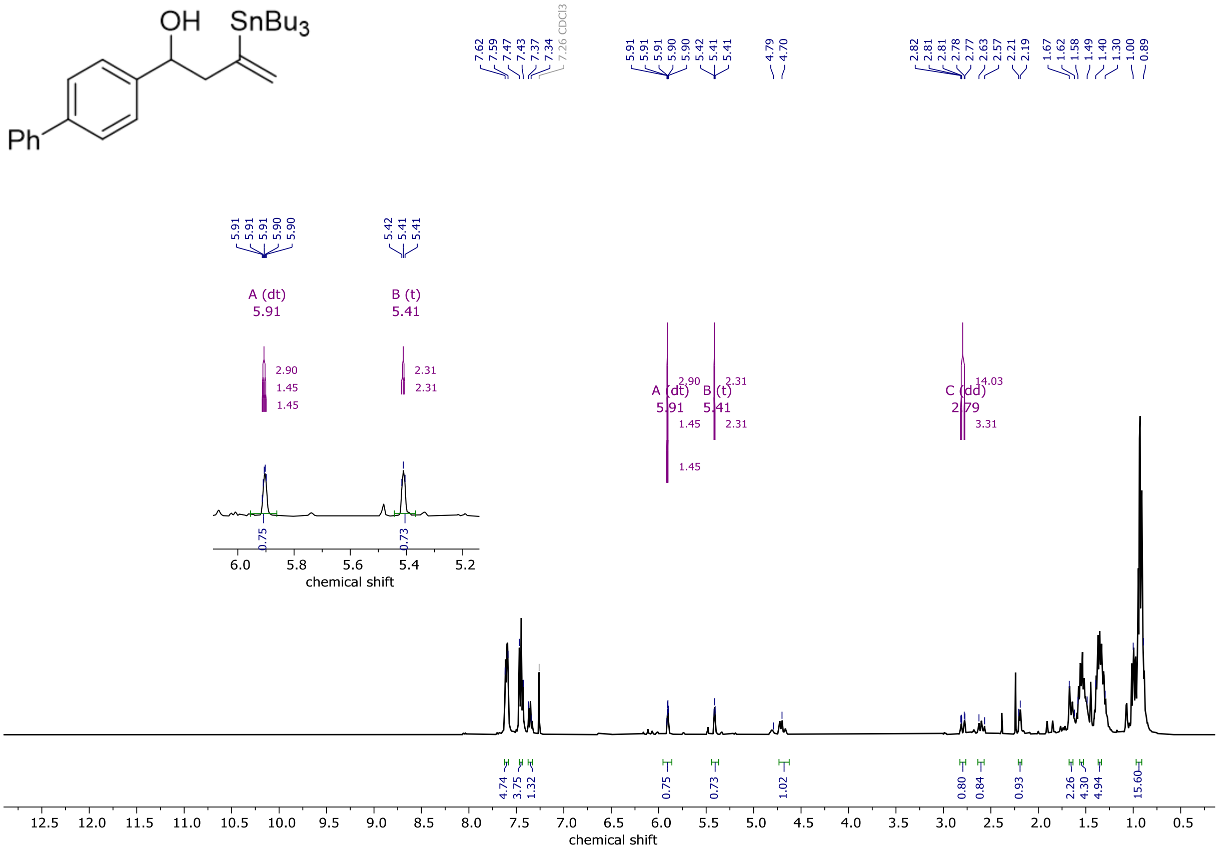
OH
SnBu3
Ph
7.62 7.59 7.47 7.43 7.37 7.34 7.26 CDCl3
5.91 5.91 5.91 5.90 5.90 5.42 5.41 5.41
4.79 4.70
2.82 2.81 2.81 2.78 2.77 2.63 2.57 2.21 2.19 1.67 1.62 1.58 1.49 1.40 1.30 1.00 0.89
A (dt)
5.91
B (t)
5.41
C (dd)
2.79
4.74 3.75 1.32 0.75 0.73 1.02 0.80 0.84 0.93 2.26 4.30 4.94 15.60
chemical shift

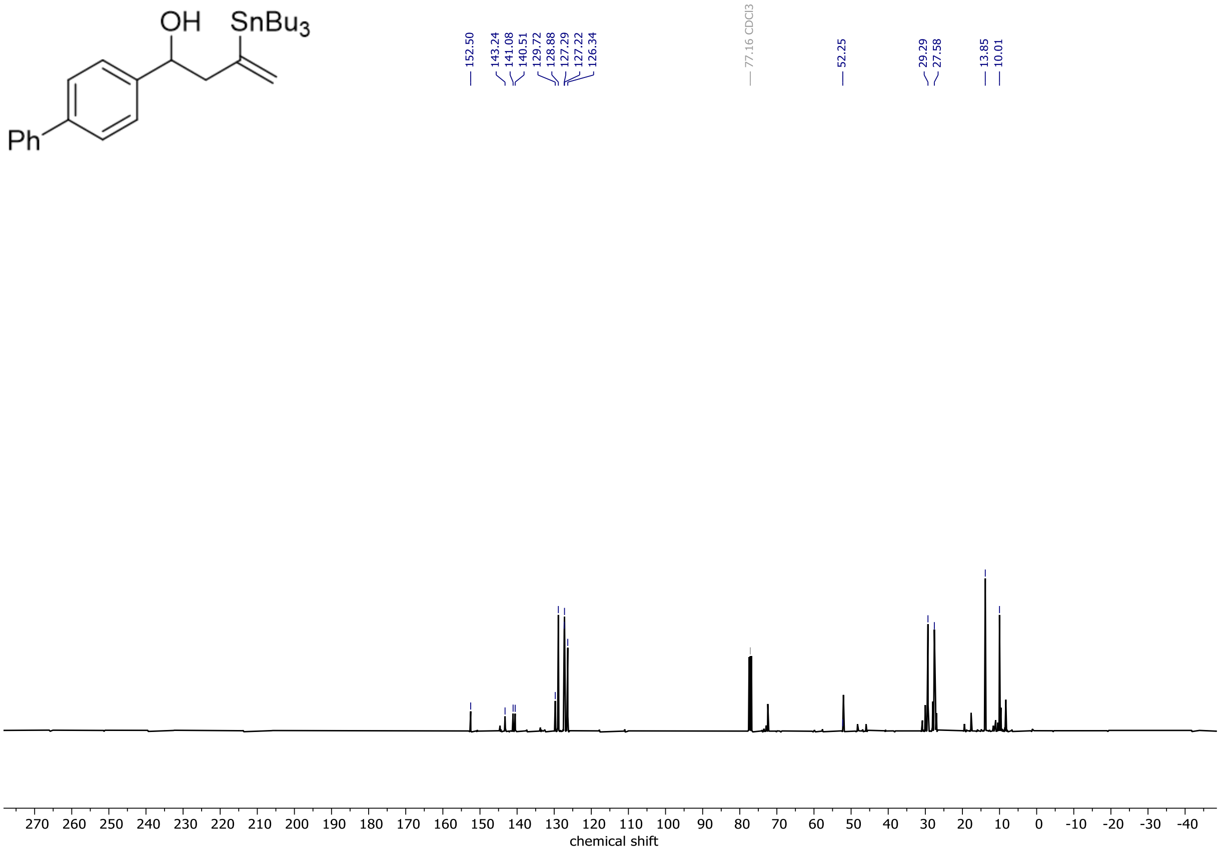
OH
SnBu3
Ph
152.50 143.24 141.08 140.51 129.72 128.88 127.29 127.22 126.34
77.16 CDCl3
52.25
29.29 27.58
13.85 10.01
chemical shift

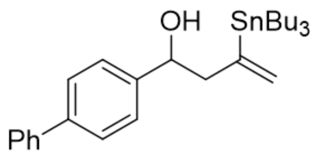
OH
SnBu3
Ph

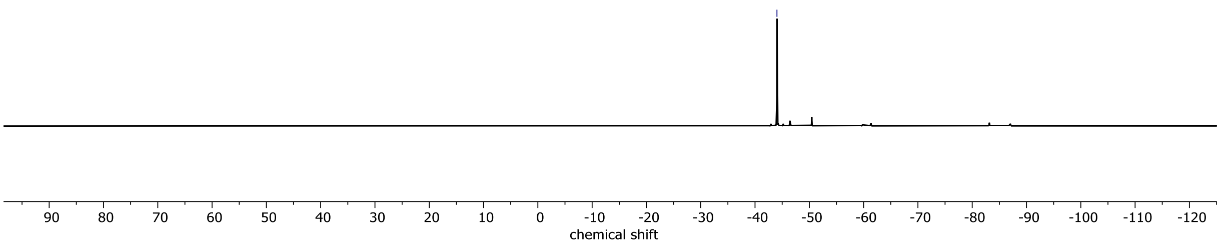
-44.04
90 80 70 60 50 40 30 20 10 0 -10 -20 -30 -40 -50 -60 -70 -80 -90 -100 -110 -120
chemical shift

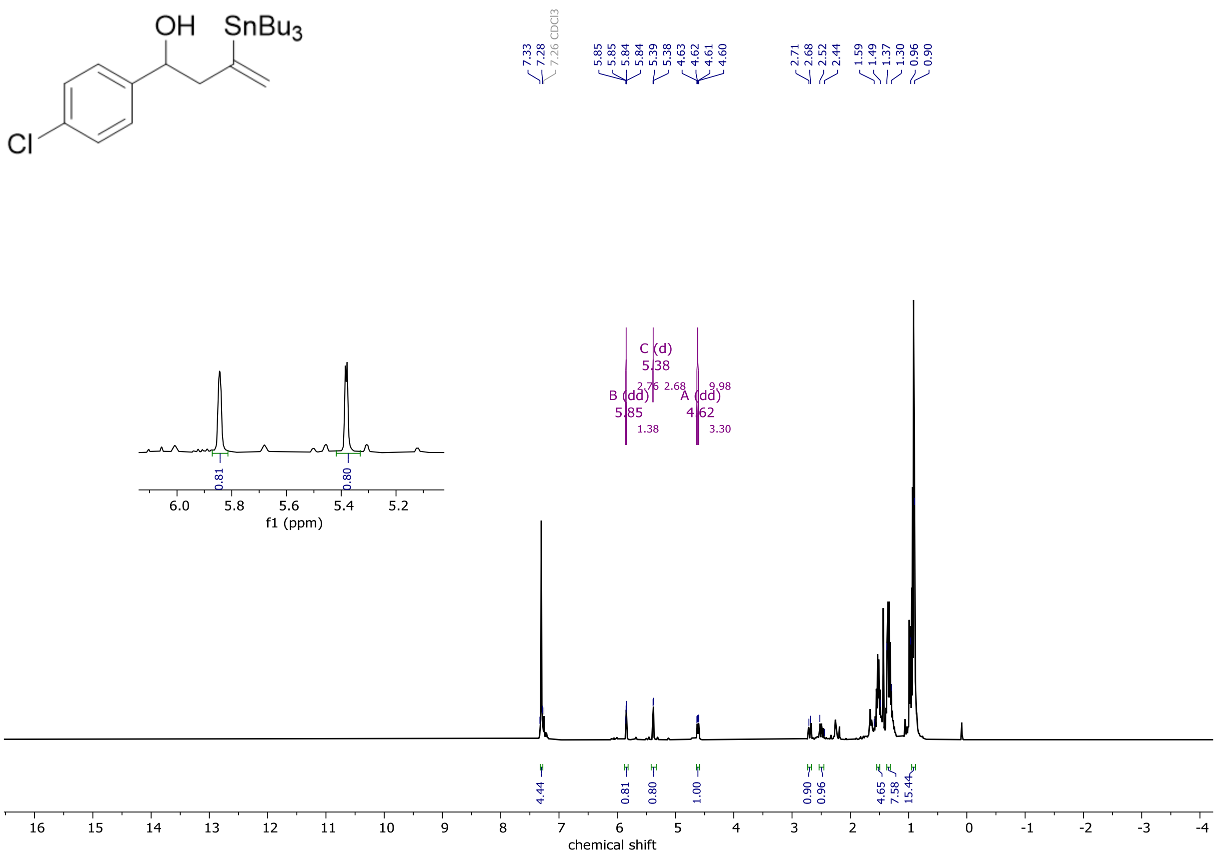
OH
SnBu3
Cl
7.33
7.28
7.26 CDCl3
5.85
5.85
5.84
5.84
5.39
5.38
4.63
4.62
4.61
4.60
2.71
2.68
2.52
2.44
1.59
1.49
1.37
1.30
0.96
0.90
C (d)
5.38
B (dd)
5.85
A (dd)
4.62
4.44
0.81
0.80
1.00
0.90
0.96
4.65
7.58
15.44
f1 (ppm)
chemical shift

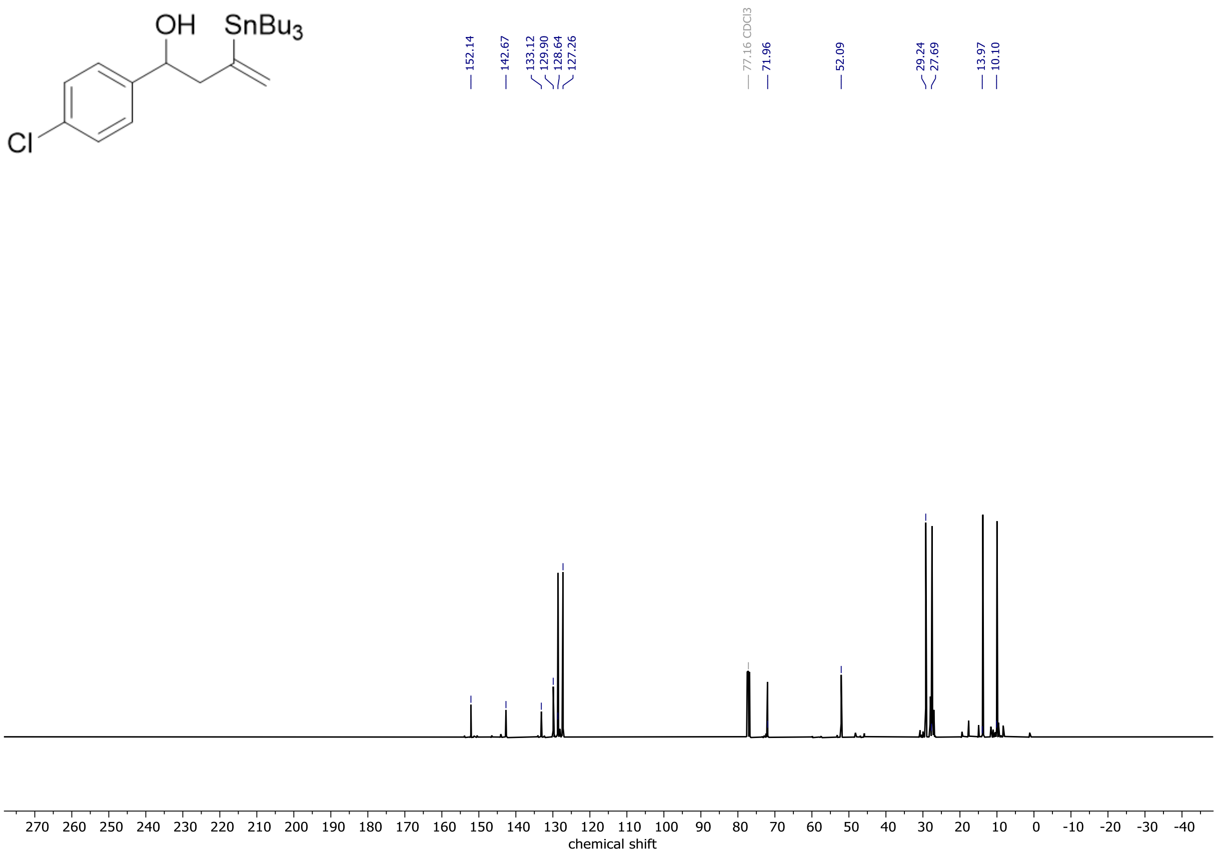
OH
SnBu3
Cl
152.14
142.67
133.12
129.90
128.64
127.26
77.16 CDCl3
71.96
52.09
29.24
27.69
13.97
10.10
chemical shift

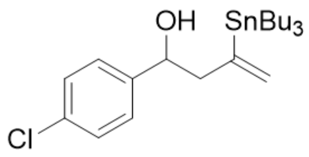
OH
SnBu3
Cl

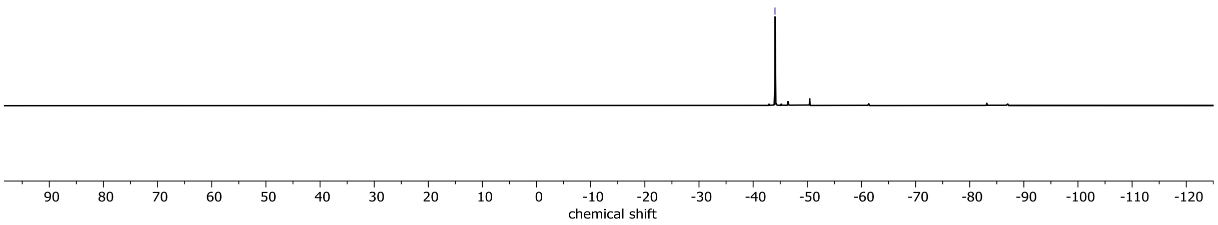
-44.04
90
80
70
60
50
40
30
20
10
0
-10
-20
-30
-40
-50
-60
-70
-80
-90
-100
-110
-120
chemical shift

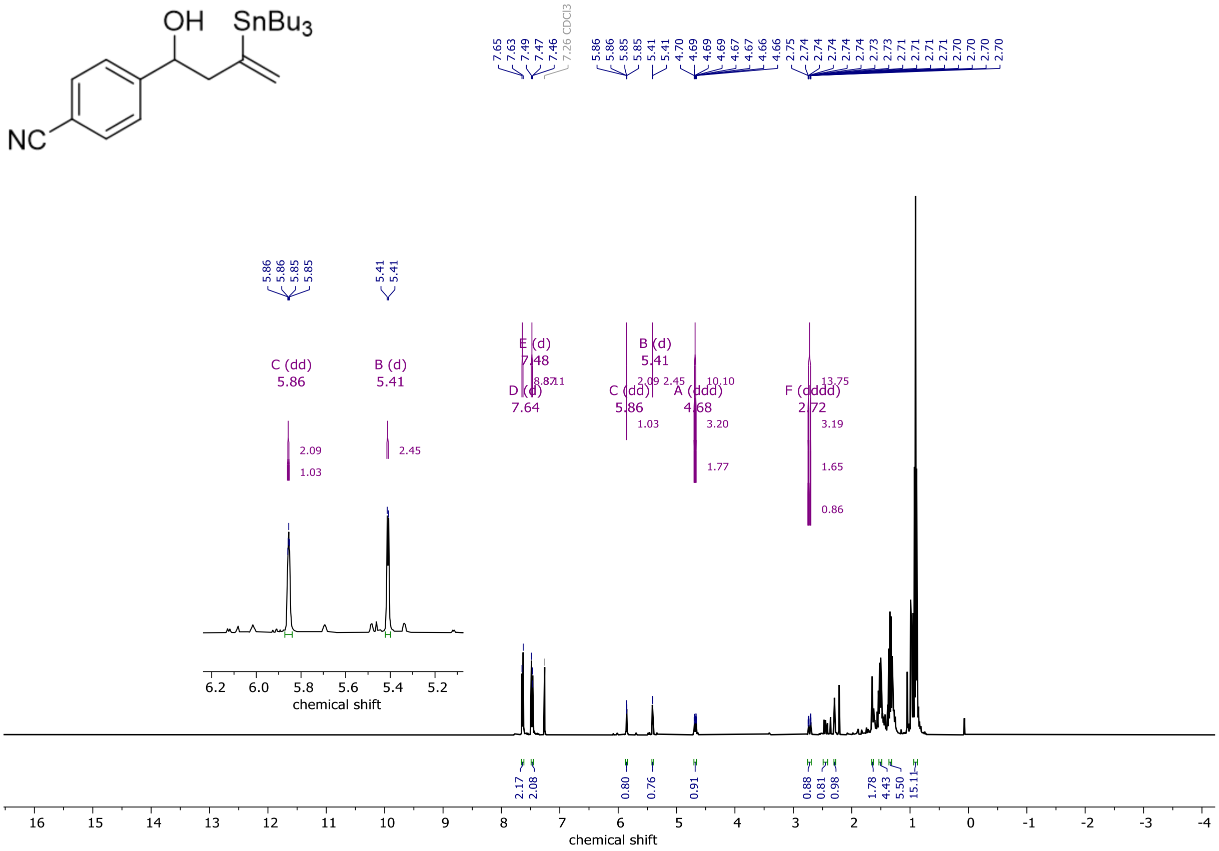

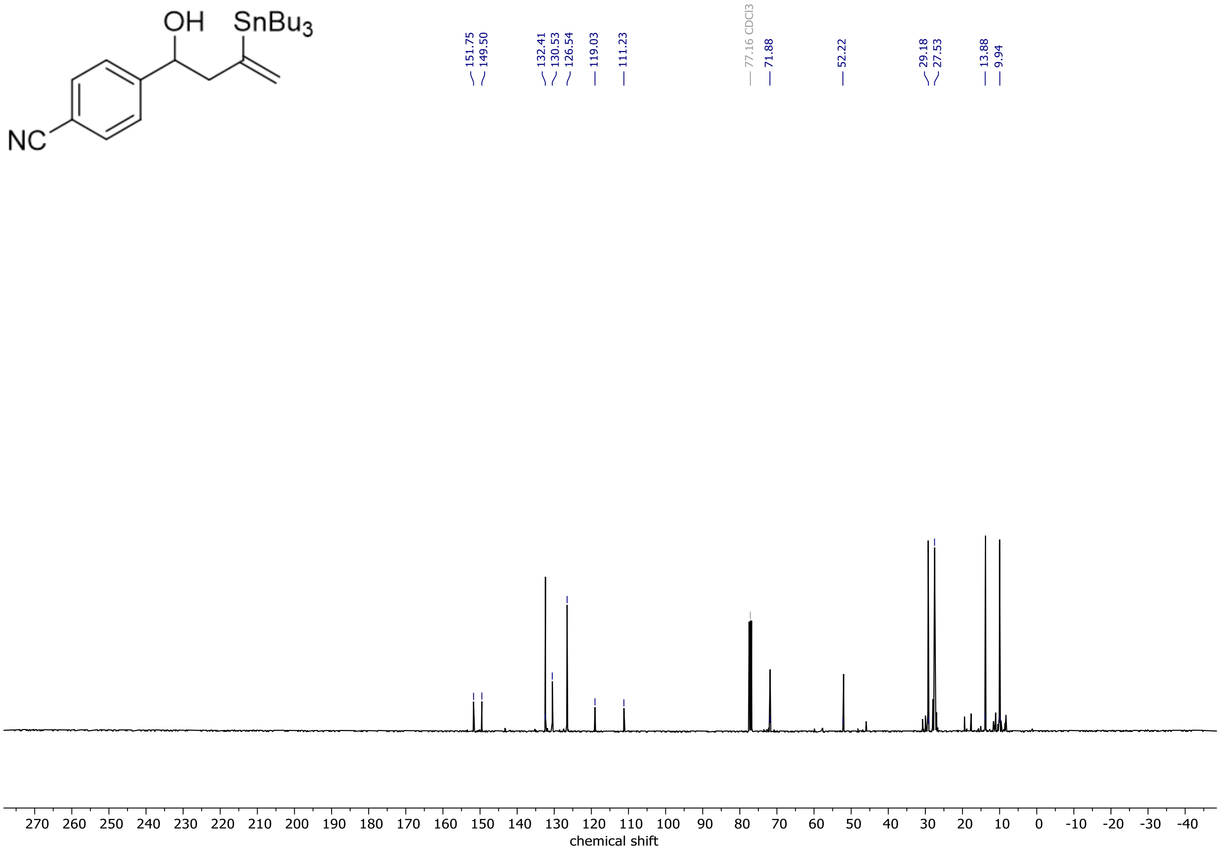

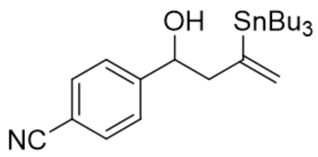
OH
SnBu3
NC

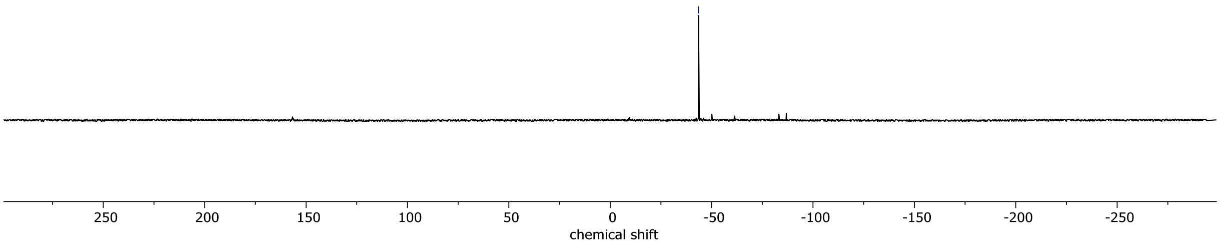
-43.54
250
200
150
100
50
0
-50
-100
-150
-200
-250
chemical shift

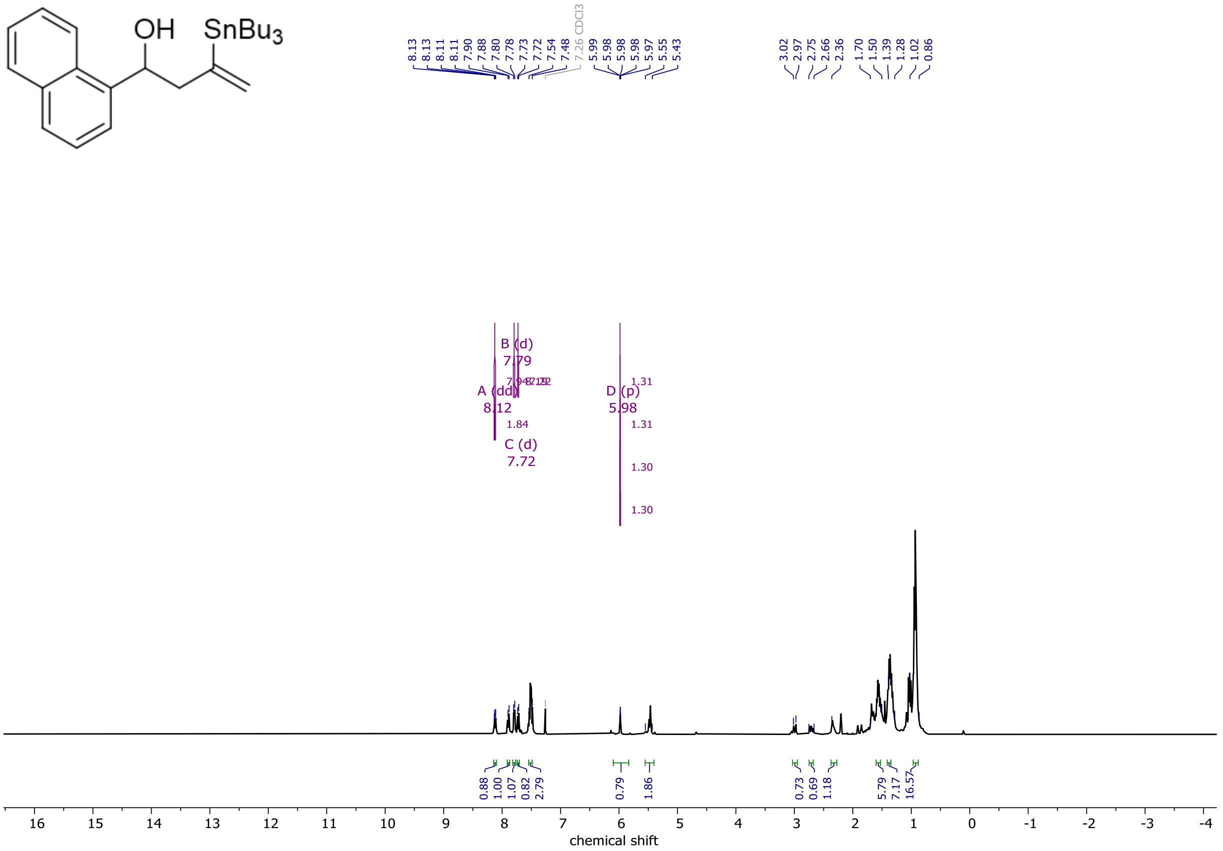
OH SnBu3
8.13 8.13 8.11 8.11 7.90 7.88 7.80 7.78 7.73 7.72 7.54 7.48 7.26 CDCl3 5.99 5.98 5.98 5.98 5.98 5.97 5.55 5.43
3.02 2.97 2.75 2.66 2.36 1.70 1.50 1.39 1.28 1.02 0.86
B (d) 7.79
A (dd) 8.12
C (d) 7.72
D (p) 5.98
0.88 1.00 1.07 0.82 2.79 0.79 1.86 0.73 0.69 1.18 5.79 7.17 16.57
chemical shift

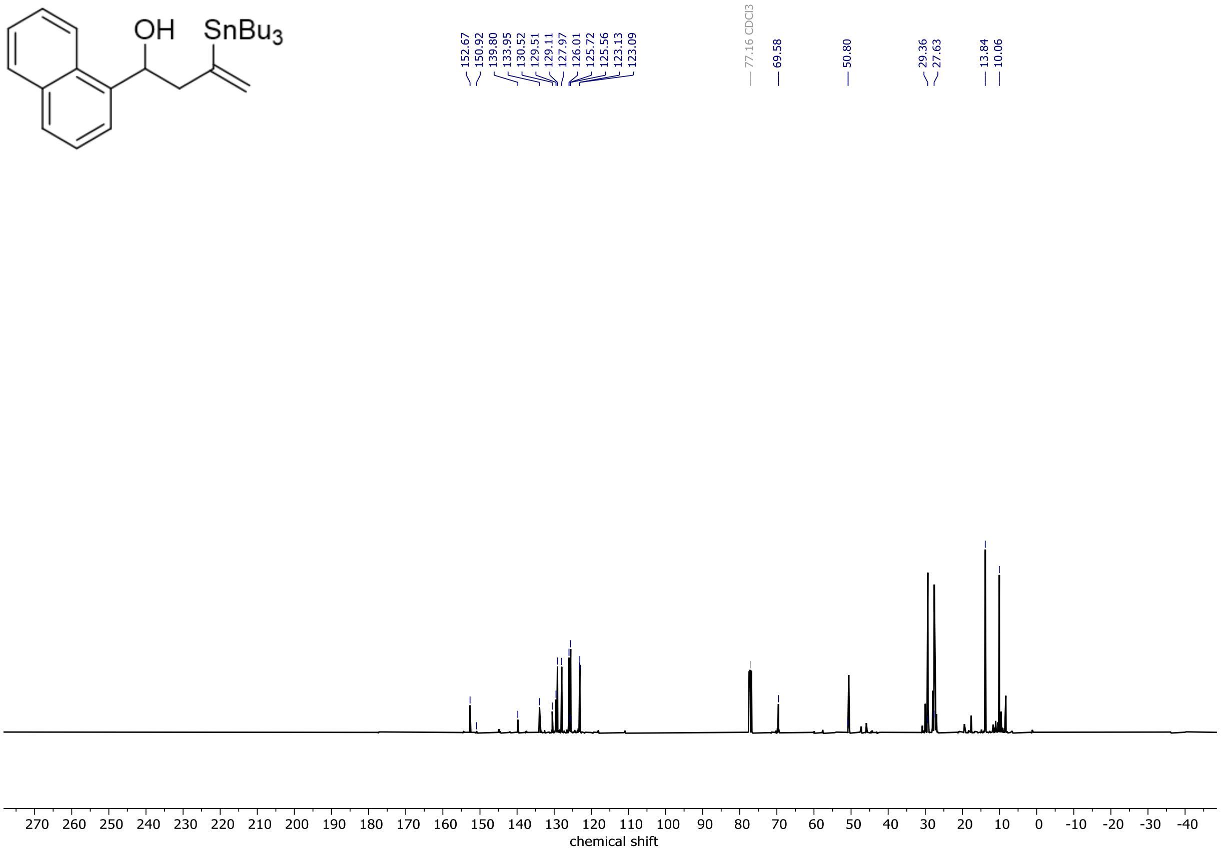
OH SnBu3
152.67 150.92 139.80 133.95 130.52 129.51 129.11 127.97 126.01 125.72 125.56 123.13 123.09
77.16 CDCl3 69.58 50.80 29.36 27.63 13.84 10.06
chemical shift

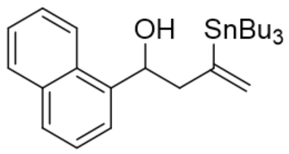
OH
SnBu3

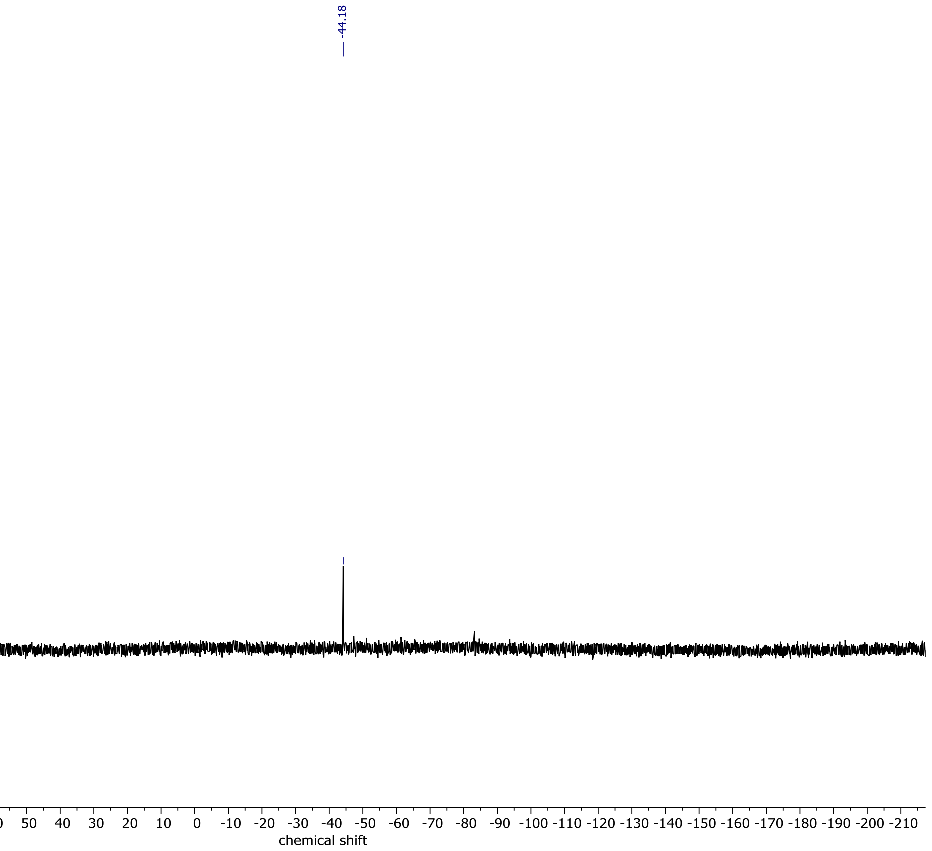
-44.18
140 130 120 110 100 90 80 70 60 50 40 30 20 10 0 -10 -20 -30 -40 -50 -60 -70 -80 -90 -100 -110 -120 -130 -140 -150 -160 -170 -180 -190 -200 -210
chemical shift

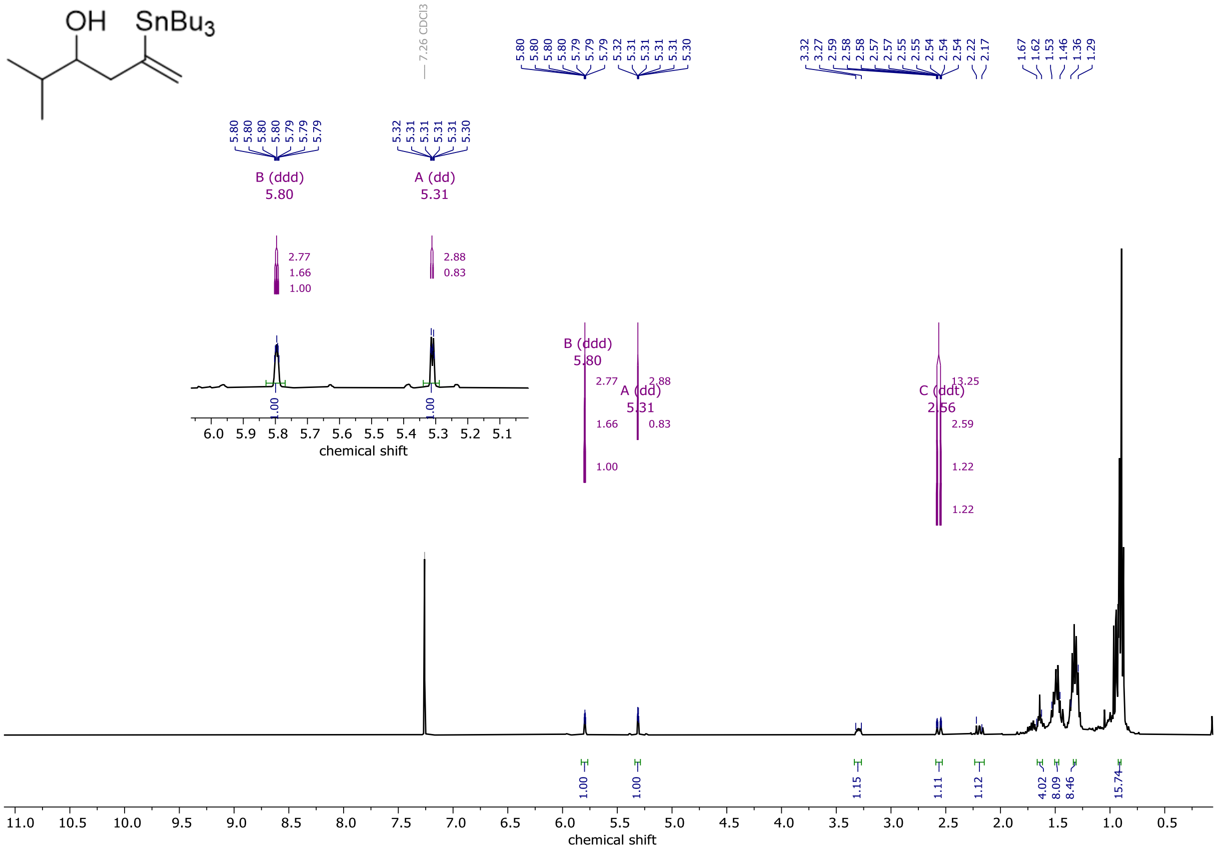
OH SnBu3
7.26 CDCl3
5.80 5.80 5.80 5.80 5.79 5.79 5.79 5.32 5.31 5.31 5.31 5.31 5.30
3.32 3.27 2.59 2.58 2.58 2.57 2.57 2.55 2.55 2.54 2.54 2.54 2.22 2.17
1.67 1.62 1.53 1.46 1.36 1.29
B (ddd)
5.80
A (dd)
5.31
C (ddt)
2.56
1.00 1.00 1.15 1.11 1.12 4.02 8.09 8.46 15.74
chemical shift

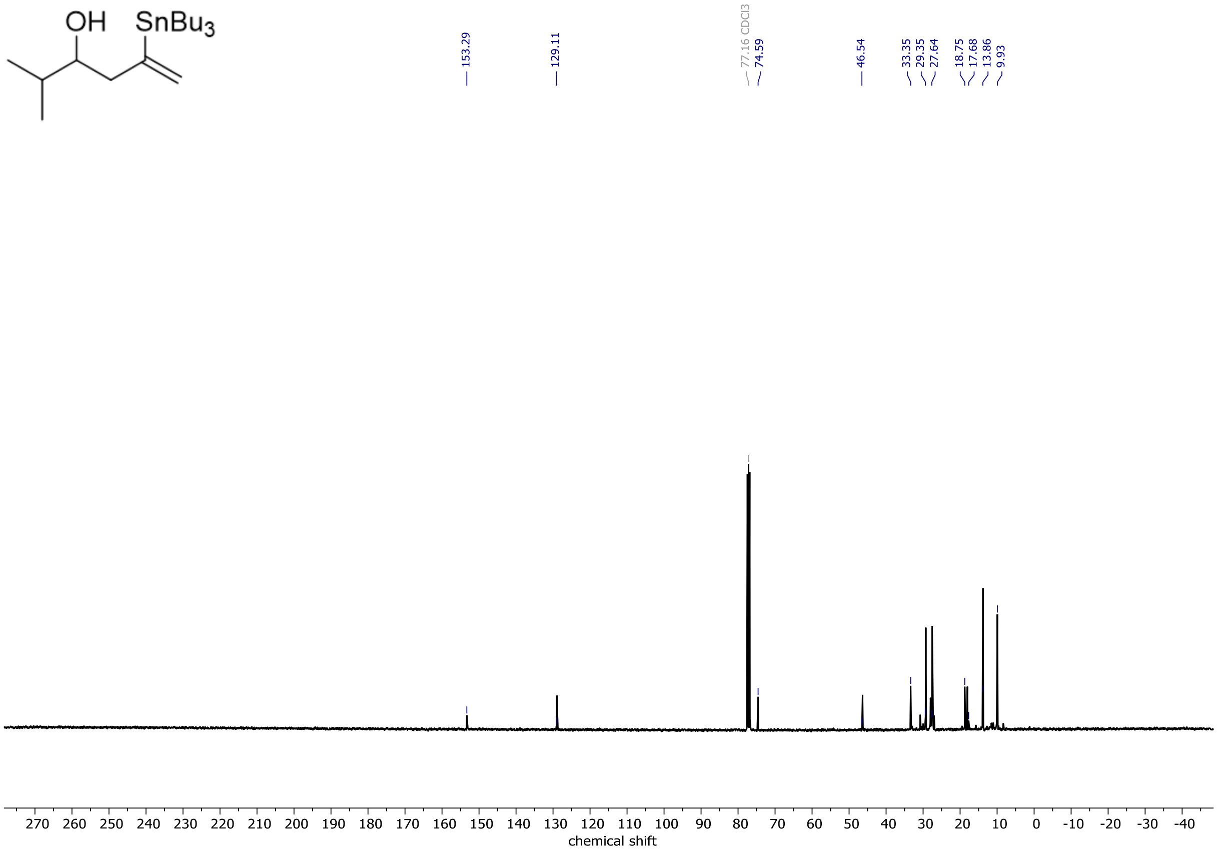
OH SnBu3
153.29
129.11
77.16 CDCl3
74.59
46.54
33.35 29.35 27.64
18.75 17.68 13.86 9.93
chemical shift

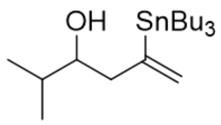
OH
SnBu3

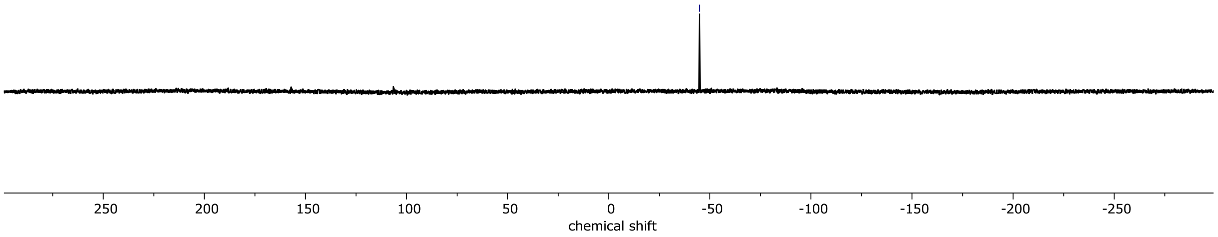
-44.89
250
200
150
100
50
0
-50
-100
-150
-200
-250
chemical shift

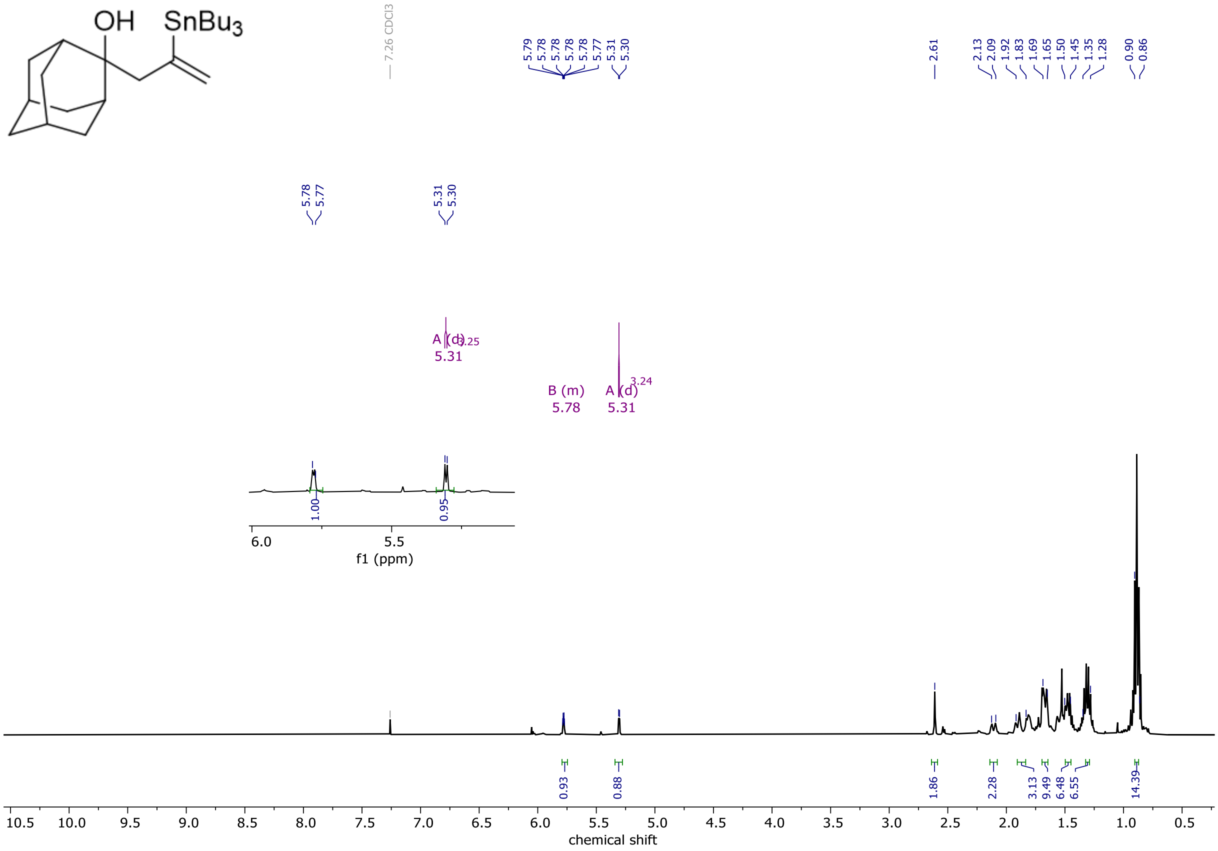
OH SnBu3
7.26 CDCl3
5.79 5.78 5.78 5.78 5.78 5.77 5.31 5.30
2.61
2.13 2.09 1.92 1.83 1.69 1.65 1.50 1.45 1.35 1.28
0.90 0.86
5.78 5.77
5.31 5.30
A (d) 5.31
B (m) 5.78
A (d) 5.31
1.00
0.95
6.0
5.5
f1 (ppm)
0.93
0.88
1.86
2.28
3.13
9.49
6.48
6.55
14.39
10.5 10.0 9.5 9.0 8.5 8.0 7.5 7.0 6.5 6.0 5.5 5.0 4.5 4.0 3.5 3.0 2.5 2.0 1.5 1.0 0.5
chemical shift

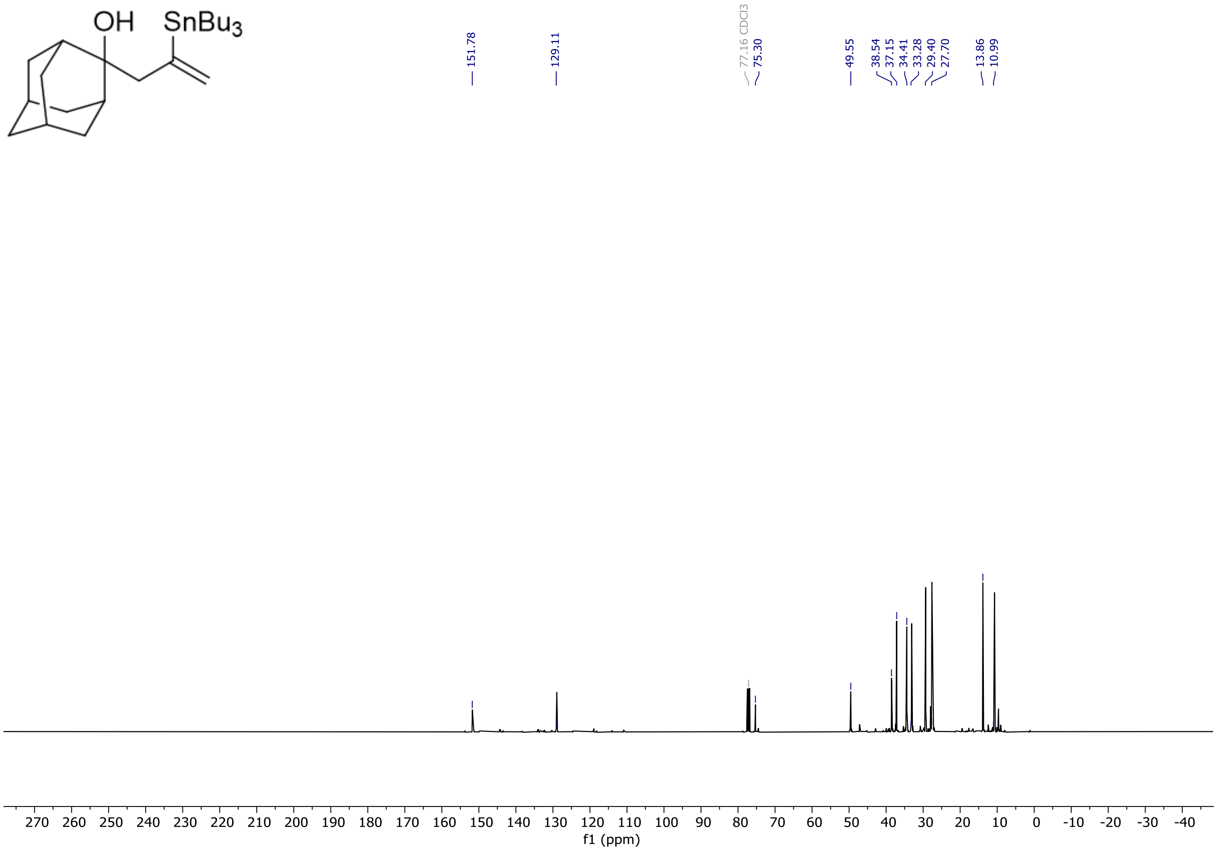
OH SnBu3
151.78
129.11
77.16 CDCl3
75.30
49.55
38.54 37.15 34.41 33.28 29.40 27.70
13.86 10.99
270 260 250 240 230 220 210 200 190 180 170 160 150 140 130 120 110 100 90 80 70 60 50 40 30 20 10 0 -10 -20 -30 -40
f1 (ppm)

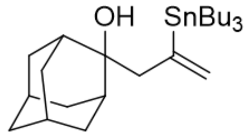
OH
SnBu3

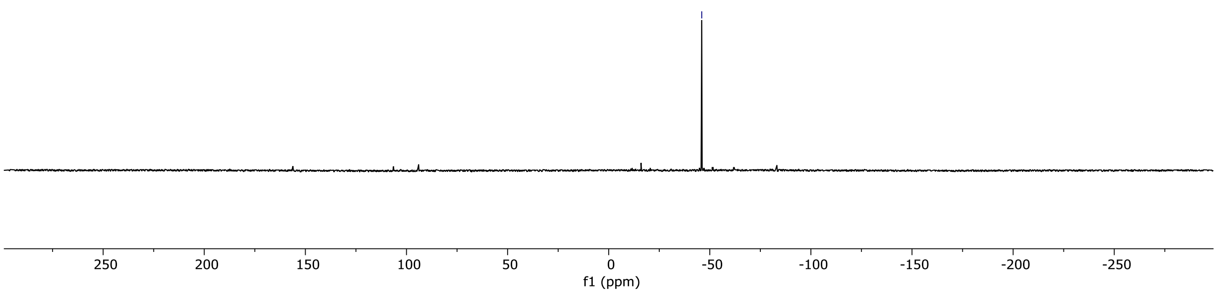
-46.02
250
200
150
100
50
0
-50
-100
-150
-200
-250
f1 (ppm)

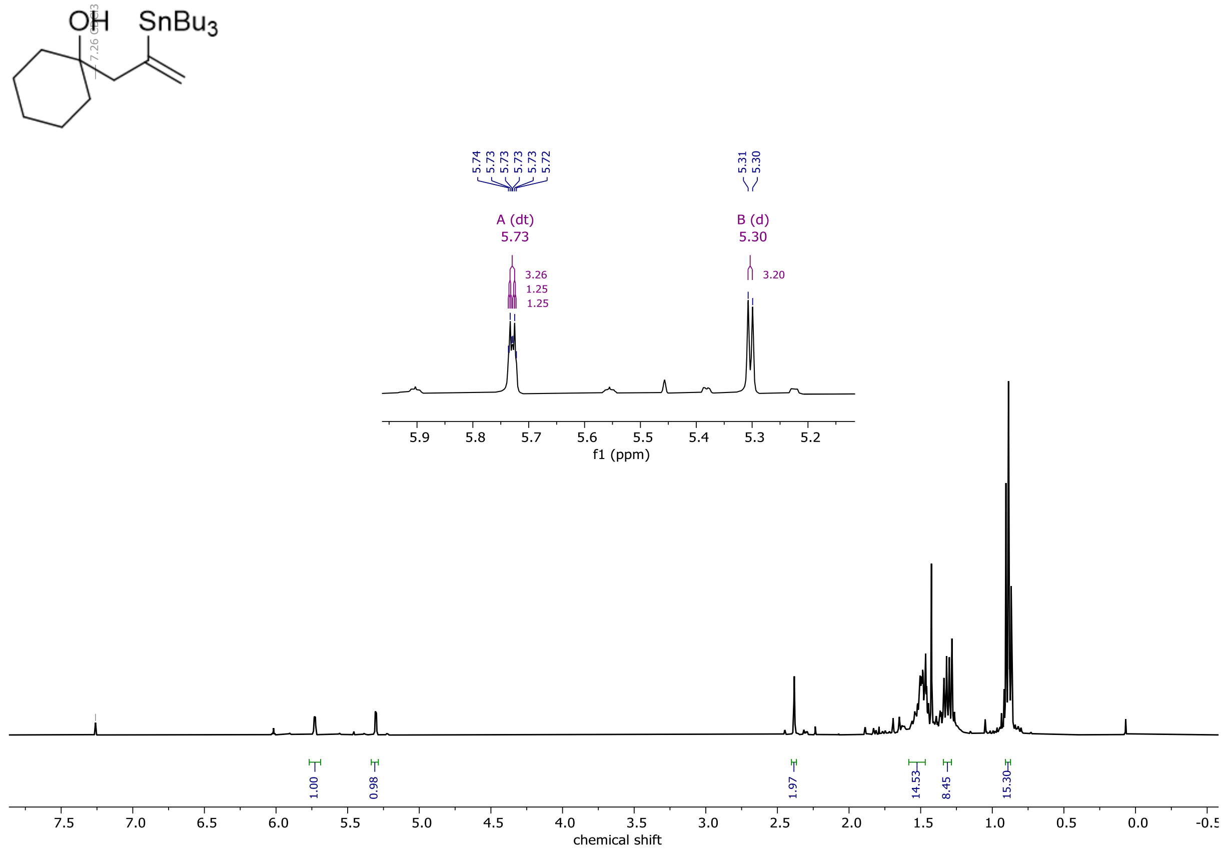

OH
SnBu3
7.26 CDCl3
5.74
5.73
5.73
5.73
5.73
5.72
5.31
5.30
A (dt)
5.73
3.26
1.25
1.25
B (d)
5.30
3.20
f1 (ppm)
1.00
0.98
1.97
14.53
8.45
15.30
chemical shift

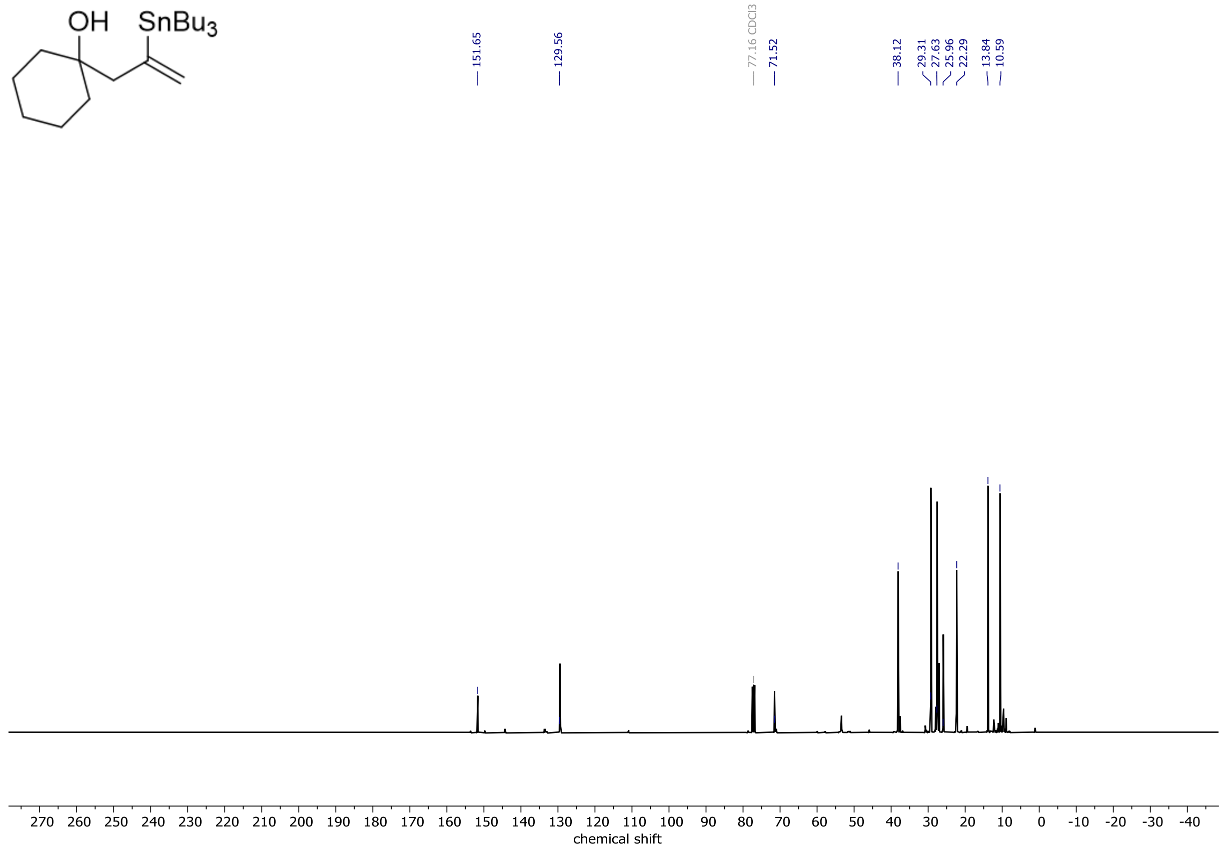

OH
SnBu3
151.65
129.56
77.16 CDCl3
71.52
38.12
29.31
27.63
25.96
22.29
13.84
10.59
chemical shift

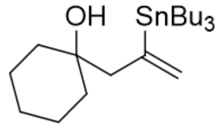
OH
SnBu3

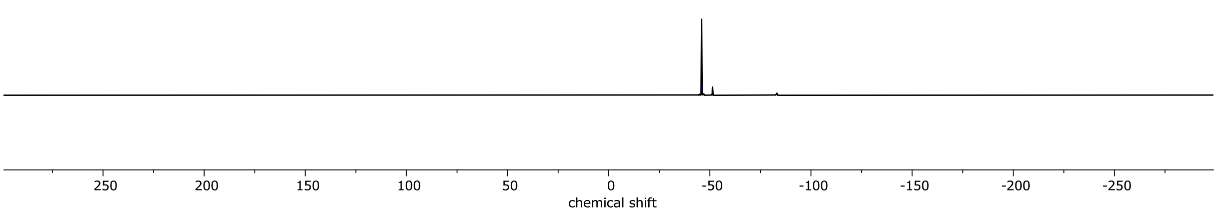
-46.11
250
200
150
100
50
0
-50
-100
-150
-200
-250
chemical shift

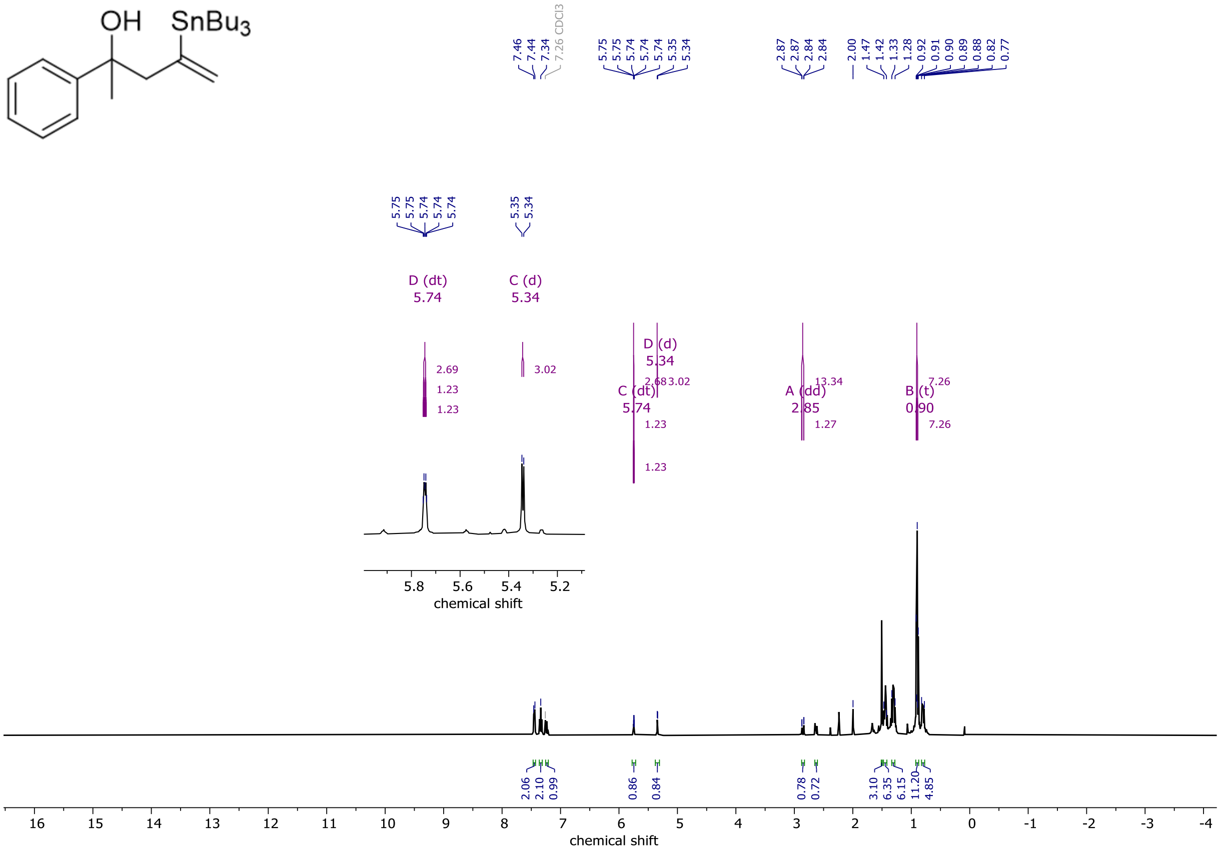
OH
SnBu3
7.46
7.44
7.34
7.26 CDCl3
5.75
5.75
5.74
5.74
5.74
5.35
5.34
2.87
2.87
2.84
2.84
2.00
1.47
1.42
1.33
1.28
0.92
0.91
0.90
0.89
0.88
0.82
0.77
D (dt)
5.74
C (d)
5.34
2.69
1.23
1.23
3.02
A (dd)
2.85
13.34
1.27
B (t)
0.90
7.26
7.26
5.8
5.6
5.4
5.2
chemical shift
2.06
2.10
0.99
0.86
0.84
0.78
0.72
3.10
6.35
6.15
11.20
4.85
16
15
14
13
12
11
10
9
8
7
6
5
4
3
2
1
0
-1
-2
-3
-4
chemical shift

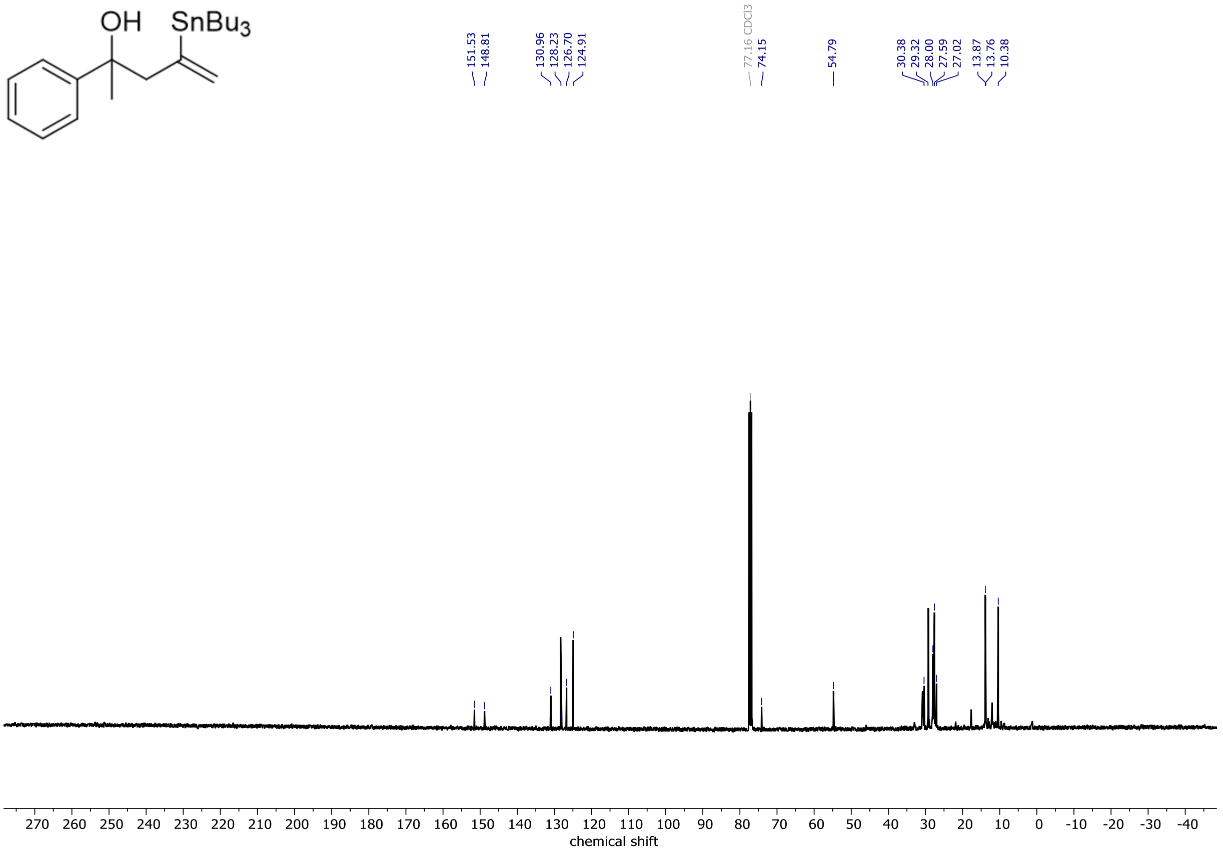
OH
SnBu3
151.53
148.81
130.96
128.23
126.70
124.91
77.16 CDCl3
74.15
54.79
30.38
29.32
28.00
27.59
27.02
13.87
13.76
10.38
270
260
250
240
230
220
210
200
190
180
170
160
150
140
130
120
110
100
90
80
70
60
50
40
30
20
10
0
-10
-20
-30
-40
chemical shift

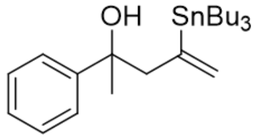
OH
SnBu3

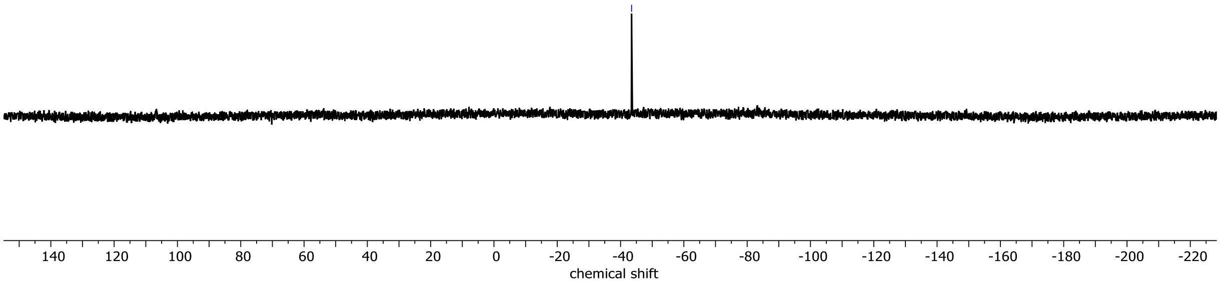
-43.49
140
120
100
80
60
40
20
0
-20
-40
-60
-80
-100
-120
-140
-160
-180
-200
-220
chemical shift

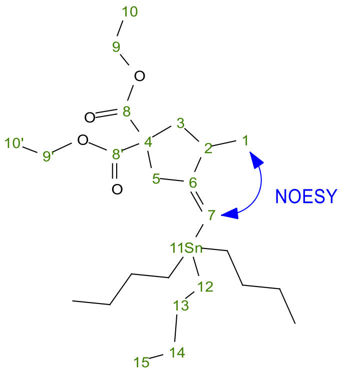

Atom	δ (ppm)	Min..Max (ppm)	J	COSY	HSQC	HMBC	NOESY
1 C	18.57	18.56..18.57			1	3a	
H3	1.09	1.08..1.10	6.30(?)	2, 3a	1	3, 6	7
2 C	39.82	39.81..39.82			2	3a, 3b, 5b, 7	
H	2.57	2.57..2.57		1	2	4, 5, 7, 11	
3 C	42.33	42.33..42.33			3a, 3b	1, 5b	
Ha	1.74	1.72..1.77		1, 3b	3	1, 2, 4, 6, 8, 8'	
Hb	2.57	2.57..2.57		3a	3	2, 4, 5, 6, 8, 8'	
4 C	58.41	58.40..58.41				2, 3a, 3b, 5a, 5b	
5 C	42.63	42.62..42.63			5a, 5b	2, 3b, 7	
Ha	2.88	2.86..2.91	16.80(5b), 2.30(?), 2.30(?	5b, 7	5	4, 6, 7, 8, 8', 11	
Hb	2.99	2.97..3.02	16.80(5a)	5a, 7	5	2, 3, 4, 6, 7, 8, 8', 11	
6 C	162.78	162.77..162.78				1, 3a, 3b, 5a, 5b, 7	
7 C	116.92	116.91..116.92	392.60(?), 375.01(?)		7	2, 5a, 5b, 12	
H	5.61	5.60..5.62	63.10(11Sn)	5a, 5b	7	2, 5, 6, 11	1
8 C	172.13	172.12..172.13				3a, 3b, 5a, 5b, 9	
8' C	172.11	172.10..172.11				3a, 3b, 5a, 5b, 9'	
9 C	61.53	61.52..61.54			9	10	
H2	4.18	4.16..4.20		10	9	8, 10	

Atom	δ (ppm)	Min..Max (ppm)	J	COSY	HSQC	HMBC	NOESY
9' C	61.53	61.52..61.54			9'	10'	
H2	4.18	4.16..4.20		10'	9'	8', 10'	
10 C	14.18	14.18..14.19			10	9	
H3	1.25	1.24..1.25		9	10	9	
10' C	14.18	14.18..14.19			10'	9'	
H3	1.24	1.24..1.25		9'	10'	9'	
11 Sn	-55.73	-55.74..-55.73	63.10(7H), 392.60(?), 375.01(?)			2, 5a, 5b, 7, 12, 13	
12 C	9.90	9.90..9.91			12	12, 13, 14	
H2	0.91	0.91..0.91		13	12	7, 11, 12, 13, 14	
13 C	29.33	29.33..29.34			13	12, 14, 15	
H2	1.49	1.49..1.50		12, 14	13	11, 12, 14, 15	
14 C	27.44	27.43..27.44			14	12, 13, 15	
H2	1.31	1.31..1.31		13, 15	14	12, 13, 15	
15 C	13.82	13.81..13.83			15	13, 14	
H3	0.89	0.86..0.91		14	15	13, 14	

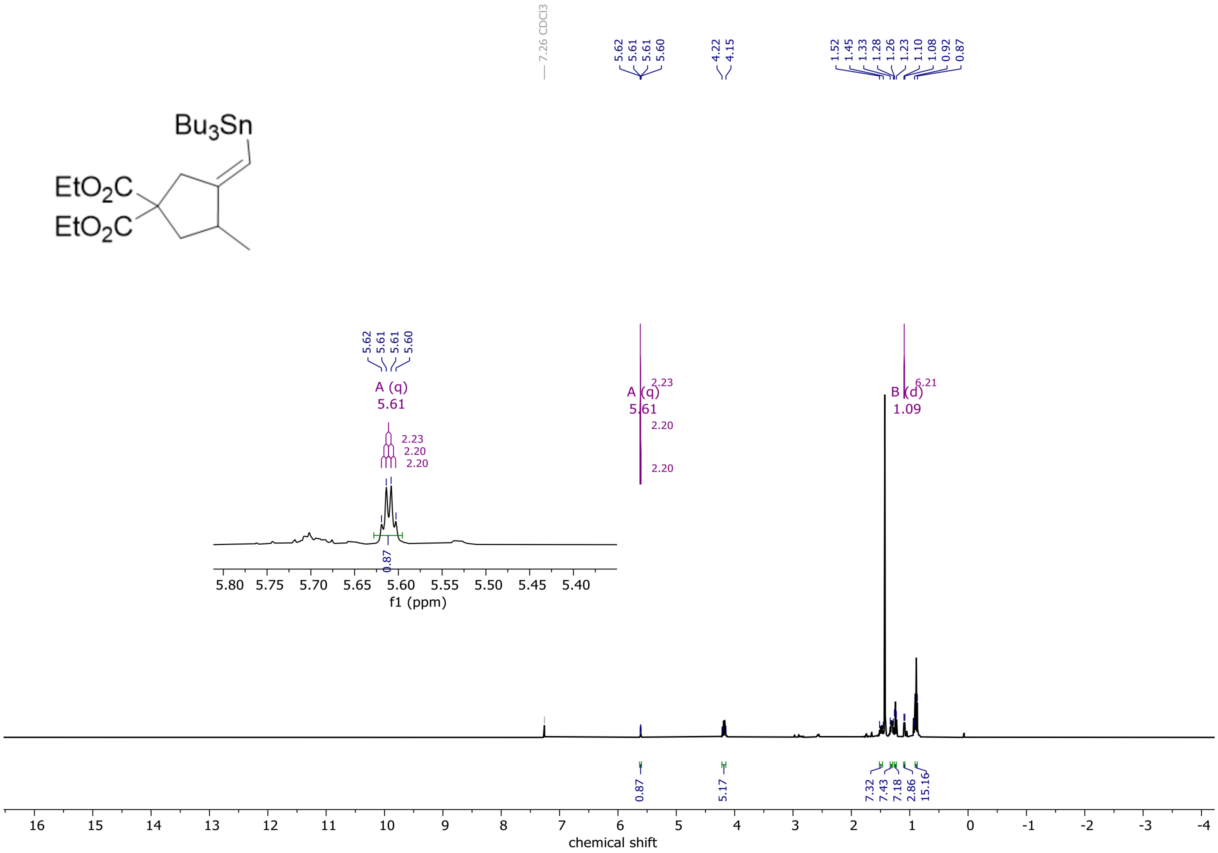

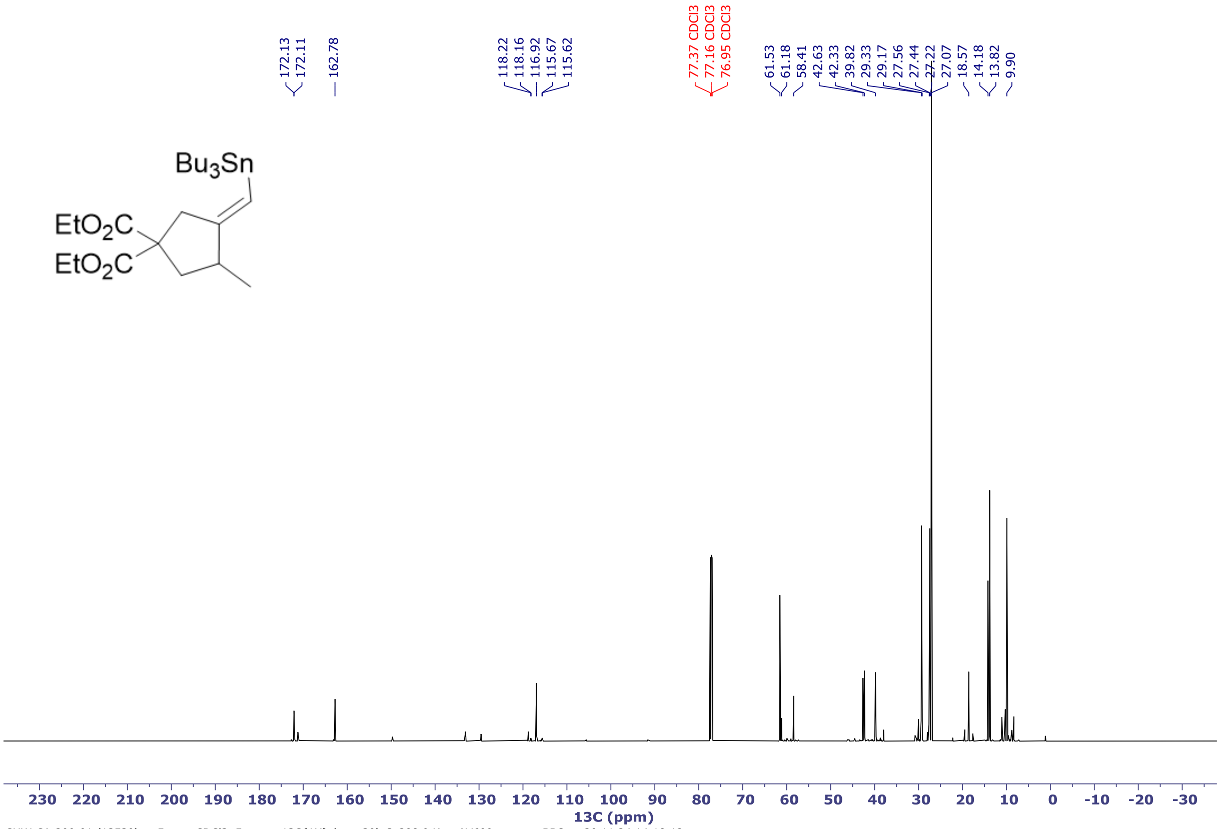

CHW-CA-200-01 (13530) — 5 mm, CDCl3, 5 mg — 13C{1H} (zgpg30) @ 298.0 K — AV600neo, cryoBBO — 20.11.24 14:18:12

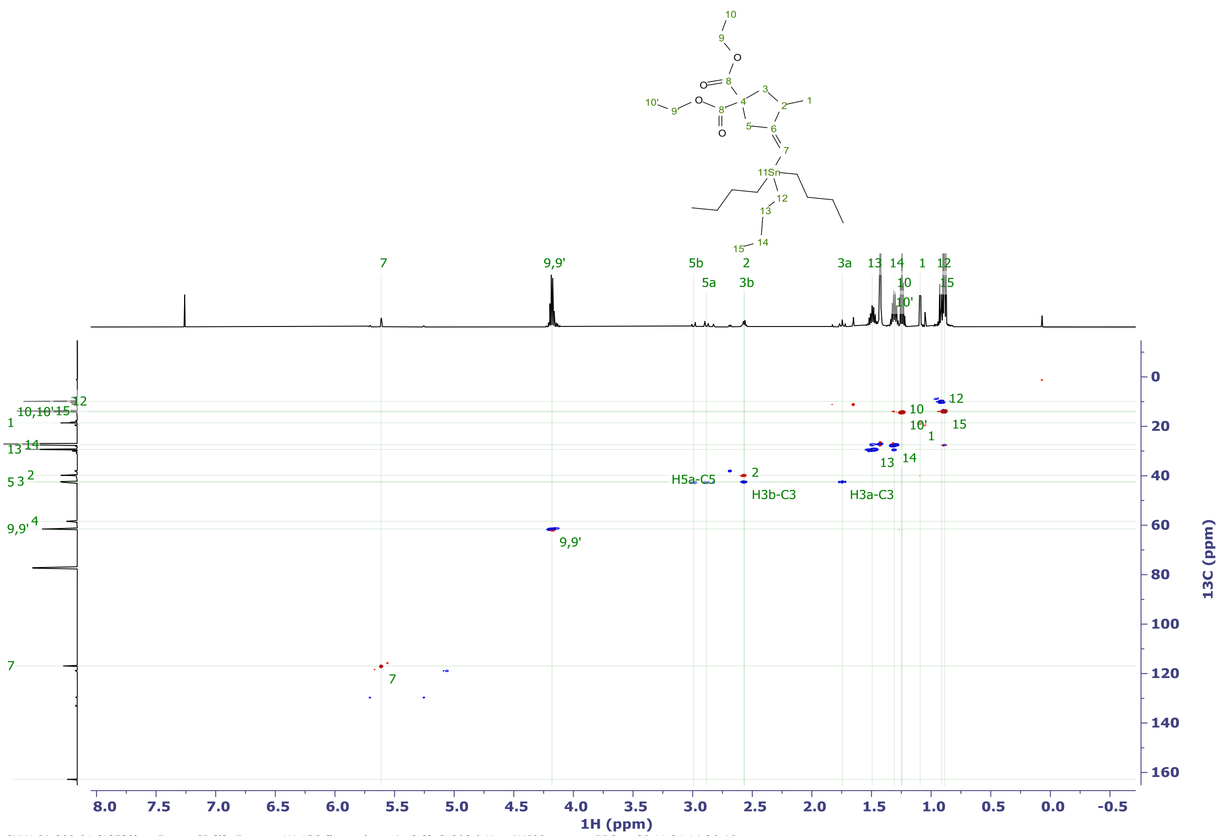

CHW-CA-200-01 (13530) — 5 mm, CDCl3, 5 mg — 1H-13C (hsqcedetgpsisp2.3) @ 298.0 K — AV600neo, cryoBBO — 20.11.24 14:26:46

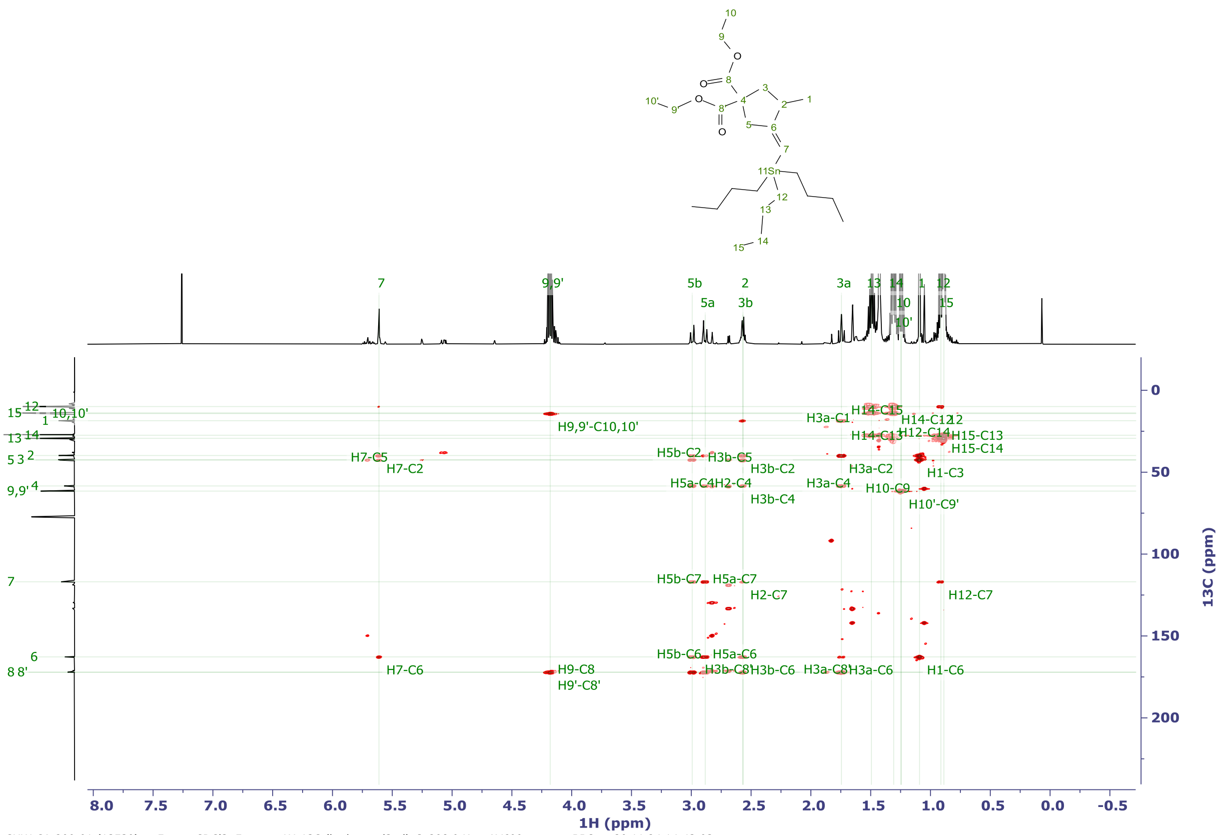

CHW-CA-200-01 (13530) — 5 mm, CDCl3, 5 mg — 1H-13C (hmbcetgpl3nd) @ 298.0 K — AV600neo, cryoBBO — 20.11.24 14:43:03

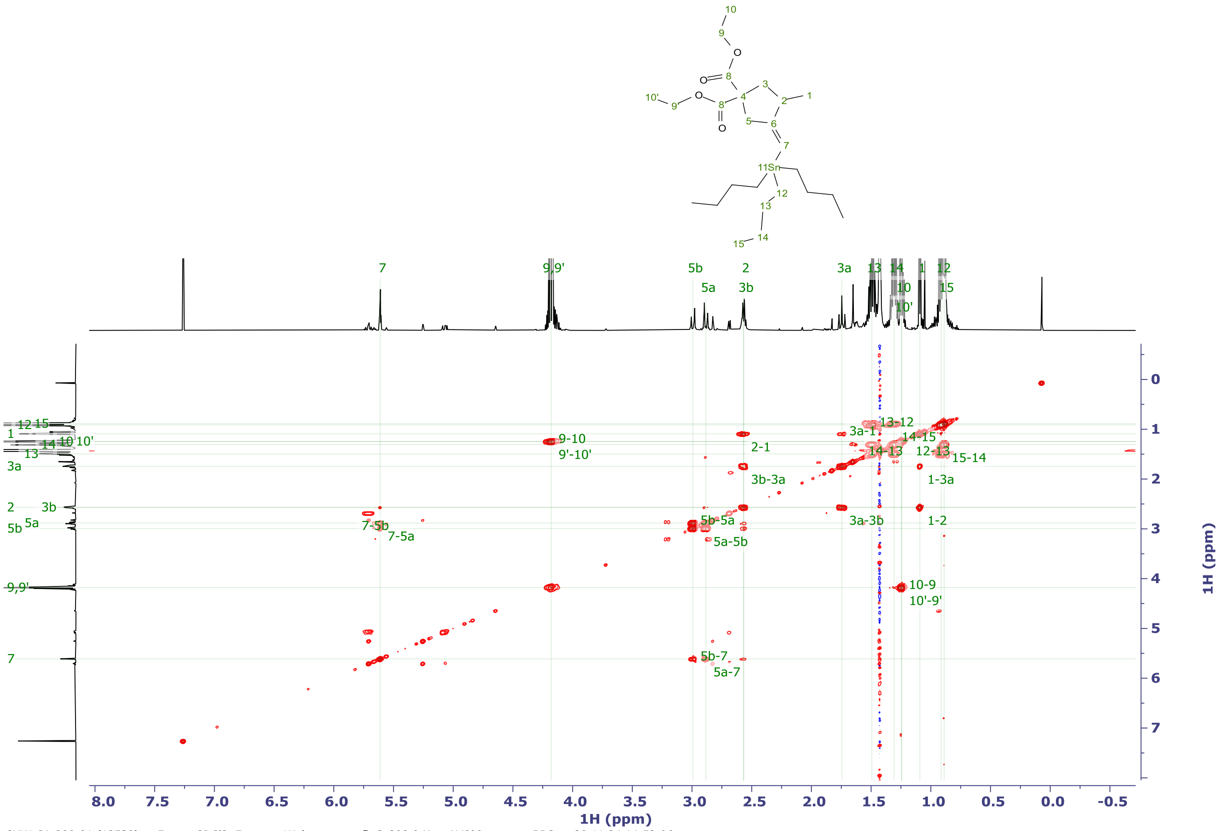

CHW-CA-200-01 (13530) — 5 mm, CDCl3, 5 mg — 1H (cosygpppqf) @ 298.0 K — AV600neo, cryoBBO — 20.11.24 14:52:06

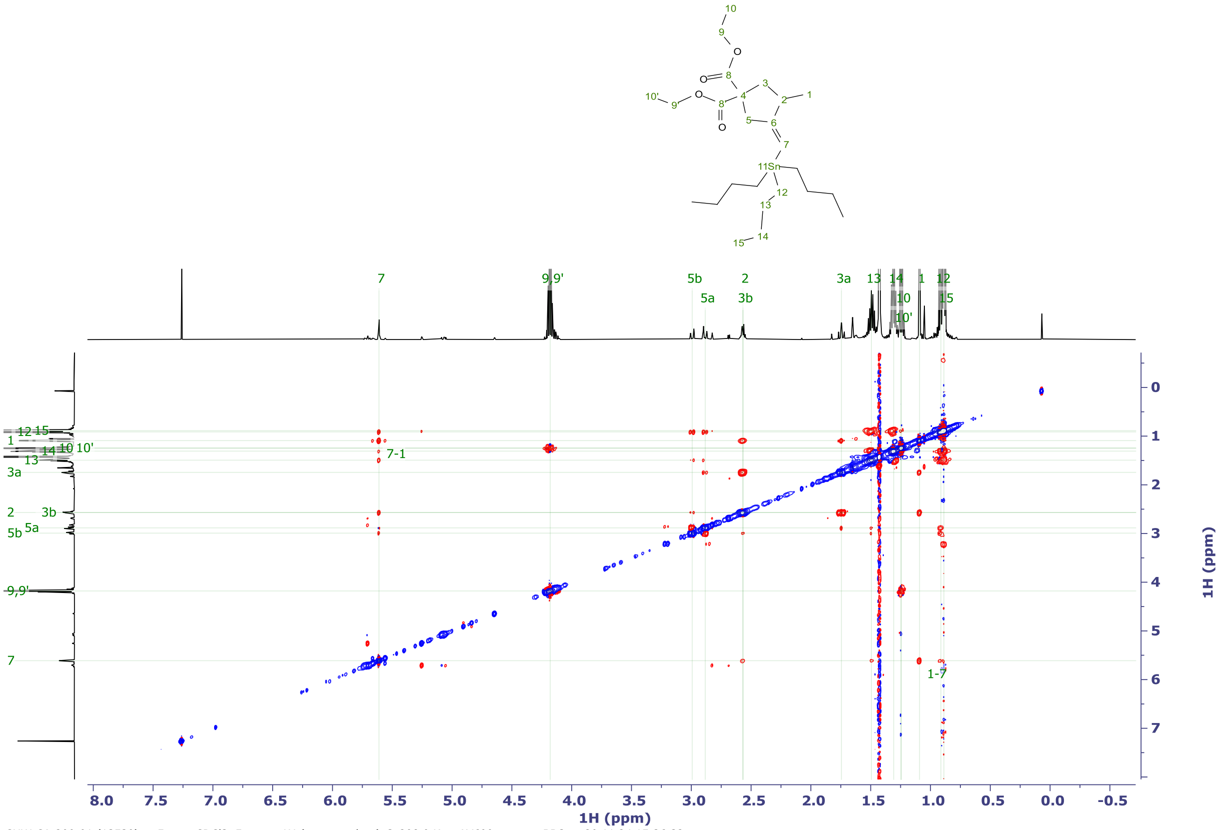

CHW-CA-200-01 (13530) — 5 mm, CDCl3, 5 mg — 1H (noesygpphpp) @ 298.0 K — AV600neo, cryoBBO — 20.11.24 15:36:32

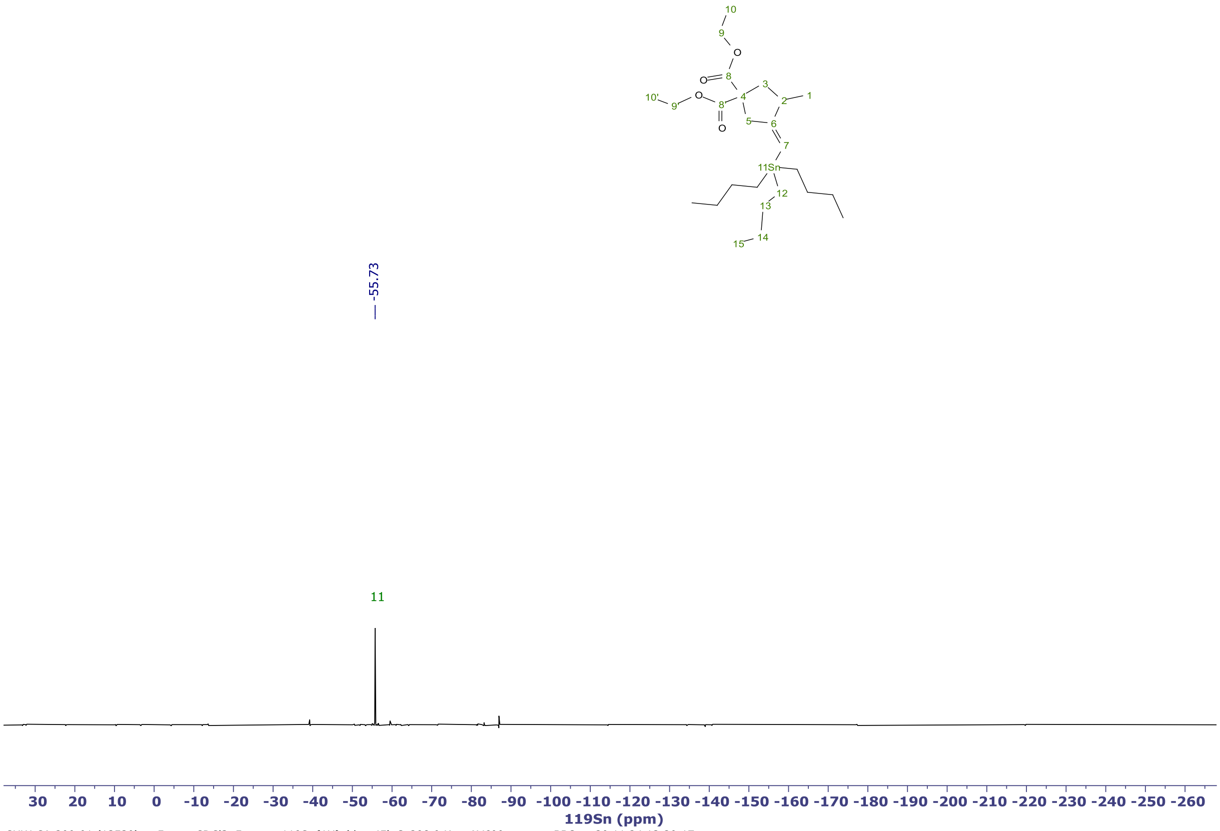

CHW-CA-200-01 (13530) — 5 mm, CDCl3, 5 mg — 119Sn{1H} (dept45) @ 298.0 K — AV600neo, cryoBBO — 20.11.24 13:20:17

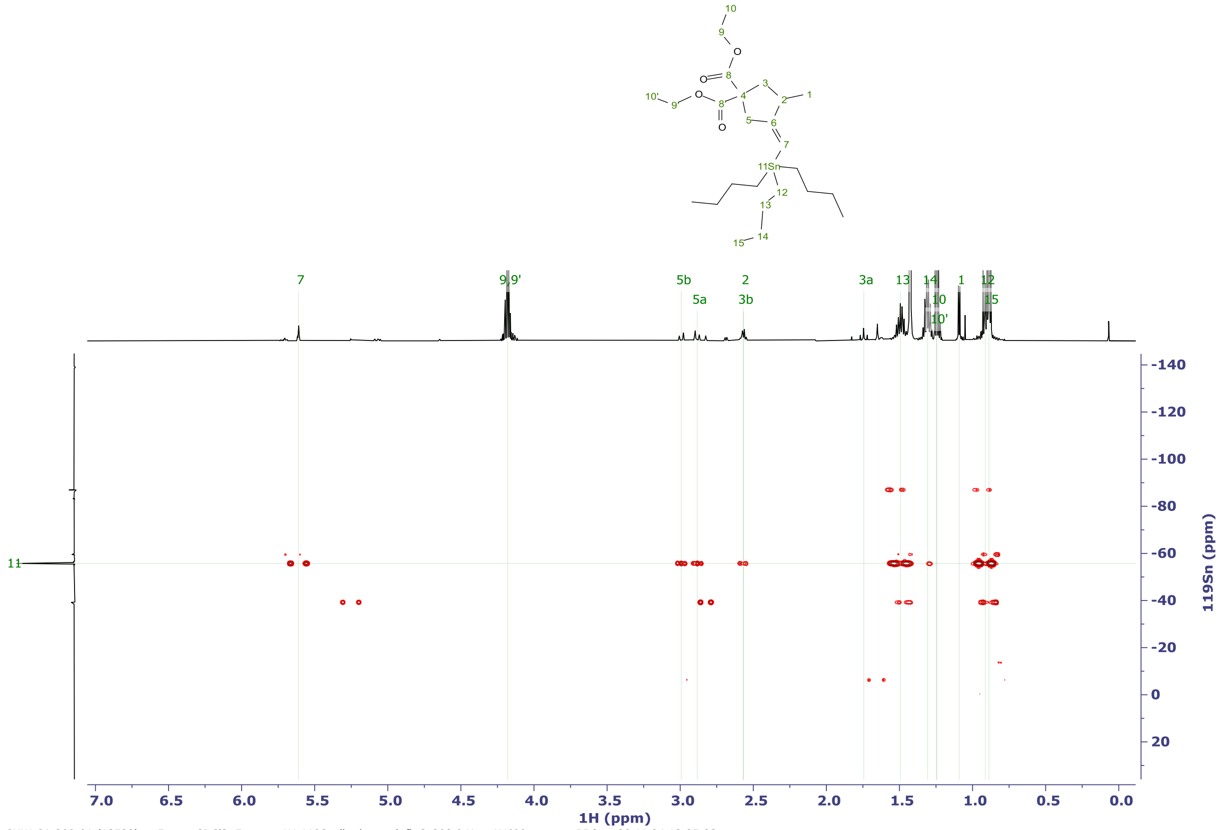

CHW-CA-200-01 (13530) — 5 mm, CDCl3, 5 mg — 1H-119Sn (hmbcgpndqf) @ 298.0 K — AV600neo, cryoBBO — 20.11.24 13:25:28

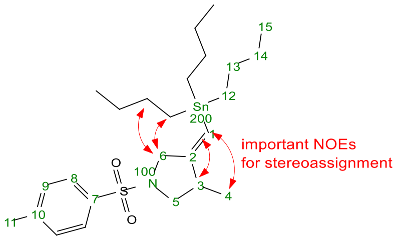

Atom	δ (ppm)	J	COSY	HSQC	HMBC	NOESY
1 C	118.192			1	6', 6''	
H	5.665	2.40(6'')	6', 6''	1	2, 3, 200	3, 4, 12, 13, 14, 15
2 C	158.224				1, 4, 6', 6''	
3 C	40.131			3	1, 4, 5', 5'', 6', 6''	
H	2.657	6.40(4)	4, 5''	3	4, 5, 200	1, 4
4 C	16.366			4	3, 5', 5''	
H3	1.023	6.40(3)	3, 5''	4	2, 3, 5	1, 3, 5', 5''
5 C	55.356			5', 5''	3, 4, 6''	
H'	2.657		5''	5	3, 4	4, 8
H''	3.596		3, 4, 5'	5	3, 4, 6, 100	4, 8
6 C	53.606			6', 6''	5''	
H'	3.596		1, 6''	6	1, 2, 3, 200	8, 12, 13
H''	3.867	2.40(1)	1, 6'	6	1, 2, 3, 5, 100, 200	8, 12, 13
7 C	132.784				9	
8 C	127.928			8	8	
H	7.693		9	8	8, 10	5', 5'', 6', 6''

Atom	δ (ppm)	J	COSY	HSQC	HMBC	NOESY
9 C	129.749			9	9, 11	
H	7.317		8, 11	9	7, 9, 11	11
10 C	143.654				8, 11	
11 C	21.647			11	9	
H3	2.424		9	11	9, 10	9
12 C	9.829			12	13, 14	
H2	0.871		13	12	13, 14, 200	1, 6', 6''
13 C	29.231			13	12, 14, 15	
H2	1.427		12, 14	13	12, 14, 15, 200	1, 6', 6''
14 C	27.367			14	12, 13, 15	
H2	1.275		13, 15	14	12, 13, 15	1
15 C	13.780			15	13, 14	
H3	0.871		14	15	13, 14	1
100 N	-283.209				5'', 6''	
200 Sn	-53.674				1, 3, 6', 6'', 12, 13	

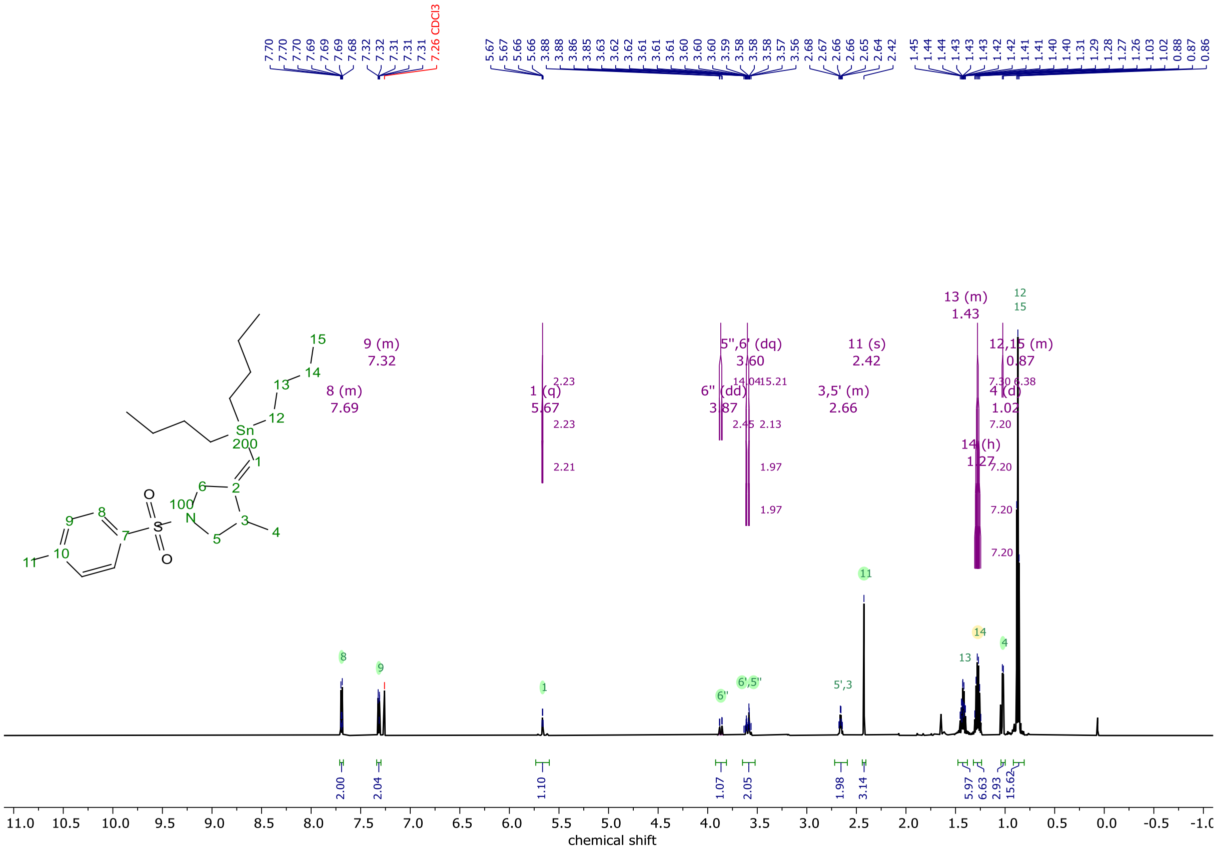

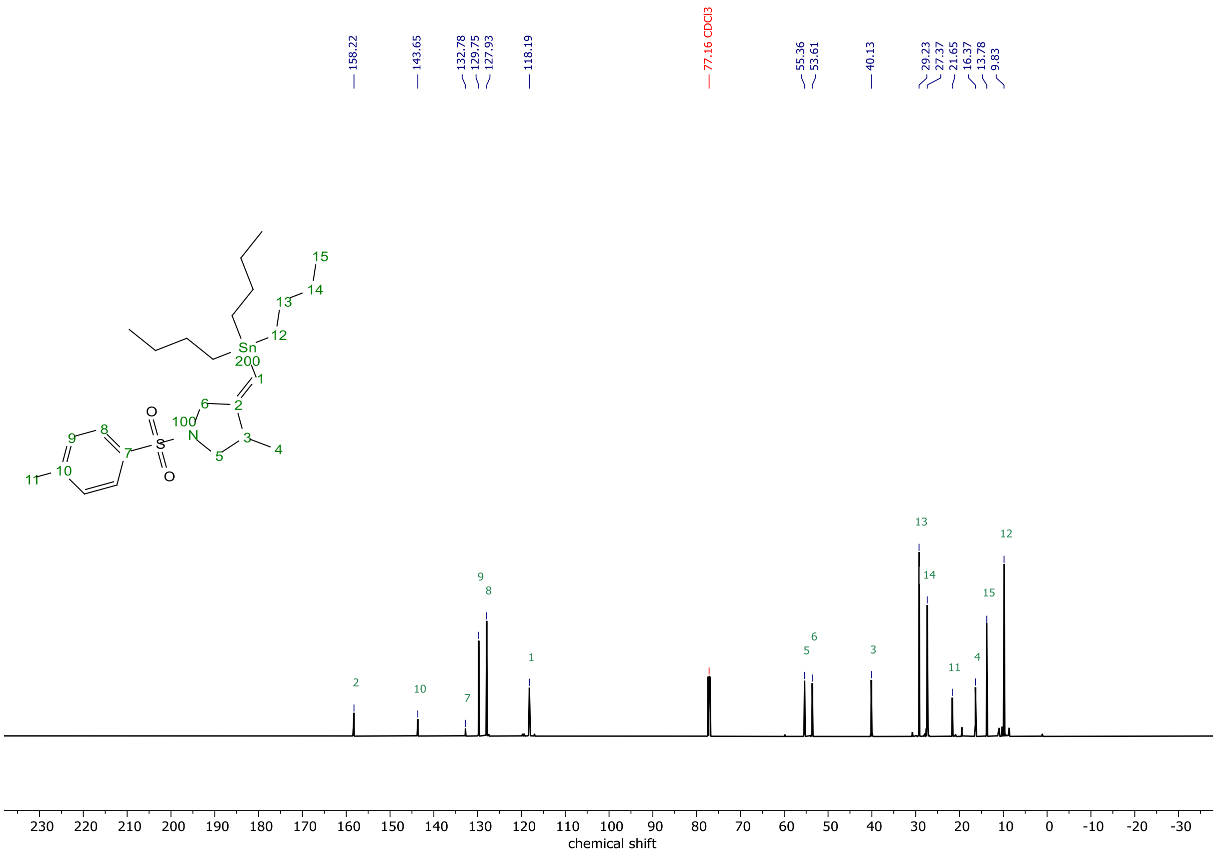

1H{13C},HSQC-EDITED, 600.20 MHz,CDCl3,298.0K, pulse sequence: hsqcedetgpsisp2.3

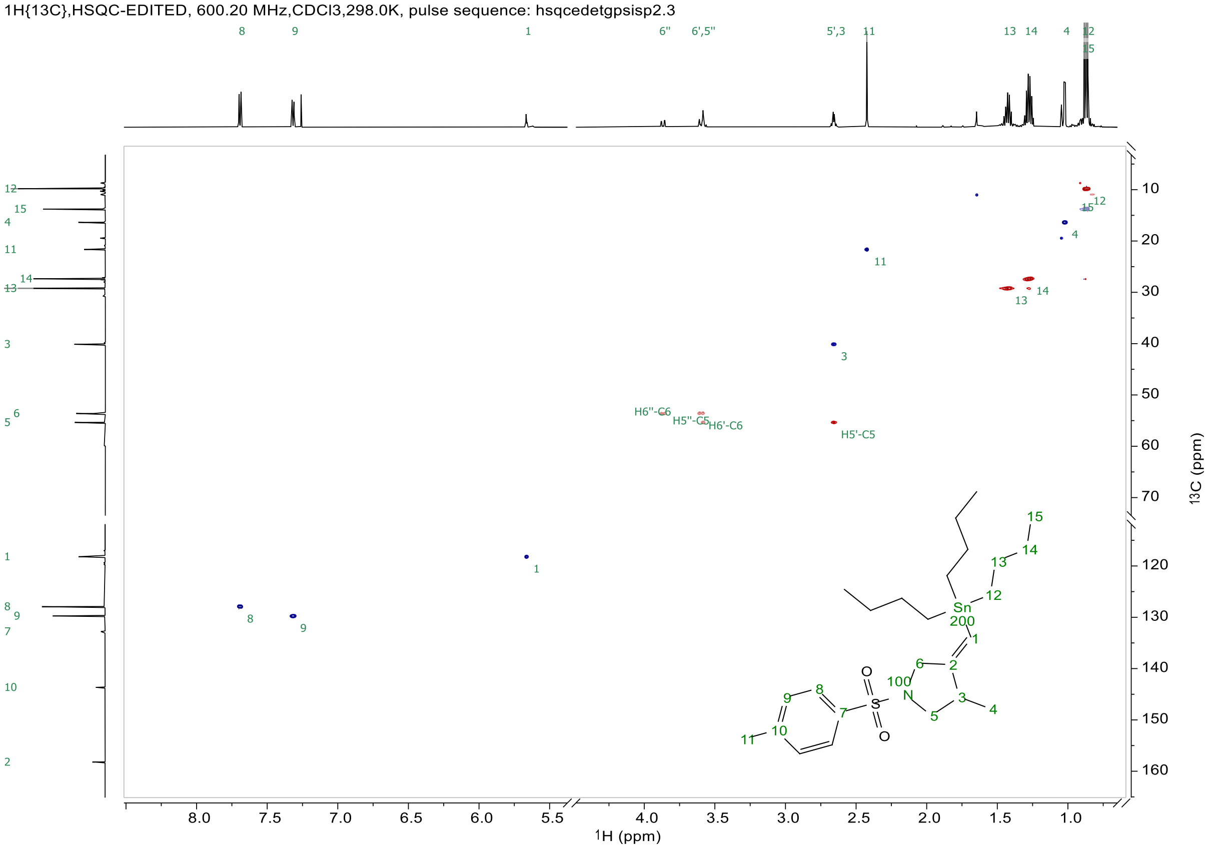

1H{13C},HMBC, 600.20 MHz,CDCl3,298.0K, pulse sequence: hmbcetgpl3nd

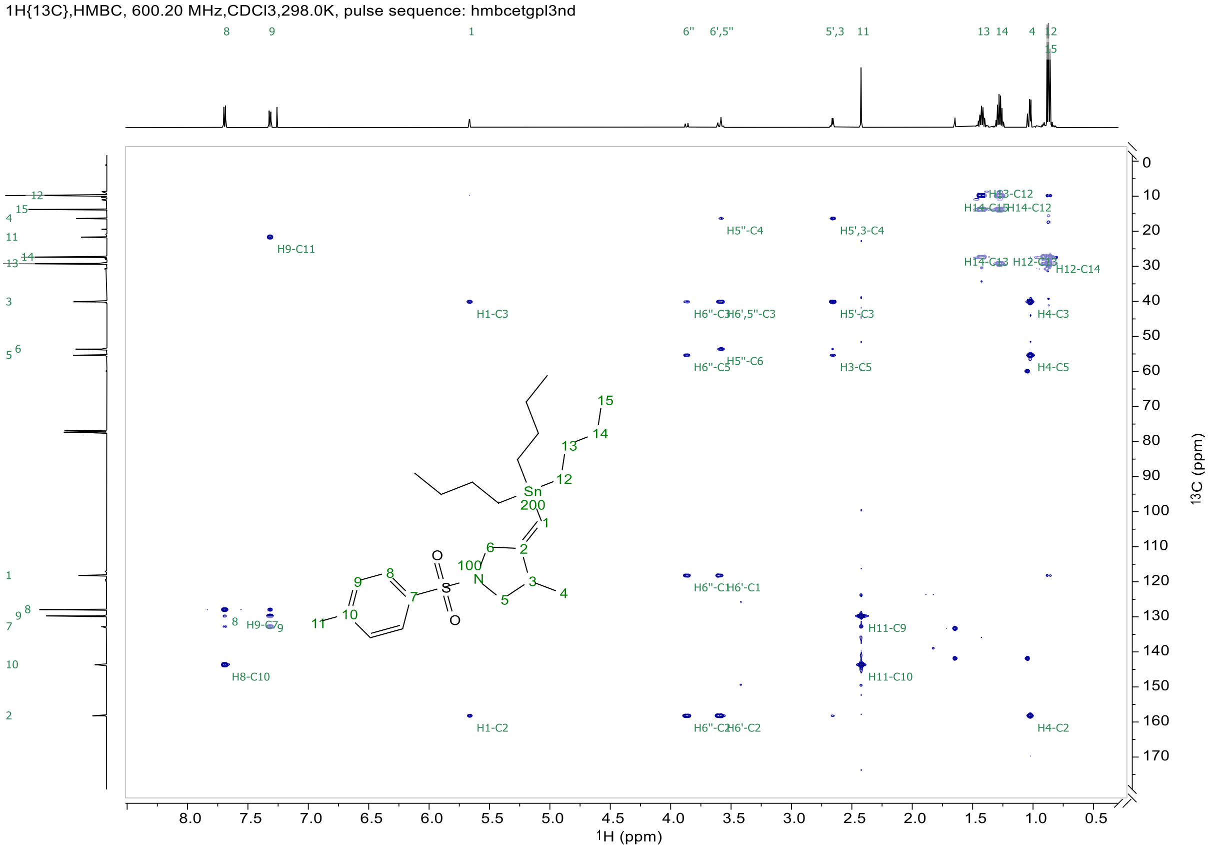

1H{off},COSY, 600.20 MHz,CDCl3,298.0K, pulse sequence: cosygpppqf

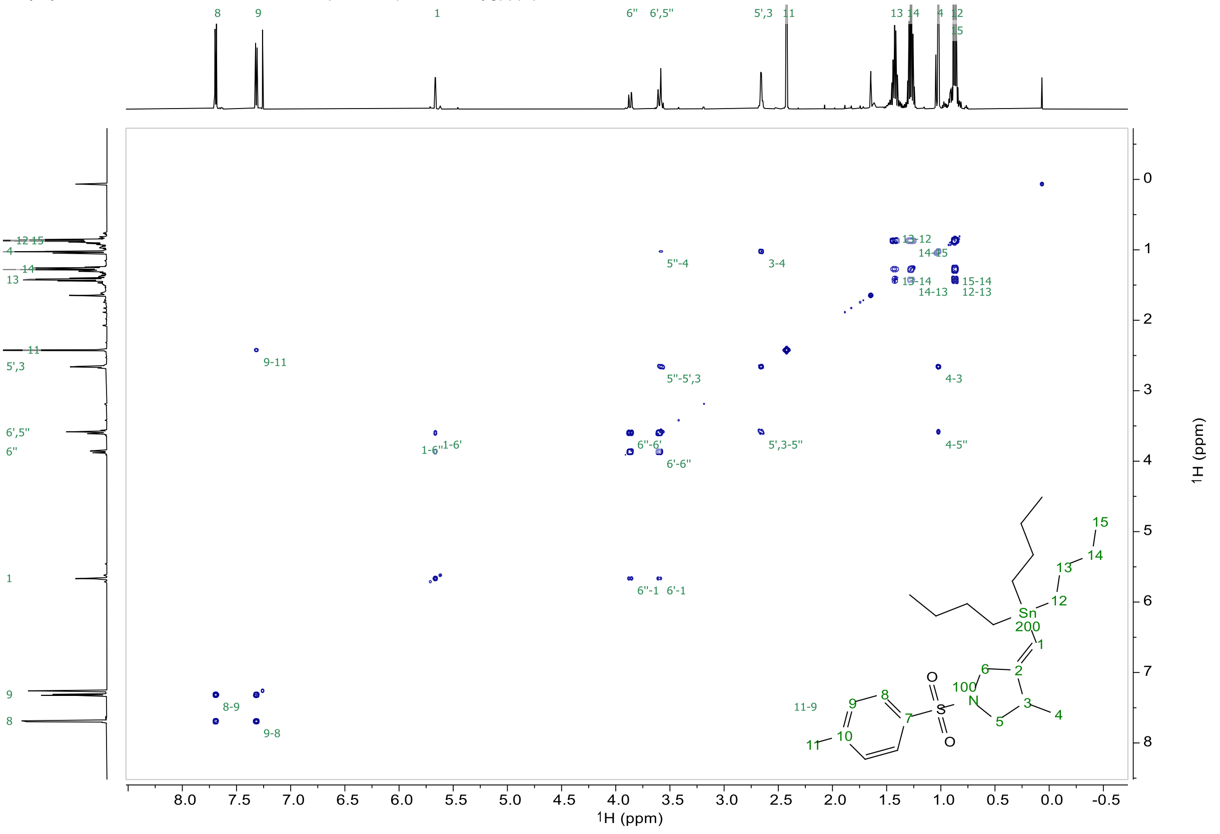

1H{off},NOESY, 600.20 MHz,CDCl3,298.0K, pulse sequence: noesygpphpp

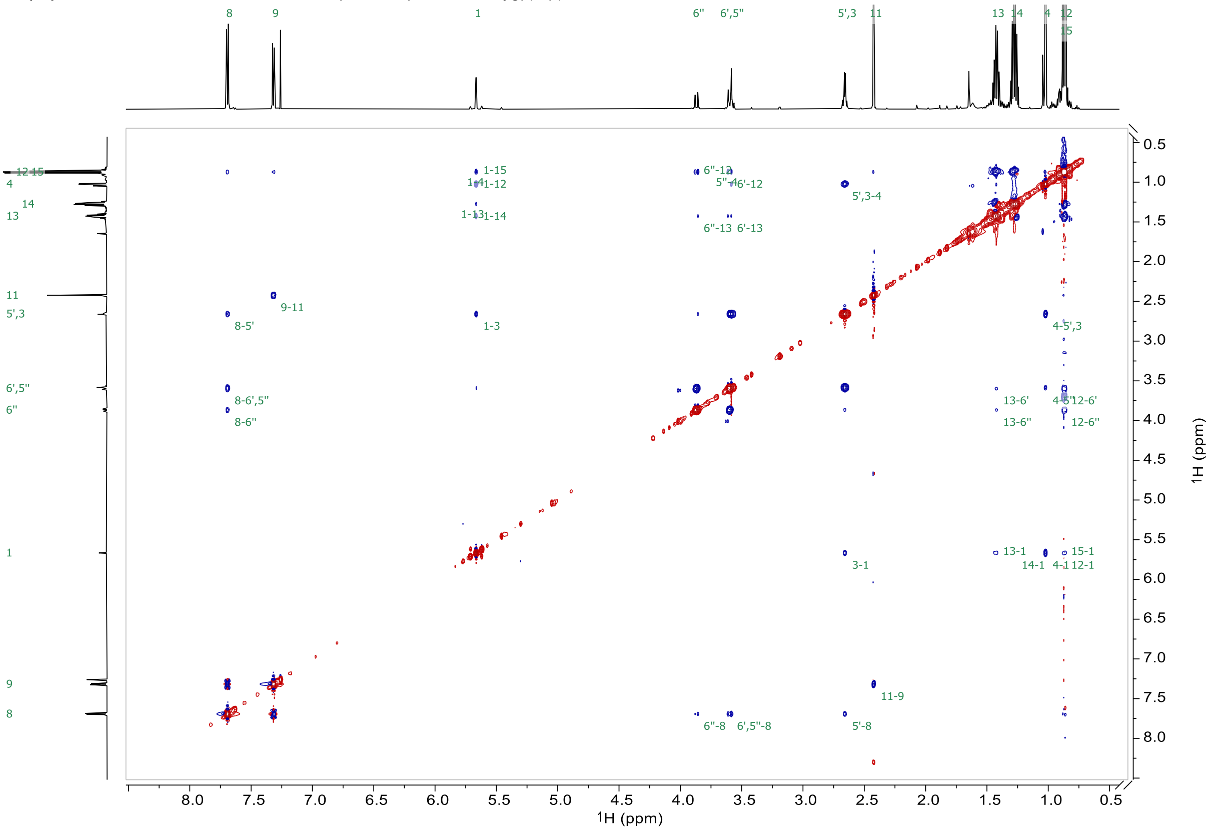

119Sn{1H},DEPT-45, 223.79 MHz,CDCl3,298.0K, pulse sequence: dept45

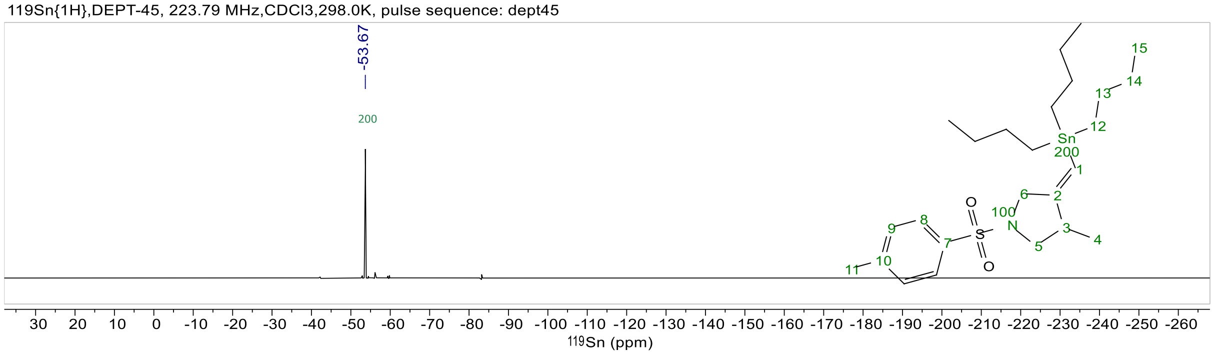

1H{119Sn},HMBC, 600.20 MHz,CDCl3,298.0K, pulse sequence: hmbcgpndqf

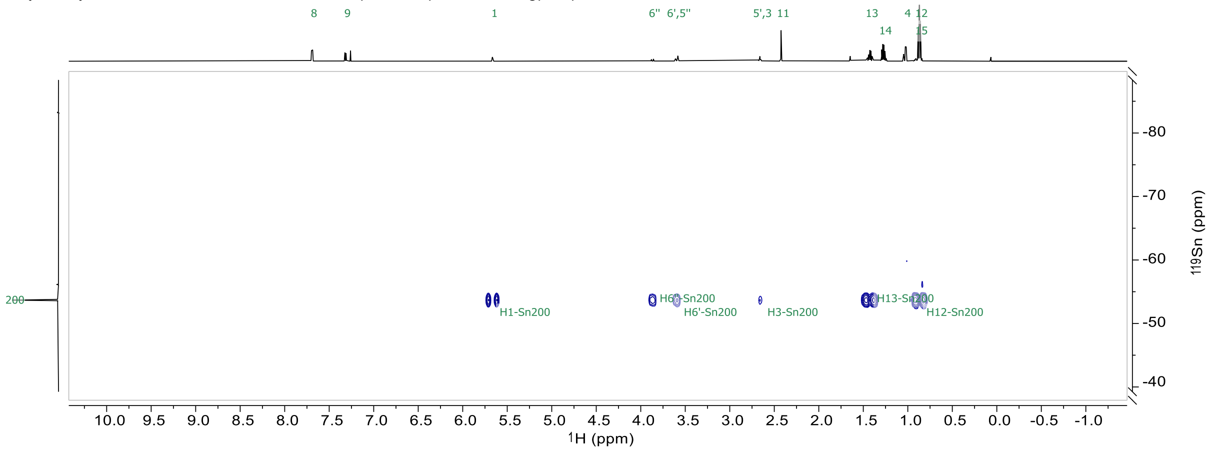

1H{15N},HMBC, 600.20 MHz,CDCl3,298.0K, pulse sequence: hmbcgpndqf

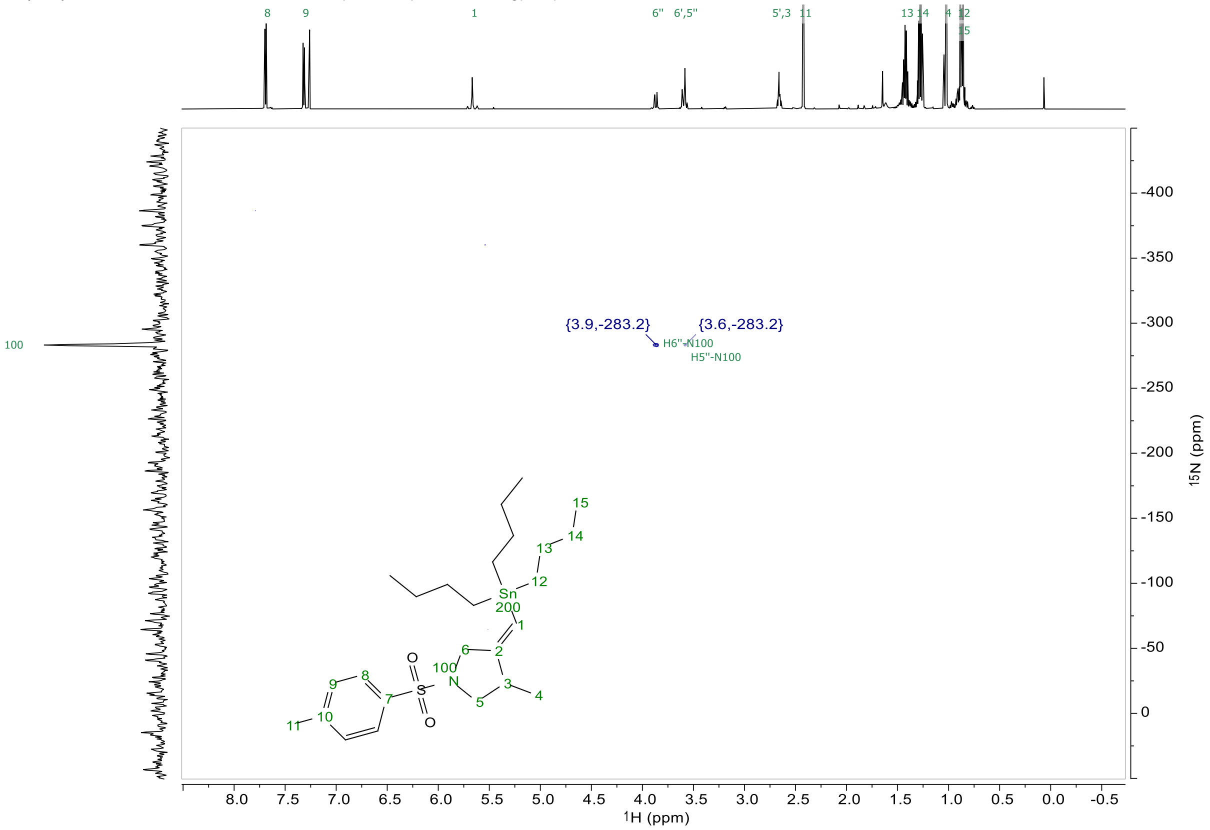

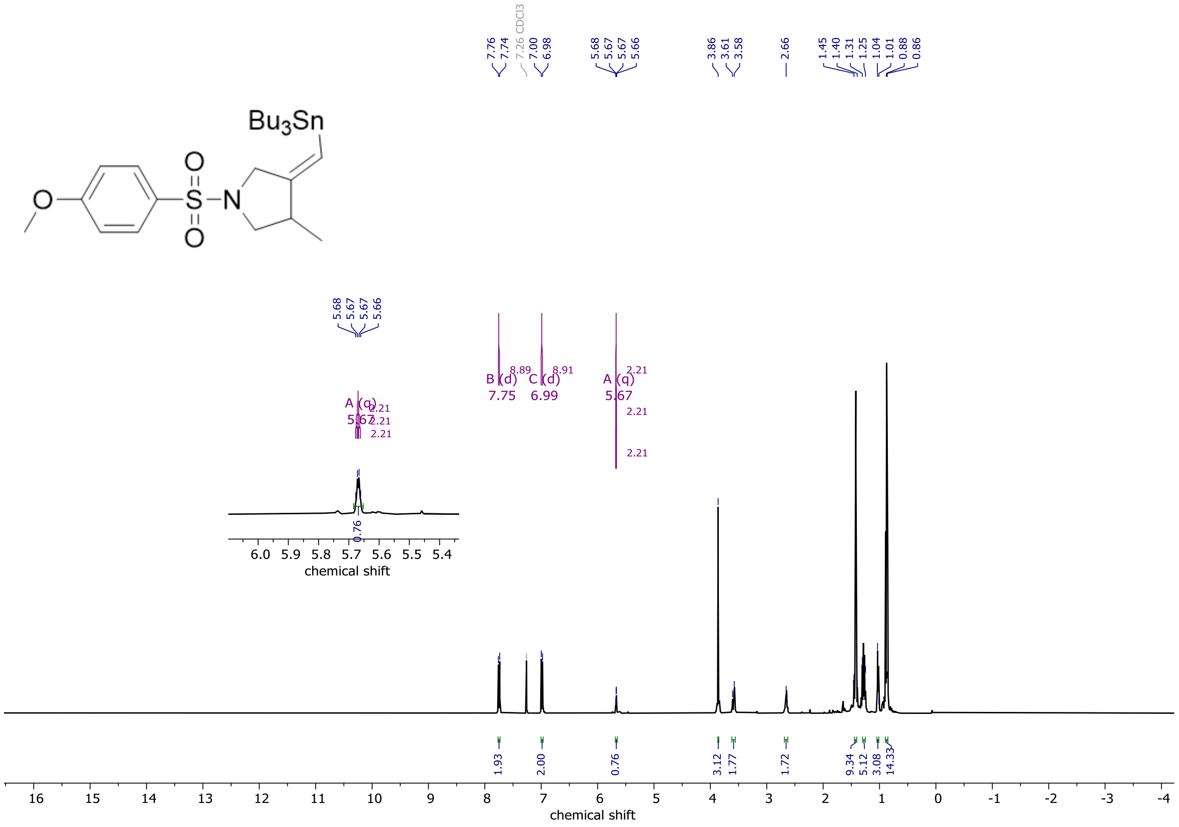
7.76
7.74
7.26 CDCl3
7.00
6.98
5.68
5.67
5.67
5.66
3.86
3.61
3.58
2.66
1.45
1.40
1.31
1.25
1.04
1.01
0.88
0.86
Bu3Sn
O
S
N
O
O
B (d)
7.75
C (d)
6.99
A (q)
5.67
1.93
2.00
0.76
3.12
1.77
1.72
9.34
5.12
3.08
14.33
chemical shift

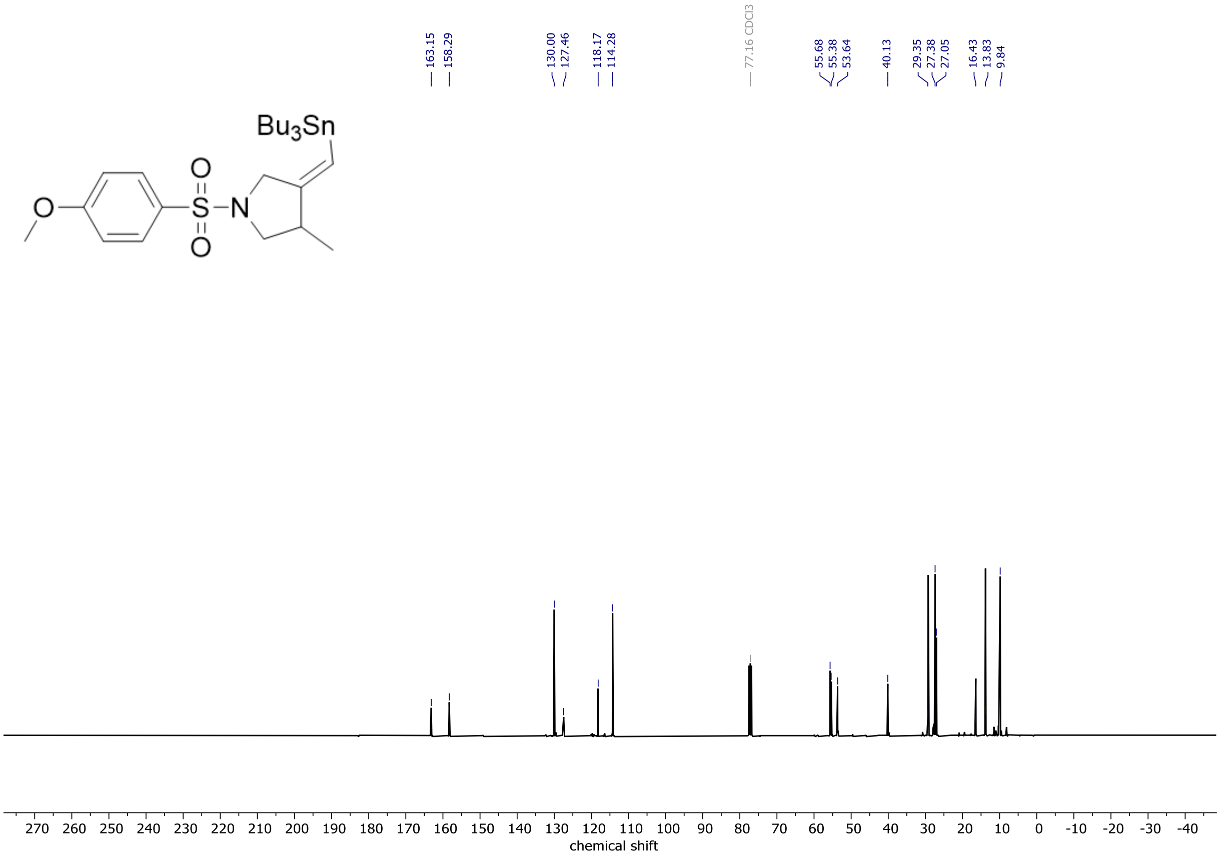
163.15
158.29
130.00
127.46
118.17
114.28
77.16 CDCl3
55.68
55.38
53.64
40.13
29.35
27.38
27.05
16.43
13.83
9.84
Bu3Sn
O
S
N
O
O
chemical shift

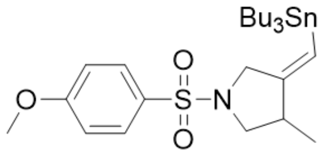

Bu3Sn
O
O
S
N
O

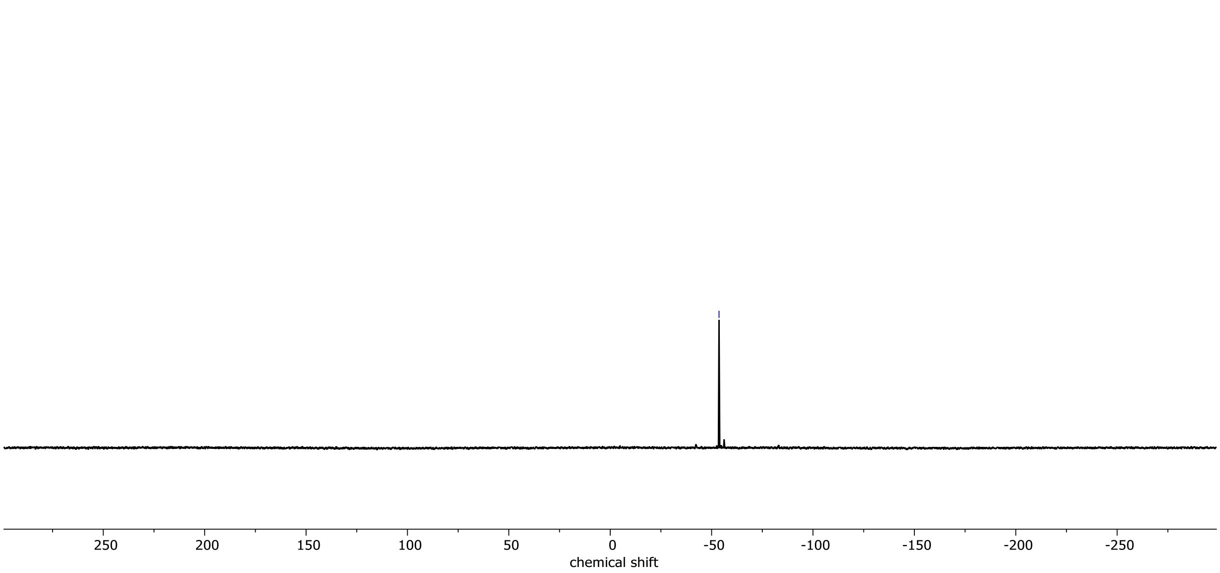

-53.66
250
200
150
100
50
0
-50
-100
-150
-200
-250
chemical shift

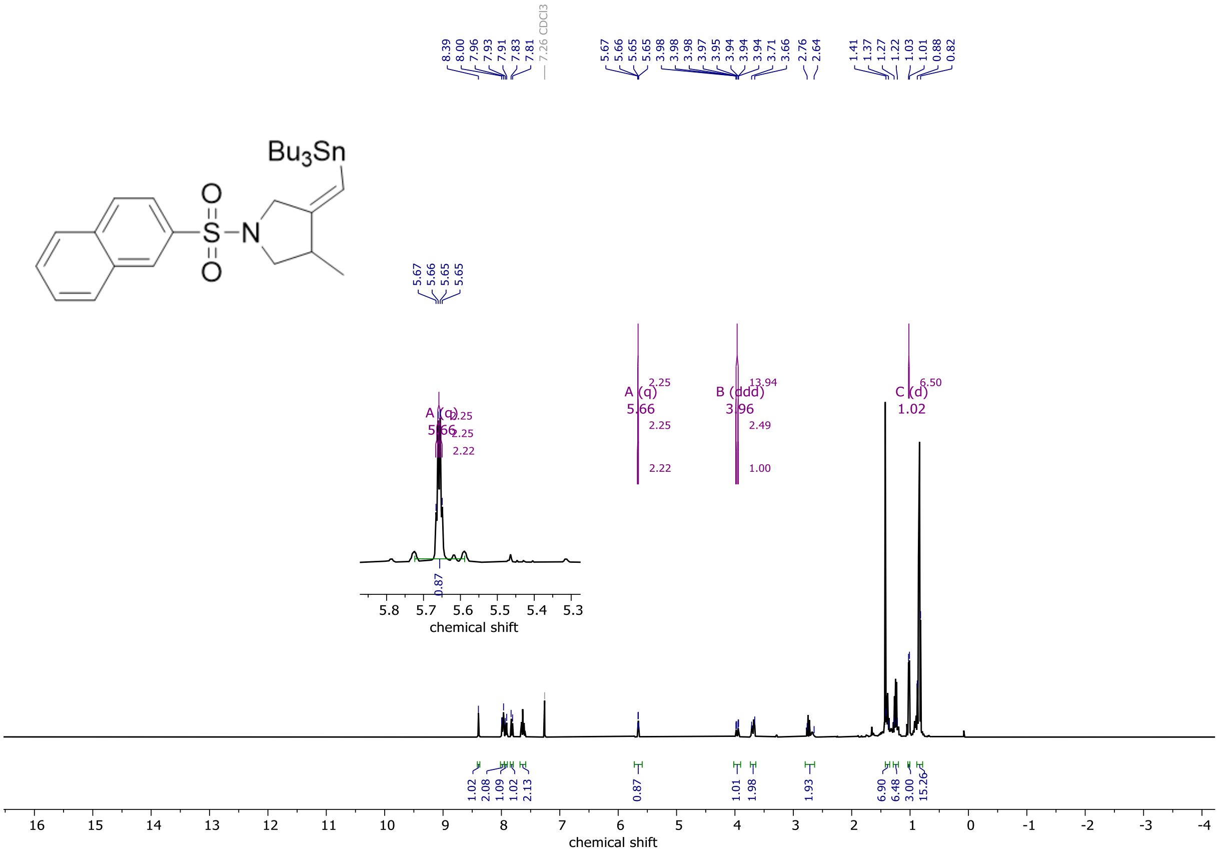

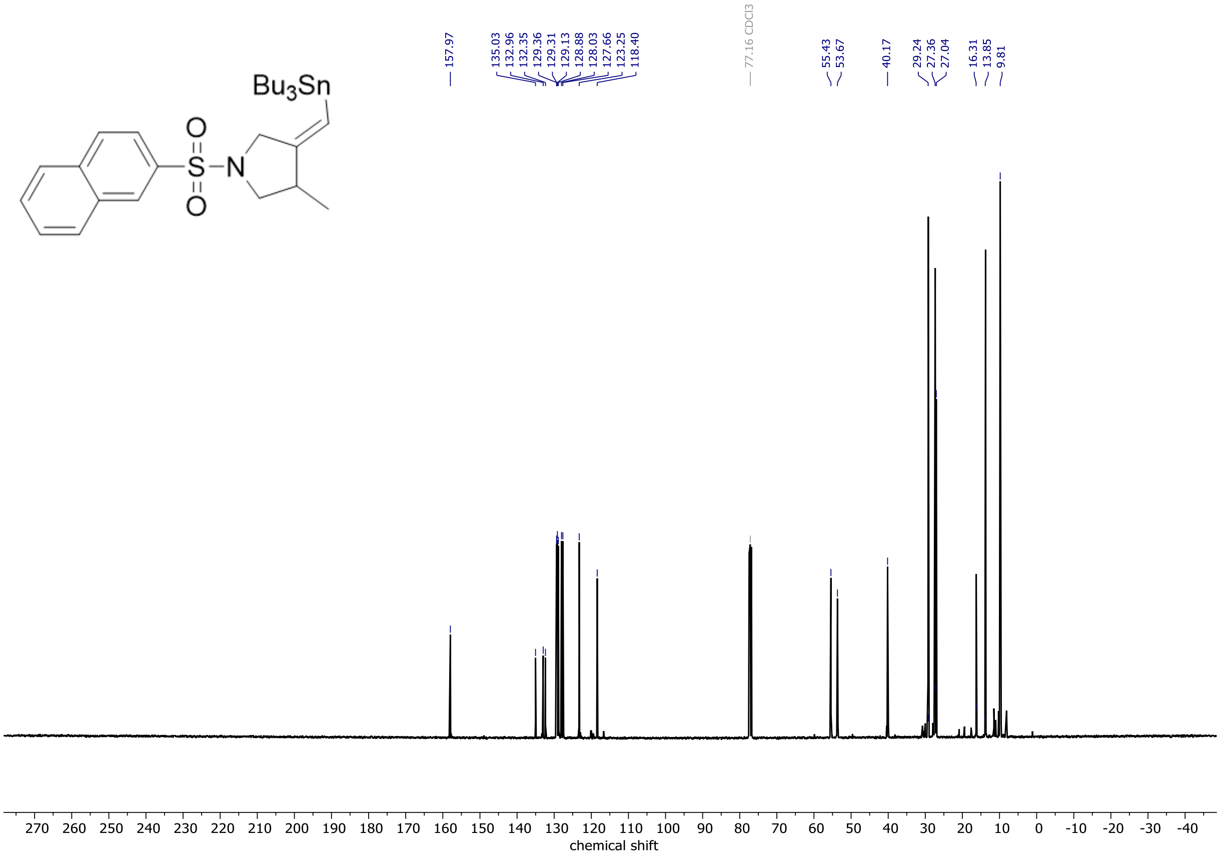

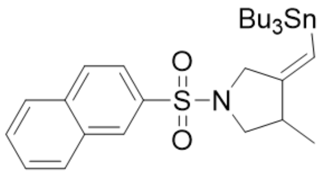
Bu3Sn
O
S
N
O

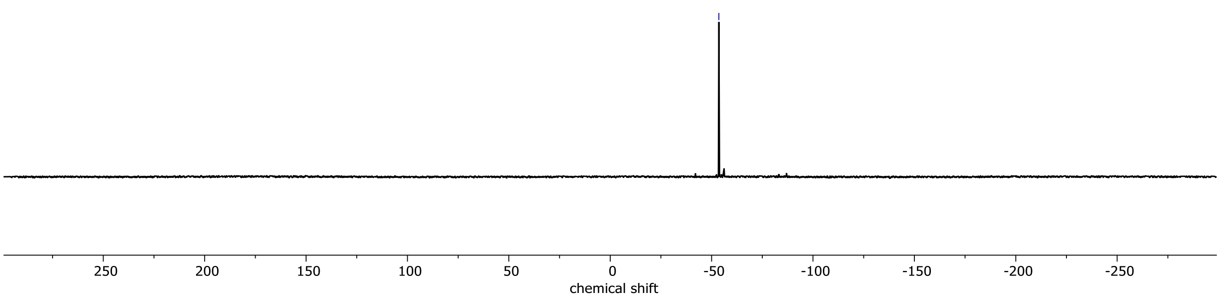
-53.59
250
200
150
100
50
0
-50
-100
-150
-200
-250
chemical shift

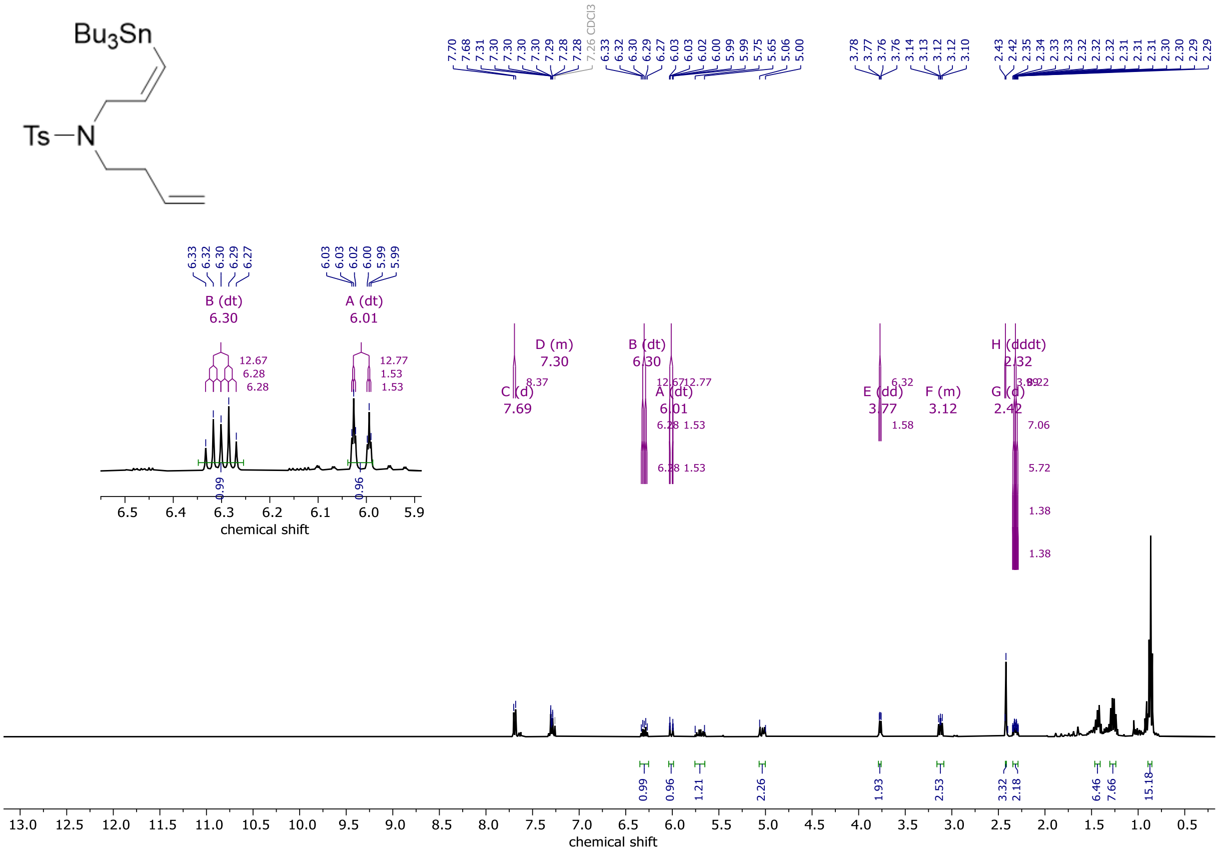

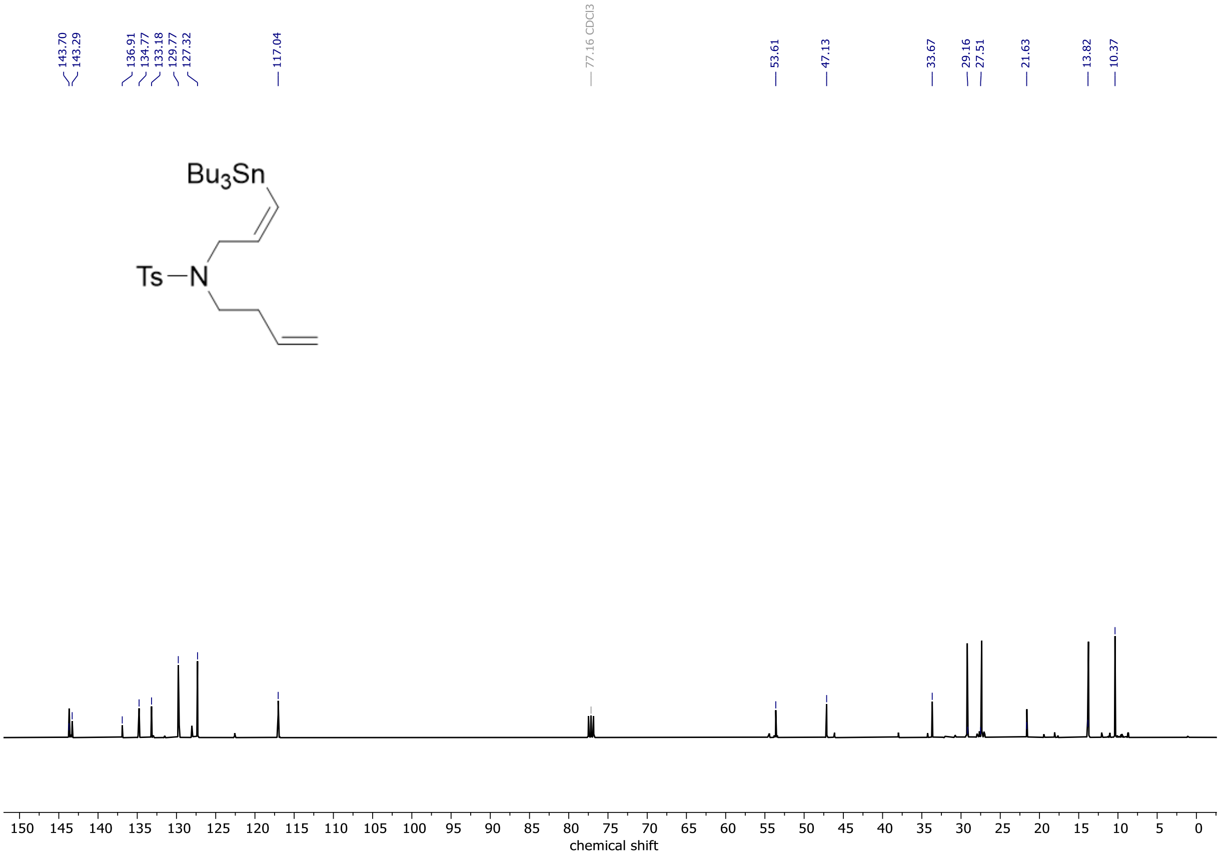

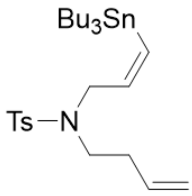
Bu3Sn
Ts
N

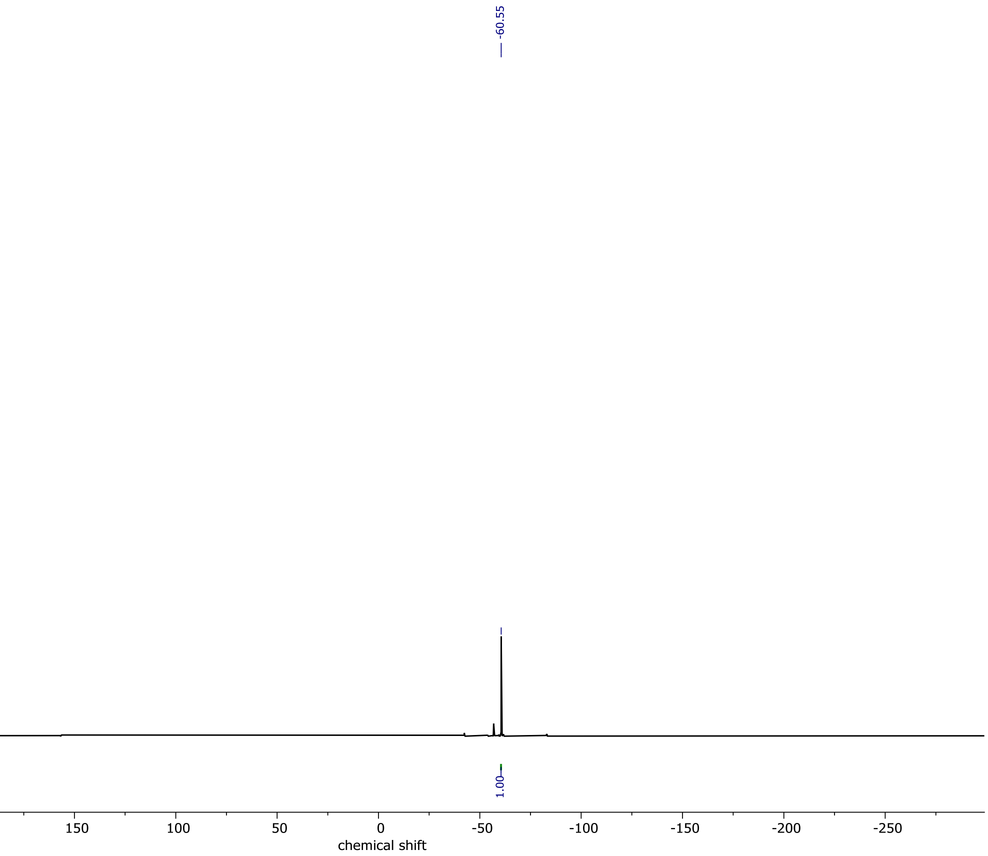
-60.55
1.00
250
200
150
100
50
0
-50
-100
-150
-200
-250
chemical shift

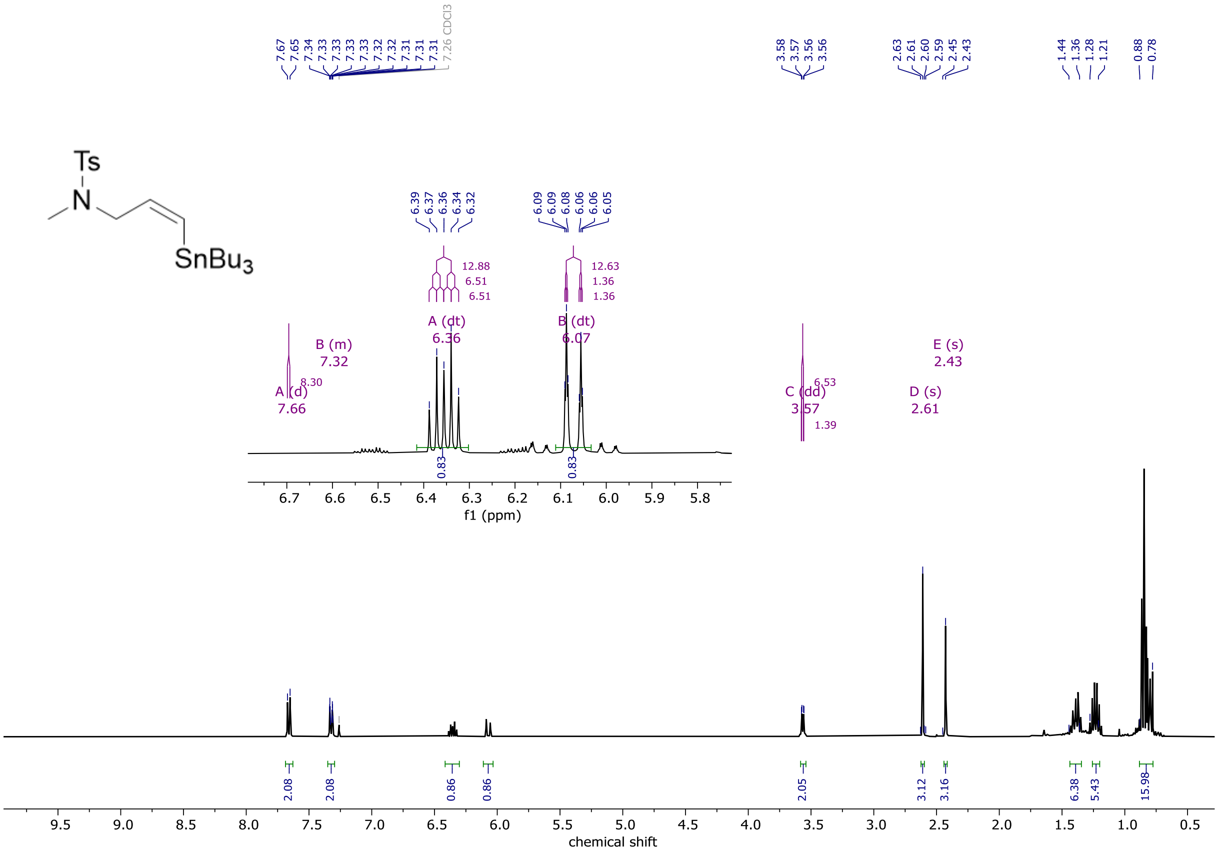

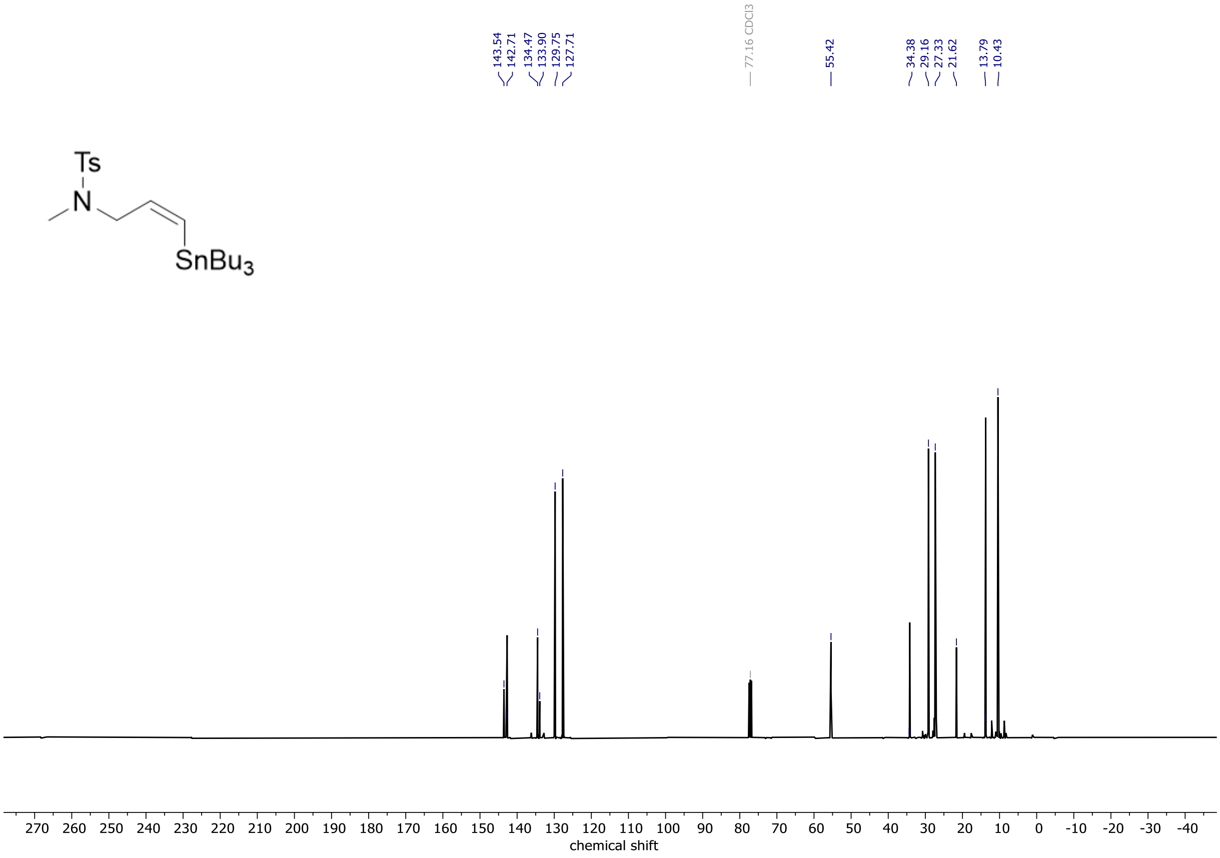

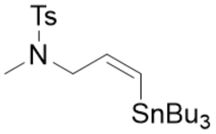
Ts
N
SnBu3

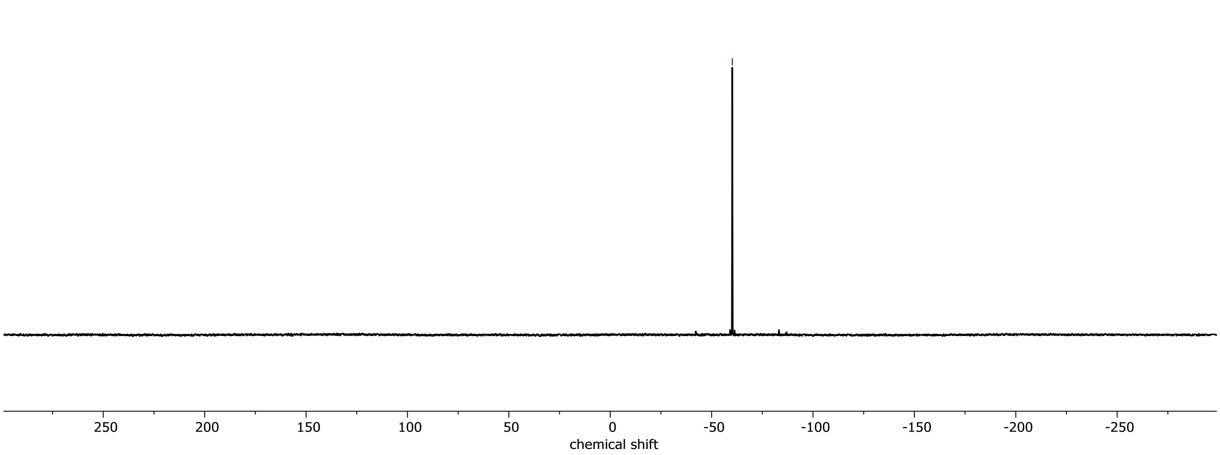
-60.19
250
200
150
100
50
0
-50
-100
-150
-200
-250
chemical shift

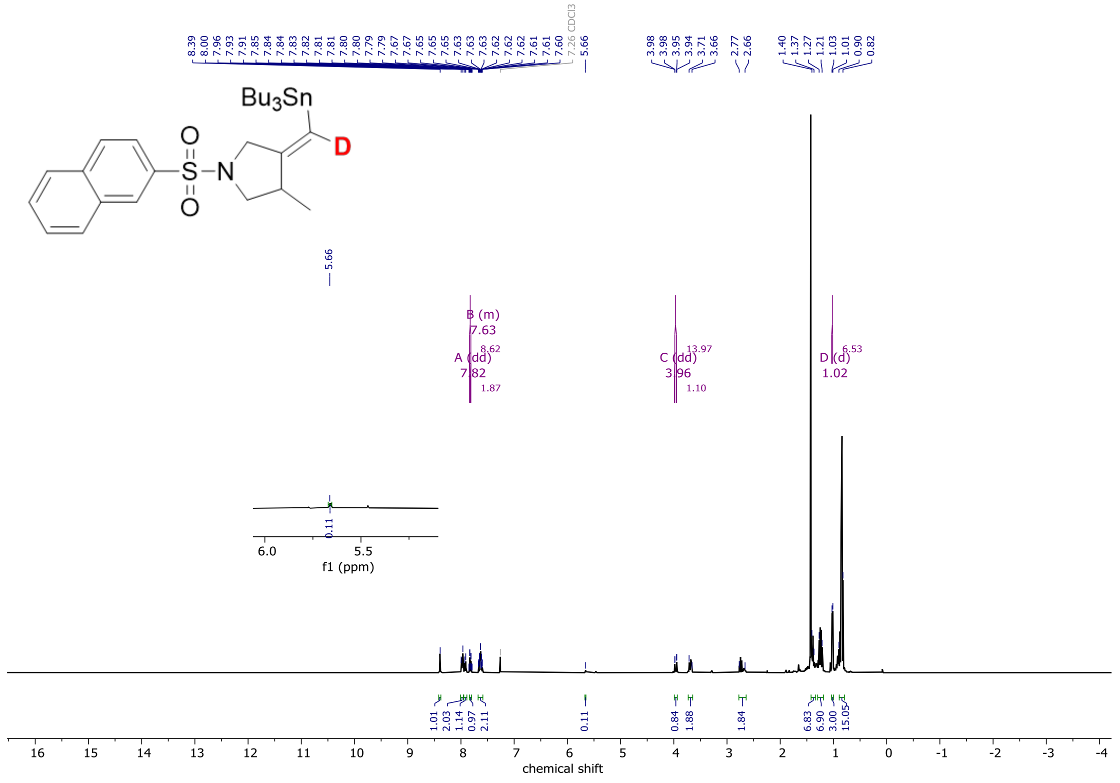

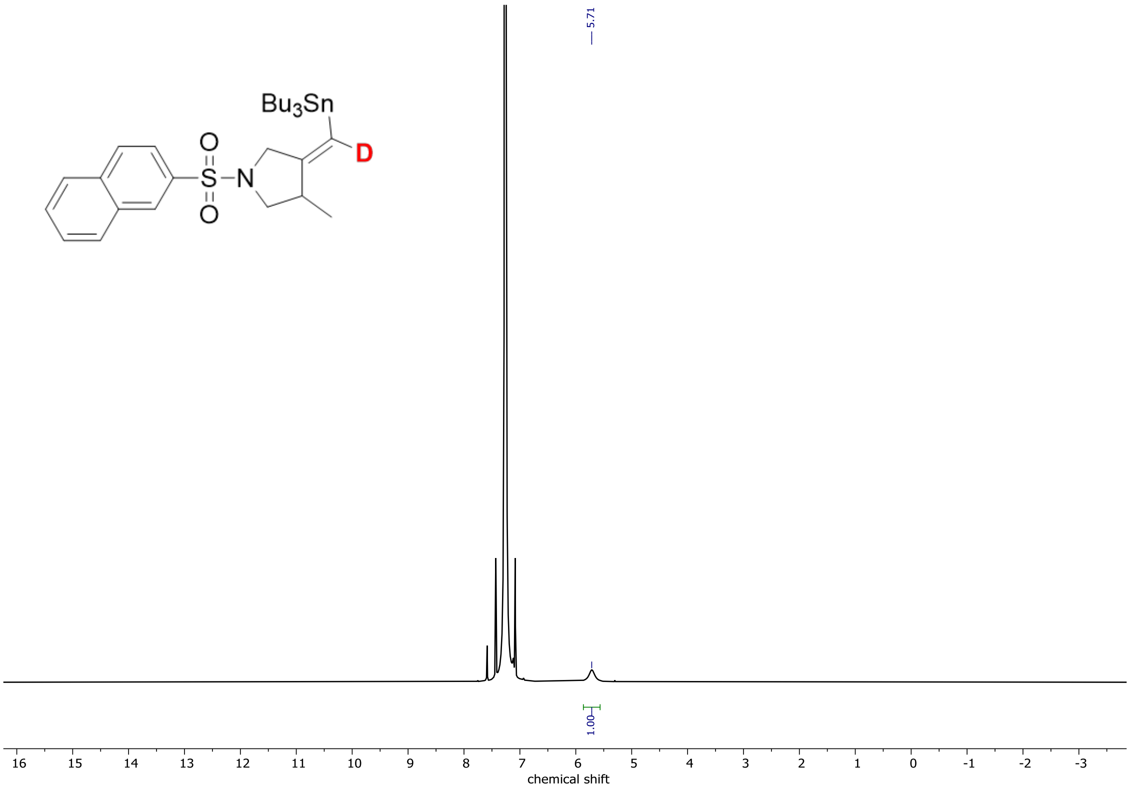

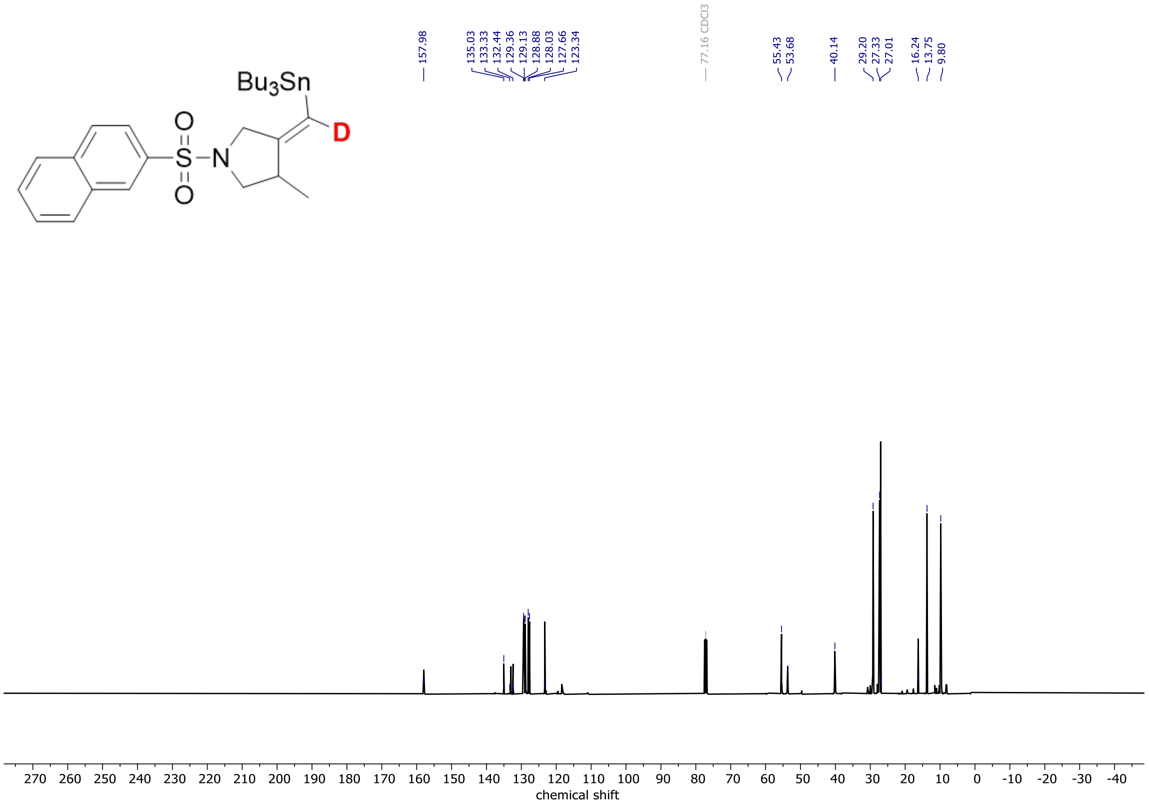
Bu3Sn
D
O
S
O
N
157.98
135.03
133.33
132.44
129.36
129.13
128.88
128.03
127.66
123.34
77.16 CDCl3
55.43
53.68
40.14
29.20
27.33
27.01
16.24
13.75
9.80
270 260 250 240 230 220 210 200 190 180 170 160 150 140 130 120 110 100 90 80 70 60 50 40 30 20 10 0 -10 -20 -30 -40
chemical shift

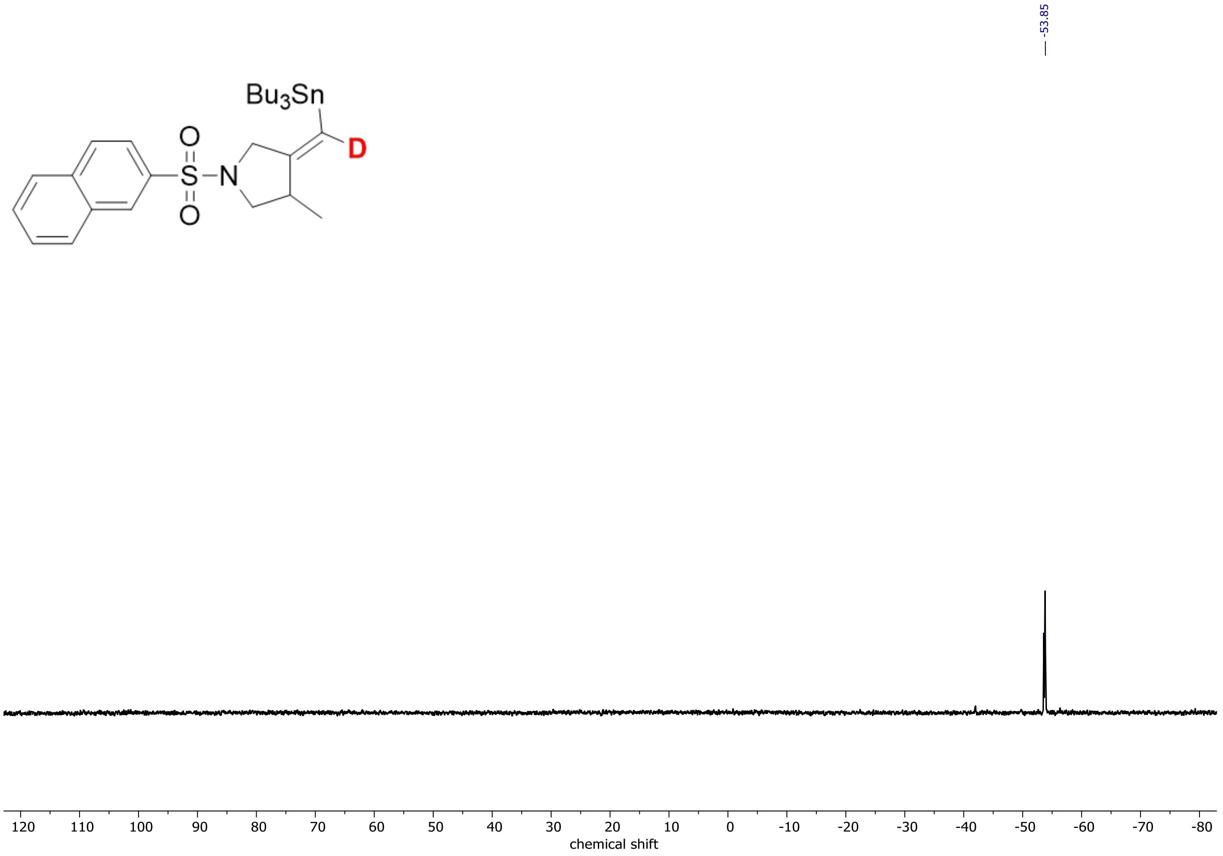
Bu3Sn
D
O
S
O
N
-53.85
120 110 100 90 80 70 60 50 40 30 20 10 0 -10 -20 -30 -40 -50 -60 -70 -80
chemical shift

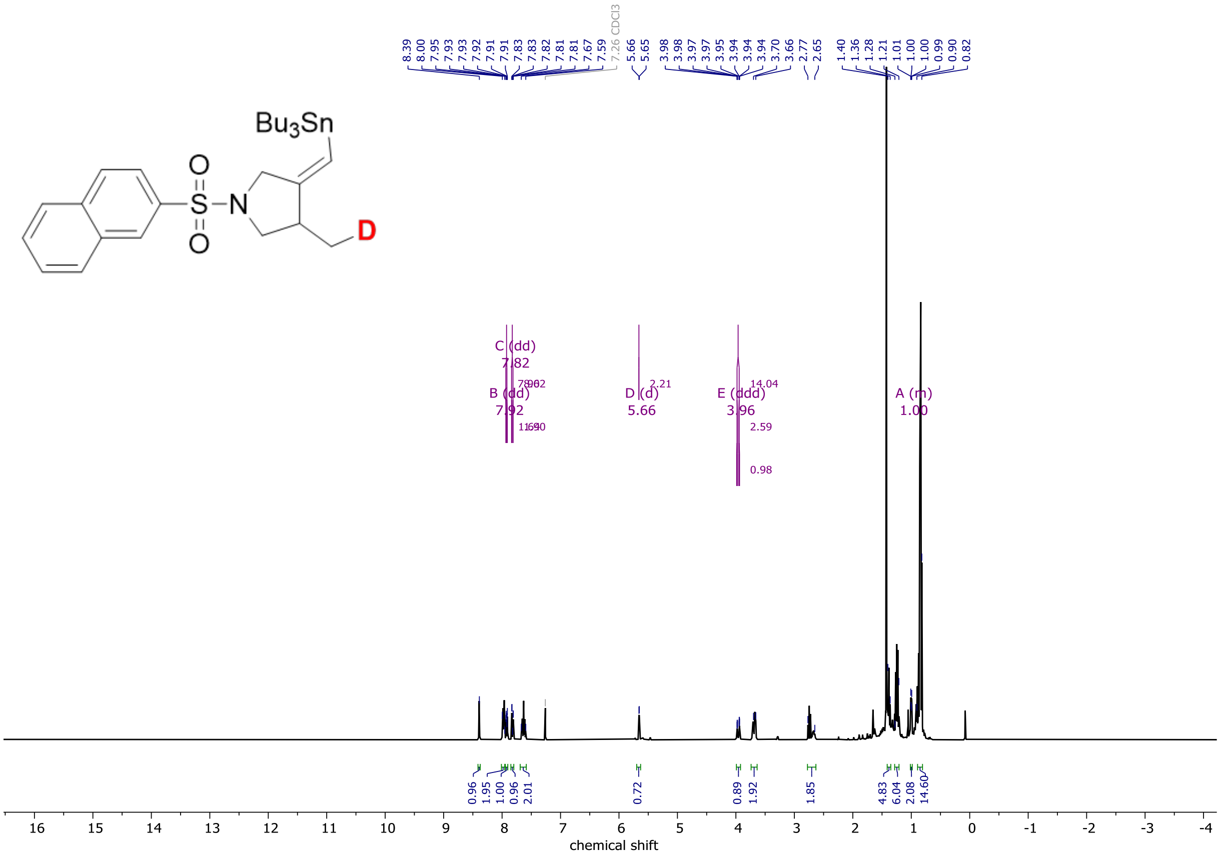
Bu3Sn
D
C (dd)
7.82
B (dd)
7.92
D (d)
5.66
E (ddd)
3.96
A (m)
1.00
chemical shift

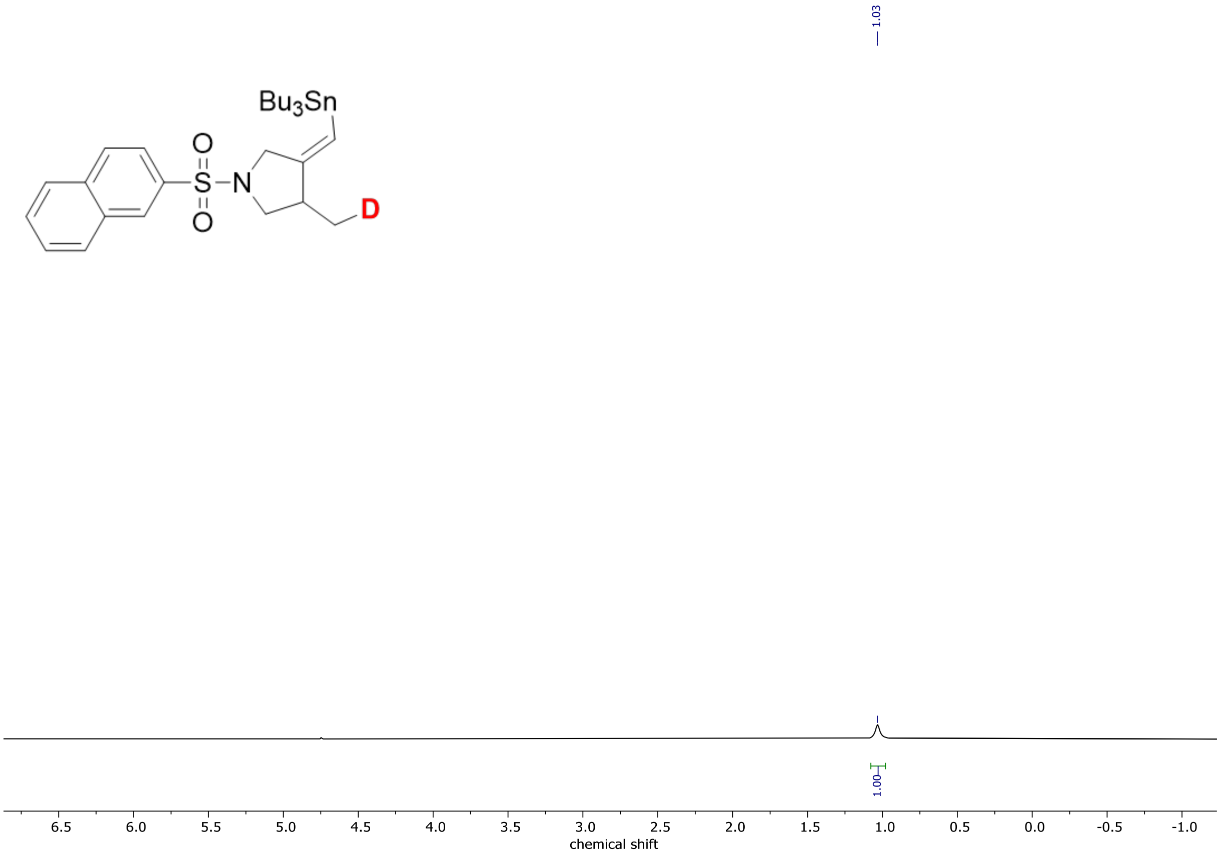
Bu3Sn
D
1.03
1.00
chemical shift

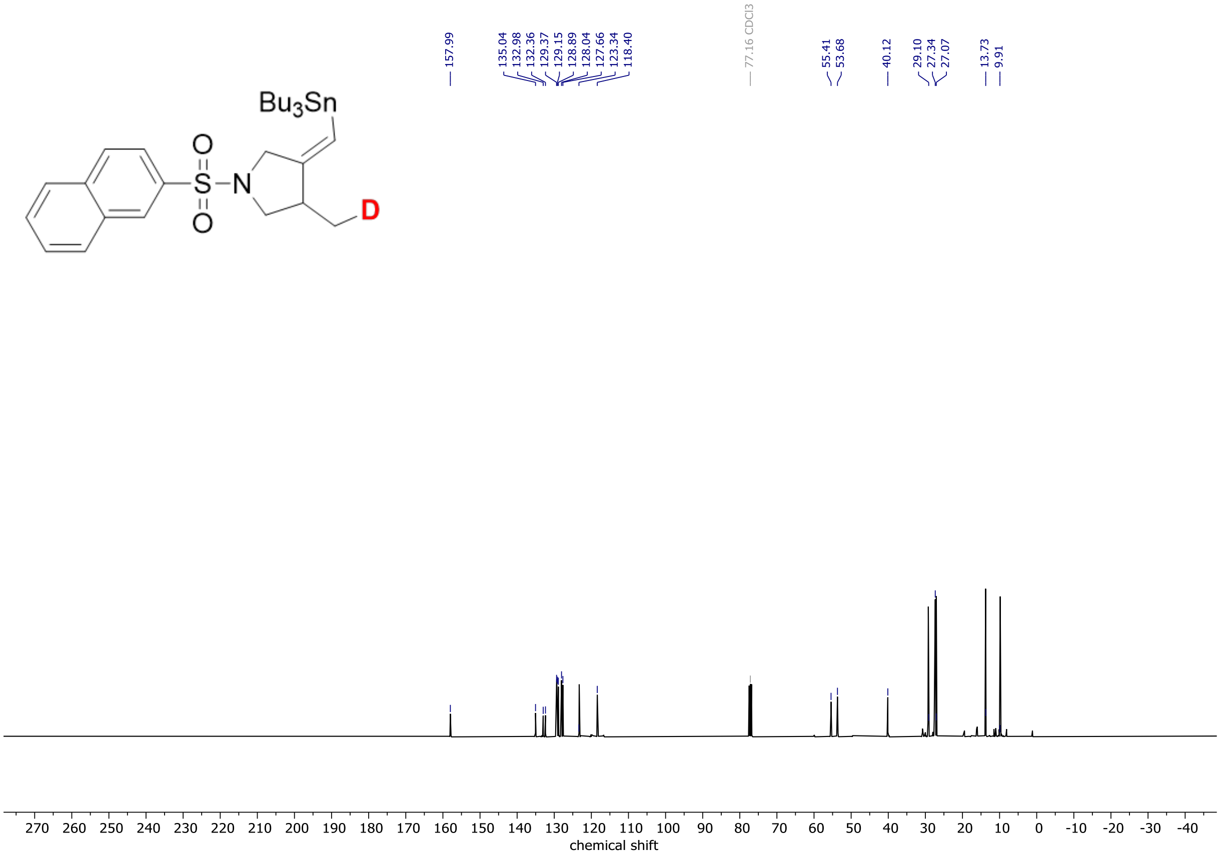
Bu3Sn
O
S
N
O
D
157.99
135.04
132.98
132.36
129.37
129.15
128.89
128.04
127.66
123.34
118.40
77.16 CDCl3
55.41
53.68
40.12
29.10
27.34
27.07
13.73
9.91
270 260 250 240 230 220 210 200 190 180 170 160 150 140 130 120 110 100 90 80 70 60 50 40 30 20 10 0 -10 -20 -30 -40
chemical shift

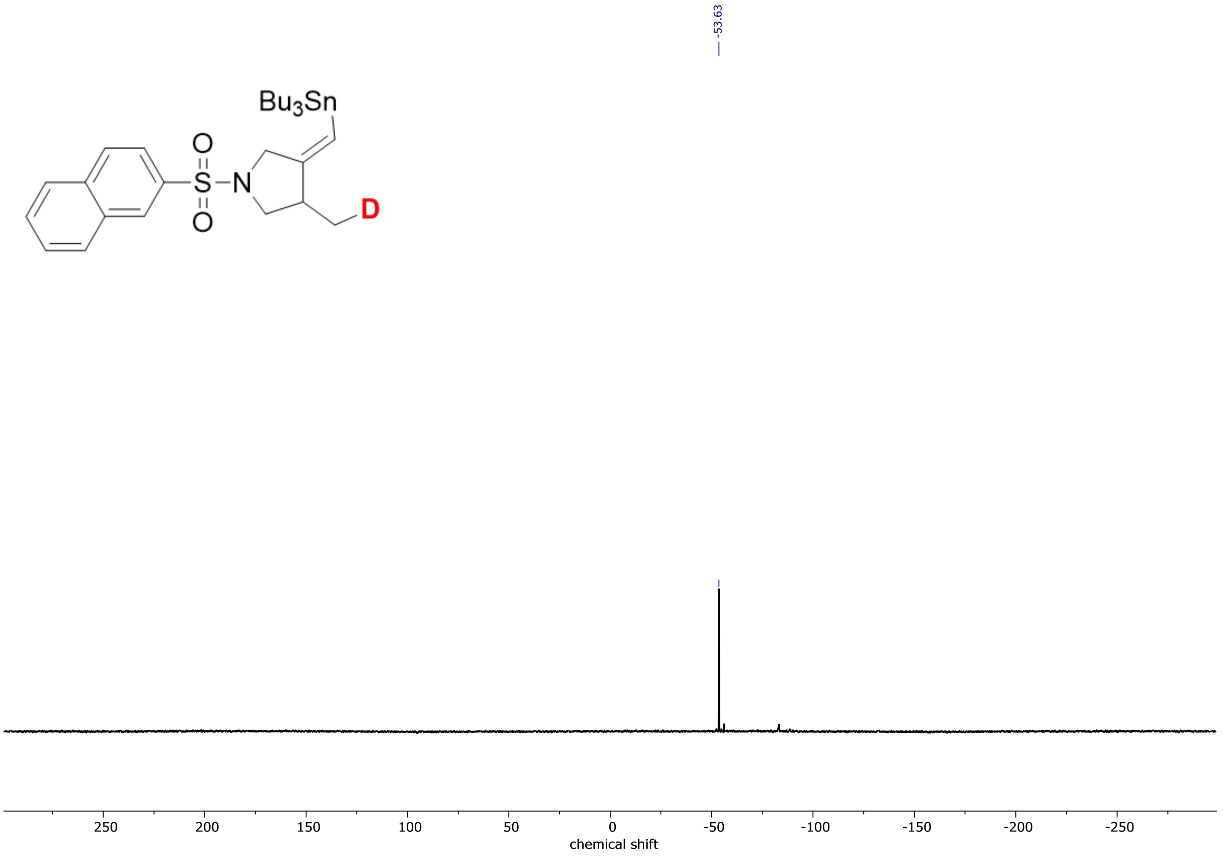
Bu3Sn
O
S
N
O
D
-53.63
250 200 150 100 50 0 -50 -100 -150 -200 -250
chemical shift

References

[1] Y. Lu, S. Yamago, *Angew. Chem. Int. Ed.* **2019**, *58*, 3952-3956.

[2] R. B. Bedford, P. B. Brenner, D. Elorriaga, J. N. Harvey, J. Nunn, *Dalton Trans.* **2016**, *45*, 15811-15817.

[3] K. Jonas, P. Klusmann, R. Goddard, *Zeitschrift für Naturforschung B* **1995**, *50*, 394-404.

[4] O. A. Groß, S. Lauk, C. Müller, W. Gidt, Y. Sun, S. Demeshko, F. Meyer, H. Sitzmann, *Eur. J. Inorg. Chem.* **2017**, *2017*, 3635-3643.

[5] F. Zhang, J. Jia, S. Dong, W. Wang, C.-H. Tung, *Organometallics* **2016**, *35*, 1151-1159.

[6] D. Patel, A. Wooles, A. D. Cornish, L. Steven, E. S. Davies, D. J. Evans, J. McMaster, W. Lewis, A. J. Blake, S. T. Liddle, *Dalton Trans.* **2015**, *44*, 14159-14177.

[7] J. Pornet, D. Damour, L. Miginiac, *Tetrahedron* **1986**, *42*, 2017-2024.

[8] S. Liang, J. Jasinski, G. B. Hammond, B. Xu, *Org. Lett.* **2015**, *17*, 162-165.

[9] D. Daniel, R. Middleton, H. L. Henry, W. H. Okamura, *J. Org. Chem.* **1996**, *61*, 5617-5625.

[10] E. M. Barreiro, E. V. Boltukhina, A. J. P. White, K. K. Hii, *Chem. - Eur. J.* **2015**, *21*, 2686-2690.

[11] A.-C. Bédard, S. K. Collins, *J. Am. Chem. Soc.* **2011**, *133*, 19976-19981.

[12] A. B. Smith, III, J. W. Leahy, I. Noda, S. W. Remiszewski, N. J. Liverton, R. Zibuck, *J. Am. Chem. Soc.* **1992**, *114*, 2995-3007.

[13] A. G. Dalling, G. Späth, A. Fürstner, *Angew. Chem. Int. Ed.* **2022**, *61*, e202209651.

[14] J. E. Baldwin, V. P. Reddy, *J. Am. Chem. Soc.* **1988**, *110*, 8223-8228.

[15] A. S. K. Hashmi, R. Salathé, W. Frey, *Chem. - Eur. J.* **2006**, *12*, 6991-6996.

[16] D. Yamane, H. Tanaka, A. Hirata, Y. Tamura, D. Takahashi, Y. Takahashi, T. Nagamitsu, M. Ohtawa, *Org. Lett.* **2021**, *23*, 2831-2835.

[17] X. Ma, J.-X. Wang, S. Li, K.-H. Wang, D. Huang, *Tetrahedron* **2009**, *65*, 8683-8689.

[18] Q. Wu, J. Hu, X. Ren, J. Zhou, *Chem. Eur. J.* **2011**, *17*, 11553-11558.

[19] D. Campolo, T. Arif, C. Borie, D. Mouysset, N. Vanthuyne, J.-V. Naubron, M. P. Bertrand, M. Nechab, *Angew. Chem. Int. Ed.* **2014**, *53*, 3227-3231.

[20] X. Pan, J. Gao, J. Liu, J. Lai, H. Jiang, G. Yuan, *Green Chem.* **2015**, *17*, 1400-1403.

[21] S. Hou, H. Yang, B. Cheng, H. Zhai, Y. Li, *Chem. Commun.* **2017**, *53*, 6926-6929.

[22] Y. Wang, S. G. Scrivener, X.-D. Zuo, R. Wang, P. N. Palermo, E. Murphy, A. C. Durham, Y.-M. Wang, *J. Am. Chem. Soc.* **2021**, *143*, 14998-15004.

[23] J.-J. Li, C.-G. Wang, J.-F. Yu, P. Wang, J.-Q. Yu, *Org. Lett.* **2020**, *22*, 4692-4696.

[24] S. Pan, F. Chen, Y. Zhang, L. Shao, L. Chu, *Angew. Chem. Int. Ed.* **2023**, *62*, e202305426.

[25] Y. Nishimoto, A. Okita, M. Yasuda, A. Baba, *Org. Lett.* **2012**, *14*, 1846-1849.

[26] Z. Wu, N. Fatuzzo, G. Dong, *J. Am. Chem. Soc.* **2020**, *142*, 2715-2720.

[27] C. Kim, H. J. Bae, J. H. Lee, W. Jeong, H. Kim, V. Sampath, Y. H. Rhee, *J. Am. Chem. Soc.* **2009**, *131*, 14660-14661.
